# HIV Glasgow, 23–26 October 2022, Glasgow, UK / Virtual

**DOI:** 10.1002/jia2.26009

**Published:** 2022-10-21

## ORAL ABSTRACTS

### HIV and Beyond: Planning the Unplanned

#### O11 Clinical perspective on Ukrainian war refugee HIV care in Poland

M Parczewski ^1^, E Jablonowska^2^, K Wójcik‐Cichy^2^, D Zhyvytsia^3^, M Witak‐Jedra^3^, E Siwak^4^, J Kowalska^5^, A Olczak^6^, A Szymczak^7^, M Bogiaga‐Jasik^8^, A Kalinowska‐Nowak^8^, P Jakubowski^9^, M Hlebowicz^9^, B Rozplochowski^10^, W Lojewski^11^, K Scheibe^1^, K Serwin^1^

^1^Infectious, Tropical Diseases and Immune Deficiency, Pomeranian Medical University in Szczecin, Szczecin, Poland; ^2^Department of Infectious Diseases and Hepatology, Medical University of Lódz, Lódz, Poland; ^3^Infectious, Tropical Diseases and Immune Deficiency, Regional Hospital, Szczecin, Poland; ^4^Department of Infection, Tropical Diseases and Hepatology, Medical University in Warsaw, Warsaw, Poland; ^5^Department for Adult Infection Diseases, Medical University in Warsaw, Warsaw, Poland; ^6^Department of Infectious Diseases and Hepatology, Faculty of Medicine, Nicolaus Copernicus University Ludwik Rydygier Collegium, Bydgoszcz, Poland; ^7^Department of Infectious Diseases, Liver Disease and Acquired Immune Deficiencies, Wroclaw Medical University, Wroclaw, Poland; ^8^Department of Infectious and Tropical Diseases, Jagiellonian University Medical College, Kraków, Poland; ^9^Infectious Diseases, Pomeranian Center for Infectious Diseases and Tuberculosis, Gdansk, Poland; ^10^Department of Infectious Diseases, Hepatology and Acquired Immunodeficiencies, Karol Marcinkowski University of Medical Sciences, Poznan, Poland; ^11^Department of Infectious Diseases, Regional Hospital in Zielona Gora, Zielona Góra, Poland

**Background**: War in Ukraine has forced migration for safety, protection and assistance, including medical care. So far >10 million border crossings to the neighbour countries were registered, with Poland being primary refugee sheltering country with over 1.2 million Ukrainian refugees registered for protection scheme. HIV disease burden in Ukraine is disproportionately high with approximately 0.6% prevalence and ∼130 000 antiretroviral (ARV) treated compared to ∼0.1% for Poland with ∼15 000 people on ARV as of March 2022. Since the beginning of war 2252 migrants entered HIV care (13% increase), posing the challenge to provide high‐quality care. Within this study we wished to present Polish clinical experience on the HIV clinical care provided for war migrants from Ukraine.

**Materials and methods**: Clinical, antiretroviral treatment, immunological and virological data from 631 Ukrainian PLWHIV entering care since February 2022 were analysed. The dataset included patients seeking medical assistance due to war‐associated displacement from home country having entered Poland since February 2022, both antiretroviral treated and newly diagnosed with HIV and already living in Poland, HIV diagnosed and treated in Ukraine requiring medical care entry due to restrictions in ARV access. For virologically failing and newly diagnosed cases population protease/reverse transcriptase/integrase sequencing was performed with Stanford HIV‐database used for interpretation of drug resistance (available for 50 cases). HIV subtype was assigned with maximum likelihood method phylogeny.

**Results**: Median age was 40 (IQR 34 to 45) years. Majority (71.0%) of patients were female, with predominance of heterosexual (70.4%) transmissions followed by 13.2% PWID, 6.3% MSM, 1.9% vertical, 0.6% nosocomial and 7.4% undisclosed transmission routes. HCV antibody was present in 29.7%, HBs in 3.1%. 91.8% PLWHIV were diagnosed and initiated ARV in Ukraine with 52 (8.2%) patients HIV diagnosed in Poland, of these 77.3% diagnosed late. At care entry in Poland the most common ARV combinations were tenofovir disoproxil, lamivudine, dolutegravir (TLD) single tablet (n = 443, 79.4%), followed by two nucleoside (2NRTI) plus efavirenz (n = 51, 9.13%) or 2NRTI+dolutegravir (n = 40, 6.3%). Viral load was undetectable (<50 copies/mL) in 89.5%. Majority of patients were antiretroviral switched ‐ most commonly to TDF/FTC +DTG (n = 260, 41.5%) or TAF/FTC/BIC (n = 216, 34.2%). In overall, 86.9% of patients treatment was switched within the antiretroviral class, in 6.6% ARV class was switched while in 6.5% no ARVs were changed. Subtype A6 was the most common (n = 44, 88%), with 14% of NNRTI, 2% of NRTI and PI sequences with resistance mutations but no major integrase resistance.

**Conclusions**: Antiretroviral treatment efficacy in the group of migrants entering care was high, but within‐class treatment switch was necessary due to TLD unavailability. New cases were diagnosed late, while A6 subtype and NNRTI resistance more common than in other European cohorts.

#### O12 New challenges of PrEP implementation during Russian‐Ukrainian war

A Koval ^1^, L Hetman^1^, S Riabokon^1^, A Bilets^1^, T Koval^2^

^1^HIV Department, Public Health Center of the Ministry of Health of Ukraine, Kyiv, Ukraine; ^2^Infectious Diseases, Poltava State Medical University, Poltava, Ukraine

**Background**: Development of prevention programme and PrEP implementation in key population groups are highly important to achieve control of HIV epidemic especially in such a critical period for Ukraine. Despite significant disruptions in the provision of HIV prevention programme in the regions with military actions in Ukraine, through collaborative efforts the Center for Public Health and nongovernment organisations with the support of international partners, implementation of PrEP programme was able to continue in Ukraine. We aimed to assess of recruitment dynamics of new PrEP clients and changes in their geographic distribution in Ukraine during period of military actions.

**Materials and methods**: We analysed recruitment dynamics of new PrEP clients for the first half of 2022 in various regions of Ukraine based on the data of the Center for Public Health. These data were analysed in comparison with the corresponding period in 2021 (Figure 1).

**Figure:**
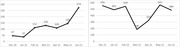
**Abstract O12 – Figure 1**. Dynamics of amount of new PrEP clients in Ukraine in comparison between first half of 2022 and the corresponding period 2021.

**Table:** 

**Abstract O12 – Table 1**. Growth of PrEP clients in regions of Ukraine in comparison between first half of 2022 and the corresponding period 2021.							
Region	Jan 21	Jun 21	Growth of clients (%)	Jan 22	Jun 22	Growth of clients (%)	Growth difference between first half of 2021 and 2022
Western regions							
Volyn	35	43	22.8%	45	62	37.7%	+14.9%
Chernivtsi	9	21	133.3%	22	56	154.5%	+21.2%
Lviv	6	14	133.3%	16	166	937.5%	+804.2%
Regions with active military actions in 2022							
Kyiv	303	439	44.8%	1005	1509	50.1%	+5.3%
Kharkiv	124	191	54.0%	339	402	18.6%	−35.4%
Chernigiv	77	120	55.8%	430	545	26.7%	−29.2%
Symu	7	19	171.4%	38	51	34.2%	−137.2%
Kherson	54	90	66.6%	167	182	8.9%	−57.7%
Zaporizzia	41	64	56.1%	148	179	20.9%	−35.2%
Mukolaiv	23	35	52.2%	128	180	40.6%	−11.6%
Odessa	38	260	584.2%	880	1334	51.6%	−532.6%

**Results**: Amount of new PrEP clients continues to increase from 4510 in January 2022 to 7162 in June 2022, the monthly average fluctuated from 491 to 565 with a significant decline ‐ 185 person in March 2022 after start of military actions in Ukraine. Among new 2593 PrEP clients in 2022, 72.0% were men, with a predominance of the age group 30 to 45 years (65.6%). Participants self‐identified as MSM ‐ 41.4%, PWID – 16.5%, sexual partners of HIV‐infected people ‐ 33.8%, sex workers – 2.0%, others – 6.3%. Growth of PrEP clients in central regions of Ukraine kept at the same level by maintaining in social support and dispensing PrEP to clients in advance. In regions with active military actions in 2022 significant decline in growth of new PrEP clients was revealed – in Kherson district ‐57.7%, in Symu ‐137.2%, in Odessa ‐532.6% due to the migration of clients from these regions. In Western region a significant increase of growth of new PrEP clients was found due internally displaced clients, predominantly MSM (Table 1).

**Conclusions**: During period of military action in Ukraine in the first half of 2022, amount of new PrEP clients continues to increase with predominance of the growth of new PrEP clients in the Western regions.

### HIV and Sexual Health

#### O21 Laboratory analysis of HIV infections in the year 1 unblinded period of HPTN 083: injectable cabotegravir for PrEP in MSM and TGW

M Marzinke^1^, B Grinsztejn^2^, J Fogel^1^, E Piwowar‐Mann^1^, B Hanscom^3^, Z Wang^4^, C Petropoulos^5^, E Halvas^6^, J Mellors^7^, P Anderson^8^, O Sued^9^, S Chariyalertsak^10^, H Scott^11^, K Mayer^12^, R Arduino^13^, R Kofron^14^, M Cohen^15^, M St. Clair^16^, A Rinehart^17^, J Rooney^18^, A Adeyeye^19^, M McCauley^20^, S Eshleman^1^, R Landovitz ^14^

^1^Medicine, Johns Hopkins University, Baltimore, MD, USA; ^2^Instituto de Pesquisa Clinica Evandro Chagas‐Fiocruz, Rio de Janeiro, Brazil; ^3^Vaccine and Infectious Disease, Fred Hutchinson Cancer Research Center, Seattle, WA, USA; ^4^Statistical Center for HIV/AIDS Research and Prevention, Fred Hutchinson Cancer Research Center, Seattle, WA, USA; ^5^Research and Development, Labcorp, San Francisco, CA, USA; ^6^Medicine, University of Pittsburgh, Pittsburgh, PA, USA; ^7^Immunology, University of Pittsburgh, Pittsburgh, PA, USA; ^8^Pharmacy, University of Colorado, Aurora, CO, USA; ^9^Fundación Huésped, Buenos Aires, Argentina; ^10^Research Institute for Health Sciences, Chiang Mai University, Chiang Mai, Thailand; ^11^San Francisco Department of Public Health, San Francisco, CA, USA; ^12^Medicine, Harvard Medical School, Boston, MA, USA; ^13^Internal Medicine, McGovern Medical School, Houston, TX, USA; ^14^Medicine, University of California, Los Angeles, Los Angeles, CA, USA; ^15^Medicine, The University of North Carolina at Chapel Hill, Chapel Hill, NC, USA; ^16^Virology, ViiV Healthcare, Durham, NC, USA; ^17^Global HIV Prevention Strategy, ViiV Healthcare, Durham, NC, USA; ^18^Medical Affairs, Gilead Sciences, San Mateo, CA, USA; ^19^Division of AIDS, National Institutes of Health, Bethesda, MD, USA; ^20^FHI 360, Durham, NC, USA

HPTN 083 showed a 66% reduction in HIV incidence in cisgender men and transgender women (MSM/TGW) assigned to cabotegravir (CAB) injections versus daily oral tenofovir disoproxil fumarate/emtricitabine (TDF/FTC) for pre‐exposure prophylaxis (PrEP). We previously characterized 58 HIV infections from the blinded study period. We now present virology and pharmacology findings for 52 additional cases that occurred up to 1 year after study unblinding. Concentrations of CAB and tenofovir (TFV) in plasma and TFV‐diphosphate in dried blood spots were quantified by liquid chromatography‐tandem mass spectrometry.  Timing of HIV infection was assessed using an antigen/antibody test, a discriminatory test and RNA assays. Drug resistance testing was performed using a commercial assay (viral load [VL] >500 copies/mL) and a low VL genotyping assay (VL <500 copies/mL). We identified three additional incident infections that occurred in the blinded study phase (one in the CAB arm, two in the TDF/FTC arm). The new CAB infection occurred despite on‐time CAB injections. In the first year after unblinding, we identified 49 incident infections (17 in the CAB arm, 32 in the TDF/FTC arm). The CAB arm infections included one with on‐time injections, three with at least one delayed injection, and 13 with no recent CAB dosing. Plasma CAB concentrations were generally as expected. Major integrase strand transfer inhibitor (INSTI) resistance associated mutations (RAMs) were observed in three cases (one case with R263K, two cases with Q148R); in one case, a major INSTI RAM emerged after CAB PrEP was restarted in a person with undiagnosed infection. Both of the newly identified infections that occurred in persons with on‐time CAB injections had diagnostic delays using conventional HIV testing algorithms. Diagnostic delays were also observed in one case where CAB was restarted after infection and in one case with no recent CAB exposure. In the newly‐identified cases, major INSTI RAMs were observed with on‐time injections and with re‐initiation of CAB after infection. To date, most cases with HIV acquisition >6 months after the last CAB injection in MSM/TGW did not have HIV diagnostic delays; no major INSTI RAMs were observed in these cases unless CAB PrEP was restarted after infection.

### HIV Clinical Challenges (I)

#### O22 Use of preventive measures for cardiovascular disease in people living with HIV

N Jaschinski ^1^, B Neesgaard^1^, F Wit^2^, M van der Valk^3^, H Günthard^4^, M Stöckle^5^, E Wallner^6^, J Kowalska^7^, A Ridolfo^8^, P Nowak^9^, A Castagna^10^, A d'Arminio Monforte^11^, N Chkhartishvili^12^, K Petoumenos^13^, J Hoy^14^, H Garges^15^, J Rooney^16^, L Young^17^, S Hosein^18^, J Lundgren^1^, L Peters^1^, A Mocroft^19^, L Ryom^20^

^1^University of Copenhagen, CHIP, Rigshospitalet, Copenhagen, Denmark; ^2^Stichting HIV Monitoring, AIDS Therapy Evaluation in the Netherlands (ATHENA) cohort, Amsterdam, Netherlands; ^3^University of Amsterdam, Division of Infectious Diseases, AIDS Therapy Evaluation in the Netherlands (ATHENA) cohort, Stichting HIV Monitoring; Amsterdam University Medical Centers, Amsterdam, Netherlands; ^4^Department of Infectious Diseases, Swiss HIV Cohort Study (SHCS), University of Zurich; University Hospital Zurich, Zurich, Switzerland; ^5^Division of Infectious Diseases and Hospital Epidemiology, University Hospital of Basel, University of Basel, Swiss HIV Cohort Study (SHCS), University of Zurich, Basel, Switzerland; ^6^Department für Gastroenterologie, Infektiologie, Pneumologie, Landeskrankenhaus Graz II, Standort West, Graz, Austria; ^7^Infectious Diseases, Hospital for Infectious Diseases in Warsaw, Warsaw, Poland; ^8^III Infectious Diseases Unit, ASST Fatebenefratelli‐Sacco, Milano, Italy; ^9^Swedish InfCare HIV Cohort, Karolinska University Hospital, Stockholm, Sweden; ^10^San Raffaele Scientific Institute, Università Vita‐Salute San Raffaele, Milano, Italy; ^11^Italian Cohort Naive Antiretrovirals (ICONA), ASST Santi Paolo e Carlo, Milano, Italy; ^12^Infectious Diseases, AIDS and Clinical Immunology Research Center, Georgian National AIDS Health Information System (AIDS HIS), Tbilisi, Georgia; ^13^The Australian HIV Observational Database (AHOD), University of New South Wales, Sydney, Australia; ^14^HIV Medicine, The Alfred Hospital and Monash University, Melbourne, Australia; ^15^RTP, ViiV Healthcare, Durham, NC, USA; ^16^Gilead Sciences, Foster City, CA, USA; ^17^Merck Sharp & Dohme, Kenilworth, NJ, USA; ^18^European AIDS Treatment Group (EATG), Brussels, Belgium; ^19^Centre for Clinical Research, Epidemiology, Modelling and Evaluation (CREME), Institute for Global Health, University College London, UK; and CHIP, Rigshospitalet, University of Copenhagen, Copenhagen, Denmark; ^20^Hvidovre University Hospital, Department of Infectious Diseases 144, CHIP, Rigshospitalet, University of Copenhagen, Copenhagen, Denmark

**Background**: While cardiovascular disease (CVD) contributes significantly to morbidity and mortality in people living with HIV (PLWHIV), data on the uptake of preventive measures for CVD are limited.

**Materials and methods**: We included participants from the multinational RESPOND cohort in whom an estimated 10‐year D:A:D CVD risk could be calculated. We determined the annual prevalence (1 July 2012 to 1 July 2019) of preventive measures use for those with a very high (>10%) estimated CVD risk and eligible for each specific measure. Binomial regression with robust standard errors assessed factors associated with the uptake of each preventative measure.

**Figure:**
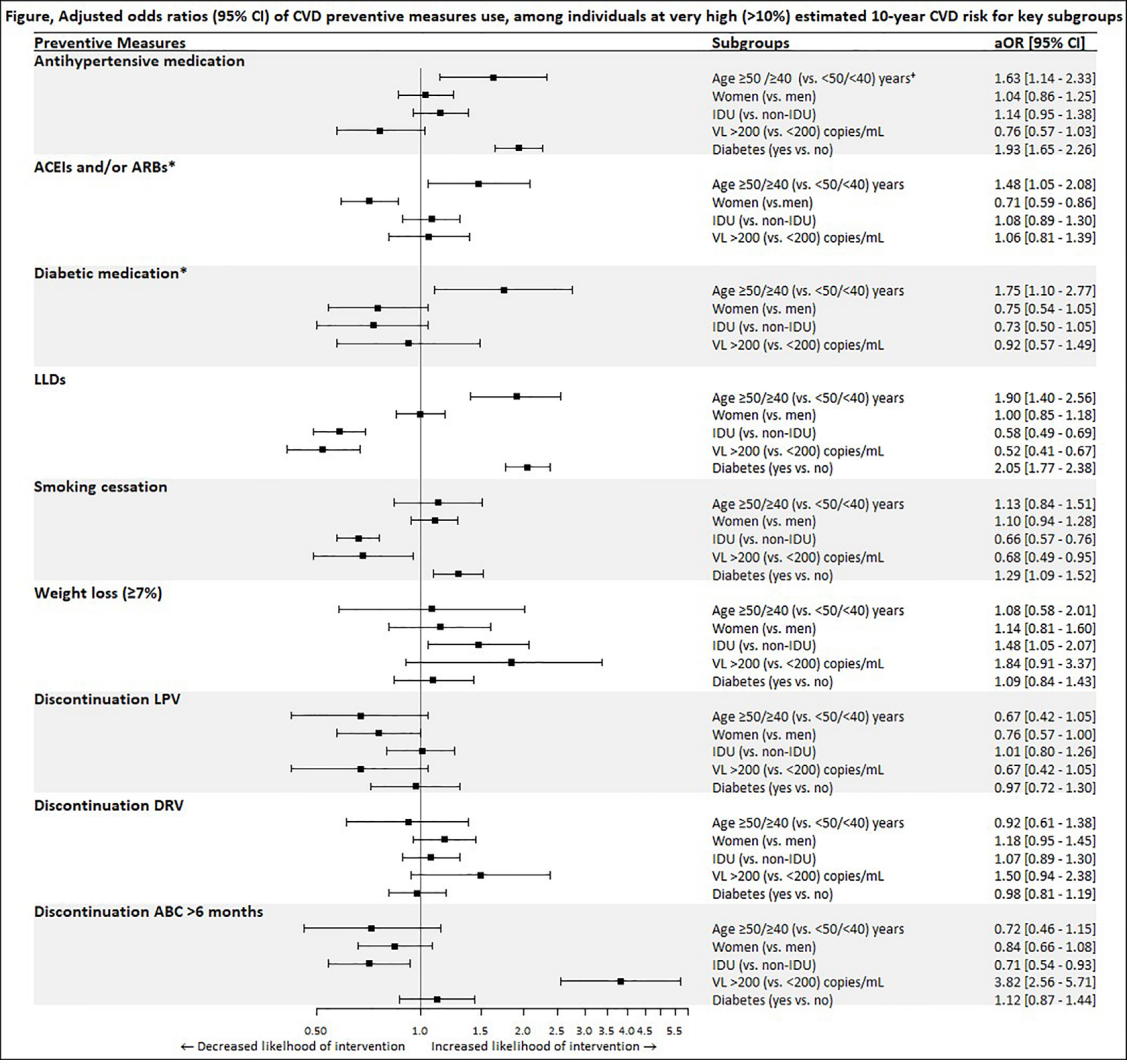
**Abstract O22 – Figure 1**. aOR adjusted for age (<40/≥40 men, <50/≥50 women), gender, ethnicity, CVD risk region, body mass index (*not included for weight loss*), HIV acquisition risk, CD4 cell count, CD4 nadir, hypertension (*not included for antihypertensives and ACEIs/ARBs*), diabetes (*not included for diabetic medication and ACEIs/ARBs*), AIDS, cancer, chronic kidney disease, dyslipidaemia (*not included for LLDs*), calendar year, current smoking (*not included for smoking cessation*), cumulative exposure to LPV, DRV, and IDV, ABC use in the past 6 months, and INSTIs exposure. Apart from ACEIs/ARBs (where use was more likely), use of CVD preventive measures was similar in individuals with a body mass index ≥30 (vs <30 kg/m^2^). Individuals with dyslipidaemia were significantly more likely to use antihypertensives, ACEIs/ARBs and antidiabetics. Use of antihypertensives, antidiabetics and LLDs was less likely in current smokers (vs non‐smokers). ^+^ Age subgroup: <40/≥40 years of age for men, <50/≥50 for women; ^*^ not including ACEIs/ARBs and antidiabetics since diabetes is part of their eligibility criteria. ABC, abacavir; ACEIs, angiotensin‐converting enzyme inhibitors; aOR, adjusted odds ratio; ARBs, angiotensin receptor blockers; CVD, cardiovascular disease; DRV, darunavir; IDU, intravenous drug use; INSTI, integrase strand transfer inhibitor; LLDs, lipid‐lowering drugs; LPV, lopinavir.

**Results**: The crude proportion with >10% estimated 10‐year CVD risk increased from 31.5% (4144/13 146) in 2012 to 45.0% (7187/15 964) in 2019 (p < 0.0001). In 2019, in those at very high risk, 65.6% (1462/2229) with hypertension received antihypertensives, 56.4% (1520/2696) with dyslipidaemia received lipid‐lowering drugs (LLDs) and 41.8% (1055/2523) with diabetes/hypertension used angiotensin‐converting enzyme inhibitors (ACEIs)/angiotensin receptor blockers (ARBs). Equally, 8.1% (178/2199) of smokers ceased smoking and 10.6% (48/454) with BMI >30 kg/m^2^ lost ≥7% bodyweight. We found no significant changes over time in the use of any of these measures (2012 to 2019; all multivariate p > 0.05). While fewer diabetics received antidiabetics in later years (2012; 63.5% [382/602] vs 2019; 56.7% [435/767]), discontinuation of darunavir (7.5% [49/650] vs 12.1% [85/702]), lopinavir (15.2% [90/593] vs 26.7% [23/86]) and abacavir (2.5% [34/1337] vs 7.9% [120/1515]) increased among individuals using these drugs (all multivariate p < 0.01). In multivariable analyses (Figure 1), older individuals (≥40 years for men, ≥50 for women vs <40/<50) were more likely to use antihypertensives, ACEIs/ARBs, antidiabetics and LLDs. Individuals with diabetes (vs those without) were also more likely to use antihypertensives, LLDs and cease smoking. In contrast, LLD use and smoking cessation were less likely in those with a viral load ≥200 copies/mL (vs <200) and intravenous drug use (IDU) as HIV acquisition risk (vs non‐IDU). Besides women being less likely to receive ACEIs/ARBs, the use of preventive measures was similar between genders.

**Conclusions**: Despite an increased proportion of individuals at very high estimated CVD risk, CVD preventive measures were underused in RESPOND. Our findings call for greater awareness of management guidelines for CVD risk factors in PLWHIV.

#### O23 Risk of tuberculosis after initiation of antiretroviral therapy among people living with HIV in Europe

I Johansen ^1^, A Roen^2^, O Kirk^3^, on behalf of the RESPOND study group

^1^Department of Infectious Diseases, Odense University Hospital, Odense, Denmark; ^2^Centre for Clinical Research, Epidemiology, Modelling and Evaluation (CREME), Institute for Global Health, UCL, London, UK; ^3^CHIP, Centre of Excellence for Health, Immunity and Infections & Department of Infectious Diseases, Rigshospitalet, University of Copenhagen, Copenhagen, Denmark

**Background**: Tuberculosis (TB) is the most frequent HIV‐/AIDS‐related cause of deaths worldwide. TB preventive treatment (TPT) is recommended for people with HIV (PWHIV) irrespective of the degree of immunosuppression or antiretroviral treatment (ART) status. However, the benefit of TPT in a low TB/HIV incidence setting is unclear and in many European countries, latent TB infection and TPT is not routinely used.

**Materials and method**: PWHIV first starting ART after 2012 within the RESPOND cohort were included and followed until the first of TB diagnosis, death, last visit, or December 2020. TB incidence rates (IR) were assessed for consecutive time intervals (0 to 3, 3 to 6, 6 to 12, and >12 months), and risk factors for developing TB within 6 months of initiating ART were evaluated using Cox proportional hazards models.

**Figure:**
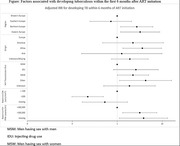
**Abstract O23 – Figure 1**. Factors associated with developing tuberculosis within the first 6 months after ART initiation.

**Results**: Among 8241 adult PWHIV who started ART, 66 TB events were diagnosed during 34 239 person‐years of follow‐up (PYFU), corresponding to an IR of 1.93/1000 PYFU (95% CI 1.51 to 2.45). TB IR were 14.76 (10.32 to 21.12), 6.04 (3.43 to 10.63), 2.61 (1.40 to 4.85) and 0.53 (0.31 to 0.90)/1000 PYFU in the intervals 0 to 3, 3 to 6, 6 to 12, and >12 months after ART initiation, respectively. Independent risk factors for TB within the first 6 months after ART initiation were follow‐up in Northern or Eastern Europe, African origin, baseline CD4 count <200 cells/mm^3^, and HIV‐RNA >100 000 copies/mL (or missing HIV‐RNA), whereas men having sex with men were at lower risk of TB compared with other HIV transmission risk categories (Figure 1). Among 24 PWHIV with TB ≥6 months after ART initiation, 12 were diagnosed while being well treated (latest CD4 count >200 cells/mm^3^ and HIV‐RNA <100 copies/mL). Of the 24, 10 were diagnosed in Eastern Europe and five of these had IDU as transmission risk.

**Conclusion**: Overall, TB incidence rates were substantially higher in the first 3 months after initiation of ART. This highlights the need for a thorough TB risk assessment before starting ART. The risk of TB was lower after 12 months of ART, but remained higher than in the general population in most European countries. This supports directing strategies of careful diagnostics and TPT towards PWHIV with clear TB risk factors.

### Joep Lange and Jacqueline van Tongeren Memorial Lecture – Advances in Treatment of Cryptococcal Meningitis

#### KL1 Advances in treatment of cryptococcal meningitis

Joe Jarvis

Tropical Medicine and International Health, London School of Hygiene and Tropical Medicine, Gaborone, Botswana

Cryptococcal meningitis remains a leading killer of people living with HIV, causing an estimated 19% of all AIDS‐related deaths globally. The vast majority of these deaths occur in sub‐Saharan Africa, where to date treatment has been based on fluconazole monotherapy or prolonged courses of amphotericin B deoxycholate. Fluconazole monotherapy leads to acute mortality rates in excess of 60%, and 2‐week courses of amphotericin B deoxycholate are associated with frequent and severe drug‐related toxicities. New strategies for the management of cryptococcal meningitis are urgently needed if UNAIDS targets to end the AIDS epidemic by 2030 are to be met. Fortunately, there have been several major research advances in the management of HIV‐associated fungal infections over the past decade; notably the development of a screen‐and‐treat approach to prevent the development of HIV‐associated cryptococcal meningitis using novel highly sensitive antigen tests, and the discovery of highly effective short‐course treatments for CM based on optimising the pharmacokinetics of existing antifungal drugs. For the potential of these recent advances to be realised, it is essential that rigorous implementation work is undertaken to translate the clinical research findings into meaningful patient outcomes and ensure that context‐specific interventions are developed and applied. This talk will present the evidence underpinning these recent advances and discuss the implementation challenges faced and the work being undertaken to address them.

### The Evidence for Same Day ARV Therapy

#### O24 Rapid ART initiation using BIC/FTC/TAF and TDF+3TC+EFV in people with HIV in China: a randomised control trial

S Lv, L Sun, W Hua, A Li, L Dai

No. 8, West Toutiao, outside You'anmen, Fengtai District, Youan Hospital, Beijing, China

**Background**: Most guidelines recommend rapid antiretroviral therapy (rapid ART) for newly diagnosed HIV‐1‐infected individuals, but related data are still limited in China. This study analysed efavirenz 400 mg + lamivudine 300 mg + tenofovir disoproxil fumarate 300 mg (EFV+3TC+TDF) and bictegravir/emtricitabine/tenofovir alafenamide (BIC/FTC/TAF) for efficacy and safety.

**Figure:**
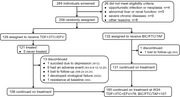
**Abstract O24 – Figure 1**. Study flowchart.

**Table:** 

**Abstract O24 – Table 1**. Baseline patient characteristics.			
Characteristic	Group A (n = 126)	Group B (n = 132)	p‐value^a^
Age (year), IQR	29 (25.0 to 35.0)	29 (25.0 to 38.3)	0.488
Race (Han), n (%)	109 (96.5)	110 (90.2)	0.056
BMI (kg/m^2^), IQR	23.2 (20.2 to 25.3)	22.9 (20.3 to 24.8)	0.552
Number of comorbidities >0, n (%)	11 (18.3)	12 (18.7)	0.952
Number of medications >0, n (%)	10 (16.7)	12 (18.7)	0.762
HBs antigen positive, n (%)	7 (6.3)	6 (5.0)	0.679
HCV antibody positive, n (%)	1 (0.9)	1 (0.8)	0.966
RPR positive, n (%)	22 (22.4)	31 (30.7)	0.188
Baseline viral load (lg copies/mL), IQR	4.4 (4.0 to 4.8)	4.3 (3.8 to 5.0)	0.444
Viral load >100 000 (copies/mL), n (%)	19 (18.4)	27 (25.0)	0.249
Baseline CD4 count (cells/μL), IQR	342 (243.0 to 448.6)	342 (241.0 to 449.6)	0.913
Baseline CD4 count <200 cells/uL, n (%)	22 (17.9)	22 (17.3)	0.907
CD4/CD8, IQR	0.3 (0.2 to 0.5)	0.4 (0.2 to 0.5)	0.779
Time from diagnosis to treatment (day), IQR	6 (3.0 to 8.0)	5 (2.0 to 7.0)	0.064

^a^Continuous and categorical variables were compared by the Mann‐Whitney U test and the chi‐square test, respectively.

**Methods**: This was a national, randomised open clinical trial. We enrolled HIV‐1 infected adult (age ≥18 years) men who have sex with men (MSM) who started ART within 14 days after HIV diagnosis confirmation. The participants were randomly assigned (1:1) to the EFV+3TC+TDF and BIC/FTC/TAF groups. The primary endpoint was the percentage of patients with successful viral suppression (<50 copies/mL) after 24 weeks; secondary endpoints included viral load, CD4 count, changes in weight and blood lipids, and cohort retention after 12 weeks, 24 weeks, 36 weeks and 48 weeks.

**Results**: A total of 258 participants, including 126 and 132 in the EFV and BIC groups were enrolled, respectively, across eight sites in China from March 2021 to April 2022 (Table 1). In the EFV group, 68 (74.7%) participants were retained in care with a 24‐week HIV‐1 RNA load <50 copies and 13 were discontinued (11.2%) because of AEs, death or lost to follow‐up. In the BIC group, 101 (93.5%) participants were retained with HIV‐1 RNA load <50 copies/mL and one (0.8%) was lost to follow‐up (Figure 1). Viral suppression rate was higher in the BIC group than in the EFV group per FDA Snapshot (93.5% vs 74.7%, p < 0.001). In the EFV group, CD4 count increased from 356.3 [268.5 to 463.6] to 459 [373.6 to 612.5] cells/mm^3^; in the BIC group, CD4 count increased from 338.3 [239.5 to 441] to 476 [329.8 to 625.5] cells/mm^3^ after 12 weeks. Follow‐up will continue to 48 weeks.

**Conclusion**: These results suggested that BIC/FTC/TAF was safe and effective in rapid ART.

### HIV Clinical Challenges (II)

#### O31 Older people with well‐controlled HIV have similar antibody and higher T‐cell responses after vaccination against SARS‐CoV‐2 compared to demographically and lifestyle‐comparable people without HIV

M Verburgh ^1^, L van Pul^2^, M Grobben^3^, A Boyd^4^, F Wit^1^, A van Nuenen^2^, K van Dort^2^, K Tejjani^3^, J van Rijswijk^3^, M Bakker^3^, L van der Hoek^3^, M Schim van der Loeff^5^, M van der Valk^1^, M van Gils^3^, N Kootstra^2^, P Reiss^1^

^1^Infectious Diseases, Amsterdam UMC, University of Amsterdam, Amsterdam, Netherlands; ^2^Experimental Immunology, Amsterdam UMC, University of Amsterdam, Amsterdam, Netherlands; ^3^Medical Microbiology and Infection Prevention, Laboratory of Experimental Virology, Amsterdam UMC, University of Amsterdam, Amsterdam, Netherlands; ^4^Infectious Diseases, HIV Monitoring Foundation, Amsterdam, Netherlands; ^5^Infectious Diseases, Public Health Service of Amsterdam, Amsterdam, Netherlands

**Background**: Studies comparing humoral and cellular SARS‐CoV‐2 vaccine responses in people with HIV (PWHIV) and demographically and lifestyle‐comparable HIV‐negative controls are scarce.

**Figure:**
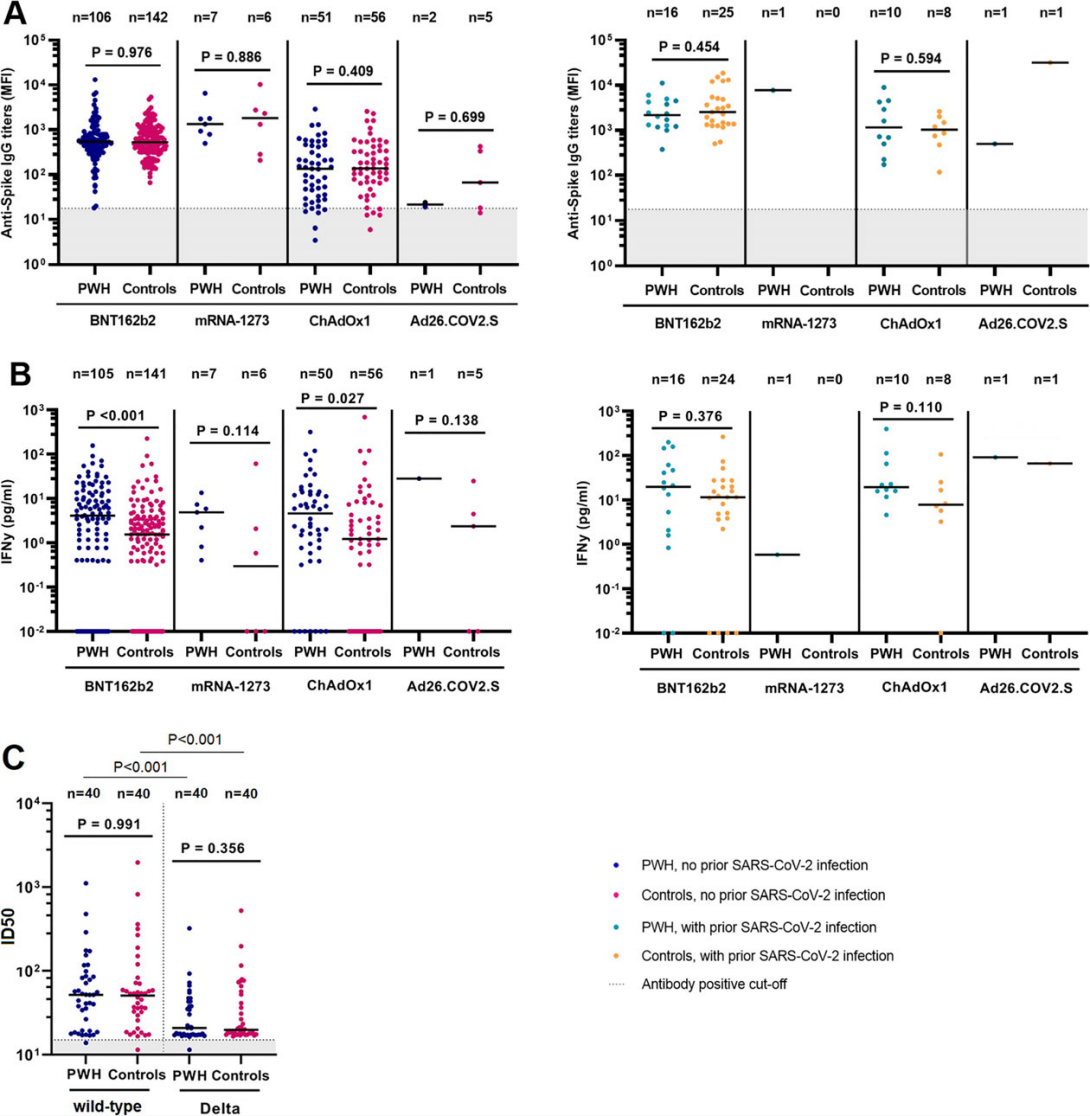
**Abstract O31 – Figure 1**. Post‐vaccination SARS‐CoV‐2 IgG spike‐antibody titers, T‐cell responses and neutralisation against wild‐type and Delta variants in participants of the AGEhIV COVID‐19 substudy. P‐values, comparing people with HIV (PWHIV) and HIV‐negative controls, were calculated using Wilcoxon rank‐sum test. (A) Post‐vaccination SARS‐CoV‐2 IgG spike‐antibody titers in participants without (left panel) and with (right panel) prior SARS‐CoV‐2 infection, by HIV‐status and vaccine type. Resulting values are expressed as the median fluorescence intensity (MFI) of at least 50 beads per antigen. The dotted line represents the antibody non‐response cut‐off value (IgG S‐antibody titer <17.8 MFI). (B) Post‐vaccination SARS‐CoV‐2 T‐cell responses in participants without (left panel) and with (right panel) prior SARS‐CoV‐2 infection, by HIV‐status and vaccine type. Resulting values are expressed as the IFNγ release (in pg/mL, lower detection limit 0.09 pg/mL). (C) Post‐vaccination virus‐neutralisation against SARS‐CoV‐2 wild‐type and Delta variants in 40 PWHIV and 40 controls (1:1 matched on age, sex and vaccine type). Resulting values are expressed as the serum dilution at which 50% of the infectivity was inhibited (ID50).

**Table:** 

**Abstract O31 – Table 1**. Characteristics of 441 included participants in the AGEhIV COVID‐19 substudy (at the time of 4 to 13 weeks after last dose of a COVID‐19 vaccine), by HIV‐status. All values are n (%) or median (interquartile range). A) Last available data prior to receiving last vaccine dose of the primary vaccination course. B) HIV‐1 viral load missing in 1/195 PWHIV.			
	People with HIV (n = 195)	Controls (n = 246)	p
Age, yr	63.3 (58.7 to 68.3)	61.6 (58.0 to 67.5)	0.145^a^
Male sex at birth	184 (94.4%)	211 (85.8%)	0.003^b^
Ethnic origin ‐ Caucasian ‐ African ‐ Asian	‐ 190 (97.4%) ‐ 5 (2.6%) ‐ 0 (0.0%)	‐ 237 (96.4%) ‐ 4 (1.6%) ‐ 5 (2.0%)	0.109^c^
BMI, kg/m^2^ (A) ‐ Underweight (<18.5) ‐ Normal weight (18.5 to 24.9) ‐ Overweight (25.0 to 29.9) ‐ Obese (30.0)	‐ 1 (0.5%) ‐ 101 (51.8%) ‐ 72 (36.9%) ‐ 21 (10.8%)	‐ 0 (0.0%) ‐ 118 (48.0%) ‐ 97 (39.4%) ‐ 31 (12.6%)	0.608^c^
Total comorbidities (A) ‐ 0 comorbidities ‐ 1 to 2 comorbidities ‐ 3 to 7 comorbidities	‐ 79 (40.5%) ‐ 96 (49.2%) ‐ 20 (10.3%)	‐ 154 (62.6%) ‐ 78 (31.7%) ‐ 14 (5.7%)	<0.001^b^
Current CD4 count, cells/mm^3^ (A)	640 (500 to 850)	810 (650 to 1010)	<0.001^a^
Current CD4 count (A) ‐ <350 cells/mm^3^ ‐ 350 to 499 cells/mm^3^ ‐ 500 to 749 cells/mm^3^ ‐ 750 cells/mm^3^	‐ 19 (9.8%) ‐ 26 (13.3%) ‐ 77 (39.5%) ‐ 73 (37.4%)	‐ 3 (1.2%) ‐ 21 (8.5%) ‐ 72 (29.3%) ‐ 150 (61.0%)	<0.001^c^
Current CD8 count, cells/mm^3^ (A)	750 (500 to 990)	410 (300 to 560)	<0.001^a^
Current CD8 count (A) ‐ <350 cells/mm^3^ ‐ 350 to 499 cells/mm^3^ ‐ 500 to 749 cells/mm^3^ ‐ 750 cells/mm^3^	‐ 22 (11.3%) ‐ 24 (12.3%) ‐ 50 (25.6%) ‐ 99 (50.8%)	‐ 88 (35.8%) ‐ 73 (29.6%) ‐ 42 (17.1%) ‐ 43 (17.5%)	<0.001^c^
Current CD4/8 ratio (A)	0.86 (0.65 to 1.22)	1.87 (1.32 to 2.56)	<0.001^a^
Current CD4/8 ratio (A) ‐ <0.50 ‐ 0.50 to 0.99 ‐ 1.0	‐ 21 (10.8%) ‐ 98 (50.2%) ‐ 76 (39.0%)	‐ 0 (0.0%) ‐ 21 (8.5%) ‐ 225 (91.5%)	<0.001^c^
Time since HIV diagnosis, yr	22.6 (17.1 to 27.9)	NA	…
Time since first starting ART, yr	19.8 (13.9 to 24.7)	NA	…
CD4 nadir, cells/mm^3^	180 (70 to 260)	NA	…
Undetectable HIV‐1 viral load (A, B)	193 (99.5%)	NA	…
COVID‐19 vaccine type ‐ BNT162b2 ‐ mRNA‐1273 ‐ ChAdOx1 ‐ Ad26.COV2.S ‐ ChAdOx1+BNT162b2	‐ 122 (62.6%) ‐ 8 (4.1%) ‐ 61 (31.3%) ‐ 3 (1.5%) ‐ 1 (0.5%)	‐ 167 (67.9%) ‐ 6 (2.4%) ‐ 64 (26.0%) ‐ 6 (2.4%) ‐ 3 (1.2%)	0.491^c^
Only one dose of BNT162b2, mRNA‐1273 or ChAdOx1 due to prior SARS‐CoV‐2 infection	2 (1.0%)	8 (3.3%)	0.284^c^
Days between pre‐vaccination sample and first vaccine dose	44 (26 to 67)	45 (28 to 74)	0.702^a^
Days between first and second vaccine dose ‐ BNT162b2 ‐ mRNA‐1273 ‐ ChAdOx1 ‐ ChAdOx1 + BNT162b2	‐ 35 (35 to 36) ‐ 28 (28 to 32) ‐ 77 (63 to 77) ‐ 88 (88 to 88)	‐ 36 (35 to 36) ‐ 32 (28 to 36) ‐ 76 (68 to 77) ‐ 65 (33 to 113)	‐ 0.096^a^ ‐ 0.459^a^ ‐ 0.538^a^ ‐ 0.655^a^
Days between last vaccine dose and post‐vaccination sample	64 (46 to 76)	70 (43 to 77)	0.262^a^
Prior SARS‐CoV‐2 infection ‐ prior to pre‐vaccination sample ‐ between pre‐ and post‐vaccination sample	‐ 20 (10.3%) ‐ 8 (4.1%)	‐ 25 (10.2%) ‐ 9 (3.7%)	0.877^b^

BMI, body mass index; NA, not applicable; yr, in years.

^a^Wilcoxon rank‐sum test;

^b^Pearson χ2 test;

^c^Fisher's exact test.

**Methods**: SARS‐CoV‐2‐spike(S)‐IgG antibody (custom Luminex immunoassay) and T‐cell responses (IFNγ release upon S‐peptide stimulation) were measured in last available stored samples prior to vaccination and 4 to 13 weeks after completing primary vaccination from PWHIV and HIV‐negative Amsterdam AGEhIV COVID‐19 substudy participants [1]. A positive nucleocapsid‐antibody test (INgezim IgA/IgM/IgG) or self‐reported positive PCR defined prior SARS‐CoV‐2 infection. Factors associated with post‐vaccination IgG S‐titers and T‐cell responses were assessed by multivariable linear and tobit regression, respectively, grouping vaccines as mRNA‐ or vector‐based. In 2 x 40 age‐/sex‐/vaccine type‐matched PWHIV and controls without prior SARS‐CoV‐2, virus‐neutralisation (wild‐type and Delta variants) was determined on VeroE6 cells by cytotoxicity‐assay.

**Results**: Characteristics of 195 enrolled PWHIV and 246 controls include a similar distribution of vaccines received (Table 1). Both pre‐ and post‐(Figure 1A) vaccination IgG S‐titers, regardless of vaccine type, did not significantly differ between groups. Pre‐ and post‐(Figure 1B) vaccination T‐cell responses were higher in PWHIV. HIV‐status was not associated with IgG S‐titer. Prior SARS‐CoV‐2 infection (β = 0.77), mRNA vaccine (β = 0.56), female sex (β = 0.24) and fewer days between last vaccination and sampling (β = 0.07) were significantly associated with higher, and a CD4/8 ratio<1.0 with lower (β = ‐0.39) IgG S‐titers, without significant interactions between HIV‐status and any of these factors. Prior SARS‐CoV‐2 infection (β = 0.97), HIV‐positive status (β = 0.63) and fewer days between last vaccination and sampling (β = 0.10) were associated with higher T‐cell responses, after adjusting for pre‐vaccination levels. SARS‐CoV‐2‐neutralisation was not significantly different between the subgroup of PWHIV and controls, but significantly reduced for the Delta variant in both groups (Figure 1C).

**Conclusions**: Total and neutralising antibody responses to SARS‐CoV‐2 vaccines did not differ significantly, whereas the T‐cell response was increased in these older PWHIV with well‐controlled HIV compared to demographically and lifestyle‐similar individuals without HIV. Factors affecting the height of response were similar in both groups. Interestingly, this included a lower CD4/8 ratio being associated with an overall lower antibody response in both PWHIV and controls. Further analyses will explore potential relationships with immune senescence and functionality of the T‐cell response.

**Reference**

1. Verburgh ML, Boyd A, Wit FWNM, Schim van der Loeff MF, van der Valk M, Bakker M, et al. Similar risk of severe acute respiratory syndrome coronavirus 2 infection and similar nucleocapsid antibody levels in people with well‐controlled human immunodeficiency virus (HIV) and a comparable cohort of people without HIV. J Infect Dis. 2022;225:1937‐47.

#### O32 External validation of the Dat'AIDS score for predicting 5‐year mortality among elderly people with HIV in the Swiss HIV Cohort Study

M Hentzien ^1^, J Frossard^1^, R Kouyos^2^, V Prendki^3^, J Damas^4^, E Hofmann^5^, D Braun^6^, P Schmid^7^, E Bernasconi^8^, S Ragozzino^9^, O Efthimiou^10^, C Delpierre^11^, C Allavena^12^, F Bani‐Sadr^13^, A Calmy^1^

^1^HIV/AIDS Research Unit, Geneva University Hospitals, Geneva, Switzerland; ^2^Institute of Medical Virology, University of Zurich, Zurich, Switzerland; ^3^Division of Internal Medicine for the Aged, Geneva University Hospitals, Geneva, Switzerland; ^4^Infectious Diseases Service, University Hospital Lausanne, University of Lausanne, Lausanne, Switzerland; ^5^Department of Infectious Diseases, Bern University Hospital, University of Bern, Bern, Switzerland; ^6^Division of Infectious Diseases and Hospital Epidemiology, University Hospital Zurich, University of Zurich, Zurich, Switzerland; ^7^Division of Infectious Diseases, Cantonal Hospital St. Gallen, St. Gallen, Switzerland; ^8^Division of Infectious Diseases, Ospedale Regionale di Lugano (Ente Ospedaliero Cantonale), and University of Southern Switzerland, Lugano, Switzerland; ^9^Department of Infectious Diseases and Hospital Epidemiology, University Hospital Basel, Basel, Switzerland; ^10^Institute of Social and Preventive Medicine (ISPM), University of Bern, Bern, Switzerland; ^11^Unité Mixte de Recherche (UMR) 1027, Institut National de la Santé et de la Recherche Médicale, Toulouse, France; ^12^Infectious Diseases Department, Institut National de la Santé et de la Recherche Médicale (INSERM), Equipe d'Accueil (EA) 1413, Centre Hospitalier Universitaire, Nantes, France; ^13^Department of Internal Medicine, Clinical Immunology and Infectious Diseases, Reims University Hospital, Reims, France

**Background**: People living with HIV (PLWHIV) are ageing and adapted mortality prognostic indexes are needed in this future predominant population. The Dat'AIDS score includes age, comorbidities (non‐HIV related cancer, cardiovascular diseases, estimated glomerular filtration rate, cirrhosis and anaemia), low body mass index and HIV‐specific variables (CD4 cell count). It has been derived and internally validated in PLWHIV aged 60 years and over and allows the discrimination of four risk groups ranging from low to very high risk with the very high risk group having an expected 54% probability of 5‐year survival. The score showed good discrimination and calibration in a single French cohort but has never been externally validated.

**Table:** 

**Abstract O32 – Table 1**. Hazard ratios across risk groups of the Dat'AIDS score.			
Characteristic	HR	95% CI	p‐value
Moderate (4 to 13 points) vs low risk (0 to 3 points)	2.53	1.65 to 3.88	<0.001
High (14 to 19 points) vs moderate risk (4 to 13 points)	2.55	1.63 to 3.98	<0.001
Very high (≥20 points) vs high risk (14 to 19 points)	1.72	1.00 to 2.96	0.048

HR, hazard ratio.

**Methods**: The Dat'AIDS score was calculated at the first follow‐up after 1 January 2015 for all PLWHIV aged ≥60 years actively followed in the Swiss HIV Cohort Study. Survival times were evaluated until 1 January 2020. The score's prognostic capacity was evaluated by fitting a Cox model. Its discrimination capacity was first assessed using the Harrell C‐statistic on the selected population and subgroups by gender, age, HIV viral load, CD4 and CD4 nadir strata, and then by calculating hazard ratios between adjacent risk groups. Calibration was assessed by comparing observed and expected survival.

**Results**: Among 2212 PLWHIV (1801 males; 411 females) included, 144 deaths were recorded. Mean CD4 cell count was 621±296 /mm^3^; 92.7% had a baseline HIV viral load <50 copies/mL. Mean observed Dat'AIDS score was 5.1±6.5 and ranged from 0 to 46. Using the validation dataset, the Cox model on the Dat'AIDS score confirmed good prognostic capacities (hazard ratio 1.09; 95% CI 1.07 to 1.11; p < 0.001). Discrimination was good, as the overall Harrell C‐statistic was 0.73 (95% CI 0.69 to 0.77), similar to the derivation dataset, and ranged from 0.71 to 0.78 across subgroups. Hazard ratios across pre‐defined risk groups showed a higher probability of death for higher predicted risk (Table 1) as well as good calibration (Figure 1).

**Conclusion**: The Dat'AIDS score showed good external validity to predict the 5‐year survival, with an excellent discrimination and calibration, and will allow careful clinical monitoring in the most fragile patients.

**Figure:**
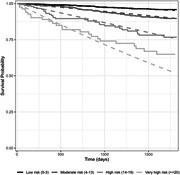
**Abstract O32 – Figure 1**. Calibration of the Dat'AIDS score in the validation dataset. Expected (dashed lines) versus observed (solid lines) survival probability.

#### O33 COCOVIH study: impact of comorbidities on the over‐mortality of people living with HIV

F Prevoteau du Clary ^1^, C Majerholc^2^, D Zucman^3^, J Livrozet^4^, B Guigui^5^, A Vallee^6^, C Laurendeau^7^, S Bouee^7^

^1^Centre de Santé Sexuelle, La Grave Hospital, University Hospital Toulouse, Toulouse, France; ^2^Medecine Interne, University Hospital Toulouse, Toulouse, France; ^3^Medecine Interne, Foch Hospital, Suresnes, France; ^4^Service de Maladies Infectieuses et Tropicales, Edouard Herriot Hospital, Lyon, France; ^5^Departement de Medecine Generale, Université Paris Cité, Paris, France; ^6^Department of Epidemiology ‐ Data ‐ Biostatistics, Foch Hospital, Suresnes, France; ^7^Public Health and Epidemiology, CEMKA‐EVAL, Bourg La Reine, France

**Background and objectives**: Efficacious treatments prevent immunodeficiency and opportunistic infection in people living with HIV (PLWHIV). However, PLWHIV have more frequent other chronic conditions such as cardiovascular diseases, cancer, due to the infection itself or to side effects of antiviral treatments. Moreover, some patients remain untreated because of unknown reasons. The objectives of this study were to estimate mortality of PLWHIV and the impact of other conditions on the over‐mortality.

**Methods**: COCOVIH draws upon anonymised records from the national health database SNDS, which includes >90% of the French population registered in CNAM (National Health Insurance Fund). A cohort of PLWHIV and age‐ and gender‐matched controls was extracted from the French National Healthcare System Database (SNDS). PLWHIV were identified between 2006 and 2019 and followed up until 2019. The incidence of deaths was estimated and compared between both groups. Comorbidities were identified through classical algorithms used in this database (ICD‐10 codes, specific drugs or procedures etc.). A Cox model was used to estimate the increased risk of deaths. Impact of comorbidities was estimated by adjusting on them.

**Results**: 173 712 PLWHIV and controls were followed up 8 years on average. Mean age at inception was 42 years and 66% were males. Significant increase of death rates was found in PLWHIV with a HR of 2.1 (CI 95% 2.0 to 2.2). This HR was 1.961 (CI 95% 1.898 to 2.027) for men and 2.966 (CI 95% 2.767 to 3.180) for women. The HR was higher in young PLWHIV: 3.5 (18 to 30‐year‐old subjects), 3.7 (30 to 40), 2.9 (40 to 50), 1.7 (50 to 60), 1.5 (60 to 70), 1.4 (70 to 80). Infectious diseases had the higher impact on the over‐mortality: the HR decreased from 2.1 to 1.6 after adjusting on infectious diseases, hence an attributable risk (AR) of 50%. The other conditions were: hepatitis C (AR 30%), psychiatric diseases (AR 16%), hepatitis B (AR 6%), coronary diseases (4%), and phlebitis/pulmonary embolism (4%). Other studied diseases had ARs below 3% (Table 1).

**Conclusion**: HIV infection doubles the risk of death and infectious diseases explain half of this over‐mortality. The relative over‐mortality is higher among women and young patients.

**Table:** 

**Abstract O33 – Table 1**. Hazard ratio (HR) in different categories of subjects according to age, gender and adjusted on comorbidities.				
		HR	CI 95%	CI 95%
CRUDE HR	Overall	2.1	2.0	2.2
CRUDE HR	Male	1.961	1.898	2.027
CRUDE HR	Female	2.966	2.767	3.180
CRUDE HR	[18‐30] years	3.517	2.704	4.574
CRUDE HR	[30‐40] years	3.664	3.331	4.03
CRUDE HR	[40‐50] years	2.896	2.75	3.05
CRUDE HR	[50‐60] years	1.705	1.61	1.806
CRUDE HR	[60‐70] years	1.483	1.375	1.600
CRUDE HR	[70‐80] years	1.379	1.246	1.527
CRUDE HR	≥80 years	1.691	1.4	2.041
HR Adjusted on comorbidities	Infectious diseases	1.587	1.538	1.638
HR Adjusted on comorbidities	Hepatitis C	1.791	1.736	1.847
HR Adjusted on comorbidities	Psychiatric diseases	1.950	1.893	2.009
HR Adjusted on comorbidities	Coronary diseases	2.086	2.025	2.149
HR Adjusted on comorbidities	Hepatitis B	2.063	2.002	2.126
HR Adjusted on comorbidities	Phlebitis/pulmonary embolism	2.089	2.028	2.152
HR Adjusted on comorbidities	Peripheral artery disease	2.105	2.043	2.168
HR Adjusted on comorbidities	Kidney diseases	2.134	2.072	2.199
HR Adjusted on comorbidities	All above comorbidities (multivariate model)	1.333	1.290	1.377

### Lock Lecture – PrEP vs PEP Interface: Where Are We Now?

#### KL2 PrEP vs PEP interface: where are we now?

Sheena McCormack

Clinical Epidemiology, Imperial College, London, UK

In a mess! Multiple randomised controlled clinical trials have successfully demonstrated the biological efficacy of pre‐exposure prophylaxis (PrEP) whether administered as an oral tablet, in a vaginal gel or ring, or as a long‐acting injectable. Single antiretroviral agents have proved effective in diverse populations when used correctly and consistently, and an event‐based regimen of TDF‐FTC with only four tablets per event has provided protection in men who have sex with men in a randomised placebo‐controlled trial. The most common recommendation for post‐exposure prophylaxis globally continues to be 28 days of a three‐drug regimen. This is based on expert opinion after review of animal models, observational studies, vertical transmission and surveillance data. Randomised controlled trials are considered unethical. Antiretrovirals do not ***prevent*** transmission which is rapid (∼30 minutes), but work with innate immune responses to prevent an established infection in the few days that follow exposure. The window of opportunity is uncertain as the replication cycle is highly variable depending on the level of activation in the cells, but it seems highly likely that it lies in the short period before virus is detectable in the blood which is ∼5 days. At this point viral replication is generalised and preventing an established infection must be impossible. PrEP users are in a position to start a two‐drug PEPSE regimen within hours of exposure ‐ they should be enabled to do so as missed pills prior to sex is common. How long they should continue for is a conundrum, but 28 days makes little sense. Whether or not a third drug is required is dubious. A randomised controlled trial of a shorter course of two‐drug PEP following sexual exposure is planned!

### Novel Therapeutics

#### O34 VH3810109 (N6LS) reduces viremia across a range of doses in ART‐naive adults living with HIV: proof of concept achieved in the phase IIa BANNER (207959, NCT04871113) study

P Leone ^1^, A Ferro^2^, C Rolle^3^, S Lupo^4^, J McGowan^5^, M Klein^6^, P Cahn^7^, P Benson^8^, M Sanchez^9^, C Bettacchi^10^, S Schneider^11^, P Wannamaker^12^, B Win^13^, J Abberbock^14^, M Baker^15^, V Wilches^16^, D Bentley^17^, M Gartland^12^, M Lataillade^18^, J Losos^19^

^1^Clinical Development, ViiV Healthcare, Durham, NC, USA; ^2^Infectious Diseases, Centro de Investigaciones Medicas, Mar del Plata, Argentina; ^3^Infectious Diseases, Orlando Immunology Center, Orlando, FL, USA; ^4^Infectious Diseases, Centro de Asistencia e Investigación Clínica Integral (CAICI), Rosario, Argentina; ^5^Infectious Diseases, Northwell Health, New York, NY, USA; ^6^McGill University Health Centre, Montreal, Quebec, Canada; ^7^Infectious Diseases, Fundacion Huesped, Buenos Aires, Argentina; ^8^Infectious Diseases, Be Well Medical Center, Berkley, MI, USA; ^9^Infectious Diseases, Hospital Italiano de Buenos Aires, Buenos Aires, Argentina; ^10^Infectious Diseases, North Texas Infectious Disease Consultants, Dallas, TX, USA; ^11^Infectious Diseases, Long Beach Education and Research Consultants, Long Beach, CA, USA; ^12^Clinical Development, ViiV Healthcare, Research Triangle Park, NC, USA; ^13^Safety Evaluation and Risk Management (SERM), GSK, Brentford, UK; ^14^Statistics, GSK, Upper Providence, PA, USA; ^15^Clinical Pharmacology, GSK, Brentford, UK; ^16^Clinical Development, GSK, Upper Providence, PA, USA; ^17^Clinical Pharmacology, Certara, Sheffield, UK; ^18^Global Research Strategy, ViiV Healthcare, Branford, CT, USA; ^19^Early Development, ViiV Healthcare, Durham, NC, USA

**Background**: Broadly neutralizing antibodies (bNAbs) are being developed for long‐acting HIV‐1 therapy. VH3810109 is a CD4‐binding site antibody with broad and potent neutralization activity in vitro.

**Materials and methods**: BANNER is a randomized, open‐label, two‐part, multicenter study in treatment‐naive viremic adults to evaluate safety, pharmacokinetics, and antiviral activity of VH3810109. VH3810109 was evaluated during monotherapy after a single intravenous (IV) infusion of 40 mg/kg or 280 mg (∼4 mg/kg) followed by 48 weeks of standard‐of‐care antiretroviral therapy. Monotherapy duration was determined by either virologic non‐response (viral load [VL] <0.5 log10 by day 11) or rebound (VL ≥1.0 log10 over nadir or <0.5 log10 from baseline). Antibody susceptibility was determined retrospectively, using the PhenoSense monoclonal antibody assay. Here we report first‐time antiviral activity during monotherapy and cumulative ongoing safety.

**Results**: Fourteen participants enrolled (United States: n = 6; Canada: n = 1; Argentina: n = 7): 13 were male, median (range) age was 30 (18 to 54) years, 21% were Black, and median (range) baseline VL was 4.31 (3.13 to 5.24) log10 copies/mL. Virologic response was observed in 13 participants; median (range) viral nadir from baseline was 1.72 (0.60 to 2.60) and 1.18 (0.30 to 2.18) log10 copies/mL for 40 mg/kg and 280 mg, respectively (Figure 1). Nine participants experienced 35 adverse events (AEs): 28 grade 1, seven grade 2, 0 grade 3/grade 4/serious AEs, six drug‐related (abdominal pain [n = 2], gastrointestinal pain, pruritis, asthenia, myalgia [n = 1 each]). Pharmacokinetics demonstrated an ∼10‐fold difference in exposure between doses.

**Figure:**
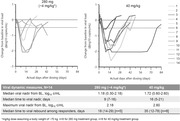
**Abstract O34 – Figure 1**. Change from baseline in plasma HIV‐1 RNA following a single infusion of VH3810109.

**Conclusions**: A single IV infusion of VH3810109 was well tolerated, with few drug‐related AEs and robust antiviral efficacy at both doses. When administered at 40 mg/kg, VH3810109 led to decline in viremia consistent with antiviral activity reported for other bNAbs. VL decline and duration of response observed with the ∼10‐fold lower dose of ∼4 mg/kg exceeded efficacy reported for other bNAbs at similarly low doses. These data warrant further development, including alternate dosing options and modalities for VH3810109.

### Mini Oral Session

#### MO41 Durable efficacy of switching from a three‐/four‐drug tenofovir alafenamide (TAF)‐based regimen to the two‐drug regimen dolutegravir/lamivudine (DTG/3TC) in the TANGO study through week 196

S De Wit^1^, F Bonnet^2^, O Osiyemi^3^, F Bisshop^4^, J Olalla^5^, J Routy ^6^, C Wyen^7^, R Moodley^8^, K Pappa^9^, R Wang^10^, J Oyee^11^, P Saggu^11^, E Letang^12^, B Wynne^9^, B Jones^13^, K Smith^14^, M Ait‐Khaled^8^

^1^Infectious Diseases, Centre Hospitalier Universitaire Saint‐Pierre, Université Libre de Bruxelles, Brussels, Belgium; ^2^Infectious Diseases, Centre Hospitalier Universitaire de Bordeaux, Service de Médecine Interne et Maladies Infectieuses, Bordeaux, France; ^3^Infectious Diseases, Triple O Research Institute PA, West Palm Beach, FL, USA; ^4^Infectious Diseases, Holdsworth House Medical Brisbane, Brisbane, Queensland, Australia; ^5^Infectious Diseases, Unidad de Medicina Interna, Hospital Costa del Sol, Marbella, Spain; ^6^Infectious Diseases, McGill University Health Centre, Montreal, Canada; ^7^Infectious Diseases, Praxis am Ebertplatz, Cologne, Germany; ^8^Clinical Development, ViiV Healthcare, Brentford, UK; ^9^Clinical Development, ViiV Healthcare, Durham, NC, USA; ^10^Translation Medicine, ViiV Healthcare, Durham, NC, USA; ^11^Statistics, GSK, Brentford, UK; ^12^Global Medical Affairs, ViiV Healthcare, Madrid, Spain; ^13^Global Medical Affairs, ViiV Healthcare, Brentford, UK; ^14^Global Research Strategy, ViiV Healthcare, Durham, NC, USA

**Background**: Switching to DTG/3TC has demonstrated durable and non‐inferior efficacy versus continuing three‐/four‐drug regimens for maintaining virologic suppression in people living with HIV‐1 through week (W) 48 in SALSA and W144 in TANGO. Efficacy and safety at W196 from TANGO, for those who were virologically suppressed on TAF‐based regimens at baseline and switched to DTG/3TC at W148 and for those who switched to DTG/3TC at day 1, are presented.

**Table:** 

**Abstract MO41 – Table 1**. Summary of TANGO study outcomes at week 196.				
	ES DTG/3TC		LS DTG/3TC	
	(N = 369)		(N = 298)	
Parameter, n (%)	Day 1 to week 48	Day 1 to week 144	Day 1 to week 196	Week 148 to week 196
Efficacy outcomes (Snapshot, ITT‐E population)				
HIV‐1 RNA <50 copies/mL	344 (93)	317 (86)	306 (83)	278 (93)
HIV‐1 RNA ≥50 copies/mL	1 (<1)	1 (<1)	3 (<1)	0 (0)
No virologic data	24 (7)	51 (14)	60 (16)	20 (7)
Key safety outcomes (safety population)				
Any AE	295 (80)	336 (91)	347 (94)	239 (80)
AEs leading to withdrawal	13 (4)	23 (6)	25 (7)	9 (3)
Drug‐related grade 2 to 5 AEs	17 (5)	21 (6)	23 (6)	11 (4)
SAEs	21 (6)	57 (15)	65 (18)	15 (5)
Fatal AEs	1 (<1)	3 (<1)	4 (1)	0 (0)
Confirmed virologic withdrawals (all screened participants)	0 (0)	0 (0)	1 (<1)^a^	0 (0)

AE, adverse event; DTG/3TC, dolutegravir/lamivudine; ES, Early‐Switch; LS, Late‐Switch; SAE, serious AE.

^a^No resistance‐associated mutations were observed.

**Materials and methods**: TANGO evaluated efficacy and safety of switching to DTG/3TC from stable TAF‐based regimens in virologically suppressed adults (HIV‐1 RNA <50 copies/mL for >6 months) with no prior virologic failure. Participants were stratified by baseline third agent class and randomized 1:1 to switch to DTG/3TC at day 1 (Early‐Switch [ES] group) or continue TAF‐based regimens for 144 weeks. Those continuing TAF‐based regimens and maintaining virologic suppression at W144 switched to DTG/3TC at W148 (Late‐Switch [LS] group). Efficacy through W196 was analyzed via Snapshot algorithm (ITT‐E population). Clinical safety and laboratory toxicity were also evaluated.

**Results**: Overall, 369 participants switched to DTG/3TC at day 1 (ES) and 298 switched to DTG/3TC at W148 (LS). A high proportion of the ES group maintained virologic suppression through year 4, with few new safety events between W144 and W196 (Table 1). After 48 weeks of DTG/3TC, the LS group at W196 and the ES group at W48 had comparable proportions of participants with virologic suppression and similar safety profiles (Table 1). Through W144, no DTG/3TC participants met confirmed virologic withdrawal (CVW) criteria versus three TAF‐based regimen participants. Post‐W144, no LS group participants and one ES group participant met CVW criteria at W196. No resistance‐associated mutations were observed for any CVW.

**Conclusions**: Switching from three‐/four‐drug TAF‐based regimens to the two‐drug regimen DTG/3TC showed durable efficacy, high barrier to resistance, and good tolerability through 4 years, with few new safety events between years 3 and 4. W196 efficacy and safety in the LS group were consistent with W48 data in the ES group. These results support DTG/3TC as a robust and well‐tolerated treatment alternative to three‐/four‐drug TAF‐based regimens with fewer antiretroviral agents for maintaining virologic suppression.

#### MO42 Impact of switch towards 3TC/dolutegravir on the intact and total viral reservoir in the Rumba study

E Blomme^1^, W Trypsteen^1^, M Delporte^1^, C Muccini^2^, E De Smet^1^, S Degroote^3^, E Tobback^3^, S Vanherrewege^3^, E Caluwé^3^, M De Scheerder^3^, L Vandekerckhove ^1^

^1^Department of Internal Medicine and Pediatrics, HIV Cure Research Center, Ghent University, Ghent, Belgium; ^2^Department of Infectious Diseases, IRCCS San Raffaele Scientific Institute, Milan, Italy; ^3^Department of Internal Medicine, Ghent University Hospital, Ghent, Belgium

**Background**: Dual therapy with 3TC/dolutegravir (Dovato^®^) has been thoroughly evaluated in several switch clinical trials using plasma viral load as a primary endpoint (50c/mL threshold) showing non‐inferiority to 3‐drug regimen and is now part of the EACS and other global  guidelines as first‐line regimen for antiretroviral therapy (ART)‐naïve and ART‐experienced adults with HIV. We report the week 48 results of Rumba, the first randomized clinical trial evaluating the impact on the viral reservoir of switch from a 2nd generation integrase inhibitor (INI)‐based triple ART regimen towards Dovato^®^ versus Biktarvy^®^.

**Materials and methods**: One hundred and thirty‐four people living with HIV were included at the Ghent HIV reference centre, with HIV‐1 RNA <50 copies/mL plasma and at least 3 months on any stable second‐generation INI‐based triple ART. Participants were randomised 2:1 to switch to Dovato^®^ (N = 89) or to switch or stay on Biktarvy^®^ (N = 45). After blood collection at baseline and W48, CD4+ T cells were isolated from peripheral blood mononuclear cells (EasySep Human CD4+ T cell isolation kit, Stemcell), followed by DNA extraction (DNeasy Blood&Tissue kit, Qiagen). Total and intact proviral HIV‐1 DNA copies were quantified in triplicate using a digital PCR assay combining the cross‐subtype intact proviral DNA assay [1] and the total HIV‐1 DNA assay [2] (Qiacuity, Qiagen). *RPP30* was measured for normalisation based on cell input. Data analysis was performed with the ddpcrRquant algorithm.

**Results**: Of the 134 patients randomised in the study, 120 reached the W48 primary endpoint (Dovato^®^ N = 80, Biktarvy^®^ N = 40). Patient dropouts due to non‐virological reasons were similar between the two study arms. Baseline levels of total and intact HIV‐1 DNA are presented in Table 1. At 48 weeks, a similar decline of 131.26 (‐397.41 to ‐93.83) and 112.18 (‐219.06 to 42.78) total HIV‐1 DNA copies/million CD4+ T cells and of 6.47 (‐30.00 to 15.87) and 4.65 (‐18.67 to 14.94) intact HIV‐1 DNA copies/million CD4+ T cells was observed in the Dovato^®^ and Biktarvy^®^ group respectively.

**Table:** 

**Abstract MO42 – Table 1**. Patient characteristics.			
Baseline	Total N = 134	Dovato^®^ N = 89	Biktarvy^®^ N = 45
Dropouts after randomisation	13/134	9/89	5/45
Sex (M/F)	118/12	79/8	39/4
Age (year), median (IQR)	46 (37 to 54) n = 128	46 (36 to 53) n = 86	45 (40 to 56) n = 42
CD4 at screening (cells/μL), median (IQR)	689 (550.5 to 929.5) n = 125	691 (558 to 933) n = 83	676.5 (526.75 to 871.75) n = 42
CD4 nadir (cells/μL), median (IQR)	289 (168 to 424) n = 123	296.5 (165.75 to 449) n = 84	273 (194 to 385) n = 39
Peak viral load (copies/mL plasma), median (IQR)	97 646.5 (26 736.73 to 323 510.5) n = 114	122 563 (32 291.7 to 405 526.8) n = 76	62 447.85 (12 097.4 to 192 502.3) n = 38
Time on ART (year), median (IQR)	7.2 (4.6 to 10.8) n = 123	8.1 (4.75 to 11.15) n = 82	6 (4.35 to 8.95) n = 41
Time from start ART to undetectable viral load (year), median (IQR)	0.3 (0.2 to 0.4) n = 117	0.3 (0.2 to 0.4) n = 78	0.3 (0.1 to 0.7) n = 39
Total HIV‐1 DNA copies/10^6 CD4+ T cells, median (IQR)	651.83 (267.47 to 1322.92) n = 112	772.75 (419.53 to 1387.88) n = 75	511.41 (282.84 to 1482.83) n = 37
Intact proviral HIV‐1 DNA copies/10^6 CD4+ T cells, median (IQR)	21.21 (1.9 to 58.48) n = 87^a^	21.21 (2.07 to 41.56) n = 61	26.01 (0 to 108.68) n = 26
Week 48			
Total HIV‐1 DNA copies/10^6 CD4+ T cells, median (IQR)	439.37 (189.14 to 1185.35) n = 108	503.87 (233.49 to 1246.49) n = 73	324.57 (140.95 to 1123.68) n = 35
Intact proviral HIV‐1 DNA copies/10^6 CD4+ T cells, median (IQR)	12.03 (2.55 to 41.68) n = 87	8.7 (0 to 37.37) n = 61	28.06 (4.75 to 63.45) n = 26
Delta week 48‐baseline			
Total HIV‐1 DNA copies/10^6 CD4+ T cells, median (IQR)	‐122.67 (‐330.98 to 57.03) n = 108	‐131.26 (‐397.41 to ‐93.83) n = 73	‐112.18 (‐219.06 to 42.78) n = 35
Intact proviral HIV‐1 DNA copies/10^6 CD4+ T cells, median (IQR)	‐5.05 (‐28.57 to 14.56) n = 87	‐6.47 (‐30.00 to 15.87) n = 61	‐4.65 (‐18.67 to 14.94) n = 26

^a^Dataset (n = 112) further refined: only participants with quantifiable results at baseline were included.

**Conclusions**: Preliminary investigations of this first head‐to‐head study suggest that HIV‐1 reservoir dynamics are similar between Dovato^®^ and Biktarvy^®^ and that switch towards 3TC/dolutegravir does not increase the total or intact HIV‐1 viral reservoir. Adjusted analyses are on‐going and will be presented.

**References**

1. Cassidy NA, Fish CS, Levy CN, Roychoudhury P, Reeves DB, Hughes SM, et al. HIV reservoir quantification using cross‐subtype multiplex ddPCR. iScience. 2021;25:103615.

2. Jianqing JY, Wu TL, Liszewski MK, Dai J, Swiggard WJ, Baytop C, et al. A more precise HIV integration assay designed to detect small differences finds lower levels of integrated DNA in HAART treated patients. Virology. 2008;379:78‐86.

#### MO43 Prevalence, risk factors and the impact of antiretroviral treatment in SARS‐CoV‐2 infection in people with HIV: a cross‐sectional study

E de Lazzari^1^, J Blanco^1^, N Rico^2^, X Filella^2^, N Egri^3^, R Ruiz^3^, M Marcos^4^, M Mosquera^4^, J Alcami^1^, S Sánchez‐Palomino^1^, C Hurtado^1^, C Rovira^1^, J Ambrosioni^1^, I Chivite^1^, A González‐Cordón^1^, A Inciarte^1^, M Laguno^1^, M Martínez‐Rebollar^1^, L de la Mora^1^, B Torres^1^, E Martínez^1^, J Mallolas^1^, J Miró ^1^

^1^HIV Unit, Infectious Diseases Service, Hospital Clinic ‐ August Pi i Sunyer Biomedical Research Institute (IDIBAPS), University of Barcelona, Barcelona, Spain; ^2^Biochemistry Service, Biological Diagnostic Center, Hospital Clinic ‐ August Pi i Sunyer Biomedical Research Institute (IDIBAPS), University of Barcelona, Barcelona, Spain; ^3^Immunology Service, Biological Diagnostic Center, Hospital Clinic ‐ August Pi i Sunyer Biomedical Research Institute (IDIBAPS), University of Barcelona, Barcelona, Spain; ^4^Microbiology Service, Biological Diagnostic Center, Hospital Clinic ‐ August Pi i Sunyer Biomedical Research Institute (IDIBAPS), University of Barcelona, Barcelona, Spain

**Background**: The risk factors for SARS‐CoV‐2 infection in people living with HIV (PLHIV) are not well known. The protective role of antiretroviral treatment (ART), and in particular of tenofovir disoproxil fumarate (TDF), is controversial, being confirmed by some cohort studies [1] but not others [2]. The objective of this study is to know the prevalence and risk factors of SARS‐CoV‐2 infection and the role of ART in the cohort of 5476 PLHIV at the Hospital Clinic of Barcelona.

**Methods**: Cross‐sectional study of all consecutive PLHIV attending the HIV Unit between November 2020 and May 2021. We determined total antibodies, IgG (Atellica Solution IM analyzer from Siemens Healthiness), IgM and IgA (Luminex) antibodies in plasma against the receptor binding domain (RBD) of the spike glycoprotein of SARS‐CoV‐2. Multivariable Poisson regression with robust standard errors was used to identify predictors of SARS‐CoV‐2 infection (StataCorp, 2021).

**Results**: Of the 5476 patients, 1076 were excluded due to lack of plasma samples (n = 639), previous vaccination (n = 431) or absence of informed consent (n = 6). Four thousand, four hundred patients were included in the study. Overall, median (IQR) age was 48 (39 to 56) years, 84% were male, 68% were men who have sex with men (MSM), 57% were European, 44% had university education, 17% had previous AIDS‐defining diseases, 98% were taking ART, and 92% had an undetectable plasma HIV RNA viral load (<50 copies/mL) with median (IQR) CD4 of 673 (496 to 886.5) and CD8 of 782 (580 to 1068). Sixty‐one percent were on an InSTI‐based ART, 57% on TAF/FTC and 5% on TDF/FTC. Five percent of patients had syphilis during the study period. One thousand, one hundred and eighty had total antibodies against SARS‐COV‐2, but only 780 (18%; 95% CI 17 to 19) had positive IgG (n = 553, 13%), IgA (n = 444, 10%) and/or IgM (n = 483, 11%). Being young and female, MSM, non‐European origin, and infected with syphilis were independently associated with SARS‐COV‐2 infection (Table 1). Neither ART nor the use of tenofovir (TDF or TAF) protected against SARS‐CoV‐2 infection.

**Table:** 

**Abstract MO43 –** Table 1. Independent risk factors of SARS‐CoV2 infection in PLHIV.				
Variable	Variable	Adjusted prevalence rate ratio	(95% confidence interval)	p‐value
Age*Sex	18‐34 Men	1		0.0004
Age*Sex	18‐34 Women	1.719	(1.109; 2.664)	0.0004
Age*Sex	35‐49 Men	0.837	(0.705; 0.993)	0.0004
Age*Sex	35‐49 Women	0.953	(0.669; 1.357)	0.0004
Age*Sex	50‐64 Men	0.651	(0.520; 0.814)	0.0004
Age*Sex	50‐64 Women	0.887	(0.619; 1.270)	0.0004
Age*Sex	≥65 Men	0.648	(0.432; 0.972)	0.0004
Age*Sex	≤65 Women	0.660	(0.299; 1.454)	0.0004
Origin: Continent	Europe	1		<0.0001
Origin: Continent	Americas	1.659	(1.428; 1.928)	<0.0001
Origin: Continent	Others	1.601	(1.129; 2.269)	<0.0001
Origin: Continent	*Missing*	0.943	(0.714; 1.245)	<0.0001
MSM (or Bisexual)	No	1		0.0024
MSM (or Bisexual)	Yes	1.378	(1.120; 1.694)	0.0024
Transexual	No	1		0.6871
Transexual	Yes	0.937	(0.682; 1.287)	0.6871
AIDS‐defining disease	No	1		0.7953
AIDS‐defining disease	Yes	1.028	(0.835; 1.265)	0.7953
Plasma HIV viral load	Detectable	1		0.5664
Plasma HIV viral load	<50 copies/mL	1.078	(0.834; 1.392)	0.5664
CD4 T‐cell count^a^	CD4 T‐cell count1	0.995	(0.984; 1.007)	0.4156
CD8 T‐cell count^a^	CD8 T‐cell count^a^	0.994	(0.986; 1.003)	0.2044
Active syphilis	No	1		0.0001
Active syphilis	Yes	1.507	(1.227; 1.850)	0.0001
on ART	No	1		0.2299
on ART	Yes	0.724	(0.427; 1.227)	0.2299
Type of ART (3rd drug)	InSTI	1		0.2725
Type of ART (3rd drug)	NNRTI	1.160	(0.961; 1.399)	0.2725
Type of ART (3rd drug)	IP	1.085	(0.900; 1.308)	0.2725
NRTI backbone	TAF/FTC	1		0.6372
NRTI backbone	TDF/FTC	1.067	(0.785; 1.448)	0.6372
NRTI backbone	ABC/3TC	1.091	(0.904; 1.317)	0.6372
NRTI backbone	Other	1.169	(0.944; 1.448)	0.6372
NRTI backbone	No NRTI	1.033	(0.783; 1.363)	0.6372

^a^Adjusted prevalence rate ratio per 50 units increase.

**Conclusions**: Nearly a fifth of PLHIV were infected with SARS‐CoV‐2, being infection associated with non‐European young MSM or women, and syphilis. Neither ART nor the use of tenofovir was protective.

**References**

1. Del Amo J, Polo R, Moreno S, Díaz A, Martínez E, Ramón Arribas J, et al. Incidence and severity of COVID‐19 in HIV‐positive persons receiving antiretroviral therapy: a cohort study. Ann Intern Med. 2020;173:536‐41.

2. Nomah DK, Reyes‐Urueña J, Díaz Y, Moreno S, Aceiton J, Bruguera A, et al. Impact of tenofovir on SARS‐CoV‐2 infection and severe outcomes among people living with HIV: a propensity score‐matched study. J Antimicrob Chemother. 2022 Jun 9:dkac177. doi: 10.1093/jac/dkac177.

#### MO44 Prevalence, outcomes, and factors associated with testing for severe acute respiratory syndrome coronavirus 2 (SARS‐CoV‐2) infection among people living with HIV across Europe in the multinational EuroSIDA cohort

O Fursa ^1^, W Bannister^1^, B Neesgaard^1^, D Podlekareva^1^, J Kowalska^2^, T Benfield^3^, J Gerstoft^4^, J Reekie^1^, L Rasmussen^5^, I Aho^6^, G Guaraldi^7^, T Staub^8^, J Miró^9^, J Laporte^10^, D Elbirt^11^, T Trofimova^12^, D Sedlacek^13^, R Matulionyte^14^, C Oprea^15^, V Hadžiosmanović^16^, A Mocroft^1^, L Peters^1^

^1^Rigshospitalet, Centre of Excellence for Health, Immunity and Infections, Copenhagen, Denmark; ^2^Department of Adults' Infectious Diseases, Medical University of Warsaw, Warsaw, Poland; ^3^Department of Infectious Diseases, Copenhagen University Hospital‐Amager and Hvidovre, Hvidovre, Denmark; ^4^Department of Infectious Diseases, Rigshospitalet, Copenhagen, Denmark; ^5^Department of Infectious Diseases, Odense University Hospital, Odense, Denmark; ^6^Division of Infectious Diseases, Helsinki University Hospital, Helsinki, Finland; ^7^Modena HIV Cohort, Università degli Studi di Modena, Modena, Italy; ^8^Service des Maladies Infectieuses, Centre Hospitalier de Luxembourg, Luxembourg, Luxembourg; ^9^Infectious Diseases Service, Hospital Clínic‐IDIBAPS, University of Barcelona, CIBERINFEC, Barcelona, Spain; ^10^Servicio de Hospital a Domicilio, Hospital Universitario de Alava, Vitoria‐Gasteiz, Spain; ^11^Allergy, Immunology and HIV Unit, Kaplan Medical Center, Rehovot, Israel; ^12^HELPER, Novgorod Centre for AIDS Prevention and Control, Veliky Novgorod, Russian Federation; ^13^Department of Infectious Diseases, Charles University Hospital, Plzen, Czech Republic; ^14^Department of Infectious Diseases, Vilnius University, Faculty of Medicine, Vilnius, Lithuania; ^15^HIV Department, Victor Babes Clinical Hospital for Infectious and Tropical Diseases, Bucharest, Romania; ^16^Infectious Diseases Clinic, Clinical Center University of Sarajevo, Sarajevo, Bosnia and Herzegovina

**Background**: With the increasing age and growing burden of comorbidities the population of people living with HIV (PLWHIV) [1,2] might be at higher risk of symptomatic COVID‐19 and worse outcomes [3,4]. We aim to describe SARS‐CoV‐2 testing in a large cohort of PLWHIV and assess factors associated with PCR testing as well as with positive test results.

**Figure:**
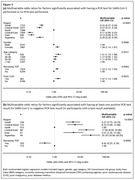
**Abstract MO44 – Figure 1**. Multivariable odds ratios for factors significantly associated with having a PCR test for SARS‐CoV‐2 (a) and having at least one positive PCR test result for SARS‐CoV‐2 (b).

**Table:** 

**Abstract MO44 – Table 1**. Prevalence of SARS‐CoV‐2 PCR tests, results, and hospitalisations in the EuroSIDA regions in 2020.							
SARS‐CoV‐2 PCR testing and results	Total (N = 7228)	Southern Europe (N = 1723)	Central Western Europe (N = 1071)	Northern Europe (N = 1588)	Central Eastern Europe (N = 1584)	Eastern Europe (N = 940)	Argentina (N = 322)
Tested, N	1070	236	122	513	88	67	44
Tested, % (95% CI)	14.8 (14.0 to 15.6)	13.7 (12.1 to 15.4)	11.4 (9.6 to 13.5)	32.3 (30.0 to 34.7)	5.6 (4.5 to 6.8)	7.1 (5.6 to 9.0)	13.7 (10.1 to 17.9)
Positive, N	140	39	23	15	33	12	18
Positive, % (95% CI)	1.9 (1.6 to 2.3)	2.3 (1.6 to 3.1)	2.1 (1.4 to 3.2)	0.9 (0.5 to 1.6)	2.1 (1.4 to 2.9)	1.3 (0.7 to 2.2)	5.6 (3.4 to 8.7)
Negative, N	930	197	99	498	55	55	26
Negative, % (95% CI)	12.9 (12.1 to 13.7)	11.4 (10.0 to 13.0)	9.2 (7.6 to 11.1)	31.4 (29.1 to 33.7)	3.5 (2.6 to 4.5)	5.9 (4.4 to 7.6)	8.1 (5.3 to 11.6)
Hospitalised due to SARS‐CoV‐2, N	28	8	5	4	5	5	1
Hospitalised due to SARS‐CoV‐2, % (95% CI)	0.4 (0.3 to 0.6)	0.5 (0.2 to 0.9)	0.5 (0.2 to 1.1)	0.3 (0.1 to 0.6)	0.3 (0.1 to 0.7)	0.5 (0.2 to 1.2)	0.3 (0.0 to 1.7)

**Materials and methods**: PLWHIV from the EuroSIDA cohort under prospective follow‐up on 1 January 2020 were included from the sites that provided any testing data. Proportions of PCR testing, positive test results, and hospitalisations reported up to 1 January 2021 were compared across five European regions plus Argentina. Multivariable logistic regression was used to determine factors from a pre‐specified set of potential predictors associated (p < 0.05) with being tested for SARS‐CoV‐2 (vs untested) and with at least one positive test result (vs negative).

**Results**: Of 7228 participants, 1070 (14.8%, 95% CI 14.0 to 15.6) had a SARS‐CoV‐2 test reported during 2020. The proportion ranged from 32.3% in Northern Europe to 5.6% in Central‐Eastern and 7.1% in Eastern Europe (Table 1). These differences between regions remained significant after adjustment. Likewise, women, people under 40 years, those with prior CVD, and those receiving TDF‐containing regimen were significantly more likely to have been tested (Figure 1a). Overall, 140 PLWHIV (1.9%, 95% CI 1.6 to 2.3) tested positive, ranging from 0.9% in Northern Europe to 5.6% in Argentina. The adjusted odds of testing positive were the highest in Argentina and Central‐Eastern Europe compared to the North, and lower in PLWHIV receiving TDF (Figure 1b). No other factors reached significance threshold. Twenty‐eight people were hospitalised due to COVID‐19 (0.4% of the study population, 95% CI 0.3 to 0.6), ranging from 0.3% to 0.5% across regions. Of these, five received life support, and six died.

**Conclusions**: We observed large heterogeneity in SARS‐CoV‐2 testing in PLWHIV across EuroSIDA regions, reflecting differences in testing policies and data availability. All regions except North reported a proportion tested below 15% and a high fraction of positive results. TDF was associated both with testing and a negative test result, requiring further investigation. The proportion of hospitalisations was consistent across regions, with a low observed proportion of COVID‐related deaths.

**References**

1. Autenrieth CS, Beck EJ, Stelzle D, Mallouris C, Mahy M, Ghys P. Global and regional trends of people living with HIV aged 50 and over: estimates and projections for 2000–2020. PLoS One. 2018;13:e0207005.

2. Schouten J, Wit FW, Stolte IG, Kootstra NA, van der Valk M, Geerlings SE, et al. Cross‐sectional comparison of the prevalence of age‐associated comorbidities and their risk factors between HIV‐infected and uninfected individuals: the AGEhIV cohort study. Clin Infect Dis. 2014;59:1787‐97.

3. Ssentongo P, Heilbrunn ES, Ssentongo AE, Advani S, Chinchilli VM, Nunez JJ, et al. Epidemiology and outcomes of COVID‐19 in HIV‐infected individuals: a systematic review and meta‐analysis. Sci Rep. 2021;11:6283.

4. Nomah DK, Reyes‐Urueña J, Díaz Y, Moreno S, Aceiton J, Bruguera A, et al. Sociodemographic, clinical, and immunological factors associated with SARS‐CoV‐2 diagnosis and severe COVID‐19 outcomes in people living with HIV. Lancet HIV. 2021;8:e701‐10.

#### MO45 The stability and predictors of change in clinically relevant multimorbidity clusters over time among people with HIV in the Pharmacokinetic and clinical Observations in PeoPle over fifty (POPPY) study

L Sukumaran ^1^, P Mallon^2^, F Post^3^, M Sachikonye^4^, M Boffito^5^, J Meyerowitz^6^, J Vera^7^, I Williams^1^, J Anderson^8^, M Johnson^9^, A Winston^10^, C Sabin^1^

^1^Infection & Population Health, University College London, London, UK; ^2^School Of Medicine, University College Dublin, Dublin, UK; ^3^Inflammation Biology, School of Immunology & Microbial Sciences, Kings College London, London, UK; ^4^HIV i‐Base, UK Community Advisory Board (UKCAB), London, UK; ^5^Faculty of Medicine Centre, Imperial College London, London, UK; ^6^UK Clinical Research Collaboration, Clinical Trials Unit, Imperial College London, London, UK; ^7^Global Health and Infection, Brighton and Sussex Medical School, Brighton, UK; ^8^Centre for the Study of Sexual Health and HIV, Homerton University Hospital NHS Foundation Trust, London, UK; ^9^UCL Division of Medicine, Royal Free Hospital, London, UK; ^10^Department of Infectious Disease, Imperial College London, London, UK

**Background**: The prevalence of multimorbidity is increasing among people with HIV (PWHIV). Although multimorbidity clusters have been defined using cross‐sectional data, their trajectories have not been well studied. We examine the stability of clusters and factors associated with any changes over a 3‐ to 5‐year period in PWHIV participating in the POPPY study.

**Materials and methods**: Common comorbidity patterns in PWHIV were identified using principal component analysis (PCA), based on Somers’ D statistic, at study entry and after 3 to 5 years. Three patterns were extracted based on biological relevance (cardiovascular diseases (CVD), sexually transmitted diseases (STDs) and mental health (MH)) and severity scores for each participant/pattern were determined using PCA coefficients (higher severity scores represent the presence of a greater number of comorbidities). The distribution (median, interquartile range [IQR]) of severity scores were described over time. Predictors (age, gender, ethnicity, sexual orientation, current smoker, body mass index ≥30 kg/m^2^) of changes in the severity scores were assessed using linear regression.

**Results**: The 694 included participants had a median age of 52 [IQR 46 to 59] years, 83.7% were white, 86.6% male, 76.5% MSM, 97.4% on antiretroviral therapy; 90.6% with undetectable HIV viral load. The median [IQR] CVD severity score increased from 0.12 [0.00 to 1.29] at baseline to 0.54 [0.07 to 1.14] at visit 3, with that for MH increasing from 0.29 [0.08 to 1.54] to 0.60 [1.18 to 1.19] over the same period. In contrast, the median STD severity score decreased from 1.35 [0.03 to 2.88] to 0.80 [0.07 to 1.64]. White ethnicity was associated with an increase in severity scores for CVD (0.52 [0.11 to 0.92], p = 0.01) whereas male gender was associated with an increase in MH severity score (0.35 [0.04 to 0.66], p = 0.03). Additionally, MSM was associated with an increase in severity scores for both STDs and MH (1.51 [0.95 to 2.06], p < 0.0001 and 0.52 [0.19 to 0.85], p = 0.002, respectively) (Figure 1).

**Figure:**
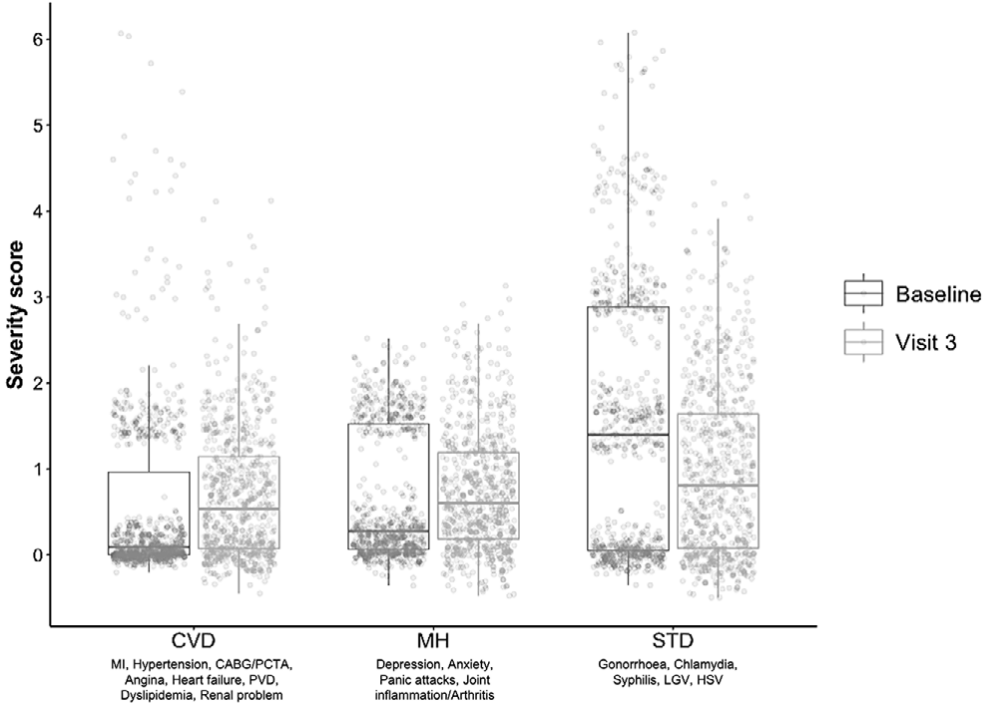
**Abstract MO45 – Figure 1**. Patterns of comorbidities and distribution of their severity scores over a 3‐ to 5‐year period in people with HIV (PWHIV) participating in the POPPY study (n = 694). * Severity scores were determined using participants principal component analysis (PCA) coefficients for each cluster. A higher severity score denotes a participant having a greater number of comorbidities within that cluster.

**Conclusion**: The burden of patterns of comorbidities, especially CVD and MH, have increased over time. Changes in severity scores across patterns appear to be attributable to different demographic/lifestyle factors, suggesting that development of targeted interventions are crucial for PWHIV exhibiting distinct patterns of multimorbidity.

#### MO46 Trends in maternal characteristics and pregnancy outcomes among women living with HIV in the UK: 2014 to 2019

H Peters, L Bukasa, K Francis, R Sconza, C Thorne

The Integrated Screening Outcomes Surveillance Service, part of the NHS Infectious Diseases in Pregnancy Screening Programme, UCL Great Ormond Street Institute of Child Health, London, UK

**Background**: The vertical transmission rate in the UK has remained <0.3% since 2012. We describe recent trends in characteristics and outcomes of pregnancies in women living with HIV (WLWH) in the UK in 2014 to 2019.

**Materials and methods**: The Integrated Screening Outcomes Surveillance Service (ISOSS), part of the NHS Infectious Diseases in Pregnancy Screening Programme, commissioned by NHSE, conducts population‐based surveillance of pregnancies in WLWH. Analyses covered pregnancies in WLWH diagnosed before delivery with estimated date of delivery (EDD) 2014 to 2019, reported by 31/12/2021.

**Results**: There were 5858 pregnancies among 3353 women, with annual numbers decreasing from ∼1100 in 2014 to 2015 to 800 to 900 in 2018 to 2019. The median age at EDD was 34 years (IQR 30 to 38) with the proportion of pregnancies in women aged ≥40 years increasing from 12.5% (278/2224) in 2014 to 2015 to 19.1% (316/1655) in 2018 to 2019, p < 0.001. Pregnancies in women born in sub‐Saharan Africa declined, from 72.0% (1575/2187) in 2014 to 2015 to 64.1% (1052/1642) in 2018 to 2019, while those among women born in Eastern Europe increased from 4.3% (95/2187) to 6.9% (114/1642), p < 0.001. Proportion of pregnancies in women with vertically‐acquired HIV increased from 1.7% (35/2055) in 2014 to 2015 to 3.7% (55/1500) in 2018 to 2019, p < 0.01. By 2018 to 2019, 90.6% (1500/1655) of pregnancies were in women diagnosed before pregnancy, an increase from 86.8% (1925/2219) in 2014 to 2015 (p < 0.001). The proportion of women on ART at conception increased from 67.2% (1453/2162) to 81.0% (1321/1630) (p < 0.001). Among women with antenatal diagnosis, there was earlier median start of ART (19 weeks [IQR 16 to 23] in 2014 to 2015, 16 weeks [14 to 20] in 2018 to 2019). Proportion of women with first antenatal CD4 count >500 increased from 51.2% in 2014 to 2015 to 58.5% in 2018 to 2019 (p = 0.001). Over the period, >90% of delivery viral loads were undetectable (<50 copies/mL) (91.3% in 2014 to 2015, 93.1% in 2018 to 2019), p = 0.278. Vaginal deliveries increased from 44.3% to 47.4% in 2014 to 2019 (p = 0.018); the preterm delivery rate remained ∼12%. Supported breastfeeding cases increased from 1.5% (24/1595) in 2014 to 2015 to 5.8% (72/1240) in 2018 to 2019, p < 0.001.

**Conclusion**: Changes in the population of WLWH accessing antenatal care in the UK have implications for service provision and require monitoring. Clinical outcomes are reassuring and ISOSS will continue to monitor emerging areas of interest including infant feeding and health inequalities.

### Monkeypox: Where Are We Now and What Have We Learned?

#### O41A Perceptions and understandings of media and public health messaging about the monkeypox outbreak in the UK: findings from a rapid response, co‐produced survey

S Paparini ^1^, J Thornhill^1^, C Mwendera^1^, S Strachan^2^, R Whitacre^3^, W Nutland^4^, C Orkin^1^

^1^SHARE Collaborative, Queen Mary University of London, London, UK; ^2^Sophia Forum, London, UK; ^3^Global Health Centre, Graduate Institute of International and Development Studies, Geneva, Switzerland; ^4^PrEPster / The Love Tank CIC, London, UK

**Background**: The current global human monkeypox (MPXV) emergency almost exclusively affects men who have sex with men (MSM). In an era of misinformation, clear scientific communication is vital to pandemic control. Non‐stigmatising, community co‐created public health interventions are essential to engage at‐risk populations. We report the first community survey during this pandemic.

**Methods**: A UK‐based, anonymous, online community survey, co‐produced by academics at Queen Mary University of London, Sophia Forum and The Love Tank CIC, was administered between 15 June and 24 July 2022. Aim was to explore: understanding of risk; trusted sources of information; views on public health and media messaging; understanding and acceptance of public health messages (engagement with care, isolation rules and vaccine acceptability). Respondents were invited via mailing lists, social media and dating app broadcasts. Categorical data were analysed using SPSS, group comparisons were made using Chi square tests.

**Results**: One thousand, nine hundred and thirty‐two respondents who completed the survey, 1751 (91%) identified as men, 88 (5%) as women, 64 (3%) as gender non‐conforming, 1537 (80%) as gay/lesbian/queer, 223 (12%) bisexual, 81 (5%) heterosexual. One thousand, three hundred and sixty‐six (71%) identified as White, Black 67 (3%), Asian 158 (8%); 742 (38%) were <40 years, 76% were employed, 72% had completed higher education. The most trusted sources of information were 'healthcare professionals' (37%) 'official health agencies' (29%), 'mainstream media' (12%). Sixty‐seven percent considered media representation of MPXV discriminatory against gay men, 30% against Black people. In terms of public health messages: 66% reported that they understood MPXV public health messaging and 61% agreed with isolating for MPXV; 49% would first attend a sexual health clinic for MPXV infection; 41% did not know or believe that MPXV originates from animals. Overall, 52% felt they were at risk of MPXV, while 84% would answer yes to a vaccine if exposed. Significant differences were observed between respondent groups: MSM/non‐MSM, male/female, those <40/>40 years, gender non‐conforming individuals/others and those with/without university education with respect to risk perception, trusted information, and attitudes to vaccination.

**Conclusions**: In this group of largely White, university‐educated and employed MSM, MPXV vaccine acceptability was very high. However other key public health messages such as where to attend for care, origins of the virus and isolation rules were not as evenly accepted or correctly understood.

#### O41B Including women in the public health response to the monkeypox (MPXV) outbreak in the UK: findings from a rapid response, co‐produced survey

S Paparini ^1^, S Strachan^2^, J Thornhill^3^, W Nutland^4^, C Mwendera^3^, C Orkin^3^

^1^Wolfson Institute of Population Health and SHARE Collaborative, Queen Mary University of London, London, UK; ^2^Sophia Forum, London, UK; ^3^Blizard Institute and SHARE Collaborative, Queen Mary University of London, London, UK; ^4^PrEPster / The Love Tank CIC, London, UK

**Background**: New global outbreaks of human monkeypox (MPXV) in non‐endemic regions affect men who have sex with men (MSM) almost exclusively. Successful public health promotion must alert those most at risk. Balanced and comprehensive information for the broader public is however essential to reduce stigmatisation of specific communities and to ensure care engagement where appropriate. A diversity of views is needed in the MPXV public health response.

**Methods**: A UK‐based, anonymous, online community survey, co‐produced by researchers at Queen Mary University of London, Sophia Forum and The Love Tank CIC, was administered between 15 June and 24 July 2022. Aim was to explore: understanding of risk; trusted sources of information; views on public health and media messaging; understanding and acceptance of public health messaging. Respondents were invited via mailing lists, social media and dating app broadcasts. Categorical data were analysed using SPSS. Content and thematic analyses were used for free text responses. A focussed sub‐analysis, presented here, was done for respondents identifying as women.

**Results**: Of the 1932 survey respondents, 103 identified as cis (88) or trans (15) women, and mostly as heterosexual (59%). Over 50% were between the age of 30 and 59. Self‐identified ethnicities were Asian (six), Black (five), LatinX (one), White (78), Other or Mixed (13). Twenty‐three percent of women reported they had a disability and 11 were living with HIV. Women were largely employed (72%) and highly educated (80%). Most (73%) received their information about MPXV via social media platforms yet trusted healthcare providers (39%) and government websites (15%) the most. Seventy‐four percent were in favour of receiving a vaccine. Women agreed media representations about MPXV had been racist (50%) and homophobic (70%). Respondents commented in the free text that public health messaging needs to emphasise risk for MSM and must be clear and factual to avoid the general public ignoring risk and symptoms, and further stigmatising MSM. Some women criticised media representations of MPXV as an 'African disease'.

**Conclusions**: It is unusual to succeed in including a substantial amount of cis and trans women in surveys predominantly recruiting MSM. Women in the survey showed high levels of awareness and understanding about the MPXV outbreak, and high vaccine acceptance.

### Late Breakers/Hot Topics

#### O42 Virological failure and HIV RNA re‐suppression rates in four randomised trials of dolutegravir, efavirenz or protease inhibitor‐based treatment in 3116 participants

S Sokhela^1^, F Venter^1^, B Bosch^1^, G Akpomiemie^1^, A Tembo^1^, T Pepperrell^2^, B Simmons^3^, L Mulenga^4^, A Calmy^5^, T Sanchez^6^, E Delaporte^6^, C Casas^7^, S Khoo^8^, H Reynolds^8^, A Hill ^9^

^1^Faculty of Health Sciences, Ezintsha, University of the Witwatersrand, Johannesburg, South Africa; ^2^School of Medicine and Veterinary Medicine, University of Edinburgh, Edinburgh, UK; ^3^LSE Health, London School of Economics and Political Science, London, UK; ^4^Department of Medicine, University Teaching Hospital, Lusaka, Zambia; ^5^Division of Infectious Diseases, HIV‐AIDS Unit, Geneva University Hospitals, Geneva, Switzerland; ^6^TransVIHMI, University of Montpellier, Montpellier, France; ^7^Global Health Campus, Unitaid, Le Grand‐Saconnex, Switzerland; ^8^Department of Molecular and Clinical Pharmacology, University of Liverpool, Liverpool, UK; ^9^Department of Pharmacology and Therapeutics, University of Liverpool, Liverpool, UK

**Introduction**: WHO guidelines currently recommend switches in treatment for patients with HIV RNA persistently above 1000 copies/mL despite adherence counselling. Ongoing viraemia could increase the chance of drug resistance. However, patients can show re‐suppression of HIV RNA after adherence counselling, with no change in treatment. We compared rates of virological failure (VF) and re‐suppression in four randomised trials of dolutegravir (DTG), efavirenz (EFV) and protease inhibitors (PI/r).

**Methods**: Data were analysed from four randomised trials: DolPHIN‐2, ADVANCE, NAMSAL and VISEND. VF was defined as HIV RNA >1000 copies/mL after week 24. HIV RNA re‐suppression was defined as HIV RNA <50 copies/mL at the next visit in DolPHIN‐2, ADVANCE and VISEND, or <200 in NAMSAL. ‘Sustained viraemia’ was then subdivided into HIV RNA 50 to 99 or >1000 copies/mL at next visit. The percentage of participants with VF and HIV RNA re‐suppression was then compared between treatment classes in a meta‐analysis.

**Results**: DolPHIN‐2 was conducted in South Africa and Uganda, ADVANCE in South Africa, NAMSAL in Cameroon and VISEND in Zambia. Rates of VF were not significantly different between DTG and EFV in ADVANCE (DTG 12%, EFV 9%), DOLPHIN‐2 (DTG 33%, EFV 30%), and NAMSAL trials (DTG 16%, EFV 15%) (Table 1). In VISEND, VF was significantly lower for DTG versus PI/r (DTG 16%, PI/r 24%, p = 0.0048). Following VF, HIV RNA re‐suppression rates were significantly higher for DTG in ADVANCE (DTG 57%, EFV 23%) and NAMSAL (DTG 60%, EFV 29%), but not in DOLPHIN‐2 (DTG 34%, EFV 34%). In the meta‐analysis, overall re‐suppression rates were significantly higher for DTG versus EFV (p = 0.04). In VISEND, HIV RNA re‐suppression was significantly more common for DTG versus PI/r (DTG 38%, PI/r 19%, p = 0.0094).

**Discussion**: In this analysis of 3116 patients in four randomised trials, episodes of viraemia >1000 copies/mL were seen for a range of treatments. However, HIV RNA re‐suppression after initial viraemia was significantly more likely for participants taking DTG‐based treatment, compared with either EFV‐ or PI‐based treatment.  For patients with VF on DTG, the benefits of switching to new drug classes are unclear, versus remaining long term on DTG with adherence counselling.

**Table:** 

**Abstract O42 – Table 1**. Analysis results. Note: VISEND: Arm A, HIV RNA <1000 at screening; Arm B, HIV RNA >1000 at screening.						
Trial	Arm	Viral failure	Re‐suppression (<50/<200)	Sustained viraemia (>1000)	Sustained viraemia (50 to 999)	Lost to follow‐up
ADVANCE	TAF/FTC/DTG	40/351 (11%)	22/40 (55%)	11/40 (27%)	1/40 (2%)	6/40 (15%)
ADVANCE	TDF/FTC/DTG	43/351 (12%)	25/43 (58%)	6/43 (14%)	5/43 (12%)	7/43 (16%)
ADVANCE	TDF/FTC/EFV	31/351 (9%)	7/31 (23%)	16/31 (52%)	4/31 (13%)	4/31 (13%)
DOLPHIN‐2	TDF/3TC/DTG	41/124 (33%)	14/41 (34%)	10/41 (24%)	8/41 (19%)	9/41 (22%)
DOLPHIN‐2	TDF/3TC/EFV	38/125 (30%)	13/38 (34%)	4/38 (10%)	8/38 (21%)	13/38 (34%)
NAMSAL	TDF/3TC/DTG	48/307 (16%)	29/48 (60%)	10/48 (21%)	3/48 (6%)	6/48 (12%)
NAMSAL	TDF/3TC/EFV	45/306 (15%)	13/45 (29%)	21/45 (47%)	5/45 (11%)	6/45 (13%)
VISEND	TDF/FTC/DTG (A)	10/209 (5%)	5/10 (50%)	2/10 (20%)	1/10 (10%)	2/10 (20%)
VISEND	TAF/FTC/DTG (A)	13/209 (6%)	4/13 (31%)	3/13 (23%)	5/13 (38%)	1/13 (8%)
VISEND	TDF/FTC/DTG (B)	39/208 (19%)	16/39 (41%)	11/39 (28%)	7/39 (18%)	5/39 (13%)
VISEND	TAF/FTC/DTG (B)	26/211 (12%)	9/26 (35%)	8/26 (31%)	3/26 (11%)	6/26 (23%)
VISEND	ZDV/3TC/LPV/r (B)	46/167 (27%)	9/46 (20%)	18/46 (39%)	7/46 (15%)	12/46 (26%)
VISEND	ZDV/3TC/ATV/r (B)	40/197 (20%)	7/40 (17%)	19/40 (47%)	8/40 (20%)	6/40 (15%)

#### O43 Six‐month outcomes of every 2‐months long‐acting cabotegravir and rilpivirine in a real‐world setting: effectiveness, adherence to injections and patient‐reported outcomes from PLWHIV in the German CARLOS cohort

J Borch^1^, J Scherzer^2^, C Jonsson‐Oldenbuettel ^3^, G Weinberg^4^, C Wyen^5^, E Rodriguez^6^, S Scholten^7^, S Dakhia^8^, K Dymek^2^, B Westermayer^9^, K Bernhardt^2^

^1^Clinical Care, Praxis Goldstein, Berlin, Germany; ^2^Medical Affairs, ViiV Healthcare, Munich, Germany; ^3^Clinical Care, Medizinisches Versorgungszentrum München am Goetheplatz, Munich, Germany; ^4^Clinical Care, Infektiologisches Zentrum Steglitz, Berlin, Germany; ^5^Clinical Care, Praxis Ebertplatz, Cologne, Germany; ^6^Clinical Care, Praxisgemeinschaft ViRo Schillerkiez, Berlin, Germany; ^7^Clinical Care, Praxis Hohenstaufenring, Cologne, Germany; ^8^Medical Affairs, ViiV Healthcare, Brentford, UK; ^9^Medical Affairs, GlaxoSmithKline GmbH, Munich, Germany

**Background**: The long‐acting (LA) regimen of cabotegravir (CAB) and rilpivirine (RPV) offers an alternative mode of drug administration with less frequent dosing than daily oral antiretroviral therapy (ART). The prospective CARLOS cohort has been initiated to generate the first real‐world evidence on effectiveness, adherence and patient experience of individuals choosing CAB+RPV LA in routine clinical care in Germany. Here we describe interim outcomes at 6 months.

**Materials and methods**: CARLOS is a non‐interventional, 3‐year multi‐centre cohort study including people living with HIV (PLWHIV) on suppressive daily oral ART switched to every 2‐months CAB+RPV LA in accordance with the label. Interim outcomes at time of injection 4/month 6 (M6) include effectiveness, adherence to injection window and patient‐reported outcomes including change in treatment satisfaction using the HIV Treatment Satisfaction Questionnaire [status version; HIV‐TSQs].

**Results**: Two hundred and thirty‐six PLWHIV reached the target window for 4th injection and were included in the analysis population at M6. Baseline characteristics and reasons for switch to CAB+RPV LA are shown in Table 1. The majority (84.7%; n = 200/236) started with oral lead‐in (OLI). 90.7% (n = 574/633) of CAB+RPV LA injections were administered within the +/‐7 day injection window, 6.5% occurred early and 2.8% occurred late. Oral bridging was documented for six individuals. At M6 86.5% (n = 179/207) maintained virological suppression and 2.9% (6/207) discontinued due to injection site reactions (ISRs). There were two virological failures (2/207; 1.0%). Full virological outcomes are shown in Figure 1. For PLWHIV completing the HIV‐TSQs at baseline (mean score = 55.3) and at M6 (mean score = 60.6), a statistically significant treatment satisfaction score increase was observed (mean change = +5.4; p < 0.001; n = 157).

**Conclusion**: In this real‐world cohort, switch to CAB+RPV LA is primarily patient driven, with low rates of PLWHIV discontinuing due to ISRs and the vast majority of injections being administered within the window or early. CAB+RPV LA shows high rates of maintenance of viral suppression, with low rates of treatment failure in the first 6 months following switch. Despite already high scores on oral ART, the treatment satisfaction score increased statistically significantly on long‐acting ART in patients mostly self‐selecting CAB+RPV LA.

**Table:** 

**Abstract O43 – Table 1**. Baseline characteristics.		
Baseline characteristics	Total	Observed data
Sex, male, % (n)	95.3% (225)	236
Age, years, median (IQR)	43 (36 to 50)	236
Age categories <50; 50 to 65; >65, % (n)	74.6% (176); 25.0% (59); 0.4% (1)	236
BMI ≥30 kg/m^2^, % (n)	12.2% (23)	189
CD4 T‐cell count, cells/μL, median (IQR)	721 (542 to 991)	232
History of AIDS (CDC C), % (n)	8.5% (20)	236
Time on ART, years (median, IQR)	8.0 (4.9 to 11.6)	210
≥3 previous ART regimens, % (n)	53.8% (106)	197
Most common regimens (>10%) prior to switch to CAB+RPV LA	B/F/TAF: 24.0% (n = 55); DTG/3TC: 18.8% (n = 43)	229
Reason for switch to CAB+RPV LA (HCP perspective)		236
Patient wish	91.9% (n = 217)	
Adherence concerns under oral ART	5.5% (n = 13)	
Medical need for parent	1.7% (n = 4)	
Other	0.8% (n = 2)	
Resistance test available at/before switch to CAB+RPV LA	60.6% (n = 143)	236
HIV‐1 subtype		236
A	0.8% (n = 2)	
A1	1.3% (n = 3)	
A2	2.1% (n = 5)	
B	50.4% (n = 119)	
Other	5.1% (n = 12)	
Unknown subtype	40.3% (n = 95)	

B/F/TAF, bictegravir/emtricitabine/tenofovir alafenamide; DTG/3TC, dolutegravir/lamivudine; IQR, interquartile range.

**Figure:**
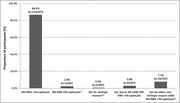
**Abstract O43 – Figure 1**. Virological outcomes at injection 4/month 6 (missing=excluded: no viral data in window (n = 25), loss to follow‐up (n = 4)). * Incl. n = 1 virological failure (confirmed HIV‐RNA ≥200 copies/mL or single HIV‐RNA ≥200 copies/mL followed by treatment d/c): HIV‐1 subtype B, BMI 23 kg/m^2^, no history of NNRTI resistance‐associated mutations (RAMs) (INSTI resistance test not performed), injections in window, emergent NNRTI RAM (Y181C) and INSTI RAMs (L74I, T97A, E138K, Q148R, N155H) detected [BW1]); n = 2 with single HIV‐RNA >200 copies/mL and n = 2 with 50 copies/mL; ** virologic failure; HIV‐1 subtype C, BMI 20 kg/m^2^, no history of NNRTI or INSTI RAMs, injections in window, emergent NNRTI RAMs (K101E, Y181C, G190A) detected; ^ incl. n = 7 participants preferring oral ART. D/c, discontinuation.

#### O44 Expanded multivariable models to assist patient selection for long‐acting cabotegravir+rilpivirine treatment: clinical utility of a combination of patient, drug concentration, and viral factors associated with virological failure over 152 weeks

C Orkin ^1^, J Schapiro^2^, C Perno^3^, D Kuritzkes^4^, P Patel^5^, R DeMoor^6^, D Dorey^7^, Y Wang^8^, K Han^9^, V Van Eygen^10^, H Crauwels^11^, S Ford^12^, C Latham^13^, M St. Clair^13^, J Polli^14^, S Vanveggel^15^, K Vandermuelen^16^, R D'Amico^17^, H Garges^18^, A Zolopa^19^, W Spreen^17^, J van Wyk^20^, A Cutrell^21^

^1^Department of Immunobiology, Queen Mary University, London, UK; ^2^National Hemophilia Center, Sheba Medical Center, Ramat Gan, Israel; ^3^Microbiology and Diagnostic Immunology, Bambino Gesu' Pediatric Hospital, Rome, Italy; ^4^Division of Infectious Diseases, Brigham and Women's Hospital, Harvard Medical School, Boston, MA, USA; ^5^Global Medical Affairs, ViiV Healthcare, Durham, NC, USA; ^6^R&D Biostatistics, GlaxoSmithKline, Collegeville, PA, USA; ^7^R&D Biostatistics, GlaxoSmithKline, Mississauga, Canada; ^8^Biostatistics and Data Management, ViiV Healthcare Ltd, Durham, NC, USA; ^9^Clinical Pharmacology Modeling & Simulation, GlaxoSmithKline, Collegeville, PA, USA; ^10^Clinical Microbiology and Immunology, Janssen Research & Development, Beerse, Belgium; ^11^Clinical Pharmacology, Janssen Research & Development, Beerse, Belgium; ^12^Clinical Pharmacology, GlaxoSmithKline, Durham, NC, USA; ^13^Translational Medicine Research, ViiV Healthcare Ltd, Durham, NC, USA; ^14^Scientific Communications and Medical Education, ViiV Healthcare Ltd, Durham, NC, USA; ^15^Statistics and Decision Sciences, Janssen Research & Development, Beerse, Belgium; ^16^Infectious Diseases, Janssen Research and Development, Beerse, Belgium; ^17^Research & Development, ViiV Healthcare Ltd, Durham, NC, USA; ^18^Global Medical, ViiV Healthcare Ltd, Durham, NC, USA; ^19^North America Medical Affairs, ViiV Healthcare Ltd, Durham, NC, USA; ^20^Global Medical, ViiV Healthcare Ltd, Brentford, UK; ^21^Clinical Statistics, ViiV Healthcare Ltd, Durham, NC, USA

**Background**: Confirmed virological failure (CVF) on long‐acting cabotegravir (CAB) and rilpivirine (RPV) therapy occurred in 1% of participants in clinical trials through 48 weeks with few cases thereafter. Post hoc multivariable analyses exploring predictors of CVF were expanded to include data beyond week 48, and to incorporate additional factors/participants.

**Materials and methods**: Pooled data from 1651 participants (n = 1431 complete records) examined the influence of baseline viral, participant, and dosing regimen factors on CVF. These factors were evaluated in additional models that included predicted CAB/RPV trough concentrations (after 4 and 44 weeks of injections) in participants with no prior CAB+RPV exposure (n = 1292). Using Poisson regression modelling with variable selection procedures, retained factors were evaluated to understand their contribution to CVF (when present alone or in combination).

**Results**: After 4291 person‐years (pys), the unadjusted CVF incidence rate was 0.54 per 100 pys (Q4W, 0.42; Q8W, 0.85; Q4W switch to Q8W, 0.54). 1.6% (n = 23/1431) of participants had CVF (n = 4/23 occurred after week 48) and 86% (n = 1231/1431) maintained virological suppression. Baseline RPV resistance‐associated mutations (RAMs), HIV‐1 subtype A6/A1, and BMI ≥30 kg/m^2^ were predictive of CVF (p < 0.05 adjusted incidence rate ratio), with ≥2 baseline factors (BLFs) conferring higher risk (19.3%; NPV 99.1%) versus no BLFs (0.4%; NPV 95.9%) (Table 1). Virological suppression rates were 87%, 85%, and 77% in participants with 0, 1, or ≥2 key BLFs, respectively. Low predicted CAB/RPV troughs (≤1st quartile), but not BMI, were additional predictive factors in models including post‐baseline pharmacokinetic variables. Regimen (Q8W/Q4W), gender, and CAB/other INSTI RAMs had no significant association with CVF.

**Table:** 

**Abstract O44 – Table 1**. Virological outcomes by the presence of key baseline and post‐baseline factors.					
Three baseline factors: RPV RAMs,^b^ subtype A6/A1,^c^ and BMI ≥30 kg/m^2^			Two baseline factors + CAB and RPV PK^a^: RPV RAMs,^b^ subtype A6/A1,^c^ low initial CAB trough,^a^ and low initial RPV trough^a^		
Baseline factors (number)	Virological suppression, n (%)^d^	CVF, n (%)^e^	Factors (number)	Virological suppression, n (%)^d^	CVF, n (%)^e^
0	844/970 (87.0)	4/970 (0.4)^f^	0	584/664 (88.0)	0/664 (0)^i^
1	343/404 (84.9)	8/404 (2.0)^g^	1	339/396 (85.6)	5/396 (1.3)^j^
≥2	44/57 (77.2)	11/57 (19.3)^h^	≥2	190/232 (81.9)	17/232 (7.3)
Total (95% CI)	1231/1431 (86.0) (84.1% to 87.8%)	23/1431 (1.6) (1.0% to 2.4%)	≥3	28/39 (71.8)	8/39 (20.5)^k^
		18/1224 (1.47)^l^	Total (95% CI)	1113/1292 (86.1) (84.1% to 88.0%)	22/1292 (1.7) (1.1% to 2.6%)

BMI, body mass index; CAB, cabotegravir; CVF, confirmed virological failure; FDA, Food and Drug Administration; NPV, negative predictive value; PK, pharmacokinetic; PPV, positive predictive value; RAM, resistance‐associated mutation; RPV, rilpivirine.

^a^Below first quartile; ^b^screening plasma samples were used for reverse transcriptase genotype analysis for participants in the FLAIR study. Baseline genotyping of participant samples from the ATLAS and ATLAS‐2M studies was carried out retrospectively in peripheral blood mononuclear cell (PBMC) samples (pro‐viral DNA); ^c^HIV‐1 subtype A1 or A6 classification based on Los Alamos National Library panel from HIV Sequence database (June 2020); ^d^based on the FDA Snapshot algorithm of HIV‐1 RNA <50 copies/mL at week 48 for ATLAS, week 124 for FLAIR, and week 152 for ATLAS‐2M; ^e^defined as two consecutive measurements of HIV‐1 RNA ≥200 copies/mL; ^f^PPV 0.4%; NPV 95.9%; sensitivity 17.4%; specificity 31.4%; ^g^PPV 2.0%; NPV 98.5%; sensitivity 34.8%; specificity 71.9%; ^h^PPV 19.3%; NPV 99.1%; sensitivity 47.8%; specificity 96.7%; ^i^PPV 0%; NPV 96.5%; sensitivity 0%; specificity 47.7%; ^j^PPV 1.3%; NPV 98.1%; sensitivity 22.7%; specificity 69.2%; ^k^PPV 20.5%; NPV 98.9%; sensitivity 36.4%; specificity 97.6%; ^l^analysis dataset for the multivariable modelling.

**Conclusions**: The presence of ≥2 BLFs (RPV RAMs, A6/A1 subtype, and/or BMI ≥30 kg/m^2^; 4% of population analysed), or ≥3 factors (when predicted trough pharmacokinetic data are included; 3% of population analysed), was associated with a higher CVF risk after up to 3 years of long‐acting therapy. In absence of baseline and/or pharmacokinetic factor combinations (e.g. <2 or 3), CVF rates were low (NPV 99%), adding to the understanding of appropriate use of this long‐acting treatment option.

#### O45 Modeling and simulation to optimize islatravir once daily (QD) doses in HIV treatment naïve and virologically suppressed populations

R Vargo ^1^, S Robey^1^, X Zang^1^, L Du^1^, J Roberts^2^, S Klopfer^1^, T Correll^1^, K Squires^1^

^1^Merck & Co., Inc., Rahway, NJ, USA; ^2^Simulation Plus, Cognigen Division, Buffalo, NY, USA

**Background**: Islatravir (ISL) is a nucleoside reverse transcriptase translocation inhibitor (NRTTI) being studied for HIV‐1 treatment and prevention. Exposure‐related decreases in total lymphocytes and CD4+ T‐cell counts were observed across ISL clinical trials, with higher frequencies and magnitude of changes observed in ISL higher‐dose regimens (20 mg once weekly; 60 and 120 mg once monthly). Data from the long term ISL treatment and PrEP trials were used to develop models that describe the changes in lymphocytes and CD4+ T‐cells over time in relationship to intracellular ISL‐TP concentrations. Optimized doses were identified to achieve efficacy thresholds and similar CD4+ T cell and lymphocyte dynamics compared to standard ART.

**Methods**: A two‐step model building approach was taken. First, the ISL population pharmacokinetic (POPPK) model was used to generate individual posthoc estimates for the PK model parameters and were included in the lymphocyte and CD4+ cell dataset. Then, the CD4+ T cell and lymphocyte models were developed using the individual PK parameters and observed cell changes over time from long term ISL studies. CD4+ T cell changes were summarized across approved ART regimens for the virologically suppressed population to compare to PK/PD model predictions. The CD4+ T cell and lymphocyte models were then used to predict changes at different ISL dose levels. To predict the efficacy of a regimen, the POPPK model was simulated to assess achievement of PK thresholds. The efficacy and CD4+ T cell and lymphocyte predictions were compared to inform on a new recommended QD dose regimen.

**Results**: 0.25 mg QD is predicted to achieve efficacy thresholds for all individuals for wild type virus and M184I/V variant. The CD4+ T cell and lymphocyte models adequately capture the dynamics for standard of care arms and ISL treatment arms for the Phase 2 and 3 treatment and Phase 2 PrEP trials. ISL 0.25 mg QD is predicted to result in similar CD4+ T cells and lymphocyte changes as standard antiretroviral therapy. Additionally, the predicted results fall within the range of CD4+ T cell changes observed in other virologically suppressed trials for approved ART regimens.

**Conclusions**: ISL 0.25 mg QD in combination with DOR 100 mg is predicted to achieve efficacious exposures for wild‐type and M184I/V HIV‐1 variants and have similar CD4+ T cell and lymphocyte changes as standard ART for treatment naïve and virologically suppressed populations.

#### O46 Total lymphocyte and CD4+ T‐cell count changes in participants receiving islatravir (0.25, 0.75 and 2.25 mg QD) and doravirine +/‐ lamivudine: post‐hoc analysis from a phase 2b dose‐ ranging study (P011)

T Correll ^1^, J Molina^2^, S Klopfer^1^, A Grandhi^1^, R Lahoulou^3^, Y‐P Zhou^1^, K Eves^1^, K Squires^1^

^1^Merck & Co., Inc., Rahway, NJ, USA; ^2^University of Paris, St‐Louis and Lariboisière Hospitals, Paris, France; ^3^MSD France, Puteaux, France

**Background**: Islatravir (ISL) is a nucleoside reverse transcriptase translocation inhibitor (NRTTI) being studied for HIV‐1 treatment and prevention. Exposure‐related decreases in total lymphocyte and/or CD4+ T‐cell counts were observed across ISL clinical trials, with higher frequencies and magnitude of changes observed in ISL higher‐dose programs (20 mg weekly; 60 and 120 mg monthly). We conducted post‐hoc analyses of changes in total lymphocyte and lymphocyte subset counts in the phase 2b dose‐ranging study (P011) of ISL with doravirine (DOR) +/‐ lamivudine (3TC).

**Methods**: P011 (NCT03272347) was a 4‐part, randomized, dose‐ranging study; participants initially received ISL (0.25, 0.75, or 2.25 mg) with DOR (100 mg) and 3TC (300 mg) or the fixed‐dose combination DOR/3TC/tenofovir disoproxil fumarate (TDF) once daily (Part 1). Participants receiving ISL+DOR+3TC and achieving HIV‐1 RNA <50 copies/mL at week 20 or later stopped 3TC and continued their assigned initial ISL dose (blinded) with DOR (Part 2). Participants randomized to ISL switched to the 0.75 mg dose between weeks 60 and 84 and continued through week 144 (Part 3). Participants in the comparator arm continued DOR/3TC/TDF through week 144. Post‐hoc analyses were conducted evaluating ISL effects on total lymphocyte and lymphocyte subset counts in Parts 1 and 2 through week 72 (prior to dose conversion). Participants who switched to ISL 0.75 mg before week 72 were censored from the week 72 analysis but included in all time points prior to switch. Incidence of infections and assessment of hematology parameters were also examined.

**Results**: Changes from baseline in total lymphocyte counts were comparable for participants in the ISL 0.25 mg and DOR/3TC/TDF groups and were more favorable than changes in the ISL 0.75 mg and 2.25 mg groups (Table 1). Increases from baseline in CD4+ T‐cell counts were similar for the ISL 0.25 mg and DOR/3TC/TDF groups (Table 1). The incidence of infections was comparable across all treatment groups. No effects on other hematology parameters were observed.

**Conclusions**: Participants receiving ISL 0.25 mg + DOR (+/‐ 3TC) and those receiving DOR/3TC/TDF had comparable changes in total lymphocyte counts, with robust increases in CD4+ T‐cell counts. These results support further evaluation of ISL 0.25 mg with DOR in treatment‐naïve and virologically suppressed people living with HIV‐1.

**Table:** 

**Abstract O46 – Table 1**. Change in total lymphocyte counts and CD4+ T‐cell counts in treatment‐naïve participants with HIV‐1, MK‐8591‐011 parts 1 and 2 (through week 72).		
**Total lymphocyte counts (10^9^ cells/L)**		
Treatment group	N	Mean % change from baseline (95% CI)^a^
DOR/3TC/TDF	22	15.9 (2.0, 29.9)
ISL 0.25 mg + DOR 100 mg (+/‐ 3TC)	19	20.5 (4.3, 36.6)
ISL 0.75 mg + DOR 100 mg (+/‐ 3TC)	19	‐0.4 (‐14.9, 14.1)
ISL 2.25 mg + DOR 100 mg (+/‐ 3TC)	16	‐15.9 (‐31.9, 0.1)
**CD4+ T‐cell counts (cells/mm^3^)**		
Treatment group	N	Mean % change from baseline (95% CI)^a^
DOR/3TC/TDF	22	60.1 (40.2, 80.0)
ISL 0.25 mg + DOR 100 mg (+/‐ 3TC)	19	79.8 (50.0, 109.6)
ISL 0.75 mg + DOR 100 mg (+/‐ 3TC)	18	47.1 (26.1, 68.2)
ISL 2.25 mg + DOR 100 mg (+/‐ 3TC)	16	24.0 (4.7, 43.4)

^a^The within‐group 95% CIs were calculated based on the t‐distribution.

## POSTERS

### ARV‐based Prevention: Perinatally Acquired HIV

#### P001 Increasing numbers of pregnancies to women with vertically‐acquired HIV in the UK: 2006 to 2021

H Peters, K Francis, L Bukasa, R Sconza, C Thorne

The Integrated Screening Outcomes Surveillance Service, part of the NHS Infectious Diseases in Pregnancy Screening Programme, UCL Great Ormond Street Institute of Child Health, London, UK

**Background**: Despite globally increasing numbers of reproductive‐aged women with vertically‐acquired HIV (WVHIV), knowledge gaps on their characteristics and pregnancy outcomes exist. We present population‐level pregnancy outcome data for this emerging cohort.

**Materials/methods**: UK surveillance of all pregnancies to women living with HIV, their infants and any children diagnosed with HIV has been carried out for >30 years, currently by the Integrated Screening Outcomes Surveillance Service (ISOSS), part of the NHS Infectious Diseases in Pregnancy Screening Programme, commissioned by NHSE. We analysed data on pregnancies in WVHIV diagnosed at <14 years, reported by 31/12/2021.

**Results**: Two hundred and two pregnancies to 131 WVHIV (37% UK‐born, 54% African‐born, 9% other) were reported since 2006 (none <2006): 81 had one pregnancy, 34 had two, 16 had ≥3. Proportion of pregnancies in WVHIV increased from 0.3% (15/5011) in 2006 to 2009 to 3.5% (83/2403) in 2018 to 2021, p < 0.001. Median age at diagnosis was 6 years (IQR 2 to 11). Most (81/131) were diagnosed in the UK, and 112/131 reported to ISOSS in childhood. Median age at expected date of delivery was 24 (IQR 20 to 27) for pregnancies to WVHIV and 33 years (IQR 29 to 37) for women with heterosexually‐acquired HIV (WHHIV). WVHIV conceived on ART in 81% of pregnancies, reaching 88% 2015 to 2021 (vs 77% for WHHIV). WVHIV had significantly lower first pregnancy CD4 count than WHHIV (≥500 cells/μL in 35% vs 42%, p < 0.001) and fewer had undetectable delivery viral load (dVL): overall 79% versus 84% for WHHIV (p = 0.127) had VL <50 copies/mL, increasing to 85% versus 93% in 2015 to 2021 (p < 0.001). Among pregnancies conceived on ART, 82% in WVHIV had undetectable dVL versus 94% in WHHIV (p < 0.001). Pregnancy outcomes for WVHIV were: 170 livebirths (84%), 10 miscarriages (5%), 18 terminations (9%) and four stillbirths (2%); 17% of livebirths were preterm, median birthweight was 3 kg (IQR 2.5 to 3.2). Of infants with complete follow‐up, one was diagnosed HIV‐positive (1/150, 0.66%).

**Conclusions**: In this growing sub‐population of WVHIV in the UK, HIV‐related markers have improved over time, with one case of second‐generation vertical transmission. Further work is needed to understand why fewer WVHIV have undetectable VL at delivery, as well as other areas of interest in this group, including sequential pregnancies and longer‐term outcomes of children born HIV‐free.

#### P002 HIV vertical transmission in England: the current picture

K Francis, H Peters, L Bukasa, R Sconza, C Thorne

The Integrated Screening Outcomes Surveillance Service, part of the NHS Infectious Diseases in Pregnancy Screening Programme, UCL Great Ormond Street Institute of Child Health, London, UK

**Background**: The UK has met 90‐90‐90 targets since 2017 and a major success is the low vertical HIV transmission rate (VTR). This reflects the high uptake of HIV antenatal testing (99.8%) and the impact of the NHS Infectious Diseases in Pregnancy Screening Programme (IDPS). A small number of vertical transmissions (VT) still occur in England, and it remains important to understand the contributing factors and contexts in these cases.

**Materials/methods**: The Integrated Screening Outcomes Surveillance Service (ISOSS), part of the IDPS, commissioned by NHSE, monitors all pregnancies to diagnosed women living with HIV (WLHIV) in England and their infants up to 18‐ to 24‐month antibody testing. Children diagnosed with vertically‐acquired HIV aged <16 years, born in England since 2006, are reported and investigated. Clinical Expert Review Panels (CERP) review circumstances surrounding transmissions and establish any contributing factors. We present VTR for infants born 2018 to 2019, and describe 13 VTs reported to ISOSS 01/06/2020 to 31/12/2021 and discussed by the CERP.

**Results**: There were three VTs among 1205 infants with known infection status born 2018 and 2019 to diagnosed women, with a VTR of 0.25% (95% CI 0.05 to 0.73). Maternal disengagement with healthcare services and late antenatal booking (≥20 weeks gestation) were identified as contributing factors by the CERP. Among the 13 VT cases, age at diagnosis ranged from birth to 7 years. Six children were born to women diagnosed before pregnancy, one to a woman during pregnancy, and six to women diagnosed postnatally. Most (12/13) children were born to women born outside the UK, with nine from sub‐Saharan Africa and three from Eastern Europe. Median maternal age at delivery was 34 years (IQR 31 to 39). In over half of cases (7/13), complicating issues during pregnancy included safeguarding, mental health issues and insecure housing were reported. Contributing factors identified by the CERP included seroconversion during pregnancy/breastfeeding, and among women aware of their diagnosis in pregnancy poor adherence and undisclosed breastfeeding.

**Conclusions**: The sustained low VTR reflects ongoing successes of the screening programme and clinical management. Increasing complexities can be seen in the small number of VTs still occurring in England, meaning ongoing monitoring and the insights provided by the CERP remain vital.

#### P003 Transfer of antiretroviral drugs into breastmilk: a prospective study from the Swiss Mother and Child HIV Cohort Study

K Aebi‐Popp^1^, C Kahlert^2^, P Crisinel^3^, L Decosterd^4^, S Alves Saldanha^4^, I Hösli^5^, B Martinez Tejada^6^, A Duppenthaler^7^, A Rauch^1^, C Marzolini ^8^

^1^Infectious Diseases, University Hospital Bern, Bern, Switzerland; ^2^Division of Infectious Diseases and Hospital Epidemiology, Cantonal Hospital St. Gallen, St. Gallen, Switzerland; ^3^Unit of Pediatric Infectious Diseases and Vaccinology, Service of Pediatrics, Women and Mother Child Department, Lausanne University Hospital, Lausanne, Switzerland; ^4^Service and Laboratory of Clinical Pharmacology, Lausanne University Hospital, Lausanne, Switzerland; ^5^Obstetrics, University Hospital Basel, Basel, Switzerland; ^6^Obstetrics Division, Geneva University Hospital, Geneva, Switzerland; ^7^Pediatrics, University Hospital Bern, Bern, Switzerland; ^8^Division of Infectious Diseases and Hospital Epidemiology, University Hospital Basel, Basel, Switzerland

**Introduction**: In 2018, Switzerland changed its guidelines to support women living with HIV who wish to breastfeed. The exposure of antiretroviral drugs (ARVs) in the breastmilk and the ingested daily dose by the breastfed infant are understudied notably for newer ARVs. The aim of this study was to measure concentrations of ARVs simultaneously in the breastmilk and in the maternal plasma in order to evaluate their transfer and estimate the daily infant ARV dose from breastfeeding.

**Methods**: Breastmilk and maternal plasma samples were collected at 1, 3 and 6 months after birth and ARVs were quantified using mass spectrometry.

**Results**: Twenty‐one mother‐child pairs were included. No vertical HIV transmission was detected in 20 newborns (one unknown). Non‐nucleoside reverse transcriptase inhibitors transferred well with efavirenz and nevirapine having the highest concentrations in the breastmilk and the infants. Integrase inhibitors transferred to variable extent, bictegravir and dolutegravir concentrations were generally lower in the breastmilk compared to those in infants. Most nucleoside reverse transcriptase inhibitors concentrated in the breastmilk but were present in low amount in the infants. The estimated infant daily dose from breastfeeding was low.

**Conclusion**: Most ARVs transferred well in the breastmilk but the daily ARV dose from breastfeeding was low and within the safety limit. Differential ARVs exposure was observed in infants with some ARVs being below or above their effective concentrations raising the concern of resistance development if HIV infection occurs. More data on this potential risk are warranted to better support breastfeeding in women living with HIV.

#### P004 Abstract withdrawn

#### P005 Could Dolutegravir/Lamivudine be a valid dual‐therapy option even in pregnancy? Data from a retrospective analysis

L Pagnucco ^1^, R Bruno^1^, V Zuccaro^1^, L Maiocchi^1^, S Novati^1^, M Roccio^2^, L Zanchi^2^, C Melito^2^, G Bossi^3^, A Borghesi^4^, R Gulminetti^1^

^1^Infectious Diseases Unit Department of Medical Science and Infectious Diseases, Fondazione Istituti di Ricovero e Cura a Carattere Scientifico Policlinico San Matteo, Pavia, Italy; ^2^Department of Obstetrics and Gynecology, Fondazione Istituti di Ricovero e Cura a Carattere Scientifico Policlinico San Matteo, Pavia, Italy; ^3^Pediatrics Unit, Fondazione Istituti di Ricovero e Cura a Carattere Scientifico Policlinico San Matteo, Pavia, Italy; ^4^Neonatal Intensive Care Unit, Fondazione Istituti di Ricovero e Cura a Carattere Scientifico Policlinico San Matteo, Pavia, Italy

**Background**: Numerous data from clinical trials and real‐life studies has shown that the two‐drug regimen dolutegravir/lamivudine was non‐inferior to three‐drug regimens [1,2]. Regarding pregnancy, while guidelines recommend regimens including dolutegravir plus a backbone of two NRTIs (ABC/3TC or TDF/FTC) [3‐6], no data on Dolutegravir/Lamivudine (DTG/3TC) use are available.

**Materials and methods**: We conducted a retrospective analysis from October 2017 to June 2022 recruiting pregnant women treated with DTG+2NRTI (TDF/FTC or ABC/3TC) or DTG/3TC. The primary efficacy outcome was a HIV‐RNA <20 copies/mL at delivery and the primary safety outcome was the occurrence of drug‐related adverse events in mothers and infants until the post‐partum visit. Variables investigated were maternal and gestational age at delivery, time of ART initiation, mode of delivery, birth weight, maternal CD4 count and HIV‐RNA during I trimester, II trimester and at delivery and infant HIV‐1 status.

**Results**: Twenty‐four pregnant women, both naïve patients and patients already on cART before pregnancy, treated with dolutegravir‐based therapy; 13 on DTG+2NRTI (four DTG/TDF/FTC, nine DTG/ABC/3TC) and 11 on DTG/3TC were enrolled. We compared the two groups. In the DTG/2NRTI group four patients were naïve; at delivery three patients had a detectable HIV‐RNA (one patient naïve 35 copies/mL while two patients >1500 copies/mL due to poor therapeutic compliance), the median CD4 count was 570 cells/mcL in the first trimester and 588 cells/mcL at delivery. Among the 11 patients in the DTG/3TC group three were naïve; at delivery only one patient had a detectable HIV‐RNA (one naïve 53 copies/mL); one patient experienced a viral blip during third trimester probably caused by taking vitamin/iron supplements and cART at the same time but HIV‐RNA returned negative at time of delivery; median CD4 count was 554 cells/mcL in the first trimester and 555 cells/mcL at delivery. We found no differences in births less than 37 weeks. All infants received antiretroviral prophylaxis and have tested negative on follow‐up. No neonatal malformations were found.

**Conclusions**: With the limits linked to the small sample size, we found no major differences in the two groups. The DTG/3TC regimen may represent a therapeutic option in patients in whom, for various reasons, DTG+2NRTI regimen is not indicated. Further data and studies are needed to support the evidence of efficacy and safety of DTG/3TC in pregnancy.

**References**

1. Cahn P, Sierra Madero J, Arribas JR, Antinori A, Ortiz R, Clarke AE, et al. Three‐year durable efficacy of dolutegravir plus lamivudine in antiretroviral therapy ‐ naive adults with HIV‐1 infection. AIDS. 2022;36:39‐48.

2. Llibre JM, Brites C, Cheng CY, Osiyemi O, Galera C, Hocqueloux L, et al. Efficacy and safety of switching to the 2‐drug regimen dolutegravir/lamivudine versus continuing a 3‐ or 4‐drug regimen for maintaining virologic suppression in adults living with HIV‐1: week 48 results from the phase 3, non‐inferiority SALSA randomized trial. Clin Infect Dis. 2022 Mar 2:ciac130. doi: 10.1093/cid/ciac130.

3. Crawford M, van Wyk J, Aboud M, Vannappagari V, Romach B, Curtis L, et al. Postmarketing surveillance of pregnancy outcomes with dolutegravir use. J Acquir Immune Defic Syndr. 2020;83:e2‐5.

4. Davey S, Ajibola G, Maswabi K, Sakoi M, Bennett K, Hughes MD, et al. Mother‐to‐child HIV transmission with in utero dolutegravir vs. efavirenz in Botswana. J Acquir Immune Defic Syndr. 2020;84:235‐41.

5. Kintu K, Malaba TR, Nakibuka J, Papamichael C, Colbers A, Byrne K, et al; DolPHIN‐2 Study Group. Dolutegravir versus efavirenz in women starting HIV therapy in late pregnancy (DolPHIN‐2): an open‐label, randomised controlled trial. Lancet HIV. 2020;7:e332‐9.

6. European AIDS Clinical Society. EACS Guidelines Version 11.0 [Internet]. October 2021 [cited 2022 Aug 23]. Available from: http://www.eacsociety.org/guidelines/eacs‐guidelines/eacs‐guidelines.html.

#### P006 Transplacental transfer of bictegravir versus dolutegravir in human placenta

A Mohammed ^1,2^, M Lehtonen^3^, N Tepponen^3^, M Forsberg^3^, K Vähäkangas^3^

^1^School of Pharmacy, Faculty of Health Sciences, University of Eastern Finland, Finland; ^2^College of Pharmacy, University of Duhok, Kurdistan, Iraq; ^3^School of Pharmacy, University of Eastern Finland, Kuopio, Finland

Bictegravir (BIC) and dolutegravir (DTG) are second‐generation integrase strand transfer inhibitors (INSTI). For treatment of HIV, BIC is combined with two nucleoside reverse transcriptase inhibitors, emtricitabine (FTC) and tenofovir alafenamide (TAF), and available as once‐daily fixed dosage. DTG is available in combination with either FTC and TAF, or abacavir and lamivudine. INSTI‐based combination regimens are recommended as first‐line treatment in HIV infection. BIC is not being used during pregnancy and the data on its pharmacokinetics during pregnancy is scarce. Currently, the use of DTG combinations at conception is questioned due to increased cases of neural defects [1]. BIC exhibits potent antiviral activity and high genetic barrier to resistance in vitro [2,3]. In clinical trials, BIC/FTC/TAF had high safety and better tolerability and non‐inferior viral suppression than the DTG‐containing regimen [4]. It is important to note that BIC has been non‐toxic to reproductive and developmental systems in rats and rabbits [5]. The use of antiviral drug combinations, including INSTIs, during pregnancy has significantly reduced the vertical transmission of HIV from mother to foetus. There are ethical limitations with clinical studies with pregnant women. Ex vivo human placenta provides a reliable alternative to probe transplacental transfer of chemicals safely. There are limited data on the transplacental transfer of BIC and DTG. The only study available on transfer of BIC in open circuit perfusion showed low transfer. The aim of this study is to investigate the transplacental transfer of bictegravir versus dolutegravir across human placenta using a pre‐validated, dual re‐circulating human placental perfusion of a single cotyledon. A set of four to five perfusions of clinically relevant concentrations of BIC and DTG are being conducted. According to the preliminary data, bictegravir crossed human placenta at all tested concentrations (500, 3000, 6000 ng/mL) but the transfer was slower than that of the reference compound, antipyrine, which diffuses passively across the placenta. The transfer of DTG at concentration 3000 ng/mL in one perfusion was slower than that of antipyrine and BTC. However, more perfusions will be performed to better understand the transfer of BIC and DTG.

**References**

1. Zash R, Holmes L, Diseko M, Jacobson DL, Brummel S, Mayondi G, et al. Neural‐tube defects and antiretroviral treatment regimens in Botswana. N Engl J Med. 2019;381:827‐40.

2. Deeks ED. Bictegravir/emtricitabine/tenofovir alafenamide: a review in HIV‐1 infection. Drugs. 2018;78:1817‐28.

3. Tsiang M, Jones GS, Goldsmith J, Mulato A, Hansen D, Kan E, et al. Antiviral activity of bictegravir (GS‐9883), a novel potent HIV‐1 integrase strand transfer inhibitor with an improved resistance profile. Antimicrob Agents Chemother. 2016;60:7086‐97.

4. Gallant J, Lazzarin A, Mills A, Orkin C, Podzamczer D, Tebas P, et al. Bictegravir, emtricitabine, and tenofovir alafenamide versus dolutegravir, abacavir, and lamivudine for initial treatment of HIV‐1 infection (GS‐US‐380‐1489): a double‐blind, multicentre, phase 3, randomised controlled non‐inferiority trial. Lancet. 2017;390:2063‐72.

5. European Medicines Agency. Biktarvy: EPAR – Product Information (Summary of Product Characteristics) [Internet];1‐104. 2018. Available from: https://www.ema.europa.eu/en/medicines/human/EPAR/biktarvy.

### ARV‐based Prevention: PEP/PrEP/TasP

#### P007 Pre‐exposure prophylaxis (PrEP) in Poland 2017 to 2021: lessons from a country with no national PrEP programme

B Szetela ^1^, I Cielniak^2^, J Kubicka^3^, T Rojewski^4^, A Katafias^5^, P Jakubowski^6^, M Hlebowicz^7^, M Sitko^8^, J Loster^9^, O Jablonowska^10^, A Cybula^11^, B Aksak‐Wąs^12^, K Gierlotka^13^, M Bociaga‐Jasik^14^, M Parczewski^12^, R Szymanski^15^, W Bludzin^16^, A Grzeszczuk^17^, W Niemczyk^18^, K Giniewicz^19^, M Rosinska^20^, J Gasiorowski^21^

^1^Infectious Diseases, Liver Disease and Acquired Immune Deficiencies, Wroclaw Medical University, Wroclaw, Poland; ^2^Department of Infectious Diseases, Hospital for Infectious Diseases in Warsaw, Warsaw, Poland; ^3^Sexually Transmitted Infection, Smart Life Clinic, Warsaw, Poland; ^4^Sexually Transmitted Infection, Poznanskie Centrum PrEP, Poznan, Poland; ^5^STI, Centrum PrEP, Gdansk, Poland; ^6^Infectious Diseases, Pomeranian Hospitals, Gdansk, Poland; ^7^Infectious Diseases, Department of Family Medicine and Infectious Diseases University of Warmia and Mazury, Olsztyn, Poland; ^8^Infectious Diseases, The University Hospital in Krakow, Krakow, Poland; ^9^STI, Solimed Jakub Loster, Wieliczka, Poland; ^10^Department of Dermatology and Venereology, Medical University of Lodz, Lodz, Poland; ^11^Department of Infectious Diseases, Tropical Disease and Hepatology, Warsaw Medical University, Warszawa, Poland; ^12^Department of Infectious, Tropical Diseases and Acquired Immunodeficiency, Pomeranian Medical University, Szczecin, Poland; ^13^Infectious Diseases, Hospital for Infectious Diseases in Bydgoszcz, Bydgoszcz, Poland; ^14^Department of Infectious and Tropical Diseases, Jagiellonian University in Krakow, Krakow, Poland; ^15^STI, Rafal Szymanski Medical Practice, Warszawa, Poland; ^16^Department of Infectious Diseases, Provincial Hospital in Opole, Opole, Poland; ^17^Department of Infectious Diseases and Neuroinfections, Medical University of Bialystok, Bialystok, Poland; ^18^Economics and Management Sciences, Nicolaus Copernicus University, Torun, Poland; ^19^Statistical Analysis Centre, Wroclaw Medical University, Wroclaw, Poland; ^20^Department of Epidemiology, National Institute of Public Health, Warszawa, Poland; ^21^STI, All Saint's Clinic, Wroclaw, Poland

**Background**: PrEP has been the mainstay of HIV prophylaxis in key populations, especially among MSM [1‐3]. Nonetheless, there are still countries with no national PrEP programmes leaving many individuals at risk of infection. As medical professionals from Polish AIDS Scientific Society we decided to develop PrEP guidelines in 2015 [1] to help create PrEP clinics in Poland. Generic TDF/FTC was available at a cost of 28 Euros/30 tablets.

**Materials and methods**: Questionnaires among medical professionals during an annual HIV conference in Poland were taken to ascertain attitudes and readiness to provide PrEP care. PrEP.edu.pl webpage was created as information exchange point for the whole country. Seventeen dedicated PrEP clinics were systematically created in largest cities. PrEP evaluation was based on actual prescriptions from Central E‐Health Systems, wholesale volume to pharmacies and data from PrEP clinics. HIV prevalence and incidence among MSM in Poland were calculated based on VCT data, national registries and national reports for 2017 to 2021 [4].

**Results**: There was a steady increase in PrEP patients with around 1000 new patients every year (smaller increase only in 2020) leading to over 4400 PrEP users at the end of 2021 (Table 1). Most of the clinics were private with no reimbursement of medical care, testing or medication. There were 15 HIV infections during 7898 PY of follow‐up giving incidence of 0.19/100PY. Based on HIV incidence of 3.8/100PY among MSM at testing sites in Poland the relative risk reduction in our cohort was 95% and NNT 27 (almost equal cost of PrEP compared to cART). With 1200 annual new HIV infections among MSM in Poland we require 32,400 MSM on PrEP to prevent them. There was no change in HIV prevalence or incidence on national level during observation period.

**Conclusions**: Despite no financial support from the Health Ministry and no reimbursement, almost 5000 patients received access to PrEP and STI care. The programme was highly efficacious with incidence similar to other PrEP projects with highly adherent and dedicated patients [5‐10]. Improvement in access is urgently needed, new clinics have to be set up also outside of large cities and universal reimbursement of medical care and medication implemented to see actual reduction in HIV incidence.

**Table:** 

**Abstract P007 – Table 1**. PrEP use characteristics and HIV incidence in Poland 2017‐2021.						
	10 to 12 2017	2018	2019	2020	2021	2017 to 2021
PrEP users at PrEP clinics, n	102	1128	2033	2684	3675	NA
PrEP users outside of PrEP clinics, n	NA	NA	394	NA	NA	NA
Total estimated PrEP users, n	102	1128	2427	3220	4410	NA
MSM PrEP users, %	NA	NA	NA	NA	NA	99
HTX PrEP users, %	NA	NA	NA	NA	NA	1
On‐demand PrEP users, %	2	15	20	17	35	NA
HIV incidence among MSM at VCTs in Poland, /100PY	NA	NA	4.3	3.4	3.7	3.8

HTX, heterosexual; PrEP, pre‐exposure prophylaxis; VCT, voluntary counselling and testing site.

**References**

1. Parczewski M, Jablonowska E, Witak‐Jedra M. Zasady opieki nad osobami zakażonymi HIV ‐ zalecenia PTN AIDS [Internet]. Warsaw 2021 [cited 2022 Aug 23] [in Polish]. Available from: http://www.ptnaids.pl/images/pliki/wytyczne_AIDS_2021.pdf.

2. European AIDS Clinical Society. Guidelines v. 11 [Internet]. 2021 [cited 2022 Aug 23]. Available from: https://www.eacsociety.org/media/final2021eacsguidelinesv11.0_oct2021.pdf.

3. Consolidated guidelines on HIV prevention, testing, treatment, service delivery and monitoring: recommendations for a public health approach. Geneva: World Health Organization; 2021.

4. Rosinska M, Wagner N. HIV incidence based on serological tests to differentiate between early and lasting infection. Warsaw 2019 for National AIDS Centre [in Polish]. Available on request from the Ministry of Health.

5. Grant RM, Lama J, Anderson P, McMahan V, Liu A, Vargas L, et al. Preexposure chemoprophylaxis for HIV prevention in men who have sex with men. N Engl J Med. 2010;363:2587‐99.

6. McCormack S, Dunn D, Desai M, Dolling D, Gafos M, Gilson R, et al. Pre‐exposure prophylaxis to prevent the acquisition of HIV‐1 infection (PROUD): effectiveness results from the pilot phase of a pragmatic open‐label randomised trial. Lancet. 2016;387:53‐60.

7. Hare B, Coll P, Ruane P, Molina J‐M, Mayer K, Jessen H, et al. The phase 3 discover study: daily F/TAF or F/TDF for HIV preexposure prophylaxis. Conference on Retroviruses and Opportunistic Infections; 2019 Mar 4‐7; Seattle, WA, USA. Oral abstract LB 104LB.

8. Molina JM, Charreau I, Spire B, Cotte L, Chas J, Capitant C, et al. Efficacy, safety, and effect on sexual behaviour of on‐demand pre‐exposure prophylaxis for HIV in men who have sex with men: an observational cohort study. Lancet HIV. 2017;4:e402‐10.

9. Molina J‐M, Ghosn J, Delaugerre C, Pialoux G, Katlama C, Slama L, et al. Incidence of HIV infection with daily or on‐demand oral PrEP with TDF/FTC in France. Conference on Retroviruses and Opportunistic Infections; 2021 Mar 6‐10; virtual. Oral abstract 148.

10. Landovitz RJ, Donnell D, Tran H, Kallas EG, Magnus M, Marzinke M, et al. Updated efficacy, safety, and case studies in HPTN 083: CAB‐LA vs TDF/FTC for PrEP. Conference on Retroviruses and Opportunistic Infections; 2022 Feb 12‐16; virtual. Oral abstract 96.

#### P008 Comparison of HIV incidence as determined by two different recency assays in Ugandan women

S Cox ^1^, E Grebe^2^, A Kintu^3^, Y Shao^4^, F Matova Kiweewa^5^, Z Lukyamuzi^5^, F Kiweewa^6^, S Facente^7^, R Ebrahimi^4^, C Carter^3^, C Callebaut^1^, J Baeten^3^, M Das^3^

^1^Clinical Virology, Gilead Sciences, Foster City, CA, USA; ^2^Eduard Grebe Consulting, Eduard Grebe Consulting, Cape Town, South Africa; ^3^Clinical Research, Gilead Sciences, Foster City, CA, USA; ^4^Biostatistics, Gilead Sciences, Foster City, CA, USA; ^5^Clinical Research, Makerere University‐Johns Hopkins University Research Collaboration Care Ltd / Research Collaboration, Kampala, Uganda; ^6^Clinical Research, Africa Medical and Behavioral Sciences Organization, Hoima, Uganda; ^7^Facente Consulting, Richmond, CA, USA

**Background**: HIV‐1 recent infection testing algorithms (RITAs) use recency assays to estimate population level HIV incidence rates (IRs), and are currently being employed in PrEP trials to estimate background HIV incidence rates (bHIV‐IR). The SIENA study was conducted to determine the HIV incidence rate among young women (18 to 25 years old with unknown HIV status) in and around central and mid‐western Uganda using RITAs. Here, we report bHIV‐IR estimates derived from two recency assay platforms, the Abbott ARCHITECT HIV Ag/Ab Combo Assay (ARCHITECT) and the Sedia HIV‐1 Limiting Antigen Avidity Enzyme ImmunoAssay (LAg‐EIA), the latter of which has been validated in large HIV surveillance studies.

**Figure:**
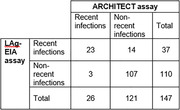
**Abstract P008 – Figure 1**. Comparison of recent versus non‐recent infection samples by the LAg‐EIA assay and the ARCHITECT assay. Out of 147 samples 23 were identified the as recent infections by both assays and 107 were identified as non‐recent by both assays. Fourteen samples were identified as non‐recent by the ARCHICTECT assay while identified as recent by the LAg‐EIA and three were identified as non‐recent by the LAg‐EIA assay and recent by the ARCHITECT assay.

**Methods**: Diagnosis of HIV was confirmed by positive results on both the Alere Determine HIV‐1/2 and Oraquick HIV‐1/2 rapid tests, per Ugandan guidelines. Positive samples were tested for recent infection using the LAg‐EIA (Sedia Biosciences, Beaverton, OR) and the ARCHITECT (LabCorp, Indianapolis, IN). HIV incidence was estimated using a RITA with a viral load (VL) cutoff of ≥75 copies/mL (COBAS TaqMan HIV‐1 Test, LabCorp, Indianapolis, IN) and study population specific mean duration of recent infection and false recent ratios.

**Results**: Of 743 women enrolled 191 were diagnosed with HIV, 44 (23%) had a VL of <75 copies/mL. Out of the remaining 147 samples, the LAg‐EIA and ARCHITECT assays identified 37 (19.4%) and 26 (13.6%) as recent, respectively (Figure 1). A total of 130 samples were identified the same by both assays. Out of the 17 samples that were classified differently, three were called recent infections by the ARCHITECT but not by the LAg‐EIA, and 14 were called recent by the LAg‐EIA but long‐term by the ARCHITECT. Based on the recency results, the calculated bHIV‐IR was 11.4/100 PY with the LAg‐EIA and 6.84/100 PY with the ARCHITECT.

**Conclusions**: In a population of young women in Uganda, both the LAg‐EIA and ARCHITECT recency assays estimated high bHIV‐IRs. While there was some incongruence between the individual assays, the findings suggest high‐level HIV transmission within this key population, providing evidence of microepidemics within Uganda and demonstrating the usefulness of RITAs for estimating bHIV in next‐generation PrEP trials.

#### P009 Prescription adherence and persistence on oral pre‐exposure prophylaxis (PrEP) among PrEP‐naive (PN) individuals after FTC/TAF approval in the United States (US)

R Elion^1^, J Gruber^2^, J Radtchenko ^1^, M Dunbar^2^, K Mayer^3^, G Huhn^4^, K Mounzer^5^, A Mills^6^

^1^Analytics, Trio Health, Louisville, CO, USA; ^2^Medical Affairs Research ‐ Virology, Gilead Sciences, Foster City, CA, USA; ^3^The Fenway Institute, Fenway Health, Boston, MA, USA; ^4^The Ruth M. Rothstein Core Center, Rush University Medical Center, Chicago, IL, USA; ^5^Philadelphia Fight Community Health Centers, School of Medicine at the University of Pennsylvania, Philadelphia, PA, USA; ^6^HIV, Men's Health, Los Angeles, CA, USA

**Background**: We evaluated utilization of emtricitabine/tenofovir disoproxil fumarate or tenofovir alafenamide (FTC/TDF, FTC/TAF) among PN after the approval of FTC/TAF for PrEP in the US.

**Table:** 

**Abstract P009 – Table 1**. Characteristics of individuals dispensed oral PrEP after October 2019.		
	PrEP‐naive (n = 1330)	
n (%) unless specified	FTC/TDF n = 186	FTC/TAF n = 1144
Male	100 (54)	760 (66)^e^
Female	19 (10)^c^	12 (1)
Transgender	0 (0)	1 (0)
Unspecified gender	67 (36)	371 (32)
White race	79 (42)	699 (61)^c^
Black	19 (10)	124 (11)
Asian, Indian, Pacific Islander	13 (7)	91 (8)
Unspecified	75 (40)^c^	230 (20)
Commercial insurance	97 (52)	735 (64)^d^
Medicare	2 (1)	26 (2)
Medicaid	19 (10)^d^	72 (6)
Ryan White	0 (0)	3 (0)
Other non‐commercial plan or self‐pay	6 (3)	24 (2)
Unknown	62 (33)^d^	284 (25)
Age 18 to 25 years	30 (16)	139 (12)
Age 26 to 50 years	133 (72)	816 (71)
Age 51+ years	23 (12)	189 (17)
High‐risk behavior (based on ICD‐10 codes)^a^	115 (62)	862 (75)^c^
PDC (%), mean (SD)^b^	87.2 (19.8)	86.3 (17.3)
Number of dispenses, mean (SD)	4.5 (3.9)	9.9 (6.2)^c^
Days supplied, mean (SD)	141.9 (122.7)	311.5 (189.2)^c^
Follow‐up months, mean (SD)	15.1 (6.3)	14.7 (5.8)
PDC >50%	171 (92)	1083 (95)
PDC >70%	160 (86)	962 (84)
PDC >80%	140 (75)	863 (75)

^a^High‐risk behavior: ICD‐10 codes for high‐risk sexual behavior or exposure to communicable diseases; ^b^proportion days covered; ^c^p < 0.001 FTC/TDF vs FTC/TAF; ^d^p < 0.05; ^e^p = 0.001.

**Figure:**
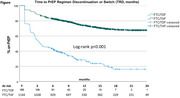
**Abstract P009 – Figure 1**. Time to PrEP regimen discontinuation or switch (TRD, months).

**Methods**: EMR and dispensing data from Trio Health were used for this retrospective study. The study included HIV‐negative PN ≥18 years with first dispense of daily oral PrEP (≥30‐day supply) between 10/19 and 5/21 followed for ≥6 months; individuals with HBV or post‐exposure prophylaxis were excluded. Prescription adherence, measured as proportion of days covered (PDC; mean and proportion with PDC ≥50, 70, and 80%) and time to regimen discontinuation (no drug >3 months) or switch (TRD; Kaplan‐Meier analysis) were compared between regimens. Characteristics associated with PDC and time to first regimen stop (switch/discontinuation) were evaluated using generalized linear regression and Cox proportional hazard models, respectively.

**Results**: Of 1330 PrEP starts, 86% (1144) were dispensed FTC/TAF versus 14% FTC/TDF (186). Baseline characteristics differed by regimen (Table 1). While PDC was similar for both regimens, FTC/TAF had higher number of dispenses and mean days supplied versus FTC/TDF; mean days of follow‐up were similar. F/TAF users had longer TRD (mean 20.2 vs 8.5 months, log‐rank p < 0.001); median TRD was 3.9 months for F/TDF and not reached for FTC/TAF (Figure 1). A higher proportion of PN on FTC/TDF discontinued (46% vs 24% FTC/TAF) and switched (26% vs 2% FTC/TAF) regimen (both p < 0.001). After accounting for gender, race, payer, age, high‐risk behavior, FTC/TDF had a higher risk of discontinuation or switch (HR 4.9, CI 3.9 to 6.2); Black race was also associated with higher risk of discontinuation or switch (HR 1.8, CI 1.4 to 2.4). Results were similar when considering only discontinuation (censoring at time of switch or loss to follow‐up). Older age was identified as the primary driver of PDC controlling for other factors (age 26 to 50, RR 1.05, CI 1.01 to 1.1; age >50, RR 1.09, CI 1.03 to 1.14; reference age 18 to 25).

**Conclusions**: In this study PN adults dispensed FTC/TAF had greater number of dispenses, mean days supplied, and were less likely to discontinue or switch from FTC/TAF compared to FTC/TDF. Older age was the primary driver of increased PDC when considering other factors, including demographics, insurance and regimen.

#### P010 Viral hepatitis and human papillomavirus vaccination during pre‐exposure prophylaxis: factors associated with missed vaccination

A Raccagni ^1^, D Ceccarelli^2^, B Trentacapilli^1^, L Galli^2^, R Lolatto^2^, D Canetti^2^, E Bruzzesi^1^, C Candela^1^, A Castagna^1^, S Nozza^2^

^1^Infectious Diseases, Vita‐Salute San Raffaele University, Milan, Italy; ^2^Infectious Diseases, San Raffaele Scientific Institute, Milan, Italy

**Introduction**: PrEP visits are an opportunity for HAV, HBV and HPV vaccination. Aim was to evaluate factors associated with missing ≥1 vaccination among men who have sex with men (MSM) receiving PrEP.

**Materials and methods**: PrEP users at the Infectious Diseases Unit of IRCCS San Raffaele, Milan, with ≥1 follow‐up visit (May 2017 to 2022) were included; dropout was defined as no visit for ≥12 months. Participants were considered protected if: i) prior to PrEP access, a positive serology (IgG‐HAV+, HbsAb >10 mUI/mL) or vaccination history were recorded; ii) after starting PrEP, ≥1 dose of each vaccination was administered. Individuals were considered fully protected if they received before or during PrEP access HAV vaccination/infection, HBV vaccination/infection, and HPV vaccination if <46 years old. Chi‐square test or Kruskal‐Wallis test were used to compare characteristics of those fully, partially and not protected. Factors associated with the probability of missing at least one vaccination were assessed by multivariable logistic regression and a classification tree analysis.

**Results**: Overall 473 MSM were considered: 146 (31%) were fully vaccinated, 231 (48%) partially and 96 (20%) not; 131 were previously protected for HAV, 151 had negative IgG‐HAV and 103 were vaccinated during PrEP visits; 200 previously for HBV, 88 had HbsAb ≤10 and 44 were vaccinated; 12 previously for HPV, 209 were vaccinated. Individuals’ characteristics in Table 1. Daily‐based PrEP users (fully 93 (63.7%), partially 107 (46.3%), not protected 40 (41.7%), p = 0.001) and those with a baseline STI diagnosis (43 (29.5%), 55 (23.8%), 15 (15.6%), p = 0.048) were more frequently fully vaccinated. At multivariable analysis, the risk of having missed ≥1 vaccination was lower among those receiving daily‐based PrEP (aOR 0.47, 95% CI 0.31 to 0.70, p = 0.034), with a Smith index 20 to 29 (aOR 0.34, 95% CI 0.12 to 0.88, p = 0.045) and >30 (aOR 0.29, 95% CI 0.08 to 0.94, p < 0.001). The classification tree analysis showed that among daily‐based PrEP users, who received a previous and baseline STI diagnosis, there was a lower chance of missing a vaccination (probability 44%) (Figure 1).

**Figure:**
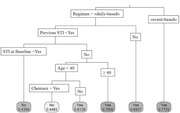
**Abstract P010 – Figure 1**. Decision tree for missing a vaccination. No: No vaccination is missing; Yes: At least one vaccination is missing.

**Conclusions**: Daily‐based PrEP was associated with full protection for HAV, HBV and HPV. Strategies targeting individuals at risk of missing vaccinations need to be implemented, with a main focus on event‐based users.

**Table:** 

**Abstract P010 – Table 1**. Individuals' characteristics among those fully protected, partially protected and not protected for HAV, HBV or HPV.					
	Overall (n = 473)	Not protected (n = 96)	Partially protected (n = 231)	Fully protected (n = 146)	p‐value
Age	34.6 (30.6 to 39.9)	33.9 (28.4 to 38.0)	34.9 (31.1 to 41.1)	34.9 (30.6 to 39.7)	p = 0.052
Ethnicity					p = 0.054
Asian	2 (0.42%)	1 (1.04%)	0 (0.00%)	1 (0.68%)	
Caucasian	460 (97.3%)	91 (94.8%)	225 (97.4%)	144 (98.6%)	
Hispanic	6 (1.27%)	1 (1.04%)	5 (2.16%)	0 (0.00%)	
Black	5 (1.06%)	3 (3.12%)	1 (0.43%)	1 (0.68%)	
Education					p = 0.784
Middle school	11 (2.33%)	2 (2.08%)	4 (1.73%)	5 (3.42%)	
High school	164 (34.7%)	31 (32.3%)	80 (34.6%)	53 (36.3%)	
University	298 (63.0%)	63 (65.6%)	147 (63.6%)	88 (60.3%)	
Partners					p = 0.193
0 to 9	192 (40.6%)	33 (34.4%)	105 (45.5%)	54 (37.0%)	
10 to 19	156 (33.0%)	41 (42.7%)	65 (28.1%)	50 (34.2%)	
20 to 49	102 (21.6%)	19 (19.8%)	48 (20.8%)	35 (24.0%)	
50 or more	23 (4.86%)	3 (3.12%)	13 (5.63%)	7 (4.79%)	
Chemsex (Yes)	222 (46.9%)	45 (46.9%)	105 (45.5%)	72 (49.3%)	p = 0.765
Smith Index					p = 0.026
10 to 19	42 (8.88%)	6 (6.25%)	29 (12.6%)	7 (4.79%)	
20 to 29	245 (51.8%)	44 (45.8%)	122 (52.8%)	79 (54.1%)	
30 or more	186 (39.3%)	46 (47.9%)	80 (34.6%)	60 (41.1%)	
Previous STI (Yes)	249 (52.6%)	50 (52.1%)	116 (50.2%)	83 (56.8%)	p = 0.451
Baseline STI (Yes)	113 (23.9%)	15 (15.6%)	55 (23.8%)	43 (29.5%)	p = 0.048
PrEP regimen					p = 0.001
Event‐based	233 (49.3%)	56 (58.3%)	124 (53.7%)	53 (36.3%)	
Daily‐based	240 (50.7%)	40 (41.7%)	107 (46.3%)	93 (63.7%)	
Dropout (Yes)	35 (7.40%)	9 (9.38%)	17 (7.36%)	9 (6.16%)	p = 0.646

#### P011 Evaluation of doravirine/lamivudine/tenofovir disoproxil fumarate for non‐occupational post‐exposure prophylaxis, a prospective open‐label study (DORAVIPEP)

A Inciarte, A Ugarte, B Torres, M Martínez‐Rebollar, M Laguno, J Ambrosioni, I Chivite, D Aguero, V Rico, L Berrocal, A González‐Cordón, P Puerta, L De La Mora, E de Lazzari, S Herrera, N Garcia‐Pouton, M Hernandez‐Meneses, R Alonso, P Monzo, P Callau, R Aguiló, E Fernández, L Barrero, E Solbes, E Martínez, J Blanco, J Miró, A Soriano, J Mallolas

Infectious Disease HIV Unit, Hospital Clinic Barcelona, Barcelona, Spain

**Background**: Most guidelines still recommend multiple pill regimens for post‐exposure prophylaxis (PEP), and completion rates for PEP are often low [1,2]. Few studies assess safety, tolerability, and adherence to new single tablet regimens (STR) [3]. We evaluated the combination of doravirine/lamivudine/tenofovir as STR for non‐occupational PEP [4‐7].

**Methods and materials**: This is a prospective, open‐label, single‐arm study. Individuals attending the emergency room due to potential sexual exposure to HIV and who met the criteria for PEP received doravirine/lamivudine/tenofovir. The primary endpoint was PEP non‐completion on day 28, and the secondary endpoints were adverse effects, adherence, and rate of seroconversions. Follow‐up consultations were appointed on days 10, 60, and 120. Clinical trials.gov number: NCT04233372.

**Results**: From 01‐09‐2020 to 14‐03‐2022, 399 individuals were enrolled in the study. The median age was 30 (27 to 36) years, and 91% (n = 365) were males. The mode of exposure was HSH in 84% (n = 331) and risk assessment as ascertained by the treating provider was high in 97% (n = 385) of the cases. PEP users self‐referred use of recreational drugs in 30% (n = 109) of cases. PEP non‐completion at day 28 was 26% (n = 103) (95% CI 22% to 30%), reasons for non‐completion were: loss to follow‐up 92 (89%), intolerance 9 (9%) and patients decision/withdrawal consent 2 (2%). In the multivariate regression model, older age for a patient makes it less likely for him to discontinue the treatment prematurely 0.95 (0.92 to 0.98) p = 0.0016. Adverse events were reported by 70 (18%) patients during the treatment period. Gastrointestinal symptoms were the most common, 51% (n = 49), followed by neurological, 30% (n = 29). There were no potentially life‐threatening (grade IV) adverse events. Most of the adverse events were mild and self‐limiting, 83% (n = 82). Adherence to PEP in the assessed users was 96% (337/351) and 99% (285/289) by self‐report and pill count data at day 10 and week 4, respectively. There were no cases of HIV transmission in this cohort.

**Conclusions**: Doravirine/lamivudine/tenofovir is a well‐tolerated option for once‐daily PEP that compares favourably with other recommended PEP regimens.

**References**

1. Parkin JM, Murphy M, Anderson J, El‐Gadi S, Forster G, Pinching AJ. Tolerability and side‐effects of post‐exposure prophylaxis for HIV infection [letter]. Lancet. 2000;355:722‐3.

2. Mayer KH, Mimiaga MJ, Gelman M, Grasso C. Raltegravir, tenofovir DF, and emtricitabine for postexposure prophylaxis to prevent the sexual transmission of HIV: safety, tolerability, and adherence. J Acquir Immune Defic Syndr. 2012;59:354‐9.

3. Inciarte A, Leal L, González E, León A, Lucero C, Mallolas J, et al. Tenofovir disoproxil fumarate/emtricitabine plus ritonavir‐boosted lopinavir or cobicistat‐boosted elvitegravir as a single‐tablet regimen for HIV post‐exposure prophylaxis. J Antimicrob Chemother. 2017;72:2857‐61.

4. Doravirine. In: Drugs and Lactation Database (LactMed). Bethesda (MD): National Library of Medicine (US); 15 June 2020.

5. Boyle A, Moss CE, Marzolini C, Khoo S. Clinical pharmacodynamics, pharmacokinetics, and drug interaction profile of doravirine. Clin Pharmacokinet. 2019;58:1553‐65.

6. Martin EA, Lai MT, Ngo W, Feng M, Graham D, Hazuda DJ, et al. Review of doravirine resistance patterns identified in participants during clinical development. J Acquir Immune Defic Syndr. 2020;85:635‐42.

7. Wandeler G, Buzzi M, Anderegg N, Sculier D, Béguelin C, Egger M, et al. Virologic failure and HIV drug resistance on simplified, dolutegravir‐based maintenance therapy: systematic review and meta‐analysis. F1000Res. 2018;7:1359.

#### P012 Willingness to use long‐acting injectable PrEP among key populations at a large HIV prevention clinic in Kampala, Uganda

D Lukubuya ^1^, M Baguma^1^, A Kaguta^1^, W Nambatya^1^, P Kyambadde^2^, E Katana^3^, T Muwonge^4^, E Odongpiny^5^

^1^Department of Pharmacy, Makerere University, Kampala, Uganda; ^2^Mulago Clinic, Most At Risk Populations Initiative, Kampala, Uganda; ^3^Clinical Epidemiology, College of Health Sciences, Makerere University, Kampala, Uganda; ^4^Clinical Research, Infectious Diseases Institute, Makerere University, Kampala, Uganda; ^5^Pharmacy Department, Infectious Diseases Institute, Makerere University, Kampala, Uganda

**Background**: Despite the success of the HIV prevention programmes, there are challenges with high drop out in some settings [1]. This is partly attributed to challenges with use of daily oral PrEP formulations [2]. Food and Drug Administration and World Health Organization recently approved long‐acting injectable formulations of PrEP (LAI‐PrEP) that is cabotegravir^®^ injection [3]. LAI‐PrEP offers advantages such as being discreet and hence causing less stigma, and could be a suitable alternative [4]. We aimed to determine the acceptability of use of LAI‐PrEP among key populations (KPs) attending Most at Risk Populations Initiatives‐HIV prevention clinic in Kampala, Uganda.

**Methods**: We conducted a cross‐sectional study between November and December 2021 using interviewer‐administered semi‐structured questionnaires to assess acceptability of LAI‐PrEP. We conveniently sampled participants ≥18 years through community and facility HIV prevention activities for KPs. Trained research assistants reached out to current oral PrEP users, disengaged users, and those at risk but who had never initiated PrEP. We determined knowledge on and level of willingness (yes, not sure, no) to use LAI‐PrEP when it becomes available and performed multinomial regression analysis to determine associated factors. Preferences for location of LAI‐administration were also determined.

**Results**: Among 234 participants (56.7%, females) of median age 28 (IQR 25 to 32), 57 (24.4%) were disengaged users, 75 (32.1%) were those at risk but who had never initiated PrEP and 102 (43.7%) were current oral PrEP users. The key populations assessed included female sex workers (49.2%), men who have sex with men (12.0%), people who inject drugs (14.1%), transgender (1.7%), and truck drivers (17.1%). Although 85% of participants had some knowledge about PrEP, 65.8% were willing to receive LAI‐PrEP, 6.8% were not sure while 24.8% were not willing. Preferences for LAI‐PrEP administration (145, 61.9%), home (58, 24.8%), community pharmacy (18, 7.1%), office (3, 1.3%) and others (9, 3.8%). Disengaged users (OR 3.13, CI 1.33 to 7.39) and those at risk but had never initiated oral PrEP (OR 3.99, CI 1.63 to 9.79) were more willing to receive LAI‐PrEP compared to those currently on oral PrEP.

**Conclusion**: There is high willingness by key populations to use LAI‐PrEP including among those who have previously disengaged. Efforts to accelerate the availability of LAI‐PrEP will meet this need.

**References**

1. Shrestha R, Copenhaver M. Exploring the use of pre‐exposure prophylaxis (PrEP) for HIV prevention among high‐risk people who use drugs in treatment. Front Public Health. 2018;6:195.

2. Liu A, Cohen S, Follansbee S, Cohan D, Weber S, Sachdev D, et al. Early experiences implementing pre‐exposure prophylaxis (PrEP) for HIV prevention in San Francisco. PLoS Med. 2014;11:e1001613.

3. The US FDA approved cabotegravir extended‐release ‐ the first long‐acting injectable option for HIV pre‐exposure prophylaxis [Internet]. [cited 2022 Jul 1]. Available from: https://www.who.int/news/item/21‐12‐2021‐fda‐approved‐cabotegravir‐extended‐release.

4. Mobula L, Barnhart M, Malati C, Rakhmanina N, Minior T, Amzel A, et al. Long‐acting, injectable antiretroviral therapy for the management of HIV infection: an update on a potential game‐changer. J AIDS Clin Res. 2015;6:466.

#### P013 Determinants of intention to use pre‐exposure prophylaxis and condom use among female sex workers in Madrid, Spain

L Vazquez Guillamet ^1^, J Valencia^2^, P Ryan^3^, I Cobo^4^, J Lazarus^1^, G Chevance^4^

^1^Viral and Bacterial Infections Program, Barcelona Institute for Global Health (ISGlobal), Barcelona, Spain; ^2^Addictions and Mental Health Office, Harm Reduction Unit, Madrid, Spain; ^3^Internal Medicine, Infanta Leonor University Hospital, Madrid, Spain; ^4^eHealth, Barcelona Institute for Global Health (ISGlobal), Barcelona, Spain

**Background**: Spain recognised female sex workers (FSWs) as a population at high risk of acquiring HIV and granted them subsidised access to pre‐exposure prophylaxis (PrEP) in 2019. Nevertheless, the national PrEP campaign only targets men who have sex with men, with FSWs representing just 0.3% of PrEP users in 2021. This study aims to identify the determinants of intention to use oral PrEP and condom use among FSWs in Madrid.

**Materials and methods**: A cross‐sectional 82‐item survey was delivered in person, along with point‐of‐care HIV testing, to FSWs in Madrid. The survey collected data on demographics, HIV risk, intention to use PrEP, as well as individual, social and structural barriers to PrEP and condom use. Stepwise regression analysis was performed to identify variables associated with condom use and intention to use PrEP.

**Results**: A total of 102 HIV‐negative FSWs were interviewed between January and March 2022. The mean age was 38.7 years (±10). The average number of clients per day was 6.1 (±4.4), and the average daily income was 115.2 euros (±80). Most FSWs were migrants (64.7%); 71.6% worked on the streets; 45% were homeless; 52% used drugs (cocaine and/or heroin), and 25.5% used condoms inconsistently. A small proportion of FSWs (9.8%) knew about PrEP before the study, and 72.5% expressed intention to use PrEP as an oral pill (81.4%), an injection (57.8%), and/or vaginally (22.5%). Intention to use oral PrEP was significantly associated (p < 0.05) with the feeling of being protected against HIV by taking PrEP, and participants’ perception that PrEP use was necessary if condom use was inconsistent. Inconsistent condom use was associated with frequent consumption of drugs, sexual encounters with people who inject drugs, and willingness to take PrEP even if it does not protect 100% against HIV infection.

**Conclusions**: PrEP awareness was low among FSWs in Madrid but the intention to use PrEP was high. HIV prevention campaigns should prioritise FSWs who use drugs and are more likely to engage in condomless sex. Interest in oral PrEP did not correlate with social and structural determinants, but with participants’ feeling of protection against HIV with condoms and PrEP.

#### P014 Are PrEP and other HIV prevention methods used by people experiencing homelessness in London?

E Pool ^1^, P Pierce^2^, J Gibbons^2^, A Story^3^, D Menezes^2^, J Surey^2^, B Sultan^1^

^1^Institute for Global Health, UCL, London, UK; ^2^Find and Treat, London, UK; ^3^UCL, London, UK

**Background**: The prevalence of HIV in people who experience homelessness (PWEH) in London is eight times higher than the general population: 3.2% versus 0.4% [1,2]. PrEP is highly effective as HIV prevention but is underutilised by marginalised groups. The COVID‐19 Homeless Rapid Integrated Screening Protocol (CHRISP) health assessment was undertaken for people temporarily accommodated in hotels in London. Using CHRISP baseline data, we aim to investigate whether PrEP and other HIV prevention methods were used by PWEH.

**Methods**: Data were collected on demographics, housing, health status and BBV risk factors, for this analysis people living with HIV were excluded and descriptive statistics were completed.

**Results**: Between May and November 2020 data were collected at 19 venues across London. One thousand, two hundred and forty‐one HIV‐negative people were identified, 82.3% were cis‐male, 17.1% cis‐female and 0.5% were trans/nonbinary. Median age: 40.9 years, 48.6% were white and 26.7% Black African/Caribbean, and 68.0% were non‐UK born. Baseline housing status was 58.6% rough sleeping, 12.8% hostel, with the remaining insecure, prison, or with family/friends. History of assault was high: 39.6% physical, and 11.4% sexual assault. Regarding HIV acquisition risk, 8.4% were people who inject drugs (PWID) and 5.3% had exchanged sex for money, accommodation or to meet basic needs. For HIV prevention interventions only 2/1241 (0.2%) were using PrEP, both were cis‐males, 22.1% used condoms for recent sex, 46.8% had never previously had HIV test, and of PWID, 55.4% recently shared needles.

**Conclusions**: There is significant unmet need for PrEP and all HIV prevention measure in PWEH. Despite a high prevalence of HIV and risk factors for HIV acquisition, <1% of PWEH were using PrEP. There might be a role for long‐acting injectable PrEP as adhering to daily oral tablets can be difficult in the context of other challenges such as homelessness. There is room for improvement in all aspects of HIV prevention including PrEP, condoms, testing, and needle exchange. More research is needed to identify the barriers and facilitators and to ensure equity of access to all HIV prevention services for PWEH.

**References**

1. Sultan B, Surey J, Gibbons J, Francis M, Ghosh I, Vora N, et al. High prevalence of HIV among people who experience homelessness in London: results of an innovative peer‐centred outreach bloodborne virus testing service initiated at the start of the COVID‐19 pandemic. HIV Med. 2021;22:8‐9.

2. UK Health Security Agency (UKHSA). Country and region HIV data tables. London: UKHSA; 2021.

#### P015 Impact of the COVID‐19 pandemic on sexual behaviour and welfare of HIV preexposure prophylaxis users in Northern France: a prospective quantitative and qualitative monocentric study

A Meybeck, A Kamadjou, A Decock, T Huleux, A Depreux, E Aissi, L Landre, V Baclet, N Viget, M Valette, O Robineau

Infectious Diseases, Centre Hospitalier de Tourcoing, Tourcoing, France

**Background**: Both COVID‐19 pandemic and its control measures can affect the sexual behaviour and the adherence to treatment of people using HIV preexposure prophylaxis (PrEP).

**Materials and methods**: We conducted a single‐centre cross‐sectional study via a self‐administered questionnaire given to all PrEP users coming in scheduled consultation in Tourcoing Hospital from 1 February 2021 to 1 May 2021. Complementarily, 14 participants took part in semi‐structured in‐depth interviews (IDIs).

**Results**: In total, 94 PrEP users completed the questionnaire. All participants were men, with a mean age of 39±11 years. Most participants identified as gay (94%). On‐demand PrEP was the preferred strategy in 59% of cases. During lockdown, 62% of participants continued PrEP, and 33% were tested for sexual transmitted infections (STIs). The average number of sexual intercourses and partners increased after the lifting of lockdown from 7±13 to 13±18 intercourse/month (p < 0.001) and from 3±11 to 12±37 partners/month (p < 0.001). During lockdown, 70% of PrEP users reported never using condom, compared to 44% after it was lifted (p < 0.001). After lockdown release, the proportion of PrEP users who engaged in group sex, sex with alcohol or chemsex increased respectively from 28% to 55% (p < 0.001), 28% to 45% (p < 0.001) and 28% to 38% (p < 0.005). Analysis of IDIs revealed frequent sexual frustration during lockdown. Sex with casual partners was reduced. The release of lockdown was perceived as a sexual liberation. Respondents reported a return to 'normal' life by defying the law. But high frequency of clandestine chemsex parties and the curfew forcing overnight stay increased fears of intimate violence and overdoses.

**Conclusions**: PrEP users reduced their sexual activity during first COVID lockdown in Northern France. Relief of containment measures led to increase in sexual risk‐taking. The reprehensible nature of non‐compliance with social distancing measures may favour medical and social harm of sexual risk‐taking, particularly chemsex.

#### P016 HIV pre‐exposure prophylaxis services, provision, and delivery in the NEAT ID network

G Liegeon^1^, A Duffy^2^, C Brooks^2^, H Honour^2^, J Molina ^1^, on behalf of the PrEP NEAT ID Study Group^3^

^1^Infectious Diseases Department, Assistance Publique ‐ Hôpitaux de Paris, Saint Louis and Lariboisière Hospitals, Paris, France; ^2^Research Organisation Kings Cross, London, UK; ^3^NEAT ID Foundation, The European Treatment Network of HIV, Hepatitis and Global Infectious Diseases, London, UK

**Background**: An increasing number of countries in the European region have integrated HIV pre‐exposure prophylaxis (PrEP) in their HIV prevention programmes. We conducted a survey to evaluate the implementation of PrEP services in European centres belonging to the European treatment network for HIV, hepatitis, and global infectious diseases (NEAT ID).

**Materials and methods**: We designed a survey comprising 23 questions to derive information on the following areas: PrEP use, PrEP services provision, user profiles, structure and delivery of services, barriers to PrEP roll‐out, and PrEP research experience. The questionnaire was sent via a secure electronic tool to 342 centres involved in the NEAT ID network across 15 countries in Europe. The survey started in November 2020 and closed on 31 October 2021.

**Results**: Fifty out of 342 sites from 11 countries provided a response (15% response rate). All sites were in Western Europe, except two sites in Poland and one in Hungary. Out of the responding sites, 45 provided PrEP services for a total of 27 416 PrEP users with 1361 new PrEP initiators each month. These centres supplied PrEP for MSM (100%), PWID (84%), sex workers (84%), women (62%) and TGW (31%). PrEP persistence after 1 year was >90%, 75% to 90% and 40% to 75% in 17, 24 and four centres, respectively. Thirty‐two of 45 (71%) centres reported a strong community‐based organisations commitment at their site, 27/45 (60%) have already been involved in communication campaigns about PrEP, and 15/45 (33%) sites developed PrEP de‐medicalisation strategies led by nurses (11/15), pharmacists (5/15) and key‐population peers (2/15). Low awareness and knowledge about PrEP (47%), unwillingness to disclose sexual identity or at‐risk behaviour (36%), lack of administrative support (29%), and PrEP cost (20%) were the biggest PrEP implementation barriers. Thirty‐two of 45 (71%) sites have already been involved in PrEP research and 43/45 (96%) were eager to participate in future studies on PrEP and STIs.

**Conclusion**: PrEP is mainly implemented in Western European sites. These centres express a high interest in conducting research on PrEP and STIs.

#### P017 Post‐trial oral pre‐exposure prophylaxis (PrEP) access among women who used oral PrEP as HIV prevention standard of care during a large clinical trial: findings from Durban, South Africa

I Beesham ^1^, M Beksinska^1^, C Milford^1^, J Smit^1^, L Mansoor^2^

^1^Department of Obstetrics and Gynaecology, Faculty of Health Sciences, University of the Witwatersrand, MatCH Research Unit (MRU), Durban, South Africa; ^2^Centre for Aids Programme of Research in South Africa (CAPRISA), Durban, South Africa

**Background**: HIV endpoint‐driven clinical trials frequently provide oral pre‐exposure prophylaxis (PrEP) as part of the HIV prevention package offered to participants during the trial; however, among participants desiring to continue using oral PrEP at trial termination, little is known about access and continued use post‐trial exit.

**Materials and methods**: We interviewed 13 women from Durban, South Africa who used oral PrEP as an HIV prevention option during the Evidence for Contraceptive Options and HIV Outcomes (ECHO) Trial, elected to continue PrEP at trial exit, and were referred to off‐site facilities such as clinics, for ongoing PrEP access. One‐time, face‐to‐face, in‐depth interviews were conducted from November to December 2021, to explore post‐trial oral PrEP access and use. All women interviewed had exited the ECHO Trial in 2018. Interviews were audio‐recorded, transcribed and analysed thematically using NVivo.

**Results**: Of the 13 women, about half accessed PrEP post‐trial exit, but majority later discontinued PrEP (Figure 1). Barriers to PrEP continuation post‐trial exit included PrEP facilities having long waiting queues, being located far from participants' homes, having inconvenient operating hours due to participants working, and participants being unable to afford transport costs. Similar barriers were reported among women who did not attempt to access PrEP post‐trial exit. Two women visited their local clinics and requested PrEP but were informed that PrEP was not available at the clinic. Only one woman was still using PrEP at the time of the interview. This woman reported that her local clinic provided PrEP education and created demand, was located close to her home, staff were friendly, and PrEP was easily available. Most women who were not on PrEP reported wanting to use it again, particularly if barriers to access could be removed.

**Conclusions**: In our study, women reported several barriers to accessing oral PrEP post‐trial exit. While strategies to enhance PrEP access such making PrEP more widely available, convenient facility operating hours, and reduction in waiting queues are needed, it is worth noting that oral PrEP access in South Africa has increased from 2018, when women exited the ECHO Trial, till now.

**Figure:**
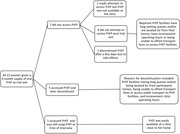
**Abstract P017 – Figure 1**. Post‐trial oral PrEP access and use among women from Durban, South Africa.

#### P018 Characterisation of a population on PrEP and loss to follow‐up

R Sousa ^1^, R Domingos^2^, J Alves^3^, S Peres^3^, A Silva^3^, T Baptista^3^, K Mansinho^3^

^1^Internal Medicine, Hospital Pedro Hispano, Matosinhos, Portugal; ^2^Internal Medicine, Hospital de Faro, Faro, Portugal; ^3^Infectious Diseases and Tropical Medicine, Hospital Egas Moniz, Lisboa, Portugal

**Background**: Oral pre‐exposure prophylaxis (PrEP) is an effective strategy to reduce the incidence of HIV infection in high‐risk individuals [1]. The effectiveness of this prophylaxis is highly dependent on user adherence, meaning that loss to follow‐up (LTFU) is a concern [2]. Key barriers to PrEP adherence are at the individual, community and healthcare structures level.

**Materials and methods**: The authors performed a descriptive and retrospective study including adults attending PrEP outpatient follow‐up in a 4‐year period (April 2018 to April 2022), using a structured form for data extraction. LTFU was defined as the patient being unreachable and/or missing further appointments. Re‐engagement in care was defined as individuals who were ever LTFU and later actively asked for a follow‐up. Findings were summarised regarding adherence and expressed reasons for poor adherence.

**Results**: A total of 508 patients were included, the median of age being 36 years old (minimum age 19; maximum 69 years old). A total of 446 patients (87.80%) started on PrEP (Table 1). At the first appointment, 14 patients had contraindications for PrEP, five of those because of a new HIV diagnosis; and 18 had no criteria for starting it (11 of them were in a monogamous relationship). Thirty patients had formal indication for PrEP but were LTFU before a prescription could be made (Figure 1). Of the patients that started PrEP (N = 446), 176 (39.46%) were no longer on PrEP by April 2022. They were accompanied for a mean of 290 days and median of 176 days; 105 patients (23.54% of all patients started on PrEP) were LTFU for unknown reasons or poor adherence, while 71 patients had clinical or socioeconomic reasons leading to the PrEP suspension. Some of these patients (27) re‐engaged in follow‐up and the main reason for suspending PrEP was professional or social incapacity for attending screenings.

**Conclusions**: In the characterised population there is still a relevant proportion of patients who are LTFU for unknown reasons and poor adherence. The identification of key barriers to PrEP adherence is of major importance so that effective measures can be implemented to prevent it.

**Table:** 

**Abstract P018 – Table 1**. Demographic characterisation.		
	Absolute frequency	Relative frequency
Age at first appointment (years)	(N)	(%)
18 to 20	7	1.38
21 to 30	191	37.60
31 to 40	162	31.89
41 to 50	100	19.69
51 to 60	39	7.68
Biological sex		
Male	496	97.64
Female	12	2.36
Gender identity		
Cis	496	97.64
Trans	15	2.95
Sexual orientation		
MSM	419	82.48
MSM/W	68	13.39
MSW	5	0.98
WSW	1	0.20
WSW/M	4	0.79
WSM	11	2.17
Nationalities		
Portugal	275	54.13
Brazil	147	28.94
Rest of Europe	34	6.69
Rest of America	24	4.72
Africa	21	4.13
Asia	7	1.38

MSM, men who have sex with men; MSM/W, men who have sex with men and women; MSW, men who have sex with women; WSM, women who have sex with men; WSW, women who have sex with women; WSW/M, women who have sex with women and men.

**Figure:**
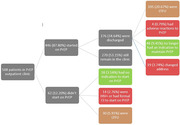
**Abstract P018 – Figure 1**. Schematics of follow‐up up to April 2022. Relative values regarding the total of patients (N = 508).

**References**

1. Sidebottom D, Ekström AM, Strömdahl S. A systematic review of adherence to oral pre‐exposure prophylaxis for HIV – how can we improve uptake and adherence? BMC Infect Dis. 2018;18:581.

2. Mayer KH, Chan PA, Patel RR, Flash CA, Krakower DS. Evolving models and ongoing challenges for HIV preexposure prophylaxis implementation in the United States. J Acquir Immune Defic Syndr. 2018;77:119‐27.

#### P019 Profile and prevention trajectory of recently HIV infected patients in Belgium

C Vanden Bulcke ^1^, J Deblonde^2^, S Callens^1^, C Verhofstede^3^

^1^General Internal Medicine, Ghent University Hospital, Ghent, Belgium; ^2^Epidemiology of Infectious Diseases, Sciensano, Brussels, Belgium; ^3^Department of Diagnostic Sciences, Ghent University, Ghent, Belgium

**Background**: Despite wide availability of prevention and treatment services, including continued roll‐out of pre‐exposure prophylaxis (PrEP), only slight declines in the number of new HIV diagnoses has been observed in recent years. Hence, there is a perceived need to improve case detection and early diagnosis in order to contain the spread of HIV further. The aim of the current study was to improve insight on the profile of patients recently infected with HIV and on their prevention trajectory.

**Materials and methods**: Between May 2018 and February 2021, we selected patients diagnosed in Belgium less than 4 months of the presumed infection and used a triangulation of data including phylogenetic cluster analysis and data on HIV testing, sexually transmitted infections (STIs), PrEP use, sexual behaviour, partner notification and substance abuse, collected using a questionnaire.

**Results**: A total of 202 individuals with a recent HIV infection were registered, of which 121 were invited to participate in the study. The questionnaire was completed by 77, mainly MSM (71.4%) (hetero women, 15.6%; hetero men, 13%). The mean age was 37.8 years. Ten percent (all MSM, mean age 33.8 years; 75% foreigner) used PrEP in the past of which all except one reported the use of substances during sex and experienced one or more STIs, syphilis being the most frequent. Half of them did not notify their previous sex partner(s). Overall, high substance use during sex (68%) and frequent HIV testing prior to the diagnosis (64.1% tested at least once a year) was reported. One third (34%) had five or more casual sex partners prior to diagnosis with whom they frequently had condomless sex (77%). No significant differences in behavioural characteristics were found between individuals who were part of a local transmission cluster (53.4%) and individuals that were not part of a cluster (46.6%).

**Conclusions**: The results of this study revealed that the majority of individuals diagnosed in Belgium early after HIV infection show characteristics corresponding to the known high‐at‐risk population and are aware of the risk. This information may help to expose lost opportunities for prevention and improve the use of future prevention measures.

#### P020 Abstract withdrawn

#### P021 High acceptance of same‐night PrEP start in a safer space for trans and male sex workers: the Night Café

B Hampel ^1^, L Guelfi^2^, J Real^3^, B Zahno^4^, J Fehr^1^

^1^Epidemiology, Biostatistics and Prevention Institute, University of Zurich, Zurich, Switzerland; ^2^Checkpoint Zurich, Zurich, Switzerland; ^3^Flora Dora, Social Department City of Zurich, Zurich, Switzerland; ^4^Isla Victoria, Solidaria Zurich, Zurich, Switzerland

**Background**: Trans and male sex workers have one of the highest risks of getting infected with HIV. However, the threshold to access PrEP is higher than in other populations.

**Methods**: Between January and July 2022 we offered monthly PrEP consultations and same‐day PrEP prescription to trans and male sex workers within the SwissPrEPared [1] programme/cohort study at a social meeting point in the red light district of the city of Zurich: the Night Café. Sex workers who were interested in PrEP could come to the café without an appointment. Translators for five different languages were available as well as social workers who could help with non‐medical concerns. All participants who wanted to start PrEP on the same day received a fourth‐generation rapid HIV test. PrEP was prescribed, if the test was negative and if there was no sign for a kidney disease in the medical history. HIV PCR, kidney and liver tests, serology for syphilis, hepatitis B and C and PCR for chlamydia and gonorrhoea were performed and the results were discussed with the participants in the following week via phone. Data on sexual risk behaviour were obtained through the SwissPrEPared questionnaire, which the participants filled out prior the consultation.

**Results**: During the six events 23 participants were enrolled in the programme. Fourteen were cis‐male of who all identified as either homo‐ or bisexual. Eight participants were trans‐women and one was a cis‐woman. Ten participants were already using PrEP, but were doing so without medical supervision. Two out of 23 participants reported to never have had an HIV test taken before and two reported that their last HIV test was longer than 12 months ago. Thirteen out of 15 participants (87%) have taken up their follow‐up appointment at a sexual health clinic.

**Conclusion**: Acceptance of PrEP consultation and follow‐up rate was high in this population, when offered in this setting. Same‐day PrEP start was safe in this preliminary pilot study. The project will be continued to be followed by the SwissPrEPared study to generate more information on how to improve access to PrEP for male and trans sex workers.

**Reference**

1. Hovaguimian F, Martin E, Reinacher M, Rasi M, Schmidt AJ, Bernasconi E, et al. Participation, retention and uptake in a multicentre pre‐exposure prophylaxis cohort using online, smartphone‐compatible data collection. HIV Med. 2022;23:146‐58.

#### P022 HCV infection in an outpatient PrEP clinic in a Portuguese tertiary hospital

M Miguel, J Lourinho, H Pires, T Nunes, D Lages, A Gomes, L Duque, J Botas, N Marques

Infectious Diseases, Garcia de Orta Hospital, Lisbon, Portugal

**Background**: The use of intravenous drugs is the primary way of transmission of the hepatitis C virus (HCV). Sexual transmission of HCV, particularly in patients infected with HIV, is also commonly seen. Nevertheless, HIV‐negative men who have sex with men (MSM), especially those under pre‐exposure prophylaxis (PrEP), are a recognised at‐risk population. In this work, we wanted to evaluate and determine the incidence of acute HCV infection and the risk factors associated with its transmission in the setting of an ambulatory PrEP clinic.

**Materials and methods**: Retrospective analysis of the electronic registries of the patients seen in the PrEP clinic in a tertiary hospital between January 2019 and June 2022.

**Results**: Between January 2019 and June 2022, there were 436 patients assessed in the PrEP consultation. Four (0.9%) were diagnosed with an acute HCV infection, with a mean age of 30. The four patients were reviewed every 3 months with a medical assessment and pathology, including HCV antibodies and alanine aminotransferase (ALT). All had genotype 1a and ALT elevation at the time of diagnosis. The majority (n = 3) were under a daily regimen of PrEP and the mean average time between the beginning of PrEP and the diagnosis of HCV infection was 326 days (min 28, max 717). One patient was diagnosed before the introduction of PrEP. Epidemiologically, they were MSM and had at least one other sexually transmitted disease, including *Neisseria gonorrhoeae, Chlamydia trachomatis* or syphilis. None of the patients were intravenous drug users. At the time of writing, two patients are undergoing treatment for HCV infection, and one has completed the treatment with a sustained virological response at 24 weeks post‐therapy. The other one is waiting to start the treatment.

**Conclusions**: The number of acute HCV infections is increasing in HIV‐negative patients, especially in MSM under PrEP. This underlines the importance of regular surveillance to allow an early diagnosis and prompt treatment, particularly in this population with risk factors for the transmission.

#### P023 Post‐exposure prophylaxis and pre‐exposure prophylaxis in gay, bisexual, and other men who have sex with men who practise sexualised drug use

D Íncera^1^, J Garrido^2^, M Gámez^1^, I Zaro^3^, L García ^2^, A García^2^, L Ibarguchi^2^

^1^Psicología, Universidad Autónoma, Madrid, Spain; ^2^Psicología, Apoyo Positivo, Madrid, Spain; ^3^Psicología, Imagina Más, Madrid, Spain

Sexualised drug use (SDU) has become a public health priority due to its associated risks. Little is known about primary and secondary prevention measures for STIs associated with SDU. The objective of this study was to study the relationship between HIV and other STI prevention measures and SDU in gay, bisexual, and other men who have sex with men (GBMSM). The sample of this study consisted of 493 GBMSM aged 18 to 78 years (mean age = 32.58, SD = 11.05). The majority (n = 415, 84.2%) self‐identified as gay and, to a lesser extent, as bisexual (n = 61, 12.4%), pansexual (romantic or sexual attraction to other people regardless of gender) (n = 13, 2.6%), or other sexual orientation (n = 4, <1%). Mean age differed significantly between participants who practiced SDU (mean age = 38.21, SD = 11.57) and those who did not (mean age = 29.13, SD = 9.44). HIV‐positive and ‐negative GBMSM aged 18 years and older were invited to participate through social networks (Instagram and Twitter), gay dating apps, and information distributed through various LGBTIQ + associations and NGOs. Men in the SDU group used more post‐exposure prophylaxis (PEP) (8.6% and 3.9%, respectively; χ2 = 34 833, p < 0.001) and pre‐exposure prophylaxis (PrEP) (19.3% and 2.6%, respectively; χ2 = 104 926, p < 0.001) in the previous 18 months. Regarding STI preventions with couple, GBMSM who had practiced SDU were significantly more likely to not use any prevention measure (38.0% vs 25.5%; χ2 = 9058, p = 0.003) or to be taking PrEP for HIV prevention with a couple (22.5% vs 3.6%; χ2 = 45 053, p < 0.001). These findings indicate the importance of to expand and facilitate access to HIV prevention strategies, such as PrEP, among those most at risk of HIV seroconversion. These strategies may represent an opportunity to acquire or reinforce the responsibility for sexual health.

#### P024 Pre‐exposure prophylaxis (PrEP) uptake and adherence experiences of gay and bisexual men who engage in chemsex: a qualitative study

S Maxwell ^1^, M Gafos^2^, M Shahmanesh^3^

^1^Department of Health, Glasgow Caledonian University, Glasgow, UK; ^2^Department of Global Health and Development, London School of Hygiene and Tropical Medicine, London, UK; ^3^Institute for Global Health, University College London, London, UK

**Background**: Pre‐exposure prophylaxis (PrEP) is the use of HIV antiretroviral medications to reduce the risk of HIV acquisition. PrEP is highly effective when used during periods of potential HIV exposure. Gay and bisexual men (GBM) who engage in unprotected chemsex (without condoms/PrEP) are at high risk of acquiring HIV. Substance use has been shown to detrimentally impact on the effective use of HIV treatment among GBM living with HIV. This study aims to qualitatively explore PrEP uptake and adherence among GBM who engage in chemsex in the United Kingdom.

**Materials and methods**: Nineteen semi‐structured in‐depth telephone interviews were conducted with self‐identifying HIV‐negative GBM who reported recently engaging in chemsex and currently using or had recently used PrEP. We explored the ways in which chemsex influenced GBM's motivation to use, access to and effective use of PrEP. Interviews were audio recorded, transcribed, and coded using thematic analysis.

**Results**: Most of the men identified as gay, were of white ethnicity and had a median age of 41. Eighteen men were using PrEP at the time of the interview and most used daily dosing. The perception of being at high risk of HIV acquisition was a key factor influencing PrEP initiation and after initiation, continued to influence high levels of adherence which was reported by the majority of participants. The few individuals who reported sub‐optimal adherence, explained that psychosocial stressors or periods of impaired mental health led to more frequent or intense chemsex sessions, which in turn contributed to occasional non‐adherence. Most participants used a variety of strategies to help them adhere, which included restricting the amount or intensity of chemsex they engaged in, strategic placement of PrEP and external triggers to remind them to take PrEP.

**Conclusions**: In this study, the majority of GBM who engaged in chemsex initiated PrEP in recognition of their potential risk of HIV acquisition and reported high levels of PrEP adherence. They used multiple strategies to support effective PrEP access and adherence. These findings support a growing body of evidence that PrEP is a viable prevention tool for GBM who engage in chemsex, and that chemsex does not negatively impact PrEP adherence.

#### P025 Abstract withdrawn

### Treatment Strategies: New Treatments and Targets

#### P026 Week 52 subgroup efficacy analyses of long‐acting subcutaneous lenacapavir in phase II/III in heavily treatment‐experienced people with multidrug‐resistant HIV (CAPELLA study)

A Castagna ^1^, J Blanco^2^, C Hung^3^, M Rassool^4^, M Ramgopal^5^, W Sanchez^6^, C Cretico^7^, D Hagins^8^, D Wheeler^9^, H Wang^10^, I Henne^11^, H Dvory‐Sobol^12^, M Rhee^12^, J Baeten^12^, O Onyema^13^

^1^San Raffaele Scientific Institute, Vita‐Salute University, Milan, Italy; ^2^Infectious Diseases Department, Hospital Clinic de Barcelona, Barcelona, Spain; ^3^Division of Infectious Diseases, National Taiwan University Hospital, Taipei, Taiwan; ^4^Department of Internal Medicine, Clinical HIV Research Unit, Helen Joseph Hospital, University of the Witwatersrand, Auckland Park, Johannesburg, South Africa; ^5^Infectious Diseases, Midway Immunology & Research Center, LLC, White City, FL, USA; ^6^Floridian Clinical Research, Miami Lakes, FL, USA; ^7^Infectious Diseases, Howard Brown Health Center, Chicago, IL, USA; ^8^Chatham County Health Department, Coastal CARE Clinics, Savannah, GA, USA; ^9^Infectious Diseases, Clinical Alliance for Research and Education Infectious Diseases, Annandale, VA, USA; ^10^Biostat, Gilead Sciences, Inc, Foster City, CA, USA; ^11^Virology PM, Gilead Sciences, Inc, Foster City, CA, USA; ^12^Virology Clinical Development, Gilead Sciences, Inc, Foster City, CA, USA; ^13^Yale AIDS Program Clinical Trials Unit, Yale University School of Medicine, New Haven, CT, USA

**Background**: Lenacapavir (LEN), a potent first‐in‐class inhibitor of HIV‐1 capsid function, is in development as a long‐acting agent for treatment and prevention of HIV‐1. The ongoing phase II/III CAPELLA study in heavily treatment‐experienced (HTE) people with HIV (PWHIV) viremic on their current regimen with multidrug resistance demonstrated that LEN in combination with an optimized background regimen (OBR) led to high rates of virologic suppression at week 52 (83%, 30/36) and CD4 increase (83 cells/μL).

**Materials and methods**: The study included randomized and non‐randomized cohorts. In the randomized cohort, participants were randomized (2:1) to add oral LEN or placebo to their failing regimen. At day 15 (D15), those on oral LEN received subcutaneous (SC) LEN (Q6M); those on placebo started the oral lead‐in, followed by SC Q6M. In the randomized cohort, participants discontinued a failing regimen and initiated an investigator‐selected OBR at D15. All participants were required to have at most two fully active antiretroviral agents remaining from the four main classes (NRTI, NNRTI, PI, INSTI), determined by prior resistance testing or testing at screening. In the non‐randomized cohort, participants initiated OBR concurrent with LEN (oral lead‐in then SC). We conducted subgroup analyses by baseline HIV‐1 RNA, CD4, and OBR of the week 52 efficacy (assessed using FDA Snapshot algorithm) in the randomized cohort; analysis is ongoing for the non‐randomized cohort.

**Results**: Seventy‐two participants enrolled (36 participants in each cohort). Resistance to NRTI, NNRTI, PI and INSTI classes was common: 99%, 97%, 81% and 69% and 17% (12/72) did not have any fully active agents in the OBR. Rates of virologic suppression were comparable among participants who had high HIV‐1 RNA, low CD4, INSTI resistance, suboptimal OBR, and regardless of use of DTG, and DRV in the OBR (Table 1).

**Table:** 

**Abstract P026 – Table 1**. HIV‐1 RNA <50 copies/mL at week 52 (Snapshot algorithm) by subgroup.	
Subgroups	Randomized cohort (n = 36)
Overall	83% (30/36)
Baseline CD4 (cells/μL) <200	78% (21/27)
Baseline CD4 (cells/μL) ≥200	100% (9/9)
Baseline HIV‐1 RNA (copies/mL) ≤100 000	86% (25/29)
Baseline HIV‐1 RNA (copies/mL) >100 000	71% (5/7)
With INSTI resistance	81% (22/27)
Without INSTI resistance	88% (7/8)
0 fully active agents in OBR	67% (4/6)
1 fully active agent in OBR	79% (11/14)
≥2 fully active agents in OBR	94% (15/16)
With dolutegravir	83% (15/18)
Without dolutegravir	83% (15/18)
With darunavir	86% (18/21)
Without darunavir	80% (12/15)
With ibalizumab	83% (10/12)
Without ibalizumab	83% (20/24)

**Conclusions**: In this heavily treatment‐experienced population with limited treatment options due to MDR, LEN in combination with OBR led to high rates of viral suppression in individuals with high HIV‐1 RNA, low CD4 count, and limited fully active agents in the OBR.

#### P027 Common adverse events in clinical studies of people using lenacapavir for HIV treatment

A Antinori ^1^, F Castelli^2^, S Ronot‐Bregigeon^3^, Y Yazdanpanah^4^, R Safran^5^, D Berger^6^, P Cook^7^, G Sinclair^8^, H Wang^9^, G Saunders^10^, T Farrow^10^, H Dvory‐Sobol^11^, M Rhee^11^, J Baeten^11^, S Gupta^12^

^1^Unità Operativa Complessa: Immunodeficienze Virali, Instituto di Ricovero e Cura a Carattere Scientifico, Rome, Italy; ^2^Department of Infectious and Tropical Diseases, University of Brescia and Azienda Socio Sanitaria Territoriale Spedali Civili di Brescia, Brescia, Italy; ^3^Aix Marseille Université, Hôpital Sainte Marguerite, Marseille, France; ^4^Infectious Diseases Department, Hôpital Bichat‐Claude Bernard, Paris, France; ^5^Rockwood Internal Medicine & HIV, MultiCare Rockwood Clinics, Spokane, WA, USA; ^6^Northstar Medical Center, University of Illinois, Chicago, IL, USA; ^7^Division of Infectious Diseases, East Carolina University, Greenville, NC, USA; ^8^Oak Cliff Health Center, Prism Health North Texas, Dallas, TX, USA; ^9^Biostat, Gilead Sciences, Inc, Foster City, CA, USA; ^10^Patient Safety, Gilead Sciences, Inc, Foster City, CA, USA; ^11^Virology Clinical Development, Gilead Sciences, Inc, Foster City, CA, USA; ^12^School of Medicine, Indiana University, Indianapolis, IN, USA

**Background**: Lenacapavir (LEN), a potent first‐in‐class inhibitor of HIV‐1 capsid function, is in development as a long‐acting agent for treatment and prevention of HIV‐1. We previously characterized LEN‐related injection site reactions (ISRs) [1]. Herein, we characterize adverse events (AEs) other than ISRs in participants who received at least one dose of oral or subcutaneous (SC) LEN in clinical studies in heavily treatment‐experienced (HTE) (CAPELLA) and in treatment‐naïve (TN) (CALIBRATE) people with HIV (PWHIV).

**Materials and methods**: In both studies, LEN was administered for oral loading (600 mg on day 1 and 2 and 300 mg on day 8), then subcutaneous (SC) LEN (927 mg Q6M) starting at day 15. CALIBRATE included an additional group of oral daily LEN (50 mg) with emtricitabine/tenofovir alafenamide (F/TAF). For all, LEN was used in combination with other antiretrovirals.

**Results**: In CAPELLA, 72 participants enrolled; in CALIBRATE, 157 enrolled. The median duration of follow‐up was 54 and 66 weeks, respectively. There were no SAEs related to study drug. Most non‐ISR AEs were grade 1 or 2 and resolved during ongoing treatment with LEN. No participant discontinued LEN due to a non‐ISR AE. The common AEs in the SC groups were nausea, diarrhea, and headache: 13%, 13%, and 8% in CAPELLA, and 14%, 7%, and 13% in CALIBRATE, respectively. Investigators considered AEs of nausea, diarrhea, and headache related to LEN in 3% to 5% of participants. In both studies, the onset and duration of each AE showed no consistent temporal pattern (Table 1). The AEs in CAPELLA occurred concurrent with co‐administration of complex optimized background regimens. In CALIBRATE, gastrointestinal AEs were similar in the SC LEN groups versus oral LEN (nausea 14% vs 12%; diarrhea 7% vs 10%; vomiting 4% vs 8%).

**Table:** 

**Abstract P027 – Table 1**. Onset and duration of common AEs in the SC groups in CAPELLA and CALIBRATE studies.				
	CAPELLA: SC LEN with OBR for HTE (N = 72)	CAPELLA: SC LEN with OBR for HTE (N = 72)	CALIBRATE: SC LEN with other agents for naive (N = 105)	CALIBRATE: SC LEN with other agents for naive (N = 105)
	Median onset, day (range)	Median duration, days (range)	Median onset, day (range)	Median duration, days (range)
Nausea	60 (3 to 166)	95 (1 to 531)	64 (1 to 176)	11 (1 to 153)
Diarrhea	67 (3 to 166)	100 (3 to 531)	81 (2 to 107)	5 (2 to 84)
Headache	110 (46 to 171)	9 (2 to 31)	69 (2 to 163)	15 (1 to 92)

**Conclusions: **Among a range of PWHIV using oral and/or SC LEN, LEN was well tolerated with no non‐ISR AEs related to LEN leading to discontinuation.

**Reference**

1. Kumar P, Gupta S, Segal‐Maurer S, Ogbuagu O, McDonald C, Brinson C, et al. Injection‐site reaction experience in clinical studies of people using lenacapavir for HIV treatment. AIDS 2022: 24th International AIDS Conference; 2022 Jul 29‐Aug 2; Montréal, Québec, Canada. Poster EPB184.

#### P028 VICDOR: EffectiVeness of swItChing to DOR‐based antiretroviral therapy (ART) under real‐world conditions in Germany

J Rockstroh ^1^, C Wyen^2^, M Sabranski^3^, H Knechten^4^, S Esser^5^, N Qurishi^6^, C Jonsson‐Oldenbuettel^7^, J Bogner^8^, S Schellberg^9^, I Kolobova^10^, J Pelz^11^, Y Whiteside^10^

^1^Private Practice, University Hospital Bonn, Bonn, Germany; ^2^Private Practice, Praxis am Ebertplatz, Cologne, Germany; ^3^Private Practice, Infektionsmedizinisches Centrum Hamburg (ICH) Study Center, Hamburg, Germany; ^4^Laboratory Dr. Knechten, Medical Center for HIV and Hepatitis, Aachen, Germany; ^5^Private Practice, University Hospital Essen, Essen, Germany; ^6^Private Practice, Gemeinschaftspraxis Gotenring, Cologne, Germany; ^7^Private Practice, Karlsplatz, Munich, Germany; ^8^Medizinische Klinik und Poliklinik IV, University Hospital Munich, Munich, Germany; ^9^Private Practice, Novopraxis Berlin GbR, Berlin, Germany; ^10^Center for Observational and Real‐World Evidence, Merck & Co., Inc., Rahway, NJ, USA; ^11^Medical Affairs Infectious Disease, Merck Sharp & Dohme GmbH, Munich, Germany

**Background**: Doravirine (DOR), a non‐nucleoside reverse transcriptase inhibitor (NNRTI), used in combination with other antiretrovirals for the treatment of HIV‐1 infection, is effective and well tolerated [1‐3]. This study characterizes the use, effectiveness, and impact on both body weight and lipids of DOR‐based ART in a virologically suppressed switch population.

**Materials and methods**: VICDOR is an ongoing, multicenter, retrospective chart review study in Germany that includes data from regular clinical visits for up to 15 months after switching to DOR‐based ART. Adult people living with HIV‐1 (PLWHIV) who were virologically suppressed at the time of switch to DOR‐based ART between January 2019 and June 2021 were included. We collected demographic, clinical, and laboratory data.

**Results**: Of the 97 PLWHIV included in this interim analysis, the median age was 48 years (IQR 40 to 55), 85.6% were male, 29.9% were obese (BMI ≥ 30), and 81.4% had at least one comorbidity. 91.8% were switched to doravirine/lamivudine/tenofovir disoproxil fumarate (DOR/3TC/TDF), and 8.2% switched to another DOR‐based regimen. The main reason for switch was to improve tolerability regarding weight gain (44.3%). Prior ART anchor classes included integrase strand transfer inhibitors (INSTI, 62.9%), NNRTIs (23.7%) and protease inhibitors (PI, 13.4%); 63.9% had been on a TAF‐containing regimen. No cases of virologic failure were observed. Of the 61 individuals with HIV‐1 RNA results at 12 months (±1 month), 100% remained virologically suppressed. Mean increase of CD4 T‐cell counts was 13.48 cells/μL (SD 194.66) from switch to month 12. Of those PLWHIV who switched to DOR/3TC/TDF (n = 89), body weight decreased a mean of ‐0.89 kg (SD 4.71), and levels of low‐density lipoprotein‐cholesterol (LDL‐C) decreased a median of ‐5.50 mg/dL (IQR ‐23.00 to 11.00) between switch and month 12. Among patients who switched to DOR/3TC/TDF specifically to improve tolerability regarding weight gain, body weight decreased by a mean of ‐2.54 kg (SD 4.72) from switch to month 12.

**Conclusion**: The preliminary data suggest that in the real world, DOR‐based ART was effective in maintaining viral suppression and had no evidence of weight increase in a switch population of PLWHIV with a high prevalence of comorbidities.

**References**

1. Molina JM, Squires K, Sax PE, Cahn P, Lombaard J, DeJesus E, et al. Doravirine versus ritonavir‐boosted darunavir in antiretroviral‐naive adults with HIV‐1 (DRIVE‐FORWARD): 96‐week results of a randomised, double‐blind, non‐inferiority, phase 3 trial. Lancet HIV. 2020;7:e16‐e26.

2. Orkin C, Squires KE, Molina JM, Sax PE, Wong WW, Sussmann O, et al. Doravirine/lamivudine/tenofovir disoproxil fumarate is non‐inferior to efavirenz/emtricitabine/tenofovir disoproxil fumarate in treatment‐naive adults with human immunodeficiency virus‐1 infection: week 48 results of the DRIVE‐AHEAD trial. Clin Infect Dis. 2019;68:535‐44.

3. Johnson M, Kumar P, Molina JM, Rizzardini G, Cahn P, Bickel M, et al. Switching to doravirine/lamivudine/tenofovir disoproxil fumarate (DOR/3TC/TDF) maintains HIV‐1 virologic suppression through 48 weeks: results of the DRIVE‐SHIFT trial. J Acquir Immune Defic Syndr. 2019;81:463‐72.

#### P029 The implementation of every‐2‐months cabotegravir and rilpivirine long‐acting injections from the perspective of healthcare providers in the German CARLOS cohort, 6‐month outcomes

J Scherzer ^1^, C Jonsson‐Oldenbuettel^2^, S Schneeweiß^3^, C Wyen^4^, J Borch^5^, K Ummard‐Berger^6^, N Postel^7^, E Rodriguez^8^, C Gutner^9^, K Dymek^1^, B Westermayer^10^, K Bernhardt^1^

^1^Medical Affairs, ViiV Healthcare, Munich, Germany; ^2^Clinical Care, Medizinisches Vesorgungszentrum München am Goetheplatz, Munich, Germany; ^3^Clinical Care, Praxis Hohenstaufenring, Cologne, Germany; ^4^Clinical Care, Praxis Ebertplatz, Cologne, Germany; ^5^Clinical Care, Praxis Goldstein, Berlin, Germany; ^6^Clinical Care, UBN / Praxis, Berlin, Germany; ^7^Clinical Care, Prinzmed, Munich, Germany; ^8^Clinical Care, Praxisgemeinschaft ViRo Schillerkiez, Berlin, Germany; ^9^Medical Affairs, ViiV Healthcare, Research Triangle Park, NC, USA; ^10^Medical Affairs, GlaxoSmithKline GmbH, Munich, Germany

**Background**: Long‐acting (LA) cabotegravir (CAB) plus rilpivirine (RPV) is the first LA regimen recommended in treatment guidelines for maintenance of viral suppression in people living with HIV (PLWHIV) [1]. The prospective CARLOS study is a non‐interventional, 3‐year multi‐centre cohort study in PLWHIV being switched from daily oral therapy to CAB+RPV LA in accordance with the label in routine clinical care in Germany. Here we describe concerns, barriers and facilitators of the implementation of LA therapy from a healthcare provider (HCP) perspective.

**Materials and methods**: Interim analysis includes quantitative data from HCPs (physicians, nurses, office staff) at 22 sites across Germany. Implementation questionnaires were administered at baseline (BL) and month 6 (M6).

**Results**: At data cut, BL questionnaire had been completed by 43 HCPs (across 18 sites), M6 questionnaire by 38 HCPs (across 18 sites). Previous experience administering CAB+RPV LA from clinical trials was reported by 65% of HCPs at BL (n = 28/43). At M6, overall feeling about implementing CAB+RPV LA was positive in 92% of HCPs (n = 35/38) (extremely 21%/very 42%/somewhat positive 29%) with no difference between those with or without prior trial experience (90% [19/21] vs 94% [n = 16/17]). Average patient time spent in clinic/practice was estimated to be ≤40 minutes by 50% of HCPs (n = 19/38) and ≤20 minutes by 37% of HCPs (n = 14/38). Sixty‐six percent of HCPs (n = 25/38) considered time spent in clinic as extremely or very acceptable for the patients. Table 1 shows the top three HCP techniques to minimise pain during injection and HCP advice for reducing soreness after the injection. To compare implementation concerns at BL and M6, only sites responding to both the BL and M6 questionnaires were included (14 sites). The top‐rated implementation concerns related to patients’ ability to adhere to injections and possible risk of resistance development. Most concerns identified at BL showed a decrease in mean scores (Figure 1).

**Table:** 

**Abstract P029 – Table 1**. Top three injection techniques to minimise injection pain and muscle soreness.	
Month 6: top three techniques to minimise pain during injection	
− Slow speed of pushing intramuscular injections	74% (n = 28/38)
− Having patient relax muscle prior to injection	71% (n = 27/38)
− Assuring medication being at room temperature	66% (n = 25/38)
**Month 6: top three advice provided for reducing soreness after the injection**	
− Taking over‐the‐counter pain relievers	50% (n = 19/38)
− Using cold compresses	42% (n = 16/38)
− Avoiding prolonged sitting	39% (n = 15/38)

**Figure:**
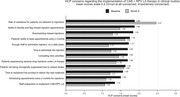
**Abstract P029 – Figure 1**. HCP concerns regarding the implementation of CAB+RPV LA therapy in clinical routine (only sites having completed both a BL and M6 questionnaire were included; BL: n = 32 HCPs, M6: n = 29 HCPs).

**Conclusion**: The CARLOS cohort provides first insights in HCP perspectives on injectable LA treatment for PLWHIV in a real‐world setting in Germany. Although some concerns remained during the first 6 months, the overall feeling about implementing CAB+RPV LA was positive in the majority of HCPs.

**Reference**

1. European AIDS Clinical Society. Guidelines for management of people living with HIV in Europe 2021 [cited 2022 Jun 20]. Available at: https://www.eacsociety.org/media/final2021eacsguidelinesv11.0_oct2021.pdf.

#### P030 Fostemsavir and QT prolongation: clinical applications for co‐administration with other agents

S Patel^1^, V Vega^1^, A Dyson ^1^, R Guduru^2^, G Morcos^3^, M Calderón^1^, A Mannebach^2^, B Gilliam^1^, A Clark^4^, A Tenorio^1^, K Moore^5^

^1^Medical Affairs, ViiV Healthcare, Durham, NC, USA; ^2^Medical Affairs, GSK, Durham, NC, USA; ^3^Clinical Development, Bios Clinical Research, Palm Springs, CA, USA; ^4^Medical Affairs, ViiV Healthcare, Brentford, UK; ^5^Clinical Pharmacology, ViiV Healthcare, Durham, NC, USA

**Background**: Fostemsavir (FTR) is an oral prodrug of temsavir (TMR), a first‐in‐class attachment inhibitor approved for use with other antiretrovirals (ARVs) for the treatment of HIV‐1 in heavily treatment‐experienced (HTE) adults with multidrug‐resistance. A thorough QT study in healthy volunteers demonstrated a supratherapeutic dose of FTR 2400 mg BID was associated with a mean (upper 90% CI) QTc prolongation of 11.2 msec (13.3 msec). Since QTc prolongation is primarily a concentration‐dependent effect, and FTR is partially metabolized by the CYP P450 (3A4), we examined potential drug‐drug interactions (DDI) between FTR, pharmacokinetic enhancers, and other agents associated with QTc prolongation or Torsade de Pointes (TdP) risk.

**Materials and methods**: Guideline recommendations, data from FTR clinical studies including the phase III BRIGHTE study, and the Liverpool HIV Drug Interaction Database were used to assess QTc prolongation risk with FTR and other co‐administered agents.

**Results**: A plasma TMR of 7500 ng/mL intersects with the clinically important QTc prolongation threshold of 10 msec (upper bound of the 90% CI), which is 4‐fold greater than the mean peak TMR plasma concentration in the BRIGHTE study (1770 ng/mL). Through week 96 of the BRIGHTE study with FTR 600 mg BID, seven participants discontinued for protocol‐defined QTc prolongation of >500 msec; all were non‐serious events.  Although discontinued from the study, six participants remained on FTR through the named patient program (NPP 207214); none experienced a symptomatic cardiovascular event or documented ventricular tachyarrhythmia. A systematic Liverpool DDI database evaluation showed no significant/reported high potential for QTc prolongation when FTR is co‐administered with other drugs. Nevertheless, the combinations of greatest concern and not recommended when FTR + boosted protease inhibitors (bPI) or FTR + RPV are used include several antiarrhythmics due to potential additive QTc risk.

**Conclusions**: FTR does not require dose adjustment when combined with other ARVs or agents with a known TdP risk. Clinically significant interactions may occur with agents with known TdP risk and the combination of FTR + bPI or FTR + RPV. Evaluation of FTR‐containing ARV regimen, pre‐existing cardiac disease, and age must be considered when combining with agents with known TdP risk.

#### P031 Patterns of doravirine use in a US cohort

M Sension ^1^, L Brunet^2^, R Hsu^3^, K Mounzer^4^, J Fusco^2^, Y Whiteside^5^, J Arduino^6^, I Kolobova^5^, G Fusco^2^

^1^Medicine, Community Aids Network (CAN) Community Health, Sarasota, FL, USA; ^2^Sciences/Epidemiology, Epividian, Inc., Durham, NC, USA; ^3^Medicine, AIDS Healthcare Foundation, New York, NY, USA; ^4^Jonathan Lax Treatment Center, Philadelphia FIGHT Community Health Centers, Philadelphia, PA, USA; ^5^Outcomes Research, Merck & Co., Inc., Rahway, NJ, USA; ^6^Epidemiology, Merck & Co., Inc., Rahway, NJ, USA

**Background**: Doravirine (DOR) is the newest non‐nucleoside reverse transcriptase inhibitor (NNRTI) to be developed. In the US, it was approved by the FDA on 30 August 2018 as a single agent or co‐formulated with lamivudine (3TC) and tenofovir disoproxil fumarate (TDF). We sought to characterize DOR users and patterns of antiretroviral therapy (ART) use before and with DOR in the US.

**Materials and methods**: All people with HIV (PWHIV) aged ≥18 years initiating DOR between 30 August 2018 and 30 November 2021 in the OPERA^®^ cohort were included and followed from DOR initiation to the first date of DOR discontinuation (i.e. switch or >45 days without ART), loss to follow‐up, death, or study end (12 May 2022). Characteristics of ART‐experienced DOR users and their patterns of treatment were stratified by viral load (VL) at DOR initiation. A Sankey diagram illustrated pathways from prior ART regimens to DOR‐containing regimens.

**Table:** 

**Abstract P031 – Table 1**. Characteristics and treatment patterns of ART‐experienced doravirine users with a baseline viral load, N = 790.			
	ART‐experienced, VL <50 (N = 435)	ART‐experienced, VL ≥50 to <200 (N = 122)	ART‐experienced, VL ≥200 (N = 233)
Median age (IQR)	54 (43 to 61)	54 (45 to 59)	49 (39 to 56)
Male sex, n (%)	329 (76)	99 (81)	163 (70)
Black race, n (%)	145 (33)	45 (37)	145 (62)
History of AIDS‐defining events, n (%)	168 (39)	49 (40)	117 (50)
Comorbidities, n (%)	392 (90)	115 (94)	208 (89)
Median VACS index^a^ (IQR)	22 (12 to 34)	18 (12 to 28)	35 (19 to 52)
Median calendar year of ART initiation (IQR)	2014 (2010 to 2017)	2016 (2012 to 2018)	2015 (2012 to 2018)
NNRTI included in prior regimen	60 (15)	≤5^b^	7 (3)
DOR/3TC/TDF single tablet formulation, n (%)	112 (26)	11 (9)	38 (16)
Anchor agent			
DOR only	139 (32)	12 (10)	39 (17)
DOR + other anchor agent(s)^c^	296 (68)	110 (90)	194 (83)

3TC, lamivudine; DOR, doravirine; IQR, interquartile range; TDF, tenofovir disoproxil fumarate; VACS, Veterans Aging Cohort Study; VL, viral load.

^a^Veterans Aging Cohort Study (VACS) index: 5‐year mortality index, scored by summing pre‐assigned points for age, CD4 cell count, HIV‐1 RNA, hemoglobin, platelets, aspartate and alanine transaminase, creatinine, and viral hepatitis C infection. A higher score is associated with a higher risk of 5‐year all‐cause mortality;

^b^Health Insurance Portability and Accountability Act (HIPAA) requires the masking of cells with one to five individuals;

^c^integrase strand transfer inhibitor (INSTI), protease inhibitor (PI), non‐nucleoside reverse transcriptase inhibitor (NNRTI), attachment inhibitor, fusion inhibitor.

**Figure:**
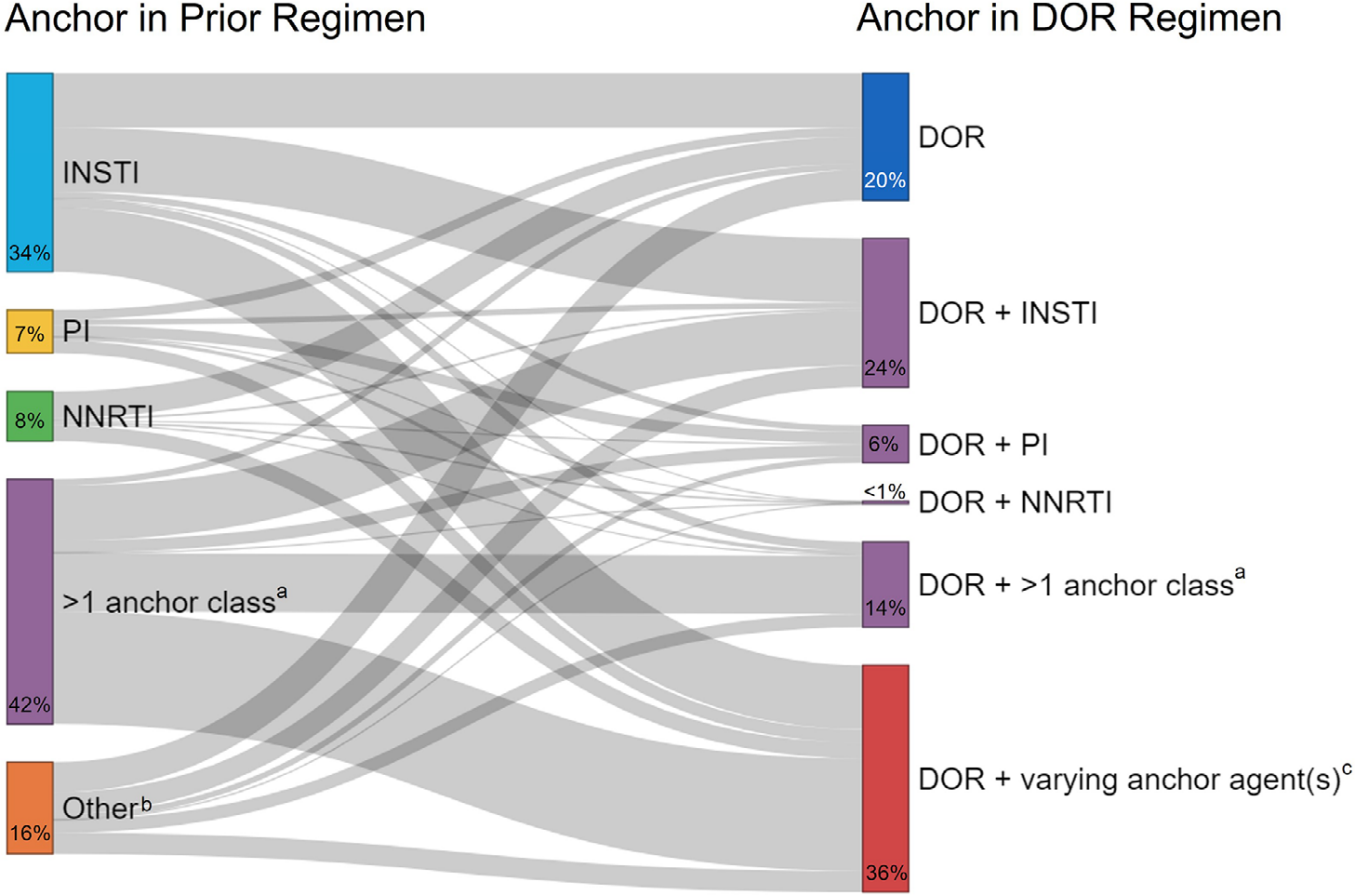
**Abstract P031 – Figure 1**. ART regimen pathways from prior regimen to doravirine‐containing regimen among PWHIV with ART experience or unknown prior experience, N = 870. ^a^ More than one anchor agent class (i.e. INSTI, PI, NNRTI, attachment inhibitor, fusion inhibitor); ^b^ attachment inhibitor, fusion inhibitor, unknown prior experience or therapeutic gap; ^c^ other anchor agent(s) changed over time while maintaining DOR. DOR, doravirine; INSTI, integrase strand transfer inhibitor; PI, protease inhibitor.

**Results**: Between 30 August 2018 and 30 November 2021, 893 PWHIV initiated DOR: 23 ART‐naïve, 56 unknown prior experience, 814 ART‐experienced. Overall, 90% of ART‐experienced PWHIV taking DOR had comorbidities, with over half experiencing endocrine disorders, hypertension and/or mental health conditions; 42% had a history of AIDS‐defining event. The Veterans Aging Cohort Study (VACS) index (i.e. 5‐year mortality risk) tended to be high (median ranging from 18 to 35 across groups). The median age at DOR prescription ranged from 49 to 54 years (Table 1). DOR was most often prescribed in combination with other anchor agent(s) (VL <50: 66%, VL ≥50 to <200: 83%, VL ≥200: 82%). A minority received the DOR/3TC/TDF single tablet formulation (VL <50: 26%, VL ≥50 to <200: 9%, VL ≥200: 16%; Table 1). The last regimen prior to DOR use most often included either an integrase strand transfer inhibitor (31%) or >1 anchor agent class (38%; Figure 1). Once DOR was initiated, 36% experienced changes in other anchor agents while maintaining DOR use (Figure 1).

**Conclusions**: In the US, DOR was most often used by ART‐experienced PWHIV with comorbidities, in combination with other anchor agent(s). Following DOR initiation, regimen readjustment was common, while DOR continued. These findings highlight the complexity of ART regimens in a population who may have comorbidities impacting treatment needs and choices.

#### P032 Development of conceptual models to understand patient experiences with and attributes of adherence to HIV oral antiretroviral therapy and considerations in switching to long‐acting oral antiretroviral therapy

J Bailey^1^, P Javidnia^2^, J Mao^3^, A Borsa^2^, E Hawryluk^2^, S Gubernick^2^, A de la Motte^2^, E Fonseca ^4^, S Karantzoulis^2^, M Reaney^5^, T Saretsky^6^

^1^IQVIA, New York, NY, USA; ^2^Patient Centered Solutions, IQVIA, New York, NY, USA; ^3^Outcomes Research, Merck & Co., Inc., Rahway, NJ, USA; ^4^Center for Observational and Real‐world Evidence, Merck & Co., Inc., Rahway, NJ, USA; ^5^Patient Centered Solutions, IQVIA, Reading, UK; ^6^Biostatistics and Research Decision Sciences, Merck & Co., Inc., Rahway, NJ, USA

**Background**: As new long‐acting oral antiretroviral therapies (LA‐OARTs) are in development for patients living with HIV (PLWHIV), it is important to understand attributes with potential effects on adherence. Following FDA patient‐reported outcome (PRO) guidance, two conceptual models (CMs) were developed to capture PLWHIVs' experiences of adhering to oral antiretroviral therapy (OART) ('adherence CM') and perspectives on a hypothetical switch to LA‐OART ('switch CM').

**Materials and methods**: A targeted literature review (TLR), in PubMed, EMBASE and PsycINFO, of qualitative research exploring PLWHIV experiences with daily OART focused on attributes defined as facilitators and/or barriers to adherence informed a preliminary adherence CM. This preliminary adherence CM was tested and finalized in 1:1 concept elicitation interviews with seven HIV‐treating clinicians and 28 PLWHIV. We also elicited perspectives on switching to LA‐OART, exploring which attributes from the adherence CM may be considered relevant in switch decisions, to inform the switch CM. A review of existing relevant PRO instruments was conducted; attributes from that review were mapped to both CMs to assess coverage.

**Results**: The TLR included 18 papers, identifying nine facilitators (e.g. small pill size) and 16 barriers (e.g. side effects) which formed an adherence preliminary CM. An additional 13 attributes were identified from clinician interviews (e.g. stigma associated with HIV status) and three from PLWHIV interviews (e.g. case management). The final adherence CM contained 16 facilitators, 22 barriers and three attributes that could be either. The final switch CM contained 24 attributes: 21 from the adherence CM and three novel ones (e.g. reduced preoccupation with regimen‐taking). No single or collection of existing PROs, among the 56 reviewed, capture all attributes in the adherence CM and the switch CM.

**Conclusions**: The CMs provide a structured framework to understand PLWHIV experiences with and facilitators and barriers to OART adherence and the factors influencing a switch to LA‐OART. The CMs may inform the development of new PRO instruments to address gaps in existing PROs and for use in appropriate clinical research for PLWHIV.

#### P033 Pediatric dolutegravir introduction for children living with HIV (CLWHIV) in Zimbabwe: lessons from early adopter sites

C Giyava ^1^, N Kawaza^1^, C Chimhundu^2^, A Mushavi^3^, P Andifasi^3^, A Mangwiro^1^

^1^HIV Access Program, Clinton Health Access Initiative, Harare, Zimbabwe; ^2^HIV Access Program, Clinton Health Access Initiative, Boston, MA, USA; ^3^Prevention of Mother to Child Transmission, Ministry of Health and Child Care, Harare, Zimbabwe

**Background**: Zimbabwe has 49 640 CLWHIV on ART, of which 43.19% take suboptimal legacy formulations with adverse clinical outcomes. The introduction of pediatric dolutegravir 10 mg (pDTG) for CLWHIV weighing 3 to 20 kg above 4 weeks of age affords access to more optimal regimens with the potential to improve adherence and clinical outcomes. Supported by Clinton Heath Access Initiative (CHAI), the Ministry of Health and Child Care (MoHCC) planned a phased rollout of pDTG, starting with 13 high‐volume sites targeting 1766 CLWHIV to generate experience and lessons with pDTG by December 2021 before national scale‐up.

**Methods**: A transition plan was compiled through iterative stakeholder consultations to guide implementation, covering pDTG eligibility, dosing, administration, pharmacovigilance, and vigilant uptake monitoring strategies referred to as 'enhanced monitoring'. Quantification of pDTG for early adopter sites relied on 2018 weight‐band assumptions (WBA), as the assessments necessary to update the WBA were delayed due to COVID‐19 disruptions. Implementing partners (IPs) were mobilised to support procurement, clinical mentorship, and uptake monitoring. Facility‐level orientations immediately followed product arrival in June 2021, reaching 246 Healthcare Workers including nurses, doctors, pharmacists, and counsellors.

**Results**: Despite rapid product distribution and capacity‐building efforts, early monitoring visits signalled a slow pDTG transition, missing original scale‐up timelines by 3 months. Bottlenecks identified included unavailability of recent viral load (VL) results, children not presenting to facilities for clinically guided transitions, HCW attrition, and COVID‐19 lockdown restrictions affecting healthcare access. In response, updated VL guidance, mentorship approaches and materials for HCWs and caregivers were disseminated in collaboration with clinical IPs to strengthen messaging and follow‐up. Community outreaches encouraged the presentation of CLWHIV for clinical review and transitioning. Once travel restrictions eased late 2021, a follow‐up WBA showed 20.2% of the CLWHIV being <20 kg, relative to 31.1% (2018) used for introduction planning. These WBA changes contributed to underconsumption, which was addressed through re‐forecasting in February 2022 and product redistribution. These concerted efforts yielded increased CLWHIV transitions from 15% (n = 257) in September 2021 to 52% (n = 920) of the scale‐up target by March 2022.

**Conclusions**: Active stakeholder collaboration and enhanced monitoring has been critical to pDTG introduction and rapid mitigation of unexpected transition bottlenecks, thus providing lessons at a smaller scale ahead of national rollout.

#### P034 Rescue therapy with ibalizumab in HIV multi‐drug resistances (MDR) patient

S Martini ^1^, N Cuomo^2^, M Pisaturo^1^, N Coppola^1^

^1^Infectious Diseases Unit, University of Campania Luigi Vanvitelli, Naples, Italy; ^2^Infectious Diseases Unit, Aorn dei Colli, Naples, Italy

**Background**: Advances in antiretroviral therapy have optimised efficacy, tolerability and adherence, but some patients remain more difficult to manage. These are patients who have changed many regimens developing multi‐drug resistances (MDR). The guidelines in this case suggest setting up a therapy that contains new‐generation drugs associated with residual ones that are still effective. One of this new‐generation drugs is ibalizumab [1]. It is a monoclonal antibody that blocks the CD4 cellular receptor thus preventing the interaction and contact with the viral gp120.

**Materials and methods**: The patient has performed several resistance tests over the years on plasma and HIV‐DNA showing a range of multiple mutations to all classes of antiretrovirals. On the basis of these tests, a rescue therapy was set up with the association of entecavir + tenofovir + dolutegravir + ibalizumab. At the time of the introduction of this therapy, the patient had HIV‐RNA of 37 800 copies/mL and CD4+ of 147 cells/μL. He started therapy with ibalizumab, first as monotherapy with a loading dose of 2000 mg, then after 7 days a dose of 800 mg associated with three drugs chosen on the resistance test.

**Results**: Viro‐immunological data were progressively evaluated and rapid efficacy of salvage therapy was noted. After 7 days, ibalizumab alone had already reduced HIV viral load by 2 log. After 3 weeks it was reduced by 1 more log and after 4 weeks, viral suppression was achieved. CD4 progressively improved from 147 to 254 cells/μL (Figure 1). The patient experienced no adverse events other than a rise in blood pressure after the first infusion of the loading dose of ibalizumab.

**Figure:**
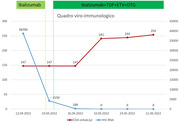
**Abstract P034 – Figure 1**. Viro‐immunological evolution under rescue therapy.

**Conclusions**: In conclusion, our case report shows that ibalizumab has been extremely effective in lowering the viral load, both alone and subsequently in combination with three other antiretrovirals. Virological suppression was accompanied by good immunological recovery. The overall tolerability profile was good, despite the onset of hypertension after the first infusion of the drug. Our data corroborates the efficacy of ibalizumab in rescue therapies of MDRs.

**Reference**

1. Blair HA. Ibalizumab: a review in multidrug‐resistant HIV‐1 infection. Drugs. 2020;80:189‐96.

### Treatment Strategies ‐ Target Populations: Adolescents and Children

#### P035 Treatment‐emergent integrase inhibitor resistance among pediatric and adolescent HIV‐1 populations: a systematic review

C Henegar^1^, C Brothers^2^, C Vavro^3^, L Tan^4^, M McKenna^5^, V Vannappagari ^1^, A Buchanan^6^

^1^Epidemiology and Real World Evidence, ViiV Healthcare, Durham, NC, USA; ^2^Clinical Development Sciences, Pediatrics, ViiV Healthcare, Durham, NC, USA; ^3^Clinical Virology, ViiV Healthcare, Durham, NC, USA; ^4^Clinical Development, ViiV Healthcare, Brentford, UK; ^5^Safety Evaluation and Risk Management Head's Group, GSK, Uxbridge, UK; ^6^ViiV Physicians, ViiV Healthcare, Durham, NC, USA

**Background**: Integrase strand transfer inhibitors (INSTIs) are globally preferred first‐line antiretroviral regimens for treatment in infants, children and adolescents living with HIV. This systematic review summarizes the frequency of first‐ and later‐line treatment‐emergent INSTI drug resistance mutations (DRMs) in these populations.

**Materials and methods**: MEDLINE, Embase, PubMed, and international conference proceedings from January 2010 to April 2022 were searched for clinical trials (CTs) and non‐interventional studies (NISs) reporting resistance data following INSTI exposure. Included publications reported on outcomes [virologic failure (VF) and treatment‐emergent DRMs] among ART‐naïve and ART‐experienced people living with HIV <18 years old. Studies with young adults (18 to 25 years) were included if individuals <18 years were enrolled.

**Results**: Sixteen publications were included (eight CTs and eight NISs with follow‐up on 1544 individuals, Table 1). A primary reason for publication exclusion was lack of integrase resistance testing prior to widespread testing availability. RAL‐based regimens (210 exposures in one CT and three NISs) most frequently reported VF (n = 94; 45% of total RAL exposures) and incident DRMs (n = 25; 12% of total RAL exposures). For EVG, there were four VFs (4%) and no documented emergent EVG‐associated DRMs across 94 exposures in three CTs. Among 943 DTG‐based regimens in three CTs and three NISs, 173 (18%) VFs were reported with 13 (1%) treatment‐emergent DTG DRMs. A single CT reported 100 BIC exposures, with two VFs (2%) and no emergent BIC DRMs. Two additional NISs aggregated outcomes for individuals taking DTG (n = 107), RAL (n = 32), and EVG (n = 58), documenting 80 VFs (41%) and three (2%) emergent mutations with potential for resistance to EVG and RAL.

**Table:** 

**Abstract P035 – Table 1. ** INSTI resistance developed after failure on an INSTI‐based regimen.									
Study	Country	Population	Age range (years)	INSTI used	Total on INSTI (n)	Treatment failure on INSTI (n)	Treatment failure on INSTI [% (95% CI)]	INSTI resistance following failure (n)	INSTI resistance following failure [% (95% CI)]
Clinical trials									
IMPAACT P1066 [1]	Multinational	ART‐experienced, viremic	4 weeks to 18	RAL	122	60	49 (40 to 58)	19	16 (10 to 23)
GS‐US‐292‐0106 [2]	Multinational	ART‐experienced, suppressed	6 to 11	EVG	23	0	0 (0 to 15)	0	0 (0 to 15)
GS‐US‐292‐0106 [3]	Multinational	ART‐naive	12 to 18	EVG	50	3	6 (1 to 17)	0	0 (0 to 7)
GS‐US‐236‐0112 [4]	Multinational	ART‐naive	12 to 17	EVG	21	2	10 (1 to 30)	0	0 (0 to 16)
IMPAACT P1093 [5]	Multinational	ART‐naive and ART‐experienced	4 weeks to 17	DTG	142	36	25 (18 to 33)	8	6 (2 to 11)
ODYSSEY [6]	Multinational	ART‐naive and ART‐experienced, viremic	4 weeks to 17	DTG	350	47	13 (10 to 17)	4	1 (0 to 3)
SMILE PENTA‐17 [7]	Multinational	ART‐experienced, suppressed	6 to 18	DTG^a^	158	8	5 (2 to 10)	0	0 (0 to 2)
GS‐US‐380‐1474 [8]	Multinational	ART‐experienced, suppressed	6 to 17	BIC	100	2	2 (0 to 7)	0	0 (0 to 4)
Non‐interventional studies									
Abo et al, 2019 [9]	United Kingdom	ART‐naive and ART‐experienced	0 to 17	DTG/ RAL/ EVG	29/ 21/ 6	8^b^	14 (6 to 26)	1^b^	2 (0 to 10)
Levy et al, 2020 [10]	United States	ART‐experienced	0 to 24	DTG	78	72^c^	51 (43 to 60)	0	0 (0 to 5)
				RAL	11			1	9 (0 to 41)
				EVG	52			1	2 (0 to 10)
Briz et al, 2012 [11]	Spain	ART‐experienced, viremic	6 to 18	RAL	19	2^d^	11 (1 to 33)	0	0 (0 to 18)
Patten et al, 2020 [12]	Multinational	ART‐experienced, viremic	0 to 17	RAL	62	24^e^	39 (27 to 52)	4^f^	6 (2 to 16)
Steegen et al, 2019 [13]	South Africa	ART‐experienced	0 to 17	RAL	7^g^	7	100 (59 to 100)	2	29 (4 to 71)
Briand et al, 2017 [14]	France	ART‐naive and ART‐experienced	12 to 17	DTG	50	17	34 (21 to 49)	0	0 (0 to 7)
Frange et al, 2019 [15]	France	ART‐naive and ART‐experienced	5 to 25	DTG	109	22	20 (13 to 29)	0	0 (0 to 3)
Frange et al, 2021 [16]	France	ART‐naive and ART‐experienced	6 to 18	DTG	134	43	32 (24 to 41)	1	1 (0 to 4)

^a^One hundred and fifty‐three took DTG+DRV/r, five took EVG+DRV/r;

^b^aggregated results for all INSTI‐based regimens reported;

^c^aggregated results for all INSTI‐based regimens reported for virologic outcomes; virologic failure defined as: 55/70 individuals not suppressed at baseline never achieved suppression or had viral rebound after suppression; 17/35 suppressed at baseline did not maintain suppression through follow‐up;

^d^virologic failure defined as: 2/19 never achieved viral load <400 copies/mL during follow‐up (non‐responders);

^e^virologic failure defined as: 16/62 never achieved viral load <400 copies/mL during follow‐up and 8/62 experienced virologic rebound ≥1000 copies/mL after achieving suppression;

^f^four discontinuations were attributed to virologic failure, immunologic failure, or resistance ‐ no additional details provided;

^g^study looked at samples from individuals currently failing INSTI‐based regimens; there were seven samples from individuals <18 years old.

**Conclusions**: VF can be more common in pediatric and adolescent populations due to adherence challenges, underscoring the importance of regimens with high barriers to resistance. In this review, regimens containing INSTIs, particularly second‐generation INSTIs, were associated with low rates of emergent drug resistance following VF. Across included studies, rates of VF and DRMs varied by prior treatment experience, follow‐up duration, and outcome definitions. Despite growing real‐world use of INSTIs in pediatrics, there were few publications looking at incident DRMs on INSTI‐based ART.

**References**

1. Nachman S, Alvero C, Teppler H, Homony B, Rodgers AJ, Graham BL, et al. Safety and efficacy at 240 weeks of different raltegravir formulations in children with HIV‐1: a phase 1/2 open label, non‐randomised, multicentre trial. Lancet HIV. 2018;5:e715‐22.

2. Natukunda N, Gaur AH, Kosalaraksa P, Batra J, Rakhmanina N, Porter D, et al. Safety, efficacy, and pharmacokinetics of single‐tablet elvitegravir, cobicistat, emtricitabine, and tenofovir alafenamide in virologically suppressed, HIV‐infected children: a single‐arm, open‐label trial. Lancet Child Adolesc Health. 2017;1:27‐34.

3. Gaur AH, Kizito H, Prasitsueubsai W, Rakhmanina N, Rassool M, Chakraborty R, et al. Safety, efficacy, and pharmacokinetics of a single‐tablet regimen containing elvitegravir, cobicistat, emtricitabine, and tenofovir alafenamide in treatment‐naive, HIV‐infected adolescents: a single‐arm, open‐label trial. Lancet HIV. 2016;3:e561‐8.

4. Porter DP, Bennett SR, Quirk E, Miller MD, White KL. Lack of emergent resistance in HIV‐1‐infected adolescents on elvitegravir‐based STRs. Conference on Retroviruses and Opportunistic Infections; 2015 Feb 23‐26; Seattle, WA, USA. Poster 952.

5. Vavro C, Ruel T, Wiznia A, Montañez N, Nangle K, Horton H, et al. Emergence of resistance in HIV‐1 integrase with dolutegravir treatment in a pediatric population from the IMPAACT P1093 study. Antimicrob Agents Chemother. 2022;66:e01645‐21.

6. Turkova A, White E, Mujuru HA, Kekitiinwa AR, Kityo CM, Violari A, et al. Dolutegravir as first‐ or second‐line treatment for HIV‐1 infection in children. N Engl J Med. 2021;385:2531‐43.

7. Compagnucci A, Chan M, Saïdi Y, Cressey T, Bamford A, Riault Y, et al. Once‐daily integrase inhibitor (INSTI) with boosted darunavir is non‐inferior to standard of care in virologically suppressed children, Week 48 results of the SMILE PENTA‐17 TRIAL. 11th IAS Conference on HIV Science; 2021 Jul 18‐21; Virtual / Berlin, Germany. Abstract 1079.

8. Gaur AH, Cotton MF, Rodriguez CA, McGrath EJ, Helström E, Liberty A, et al. Fixed‐dose combination bictegravir, emtricitabine, and tenofovir alafenamide in adolescents and children with HIV: week 48 results of a single‐arm, open‐label, multicentre, phase 2/3 trial. Lancet Child Adolesc Health. 2021;5:642‐51.

9. Abo Y‐N, Refsum E, Mackie N, Lyall H, Tudor‐Williams G, Foster C. Paediatric integrase inhibitor use in a real‐life setting: a single‐centre cohort experience 2009‐2018. Clin Drug Invest. 2019;39:585‐90.

10. Levy ME, Griffith C, Ellenberger N, Monroe AK, Castel AD, Rakhmanina N, et al. Outcomes of integrase inhibitor‐based antiretroviral therapy in a clinical cohort of treatment‐experienced children, adolescents and young adults with HIV infection. Pediatr Infect Dis J. 2020;39:421‐8.

11. Briz V, León‐Leal JA, Palladino C, Moreno‐Perez D, de Ory SJ, De José MI, et al. Potent and sustained antiviral response of raltegravir‐based highly active antiretroviral therapy in HIV type 1‐infected children and adolescents. Pediatr Infect Dis J. 2012;31:273‐7.

12. Patten G, Puthanakit T, McGowan CC, Wools‐Kaloustian K, Hazra R, Pinto JA, et al. Raltegravir use and outcomes among children and adolescents living with HIV in the IeDEA global consortium. J Int AIDS Soc. 2020;23:e25580.

13. Steegen K, van Zyl G, Letsoalo E, Claassen M, Hans L, Carmona S. Resistance in patients failing integrase strand transfer inhibitors: a call to replace raltegravir with dolutegravir in third‐line treatment in South Africa. Open Forum Infect Dis. 2019;6:ofz377.

14. Briand C, Dollfus C, Caseris M, Kantor E, Avettand‐Fenoel V, Descamps D, et al. Dolutegravir‐based cART in vertically HIV‐1–infected adolescents, real‐world setting. Conference on Retroviruses and Opportunistic Infections; 2017 Feb 13‐16; Seattle, WA, USA. Abstract 812.

15. Frange P, Avettand‐Fenoel V, Veber F, Blanche S. Similar efficacy and safety of dolutegravir between age groups of HIV‐1‐infected paediatric and young adult patients aged 5 years and older. HIV Med. 2019;20:561‐6.

16. Frange P, Blanche S, Veber F, Avettand‐Fenoel V. Dolutegravir in the long term in children and adolescents: frequent virological failure but rare acquired genotypic resistance. HIV Med. 2021;22:958‐64.

#### P036 Pediatric dolutegravir is highly preferred by patients/caregivers in Nigeria and Uganda at 1 month after initiation

J Campbell^1^, B Ngwatu ^2^, and the TORPEDO study teams in Nigeria^3^ and Uganda^4^

^1^Analytics and Implementation Research, Clinton Health Access Initiative, Santa Cruz, CA, USA; ^2^Clinical Sciences Team, Clinton Health Access Initiative, Kampala, Uganda; ^3^HIV/AIDS, Nigeria Ministry of Health, Abuja, Nigeria; ^4^HIV/AIDS, Uganda Ministry of Health, Kampala, Uganda

**Background**: Following the successful rollout of dolutegravir 50 mg (DTG), a new generic pediatric formulation of DTG 10 mg (pDTG) dispersible became available in 2021. The strawberry‐flavored once‐daily pDTG dispersible tablet is expected to make administration easier and thus improve adherence and health outcomes for children weighing 3 to <20 kg. Our study assessed pDTG acceptability among patients/caregivers in two early adopter countries in sub‐Saharan Africa.

**Materials and methods**: This is a mixed methods prospective cohort study of patients initiated on pDTG in six facilities in Uganda and seven in Nigeria between October 2021 and March 2022. Patient acceptability and experiences were assessed through one‐on‐one interviews with either patients or caregivers at the 1‐month visit post pDTG initiation. Treatment‐experienced patients were asked about side effects and regimen preferences compared to their previous regimen.

**Results**: There were 403 interviews conducted between November 2021 and April 2022, 93% were treatment‐experienced (most switching from a lopinavir/ritonavir tablet formulation) and 49% male, the average age was 5 years and BMI for age is 40%. At 1 month after initiation, 98% were satisfied or very satisfied with pDTG; 53% of the children swallowed the pill and 44% dispersed in water. Ninety‐nine percent of treatment‐experienced patients preferred pDTG over previous regimen and stated that it is easier to take without missing a dose; 97% said pDTG has improved taste, 96% said it is easier to administer, 81% said it is easier to store and 78% stated side effects were better. The most common side effects reported were increased appetite (23%) and increase in activity (7%); 94% believe the children gained weight appropriately. In treatment‐experienced children, the side effects that improved since pDTG initiation were increased appetite (47%), nausea (36%), trouble sleeping (36%) and diarrhea (33%).

**Conclusions**: There is high self‐reported acceptability to pDTG and preference over previous drug options due to better taste and easier administration and reduced nausea, which are anticipated to help improve health outcomes for children taking pDTG. This study will further evaluate the acceptability and health outcomes at 6‐months follow‐up to ascertain longer‐term effects of pDTG.

#### P037 Abstract withdrawn

### Treatment Strategies ‐ Target Populations: Ageing Population

#### P038 Switch to bictegravir/emtricitabine/tenofovir alafenamide (B/F/TAF) in people living with HIV aged 65 years or older: W24 results of the BICOLDER study ‐ IMEA 057

C Allavena ^1^, V Joly^2^, L Assoumou^3^, V Isernia^4^, F Ajana^5^, D Neau^6^, D Descamps^7^, C Charpentier^7^, A Benalycherif^8^, E Metivier^3^, G Peytavin^9^, B Phung^4^, R Landman^10^

^1^Infectious and Tropical Diseases, Bichat Hospital and IMEA Fondation, Paris, France; ^2^Infectious and Tropical Diseases, Assistance Publique des Hôpitaux de Paris, Hospital Bichat Claude Bernard, Paris, France; ^3^Institut National de la Santé Et de la Recherche Médicale, Sorbonne Université, Paris, France; ^4^Infectious and Tropical Diseases, Assistance Publique des Hôpitaux de Paris Hospital Bichat Claude Bernard, Paris, France; ^5^Infectious and Tropical Diseases, Gustave Dron Hospital, Tourcoing, France; ^6^Infectious and Tropical Diseases, Pellegrin Hospital, Bordeaux, France; ^7^Virology Laboratory, Assistance Publique des Hôpitaux de Paris, Hospital Bichat Claude Bernard, Paris, France; ^8^Clinical Research, Institut de Médecine et d'Épidémiologie Appliquée et Tropicale Fondation Léon M'BA, Paris, France; ^9^Pharmacology‐Toxicology, Assistance Publique des Hôpitaux de Paris Hospital Bichat Claude Bernard, Paris, France; ^10^Infectious and Tropical Diseases, Bichat Hospital and Institut de Médecine et d'Épidémiologie Appliquée et Tropicale Fondation Léon M'Ba, Paris, France

**Background**: Comorbidities and polymedication are frequent in ageing people living with HIV (PLWHIV) and are associated with higher rates of adverse events and pharmacological interactions [1]. We aimed to assess in an elderly PLWHIV population the efficacy, safety and feasibility of B/F/TAF, a potent antiretroviral therapy with limited drug‐drug interactions in maintenance strategy.

**Materials and methods**: The BICOLDER study (NCT04222283) is a multicentric, prospective, single‐arm study conducted in virologically controlled PLWHIV aged 65 years or older (PLWHIV 65+) who switched from a booster antiretroviral containing regimen, ritonavir or cobicistat, to B/F/TAF. The primary outcome was the proportion of participants maintaining pVL <50 copies/mL at week 24 (W24) using ITT FDA Snapshot algorithm.

**Results**: From August 2020 to July 2021, 27 participants were enrolled in eight French clinical centres (three PLWHIV never received the study drug and were excluded from ITT analysis). Patients’ characteristics are presented in Table 1. At screening, 75% of the participants were receiving elvitegravir/cobicistat, and 25% a boosted PI‐containing regimen. At W24, 91.7% (95% CI 73.0 to 99.0) of participants maintained pVL <50 copies/mL. One participant discontinued study treatment due to virological failure at W12 (pVL 807 copies/mL) possibly related to adherence difficulties consecutive to occurrence of adverse events. On the same sample, neither BIC nor FTC and TFV concentrations in plasma 3.5 hours after the last drug intake were detected. After returning to the initial treatment, the plasma HIV‐RNA became undetectable. Drug‐related adverse events (AEs) were reported in six participants (25%), including two grade 3 events in one participant that led to discontinuation of B/F/TAF (mood disorders and nightmare) at W4. No serious AE was reported. There were no significant changes from baseline in lipids, renal parameters, comorbidities, frailty (Charlson and Fried scores), and cardiovascular risk DAD score, except for weight, which increased by 1.75 kg (95% CI 0.7 to 2.8) and BMI by 0.60 kg/m^2^ (95% CI 0.23 to 0.94) at W24.

**Table:** 

**Abstract P038 – Table 1**. Baseline characteristics of the 24 participants.		
Parameter	Median or number	IQR or %
Age (years)	68.5	67 to 73
Male	19	79.2%
BMI (kg/m^2^)	25.3	24.2 to 26.6
Baseline CD4 (cells/mm^3^)	576	479 to 785
Time of known HIV infection (years)	26	19 to 32.5
Time of antiretroviral treatment (years)	20.5	17 to 24
CDC stage C	10	41.8%
Most frequent comorbidities	−	−
− High blood pressure	14	58.3%
− Dyslipidaemia	8	33.3%
− Chronic renal disease (clearance <60 mL/min/1.73 m^2^)	9	37.5%
− Diabetes	4	18.2%
Number of comedications	6	4 to 8.5
DAD score [1]	6.4	4.4 to 16.8
Charlson score [2]	0	0 to 2
Frailty/pre‐frailty/robustness (Fried Frailty Phenotype)	2/12/10	8%/50%/42%

**Conclusions**: These results suggest that B/F/TAF is safe, effective in this elderly HIV population with a long history of HIV infection, multiple comorbidities and comedication.

**References**

1. Friis‐Møller N, Weber R, Reiss P, Thiébaut R, Kirk O, d'Arminio Monforte A, et al. Cardiovascular disease risk factors in HIV patients ‐ association with antiretroviral therapy. Results from the DAD study. AIDS. 2003;17:1179‐93.

2. Quan H, Li B, Couris CM, Fushimi K, Graham P, Hider P, et al. Updating and validating the Charlson comorbidity index and score for risk adjustment in hospital discharge abstracts using data from 6 countries. Am J Epidemiol. 2011;173:676‐82.

#### P039 PROximity: digitisation in the active listening of the HIV patient; mental health as a care priority

A Díaz de Santiago^1^, S de la Fuente Moral^1^, M Corrales Rodríguez^2^, J Martín Giner^3^, C Folguera Olías^4^, B Menchén Viso^4^, A Planes Roy ^5^, R Roldán Blay^5^

^1^Internal Medicine, Hospital Universitario Puerta de Hierro Majadahonda, Madrid, Spain; ^2^Nursery, Hospital Universitario Puerta de Hierro Majadahonda, Madrid, Spain; ^3^IT Systems, Hospital Universitario Puerta de Hierro Majadahonda, Madrid, Spain; ^4^Hospital Pharmacy, Hospital Universitario Puerta de Hierro Majadahonda, Madrid, Spain; ^5^Project Management, Hopes On Health SL, Valencia, Spain

**Background**: As life expectancy of HIV patients increases, neuropsychiatric comorbidities and metabolic problems impact the quality of life of this ageing population. A specific clinical management model is needed, based on health outcomes, clinical and functional status, treatment monitoring and an assessment of the impact of the pathology and its determinants from the patient's perspective [1]. The electronic health record (EHR) based on free text and non‐standardised data hinders evidence generation and debate among professionals to import best practices. Too many HIV‐specific patient‐reported outcomes (PROs) are complex to manage in daily clinic. PROximity incorporates the patient's voice in the care process to improve health outcomes through a new PRO, the Clinical Screening Tool (CST) based on three questions in each domain (anxiety, depression, stigma, fatigue…).

**Materials and methods**: Integration of the HOPES platform in hospitals selected by the Madrid Regional Health Service for: patient contribution in their own care process, through the CST, allowing them to offer their perspective with validated methodology. Interoperability between hospital systems (EMR, drug distribution and laboratory programme, HOPES platform for CST collection) coordinated with the Madrid Health Service from the National Health Service, for a holistic view of people living with HIV. Supporting through alerts for improved decision making and to react early to changes in patient's situation.

**Results**: Multidisciplinary work based on care digitalisation and IT integration, towards the benefit of the HIV patient and the health system. Full characterisation of the hospital cohort. Complete interoperability between HOPES platform and the different hospital providers.

**Conclusions**: PROximity increases the knowledge of HIV patients’ unmet needs through PROs. Solid evidence will be generated from a collaborative, comprehensive and multidisciplinary approach, to detect patient needs and adjust the care pathway to improve HIV patients’ quality of life and wellbeing. Digital integration positions the patients at the centre of the care allowing them to participate. System needs to change and include patients in the process through new technologies.

**Reference**

1. Olry de Labry Lima A, García Mochón L, Bermúdez Tamayo C. Identificación de indicadores de resultado en salud en atención primaria. Una revisión de revisiones sistemáticas. Rev Calid Asist. 2017;32:278‐88.

### Treatment Strategies ‐ Target Populations: Women

#### P040 Adverse birth outcomes and risk of MTCT for dolutegravir versus efavirenz in five randomised trials of 1074 pregnant women

S Sokhela^1^, F Venter^1^, B Bosch^1^, G Akpomiemie^1^, A Tembo^1^, T Pepperrell^2^, B Simmons^3^, L Mulenga^4^, A Calmy^5^, T Sanchez^6^, E Delaporte^6^, C Casas^7^, S Khoo^8^, H Reynolds^8^, A Hill ^9^

^1^Faculty of Health Sciences, Ezintsha, University of the Witwatersrand, Johannesburg, South Africa; ^2^School of Medicine and Veterinary Medicine, University of Edinburgh, Edinburgh, UK; ^3^LSE Health, London School of Economics and Political Science, London, UK; ^4^University Teaching Hospital, Lusaka, Zambia; ^5^Division of Infectious Diseases, HIV‐AIDS Unit, Geneva University Hospitals, Geneva, Switzerland; ^6^TransVIHMI, University of Montpellier, Montpellier, France; ^7^Global Health Campus, Unitaid, Le Grand‐Saconnex, Switzerland; ^8^Department of Molecular and Clinical Pharmacology, University of Liverpool, Liverpool, UK; ^9^Department of Pharmacology and Therapeutics, University of Liverpool, Liverpool, UK

**Introduction**: First‐line treatment with dolutegravir (DTG) leads to rapid suppression of HIV RNA, which might then lower the risk of mother‐to‐child HIV transmission (MTCT). Worldwide, millions of women are taking DTG, so safety in pregnancy requires careful evaluation in randomised trials. Clinical obesity is associated with a wide range of adverse birth outcomes.

**Methods**: Data on adverse birth outcomes was included from five randomised trials: DolPHIN‐1, DolPHIN‐2, ADVANCE, NAMSAL and IMPAACT‐2010. These trials compared DTG with efavirenz (EFV) as first‐line treatment. In each trial, data on adverse events, adverse birth outcomes and MTCT were collected prospectively. Meta‐analysis was conducted using RevMan Software (version 5.3). The odds ratio (OR) for endpoints were calculated using the Cochrane Mantel‐Haenszel test (random‐effects model).

**Results**: DolPHIN‐1 and DolPHIN‐2 were conducted in South Africa and Uganda, ADVANCE in South Africa, NAMSAL in Cameroon and IMPAACT‐2010 internationally. DolPHIN‐1, DolPHIN‐2 and IMPAACT‐2010 were conducted in women already pregnant at screening. ADVANCE and NAMSAL were conducted in women who were not already pregnant at baseline. In the meta‐analysis, there were 26/657 stillbirths for DTG‐treated mothers versus 9/417 for EFV‐treated mothers (OR 1.77, p = 0.17) (Figure 1). There were five cases of MTCT for DTG arms and one for EFV (OR 2.80, p = 0.27). There were 14 neonatal deaths for DTG versus 13 for EFV (OR 0.91, p = 0.93). On combining the sum of stillbirths, MTCT and neonatal deaths there were 45 events for DTG versus 24 for EFV (OR 1.20, p = 0.49). No cases of neural tube defects (NTDs) were observed among infants born in any of the trials. In the ADVANCE trial, the risk of developing clinical obesity (BMI >30 kg/m^2^) was significantly higher for women taking DTG/FTC/TAF for 4 years (42%) versus DTG/FTC/TDF (27%) or EFV/FTC/TAF (20%).

**Conclusions**: In this analysis of 1074 pregnant women, there was no significant difference between DTG and EFV in the overall risk of neonatal deaths, stillbirths or MTCT cases. This analysis includes outcomes after first‐line treatment, typically up to 6 months before birth. Outcomes for women becoming pregnant after long‐term treatment could be different, given higher risks of clinical obesity for DTG, especially if combined with TAF/FTC.

**Figure:**
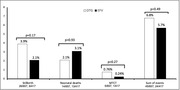
**Abstract P040 – Figure 1**. Results from meta‐analysis on adverse birth outcomes.

#### P041 Supported breastfeeding among women living with HIV in the UK: the current picture

K Francis, C Thorne, R Sconza, H Peters

The Integrated Screening Outcomes Surveillance Service, part of the NHS Infectious Diseases in Pregnancy Screening Programme, UCL Great Ormond Street Institute of Child Health, London, UK

**Background**: The UK HIV vertical transmission (VT) rate is <0.3% among diagnosed women living with HIV (WLHIV). The British HIV Association (BHIVA) recommends formula‐feeding, eliminating postnatal transmission, but states that virologically‐suppressed treated women with good adherence may be supported to breastfeed. Guidelines recommend monthly maternal and infant testing. The BHIVA March 2020 COVID‐19 statement discouraged breastfeeding owing to the testing burden, reverting to original guidance in September 2021.

**Materials/methods**: The Integrated Screening Outcomes Surveillance Service (ISOSS), part of the NHS Infectious Diseases in Pregnancy Screening Programme, commissioned by NHSE, monitors all pregnancies to WLHIV in the UK and of HIV‐diagnosed children <16 years. We describe clinical practice of supported breastfeeding using population‐level data.

**Results**: Among 8513 livebirth deliveries, 203 (2.4%) were reported as having supported breastfeeding, some women BF >1 infant. Cases increased four‐fold from <10 per year 2012 to 2014 to 40 to 50 per year 2019 to 2021. Of these, 94.5% (190/201) were in women diagnosed pre‐pregnancy and 84.0% (170/201) in women born abroad, with the majority from sub‐Saharan Africa. Median maternal age was 35 years (IQR 31 to 40) and BF duration ranged from 1 day to 2 years. Reported reason(s) for BF included: bonding (158), health benefits (137), family/friends’ expectations/pressures (51) and HIV‐disclosure concerns (60) (>1 reason reported in many cases). 80.2% (77/96) were known to have had monthly testing arranged in line with BHIVA guidelines. Attendance issues were reported in a quarter of cases (24/96). BF was reported to have stopped in 150/203, in most cases breastfeeding stopped as part of a plan however in 10 cases maternal viral load rebound prompted cessation. Among 150 infants where BF stopped, 106 had a negative antibody test (≥18 months); 34 are awaiting confirmatory testing and five were lost to follow‐up before infection status was confirmed.

**Conclusions**: Numbers of supported BF in the UK are small but increasing. Cases remain varied, particularly regarding duration and attendance for monthly testing. There are no vertical transmissions to date, but some infants are lost to follow‐up and/or still in follow‐up. Among VTs occurring in the UK, a number are attributable to undisclosed breastfeeding by women undetectable throughout pregnancy. Ongoing monitoring of clinical management remains essential to support future guidelines.

#### P042 Interim doravirine safety results from a pilot switch study for women of childbearing potential in South Africa

J Woods, S Sokhela, B Bosch, G Akpomiemie

Clinical Trials, Ezintsha (Wits Health Consortium), Johannesburg, South Africa

**Background**: Women comprise almost half of people living with HIV globally, yet fewer antiretroviral treatment (ART) options are available to them particularly in low‐ and middle‐income countries. Doravirine is a non‐nucleoside reverse transcriptase inhibitor, a potential alternative for women who do not tolerate either efavirenz‐ or dolutegravir‐based ART.

**Methods**: A pilot open‐label, single‐arm, single‐centre, phase III, switch study, recruited 100 HIV‐positive ART‐experienced women in South Africa, to evaluate viral suppression, tolerability, overall safety, and efficacy of DOR/3TC/TDF.

**Results**: In a 24‐week interim analysis, participants had: a mean age of 34 (IQR 22 to 49), 100 African black females and 32% unemployed. Pre‐study, 54% were on efavirenz and 47% on dolutegravir‐based ART, with baseline virological suppression (VL <50) at screening. No serious adverse events (AEs) were reported, with 16% of AEs possibly related to doravirine (97% of AEs were grade 1), there were no clinically significant neuropsychiatric outcomes. Statistically significant decreases in lipid panel from baseline to week 24 (n = 94) include: cholesterol ‐0.50 (IQR ‐0.60 to ‐0.39) p < 0.001; triglycerides ‐0.15 (‐0.23 to ‐0.07) p < 0.001; LDL ‐0.18 (‐0.27 to ‐0.09) p < 0.001; HDL ‐0.25 (‐0.31 to ‐0.19) p < 0.001. Glucose changes not significant. Median weight gain from baseline to week 24 (n = 95) was 2 kg (IQR 0 to 5.4) in 82% of patients (95% CI 74 to 88). Of these, there was 3.2 kg weight gain (SD 4.4) in the efavirenz group, and 1.4 kg (SD 3.7) in the dolutegravir group. Viral suppression at week 24 (n = 95): 92% HIV‐1 RNA <50 copies/mL; 7% RNA 50 to 1000 copies/mL, 1% RNA >1000 copies/mL (at week 48 [n = 40]: 95%, 3%, 2% respectively) (Figure 1). One patient developed doravirine resistance due to poor adherence. One pregnancy occurred, with good foetal outcomes.

**Conclusion**: Doravirine‐based regimes are an effective, tolerable alternative first‐line treatment in WCBP, with improved lipid profile, although potential long‐term weight gain needs to be further investigated.

**Figure:**
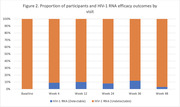
**Abstract P042 – Figure 1**. Proportion of participants and HIV‐1 RNA efficacy outcomes by visit.

#### P043 Guidelines and practice of breastfeeding in women living with HIV: a European survey

A Keane ^1^, F Lyons^1^, K Aebi‐Popp^2^, C Feiterna‐Sperling^3^, H Lyall^4^, A Martínez Hoffart^5^, H Scherpbier^6^, C Thorne^7^, H Albayrak Ucak^8^, A Haberl^9^

^1^Infectious Disease and Genitourinary Medicine, St James Hospital Dublin, Dublin, Ireland; ^2^Department of Infectious Diseases, University Hospital Bern, Bern, Switzerland; ^3^Klinik für Pädiatrie m. S. Pneumologie, Immunologie und Intensivmedizin, Charité Universitätsmedizin Berlin, Berlin, Germany; ^4^Paediatric Infectious Disease, Imperial College Healthcare NHS Trust, London, UK; ^5^Knowledge Network for Women Living with HIV, Posithiva Gruppen, Stockholm, Sweden; ^6^Department of Paediatrics, Amsterdam University Medical Centre, Amsterdam, Netherlands; ^7^Infectious Disease and Epidemiology, University College London, London, UK; ^8^Infectious Disease and Microbiology, Ankara City Hospital, Turkey; ^9^HIVCENTER University Clinic, University Clinic Frankfurt, Frankfurt, Germany

**Background**: Antiretroviral therapy (ART) is central to HIV prevention, including averting vertical transmission. The World Health Organization (WHO) recommends ART and exclusive breastfeeding for women living with HIV (WLWHIV) with particular focus on high HIV prevalence countries and settings in which diarrhoea, pneumonia and undernutrition are common causes of infant/child mortality [1]. There is a paucity of data regarding breastfeeding amongst WLWHIV in high income countries. Women Against Viruses in Europe (WAVE), part of the European AIDS Clinical Society (EACS) aims to improve the life of women and promote equality of access to care for WLWHIV.

**Materials and methods**: A WAVE‐convened steering group developed a survey asking about breastfeeding recommendations for WLWHIV across EACS countries and to determine desire to become involved in a network to further our understanding of breastfeeding in WLWHIV. The survey was disseminated to 31 WAVE members, EACS members or those identified as key respondents by WAVE. One response from each country was requested. Surveys were disseminated in March 2022 and results collated using the online platform Jotform.

**Results**: Thirty‐one countries were contacted, 28 responses from 26 countries. Three responses excluded: two duplicate country responses; one respondent only provided a link to national guidelines, not completing the survey. Twenty‐two of 25 (88%) countries have HIV and pregnancy guidelines; 20/22 (90%) refer specifically to breastfeeding. Nine of 18 (50%) recommend against breastfeeding, 7/18 (39%) offer an option if certain criteria are met. Eleven of 20 (55%) and 9/20 (45%) do not have recommendations for viral load testing during/after breastfeeding for mother and infant respectively. Twelve of 25 (48%) reported that the number of WLWHIV breastfeeding in their country was increasing while 12/25 (48%) reported it was stable. Seventeen of 25 (68%) reported no studies being done on breastfeeding in WLWHIV in their country. Twenty‐four of 25 (96%) respondents were interested in joining a WAVE network for learning and research around breastfeeding in WLWHIV.

**Conclusions**: Breastfeeding recommendations for WLWHIV vary across Europe, many national guidelines recommend against breastfeeding. Many countries reported an increase in numbers of WLWHIV breastfeeding whilst few are actively participating in research. With interest of colleagues around Europe, WAVE will work to establish a Breastfeeding Network to undertake research and improve support for WLWHIV who choose to breastfeed.

**Reference**

1. World Health Organization. Guideline: updates on HIV and infant feeding: the duration of breastfeeding, and support from health services to improve feeding practices among mothers living with HIV. Geneva: World Health Organization; 2016.

#### P044 Real‐world experience with the two‐drug regimen dolutegravir and lamivudine in women with HIV: a systematic literature review

S di Giambenedetto^1^, S Walmsley^2^, B Grinsztejn^3^, M Pérez‐Elías^4^, N Nwokolo^5^, M Kabra^6^, B Jones^7^, E Letang^8^, M Kisare ^9^

^1^Infectious Diseases, Fondazione Policlinico Universitario Agostino Gemelli Istituto di Ricovero e Cura a Carattere Scientifico, Rome, Italy; ^2^Infectious Diseases, University Health Network, Toronto, Canada; ^3^Infectious Diseases, Instituto Nacional de Infectologia Evandro Chagas‐Fiocruz, Rio de Janeiro, Brazil; ^4^Infectious Diseases, Hospital Universitario Ramón y Cajal, Madrid, Spain; ^5^Global Patient Affairs, ViiV Healthcare, Brentford, UK; ^6^Global Health Outcomes, ViiV Healthcare, Brentford, UK; ^7^Global Medical Affairs, ViiV Healthcare, Brentford, UK; ^8^Global Medical Affairs, ViiV Healthcare, Madrid, Spain; ^9^Global Medical Affairs, ViiV Healthcare, Nairobi, Kenya

**Background**: Women represent >50% of the global population with HIV but are underrepresented in clinical trials, leading to gaps in the scientific understanding of treatment considerations for women living with HIV (WLWHIV). Furthermore, treatment considerations for WLWHIV evolve during their life span. In 48‐week pooled analyses of phase III trials, 113 treatment‐naive WLWHIV initiating the two‐drug regimen dolutegravir + lamivudine (DTG + 3TC; GEMINI‐1/‐2) and 133 virologically suppressed WLWHIV switching to co‐formulated DTG/3TC (TANGO, SALSA) achieved or maintained high rates of virologic suppression with good tolerability. Real‐world studies can help address underrepresentation in clinical trials.

**Materials and methods**: A systematic literature review was conducted according to the Preferred Reporting Items for Systematic Reviews and Meta‐analysis statement. Real‐world studies of DTG + 3TC (dosed separately or as a fixed‐dose combination) in treatment‐naive and ‐experienced people with HIV were retrieved from January 2013 to February 2022.

**Results**: Overall, 122 publications of real‐world studies from 44 unique cohorts reported on DTG + 3TC use, representing 8034 people with HIV. Of these, 30 studies reported baseline sex at birth, representing 1512 WLWHIV; four studies reported outcomes in WLWHIV (N = 254; 240 virologically suppressed and 14 treatment‐naive at DTG + 3TC initiation), including four on effectiveness, two on safety, and one on tolerability. High rates of virologic effectiveness in WLWHIV on DTG + 3TC were observed across identified studies (96% to 100%; Figure 1), and there was no significant difference in odds of virologic suppression by sex at birth. Two studies reported more discontinuations in women versus men (Table 1). No real‐world studies reported on outcomes related to weight, effectiveness and birth outcomes in pregnancy, or addressed data gaps for specific groups of women across the age or gender spectrum.

**Figure:**
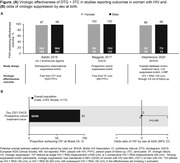
**Abstract P044 – Figure 1**. (A) Virologic effectiveness of DTG + 3TC in studies reporting outcomes in women with HIV and (B) odds of virologic suppression by sex at birth.

**Table:** 

**Abstract P044 – Table 1**. Discontinuations of DTG + 3TC in women and men with HIV.		
Maggiolo EACS 2017 (N = 203)		
Cause of discontinuation, n (%)	Cause of discontinuation, n (%)	Men (n = 153)
Discontinuations^a^	5 (10)^b^	7 (5)
Muscle aches	3 (6)	0
Headache	1 (2)	0
CNS symptoms	1 (2)	1 (<1)
Asthenia	1 (2)	0
Lost to follow‐up	1 (2)	1 (<1)
Cancer‐related metastasis	1 (2)	1 (<1)
Voluntary treatment interruption	0	2 (1)
Elevated liver function tests	0	1 (<1)
Alcohol‐induced cirrhosis	0	1 (<1)
**Stephenson BHIVA 2020 (N = 100)**		
Cause of discontinuation, n (%)	Women (n = 26)	Men (n = 74)
Discontinuations	4 (15)	2 (3)
CNS intolerance	1 (4)	0
Gastrointestinal intolerance	1 (4)	0
Virologic escape	1 (4)^c^	1 (1)^d^
Pregnancy	1 (4)	0
Arthralgia	0	1 (1)

3TC, lamivudine; BHIVA, British HIV Association; CNS, central nervous system; DTG, dolutegravir; EACS, European AIDS Clinical Society.

^a^All participants had viral load <50 copies/mL at last visit;

^b^two women had multiple causes of discontinuation: one with muscle aches and asthenia and one with headache, CNS symptoms, and muscle aches;

^c^viral load was 15 135 copies/mL before discontinuation. Virologic suppression achieved on bictegravir/emtricitabine/tenofovir alafenamide;

^d^viral load was 124 copies/mL before discontinuation. Virologic suppression achieved on DTG + tenofovir disoproxil fumarate/emtricitabine.

**Conclusions**: Available real‐world evidence for WLWHIV using DTG + 3TC supports results from phase III clinical trials, demonstrating high virologic effectiveness in this underrepresented group. The current paucity of real‐world data underscores the identified need to recruit women into well‐designed studies that assess outcomes beyond virologic effectiveness to properly address data gaps for WLWHIV.

#### P045 A wonderful experience: a difficult decision ‐ feedback from breastfeeding mothers living with HIV in Germany (SISTER study)

L Haberl ^1^, F Audebert^2^, F Borkel^3^, H Dorsch^4^, J Eger^5^, C Feiterna‐Sperling^6^, D Gillor^7^, U Haars^8^, P Jakuboswki^9^, C Jonsson‐Oldenbuettel^10^, P Khaykin^11^, R Kiener^12^, A Reitter^13^, A Rieke^14^, S Rößler^15^, I Rohr^16^, J Rump^17^, G Schuettfort^18^, M Speer^19^, C Stephan^18^, A Ulmer^20^, A von Braun^21^, K von Weizsäcker^16^, A Haberl^18^

^1^Clinic for Gastroenterology, Hepatology and Infectious Diseases, University Hospital Düsseldorf, Heinrich Heine University Düsseldorf, Düsseldorf, Germany; ^2^Hausärztl.‐ Internistische Gemeinschaftspraxis, Praxiszentrum Alte Mälzerei, Regensburg, Germany; ^3^Selbsthilfegruppe für Frauen und Mütter mit HIV, Frau+Mama, Berlin, Germany; ^4^Oberpfalz, Psychosoziale AIDS Beratungsstelle, Regensburg, Germany; ^5^Zentrum für Innere Medizin, Zentrum für Innere Medizin und Infektiologie, München, Germany; ^6^Department of Pediatrics, Charité Universitätsmedizin Berlin, Berlin, Germany; ^7^Innere Medizin, Medizinisches Versorgungszentrum Köln, Köln, Germany; ^8^Innere Medizin und Infektiologie, Infektiologie Krefeld, Krefeld, Germany; ^9^Center of Women's Health, University of Tübingen, Tübingen, Germany; ^10^HIV‐Schwerpunktpraxis, Medizinisches Versorgungszentrum München am Goetheplatz, München, Germany; ^11^Hausärztliche und Internistische Betreuung, MainFachArzt, Frankfurt, Germany; ^12^Kinderonkologie und ‐hämatologie, Universitätsklinikum Köln, Köln, Germany; ^13^Department of Obstetrics and Prenatal Medicine, Krankenhaus Sachsenhausen, Frankfurt, Germany; ^14^Department of Internal Medicine, Gemeinschaftsklinikum Mittelrhein, Koblenz, Germany; ^15^Klinik für Infektions‐ und Tropenmedizin, Klinikum Chemnitz, Chemnitz, Germany; ^16^Department of Obstetrics, Charité Universitätsmedizin Berlin, Berlin, Germany; ^17^Innere Medizin und Rheumatologie, Praxis Dr. Rump, Freiburg, Germany; ^18^Department of Infectious Diseases, University Hospital, Goethe University Frankfurt am Main, Frankfurt, Germany; ^19^Innere Medizin und Infektiologie, Praxis Kreuzberg, Berlin, Germany; ^20^Gemeinschaftspraxis, Schwabstraße 26, Stuttgart, Germany; ^21^Division of Infectious Diseases and Tropical Medicine, University Hospital Leipzig, Leipzig, Germany

**Background**: Guidelines do not recommend breastfeeding (BF) for mothers living with HIV (MLWHIV) in resource‐rich countries. However, the number of breastfeeding MLWHIV is increasing in some of these countries, such as Germany. Regarding BF and HIV there are still important data gaps. The SISTER study aims to depict experiences of breastfeeding MLWHIV in Germany.

**Methods**: SISTER was launched in April 2019. An interdisciplinary study group including HIV community representatives designed a questionnaire to capture BF experiences of MLWHIV in Germany. Study materials are distributed by community networks and HIV specialists.

**Results**: Thirty‐eight MLWHIV participated in the study to date. Median age: 34 (18 to 43); 23 (61%) born abroad; 33 (87%) completed education. All MLWHIV had a viral load <50 copies/mL at start of BF. Maternal ART during BF period: 61% NNRTI, 32% INSTI, 5% PI. Sixteen (42%) women already had BF experience. Most important reason for BF was bonding (87%). Information on BF came from HIV specialists (82%), internet (26%) and/or gynaecologists (47%). MLWHIV rated reactions to BF from 1 to 5 (1 = most negative; 5 = most positive reaction). Ranking: HIV specialists 4.5; gynaecologists 4.0; midwives 4.3; paediatricians 3.1; nursing staff 3.0. Thirty‐two percent of MLWHIV did not disclose HIV to midwives; 29% not to nursing staff. Ten percent each did not inform gynaecologists or paediatricians about BF; 5% did not inform HIV specialists. Median duration of BF: 24 weeks (1 to 104). Eighty‐seven percent of MLWHIV would breastfeed again; 84% would recommend breastfeeding to other MLWHIV.

**Conclusions**: The SISTER study is the first study collecting data on breastfeeding experiences of MLWHIV in Germany. So far 38 women participated in the study. MLWHIV were healthy, well educated, on effective ART and almost half of them already had BF experience. The majority of MLWHIV gave a positive feedback to BF. However, some women did not disclose their HIV status to health care providers (HCPs). In the group who disclosed, negative reactions of HCPs were reported. This could be due to missing or outdated information, but also to negative attitudes towards breastfeeding of MLWHIV. One key finding of SISTER is that MLWH who breastfeed have a risk of experiencing HIV‐related discrimination.

#### P046 HIV testing reasons from 2000 to 2020 in an active cohort of women living with HIV (WLWHIV) in a tertiary hospital in Barcelona, Spain: a retrospective study

S Toyos^1^, L Berrocal^2^, A González‐Cordón^2^, E Fernández^2^, A Inciarte^2^, L de la Mora^2^, M Martínez‐Rebollar^2^, M Laguno^2^, J Ambrosioni^2^, I Chivite^2^, E de Lazzari^2^, J Blanco^2^, E Martínez^2^, J Miró^2^, J Mallolas^2^, B Torres ^2^

^1^Internal Medicine, Hospital Verge de la Cinta, Tortosa, Spain; ^2^HIV Unit, Infectious Diseases Service, Hospital Clínic, Barcelona, Spain

**Background**: Early diagnosis of HIV is key to prevent disease progression and viral transmission. In some regions WLWHIV represent a low percentage of PLWHIV and prevention strategies in women are often scarce. The present study aimed to analyse the main reasons for HIV testing and to describe the proportions of late and advance disease stage at diagnosis accordingly among WLWHIV visiting the HIV unit of a tertiary hospital in Barcelona, Spain, between 2000 and 2020.

**Materials and methods**: WLWHIV in current follow‐up and diagnosed from January 2000 to December 2020 for whom the variable 'reason for testing' was reported were retrospectively included. Age at diagnosis, country of origin, period of diagnosis (2000 to 2009 vs 2010 to 2020), first viral load and first CD4 count were analysed. The category 'reason for testing' was divided into seven groups: reproductive health (including pregnancy and reproductive health study), AIDS‐defining events (ADE), diagnosis in a sexual partner, indicator condition (non‐ADE), patient‐motivated routine control, physician‐motivated routine control and 'other'. Multinomial logistic regressions were performed with 'reason for testing' as the dependent variable. Stata 17 software was used.

**Results**: Three hundred and nineteen WLWHIV were included in the study. Overall, the main reason for HIV testing was the presence of an indicator condition (25%), followed by reproductive health (21%), sexual partner (20%), physician‐motivated routine control (18%), ADE (10%), other reasons (4%) and patient‐motivated routine controls (2%). Less testing was performed due to ADE in the period 2010 to 2020 (4% vs 14%) and more due to diagnosis in the sexual partner (25% vs 17%), respectively. Baseline CD4 count <350 cells/mm^3^ was present in 71% of women in the indicator condition group. Women diagnosed for causes other than reproductive health had a higher relative risk of being older. No differences were observed in the form of diagnosis depending on the country of origin.

**Conclusions**: Indicator condition was the main reason leading to diagnosing in WLWHIV in this study. However, late diagnosis was high in this group. New strategies to improve early diagnosis in WLWHIV are necessary, especially in settings where women represent a low percentage of PLWHIV compared to other population groups.

#### P047 HIV and women in Latin America: characteristics, therapy, and outcomes ‐ results from the Latin American HIV Workshop Study Group (LAHWSG)

P Zitko ^1^, I Cassetti^2^, P Celi^3^, E Barthel^4^, C Mazariegos^5^, S Pereira^6^, M Rodriguez^7^, L Astocondor^8^, E Álvarez^9^, C Beltran^10^

^1^Unit of Healthcare Research, Hospital Barros Luco Trudeau, Santiago, Chile; ^2^HIV/AIDS, Helios Salud Ambulatory AIDS Care Center, Buenos Aires, Argentina; ^3^HIV/AIDS, Hospital de Especialidades de las Fuerzas Armadas, Quito, Ecuador; ^4^HIV/AIDS, Hospital Carlos Van Buren, Valparaiso, Chile; ^5^Clínica de Atención Integral, Hospital Regional de Zapaca, Zapaca, Guatemala; ^6^HIV/AIDS, Asociación Española, Montevideo, Uruguay; ^7^HIV/AIDS, Círculo Católico de Obreros y Empleados del Uruguay, Montevideo, Uruguay; ^8^HIV/AIDS, Hospital Daniel Carrión Callao, Callao, Peru; ^9^HIV/AIDS, Hospital Dr. Salvador B. Gautier, Santo Domingo, Dominican Republic; ^10^Infectology, Hospital Barros Luco Trudeau, Santiago, Chile

**Background**: HIV data usually are shown without desegregating by gender, obscuring dissimilarities. Latin America is one of the regions with fewest research in HIV. We aim to describe age distribution, count of CD4, first antiretroviral therapy, and outcomes 1 year after admission to care in women living with HIV from centres of the LAHWSG.

**Material and methods**: We performed a cross‐sectional and time‐series analysis using aggregated administrative data from centres of the LAHWSG. Cross‐sectional data were collected for 31 December 2017. Time‐series analysis covered admissions to HIV outpatient care between 2013 and 2017. Outcomes account the period between admission during 2016 until the end of 2017. Three multilevel random‐effect meta‐regressions were implemented, weighting results by the expected number of people living with HIV in each country and features of centres. Heterogeneity I^2^ parameter is reported.

**Results**: Seventy‐one out of 101 centres from 14 Latin‐American countries completed the data for the analysis, gathering information from 25 816 women. 27.5% [95% CI 22.7 to 31.2; I^2^centre = 66.0%, I^2^country = 33.9%] of patients under care were women, mostly aged 30 to 39 years. The number of women annually admitted to care increased between 2013 and 2017; however, compared with men the proportion decreased. More than a half of admitted women between 2013 and 2017 had <350 CD4 cells/mL count: 59.6% [95% CI 52.3 to 66.8; I^2^centre = 55.9%, I^2^country = 43.3%]. Proportion of late presenters at admission remained constant during the period. Zidovudine use as part of first antiretroviral therapy diminished from 32.6% [20.4 to 44.7; I^2^centre = 91.6%, I^2^country = 8.0%] in 2013 to 11.1% [1.9 to 20.3; I^2^centre = 87.9%, I^2^country = 11.9%] in 2017, while efavirenz use as third drug remained constant around fifty percent, and a slight increase of INSTI use. Overall, the largest gap in outcomes is observed in retention in care: 80.0% [71.6 to 88.5; I^2^centre = 70.6%, I^2^country = 28.7%] of admitted women. Viral suppression was achieved in 73.3% [67.0 to 79.7; I^2^centre = 98.4%, I^2^country = 0%] of cases. I^2^ for centre and country levels are reported as a measure of variability.

**Conclusions**: In this study, one with largest collection of information from women in Latin America, females represent a significant part of HIV‐infected people under care. A high proportion of women present late to care. Important changes have occurred in antiretroviral use, while a high variability in outcomes between centres and countries is observed.  More interventions are needed to ensure retention in care of women in Latin America.

#### P048 Under the covers: HIV and women in Saskatchewan, Canada

C Spence ^1^, D Magnusson^2^, L Kiesman^3^, B Wudel^4^, E Yip‐Liang^1^, S Kogilwaimath^4^

^1^Medicine, University of Saskatchewan, Saskatoon, Canada; ^2^Nursing, Westside Community Clinic, Saskatoon, Canada; ^3^Medicine, Westside Community Clinic, Saskatoon, Canada; ^4^Infectious Diseases, Royal University Hospital, Saskatoon, Canada

**Background**: The prairie province of Saskatchewan (SK) has had the highest rates of HIV in Canada since 2009, with almost 4x the national average, and 45% of cases within the urban center of Saskatoon. This unique epidemic is driven largely by injection drug use (IVDU) among a largely Indigenous, marginalized population. The risk for co‐infection with HCV and tuberculosis (TB), and other opportunist infections including endocarditis and cancer, are amplified by malnutrition/food insecurity and unsafe/unstable social and housing environments. Care of HIV in Saskatoon is primarily accessed in two centers ‐ a specialized acute care hospital setting and a primary community‐based clinic. The two clinics serve more than 2000 active patients with HIV. The deficits in services and resources since the onset of COVID‐19 have enhanced infection and disease interaction, resulting in an increase in exposures and infections across the population. Also during this time, syphilis rates have exploded within the population and pregnancy rates have doubled.

**Materials and methods**: A gendered analysis was conducted on clinical variables extracted from electronic medical records (EMR) for the two Saskatoon based clinics and hospitalization data for inpatient and pregnant HIV individuals.

**Results**: Woman with IVDU as a HIV risk factor are disproportionately represented, with 86% of new diagnosis among Indigenous women, most of reproductive age (15 to 39 years old). Fifty percent of all cases progress to AIDS within 3 years of diagnosis and fatality within 5 years. The findings are consistent among opportunist infections, such as endocarditis, where 58% infected are women. Syphilis rates have increased almost 600% in the past 2 years and cases of babies born with HIV in 2022 because healthcare was not accessed.

**Conclusions**: Since the onset of COVID‐19, this unique HIV population has been increasingly marginalized and access to services has been limited, resulting in poor health outcomes and premature death.

### Treatment Strategies ‐ Target Populations: Late Presenters

#### P049 Effects of advanced HIV disease on inflammation following ART initiation

O Bisbal ^1^, S Serrano‐Villar^2^, L Domínguez‐Domínguez^1^, M Rava^3^, C Guitierrez^4^, A Rull^5^, L Pérez‐LaTorre^6^, F Gutierrez^7^, M Saumoy^8^, C Amador^9^, I Jarrin^3^, J Iribarren^10^, R Rubio^1^, S Moreno Guillén^2^

^1^HIV Unit, University 12 Octubre Hospital, Madrid, Spain; ^2^HIV Unit, University Ramon y Cajal Hospital, Madrid, Spain; ^3^National Epidemiology Center, Institute of Health Carlos III, Madrid, Spain; ^4^Laboratory, Institute of Health Research Ramón y Cajal, Madrid, Spain; ^5^Infectious Diseases Department, University Hospital of Tarragona Joan XXIII, Tarragona, Spain; ^6^HIV Unit, Gregorio Marañon University Hospital, Madrid, Spain; ^7^HIV Unit, University General Hospital of Elche, Elche, Spain; ^8^HIV Unit, Bellvitge University Hospital, Barcelona, Spain; ^9^HIV Unit, Marina Baixa Hospital, Villajoyosa, Spain; ^10^HIV Unit, Donostia Hospital, San Sebastián, Spain

**Background**: Since early ART initiation has been shown to reduce immune activation, we explored the differences associated with late versus early ART initiation on changes on biomarkers of inflammation

**Method**: We selected 100 patients from the Cohort of the Spanish HIV/AIDS Research Network (CoRIS) based on an early (CD4 >350/mm^3^) or late (CD4 <100/mm^3^) presentation at cohort entry and seeking representation of the main treatment regimens in both groups. Outcomes were changes in four plasma biomarkers, previously associated with mortality (hsCRP, sCD14, D‐dimer, and IFABP) measured at baseline, week 48 and 96. We modelled the biomarker changes using linear mixed models, which included the interaction term time‐versus‐treatment group as a fixed‐effect and a random effect for each patient. We adjusted for sex, age, ART regimen, period of enrolment, risk factor for HIV acquisition and region of origin. Continuous outcome variables were log‐transformed to satisfy model assumptions.

**Results**: From 100 participants, 98 were evaluable: 50 early presenters (EP) and 48 late presenters (LP). Eighty‐four percent were males, 66% MSM and mean ± SD age was 39±10 years. No significant differences were found in sex, age, type of ART, period of enrolment, transmission category, educational level and country of origin. Mean ± SD CD4 count was 484.8 cells/microL ± 177.9 in EP and 44.5 cells/microL ± 34.8 in LP. At baseline, sCD14 and D‐dimer levels were significantly higher in LP compared to EP (p < 0.001 and p = 0.003, respectively), without significant differences in baseline hsCRP (p = 0.074) or IFABP (p = 0.729). The levels of hs‐CRP or IFABP did not appear affected by either the study group or time (differences in global trajectories, p = 0.421 and p = 0.671, respectively). However, sCD14 and D‐dimer decreased faster in LP compared to EP during the first 48 weeks, approaching the levels achieved in the EP group at 96 weeks (p = 0.052 and p = 0.682) (Figure 1).

**Conclusions**: Despite the fact that LP exhibit higher baseline sCD14 and D‐dimer levels than EP, these levels tended to converge after 96 weeks. No differences were found in hsCRP and IFABP. Our study suggests that advanced HIV disease impacts on monocyte activation and pro‐thrombotic pathways, although this effect is attenuated following ART.

**Figure:**
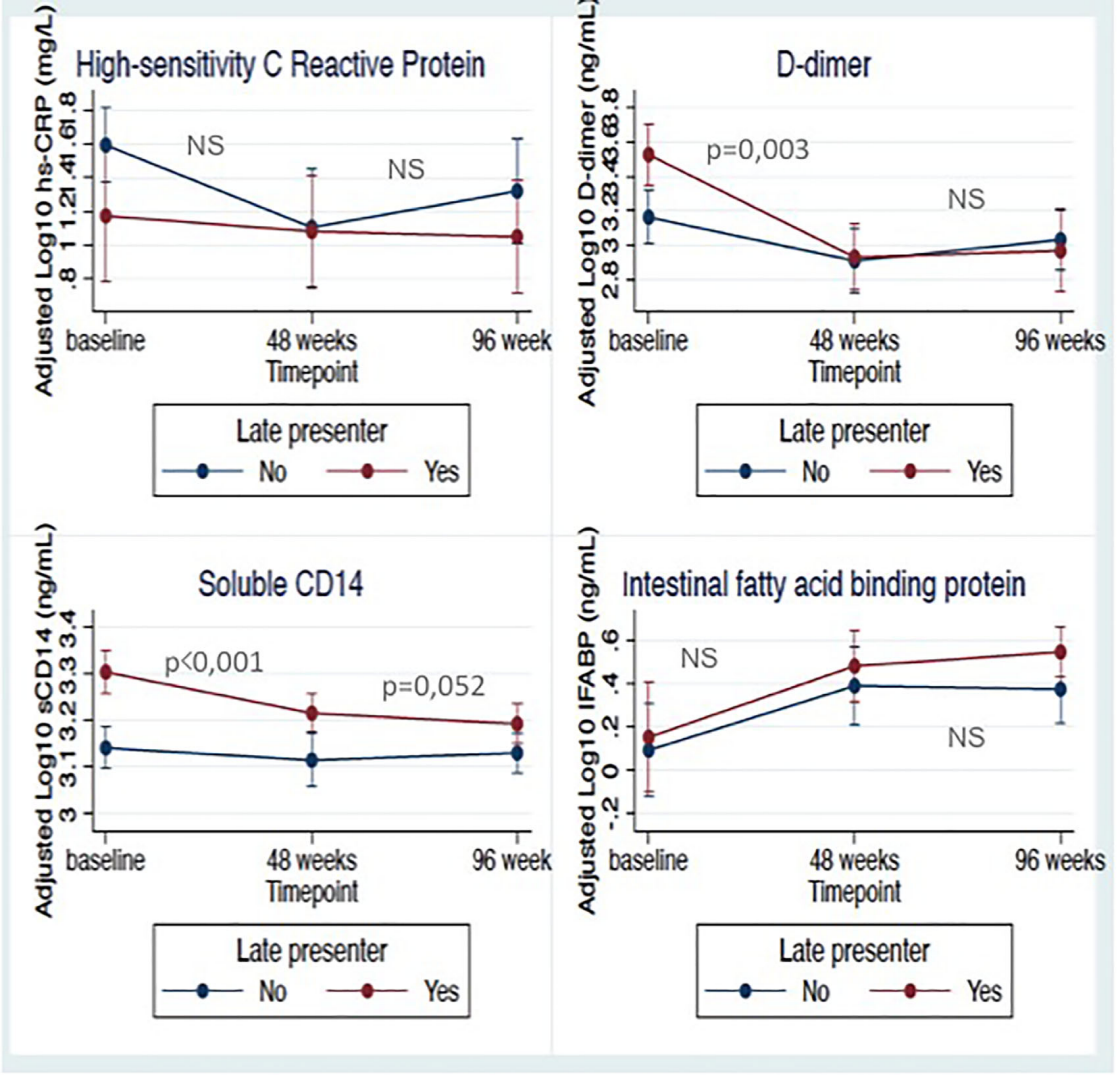
**Abstract P049 – Figure 1**. Comparison of the evolution of inflammation markers (hs‐CRP, D‐dimer sCD14 and IFABP) in HIV late and early presenters at 48 and 96 weeks.

#### P050 Comparison of the efficacy of preferred antiretroviral regimens in patients with CD4 cell counts <200 versus 200/L or VL >100 000 versus 100 000 copies/mL: a systematic review with meta‐analysis. GESIDA‐11520 Study

J Perez‐Molina^1^, C Crespillo‐Andujar^1^, J Zamora^2^, B Fernández‐Félix^2^, A Gaetano^2^, J López Bernaldo de Quirós^3^, S Serrano‐Villar^1^, S Moreno Guillén ^1^, J Berenguer^3^

^1^Infectious Diseases Dept., Hospital Universitario Ramón y Cajal, Madrid, Spain; ^2^Clinical Biostatistics Dept., Hospital Universitario Ramón y Cajal, Madrid, Spain; ^3^Infectious Diseases Unit, Hospital General Universitario Gregorio Marañón, Madrid, Spain

**Background**: To assess the impact of low CD4 cell counts or high HIV‐RNA viral load on the efficacy of currently preferred antiretroviral treatment (ART) regimens for HIV‐1‐infected naïve adults.

**Materials and methods**: We selected first‐line ART recommended in the leading guidelines (US DHHS, IAS USA, EACS). We performed a systematic review from January 2006 to September 2021, including randomised controlled clinical trials (RCTs) with at least 48 weeks of follow‐up and subgroup analysis by CD4 count (≤ or > 200 CD4 cells) or viral load (≤ or > 100 000 copies) at baseline (https://www.doi.org/10.17605/OSF.IO/S7AHX). The primary endpoint was treatment efficacy, measured as the proportion of participants with an undetectable VL at 48 weeks by intent‐to‐treat analysis. We computed the OR of treatment failure (TF) rate for each subgroup and individual treatment arm. We pooled ORs using a random‐effects model.

**Results**: We identified a total of 1223 articles of which we finally selected 23, corresponding to 12 RCTs: 17 ART arms (6597 participants) for CD4 cell and 18 ART arms (6845 participants) for VL subgroups, respectively. Patients with ≤200 CD4 cell or VL >100 000 copies showed an increased odds of TF at 48 weeks: ORs 1.94 (95% CI 1.45 to 2.61; prediction interval 0.74 to 5.1) (Figure 1) and 1.74 (95% CI 1.28 to 2.36; prediction interval 0.54 to 5.55), respectively. There was no significant heterogeneity regarding INSTI, NRTI backbone, quality of subgroup analysis, or study's year of publication. At 96 weeks, OR for TF were 1.46 (95% CI 1.01 to 2.11) and 1.54 (95% CI 1.26 to 1.88) for CD4 cell ≤200 and VL >100 000 subgroups, respectively. Significant heterogeneity was detected at 96 weeks for the NRTI backbone. VL >500 000 copies at 48 weeks increased the OR of TF to 2.73 (95% CI 1.65 to 4.53).

**Figure:**
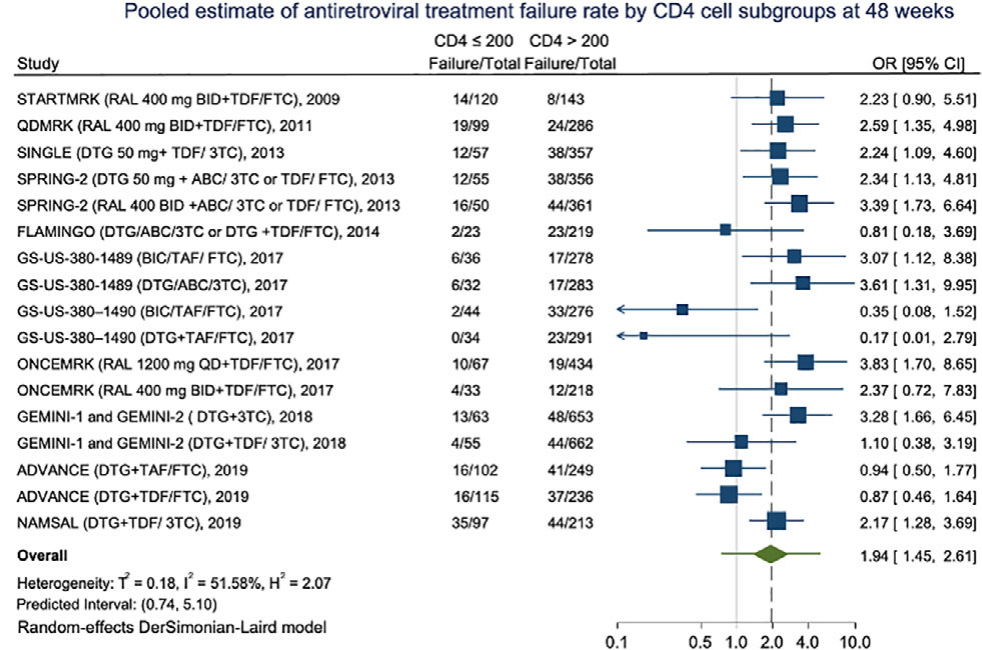
**Abstract P050 – Figure 1**. Pooled estimate of antiretroviral treatment failure rate by CD4 cell subgroups at 48 weeks.

**Conclusions**: Low CD4 cell counts and high VL are associated with poorer ART outcomes in treatment‐naïve patients. At 48 weeks, this effect does not seem related to the individual drugs comprising the ART regimen though more information is needed given the high uncertainty of the estimations. As these factors are not modifiable in treatment‐naive patients, comparative RCTs should determine the best ART in patients with severe immunosuppression or high VL.

#### P051 Predictors of HIV late presentation and barriers to the HIV testing in Central region of Ukraine

T Koval ^1^, O Marchenko^2^, A Mischenko^3^, S Nesterenko^3^

^1^Infectious Diseases, Poltava Regional Clinical Infectious Hospital, Poltava, Ukraine; ^2^Infectious Diseases, Poltava State Medical University, Poltava, Ukraine; ^3^Infectious Diseases, Poltava Regional HIV/AIDS Prevention and Control Center, Poltava, Ukraine

**Background**: In Ukraine about one half of new HIV‐positive individuals are late presenters every year. Despite the military actions in Ukraine from February 2022 observed significant disruptions in the provision of HIV prevention and treatment programmes in sоme regions. Amount of internally displaced people has significantly increased in the Central region of Ukraine and respectively new challenges observed in the diagnosis of HIV infection. We aimed to explore predictors for late HIV diagnosis and barriers to the HIV testing.

**Method**: We analysed the medical records of 115 new HIV‐infected patients registered in Poltava Regional AIDS Center from January 2021 to May 2022. We conducted questionnaires with 42 late presenters. Nadir CD4 count <350 cells/mL and/or AIDS‐defining illness defined as late presenters. Binary and multinomial logistic regressions were used to identify the predictors for late HIV presentation and barriers to the HIV testing.

**Results**: Among 115 new HIV‐infected patients, 51 (44.3%) were late presenters. Main predictors of late presentation identified as male sex (74.5%, p = 0.02), history of injecting drugs (65.3%, p = 0.045), age >40 years (52.0%, p = 0.05) and lower educational level (p = 0.001). Most late presenters visited of different medical clinics with symptoms during last year preceding HIV test (76.8%, p = 0.001) and obtained first HIV test following a doctor's recommendation (62.9%, p < 0.05). The main barriers to the HIV testing in 2022 were fear of disclosure HIV diagnosis (OR 2.72, 95% CI 1.48 to 5.53), not feeling ill (OR 1.25, 95% CI 1.10 to 3.25) and war‐related anxiety (OR 2.05, 95% CI 1.15 to 3.76).

**Conclusion**: This study suggests association between the male sex, age >40 years, drug using, lower educational level and late presentation of HIV. The barriers to the HIV testing in 2022 were associated with fear of disclosure HIV diagnosis and war‐related anxiety. Implementation of new HIV testing strategies and enhanced HIV testing in medical clinics and nongovernment organisations are urgently needed.

#### P052 High rates of virological suppression after 24 weeks of tenofovir alafenamide/emtricitabine/bictegravir (BIC/FTC/TAF) in people living with HIV (PLWHIV) starting antiretroviral therapy with <200 CD4 cell count

I Pérez‐Valero^1^, D Corona Mata ^1^, A Camacho Espejo^1^, C Roca‐Oporto^2^, C Tomas^3^, N Cabello^4^, M Cervero Jimenez^5^, M Navarro^6^, A Rivero‐Juarez^7^, A Rivero Roman^1^

^1^Enfermedades Infecciosas ‐ VIH, Hospital Universitario Reina Sofia ‐ (IMIBIC), Cordoba, Spain; ^2^Enfermedades Infecciosas, Hospital Universitario Virgen del Rocio, Sevilla, Spain; ^3^Enfermedades Infecciosas, Hospital Reina Sofia, Murcia, Spain; ^4^Enfermedades Infecciosas, Hospital Clínico San Carlos, Madrid, Spain; ^5^Servicio de Medicina Interna, Hospital Universitario Severo Ochoa, Leganes, Spain; ^6^Enfermedades Infecciosas, Parc Taulli Hospital Universitari, Barcelona, Spain; ^7^Grupo de Virología Clínica y Zoonosis, Instituto Maimónides de Investigación Biomédica de Córdoba (IMIBIC), Cordoba, Spain

**Background**: There is limited evidence regarding the effectiveness of BIC/FTC/TAF in PLWHIV starting ART with a CD4 cell count <200 cells/mm^3^ (CD4<200) or previous AIDS diagnosis.

**Materials and methods**: Retrospective analysis performed to evaluate the rate of virological suppression (VS) after 24 weeks of BIC/FTC/TAF in PLWHIV with severe immunodepression. All the participants with CD4<200 or a previous diagnosis of AIDS enrolled in a multicentre, prospective, Spanish HIV cohort (CORIS), who started therapy with BIC/FTC/TAF (2019 to 2020) and with at least 24 weeks of follow‐up, were included and allocated in two groups based on their initial ART regimen: BIC/FTC/TAF versus other regimens. Our primary objective was to evaluate the rate of VS (HIV‐RNA <50 copies/mL) at week 24 with BIC/FTC/TAF. As secondary objectives we compared the rates of VS in both study groups, using chi‐square, and we assessed factors associated with achieving VS at week 24 using logistic regression.

**Results**: Between 2019 and 2020, 232 CORIS participants started ART; 95 (41%) with BIC/FTC/TAF and 137 (59%) with other regimens. Baseline characteristics (Table 1) were similar between groups. After 24 weeks of therapy, 73.7% of the participants starting BIC/FTC/TAF achieved VS versus 59.9% of the participants starting other regimens. The probability of achieving VS at week 24 was 1.9 times higher with BIC/FTC/TAF than with other regimens (95% CI 1.1 to 3.3). Factors independently associated with achieving VS at week 24 were being on BIC/FTC/TAF (OR 2.2 [95% CI 1.1 to 4.2]) and HIV‐RNA >100 000 copies/mL at baseline (OR 0.2 [95% CI 0.1 to 0.3]).

**Conclusions**: In the CORIS cohort, starting ART with BIC/FTC/TAF in PLWHIV with severe immunodepression was associated with high rates of effectiveness at week 24.

**Table:** 

**Abstract P052 – Table 1**. Baseline characteristics.			
Baseline characteristics	BIC/FTC/TAF (n=95)	Other ART options (n=137)	p‐value
Gender: Women, n (5)	12 (12.6)	21 (15.3)	0.563
Age (yrs), median (IQR)	39.9 (31.7 ‐ 48.1)	39.6 (32.8 ‐ 49.0)	0.532
Way of transmission, n (%)			
Homosexual	52 (54.7)	75 (55.5)	0.088
Heterosexual	39 (41.1)	43 (31.4)	
Intravenous drug use	1(1)	2 (1.4)	
Unknown	3 (3.2)	16 (11.7)	
Country of born, n (%)			
Spain	45 (47.4)	67 (48.9)	0.818
Other	50 (52.6)	70 (51.1)	
AIDS diagnosis, n (%)	35 (36.8)	49 (35.8)	0.867
CD4 nadir, n (%)	103 (53 ‐ 162)	92 (47 ‐ 167)	0.566
CD4 nadir <59 cel/mm^3^, n (%)	22 (23.2)	33 (24.1)	0.142
HIV RNA >10⁵ cop/mL	61 (64.2)	85 (62.0)	0.639

#### P053 Risk factors associated with late presentation to HIV care in the 'treat all' era in sub‐Saharan Africa: a systematic literature review

D Mwamba

HIV Programs, Centre for Infectious Diseases Research in Zambia, Lusaka, Zambia

**Introduction**: Late presentation to HIV care (with CD4 count <350 cells or World Health Organization [WHO] clinical stage 3 or 4) remains a norm in sub‐Saharan Africa. This is despite the 2016 WHO HIV guidelines recommending the initiation of antiretroviral therapy (ART) in all people living with HIV regardless of clinical and immunological status. The aim of this systematic review was to describe the prevalence and demographics of adults aged >15 years who are late to present to HIV care in sub‐Saharan Africa

**Methods**: PubMed, Embase, ISI Web of Knowledge, Health System Evidence Global Index Medicus databases, web engines and conference websites were searched for relevant studies, grey literature and abstracts conducted between 2015 and 2020 (Figure 1).

**Results**: Nine studies were included in the review. Males represented 58% of the total 714 929 participants aged >15. The prevalence of late presentation to care was 44% (95% CI 37 to 51). The odds of late presentation to care for males was 1.54 (95% CI 1.05 to 2.36); aged >36 was 1.55 (95% CI 0.98 to 2.69); not being married was 1.065 (95% CI 0.99 to 1.15).

**Figure:**
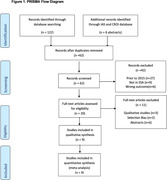
**Abstract P053 – Figure 1**. PRISMA flow diagram.

**Conclusion**: Late presentation to HIV care remains high among adults living with HIV in sub‐Saharan Africa. Being male, not married, and being above 35 years of age were found to be associated with higher odds of late presentation to care. Strategies that allow early HIV detection and treatment and innovative approaches targeting population at risk are needed to achieve expected HIV programme outcome of the 'treat all' policy.

### Treatment Strategies ‐ Target Populations: Naive Patients

#### P054 Transmitted drug resistance in Spain (CoRIS) patients during 2019 to 2021

L Viñuela^1^, A Fuentes‐López^1^, A de Salazar^1^, E Serrano‐Conde^1^, M Pérez‐Elías^2^, J Olalla^3^, O Arce‐García^4^, J Iribarren^5^, M Masiá^6^, M Montero‐Alonso^7^, I Falces‐Romero^8^, J Blanco^9^, M Rivero^10^, L García Fraile^11^, N Espinosa Aguilera^12^, B Baza^13^, A Aguilera^14^, M Maciá^15^, M Martínez‐Velasco^16^, A Iborra^17^, A Imaz^18^, A López Lirola^19^, J Peraire^20^, I Portilla^21^, A Rando^22^, I Suárez‐García^23^, R Izquierdo^24^, F Garcia ^1^

^1^Microbiology, Hospital Universitario Clínico San Cecilio, Granada, Spain; ^2^Infectious Diseases and Microbiology, Hospital Ramón y Cajal, Madrid, Spain; ^3^Infectious Diseases and Microbiology, Hospital Costa del Sol, Marbella, Spain; ^4^Infectious Diseases and Microbiology, Hospital Universitario 12 de Octubre, Madrid, Spain; ^5^Infectious Diseases and Microbiology, Hospital Universitario de Donosti, San Sebastian, Spain; ^6^Infectious Diseases and Microbiology, Hospital General Universitario de Elche, Elche, Spain; ^7^Infectious Diseases and Microbiology, Hospital Universitario La Fe, Valencia, Spain; ^8^Infectious Diseases and Microbiology, Hospital Universitario La Paz, Madrid, Spain; ^9^Infectious Diseases and Microbiology, Hospital San Pedro, Logroño, Spain; ^10^Infectious Diseases and Microbiology, Hospital de Navarra, Pamplona, Spain; ^11^Infectious Diseases and Microbiology, Hospital La Princesa, Madrid, Spain; ^12^Infectious Diseases and Microbiology, Hospital Virgen del Rocio, Sevilla, Spain; ^13^Infectious Diseases and Microbiology, Centro Sanitario Sandoval, Madrid, Spain; ^14^Infectious Diseases and Microbiology, Complejo Hospitalario Santiago de Compostela, Santiago de Compostela, Spain; ^15^Infectious Diseases and Microbiology, Hospital Son Espases, Mallorca, Spain; ^16^Infectious Diseases and Microbiology, Hospital Universitari Mutua Tarrasa, Tarrasa, Spain; ^17^Infectious Diseases and Microbiology, Hospital Virgen Arrixaca, Murcia, Spain; ^18^Infectious Diseases and Microbiology, Hospital Universitario de Bellvitge, Bellvitge, Spain; ^19^Infectious Diseases and Microbiology, Hospital Universitario de Canarias, Las Palmas, Spain; ^20^Infectious Diseases and Microbiology, Hospital Universitari Joan XXIII, Tarragona, Spain; ^21^Infectious Diseases and Microbiology, Hospital Alicante, Alicante, Spain; ^22^Infectious Diseases and Microbiology, Hospital Universitario Vall d'Hebron, Barcelona, Spain; ^23^Infectious Diseases and Microbiology, Hospital Infanta Sofía, Madrid, Spain; ^24^Infectious Diseases and Microbiology, Instituto de Salud Carlos III, Madrid, Spain

**Background**: Evaluation of transmitted drug resistance (TDR) in Spain (CoRIS) is running since 2007. In this paper we present the results of the TDR update in newly diagnosed patients in CoRIS for the period 2019 to 2021.

**Methods**: Newly diagnosed patients from CoRIS centres with FASTA sequences available were included. After FASTA quality control, mutations in RT and Pro associated with TDR were investigated (CPR‐Stanford tool, based on Bennett, 2009). For the integrase, we also used the CPR‐Stanford tool. In addition, we also used Stanford interpretation to evaluate clinically relevant resistance to the drugs currently recommended as first‐line treatment in the GESIDA guidelines, as well as to the NNRTIs EFV, DOR and RPV.

**Results**: A total of 1859 patients were analysed, 758 (2019), 567 (2020) and 534 (2021); of these, integrase data were available in 651 patients, 264 (2019), 167 (2020) and 220 (2021). TDR in the period 2019 to 2021 was: 3.7% for NRTIs, 6.02% for NNRTIs, 0.97% for PIs, and 0.37% for INIs. In 2021 it was 3.2% for NRTIs, 5.9% for NNRTIs, 0.75% for PIs, and 0% for INIs.  Clinically relevant resistance to first‐line drugs in the period 2019 to 2021 was 1.3% for TDF (of which 1.2% were Intermediates), 2% for ABC, 0.8% for 3TC/FTC, 6.4% for EFV, 2.6% for DOR (1.5% I), 7.1% for RPV, 2% for RAL (all Intermediate), 0.15% for BIC and DTG (n = 1, S153F) and 0.05% for DRV; and in 2021, it was 1.2% for TDF (all Intermediate), 1.7% for ABC (all Intermediate), 0.2% for 3TC/FTC, 6.2% for EFV, 2% for DOR (1.3% Intermediate), 8.8% for RPV (6.7% Intermediate), 2.4% for RAL (all Intermediate); no patient showed clinically relevant resistance to BIC, DTG and DRV.

**Conclusions**: As in previous years, the highest prevalence of transmitted resistance occurred for NNRTIs, with doravirine being the drug in this family with the lowest levels of TDR. Transmitted resistance to PIs, INIs, and 3TC/FTC continues at very low levels, with no patient presenting full resistance to second‐generation integrase inhibitors or darunavir in 2021.

#### P055 Phenotypic analysis of the impact of V106I in HIV‐1 reverse transcriptase on resistance to doravirine

F Saladini^1^, A de Salazar^2^, A Fuentes‐López^2^, L Viñuela^2^, F Giammarino^1^, N Bartolini^1^, C Charpentier^3^, S Lambert‐Niclot^4^, G Sterrantino^5^, G Colao^6^, V Micheli^7^, A Bertoli^8^, L Fabeni^9^, I Malet^10^, E Teyssou^10^, R Delgado^11^, I Falces‐Romero^12^, A Aguilera^13^, P Gomes^14^, D Paraskevis^15^, M Santoro^16^, A Marcelin^10^, F Ceccherini‐Silberstein^16^, M Zazzi^1^, F Garcia ^2^

^1^Department of Medical Biotechnologies, University of Siena, Siena, Italy; ^2^Clinical Microbiology, Hospital Universitario Clinico San Cecilio, Granada, Spain; ^3^Laboratoire de Virologie, Assistance Publique ‐ Hôpitaux de Paris, Hôpital Bichat‐Claude Bernard, Paris, France; ^4^Laboratoire de Virologie, Assistance Publique ‐ Hôpitaux de Paris, Hôpital Saint‐Antoine, Paris, France; ^5^Department of Clinical and Experimental Medicine, University of Florence, Florence, Italy; ^6^Laboratory of Virology, Careggi Hospital, Florence, Italy; ^7^Department of Clinical Microbiology, Virology and Bioemergencies, Sacco University Hospital, Milan, Italy; ^8^Microbiology and Virology Unit, University of Rome, Rome, Italy; ^9^Virology and Biosafety Laboratories Unit, Lazzaro Spallanzani ‐ IRCCS, National Institute for Infectious Diseases, Rome, Italy; ^10^Laboratoire de Virologie, Assistance Publique ‐ Hôpitaux de Paris, Hôpitaux Universitaires Pitié‐Salpêtrière ‐ Charles Foix, Paris, France; ^11^Clinical Microbiology Service, Hospital 12 de Octubre, Madrid, Spain; ^12^Clinical Microbiology Service, Hospital La Paz, Madrid, Spain; ^13^Clinical Microbiology Service, Complejo Hospitalario Santiago, Santiago de Compostela, Spain; ^14^Laboratório de Biología Molecular, LMCBM, SPC, Centro Hospitalar Lisboa Ocidental – HEM, Lisboa, Portugal; ^15^Department of Hygiene, Epidemiology and Medical Statistics, National and Kapodistrian University of Athens, Athens, Greece; ^16^Department of Experimental Medicine, University of Rome "Tor Vergata", Rome, Italy

**Aim**: To evaluate the impact of V106I mutation on phenotypic resistance to doravirine in the background of B and non‐B subtypes; in addition, we describe its prevalence in MeditRes HIV.

**Materials and methods**: MeditRes HIV is a consortium that includes ART‐naïve people living with HIV newly diagnosed in France, Greece, Italy, Portugal and Spain during the years 2018 to 2021. We evaluated the impact of V106I on susceptibility to doravirine (a) in site directed mutants containing V106I, V106A, V106M & Y188L mutations in subtype B (NL4.3, HXB2) and CRF02_AG background and (b) in a subset of recombinant viruses with clinically derived RT‐RNAseH coding region harbouring V106I and no other major NNRTI resistance associated mutations (RAMs). Phenotypic susceptibility to doravirine was determined through a TZM‐bl cell‐based assay and expressed as fold‐change (FC) with respect to the reference wild type virus.

**Results**: MeditRes HIV includes 2705 patients. The prevalence of V106I in the dataset was 2.85%, while for V106A and V106M was 0.015% and 0.14% resepctively. FC values for site directed mutants in the NL4.3, HXB2 and CRF02_AG background were 0.7, 2.0 and 2.5 with V106I, respectively; 3.4, 19.9 and NA (not available) with V106A; 9.4, 27.3 and 13.5 with V106M; >100, NA, and >100 with Y188L. The panel of clinically derived viruses tested so far includes 20 subtypes B and 15 non‐B subtypes (two A1, two CRF02_AG, three CRF06_cpx, one CRF44_BF, two D, four F1 and one URF). The median doravirine FC values were 1.5 (range 0.3 to 6.5) in the whole data set, 1.2 (range 0.3 to 1.9) for the B subtypes, and 2.45 (range 0.5 to 6.5) for non‐Bs; only three non‐B clinical isolates showed FC values higher than doravirine biological cutoff (3.0) (CRF06_cpx, FC=3.7; A1, FC=5.5; F1, FC=6.5).

**Conclusions**: Pretreatment drug resistance to doravirine through the years 2018 to 2021 remains low in the MeditRes HIV countries. Using site directed mutagenesis on a B and CRF02_AG background, there was no impact of V106I mutation on resistance to doravirine. Likewise, clinical isolates harbouring V106I and no other major NNRTI RAMs retained in vitro susceptibility to doravirine.

#### P056 High efficacy of dolutegravir/lamivudine (DTG/3TC) in treatment‐naive adults with HIV‐1 and high baseline viral load (VL): 48‐week subgroup analyses of the GEMINI‐1/‐2 and STAT trials

C Rolle ^1^, J Arribas^2^, R Ortiz^3^, J Matthews^4^, C Man^5^, R Grove^6^, C Donovan^7^, B Wynne^5^, M Kisare^8^, B Jones^9^

^1^Infectious Diseases, Orlando Immunology Center, Orlando, FL, USA; ^2^Infectious Diseases, Hospital Universitario La Paz, Madrid, Spain; ^3^Infectious Diseases, Bliss Healthcare Services, Orlando, FL, USA; ^4^Global Medical Affairs, ViiV Healthcare, Durham, NC, USA; ^5^Clinical Development, ViiV Healthcare, Durham, NC, USA; ^6^Statistics, GSK, Brentford, UK; ^7^Scientific Communication & Medical Education, ViiV Healthcare, Durham, NC, USA; ^8^Global Medical Affairs, GSK, Nairobi, Kenya; ^9^Global Medical Affairs, ViiV Healthcare, Brentford, UK

**Background**: Limited efficacy data for 2DRs versus 3DRs are available in treatment‐naive adults with HIV‐1 and high VL (≥500 000 copies/mL). DTG/3TC demonstrated high efficacy and a favorable safety profile in treatment‐naive adults in the GEMINI‐1/‐2 studies and the STAT test‐and‐treat study. Here we present 48‐week efficacy and safety for DTG/3TC in treatment‐naive participants in GEMINI‐1/‐2 and STAT by baseline VL.

**Materials and methods**: GEMINI‐1/‐2 are randomized (1:1) phase III studies of once‐daily DTG + 3TC versus DTG + TDF/FTC in treatment‐naive adults with screening HIV‐1 RNA ≤500 000 copies/mL and no major resistance‐associated mutations. STAT is a single‐arm study in treatment‐naive adults who initiated DTG/3TC ≤14 days after HIV‐1 diagnosis without availability of baseline laboratory results, with potential to modify ART based on baseline testing. Week 48 summaries included proportions with HIV‐1 RNA <50 and ≥50 copies/mL (Snapshot, ITT‐E), change from baseline in CD4+ cell count, and safety by baseline VL.

**Results**: Of 1433 GEMINI‐1/‐2 participants, 18% and 2% had baseline VL >100 000 to ≤500 000 and >500 000 copies/mL, respectively. Of 131 STAT participants, 24% and 15% had baseline VL >100 000 to ≤500 000 and >500 000 copies/mL, respectively; 8% had baseline VL >1 000 000 copies/mL. At week 48, proportions with HIV‐1 RNA <50 copies/mL were high across all studies, including in participants with high and very high baseline VL (Table 1). Few participants with baseline VL >500 000 copies/mL had HIV‐1 RNA ≥50 copies/mL (GEMINI‐1/‐2, n = 1 in the DTG + TDF/FTC group; STAT, n = 3). Mean increase from baseline to week 48 in CD4+ cell count was generally similar across baseline VL categories in GEMINI‐1/‐2 (DTG + 3TC, 218.0 to 247.2 cells/mm^3^; DTG + TDF/FTC, 210.9 to 278.3 cells/mm^3^) and STAT (239.4 to 539.5 cells/mm^3^). Incidence of drug‐related AEs was similar among participants with baseline VL ≤100 000 versus >100 000 copies/mL, respectively, in GEMINI‐1/‐2 (DTG + 3TC, 20% vs 23%; DTG + TDF/FTC, 27% vs 25%) and STAT (10% vs 10%); most were grade 1 or 2.

**Conclusions**: These data support the robust efficacy and safety of DTG/3TC as a first‐line regimen and in a test‐and‐treat setting in treatment‐naive adults with high baseline VL, with similarly high efficacy demonstrated between 2DRs and 3DRs.

**Table:** 

**Abstract P056 – Table 1**. Efficacy results at week 48 from the GEMINI‐1/‐2 and STAT studies by baseline viral load: ITT‐E populations.								
	≤100 000 copies/mL		>100 000 to ≤500 000 copies/mL		>500 000 to ≤1 000 000 copies/mL		>1 000 000 copies/mL	
	DTG + 3TC	DTG + TDF/FTC	DTG + 3TC	DTG + TDF/FTC	DTG + 3TC	DTG + TDF/FTC	DTG + 3TC	DTG + TDF/FTC
GEMINI‐1/‐2	(N = 576)	(N = 564)	(N = 127)	(N = 138)	(N = 11)	(N = 14)	(N = 2)	(N = 1)
HIV‐1 RNA <50 copies/mL	526 (91)	531 (94)	118 (93)	126 (91)	10 (91)	11 (79)	1 (50)^a^	1 (100)
HIV‐1 RNA ≥50 copies/mL	15 (3)	9 (2)	5 (4)	3 (2)	0	1 (7)	0	0
CD4+ cell count, cells/mm^3^								
Baseline, mean (SD)	488.0	484.8	364.2	387.4	289.2	276.1	142.0	27
	(219.6)	(213.7)	(186.2)	(185.2)	(98.7)	(166.6)	(169.7)	(NC)
Change from baseline, mean (SD) [n]	218.0	210.9	247.2	237.3	242.0	278.3	NR [n = 1]	NR [n = 1]
	(178.5)	(195.0)	(144.0)	(173.2)	(92.9)	(143.9)		
	[530]	[535]	[120]	[126]	[10]	[12]		
	≤100 000 copies/mL		>100 000 to ≤500 000 copies/mL		>500 000 to ≤1 000 000 copies/mL		>1 000 000 copies/mL	
	DTG/3TC		DTG/3TC		DTG/3TC		DTG/3TC	
STAT	(N = 79)		(N = 32)		(N = 9)		(N = 10)	
HIV‐1 RNA <50 copies/mL	60 (76)		23 (72)		8 (89)		8 (80)	
HIV‐1 RNA ≥50 copies/mL^b^	8 (10)		8 (25)		1 (11)		2 (20)	
CD4+ cell count, cells/mm^3^								
Baseline, mean (SD)	505.3 (302.6)		266.3 (169.9)		105.3 (102.2)		388.9 (221.9)	
Change from baseline, mean (SD) [n]	239.4 (219.9) [60]		260.5 (153.9) [26]		290.4 (183.8) [8]		539.5 (333.2) [8]	

^a^The other participant withdrew from the study due to physician decision and had no virologic data at week 48;

^b^non‐suppression at week 48 driven by study withdrawals (n = 6) and ART modifications (n = 10); three participants had data in window and HIV‐1 RNA ≥50 copies/mL (all in the >100 000 to ≤500 000 copies/mL VL category).

#### P057 Real‐world use of dolutegravir/lamivudine in treatment‐naïve people living with HIV during the COVID pandemic

G Pierone, Jr. ^1^, J Fusco^2^, L Brunet^2^, V Vannappagari^3^, S Sarkar^3^, C Henegar^3^, J van Wyk^4^, A Zolopa^5^, G Fusco^2^

^1^Medicine, Whole Family Health Center, Vero Beach, FL, USA; ^2^Sciences/Epidemiology, Epividian, Inc., Durham, NC, USA; ^3^Epidemiology and Real World Evidence, ViiV Healthcare, Research Triangle Park, NC, USA; ^4^Medical Affairs, ViiV Healthcare, London, UK; ^5^Medical Affairs, ViiV Healthcare, Research Triangle Park, NC, USA

**Background**: Fixed dose dolutegravir/lamivudine (DTG/3TC) two‐drug regimen (2DR) was first approved for use in antiretroviral treatment (ART)‐naïve people living with HIV (PLWHIV) in April 2019. Its uptake coincided with the COVID‐19 pandemic. We sought to compare DTG/3TC to typical three‐drug regimens (3DR) among ART‐naïve initiators.

**Materials and methods**: ART‐naïve PLWHIV initiating DTG/3TC 2DR (n = 360), bictegravir (BIC) 3DR (n = 3114), or DTG 3DR (n = 336) between 1 May 2019 and 30 April 2021 in the OPERA^®^ Cohort were followed until 31 October 2021 (potential for ≥6 months of follow‐up). Univariate Poisson regression (incidence rates) and Cox proportional hazards marginal structural models (hazard ratios) were used to assess the risk of virologic failure (VF; defined as two consecutive viral loads [VL] ≥200 copies/mL after 24 weeks on the regimen) and discontinuation (D/C; defined as a change in regimen or prescription gaps >45 days).

**Table:** 

**Abstract P057 – Table 1**. Population characteristics at baseline and virologic failure over follow‐up among ART‐naïve PLWHIV			
(N = 3810).			
Baseline characteristics	DTG/3TC 2DR, N = 360	BIC 3DR, N = 3114	DTG 3DR,^a^ N = 336
Median age (IQR)	30.7 (25.2 to 40.3)	31.8 (26.2 to 41.5)	34.1 (27.6 to 45.8)
Female, n (%)	48 (13)	444 (14)	63 (19)
Black race, n (%)	190 (53)	1848 (59)	214 (64)
Median viral load, log copies/mL (IQR)	4.7 (4.2 to 5.2)	4.8 (4.2 to 5.3)	4.7 (4.0 to 5.2)
CD4 cell count <200 cells/μL, n (%)	53 (15)	763 (24)	81 (24)
Comorbidities, n (%)	140 (39)	1221 (39)	183 (54)
Confirmed virologic failure^b^			
n (%)	8 (2)	97 (3)	15 (5)
IR per 100 py (95% CI)	1.74 (0.87 to 3.49)	2.25 (1.85 to 2.75)	3.55 (2.14 to 5.88)
Adjusted HR^c,d^ (95% CI)	Ref	1.12 (0.54 to 2.33)	2.30 (0.94 to 5.60)
Months on regimen, median (IQR)	15.0 (8.9 to 21.5)	15.8 (11.0 to 23.2)	14.6 (9.9 to 22.9)

2DR, two‐drug regimen; 3DR, three‐drug regimen; 3TC, lamivudine; ABC, abacavir; BIC, bictegravir; DTG, dolutegravir; FTC, emtricitabine; HR, hazard ratio; IQR, interquartile range; IR, incidence rate; py, person‐years; TAF, tenofovir alafenamide; TDF, tenofovir disoproxil fumarate.

^a^DTG 3DR includes DTG/ABC/3TC, DTG/TDF/FTC, and DTG/TAF/FTC;

^b^defined as two consecutive viral loads ≥200 copies/mL after 24 weeks;

^c^marginal structural model with inverse probability of treatment weights controlled for baseline age (quadratic), female, Black, Hispanic, Southern US, log10 viral load, CD4 cell count (quadratic);

^d^128 patients were excluded due to missing values (CD4).

**Results**: Characteristics of the 3810 ART‐naïve PLWHIV included are detailed in Table 1. Incidence rates of VF were low at 1.74 (DTG/3TC), 2.25 (BIC 3DR), and 3.55 (DTG 3DR) per 100 person‐years over a follow‐up of about 15 months for each group (Table 1). Compared to DTG/3TC, only DTG 3DR was associated with a numeric increase in the hazard of VF. D/C was high during the COVID pandemic with incidence rates of 8.50 (BIC 3DR), 14.91 (DTG/3TC), and 27.91 (DTG 3DR) per 100 person‐years. Prescription gaps >45 days in duration were common (n = 301). Nearly a third (97, 32%) of individuals with extended gaps went back on therapy prior to the end of the study period, often with the same regimen (6/17 DTG/3TC, 75/231 BIC 3DR, 12/53 DTG 3DR); median gap duration was 111 days (IQR 79 to 161). Regardless of regimen, most discontinuers were suppressed (VL <200 copies/mL) or not retested at the time of D/C (78% DTG/3TC, 78% BIC 3DR, 79% DTG 3DR).

**Conclusions**: Among ART‐naive PLWHIV, VF was uncommon with DTG/3TC 2DR, BIC 3DR or DTG 3DR. Many D/Cs were actually gaps in prescriptions, with nearly a third returning to the initial regimen. Such ART prescription patterns may be an anomaly of the COVID‐19 pandemic.

#### P058 BICNOW clinical trial: preliminary results of rapid test and treat BIC/FTC/TAF study in naive PLWHIV

C Hidalgo‐Tenorio ^1^, S Sequera^1^, A Collado^2^, M Vivancos Gallego^3^, I De los Santos^4^, P Sorni^5^, N Cabello^6^, M Montero‐Alonso^7^, A Terrón^8^, O Martinez^9^, M Omar^10^, P Ryan^11^, M Galindo^12^, J Pasquau^1^, R Javier^1^, M Lopez Ruz^1^, C Garcia Vallecillos^1^

^1^Infectious Diseases Unit, University Hospital Virgen de las Nieves, Granada, Spain; ^2^Infectious Diseases Unit, Hopsital Torrecárdenas, Almería, Spain; ^3^Infectious Diseases Service, Hospital Ramón y Cajal, Madrid, Spain; ^4^Infectious Diseases Unit, Centro de Investigación Biomédica en Red Enfermedades Infecciosas, Madrid, Spain; ^5^Infectious Diseases Unit, Hospital Son Llàtzer, Palma Mallorca, Spain; ^6^Infectious Diseases Service, Hospital Clinico San Carlos, Madrid, Spain; ^7^Infectious Diseases Service, University Hospital La Fe, Valencia, Spain; ^8^Infectious Diseases Unit, Hospital de Jerez, Jerez, Spain; ^9^Infectious Diseases Unit, University Hospital Santa Lucia, Cartagena, Spain; ^10^Infectious Diseases Unit, Hospital Jaen, Jaen, Spain; ^11^Infectious Diseases Unit, Hospital Infanta Leonor, Madrid, Spain; ^12^Infectious Diseases Service, University Hospital Valencia, Valencia, Spain

**Background**: The global HIV epidemic is still not under control, even in high‐income countries. To reduce the number of new diagnoses, several strategies have been implemented, including pre‐exposure prophylaxis (PrEP) or rapid initiation (test and treat) of antiretroviral therapy (ART). The single‐tablet regimen BIC/FTC/TAF is an ideal drug for rapid initiation. We present the preliminary data (24 weeks(w)) from the BICNOW trial.

**Materials and methods**: In this phase IV, multi‐centre, open label, single‐arm, 48‐week study, adult participants were enrolled from December 2020 to June 2022 with follow‐up through 48w at baseline, 4, 24, and 48w. Adherence to treatment was assessed using the SMAQ questionnaire. The EudraCT number: 2019‐003251‐11.

**Results**: One hundred and sixty participants were included with a mean age of 35.7 years; 90% were male, with mean CD4 count of 395.2 cells/uL (22.2% had CD4 count <200 cells/uL), mean viral load (VL) of 5.6 log_10_ copies/mL (46.8% VL >100 000 copies/mL), and 23.1% had CDC‐defined AIDS. 0.6% had HBV coinfection. BIC/FTC/TAF was initiated in 98.8% of the subjects on the day they first attended the HIV specialist's office without baseline laboratory results. After 4w of ART, 56.9% had VL <50 copies/mL, and 77.8% had VL <200 copies/mL; at 24w 88.8% had VL <50 copies/mL, and 100% had VL <90 copies/mL. From 4w to 24w, mean CD4 count increased from 395.2 to 589.8 cells/uL (p = 0.0001), and VL decreased from 5.6 to 1.57 log_10_ copies/mL (p = 0.0001). Weight, body mass index and abdominal circumference increased (73.5 kg (IQR 65.7 to 83) vs 76 (IQR 67.8 to 87.1) [p = 0.0001]; 24.3 (IQR 21.5 to 26.3) vs 25 (IQR 23 to 27) [p = 0.0001]; 85.7 vs 87.8 cm [p = 0.001], respectively). There was an increase in total and HDL cholesterol levels (159.3 to 168.5 mg/dL [p = 0.006], 41.2 to 47.2 mg/dL [p = 0.0001], respectively), and the TC/HDL ratio was reduced (4.74 vs 3.9 [p = 0.013]). Triglycerides and mean LDLc did not change (106.2 to 109.5 mg/dL [p = 0.9] and 107.5 to 107.8 mg/dL [p = 0.053]). At week 4 and 24, no patients had adverse events and forgetfulness.

**Conclusion**: BIC/FTC/TAF is a suitable option for the rapid initiation of ART in naïve individuals infected with HIV. Treatment was associated with a rapid reduction in viral load, significant increase in CD4 and small increase in weight, BMI, abdominal circumference, with TC/HDL ratio decreased.

#### P059 96 weeks effectiveness and tolerability of DTG+3TC in naive patients: the REDOLA study

F Pulido^1^, J López Bernaldo de Quirós^2^, M Górgolas^3^, M Torralba^4^, L Martín‐Carbonero^5^, Á Mena^6^, J Sanz^7^, J Vergas^8^, Á Gutiérrez ^7^, M Hernández‐Segurado^3^, E Palmier^5^, F Tejerina^2^, A Pinto^1^, M Téllez^8^, P Vázquez^6^, A Cabello^3^

^1^HIV Unit, 12 de Octubre University Hospital, Imas12, Madrid, Spain; ^2^Infectious Diseases, Gregorio Marañón University Hospital, Madrid, Spain; ^3^Infectious Diseases, Fundación Jiménez Díaz University Hospital, Madrid, Spain; ^4^Infectious Diseases, Guadalajara University Hospital, Guadalajara, Spain; ^5^Infectious Diseases, La Paz University Hospital, Madrid, Spain; ^6^Infectious Diseases, A Coruña University Hospital, A Coruña, Spain; ^7^Infectious Diseases, La Princesa University Hospital, Madrid, Spain; ^8^Infectious Diseases, Clínico San Carlos University Hospital, Madrid, Spain

**Background**: DTG/3TC therapy is a preferred regimen for people living with HIV (PLWHIV) in international guidelines, due to the efficacy observed in clinical trials. However, information in real‐life cohorts is still scarce.

**Materials and methods**: Multicentre retrospective and prospective cohort study of ART‐naïve PLWHIV starting DTG/3TC as first‐line regimen before 31 March 2020. Confirmed virological failure (CVF): two consecutive plasma HIV‐RNA ≥50 copies/mL.

**Results**: One hundred and eighty‐five patients. Baseline characteristics in Table 1. Treatment was started without the baseline drug resistance testing (bDRT) results in 132 (71.4%). Resistance to 3TC in bDRT was found in 2/185 (1.1%). In the intention‐to‐treat (ITT) analysis (missing=failure), effectiveness at week 96 was 83.8% (155/185), and in the per‐protocol (PP) analysis (discontinuations not related with therapy excluded) was 95.7% (155/162). One patient had CVF at week 96 (79 and 365 copies/mL) and continues DTG/3TC. Eleven patients (5.9%) discontinued treatment: three due to poor adherence, with a single with HIV‐RNA ≥50 copies/mL and no emerging resistance; three due to CNS side effects (1.6%); two after receiving bDRT (M184V mutation); one due to an extrapulmonary tuberculosis (IRIS) and another two to be included in a clinical trial. Finally, 18 patients (9.7%) were lost to follow‐up. The results (ITT) in the stratified analysis by baseline HIV‐1 viral load were: ≥100 000 copies/mL 86.7% versus 82.9% <100 000 copies/mL; Dif ‐3.8 [95% CI ‐13.8 to 10.1], by CD4+: <200 cells/mm^3^ 70% versus 84.6% ≥200 cells/mm^3^; Dif 14.6 [95% CI ‐5.6 to 45.2] and by availability of bDRT before starting therapy: no‐bDRT 84.1% versus 83% bDRT; Dif ‐1.1 [95% CI: ‐14.4 to 9.5]. There were no significant changes in the lipid profile. The mean weight gain in a subgroup of patients (N = 70) was 2.6±5.6 kg.

**Table:** 

**Abstract P059 – Table 1**. Subjects’ baseline characteristics.				
Baseline characteristics			Patients (p) (N = 185)	
Age (years) (median ‐ IQR)			33 (27‐41)	
Gender	Male		168 (90.8%)	
Gender	Female		16 (8.7%)	
Gender	Transgender woman		1 (0.5%)	
HIV transmission	MSM		156 (84.3%)	
HIV transmission	MSW		26 (14.1%)	
HIV transmission	IDU		3 (1.6%)	
Country ‐ region	Spain		94 (50.8%)	
Country ‐ region	Latin American		77 (41.6%)	
Country ‐ region	Europe		6 (3.3%)	
Country ‐ region	Others		7 (4.3%)	
CD4 cells/mm^3^ (median ‐ IQR)	Basal CD4+		446 (342‐608)	
CD4 cells/mm^3^ (median ‐ IQR)	CD4 < 200		10 (5.4%)	
HIV‐1 VL (c/mL)	≥ 100,000 c/mL		45 (24.3%)	
HIV‐1 VL (c/mL)	< 100,000 c/mL		140 (75.7%)	
Hepatitis B co‐infection	HBsAg +		0%	
Hepatitis B co‐infection	Anti‐HBc +		41 (22.2%)	
Hepatitis B co‐infection	Anti‐HBs +		121 (65.4%)	
Hepatitis C co‐infection (IgG HCV +)			8 (4.3%)	
HLAB5701 positive			15 (8.1%)	
Median time from diagnosis to start of treatment (weeks)			6 (IQR: 2‐12)	
Major mutations in Baseline drug resistance testing (bDRT)	22 p (11.9%)	INSTIs	1 p (0.5%)	G163K
Major mutations in Baseline drug resistance testing (bDRT)	22 p (11.9%)	PIs	1 p (0.5%)	V82C
Major mutations in Baseline drug resistance testing (bDRT)	22 p (11.9%)	NNRTIs	17 p (9.2%)	E138A(5), K103N(8), V1061(3), V108Vi(2), E138EG, E138E, G190A(2)
Major mutations in Baseline drug resistance testing (bDRT)	22 p (11.9%)	NRTIs	5 p (2.7%)	M184V(2), M14L, E44D, T215D, T215C, 210W, 215S

IQR, interquartile range; MSM, men who have sex with men; MSW, men who have sex with women; IDU, injecting drug users; INSTIs, integrase strand transfer inhibitors; PIs, protease inhibitors; NNRTIs, non‐nucleoside reverse transcriptase inhibitors; NRTIs, nucleoside reverse transcriptase inhibitors.

**Conclusions**: In a real‐life multicentre cohort of ART‐naïve PLWHIV, treatment initiation with DTG/3TC showed high effectiveness and tolerability, without treatment‐emergent resistance through 96 weeks. Starting treatment without knowing results of the baseline drug resistance test did not have an impact on the effectiveness of the regimen.

#### P060 Effectiveness of bictegravir/emtricitabine/tenofovir alafenamide (BTC/FTC/TAF) in ART‐naive patients: real‐world data from the ICONA cohort

A Tavelli ^1^, G Marchetti^2^, A Vergori^3^, E Quiros‐Roldan^4^, V Malagnino^5^, F Vichi^6^, M Lichtner^7^, S Nozza^8^, R Rossotti^9^, L Sarmati^5^, F Bai^2^, A Di Biagio^10^, A Antinori^3^, A d'Arminio Monforte^2^

^1^Icona Foundation, Milan, Italy; ^2^Department of Health Sciences, Clinic of Infectious Diseases, ASST Santi Paolo e Carlo, University of Milan, Milan, Italy; ^3^Clinical Department, HIV/AIDS Unit, National Institute for Infectious Diseases Lazzaro Spallanzani IRCCS, Rome, Italy; ^4^Department of Infectious and Tropical Diseases, University of Brescia, ASST Spedali Civili of Brescia, Brescia, Italy; ^5^Unit of Clinical Infectious Disease, Department of System Medicine, Tor Vergata University, Policlinico Tor Vergata, Rome, Italy; ^6^Infectious Diseases Unit 1, Santa Maria Annunziata Hospital, Azienda USL Toscana Centro, Florence, Italy; ^7^Infectious Diseases Unit, Santa Maria Goretti Hospital, Sapienza University of Rome, Latina, Italy; ^8^Infectious Diseases Unit, IRCCS San Raffaele Scientific Institute, Milan, Italy; ^9^Department of Infectious Diseases Unit, ASST Grande Ospedale Metropolitano Niguarda, Milan, Italy; ^10^Department of Health Sciences, Unit of Infectious Diseases, Ospedale Policlinico San Martino IRCCS, University of Genoa, Genoa, Italy

**Background**: Use of BIC/FTC/TAF is based on several pivotal trials, but real‐life data, especially from key‐populations, are still lacking. The aim of this study is to evaluate the effectiveness of BIC/FTC/TAF in ART‐naïve people living with HIV (PLWHIV).

**Materials and methods**: Observational study including ART‐naïve PLWHIV from Icona cohort who started BIC/FTC/TAF from June 2016 to December 2021. Primary objective: treatment failure (TF) i.e. virological failure (VF: two HIV‐RNA >200 copies/mL or one >1000 copies/mL >6 months from start) or treatment discontinuation (TD) for any reason. Secondary objectives: TD for any reason; TD for toxicity/intolerance; VF; change in CD4, CD4/CD8 and weight at 12 (±3) months from start. Standard survival analysis (Kaplan–Meier curves and log‐rank test) were used. Unadjusted and adjusted hazard ratios (HR) of TF by Cox regression models for different groups: ≥50 years old; female; late presenters (LP, CD4 <350 cells/mm^3^ or AIDS) and advanced HIV disease (AD, CD4 <200 cells/mm^3^ or AIDS). Paired t‐tests to evaluate mean changes at 1 year.

**Table:** 

**Abstract P060 – Table 1**. Baseline demographic and clinical characteristics of the 416 PLWHIV starting BIC/FTC/TAF in the Icona cohort.		
	ART‐naive (N=416)	
Italian, n (%)	239	(70.43)
Ethnicity, Caucasian, n (%)	329	(79.1)
Gender, female, n (%)	73	(17.5)
Year of BIC start, median (IQR)	2020	(2020‐2021)
Year ART start, median (IQR)	2020	(2020‐2021)
Age, years, median (IQR)	42	(32‐52)
>50 years, n (%)	124	(29.81)
Mode of HIV transmission, n (%)		
Heterosexual	172	(41.35)
IVDU	19	(4.57)
MSM	188	(45.19)
Other/Unknown	37	(8.89)
HCVAb positive status, n (%)	20	(4.81)
HBsAg positive status, n (%)	11	(2.64)
AIDS, n (%)	59	(14.2)
CD4, cells/mmc, median (IQR)	280	(87‐495)
CD4<200 cells/mmc, n (%)	165	(39.7)
CD4<350 cells/mmc, n (%)	241	(57.9)
CD4/CD8 ratio, median (IQR)	0.33	(0.14‐0.58)
HIV‐RNA, log10 copies/mL, median (IQR)	5.02	(4.39‐5.60)
LDL cholesterol, mg/dL, median (IQR)	102	(80‐124)
HDL cholesterol, mg/dL, median (IQR)	40	(33‐49)
Triglycerides, mg/dL, median (IQR)	94	(71‐139)
Serum glucose, mg/dL, median (IQR)	87	(80‐93)
eGFR, CKD‐EPI, ml/min, median (IQR)	106.4	(92.2‐117.5)
Weight, kg, median (IQR)	70	(60‐78)
BMI, kg/m^2^, median (IQR)	23	(20.6‐24.7)
Follow‐up on BIC, years, median (IQR)	0.83	(0.44‐1.22)

**Results**: Four hundred and sixteen ART‐naïve (17.5% female, 29.8% ≥50 years, 58.2% LP, 40.6% AD). Patients’ characteristics in Table 1. Over a median follow‐up of 0.9 years (IQR 0.4 to 1.2), 51 TF occurred (12.2%, seven VF and 44 TD). The 1‐year probability of TF was 11.0% (95% CI 7.9 to 15.1), TF probabilities by subgroups are shown in Figure 1A. In the Cox regression models adjusted for confounders, none of the groups analysed had a higher risk of TF (Figure 1B). Forty‐five TD (10.8%): 16 toxicity/intolerance (3.8%), 15 simplification (3.6%), four failure (1.0%), one patient's decision (0.2%) and nine other reasons (2.2%). Seven VF occurred (2.6%). Figure 1A shows the 1‐year probabilities of TD for any reason, toxicity/intolerance and VF. Mean CD4 increase at 1 year was +244 cells/mm^3^ (95% CI 216 to 271, p < 0.001), mean CD4/CD8 change was +0.09% (‐15.2 to 0.32, p = 0.476). Weight change among 43 PLWHIV included was +5.2kg (3.3 to 6.9, p < 0.001) with greater increase for AD +7.2 kg (4.3 to 10.0, p < 0.001).

**Figure:**
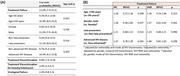
**Abstract P060 – Figure 1**. (A) Kaplan‐Meier estimated 1‐year probability of TF, TD for any reason, TD toxicity/intolerance and VF, overall and in the different groups for primary endpoint; (B) hazard ratios (HR) and adjusted hazard ratios (AHR) of TF from fitting different Cox regression models in the different subgroups.

**Conclusions**: BIC/FTC/TAF demonstrated high effectiveness in a real world setting (at 1‐year 11.0% TF, 2.1% VF and +244 cells CD4/mmc). Also confirmed, in populations usually at risk of lower response (LP and AD). Discontinuation was mainly driven by toxicity/intolerance and simplification.

### Treatment Strategies ‐ Target Populations: Experienced Patients

#### P061 Efficacy and safety of fostemsavir plus optimized background therapy in heavily treatment‐experienced adults with HIV‐1: week 240 results of the phase III BRIGHTE study

J Aberg ^1^, B Shepherd^2^, M Wang^3^, J Madruga^4^, F Mendo Urbina^5^, C Katlama^6^, S Schrader^7^, J Eron^8^, S Chabria^9^, A Clark^10^, A Pierce^11^, M Lataillade^12^, P Ackerman^9^

^1^Infectious Diseases, Icahn School of Medicine at Mount Sinai, New York, NY, USA; ^2^Clinical Safety & Pharmacovigilance, GSK, Brentford, UK; ^3^Statistics, GSK, Upper Providence, PA, USA; ^4^Infectious Diseases, Centro de Referência e Treinamento DST / AIDS SP, São Paulo, Brazil; ^5^Infectious Diseases, Hospital Nacional Edgardo Rebagliati Martins, Lima, Peru; ^6^Infectious Diseases, AP‐HP, Hôpital Pitié‐Salpêtrière, Service de Maladies Infectieuses et Tropicales, INSERM‐Sorbonne Universités, Paris, France; ^7^Infectious Diseases, Schrader Clinic, Houston, TX, USA; ^8^Infectious Diseases, University of North Carolina at Chapel Hill School of Medicine, Chapel Hill, NC, USA; ^9^Clinical Development, ViiV Healthcare, Branford, CT, USA; ^10^Global Medical Affairs, ViiV Healthcare, Brentford, UK; ^11^Clinical Development, ViiV Healthcare, Research Triangle Park, NC, USA; ^12^Global Research Strategy, ViiV Healthcare, Branford, CT, USA

**Background**: In the ongoing phase III BRIGHTE study, fostemsavir plus optimized background therapy (OBT) demonstrated durable virologic suppression through 96 weeks in heavily treatment‐experienced (HTE) adults with HIV‐1.

**Figure:**
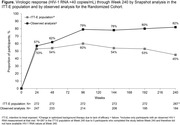
**Abstract P061 – Figure 1**. Virologic response (HIV‐1 RNA <40 copies/mL) through week 240 by Snapshot analysis in the ITT‐E population and by observed analysis for the randomized cohort.

**Materials and methods**: In the randomized cohort (RC), participants with fully active agents in one or two remaining antiretroviral classes received fostemsavir 600 mg twice daily or placebo for 8 days followed by open‐label fostemsavir plus OBT. In the non‐randomized cohort (NRC), participants with no approved fully active agents received fostemsavir 600 mg twice daily plus OBT. Week 240 assessments included virologic outcomes (Snapshot [intention‐to‐treat exposed] and observed analyses), CD4+ cell count, and safety.

**Results**: Of 371 participants enrolled, 71% (193/272) in the RC and 55% (54/99) in the NRC were ongoing at week 240. In the RC, virologic response rates generally remained consistent over time through week 240 (Figure 1). Lower virologic suppression by Snapshot analysis beyond week 192 was partially confounded by missing data due to COVID‐19. By observed analysis, mean (SD) CD4+ cell counts steadily increased from baseline (n = 272) over time and increased by 296 (228) cells/mm^3^ by week 240 (n = 139); mean CD4+/CD8+ ratio increased from 0.20 to 0.60. In the RC, 78% (73/94) of participants with baseline CD4+ count <200 cells/mm^3^ had CD4+ counts increase to ≥200 cells/mm^3^. Consistent with earlier findings across the RC and NRC, the most common drug‐related adverse events (AEs) were nausea (35/371, 9%) and diarrhea (18/371, 5%). Grade 2 to 4 drug‐related AEs were reported in 24% (88/371) of participants; 8% (30/371) reported AEs leading to discontinuation. No reported deaths (RC: 15/272, 6%; NRC: 20/99, 20%) were due to COVID‐19.

**Conclusions**: HTE participants treated through ∼5 years with optimized fostemsavir‐based regimens demonstrated durable virologic responses, continued clinically meaningful improvements in CD4+ cell count, and favorable safety. Although COVID‐19 impacted Snapshot virologic response rates, overall observed rates remained high (≥80%).

#### P062 Meta‐analysis of efficacy for DTG versus PI/r as second‐line treatment in four randomised trials, 2662 participants

M Mirchandani^1^, A Qavi^1^, B Simmons^2^, A Hill ^3^

^1^Faculty of Medicine, Imperial College London, London, UK; ^2^LSE Health, London School of Economics and Political Science, London, UK; ^3^Department of Pharmacology and Therapeutics, University of Liverpool, Liverpool, UK

**Background**: For second‐line treatment, WHO recommends either dolutegravir (DTG) or boosted protease inhibitors (PI/r) with optimised nucleoside reverse transcriptase inhibitors (NRTIs). Disadvantages of PIs include drug interactions, multi‐pill dosing, adverse events and higher costs. However, the genetic barrier to resistance is high for PIs. This analysis compares the efficacy of DTG and PI/r as second‐line regimens for HIV.

**Figure:**
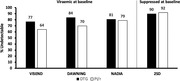
**Abstract P062 – Figure 1**. HIV RNA <50 at week 48.

**Methods**: Data on HIV RNA was included from four randomised trials: VISEND (n = 783), DAWNING (n = 624), NADIA (n = 464) and 2SD (n = 791) which recruited NNRTI‐experienced patients given second‐line treatment with either DTG or a PI/r. Data on HIV RNA suppression <50 and <1000 copies/mL from each study was extracted using patient‐level databases or publications. The meta‐analysis was conducted using RevMan Software (version 5.3). The risk differences (RD) for HIV RNA suppression were calculated using the Cochrane Mantel‐Haenszel test (random‐effects model). The non‐inferiority margin was ‐10%.

**Results**: The VISEND trial (Zambia), DAWNING (international) and NADIA (sub‐Saharan Africa) recruited patients with HIV RNA >1000 copies/mL at baseline. 2SD (Kenya) recruited patients stable on PIs with undetectable HIV RNA. At week 48, for patients viraemic at baseline, there was a significant difference between the DTG and PI/r arms in the number of patients with RNA <50 (RD +10%, 95% CI +2% to +17%, p = 0.01) (Figure 1). However, for patients already suppressed by PI/r at baseline, there was no significant difference between the DTG and PI/r arms (RD ‐1%, 95% CI ‐5% to +3%, p = 0.47). Similarly, there was no significant difference between the arms in the number of patients with RNA <1000 at week 48 (RD +5%, 95% CI ‐7% to +16%, p = 0.43). At week 96, there was a significant difference between the DTG and PI/r arms in the number of patients with RNA <50 (RD +11%, 95% CI +1% to +21%, p = 0.04) but no significant difference for patients with RNA <1000 (RD +10%, 95% CI ‐6% to +26%, p = 0.22).

**Conclusions**: In this meta‐analysis of 2662 participants evaluated in four randomised trials, DTG showed a superior rate of HIV RNA suppression <50 copies/mL in the primary analysis at week 48. In the sensitivity analyses, DTG was non‐inferior to PI/r.

#### P063 The impact of triggers of changes in sexual behaviour among persons living with HIV: Swiss Statement (U=U) and Covid‐19 compared

K Hamusonde ^1^, D Nicca^2^, H Günthard^3^, M Stöckle^4^, K Darling^5^, A Calmy^6^, E Bernasconi^7^, D Haerry^8^, R Kouyos^3^, A Rauch^1^, L Salazar‐Vizcaya^1^

^1^Infectious Diseases, University Hospital of Bern, Bern, Switzerland; ^2^Infectious Diseases, University of Basel, Basel, Switzerland; ^3^Infectious Diseases, University Hospital Zurich, Zurich, Switzerland; ^4^Infectious Diseases, University Hospital Basel, Basel, Switzerland; ^5^Infectious Diseases, University of Lausanne, Lausanne, Switzerland; ^6^Infectious Diseases, University of Geneva, Geneva, Switzerland; ^7^Infectious Diseases, Regional Hospital Lugano, Lugano, Switzerland; ^8^Positive Council, Positivrat, Bern, Switzerland

**Background**: Sexual behaviour differs across sexual preferences and adapts to changing factors intrinsic and external to the individual. We studied 22 years of self‐reported sexual behaviour among persons living with HIV in the Swiss HIV cohort study and short‐term changes following two external triggers likely to influence sexual behaviour: The Swiss Statement and the Covid‐19 Social Distancing (SD).

**Figure:**
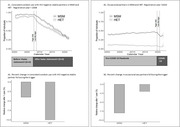
**Abstract P063 – Figure A1**. Proportion of persons reporting consistent condom use with HIV negative stable partners between MSM and HET registered in the SHCS before 2008. **Figure A2**. Percent change in consistent condom use with HIV negative stable partners following the trigger. **Figure B1**. Proportion of persons reporting occasional partners between MSM and HET registered in the SHCS before 2019. **Figure B2**. Percent change in occasional partners following the trigger.

**Materials and methods**: Data on sexual behaviour between 2000 and 2022 was recorded bi‐annually from registration. Persons with at least one follow‐up visit were included, and all analyses stratified by sexual preference: men who have sex with men (MSM) and heterosexuals (HET). We estimated trends in condom use after the Swiss Statement and in occasional partnership after the onset of Covid‐19 SD. We focused on consistent condom use with HIV‐negative stable partners among persons registered before the Swiss Statement (year: 2008), and occasional partnership among persons registered before Covid‐19 SD (year: 2020). We calculated short‐term changes following the respective triggers.

**Results**: Analyses for consistent condom use with stable partners included 4629 (MSM = 2212 and HET = 2417) PLWHIV while the frequency of occasional partners included 11 973 (MSM = 5617 and HET = 6356) PLWHIV. Between the Swiss Statement in 2008 and 2021, consistent condom use with stable partners declined from 94% to 28% in MSM and from 88% to 30% in HET (Figure A1). One year after the Swiss Statement, we observed declines in consistent condom use with stable partners of 4% among MSM and 10% among HET (Figure A2). Occasional partnership declined rapidly among MSM following Covid‐19 SD, but remained considerably higher in MSM (37%) than in HET (6%) (Figure B1). The speed of short‐term behavioural change differed between MSM and HET (Figure B2).

**Conclusion**: Condom use with stable partners has steadily declined from over 90% to 29% in the Swiss HIV Cohort Study (SHCS) since the Swiss Statement with similar trajectories between MSM and HET, while occasional partnership remained higher among MSM than among HET even during Covid‐19 SD. Our results suggest that the speed of adaptation to external triggers varies between MSM and HET.

#### P064 A multivariate analysis of the phase III BRIGHTE trial, through week 24, to identify predictors of virologic response to fostemsavir in heavily treatment‐experienced people living with HIV

M Gartland ^1^, X Wang^2^, Q Liao^2^, B Li^3^, F Du^3^, S Chabria^4^, M Krystal^4^, A Clark^5^, M Lataillade^6^, A Tenorio^1^

^1^Clinical Development, ViiV Healthcare, Durham, NC, USA; ^2^Statistics, ViiV Healthcare, Durham, NC, USA; ^3^Statistics, GSK, Collegeville, PA, USA; ^4^Clinical Development, ViiV Healthcare, Branford, CT, USA; ^5^Global Medical Affairs, ViiV Healthcare, Brentford, UK; ^6^Global Research Strategy, ViiV Healthcare, Branford, CT, USA

**Background**: Efficacy and safety of fostemsavir (Rukobia) in heavily treatment‐experienced individuals were demonstrated in the phase III BRIGHTE trial. Here, factors associated with virologic outcomes were evaluated post hoc.

**Materials and methods**: Data from 272 randomized and 99 non‐randomized cohort adults enrolled in BRIGHTE were evaluated in a multivariable analysis to examine the influence of baseline viral and participant factors and drug concentration on change from baseline in log10HIV‐1 RNA at day 8 for the randomized cohort, and virologic outcome (HIV‐1 RNA <40 copies/mL by Snapshot) at week 24 for both cohorts, using multiple linear and logistic regression models, with stepwise selection. Baseline factors identified from modeling were explored for association with protocol‐defined virologic failure (PDVF) with emergent phenotypic or genotypic changes to FTR or optimized background therapy (OBT).

**Results**: In randomized participants, baseline CD4+ cell count, baseline log10HIV‐1 RNA, presence of relevant gp120 substitutions (S375H/I/M/N/Y or M426L), and temsavir day 8 trough concentration (C_tau_) were significantly associated (p < 0.05) with decrease in HIV‐1 RNA >0.5log10 at day 8. Factors significantly associated with virologic response <40 copies/mL at week 24 in randomized participants were baseline log10HIV‐1 RNA and in non‐randomized participants were baseline log10HIV‐1 RNA, inhibitory quotient (week 24 C_tau_/protein‐binding‐adjusted temsavir IC50), and overall susceptibility score of OBT. Among evaluable participants through week 24, 8% (22/263) of randomized and 25% (24/95) of non‐randomized cohort participants experienced PDVF with emergent phenotypic or genotypic changes to FTR or OBT. Incidence of PDVF with emergent changes was similar among participants with 0, 1, or ≥2 baseline factors, including CD4+ cell count <20 cells/mm^3^, HIV‐1 RNA ≥100 000 copies/mL, and presence of relevant gp120 substitutions.

**Conclusions**: Virologic response to FTR functional monotherapy or treatment with FTR plus OBT in randomized participants was significantly associated with well‐established factors such as baseline CD4+ cell count, baseline log10HIV‐1 RNA, and drug concentration. Relevant baseline gp120 substitutions were significantly associated with reduced response to FTR functional monotherapy but not with virologic outcome to FTR plus OBT over 24 weeks. Overall, the presence of baseline factors associated with virologic response to FTR was not predictive of PDVF in this patient population.

#### P065 Characterisation and outcomes of difficult‐to‐treat patients in an Italian cohort of PLWHIV starting modern ART regimens

R Gagliardini ^1^, A Tavelli^2^, S Rusconi^3^, S Lo Caputo^4^, V Spagnuolo^5^, M Santoro^6^, A Costantini^7^, S Cicalini^1^, F Maggiolo^8^, A Giacomelli^9^, G Burastero^10^, C Agrati^11^, G Madeddu^12^, E Quiros‐Roldan^13^, A d'Arminio Monforte^14^, A Antinori^1^, A Cozzi‐Lepri^15^

^1^UOC Immunodeficienze Virali, INMI L. Spallanzani IRCCS, Roma, Italy; ^2^Icona, Icona Foundation, Milan, Italy; ^3^Unità Operativa Malattie Infettive, Ospedale Civile di Legnano, Legnano, Italy; ^4^Infectious Diseases Unit, University of Foggia, Foggia, Italy; ^5^Infectious Diseases Unit, IRCCS San Raffaele Scientific Institute, Milan, Italy; ^6^Department of Experimental Medicine, University of Rome Tor Vergata, Rome, Italy; ^7^Clinical Immunology Unit, Azienda Ospedaliero‐Universitaria Ospedali Riuniti, Marche Polytechnic University, Ancona, Italy; ^8^Infectious Diseases Unit, ASST Papa Giovanni XXIII, Bergamo, Italy; ^9^III Infectious Diseases Unit, ASST Fatebenefratelli Sacco, Ospedale Luigi Sacco, Milano, Italy; ^10^Infectious Disease Clinic, Azienda Ospedaliero‐Universitaria di Modena, Modena, Italy; ^11^Cellular Immunology and Pharmacology Unit, INMI L. Spallanzani IRCCS, Roma, Italy; ^12^Department of Medical, Surgical, and Experimental Sciences, University of Sassari, Sassari, Italy; ^13^Department of Infectious and Tropical Diseases, University of Brescia and ASST Spedali Civili of Brescia, Brescia, Italy; ^14^Department of Health Sciences, University of Milan, Milano, Italy; ^15^Centre for Clinical Research, Epidemiology, Modelling and Evaluation (CREME), Institute for Global Health, UCL, London, UK

**Background**: Treatment failures to modern ART regimens are of concern, as they might limit future drug options leading to clinical failure. Real‐world estimates of the rate of multiple failures to modern regimens are lacking and long‐term consequences remain unclear.

**Material and methods**: Participants of the ICONA cohort who started a modern first‐line ART (2NRTI+DRV/b; 2NRTI+INSTI; 2NRTI+RPV; 2NRTI+DOR; DTG+3TC) were included. Patients were classified as 'difficult to treat' (DTT) if, after starting ART, experienced ≥1 of these events: i) ≥2 VF (VF defined as two consecutive viral load (VL) >50 copies/mL) with or without subsequent ART change; ii) ≥2 treatment discontinuations (TD) due to toxicity/intolerance/failure; iii) ≥1 VF followed by ART change plus ≥1 TD due to toxicity/intolerance/failure. Time to first fulfilling the DTT definition was estimated using the Kaplan‐Meier (KM). We then identified PLWHIV who, after the same time from starting ART, were still free from DTT events. In a subset of these who subsequently initiated a new regimen, we compared the treatment response between DTT and matched unexposed analysing the following endpoints: a) VF; b) TD due to intolerance/toxicity/failure; c) treatment failure (composite of VL >200 copies/mL or TD for intolerance/toxicity/failure); and d) clinical failure [AIDS/death, SNAE (serious non‐AIDS event)/death]. Survival analysis by means of KM curves and Cox regression models were employed.

**Results**: Among 8061 PLWHIV included, 320 (4%) entered in the DTT definition. KM probability of becoming DTT was 6.5% (5.8 to 7.4%) by 6 years. DTT PLWHIV had a significantly higher prevalence of AIDS diagnosis, were slightly older, had lower nadir of CD4, had higher VL at ART starting, when compared to the non‐DTT PLWHIV (Table 1). In unadjusted analyses and compared to the matched unexposed group (286 DTT and 572 matched‐unexposed), DTT showed higher probabilities of experiencing all the outcomes. After controlling for confounders, the associations remained significant for VF, treatment failure, SNAE/death (Figure 1). 

**Conclusions**: A total of 6.5% of PLWHIV who started a modern first‐line ART satisfied our arbitrary definition of DTT by 6 years from ART initiation. This appears to be a more vulnerable PLWHIV population who may experience a higher risk of treatment and clinical failure in the long term.

**Figure:**
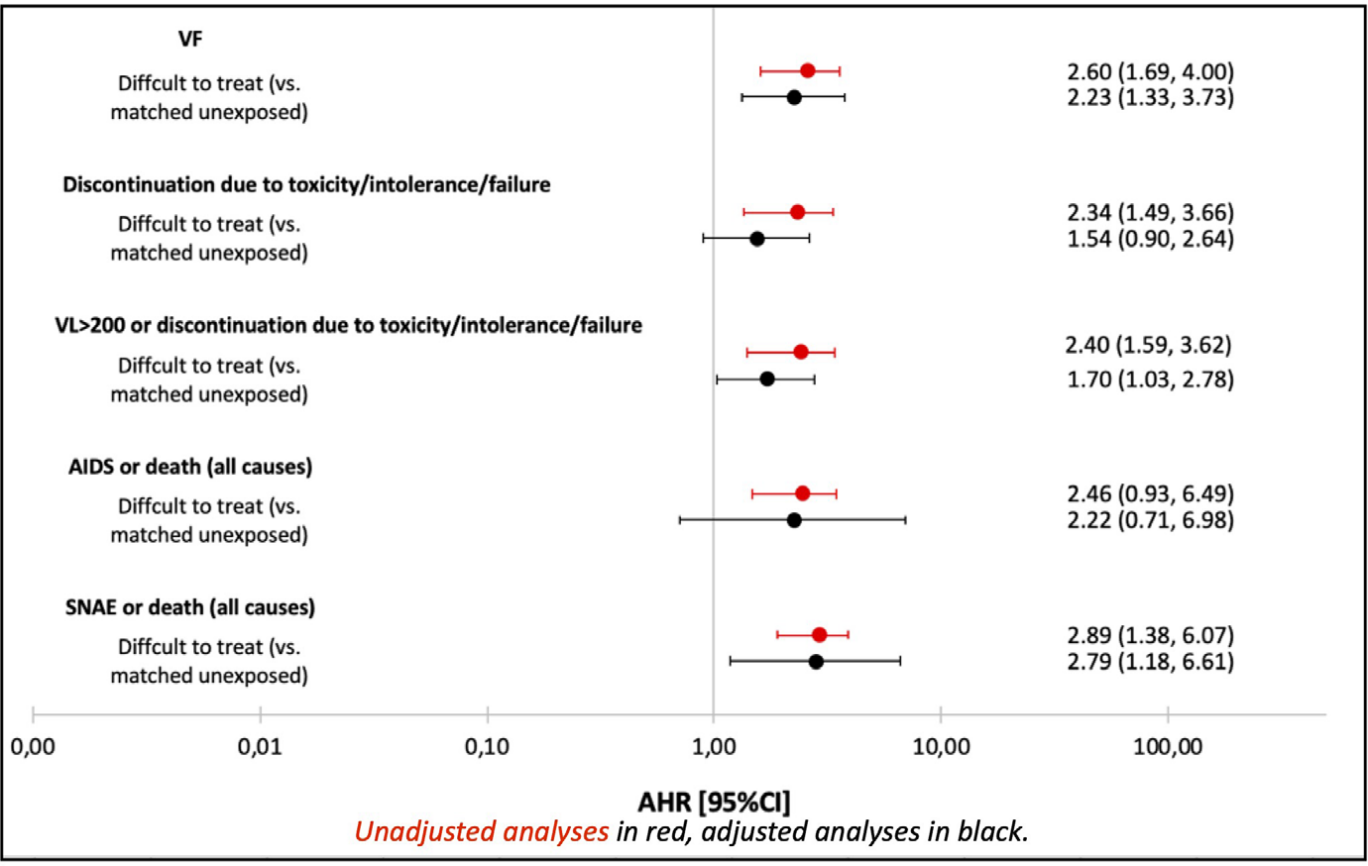
**Abstract P065 – Figure 1**. Cox regression model for VF, discontinuation, treatment failure or clinical failure. Unadjusted analyses in red, adjusted analyses in black. The model was controlled for VL at ART, year of index date, nadir and current CD4 count fitted as time fixed covariate at index date. AHR, adjusted hazard rate; SNAE, serious non‐AIDS event.

**Table:** 

**Abstract P065 – Table 1**. Main characteristics at enrolment by difficult‐to‐treat group.				
Characteristics	Difficult to treat (n = 320)	Not difficult to treat (N = 7741)	p‐value	Total (n = 8061)
Females, n (%)	61 (19.1%)	1412 (18.2%)	0.709	1473 (18.3%)
Mode of HIV transmission, n (%)				
IDU	22 (6.9%)	464 (6.1%)	0.075	486 (6.1%)
Homosexual contacts	137 (43.2%)	3774 (49.4%)		3911 (49.2%)
Heterosexual contacts	141 (44.1%)	2895 (37.4%)		3036 (37.7%)
Other/unknown	17 (5.4%)	503 (6.6%)		520 (6.5%)
Not Italian nationality, n (%)	97 (30.3%)	4171 (53.9%)	<0.001	4268 (52.9%)
HBsAg, n (%)			0.824	
Negative	254 (79.4%)	6177 (79.8%)		6431 (79.8%)
Positive	1 (0.3%)	13 (0.2%)		14 (0.2%)
Not tested	65 (20.3%)	1551 (20.0%)		1616 (20.0%)
HCVAb, n (%)			0.002	
Negative	230 (71.9%)	5774 (74.6%)		6004 (74.5%)
Positive	33 (10.3%)	438 (5.7%)		471 (5.8%)
Not tested	57 (17.8%)	1529 (19.8%)		1586 (19.7%)
Calendar year of baseline, median (IQR)	2014 (2013 to 2016)	2016 (2015 to 2018)	<0.001	2016 (2015 to 2018)
2008 to 2012	76 (23.8%)	542 (7.0%)		618 (7.7%)
2012 to 2016	193 (60.3%)	3344 (43.2%)		3537 (43.9%)
2017+	51 (15.9%)	3855 (49.8%)		3906 (48.5%)
Age, years, median (IQR)	43 (36 to 50)	39 (31 to 49)	<0.001	40 (31 to 49)
CD4 count, cells/mmc, median (IQR)	305 (105 to 473)	355 (167 to 534)		353 (163 to 532)
CD4 count nadir, cells/mmc, median (IQR)	111 (38.4%)	2057 (28.7%)	<0.001	2168 (29.1%)
AIDS diagnosis, n (%)	55 (17.2%)	763 (9.9%)	<0.001	818 (10.1%)
Viral load, log10 copies/mL, median (IQR)	4.93 (4.34 to 5.42)	4.72 (4.11 to 5.30)	0.001	4.73 (4.12 to 5.31)
Time from HIV diagnosis to date of starting ART, months, median (IQR)	1 (1 to 12)	1 (1 to 6)	0.186	1 (1 to 6)
Anchor drug started, n (%)			<0.001	
NNRTI (DOR, RPV)	37 (11.6%)	1469 (19.0%)		1506 (18.7%)
PI (DRV/r)	149 (46.6%)	1568 (20.3%)		1717 (21.3%)
INSTI (RAL, EVG, DTG, BIC)	134 (41.9%)	4704 (60.8%)		4838 (60.0%)

#### P066 Metabolic and hepatic safety in ART‐experienced PLWHIV switching to a DOR‐based regimen versus rilpivirine: data from real life

P Maggi^1^, E Ricci^2^, C Martinelli^3^, N Squillace ^4^, G De Socio^5^, G Orofino^6^, C Molteni^7^, A Masiello^1^, B Menzaghi^8^, F Vichi^9^, E Pistarà^10^, L Valsecchi^11^, A Bitti^12^, L Calza^13^, G Pellicanò^14^, A Cascio^15^, L Taramasso^16^, E Sarchi^17^, K Falasca^18^, O Bargiacchi^19^, G Cenderello^20^, A Di Biagio^21^, P Bonfanti^4^

^1^Infectious Diseases Unit, AORN Sant'Anna e San Sebastiano, Caserta, Italy; ^2^Fondazione ASIA Onlus, Fondazione ASIA Onlus, Buccinasco, Milan, Italy; ^3^AOU Infectious and Tropical Diseases, Careggi Hospital, Florence, Italy; ^4^Infectious Diseases Unit, ASST‐MONZA, San Gerardo Hospital‐University of Milano‐Bicocca, Monza, Italy; ^5^Unit of Infectious Diseases, Santa Maria Hospital, Perugia, Italy; ^6^Division I of Infectious and Tropical Diseases, ASL Città di Torino, Torino, Italy; ^7^Unit of Infectious Diseases, A. Manzoni Hospital, Lecco, Italy; ^8^Unit of Infectious Diseases, ASST della Valle Olona, Busto Arsizio, Italy; ^9^Unit of Infectious Diseases, SOC 1 USLCENTRO FIRENZE, Santa Maria Annunziata Hospital, Florence, Italy; ^10^Unit of Infectious Diseases, Garibaldi Hospital, Catania, Italy; ^11^1st Department of Infectious Diseases, ASST Fatebenefratelli Sacco, Milan, Italy; ^12^Unit of Infectious Diseases, Department of Medicine, Surgery and Pharmacy, University of Sassari, Sassari, Italy; ^13^Department of Medical and Surgical Sciences, Clinics of Infectious Diseases, S. Orsola‐Malpighi Hospital, University of Bologna, Bologna, Italy; ^14^Infectious Diseases, G. Martino Hospital‐University of Messina, Messina, Italy; ^15^Unit of Infectious Diseases, Department of Health Promotion, Mother and Child Care, Internal Medicine and Medical Specialties, University of Palermo, Palermo, Italy; ^16^Infectious Diseases, San Martino Hospital Genoa, University of Genoa, Genoa, Italy; ^17^Infectious Diseases Unit, S.Antonio e Biagio e Cesare Arrigo Hospital, Alessandria, Italy; ^18^Clinic of Infectious Diseases, Department of Medicine and Science of Aging, G. d'Annunzio University, Chieti, Italy; ^19^Unit of Infectious Diseases, Ospedale Maggiore della Carità, Novara, Italy; ^20^Department of Infectious Diseases, Sanremo Hospital, Sanremo, Italy; ^21^Infectious Diseases, San Martino Hospital Genoa, University of Genoa, Genova, Italy

**Background**: Our aim was to investigate the role of switching to a doravirine (DOR)‐based regimen on metabolic and hepatic safety in a real‐life setting. We compared DOR patients with a cohort of subjects switching to rilpivirine (RPV)‐based regimens.

**Patients and methods**: Four hundred and fifty‐nine consecutive experienced PLWHIV, enrolled in the SCOLTA project, starting DOR‐based or RPV‐based regimens, with at least one visit after enrolment were evaluated. T0 and T1 were defined as the baseline and 6‐month follow‐up respectively. Comparisons were performed using chi‐square, paired t‐test, or analysis of variance as appropriate.

**Results**: One hundred and twenty‐eight and 331 subjects were enrolled, respectively, in the DOR and RPV cohorts (Table 1). Fifty‐five (43.0%) patients on DOR were on 3TC/TDF/DOR regimen (DOR1), and the remaining were in other DOR‐based regimens (DOR2). The mean age of patients in DOR1 was lower (46.7 vs 59.5), and they were less frequently switching from integrase inhibitors (36% vs 57%).  At baseline, DOR patients were older, with higher BMI, and more frequently treated with lipid‐lowering drugs and switching from PI and INI. In the first year of observation, 12 (9.3%) patients on DOR interrupted the treatment versus 39 (11.7%) on RPV. Changes from baseline to T1 showed significantly decreasing total cholesterol (TC), LDL‐c, TC/HDL‐c ratio, and triglycerides, both in all patients and in those not on lipid‐lowering treatment. A more marked improvement in lipid profile in the DOR group is suggested by the deeper TC/HDL‐c ratio decrease seen in this group. Patients in DOR1 showed a significant reduction in TC and LDL‐c. Weight did not change. Both DOR and RPV subjects showed a slight increase in ALT, when baseline levels were <40 UI/dL. On the contrary, when ALT was>40 UI/L, the DOR group significantly reduced ALT levels.

**Table:** 

**Abstract P066 – Table 1**. Baseline characteristics of experienced patients in study.			
Variables at enrolment	Doravirine n = 128 mean SD or n (%) or median (IQR)	Rilpivirine n = 331 mean SD or n (%) or median (IQR)	p
Age, years	53.6±12.4	42.3±9.3	<0.0001
Sex, M	91 (71.1%)	237 (71.6%)	0.91
BMI, kg/m^2^	26.2±5.5	24.4±3.8	0.0002
Weight, kg	77.3±17.6	81.7±14.3	0.0007
Caucasian	117 (91.4%)	304 (91.8%)	0.88
Risk factor for HIV acquisition: sexual	89 (69.5%)	244 (73.7%)	0.16
Risk factor for HIV acquisition: IDU	24 (18.8%)	66 (19.9%)	0.16
Risk factor for HIV acquisition: other/ND	15 (11.7%)	21 (6.3%)	0.16
HBV coinfection (n = 102/321)	8 (7.8%)	20 (6.2%)	0.57
HCV coinfection (n = 107/329)	26 (24.3%)	76 (23.1%)	0.80
Detectable HIV‐RNA	16 (12.5%)	50 (15.1%)	0.48
Previous ART: PI	29 (22.7%)	152 (45.9%)	<0.0001
Previous ART: INSTI	62 (48.4%)	17 (5.1%)	<0.0001
Previous ART: NNRTI	47 (36.7%)	151 (45.6%)	0.08
CD4, cells/mm^3^	685 (493 to 955)	624 (443 to 820)	0.02
Total cholesterol, mg/dL	198±45	189±42	0.06
HDL‐cholesterol, mg/dL	51±15	48±16	0.08
LDL‐cholesterol, mg/dL	118±38	112±38	0.12
Triglycerides, mg/dL	114 (85 to 153)	120 (85 to 172)	0.56
On lipid‐lowering drugs	25 (19.5%)	29 (8.8%)	0.001
AST, UI/dL	21 (18 to 29)	24 (19 to 31)	0.008
ALT, UI/dL	24 (17 to 32)	28 (21 to 42)	0.0007

**Conclusions**: In both groups (DOR and RPV), patients showed a significant reduction in TC, LDL‐c, TC/HDL‐c ratio, and triglycerides. A deeper TC/HDL‐c ratio decrease was observed in the DOR group. Patients in DOR1 showed a significant reduction in TC and LDL‐c with respect to DOR2. Moreover, the DOR group showed a significant reduction in ALT levels when ALT were >40 UI/L at baseline.

#### P067 Bictegravir/emtricitabine/tenofovir alafenamide (B/F/TAF) for the treatment of people living with HIV: 24‐month (24M) analyses by age, race, sex, adherence and late presentation in a multi‐country cohort study

B Trottier ^1^, A Antinori^2^, J De Wet^3^, C Duvivier^4^, H Elinav^5^, S Esser^6^, J Ghosn^7^, J den Hollander^8^, J Lambert^9^, A Milinkovic^10^, C Elliott^11^, T Saifi^12^, R Haubrich^13^, D Thorpe^14^, A Marongiu^15^, C Miralles^16^, S Schellberg^17^, B van Welzen^18^

^1^Recherche, Clinique de Médecine Urbaine du Quartier Latin, Montreal, Canada; ^2^HIV/AIDS Department, National Institute of Infectious Diseases, L. Spallanzani, IRCCS, Rome, Italy; ^3^Family Medicine, Spectrum Health, Vancouver, Canada; ^4^Infectious Diseases Department, AP‐HP ‐ Necker‐Enfants Malades Hospital, Necker‐Pasteur Infectiology Center, Paris, France; ^5^Clinical Microbiology and Infectious Diseases, Hadassah Hebrew University Medical Center, Jerusalem, Israel; ^6^Department of Venerology, Clinic of Dermatology, University Hospital Essen, Essen, Germany; ^7^Department of Infectious Diseases, Bichat University Hospital, Paris, France; ^8^Internal Medicine and Infectious Diseases, Maasstad Hospital, Rotterdam, Netherlands; ^9^Infectious Diseases, Mater Misericordiae University Hospital, Dublin, Ireland; ^10^HIV Clinical Research Group, Chelsea and Westminster Hospital, London, UK; ^11^Medical Affairs, Gilead Sciences Ltd, London, UK; ^12^Medical Affairs, Gilead Sciences Inc., Mississauga, Canada; ^13^Medical Affairs, Gilead Sciences Inc., Foster City, CA, USA; ^14^Medical Affairs, Gilead Sciences Europe Ltd, Stockley Park, UK; ^15^Real World Evidence, Gilead Sciences Europe Ltd, Stockley Park, UK; ^16^Department of Internal Medicine, Complexo Hospitalario Universitario de Vigo, Vigo, Spain; ^17^Novopraxis Berlin GbR, Berlin, Germany; ^18^Internal Medicine and Infectious Diseases, University Medical Centre Utrecht, Utrecht, Netherlands

**Background**: BICSTaR (GS‐EU‐380‐4472/GS‐CA‐380‐4574/GS‐IL‐380‐5335) is an ongoing, observational cohort study evaluating the effectiveness and safety of B/F/TAF in clinical practice in antiretroviral therapy (ART)‐naïve (TN) and ART‐experienced (TE) people living with HIV.

**Table:** 

**Abstract P067 – Table 1**. Viral load at 24M by group (missing=excluded), and change in weight from baseline.		
	HIV‐1 RNA <50 copies/mL at 24M, % (95% CI) [n/N]	p‐value for between‐group change using ^a^Fisher exact test or ^b^chi‐squared test
TN participants:		
Late presenters (CD4 <200 cells/μL and/or ≥1 AIDS‐defining event at baseline) vs non‐late presenters	97 (82 to 100) [28/29] vs 97 (90 to 100) [70/72]	p = 1.00^a^
TE participants:		
Female vs male	97 (89 to 100) [62/64] vs 95 (93 to 97) [435/457]	p = 0.76^a^
Age ≥50 years vs <50 years	96 (93 to 98) [251/262] vs 95 (92 to 97) [246/259)	p = 0.66^b^
Age ≥65 years vs <65 years	100 (92 to 100) [43/43] vs 95 (93 to 97) [454/478]	p = 0.25^a^
Black race vs other races	94 (84 to 99) [48/51] vs 96 (93 to 97) [449/470]	p = 0.72^a^
Adherence <80% vs ≥80%	100 (59 to 100) [7/7] vs 95 (92 to 97) [295/310]	p = 1.00^a^
Adherence <95% vs ≥95%	93 (81 to 99) [40/43] vs 96 (93 to 98) [262/274]	p = 0.65^a^
	**Median (Q1, Q3) absolute weight change from baseline to 24M in participants, kg**	**p‐value for change from baseline using sign test**
TE participants:		
Female	+0.5 (−1.6, +3.0) [n = 49]	p = 0.55
Male	+1.3 (−1.0, +4.8) [n = 327]	p < 0.001
Age <50 years	+1.5 (−0.9, +4.7) [n = 185]	p < 0.001
Age ≥50 years	+1.0 (−1.0, +4.3) [n = 191]	p < 0.001
Black race	+0.9 (−1.0, +4.3) [n = 36]	p = 0.30
Other races	+1.2 (−1.0, +4.4) [n = 346]	p < 0.001
No prior TDF	+1.0 (−1.0, +4.5) [n = 241]	p < 0.001
Received prior TDF	+2.0 (0.0, +4.4) [n = 139]	p < 0.001

**Materials and methods**: This 24M analysis included people living with HIV receiving B/F/TAF in clinical practice (enrolled from June 2018; Canada/Germany/Spain/France/UK/Ireland/Israel/Netherlands/Italy) who were followed up during the COVID‐19 pandemic (cut‐off: 4 August 2021). Individual groups were analysed for effectiveness, safety and weight change.

**Results**: This analysis included 838 participants: TN (n = 135) and TE (n = 703). Of TN participants, 29% (37/129) were late presenters (CD4 <200 cells/μL and/or ≥1 AIDS‐defining event). For TN and TE, 87% and 86% were male; median age was 39 and 49 years; 26% and 48% were aged ≥50 years; 4% and 7% were aged ≥65 years; 77%/11%/4%, and 82%/10%/3% were White/Black/Asian, respectively. Prevalence of comorbid conditions was high, 53% and 73% of TN and TE participants, respectively. Most common overall comorbidities were neuropsychiatric (28%), hyperlipidaemia (18%) and hypertension (17%), with 59% receiving ≥1 concomitant medication(s). At 24M, 97% (104/107) and 95% (497/521) of TN and TE participants, respectively, had HIV‐1 RNA <50 copies/mL (missing=excluded analysis). High effectiveness was observed across all individual groups (Table 1). Persistence was high with 86% on B/F/TAF at 24M. No emergence of resistance to the components of B/F/TAF was reported. Drug‐related adverse events (DRAEs) and serious DRAEs occurred in 15% and 0.2% of participants, respectively. Withdrawal of B/F/TAF due to DRAEs/serious DRAEs occurred in 7%/0.1%. TE: DRAE rates were comparable by age (17% <50 years, 13% ≥50 years; p = 0.17) and sex (16% women, 15% men; p = 0.81); discontinuations/serious AEs were low in all groups. Overall, there were two deaths in the TN group, and eight deaths in the TE group (all unrelated to B/F/TAF). Median (Q1, Q3) weight change from baseline to 24M: TN [n = 75] +4.3 kg (0.0, +9.0); TE [n = 376] +1.2 kg (−1.0, +4.5).

**Conclusions**: In clinical practice, B/F/TAF showed high effectiveness and was well tolerated through 24M in a broad population of people living with HIV with a high burden of comorbidities.

#### P068 Real‐world study with dolutegravir (DTG) plus rilpivirine (RPV) as STR (JULUCA^©^) in treatment‐experienced HIV patients

C Hidalgo‐Tenorio, C Garcia Vallecillos, R Javier, M Lopez‐Ruz, S Sequera, J Pasquau

Infectious Diseases Unit, Hospital Universitario Virgen De Las Nieves, Granada, Spain

**Background**: Two‐drug regimen (2DR) in naive (DTG + lamivudine (3TC)) and treated‐experienced HIV patients (DTG+3TC, DTG+RPV, CAB (cabotegravir)+RPV) have been positioned as another strategy in clinical practice.

**Objectives**: To analyse in real life the efficacy of dual therapy (2DR) with JULUCA^©^, and its effect on virological and immunological condition, lipid profile and inflammatory markers.

**Methods**: Two hundred and twenty HIV pre‐treated patients were included between 29/01/2019 and 2/02/2022; the subjects had changed their antiretroviral treatment (ART) to DTG 50 mg plus RPV 25 mg in STR after verbal informed consent of the patient. The study was approved by the ethics committee of Granada.

**Results**: 70.5% were male, mean age of 53 years, 45.3% had AIDS, and CD4 nadir 284.5±202.4 cells/uL. Median HIV infection diagnosis was 19.1 years (IQR 11.6 to 27). Previously to 2DR, subjects had received a median of 5 lines of ART (IQR 4 to 8), during 16.5 years (IQR 12 to 24), 27.7% three‐drug regimens (3DRs), 28.2% monotherapy and 44.1% other 2DRs; 14.4% had previous virological failure (VF). Twelve patients had primary and secondary NNRTI resistance mutations on file that conferred either resistance or decreased susceptibility to rilpivirine. The reasons for 2DR were simplification 61.4%, and to avoid potential long‐term toxicities 34.3%, virological failure 2.9%; median time of 2DR (JULUCA^©^) was 23 months (P25 to P75: 14 to 28.5); 2.9% of patients had blips, 1.4% VF due to poor adherence (had no previous resistance mutations) and 0.5% changed 2DR to avoid drug interactions. During follow‐up CD4/CD8 ratio increased (0.98±0.59 vs 1.01±0.54; p = 0.002). 99.1% had viral load (VL) undetectable and 6.8% blips PLHIV prior to 2DR versus 98.1% VL <50 copies/mL and 2.9% blips during treatment with 2DR, p = 0.317 and p = 0.012, respectively; reduction of TC/HDLc ratio (4.04±1.06 vs 3.62±0.885; p = 0.048), LDLc (123.5±32.4 mg/dL vs 107.3±26.8 mg/dL, p = 0.0001) and TG levels (131.4±75.9 mg/dL vs 116.6±71.4 mg/dL, p = 0.0001); finally, we found no statistically significant differences in IL‐6, CRP, fibrinogen, and D‐dimer levels.

**Conclusions**: Switching to DTG plus RPV in STR in treatment‐experienced HIV patients is an effectiveness strategy, with a favourable lipid profile, which does not influence the inflammatory markers.

### Treatment Strategies: Adherence

#### P069 Implementation of cabotegravir and rilpivirine long‐acting (CAB+RPV LA): primary results from the CAB+RPV Implementation Study in European Locations (CARISEL)

B Van Welzen ^1^, L Vandekerckhove^2^, C Jonsson‐Oldenbuettel^3^, L Hocqueloux^4^, M Ait‐Khaled^5^, G Bontempo^6^, R DeMoor^7^, J Scherzer^8^, R D'Amico^9^, N Barnes^10^, M Hadi^10^, E Low^10^, S Anand^10^, M Czarnogorski^11^, C Gutner^12^

^1^Internal Medicine & Infectious Diseases, The University Medical Centre Utrecht, Utrecht, Netherlands; ^2^Department of Internal Medicine and Pediatrics & HIV Cure Research Centre, Ghent University, Ghent, Belgium; ^3^HIV Research and Clinical Care Center, Medizinisches Versorgungszentrum München am Goetheplatz / Munich Research GmbH, Munich, Germany; ^4^Infectious and Tropical Diseases, Le Centre Hospitalier Régional d'Orléans, Hôpital de la Source, Orléans, France; ^5^Clinical Sciences, R&D, ViiV Healthcare Ltd, Brentford, UK; ^6^Research & Development, ViiV Healthcare Ltd, Branford, CT, USA; ^7^R&D Biostatistics, GlaxoSmithKline, Collegeville, PA, USA; ^8^Global Health Outcomes, ViiV Healthcare GmbH, Munich, Germany; ^9^Research & Development, ViiV Healthcare Ltd, Durham, NC, USA; ^10^Patient‐Centered Research, Evidera, London, UK; ^11^Global Medical Sciences, ViiV Healthcare, Durham, NC, USA; ^12^Global Medical Sciences, Innovation and Implementation Science, ViiV Healthcare Ltd, Durham, NC, USA

**Background**: CARISEL examines the acceptability, appropriateness, and feasibility of cabotegravir (CAB) + rilpivirine (RPV) long‐acting (LA) administered every 2 months for virologically suppressed people living with HIV‐1, and implementation support for staff study participants (SSPs) in HIV centres across Europe.

**Materials and methods**: Eighteen clinics were randomised to implementation support packages: Enhanced Arm (Arm‐E) and Standard Arm (Arm‐S). Arm‐E included skilled wrap‐around team meetings, face‐to‐face injection training, continuous quality improvement, and provider/patient toolkits. Arm‐S included video injection trainings and provider/patient toolkits. SSPs provided quantitative/qualitative feedback, and patient study participant (PSP) data were collected over 12 months. SSPs completed questionnaires on acceptability (AIM), appropriateness (IAM), and feasibility (FIM) of implementation and intervention (Table 1).

**Results**: Thirteen of 18 (72%) clinics had no previous CAB+RPV LA experience and were generally distributed equally across arms. Sixty SSPs responded at month (M) 1 and M12. Mean AIM/IAM/FIM scores were ≥3.8 at M12 for implementation‐ and intervention‐based measures. All implementation measures improved over time. An ANCOVA controlling for provider type showed no significant difference between arms. Interview data showed that: CAB+RPV treatment attributes (e.g. reduced dosing frequency) were the strongest drivers of SSP acceptability, PSPs found CAB+RPV LA appropriate, and feasibility facilitators outnumbered barriers. Fifty‐six percent (n = 35/62) of SSPs reported optimal implementation within 1 to 3 months, with more Arm‐S SSPs (22%, n = 7/32) still working on implementation than Arm‐E (13%, n = 4/30) at M12. Most (71%, n = 24/34) SSPs in France and Spain reached optimal implementation in 1 to 3 months. Time spent in the clinic across study visits averaged 67.2 minutes in Arm‐E and 65.1 minutes in Arm‐S. At M12, 68% (n = 42/62) of SSPs thought the time spent in clinic was 'very' or 'extremely acceptable' across arms. Overall, 93% of injections occurred within ±7 days of the target date. At M12, 87% (n = 373/430) of PSPs maintained HIV‐1 RNA <50 copies/mL, and the proportion with HIV‐1 RNA ≥50 copies/mL was 0.7% (n = 3/430; FDA Snapshot).

**Conclusions**: In CARISEL, most clinics had no prior CAB+RPV LA experience. Despite this, high implementation acceptability, appropriateness, and feasibility levels were seen regardless of implementation arm. Implementation measures improved over time. CAB+RPV LA demonstrated high effectiveness and a low failure rate.

**Table:** 

**Abstract P069 – Table 1**. SSP perception of acceptability, appropriateness, and feasibility^a^.									
		Month 1 mean (SD)			Month 5 mean (SD)			Month 12 mean (SD)	
	Total	Arm‐E	Arm‐S	Total	Arm‐E	Arm‐S	Total	Arm‐E	Arm‐S
Acceptability of Implementation Measure (AIM‐Imp)	3.8 (0.76)	3.8 (0.76)	3.9 (0.75)	4.0 (0.72)	4.0 (0.79)	4.1 (0.65)	4.2 (0.71)	4.0 (0.77)	4.3 (0.63)
Implementation Appropriateness Measure (IAM‐Imp)	3.8 (0.77)	3.8 (0.78)	3.9 (0.78)	4.0 (0.74)	3.9 (0.89)	4.2 (0.53)	4.1 (0.70)	3.9 (0.76)	4.2 (0.63)
Feasibility of Implementation Measure (FIM‐Imp)	4.0 (0.64)	4.0 (0.66)	4.0 (0.64)	4.1 (0.72)	4.1 (0.82)	4.2 (0.61)	4.1 (0.73)	4.0 (0.81)	4.3 (0.61)
Acceptability of Intervention (CAB+RPV LA) Measure (AIM‐Int)	4.6 (0.50)	4.6 (0.43)	4.6 (0.56)	4.5 (0.50)	4.5 (0.50)	4.5 (0.51)	4.5 (0.60)	4.5 (0.66)	4.5 (0.55)
Intervention (CAB+RPV LA) Appropriateness Measure (IAM‐Int)	4.2 (0.54)	4.3 (0.53)	4.2 (0.57)	4.3 (0.57)	4.3 (0.43)	4.3 (0.68)	4.3 (0.54)	4.2 (0.47)	4.3 (0.60)
Feasibility of Intervention (CAB+RPV LA) Measure (FIM‐Int)	4.2 (0.56)	4.2 (0.55)	4.3 (0.58)	4.5 (0.49)	4.6 (0.47)	4.5 (0.51)	4.4 (0.60)	4.4 (0.66)	4.4 (0.54)

Arm‐E, Enhanced Arm; Arm‐S, Standard Arm; CAB, cabotegravir; LA, long‐acting; RPV, rilpivirine; SD, standard deviation; SSP, staff study participant.

^a^Acceptability, appropriateness, and feasibility measures are rated on a 1 to 5 Likert scale: 1 'completely disagree'; 2 'disagree'; 3 'neither agree nor disagree'; 4 'agree'; 5 'completely agree'.

#### P070 Patient‐reported outcomes after 152 weeks of HIV maintenance therapy with long‐acting cabotegravir + rilpivirine in the phase IIIb ATLAS‐2M study

V Chounta^1^, E Overton^2^, S Noe^3^, S Swindells^4^, E Negredo^5^, R D'Amico^6^, C Harrington^7^, S Vanveggel^8^, R Van Solingen‐Ristea^9^, Y Wang^10^, C Acuipil^11^, W Spreen ^6^

^1^Global Health Outcomes, ViiV Healthcare Ltd, Brentford, UK; ^2^Internal Medicine, University of Alabama at Birmingham, Birmingham, AL, USA; ^3^HIV Research and Clinical Care Center, MVZ München Am Goetheplatz, Munich, Germany; ^4^Internal Medicine, University of Nebraska Medical Center, Omaha, NE, USA; ^5^Infectious Diseases Department, Hospital Germans Trias i Pujol, Barcelona, Spain; ^6^Research & Development, ViiV Healthcare Ltd, Durham, NC, USA; ^7^Clinical Development, ViiV Healthcare Ltd, Durham, NC, USA; ^8^Statistics and Decision Sciences, Janssen Research & Development, Beerse, Belgium; ^9^Medical Department, Janssen Research & Development, Beerse, Belgium; ^10^Development Biostatistics, GlaxoSmithKline, Collegeville, PA, USA; ^11^Research & Development, GlaxoSmithKline, San Fernando, Buenos Aires, Argentina

**Background**: The phase IIIb ATLAS‐2M study demonstrated durable noninferior efficacy of cabotegravir + rilpivirine long‐acting (CAB+RPV LA) every 8 weeks (Q8W) versus every 4 weeks (Q4W) dosing over 152 weeks in adults with HIV‐1 infection. Herein we present participant experience outcomes through week (W) 152.

**Table:** 

**Abstract P070 – Table 1**. Treatment satisfaction and acceptance of ISRs through week 152.								
Outcome, ITT‐E	Q8W without prior CAB+RPV exposure (n = 319)	Q4W without prior CAB+RPV exposure (n = 323)	Adjusted difference Q8W to Q4W	p‐value for adjusted difference	Already receiving CAB+RPV Q8W (n = 191)	Already receiving CAB+RPV Q4W (n = 193)^b^	Adjusted difference Q8W to Q4W	p‐value for adjusted difference
Baseline total HIVTSQs score,^a^ mean (SD)	57.7 (9.2)	56.7 (9.3)	N/A	N/A	62.2 (5.4)	62.0 (6.7)	N/A	N/A
Adjusted mean change from baseline in total HIVTSQs score by visit (95% CI adjusted mean): week 24	5.1 (4.4 to 5.8)	4.0 (3.3 to 4.7)	1.1	0.036	0.6 (0.0 to 1.2)	0.5 (‐0.1 to 1.1)	0.1	0.875
Adjusted mean change from baseline in total HIVTSQs score by visit (95% CI adjusted mean): week 48	4.9 (4.0 to 5.7)	3.1 (2.3 to 4.0)	1.7	0.004	0.4 (‐0.3 to 1.2)	0.0 (‐0.7 to 0.7)	0.5	0.351
Adjusted mean change from baseline in total HIVTSQs score by visit (95% CI adjusted mean): week 152	5.0 (4.2 to 5.8)	3.2 (2.4 to 4.1)	1.8	0.004	0.4 (‐0.4 to 1.2)	0.2 (‐0.6 to 0.9)	0.3	0.642
	**Q8W (N = 522)**	**p‐value for week 24/48/152 vs week 8**			**Q4W (N = 523)**	**p‐value for week 24/48/152 vs week 8**		
Acceptance of ISRs (PIN),^c^ mean (SD): week 8	1.9 (0.9)				1.9 (0.9)			
Acceptance of ISRs (PIN),^c^ mean (SD): week 24	1.8 (0.8)	0.004			1.8 (0.8)	0.002		
Acceptance of ISRs (PIN),^c^ mean (SD): week 48	1.7 (0.9)	<0.001			1.7 (0.9)	<0.001		
Acceptance of ISRs (PIN),^c^ mean (SD): week 152	1.7 (0.8)	<0.001			1.7 (0.8)	0.002		

CAB, cabotegravir; HIVTSQs, HIV Treatment Satisfaction Questionnaire status version; ISR, injection site reaction; ITT‐E, intention‐to‐treat exposed; LA, long‐acting; N/A, not applicable; PIN, Perception of Injection; Q4W, every 4 weeks; Q8W, every 8 weeks; RPV, rilpivirine; SD, standard deviation; W, week.

^a^Scores can range from 0 (minimum; very dissatisfied) to 66 (maximum; very satisfied);

^b^W24, n = 193; W48, n = 194; W152, n = 194;

^c^scores can range from 1 (totally acceptable) to 5 (not at all acceptable).

**Materials and methods**: In ATLAS‐2M, virologically suppressed adults with HIV‐1 infection, receiving CAB+RPV LA Q4W (ATLAS [NCT02951052] study rollover) or daily oral therapy, were randomised 1:1 to receive CAB+RPV LA Q8W or Q4W. Treatment satisfaction (HIV Treatment Satisfaction Questionnaire status version [HIVTSQs]) and acceptability of injections (Perception of Injection [PIN] questionnaire) were assessed from baseline (satisfaction while on daily oral therapy) up to W152. Preference at W152 for CAB+RPV LA Q8W or Q4W compared with daily oral therapy was also assessed for participants receiving short‐term oral therapy to cover missed injections.

**Results**: Overall, 1045 participants were randomised (median age, 42 years; 27% female [sex at birth]); baseline characteristics were similar between treatment groups. In participants without prior CAB+RPV exposure, HIVTSQs mean scores improved for both LA treatment groups from baseline (satisfaction while on daily oral therapy) to W152. Participants without prior CAB+RPV LA exposure receiving Q8W dosing reported greater overall satisfaction compared with the Q4W group at W152 (p < 0.004), with main drivers presented below (Figure 1). HIVTSQs mean scores for participants already receiving CAB+RPV LA were high at baseline and remained stable through W152, regardless of dosing arm (Table 1). A statistically significant improvement from W8 (first injection assessment) was observed at each time point through W152 for the acceptance of injection site reactions category of the PIN questionnaire for both the Q4W and Q8W groups (Table 1). Most participants preferred CAB+RPV LA compared with daily oral therapy (Q8W: 97% [n = 29/30] vs daily oral: 0% [n = 0/30] vs no preference: 3% [n = 1/30]; Q4W: 88% [n = 35/40] vs daily oral: 5% [n = 2/40] vs no preference: 8% [n = 3/40]).

**Figure:**
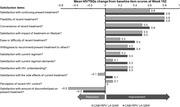
**Abstract P070 – Figure 1**. Mean HIVTSQs change from baseline to week 152 in participants without prior CAB+RPV exposure. CAB, cabotegravir; HIVTSQs, HIV Treatment Satisfaction Questionnaire status version; Q4W, every 4 weeks; Q8W, every 8 weeks; RPV, rilpivirine.

**Conclusions**: These results demonstrate improved treatment satisfaction and preference for CAB+RPV LA compared with daily oral therapy among virologically suppressed adults receiving long‐term maintenance therapy for HIV‐1 infection. Furthermore, these findings contextualise high retention and low discontinuation rates observed for persons receiving CAB+RPV LA.

#### P071 Adherence to HIV treatment in 2019: the COCOVIH study using the French national health insurance claims database

F Prevoteau du Clary ^1^, C Majerholc^2^, D Zucman^2^, J Livrozet^3^, A Vallee^4^, C Laurendeau^5^, S Bouee^5^

^1^Clinical Care, Hôpital La Grave ‐ Cité de la Santé, Toulouse, France; ^2^Clinical Care, Foch Hospital, Suresnes, France; ^3^Clinical Care, Hôpital Edouard Herriot, Lyon, France; ^4^Clinical Research, Foch Hospital, Suresnes, France; ^5^Clinical Research, CEMKA‐EVAL, Bourg La Reine, France

**Background**: COCOVIH is an observational study collecting real‐world data from 2006 to 2019 to evaluate characteristics, comorbidities and ART of people living with HIV (PLWHIV) in France. This evaluation focuses on treatment adherence in 2019 and covariables associated with adherence.

**Methods**: COCOVIH draws upon anonymised records from the national health database SNDS, which includes >90% of the French population registered in CNAM (National Health Insurance Fund). PLWHIV were identified based on ICD‐10 HIV diagnoses, HIV‐specific laboratory tests, and/or prescription of antiretrovirals. Adherence was quantified by the medication possession ratio (MPR) (issued tablets divided by the calculated number of tablets recommended during the last available treatment sequence). Variables of interest included gender, age, type of ART, relevant comorbidities/long‐term conditions and being CMUc recipient (under specific coverage for economically challenged citizens).

**Figure:**
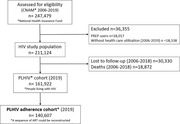
**Abstract P071 – Figure 1**. Flow diagram: selection of study population/adherence cohort.

**Table:** 

**Abstract P071 – Table 1**. MPR (medication possession ratio) >90%: multivariate logistic regression analysis.			
		OR	95% CI
Age (years) (Reference <15)	15 to 29	1.042	[0.866 to 1.254]
	30 to 39	1.307^a^	[1.090 to 1.567]
	40 to 49	1.513^a^	[1.263 to 1.812]
	50 to 64	1.805^a^	[1.506 to 2.163]
	65 to 74	2.110^a^	[1.755 to 2.536]
	>75	1.987^a^	[1.639 to 2.409]
Sex (Ref. female)	Male	1.220^a^	[1.191 to 1.248]
CMUc recipient^b^ (Ref. no)	Yes	0.830^a^	[0.806 to 0.855]
Index year^c^ (Ref. ≤2006 to 2009)	2010 to 2012	1.298^a^	[1.251 to 1.347]
	2013 to 2015	1.546^a^	[1.488 to 1.607]
	2015 to 2017	1.886^a^	[1.801 to 1.975]
	2018 to 2019	2.211^a^	[2.106 to 2.321]
Prevalent comorbidities (Ref. no)	Yes	1.012	[0.986 to 1.038]
Type of ART (Ref. monotherapy)	Dual therapy	1.932^a^	[1.747 to 2.137]
	Triple therapy	2.310^a^	[2.096 to 2.545]
	≥Quadruple therapy	1.707^a^	[1.515 to 1.922]

^a^p < 0.05;

^b^CMUc = Supplementary Universal Health Coverage;

^c^year of HIV diagnosis.

**Results**: A total of 161 922 PLWHIV were identified in 2019 (median age 50 years (IQR 41 to 58), 65.0% male, 36.8% from the capital region Ile‐de‐France, 20.1% CMUc/AME recipients). N = 140 607 participants with at least one ART sequence in 2019 formed the adherence subcohort (Figure 1) (84.4% triple therapy, mean number of annual drug issuances 10.2 (3.1)). Mean (SD) MPR was 82.5% (22.7); 12.7% with MPR <50%, 57% with MPR >90%. For 66.9% of PLWHIV identified in 2018 and for 54.1% of PLWHIV diagnosed in/before 2006, MPR was >90%. The proportion with MPR >90% was lowest in the region Ile‐de‐France (53.9%). Sixty‐nine percent had relevant comorbidities, 6% no comorbidities, with no difference in MPR (mean (SD) MPR: 82.5% (22.4) with comorbidities, 82.3% (23.5) without). Proportion of MPR >90% was however lower in PLWHIV with terminal renal insufficiency (46.6%), chronic renal disease (50.2%), tuberculosis (50.1%) and among users of psychoactive substances (52.3%). Of PLWHIV on monotherapy, 36.0% had an MPR >90%, versus 58% on triple therapy. Factors associated with an MPR >90% are shown in Table 1.

**Conclusion**: This comprehensive real‐world sample of French PLWHIV in 2019 showed that adherence gradually increased with more recent HIV diagnosis possibly reflecting advances in ART tolerability or single tablet regimens. Male sex, advanced age, triple therapy and not being CMUc recipient were identified as factors independently associated with higher MPR.

#### P072 Abstract withdrawn

#### P073 Abstract withdrawn

### Treatment Strategies: Rapid ART

#### P074 Rapid initiation of ART in Ukraine

S Vynohradova, D Shevchenko, A Trotsenko, L Hetman, L Legkostup

Management and Counteraction to HIV Infection, Public Health Center of the Ministry of Health of Ukraine, Kyiv, Ukraine

**Background**: At the end of 2021, Ukraine's progress in achieving the UNAIDS 95‐95‐95 [1] goals was 71%‐81%‐96%. According to the recommendations of the WHO in 2017, Ukraine implements the rapid appointment of ART, within the first 7 days, to accelerate the overcoming of HIV.

**Materials and methods**: We evaluated the data of all regions of Ukraine during January to December 2016 and 2021, according to the legally approved reporting forms and the electronic medical system 'MSSD'.

**Results**: In Ukraine during 2021, 1 923 468 HIV tests were conducted. Seventeen thousand, two hundred and eighty‐nine newly diagnosed PLWHIV were involved in medical supervision, 25% of them had HIV clinical stage IV, which indicates the untimely detection of HIV‐infected patients and as a result is late involvement in ART, treatment and prevention of opportunistic infections. During 2021, 16 660 PLWHIV started ART at the first time. Seventy percent of them have received ART within the first 7 days from the moment of the screening, 8 to 14 days ‐ 11%, 15 to 30 days ‐ 10%, more than 30 days ‐ 9%, which is a significant progress compared to 2016, in which it was about 31% of PLWHIV received ART within first 7 days. This progress may be related to the increasing in the specific weight of the use of rapid test to establish a diagnosis of HIV at the same day. During the first quarter of 2022, the number of HIV tests decreased by 36% compared to the first quarter of 2021, and the absolute number of positive results decreased accordingly, which can be associated with the start of full‐scale war in Ukraine. Despite of it, rapid initiation of ART within the first 7 days among newly diagnosed PLWHIV in 2022 is 83%.

**Conclusions**: Despite the challenges facing Ukraine healthcare facilities continue to perform work efficiently (initiation of ART within the first 7 days among newly diagnosed 83% in I quarter 2022). Due to the high speed of initiation of ART, the significant number of PLWHIV are able to maintain their quality of life and will not transmit HIV to their index partners, which makes possible to stop the spread of HIV in Ukraine.

**Reference**

1. UNAIDS. Understanding Fast‐Track: Accelerating Action to End the AIDS Epidemic by 2030 [Internet]. 2015 [cited 2022 Aug 23]. Available from: https://www.unaids.org/sites/default/files/media_asset/201506_JC2743_Understanding_FastTrack_en.pdf.

#### P075 Viral suppression at 1 year and rapid antiretroviral initiation in Guatemala

H Marroquín, M Robles, D Ortíz, J Samayoa

Unidad de Atención Integral del VIH e Infecciones Crónicas, Hospital Roosevelt, Ciudad de Guatemala, Guatemala

**Background**: Antiretroviral therapy (ART) initiated early after confirming HIV diagnosis has been linked to improved clinical outcomes. As this strategy becomes the standard of care results from in resource‐limited settings are needed. As part of a project evaluating the outcomes of early ART initiation we assessed the prevalence of viral suppression at 1 year in the HIV and Chronic Infectious Diseases Comprehensive Care Unit at Hospital Roosevelt in Guatemala City.

**Materials and methods**: This analysis was performed with data collected from people with HIV (PWHIV) diagnosed and initiated on ART between February 2018 and December 2019. We assessed the prevalence of viral suppression (VS) and its associated factors through univariate analysis. Potential predictors (p ≤ 0.05) were included in a multivariate binary logistic regression model. VS at 1 year was defined as a viral load measurement below 200 copies/mL, early initiation as ART initiation within 7 days of HIV diagnosis confirmation.

**Results**: Five hundred and thirty‐six PWHIV where included. Four hundred and thirty‐nine (81.9%) were <40 years old, 83 (15.5%) female, 419 (78.2%) lived in Guatemala City, 23 (4.3%) Mayan, 113 (21.1%) had primary education or less. At baseline 196 (36.6%) had less <200 cells/mL, 270 (50.4%) viral load above 105 copies/mL and 74 (13.8%) were diagnosed with an opportunistic infection. Four hundred and eighteen (78%) had early initiation of ART, 124 (23.1%) initiated an integrase strand transfer inhibitor (INSTI)‐based regimen. Four hundred and eighty‐two (89.9%) participants had VS at 1 year after ART initiation. Having primary education or less was more common in those with viral non‐suppression at 1 year, meanwhile rapid initiation of ART was more common in those suppressed at the same time point (Table 1). On multivariate analysis, early initiation of ART was associated with VS at 1 year (OR 4.6; CI 2.5 to 8.5; p < 0.01).

**Table:** 

**Abstract P075 – Table 1**. Between group differences at 1 year of initiating ART (viral suppression vs viral non‐suppression).			
Characteristic	Viral suppression	Viral non‐suppression	p‐value
Rapid ART initiation	393 (81.5%)	25 (46.3%)	<0.01
INSTI‐based regimen	106 (22.0%)	18 (33.3%)	0.06
Female	71 (14.7%)	12 (22.2%)	0.15
Age <40 years	400 (83%)	39 (72.2%)	0.05
Resident of Guatemala City	379 (78.6%)	40 (74.1%)	0.44
Mayan ethnicity	18 (3.7%)	5 (9.3%)	0.07
Primary education or less	96 (19.9%)	17 (31.5%)	<0.05
CD4 <200 at baseline	168 (34.9%)	28 (51.9%)	0.14
Viral load 100k at baseline	236 (49%)	34 (63%)	0.05
Opportunistic infection at diagnosis	62 (12.9%)	12 (22.2%)	0.06

**Conclusions**: Viral suppression after 12 months of ART initiation was significantly higher in the early ART initiation group.

#### P076 Rapid initiation of antiretroviral therapy (ART) with bictegravir/emtricitabine/tenofovir alafenamide (BIC/FTC/TAF) in a tertiary hospital in Barcelona, Spain: a prospective clinical trial

A Ugarte, L De La Mora, I Chivite, E de Lazzari, E Fernández, E Solbes, A Inciarte, J Ambrosioni, M Laguno, M Martínez‐Rebollar, A González‐Cordón, J Blanco, E Martínez, J Mallolas, B Torres

HIV Unit, Hospital Clinic, Barcelona, Spain

**Background**: Rapid initiation of ART after HIV diagnosis confers individual and public health benefits [1,2], but some regimens chosen might have limitations. BIC/FTC/TAF is a recommended regimen for rapid initiation due to its efficacy, high‐genetic barrier, safety, simplicity, and lack of major interactions and restrictions.

**Patients and methods**: Prospective, 48‐week, single‐centre, single‐arm, proof‐of‐concept trial enrolling HIV‐positive, ART‐naïve adults referred to or diagnosed at our hospital. BIC/FTC/TAF was started within the first week prior to laboratory test results. Clinical assessment and blood tests were obtained at baseline and at 4, 12, 24 and 48 weeks. Dual‐X‐absorptiometry scans were performed at baseline and 48 weeks. The primary endpoint was the proportion of participants with characteristics potentially limiting ART choice for rapid initiation: positive HLA B*5701 and/or HBsAg, genotypic resistance mutations, CD4 <200 cells/mm^3^, VL >10^5^ copies/mL, low bone mineral density (BMD), Framingham risk score (FRS) >10%, concomitant medications at risk for drug‐drug interactions and eGFR ≤50mL/min. Secondary endpoints were time from HIV first visit at our centre to start of ART, immune and virological status, inflammatory and immunosenescence markers, adverse events, treatment discontinuation, adherence, and patient satisfaction at week 48.

**Results**: We report preliminary results at week 4. Among 100 included participants, 5% were women. Median age was 32 (IQR 27 to 38) years and 64% were from Latin America. At baseline, HLA B*5701 was positive or could not be assessed in 4%; one participant was HBsAg positive; seven participants had K103, with no mutations to BIC/FTC/TAF components; 21% had CD4 counts <200 cells/mm^3^ and 32% VL >10^5^ copies/mL; 46% had low BMD and 10% had FRS >10%. Twelve percent of patients took >1 medication with potential DDI. No patient had eGFR ≤50mL/min. BIC/FTC/TAF was started in 78% of participants within 24 hours of first visit. At week 4, all participants attended, there were no treatment discontinuations, and 54% had already achieved <50 copies/mL.

**Conclusions**: Preliminary results show that rapid BIC/FTC/TAF initiation is feasible and can be safely used in a rapid treatment initiation strategy. A substantial proportion of participants were found to have baseline characteristics that would have precluded the use of some other regimens.

**References**

1. Ford N, Migone C, Calmy A, Kerschberger B, Kanters S, Nsanzimana S, et al. Benefits and risks of rapid initiation of antiretroviral therapy. AIDS. 2018;32:17‐23.

2. Pilcher CD, Ospina‐Norvell C, Dasgupta A, Jones D, Hartogensis W, Torres S, et al. The effect of same‐day observed initiation of antiretroviral therapy on HIV viral load and treatment outcomes in a US public health setting. J Acquir Immune Defic Syndr. 2017;74:44‐51.

#### P077 Patient‐reported symptom outcomes following DTG/3TC use in a test‐and‐treat setting: results from the STAT Study

A Oglesby^1^, J Matthews^2^, K Angelis^3^, P Leone^2^, M Cupo^4^, B Wynne^2^, D Merrill ^5^, C Nguyen^5^, J Van Wyk^6^, A Zolopa^5^

^1^Global Health Outcomes, ViiV Healthcare, Durham, NC, USA; ^2^Clinical Development, ViiV Healthcare, Durham, NC, USA; ^3^Biostatistics, GlaxoSmithKline, Brentford, UK; ^4^Clinical Development, GlaxoSmithKline, Philadelphia, PA, USA; ^5^Medical Affairs, ViiV Healthcare, Durham, NC, USA; ^6^Global Medical Sciences, ViiV Healthcare, Brentford, UK

**Background**: The STAT study previously demonstrated the feasibility, efficacy, and safety of using DTG/3TC in newly diagnosed people living with HIV (PLWHIV) in a test‐and‐treat setting through 48 weeks [1,2]. Here we present data on patient reported symptom burden through week 48.

**Materials and methods**: STAT is a single‐arm study of treatment‐naive adult PLWHIV who initiated DTG/3TC ≤14 days after HIV‐1 diagnosis prior to knowledge of screening/baseline laboratory results. Adjustment to DTG/3TC therapy was permitted if baseline testing indicated resistance to DTG or 3TC, HBV co‐infection, or creatinine clearance <30 mL/min/1.73 m^2^. Symptom burden was assessed with the HIV Symptom Distress Module (SDM) questionnaire which measures the presence and symptom bother level of 20 specific symptoms associated with HIV or its treatment. The SDM was assessed at baseline and through week 48. Individual symptom scores range from 1 to 4 with 4 indicating the highest level of symptom bother. Overall Symptom Bother Score (SBS) ranges from 0 (no symptoms present) to 80 (all symptoms present at highest bothering level). Change from BL in SBS was calculated at weeks (WK) 4, 8, 12, 24, 36 and 48. The proportion of participants with bothering level ≥3 for each symptom (bothersome symptoms) was also assessed.

**Results**: Mean SBS at BL was 13.8. SBS improved by WK 4 and was sustained at each assessed time point under treatment with DTG/3TC (Table 1). Of the 20 symptoms assessed, 19 were reported as bothersome at BL by ≥5% of subjects. All improved by WK 4 except for ‘Problems having sex’ which was improved by WK 8 (BL: 10%, week 4: 11%, week 8: 6%) and sustained thereafter. The most frequently reported (≥15% of subjects) bothersome symptoms at BL were: difficulty sleeping (BL: 27%, WK 4: 16%, WK 48: 10%); fatigue (21%, 10%, 7%); feeling sad or depressed (19%, 15%, 11%), anxiousness (17%, 16%, 10%), or fever, chills or sweats (15%, 5%, 3%).

**Table:** 

**Abstract P077 – Table 1**. Overall Symptom Bother Score change from baseline by visit for DTG/3TC ‐ last observation carried forward (LOCF).			
Visit	n	Mean (SD)	95% CI
Baseline	130	13.8 (14.7)	
Week 4	121	‐4.0 (10.0)	‐5.8 to ‐2.2
Week 8	123	‐5.7 (11.6)	‐7.8 to ‐3.7
Week 12	123	‐6.0 (11.4)	‐8.0 to ‐3.9
Week 24	123	‐5.3 (13.5)	‐7.7 to ‐2.9
Week 36	123	‐7.1 (13.6)	‐9.5 to ‐4.7
Week 48	123	‐6.2 (13.9)	‐8.7 to ‐3.8

**Conclusions**: In this test‐and‐treat setting, initiation with DTG/3TC resulted in rapid improvement of symptoms commonly associated with HIV or its treatment in the enrolled participants. This improvement was sustained throughout the study period.

**References**

1. Rolle C‐PM, Berhe M, Singh T, Ortiz R, Wurapa AK, Ramgopal M, et al. High rates of virologic suppression with DTG/3TC in newly diagnosed adults with HIV‐1 infection and baseline viral load >500,000 c/mL: 48‐week subgroup analysis of the STAT Study. Open Forum Inf Dis. 2021;8(suppl 1):S49.

2. Rolle C‐PM, Berhe M, Singh T, Ortiz R, Wurapa A, Ramgopal M, et al. Dolutegravir/lamivudine as a first‐line regimen in a test‐and‐treat setting for newly diagnosed people living with HIV. AIDS. 2021;35:1957‐65.

#### P078 Impact of COVID‐19 on the outcomes of rapid ART initiation among people living with HIV (PLWHIV): a multicentre study

Y Huang ^1^, S Cheng^2^, C Yang^3^, H Sun^4^, P Lu^5^, C Liu^6^, Y Lee^7^, C Tsai^8^, N Lee^8^, B Liou^9^, H Tang^10^, C Lee^11^, C Lin^12^, C Hung^4^

^1^Internal Medicine, National Taiwan University Hospital Hsin‐Chu Branch, Hsin‐Chu Country, Taiwan; ^2^Internal Medicine, Taoyuan General Hospital, Taoyuan, Taiwan; ^3^Internal Medicine, Far Eastern Memorial Hospital, New Taipei, Taiwan; ^4^Internal Medicine, National Taiwan University Hospital, Taipei, Taiwan; ^5^Internal Medicine, Kaohsiung Medical University Hospital, Kaohsiung, Taiwan; ^6^Internal Medicine, Changhua Christian Hospital, Changhua, Taiwan; ^7^Internal Medicine, Chung Shan Medical University Hospital, Taichung, Taiwan; ^8^Internal Medicine, National Cheng Kung University Hospital, Tainan, Taiwan; ^9^Internal Medicine, Hsinchu Mackay Memorial Hospital, Hsin‐Chu, Taiwan; ^10^Internal Medicine, Chi Mei Medical Center, Tainan, Taiwan; ^11^Internal Medicine, Kaohsiung Chang Gung Memorial Hospital, Kaohsiung, Taiwan; ^12^Internal Medicine, National Taiwan University Hospital Yun‐Lin Branch, Yun‐Lin, Taiwan

**Background**: Taiwan has implemented rapid ART initiation (within 7 days of confirmed HIV diagnosis) programme to improve HIV care continuum in 2018. However, the control measures during COVID‐19 pandemic may have disrupted health‐care delivery. We investigated the impact of COVID‐19 on the short‐term outcomes of PLWHIV who were on rapid ART initiation.

**Materials and methods**: Between 2018 and 2021, medical records of newly diagnosed PLWHIV who were on rapid ART initiation were reviewed to collect the information on the dates of HIV diagnosis, ART initiation, loss to follow‐up (LTFU), occurrence of sexually transmitted infections (STIs), plasma HIV RNA load (PVL), and CD4 count. All PLWHIV were followed for at least 24 weeks after ART initiation. The primary outcome was the proportion of PLWHIV engaged in care at week 48. The secondary outcomes were the proportion of PLWHIV engaged in care at week 24 and those with PVL <50 copies/mL at weeks 24 and 48.

**Results**: During the 4‐year period, 579 of 764 (75.8%) newly diagnosed PLWHIV initiated ART within 7 days of confirmed HIV diagnosis (Table 1); 38.8% had CD4 counts <200 cells/mm^3^ and 48.6% plasma HIV RNA >5 log10 copies/mL. At week 24, the proportions of retention in care among PLWHIV on rapid ART initiation in the pre‐COVID‐19 and the COVID‐19 era were 96.9% and 97.9%, respectively (p = 0.749); at week 48, they were 94.8% and 94.7% (p > 0.999). However, more scheduled blood tests were missed in the COVID‐19 era at both week 24 (6.5% vs 23.3%, p < 0.001) and week 48 (4.7% vs 34.8%, p < 0.001). Using the last‐observation‐carried‐forward (LOCF) approach, the proportions of PVL <50 copies/mL at week 24 and week 48 were 94.4% and 97.6% in the pre‐COVID‐19 era (p = 0.082) and 90.3% and 93.8% in the COVID‐19 era (p = 0.126), respectively. Factors associated with PVL <50 copies/mL with LOCF analysis at week 48 were occurrences of STIs [aOR 0.18, 95% CI 0.06 to 0.58] and baseline PVL (aOR 0.05, 95% CI 0.01 to 0.43).

**Conclusions**: While the rates of retention in care of newly diagnosed PLWHIV on rapid ART initiation remained high during the COVID‐19 era, the numbers of scheduled blood testing performed were significantly reduced.

**Table:** 

**Abstract P078 – Table 1**. Comparison of the outcomes of rapid ART initiation among PLWHIV in the pre‐COVID era (2018 to 2019) and the COVID era (2020 to 2021).			
	Pre‐COVID‐19 era (2018 to 2019) (n = 287)	COVID‐19 era (2020 to 2021) (n = 292)	p‐value
Characteristics			
Age, mean (SD)	32.4 (8.8)	31.8 (9.2)	0.451
MSM, n (%)	263 (91.6)	265 (90.8)	0.671
CD4 count cells/mm^3^, median (IQR)	270 (122 to 422)	250 (91 to 426)	0.357
CD4 counts <200 cells/mm^3^, n (%)	101 (35.7)	121 (42.0)	0.121
PVL ≥5 log10 copies/mL, n (%)	122 (43.0)	158 (54.1)	0.007
Same‐day ART initiation, n (%)	126 (43.9)	188 (64.4)	<0.001
Week 24 outcomes, n (%)			
Death	3 (1.1)	3 (1.0)	>0.999
Loss to follow‐up	6 (2.1)	3 (1.0)	0.336
Virologic failure/rebound	0	0	−
Retention in care (including transferral of care)	277 (96.5)	285 (97.6)	0.749
Retention in care but missing week 24 blood testing	17/263 (6.5)	63/271 (23.3)	<0.001
PVL <200 copies/mL^a^	287/287 (100)	282/289 (97.6)	0.015
PVL <50 copies/mL^a^	269/286 (94.4)	254/281 (90.3)	0.082
Week 48 outcomes, n (%)			
Death	5 (1.7)	5 (1.9)	>0.999
Loss to follow‐up	10 (3.5)	7 (2.7)	0.631
Virologic failure/rebound	0	2 (0.8)	−
Retention in care (including transferral of care)	272 (94.8)	246 (94.7)	>0.999
Retention in care but missing week 48 blood testing	12/253 (4.7)	60/230 (34.8)	<0.001
PVL <200 copies/mL^a^	287/287 (100)	290/291 (99.7)	>0.999
PVL <50 copies/mL^a^	280/287 (97.6)	273/291 (93.8)	0.126

^a^Last‐observation‐carried‐forward analysis.

#### P079 Rapid antiretroviral therapy initiation and acute HIV infection: current situation in a Latin American reference centre

M Santos^1^, F Grimaldi^1^, L Vignolo^1^, F Pinilla Huayta^1^, J Barletta^1^, M Cabrini^1^, O Cando^2^, M Rolón ^1^

^1^Infectious Disease, Hospital General de Agudos Dr. Juan A. Fernandez, Buenos Aires, Argentina; ^2^Virology, Hospital General de Agudos Dr. Juan A. Fernandez, Buenos Aires, Argentina

**Background**: Only 4% of notified new HIV diagnoses in Argentina are made in the context of an acute retroviral syndrome (ARS). Rapid antiretroviral therapy (ART) initiation (i.e. within 7 days of diagnosis) is recommended by the World Health Organization (WHO) and could be particularly relevant at both individual and population levels during acute HIV infection (AHI). The aim of this study is to describe clinical characteristics and time to ART initiation in a cohort of AHI in a reference centre in Buenos Aires, Argentina.

**Materials and methods**: This is an observational retrospective cohort study. Subjects with AHI (defined as positive p24 antigen, ARS with viral load >log7 or documented HIV seroconversion, i.e. negative HIV test within 6 months before inclusion) diagnosed between January 2016 and December 2021 were included.

**Results**: A total of 68 subjects met inclusion criteria (9.6% of the total HIV diagnosis in the study period); 12 were excluded due to missing clinical data. The median age was 28 years (Q1 to Q3 24 to 36); 75% of patients were cis‐men, 16% cis‐women and 9% trans‐women; 46 (82%) were symptomatic or had a laboratory abnormality: fever (78%), thrombocytopenia (50%), transaminitis (48%), adenopathy (39%), pharyngitis (35%), rash (30%) and/or leukopenia (30%); four patients required hospital admission. The prevalence of active syphilis among the tested population (n = 52) was 32%. At the time of diagnosis, median plasma HIV viral load and CD4 were 5.59 log (Q1 to Q3 5.07 to 6.43; n = 43) and 438 cell/mm^3^ (Q1 to Q3 308 to 532; n = 41) respectively. Of the 56 patients included, seven were transferred and 10 were lost to follow‐up. The remaining 39 were included in the follow‐up analysis and started ART within 6 months of diagnosis; 17.9% (n = 7) of patients met the WHO rapid ART initiation criteria; the time to ART commencement throughout the study period is shown in Table 1.

**Conclusions**: The clinical characteristics of our cohort were similar to those previously described in the literature. Although a trend towards reduction in time until ART initiation is observed throughout the study period, significant efforts are still required towards the WHO rapid ART initiation goal.

**Table:** 

**Abstract P079 – Table 1**. Time to ART initiation.		
	n (%)	Median in days (Q1 to Q3)
2016	3 (8)	81 (1 to 178)
2017	6 (15.5)	32 (22 to 69)
2018	4 (10)	19.5 (15 to 27.5)
2019	18 (46)	17.5 (8 to 36)
2020	2 (5)	16 (11 to 21)
2021	6 (15.5)	10.5 (3 to 30)

### Treatment Strategies: Simplification

#### P080 Dolutegravir/lamivudine is clinically non‐inferior to dolutegravir‐based triple drug antiretroviral therapy: 1‐year results of the DUALING real‐world nationwide prospective matched cohort study

M Vasylyev ^1^, F Wit^2^, C Jordans^1^, R Soetekouw^3^, S van Lelyveld^3^, G Kootstra^4^, C Delsing^4^, H Ammerlaan^5^, M van Kasteren^6^, E Brouwer^6^, E Leyten^7^, M Claassen^8^, R Hassing^8^, J den Hollander^9^, M van den Berge^10^, A Roukens^11^, M van Vonderen^12^, W Bierman^13^, P Groeneveld^14^, S Lowe^15^, B van Welzen^16^, O Richel^17^, J Nellen^18^, G van den Berk^19^, M van der Valk^2^, B Rijnders^1^, C Rokx^1^

^1^Internal Medicine, Erasmus MC Universitair Medisch Centrum, Rotterdam, Netherlands; ^2^HIV Monitoring Foundation, Stichting HIV Monitoring, Amsterdam, Netherlands; ^3^Internal Medicine, Spaarne Gasthuis, Haarlem, Netherlands; ^4^Internal Medicine, Medisch Spectrum Twente, Enschede, Netherlands; ^5^Internal Medicine, Catharina Ziekenhuis Endhoven, Eindhoven, Netherlands; ^6^Internal Medicine, Elisabeth Tweesteden Ziekenhuis, Tilburg, Netherlands; ^7^Internal Medicine, Haaglanden Medisch Centrum, The Hague, Netherlands; ^8^Internal Medicine, Rijnstate Ziekenhuis, Arnhem, Netherlands; ^9^Internal Medicine, Maasstadziekenhuis, Rotterdam, Netherlands; ^10^Internal Medicine, Admiraal de Ruyter Ziekenhuis, Vlissingen, Netherlands; ^11^Internal Medicine, Leiden Universitair Medisch Centrum, Leiden, Netherlands; ^12^Internal Medicine, Medisch Centrum Leeuwarden, Leeuwarden, Netherlands; ^13^Internal Medicine, Universitair Medisch Centrum Groningen, Groningen, Netherlands; ^14^Internal Medicine, Isala Ziekenhuis, Zwolle, Netherlands; ^15^Internal Medicine, Maastricht Universitair Medisch Centrum, Maastricht, Netherlands; ^16^Internal Medicine, Universitair Medisch Centrum Utrecht, Utrecht, Netherlands; ^17^Internal Medicine, Radboud Universitair Medisch Centrum, Nijmegen, Netherlands; ^18^Internal Medicine, Amsterdam Universitair Medisch Centrum, Amsterdam, Netherlands; ^19^Internal Medicine, Onze Lieve Vrouwe Gasthuis, Amsterdam, Netherlands

**Background**: Dolutegravir/lamivudine (DTG/3TC) is recommended for treatment‐naïve and ‐experienced people with HIV (PWHIV). Real‐world clinical efficacy data of switching to this dual regimen compared to well‐matched controls continuing triple drug DTG‐based regimens are unavailable.

**Materials and methods**: DUALING is a nationwide prospective cohort study embedded within the Dutch ATHENA cohort to compare treatment outcomes of switching from DTG‐based triple antiretroviral therapy (ART) to DTG/3TC in well‐suppressed PWHIV without prior virological failure, hepatitis B or mutations associated with DTG/3TC resistance (cases) with matched controls continuing DTG‐based triple ART. We matched the first 390 consecutive cases 1:2 to 780 controls by age, sex, HIV acquisition category, absent prior virological failure, pre‐ART CD4+T‐cells and viral load. Follow‐up for controls started at visit dates closest to the matched case DTG/3TC start. We report 1‐year outcomes in the 'on treatment' population with treatment failure defined as (1) two consecutive viral loads >50 copies/mL, or (2) one viral load >50 copies/mL directly followed by ART switch, death or lost‐to‐follow‐up. Individuals switching ART or becoming lost‐to‐follow‐up with viral loads <50 copies/mL were censored.

**Results**: The 1170 participants were median 48.1 years (37.9 to 56.8), 88.2% males, 94.1% acquired HIV sexually, median pre‐ART CD4+T‐cell count 315 (190 to 480) and viral load 70 900 copies/mL (23 000 to 210 000). Ten (2.6%) cases and 18 (2.3%) controls became lost‐to‐follow‐up with viral load <50 copies/mL. Eighteen (4.6%) cases and 138 (17.7%) controls switched ART with viral loads <50 copies/mL. Of the remaining 362 cases and 624 controls, five (1.4%) and six (1.0%) experienced treatment failure (p = 0.54). One case and two controls had treatment failure because they switched ART following one viral load between 50 and 200 copies/mL. Four cases and four controls had treatment failure because of two consecutive viral loads >50 copies/mL. Of these eight, two controls had peak viraemia >200 copies/mL (440 and 1120 copies/mL). Two in each group re‐suppressed to <50 copies/mL without ART switch, and two in each group continued having low‐level viraemia without ART switch. The treatment failure risk difference for cases compared to controls was +0.42% (95% CI ‐1.01 to +1.85%).

**Conclusions**: Switching to DTG/3TC in well‐suppressed PWHIV was highly efficacious and non‐inferior after 1 year compared to matched controls continuing DTG‐based triple ART in a real‐world setting.

#### P081 Effectiveness of dolutegravir + lamivudine in real‐world studies in people with HIV‐1 with M184V/I mutations: a systematic review and meta‐analysis

M Kabra^1^, T Barber^2,3^, C Allavena^4^, A Marcelin^5^, S di Giambenedetto^6^, J Pasquau^7^, N Gianotti^8^, M Turner^9^, C Harrison^9^, T Wynne^9^, G Verdier^10^, C Parry^11^, B Jones^12^, C Okoli^12^, J Priest ^13^, E Letang^14^

^1^Global Health Outcomes, ViiV Healthcare, Brentford, UK; ^2^Ian Charleson Day Centre, Royal Free London NHS Foundation Trust, London, UK; ^3^Institute for Global Health, University College London, London, UK; ^4^Infectious Diseases, Centre Hospitalier Universitaire Hôtel‐Dieu, Nantes, France; ^5^Infectious Diseases, Hôpital Pitié‐Salpétrière, Paris, France; ^6^Infectious Diseases, Fondazione Policlinico Universitario Agostino Gemelli Istituto di Ricovero e Cura a Carattere Scientifico, Rome, Italy; ^7^Infectious Diseases, Virgen de las Nieves University Hospital, Granada, Spain; ^8^Infectious Diseases, Istituto di Ricovero e Cura a Carattere Scientifico Ospedale, Milan, Italy; ^9^Health Economics and Outcomes Research, Health Economics and Outcomes Research Ltd, Cardiff, UK; ^10^Medical Affairs, ViiV Healthcare, Montréal, Canada; ^11^Translational Medicine, ViiV Healthcare, Brentford, UK; ^12^Medical Affairs, ViiV Healthcare, Brentford, UK; ^13^Global Health Outcomes, ViiV Healthcare, Durham, NC, USA; ^14^Medical Affairs, ViiV Healthcare, Madrid, Spain

**Background**: Antiretroviral regimens are typically not recommended if resistance to components is known or suspected, but historical resistance results are not always available when considering treatment options. In phase III trials evaluating switch to dolutegravir/lamivudine (DTG/3TC), absence of historical resistance results (n = 294; pooled TANGO/SALSA) or presence of archived M184V/I mutations (TANGO, n = 4; SALSA, n = 5) did not impact virologic efficacy. This meta‐analysis describes virologic failure (VF) at weeks 24, 48, and 96 using real‐world data from people with HIV‐1 (PWHIV) receiving DTG+3TC in a suppressed switch setting, with historical RNA‐ or archived proviral DNA‐detected M184V/I mutation.

**Figure:**
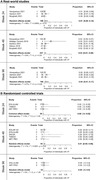
**Abstract P081 – Figure 1**. Meta‐analysis estimates of proportions of VF at weeks 24, 48, and 96 in people with HIV‐1 and reported M184V/I receiving DTG+3TC from (A) systematic literature review–identified real‐world studies and (B) targeted literature review–identified randomized controlled trials, inclusive of all VF definitions. Proportions were log‐transformed, or arcsine‐transformed if any studies reported zero events. DTG+3TC, dolutegravir + lamivudine; VF, virologic failure.

**Materials and methods**: A systematic literature review was performed following Preferred Reporting Items for Systematic Reviews and Meta‐analysis guidelines. Embase^®^, Ovid MEDLINE^®^, MEDLINE^®^ In‐Process, and Cochrane library (January 2013 to March 2022) and relevant conference archives (2016 to 2021) were searched for real‐world studies reporting virologic outcomes for PWHIV receiving DTG+3TC. A targeted literature review was performed to identify randomized controlled trials (RCTs) assessing M184V/I impact on DTG+3TC efficacy. Studies were screened for populations reporting historical M184V/I mutations before DTG+3TC initiation. Fixed‐ and random‐effects model analyses were conducted from real‐world studies (primary objective). Sensitivity analyses were performed using RCT data (secondary objective).

**Table:** 

**Abstract P081 – Table 1**. Summary of real‐world study and randomized controlled trial definitions for PWHIV with M184V/I resistance‐associated mutations receiving DTG+3TC.			
Study (cohort)	Proportion with M184V/I	VF definition	M184V/I identification method
Real‐world studies			
Hocqueloux 2021 (Dat'AIDS)	105/695 (15.11%)	2 consecutive confirmed VL >50 copies/mL or 1 VL >200 copies/mL	RNA and proviral DNA genotypes (pooling both)
Santoro 2021 (LAMRES)	36/533 (6.75%)	2 consecutive confirmed VL >50 copies/mL or 1 VL >200 copies/mL	RNA and proviral DNA genotypes
Borghetti 2021 (ODOACRE)	48/669 (7.17%)	1 VL ≥1000 copies/mL or 2 consecutive VL ≥50 copies/mL	Historical genotypes; does not specify RNA or proviral DNA
Galizzi 2018 (NR)	47/174 (27.01%)	2 consecutive confirmed VL >50 copies/mL or 1 VL >50 copies/mL followed by treatment modification, or 1 VL >1000 copies/mL	Either RNA or proviral DNA genotypes at baseline (before switch)
Hidalgo‐Tenorio 2019 (DOLAMA)	4/178 (2.25%)	2 consecutive VL >50 copies/mL	Baseline RNA genotype
Randomized controlled trials			
ART‐PRO	17/41 (41.46%)	VL ≥50 copies/mL	Proviral DNA genotype
SOLAR 3D	50/100 (50.00%)	VL ≥50 copies/mL	Historical genotypes; does not specify RNA or proviral DNA
TANGO	4/322 (1.24%)	VL ≥50 copies/mL	Proviral DNA genotype
DOLULAM	17/27 (62.96%)	VL ≥200 copies/mL	RNA and proviral DNA genotypes
SALSA	5/192 (2.60%)	VL ≥40 copies/mL	Proviral DNA genotype

DTG+3TC, dolutegravir + lamivudine; NR, not reported; PWHIV, people with HIV‐1; VF, virologic failure; VL, viral load.

**Results**: Of 3492 publications and 198 conference abstracts identified via systematic literature review, five real‐world studies met all search criteria and were analyzed; the targeted literature review also identified five relevant RCTs (Table 1). Proportions of PWHIV with historical M184V/I estimated to have VF at weeks 24, 48, and 96 were low in real‐world and RCT analyses based on VF events (real‐world: 3/186 [1.61%], 7/237 [2.95%], and 7/186 [3.76%], respectively; RCT: 0/38 [0%], 2/93 [2.15%], and 0/34 [0%], respectively; Figure 1), with no reported treatment‐emergent resistance mutations. Including all studies increased sample sizes without significantly impacting estimates.

**Conclusions**: Although overall M184V/I incidence was low, real‐world studies of PWHIV with historical M184V/I receiving DTG+3TC identified low incidence of VF through 96 weeks, as did sensitivity analyses from RCTs. Though not indicated in PWHIV with known resistance mutations, this meta‐analysis provides data on outcomes with DTG+3TC in PWHIV with incomplete history or in cases where archived M184V/I was inadvertently missed.

#### P082 Switching to bictegravir/emtricitabine/tenofovir alafenamide (BIC/FTC/TAF) plus darunavir/cobicistat (DRV/c) in heavily antiretroviral‐experienced, virologically suppressed HIV‐infected adults receiving complex regimens

D Podzamczer ^1^, A Imaz^2^, A López Lirola^3^, H Knobel^4^, M Masiá^5^, J Berenguer^6^, C Hernández^7^, M Lagarde^8^, Á Gutiérrez^9^, A Curran^10^, L Morano^11^, M Montero‐Alonso^12^, J Troya^13^, R Rigo^14^, M Casadellà^15^, A Navarro‐Alcaraz^2^, A Rivero Roman^16^

^1^Fundacio Lluita contra la SIDA Science, Fight Infections Foundation, Badalona, Spain; ^2^Infectious Diseases, Hospital Universitari de Bellvitge, Hospitalet, Spain; ^3^Internal Medicine, Hospital Universitario de Canarias, San Cristobal de la Laguna, Spain; ^4^Infectious Diseases, Hospital del Mar, Barcelona, Spain; ^5^Infectious Diseases, Hospital General Universitario de Elche, Elche, Spain; ^6^Microbiology and Infectious Diseases, Hospital Gregorio Marañon, Madrid, Spain; ^7^Infectious Diseases, Hospital Príncipe de Asturias, Alcalá de Henares, Spain; ^8^Internal Medicine, Hospital Universitario 12 de Octubre, Madrid, Spain; ^9^Internal Medicine, Hospital Universitario de la Princesa, Madrid, Spain; ^10^Infectious Diseases, Hospital Universitario Vall d'Hebron, Barcelona, Spain; ^11^Internal Medicine ‐ Infectious Diseases, Hospital Universitario Álvaro Cunqueiro, Vigo, Spain; ^12^Infectious Diseases, Hospital Universitario y Politécnico La Fe, Valencia, Spain; ^13^Internal Medicine, Hospital Universitario Infanta Leonor, Madrid, Spain; ^14^Biochemistry Laboratory, Hospital Universitari de Bellvitge, Hospitalet, Spain; ^15^Infectious Diseases, IrsiCaixa – Institut de Recerca de la SIDA, Badalona, Spain; ^16^Infectious Diseases, Hospital Universitario Reina Sofía, Córdoba, Spain

**Background**: Most patients receive single‐pill regimens as initial ART. In contrast, those with previous failures/toxicities often take complex multi‐tablet regimens, hampering adherence and long‐term success. We evaluated the efficacy and safety of a simple two‐pill regimen of BIC/FTC/TAF plus DRV/c as a switching/simplification strategy in a group of heavily treatment‐experienced patients taking a high burden of pills.

**Methods**: Multicentre, prospective, single‐arm pilot clinical trial. Virologically suppressed adults receiving a stable antiretroviral regimen of at least three pills from at least three drug families due to previous virological failures and/or toxicities without documented resistance to INSTI nor DRV in genotypic RNA drug‐resistance tests were included. Clinical and laboratory assessments were done at 0, 4, 12, 24, 36, 48 weeks (w). HIV‐1 proviral DNA was amplified and sequenced by Illumina (next‐generation sequencing, Stanford HIVdb criteria) at baseline to detect resistance mutations. Due to a potential interaction between bictegravir (BIC) and DRV/c, plasma BIC concentrations (UHPLC‐MS/MS) were determined in 22 patients at week 4. The main study endpoint was viral load (VL) <50 copies/mL at week 48 (ITT). We present protocol predefined results at 24w.

**Results**: Sixty‐three participants were enrolled and 61 (97%) reached 24w of follow‐up. Median age 63 years, 92% men, CD4 515 cells/uL, 24 years (IQR 16 to 28) on ART, most common previous regimen DRVr+ETR+RAL (14%), median number of pills 4 (range 3 to 10). At baseline, proviral DNA could be amplified in 39 participants: 33/39 had resistance mutations (RM): 16 had intermediate (11)/high resistance (five) to tenofovir, 14 to 3TC/FTC (M184V/I), nine RM to tenofovir+3TC/FTC (two of them with intermediate resistance to DRV). No participants had resistance to BIC or DTG. Twenty‐two participants had RM to other NRTI, and eight to EVG/RAL. Two participants were lost to follow‐up, one had VL >50 copies/mL (<200 copies/mL). No participant discontinued therapy due to adverse effects. By ITT, 95% (58/61) had VL <50 copies/mL and 98% by PP analysis at week 24. Median BIC plasma concentrations were 2943 ng/mL pre‐dose and 7594 ng/mL 2 hours post‐dose.

**Conclusions**: Preliminary 24w results suggest that BIC/FTC/TAF + DRV/c is an effective, well‐tolerated regimen which may improve convenience and potentially long‐term success in stable heavily pre‐treated HIV patients.

#### P083 HIV‐1 RNA blips, low‐level viral replication and mean CD4+/CD8+ ratio during phase III/IIIb cabotegravir + rilpivirine long‐acting studies up to 152 weeks of therapy

C Latham ^1^, L Garside^2^, S Byrapuneni^3^, C Smith^4^, M St. Clair^5^, V Van Eygen^6^, S Griffith^7^, R D'Amico^7^, J van Lunzen^8^, J van Wyk^9^, W Spreen^7^

^1^Translational Medicine Research, ViiV Healthcare, Durham, NC, USA; ^2^Statistical Programming, GSK, Brentford, UK; ^3^Statistical Programming, Parexel International, Research Triangle Park, NC, USA; ^4^Statistical Programming, Parexel International, Sheffield, UK; ^5^Clinical Virology, ViiV Healthcare, Durham, NC, USA; ^6^Clinical Virology, Janssen Research and Development, Beerse, Belgium; ^7^Research and Development, ViiV Healthcare, Durham, NC, USA; ^8^Translational Medicine Research, ViiV Healthcare, Brentford, UK; ^9^Global Medical Affairs, ViiV Healthcare, Brentford, UK

**Background**: Cabotegravir + rilpivirine long‐acting (CAB+RPV LA) is the only long‐acting therapy approved for virologically suppressed people living with HIV (PLWHIV). Non‐inferiority of Q4W CAB+RPV LA to daily oral standard of care (SOC) through week (W)96 was demonstrated in the phase III FLAIR study. Week 48 study data showed that the proportion of participants with HIV‐1 RNA blips (single HIV‐1 RNA between 50 and <200 with adjacent values <50 copies/mL), target virus not detected (TND) and HIV‐1 RNA <2 copies/mL were similar in the CAB+RPV LA (Q4W) and oral three‐drug SOC groups. In addition, HIV‐1 RNA blips were not associated with the development of confirmed virologic failure [(CVF) ‐ two consecutive HIV‐1 RNA ≥200 copies/mL]. Here we report HIV‐1 RNA blip, TND, HIV‐1 RNA <2 copies/mL and CD4+/CD8+ ratio data and the impact of HIV‐1 RNA blips on CVF through W96 in FLAIR.

**Table:** 

**Abstract P083 – Table 1**. Proportions of participants with CVF, blips, or HIV‐1 RNA ≥50 copies/mL through week 96.						
Treatment group	Participants with CVF through W96	Participants with HIV‐1 blip^a^ at any study visit	Participants with HIV‐1 RNA ≥50 copies/mL at W96 (FDA Snapshot)	Participants without HIV‐1 blip^a^ and HIV‐1 RNA ≥50 copies/mL at W96	Participants with HIV‐1 blip^a^ and HIV‐1 RNA ≥50 copies/mL at W96	CVF participants with HIV‐1 blip^a^ through W96
Q4W CAB+RPV LA	4/283 (1%)	45/283 (16%)	9/283 (3%)	7/238 (3%)	2/45 (4%)	0/45
Oral three‐drug SOC	4/283 (1%)	48/283 (17%)	9/283 (3%)	6/235 (3%)	3/48 (6%)	1/48 (2%)

^a^HIV‐1 RNA between 50 and <200 copies/mL with adjacent values <50 copies/mL.

**Materials and methods**: Plasma samples were analyzed for HIV‐1 RNA viral load using the Abbott RealTime HIV‐1 assay and TND outcomes were provided for HIV‐1 RNA <40 copies/mL. The HIV‐1 SuperLow assay (bioMONTR Labs) measured HIV‐1 RNA <2 copies/mL at baseline and W96. Lymphocyte subsets were assessed by flow cytometry (Q2 Solutions).

**Results**: The proportion of participants with HIV‐1 blips was 16% in Q4W and 17% in SOC groups through W96 (Table 1). FDA Snapshot analysis showed that of participants with blips, 4% of Q4W and 6% of SOC participants had HIV‐1 RNA ≥50 copies/mL at W96. No Q4W participant and 1/48 SOC participants with HIV‐1 blips developed CVF through W96. TND outcomes at individual study visits were similar between study arms (76% to 89% ‐ Q4W; 73% to 85% ‐ SOC) and proportion of participants with HIV‐1 RNA <2 copies/mL (60% to 69% ‐ Q4W; 57% to 74% ‐ SOC) was comparable between treatment groups through W96. Mean and median CD4+/CD8+ ratios through W96 were similar at individual study visits in both groups.

**Conclusions**: The proportion of study participants with HIV‐1 RNA blips, TND, HIV‐1 RNA <2 copies/mL and mean CD4+/CD8+ ratio (proxy for immune‐activation) were similar between Q4W CAB+RPV LA and oral three‐drug SOC groups through W96 in FLAIR. Although the number of CVFs in FLAIR is low, the presence of HIV‐1 RNA blips did not appear to be associated with CVF development through W96.

#### P084 Real‐world data from the prospective, multicentre study on the use of dolutegravir‐based regimens (DBRs) in ART‐naive and experienced people living with HIV: 12‐month results from the Russian TESLA study

S Kuznetsov ^1^, S Minaeva^2^, N Sizova^3^, F Nagimova^4^, E Orlova‐Morozova^5^, M Radzikhovskaya^6^, V Shevchenko^7^, A Maldonado^8^, B Jones^9^

^1^Medical Affairs, GSK, Moscow, Russian Federation; ^2^General Medicine, Nizhny Novgorod Regional Centre for AIDS and Infectious Diseases Prophylaxis and Control, Nizhny Novgorod, Russian Federation; ^3^General Medicine, Centre for AIDS and Infectious Diseases Prophylaxis and Control, St. Petersburg, Russian Federation; ^4^General Medicine, Tatarstan Republican Centre for AIDS and Infectious Diseases Prophylaxis and Control, Kazan, Russian Federation; ^5^General Medicine, Moscow Regional Centre for AIDS and Infectious Diseases, Prophylaxis and Control, Moscow, Russian Federation; ^6^General Medicine, Regional Centre for AIDS and Infectious Diseases Prophylaxis and Control, Chelyabinsk, Russian Federation; ^7^General Medicine, Altai Regional Centre of AIDS and Infectious Diseases Prophylaxis and Control, Barnaul, Russian Federation; ^8^Global Medical Affairs, ViiV Healthcare, Wavre, Belgium; ^9^Global Medical Affairs, ViiV Healthcare, Brentford, UK

**Background**: TESLA is the first prospective real‐world data study regarding the effectiveness and long‐term safety of using DBRs in both ART‐naive and experienced people living with HIV (PLWHIV) in Russia. Here we present the study demographics and month 12 outcomes.

**Materials and methods**: TESLA is a prospective, non‐interventional, 3‐year Russian study involving 1000 PLWHIV who started taking DBRs in 14 Russian centres. Study data was collected during visits in accordance with routine clinical practice. Each participant in the study was observed until dolutegravir (DTG) was discontinued, death, loss of contact, or the end of data collection. Reasons for discontinuation were recorded. Viral suppression was defined as viral load (VL) <250 copies/mL after 12 months of follow‐up.

**Results**: Nine hundred and eighty‐two PLWHIV were included in the full analysis set for 12 months (81% ART‐experienced, 27% on two‐drug regimen, 56% male, median age 40 years, median body mass index (BMI) 24 kg/m^2^). Of ART‐experienced participants, regimen prior to switch contained NNRTI, PI and INSTI in 57%, 47% and 2%, respectively. Baseline HIV‐RNA was <250 copies/mL in 83% and CD4 cell count ≥200 cells/mm^3^ in 91% of ART‐experienced PLWHIV; in ART‐naive PLWHIV, 26% had viral load ≥100 000 copies/mL and 77% had CD4 counts ≥200 cells/mm^3^ at baseline. Viral suppression rates are depicted in Figure 1. Notably, in 89% of ART‐experienced PLWHIV, HIV‐RNA levels were <250 copies/mL and 80% of the ART‐naive individuals were virally suppressed at 12 months. Overall median CD4 cell count increased by 80.0 cells/mm^3^. Nine percent of PLWHIV (n = 92/982) discontinued the study; main reasons were loss of contact (6%), discontinuation of DTG (2%), death (0.7%) and physician's decision (0.3%). Until 12 months, 98 adverse drug reactions (ADRs)/serious adverse events (SAEs) were documented in 80 PLWHIV (8%), of which 56 ADRs in 54 PLWHIV (5.5%) were evaluated as DTG‐related. There were small weight changes: median BMI increased by +0.4 kg/m^2^ (0.0 to 1.1).

**Figure:**
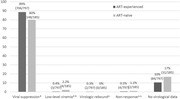
**Abstract P084 – Figure 1**. Virological outcomes at month 12 (N = 982; n = 115/982 with missing data). * VL <250 copies/mL; ** VL >250 and <500 copies/mL; ^ two consecutive measurements of VL ≥500 copies/mL after previously achieved suppression (<250 copies/mL); ^^ two consecutive measurements of VL ≥250 copies/mL after at least 6 months of treatment.

**Conclusions**: The use of DTG‐based regimens in this real‐world Russian TESLA cohort was associated with a high level of effectiveness and safety through 12 months. The rate of ADRs associated with DTG was 5.5%, no new safety signals identified.

#### P085 Safety and efficacy of switching people with HIV to dual treatment with 3TC/DTG and RPV/DTG in real life: results from the SCOLTA cohort

A De Vito ^1^, E Ricci^2^, G Orofino^3^, L Taramasso^4^, E Sarchi^5^, C Costa^6^, G Pellicanò^7^, L Pagnucco^8^, B Menzaghi^9^, G De Socio^10^, B Celesia^11^, S Piconi^12^, P Maggi^13^, P Bonfanti^14^, G Madeddu^1^

^1^Department of Medicine, Surgery and Pharmacy, University of Sassari, Sassari, Italy; ^2^Fondazione Asia, Fondazione ASIA Onlus, Buccinasco, Italy; ^3^Unit of Infectious Diseases, 'Divisione A', Amedeo di Savoia Hospital, Torino, Italy; ^4^Infectious Disease Clinic, IRCCS Policlinico San Martino Hospital, Genova, Italy; ^5^Infectious Diseases Unit, SS. Antonio e Biagio e Cesare Arrigo Hospital, Alessandria, Italy; ^6^Infectious Diseases Department, SOC 1, USLCENTROFIRENZE, Santa Maria Annunziata Hospital, Firenze, Italy; ^7^Unit of Infectious Diseases, Department of Human Pathology of the Adult and the Developmental Age, University of Messina, Messina, Italy; ^8^Infectious Diseases Unit Department of Medical Science and Infectious Diseases, Fondazioner IRCCS Policlinico San Matteo, University of Pavia, Pavia, Italy; ^9^Unit of Infectious Diseases, ASST della Valle Olona, Busto Arsizio Hospital, Busto Arsizio, Italy; ^10^Clinic of Infectious Diseases, Department of Medicine, Azienda Ospedaliera di Perugia, Santa Maria Hospital, Perugia, Italy; ^11^Unit of Infectious Diseases, University of Catania, ARNAS Garibaldi, Catania, Italy; ^12^Infectious Disease Unit, Ospedale A. Manzoni, Lecco, Italy; ^13^Department of Infectious Disease, University of Campania Luigi Vanvitelli, Napoli, Italy; ^14^Infectious Diseases Clinic, San Gerardo Hospital, University of Milano‐Bicocca, Monza, Italy

**Introduction**: After the publication of big randomised clinical trials (SWORD, TANGO, ASPIRE) [1‐3], between 2017 and 2018, guidelines added lamivudine (3TC) + dolutegravir (DTG) and rilpivirine (RPV)+DTG among the recommended regimens for switching strategies in virologically suppressed PLWHIV. However, data on these two strategies from real‐life settings are still scarce. Therefore, we aimed to describe our cohort's characteristics to better understand the profile of people who started these DTG‐based dual regimens in a real‐life setting.

**Table:** 

**Abstract P085 – Table 1**. Characteristics of 359 experienced people living with HIV that started treatment with 3TC/DTG (265) or RPV/DTG (94).				
Variables	3TC/DTG (n = 265)	RPV/DTG (n = 94)	Overall (n = 359)	p‐value
Age, mean (±SD)	50.31±11.76	52.18±11.6	50.80±11.73	0.1835
Female gender, n (%)	53 (20.0)	29 (30.1)	82 (22.8)	0.0313
Caucasian ethnicity, n (%)	245 (92.5)	84 (89.4)	329 (91.6)	0.3521
Other ethnicity, n (%)	20 (7.5)	10 (10.6)	30 (8.4)	0.3521
Risk factor				0.0209
Sexual, n (%)	219 (82.6)	67 (71.3)	286 (79.7)	
IDU, n (%)	25 (9.4)	19 (20.2)	44 (12.3)	
HIV‐RNA detectable at baseline, n (%)	253 (95.5)	89 (94.7)	342 (95.3)	0.7564
HIV‐RNA undetectable at baseline, n (%)	12 (4.5)	5 (5.3)	17 (4.7)	0.7564
Previous regimens, n (%)				
PI	92 (34.7)	32 (34.0)	124 (34.5)	0.906
NNRTI	75 (28.3)	46 (48.9)	121 (33.7)	0.0003
INSTI	80 (30.2)	22 (23.4)	102 (28.4)	0.2102
Anni ART, mean (±SD)	9.9 (5.4 to 14.9)	13.2 (7.1 to 20.6)	10.4 (5.8 to 16.8)	0.0015
CD4 cells/mmc, mean (±SD)	781.4 (359.8)	720.7 (304.2)	765 (346.2)	0.1469
CD4/CD8 ratio, mean (±SD)	1.09 (0.61)	0.97 (0.61)	1.05 (0.61)	0.1319
BMI, mean (±SD)	25.18 (3.69)	24.32 (3.84)	24.92 (3.75)	0.0744

**Figure:**
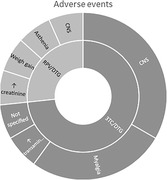
**Abstract P085 – Figure 1**. Adverse effects in the first 2 years of treatment with 3TC/DTG or RPV/DTG.

**Methods**: We analysed data from SCOLTA (Surveillance Cohort Long‐Term Toxicity Antiretrovirals) prospective database, including all experienced people with HIV that started treatment with 3TC/DTG or RPV/DTG. We collected demographical information, risk factors for HIV infection, viro‐immunological data, and cause of treatment interruption.

**Results**: We included 359 people, of which 265 (73.8%) were treated with 3TC/DTG and 94 (36.2%) were treated with RPV/DTG. There was no difference in age and gender in the two groups, while there was a difference in the HIV risk factors, with a higher percentage of people injecting drugs in the RPV/DTG group. Also, patients treated with RPV/DTG have a longer HIV history than 3TC/DTG (9.9 vs 13.2 years, p = 0.0015). Not all patients who started dual regimens had an undetectable HIV‐RNA; indeed, 17 (4.7%) had detectable HIV‐RNA. Most people treated with RPV/DTG were previously treated with an NNRTI regimen (48.9%). General characteristics are summarised in Table 1. During the follow‐up, 203 people up to 208 treated with 3TC/DTG kept an undetectable HIV‐RNA (97.6%), while 78 up to 84 (92.8%) in RPV/DTG group; regarding people who had a detectable HIV‐RNA at baseline, 8/11 (72.7%) treated with 3TC/DTG achieved a stable non‐detectable HIV‐RNA, versus 4/5 (80%) treated with RPV/DTG. Regarding the interruption during the first 2 years, 31 (8.6%) people interrupted the treatment, of which 22 were in 3TC/DTG and nine in RPV/DTG group, without statistical significance. The most common causes of interruption were adverse events (Figure 1) and loss to follow‐up.

**Conclusion**: Both treatments showed a high safety and efficacy in our cohort. RPV/DTG seemed to be preferred for people who came from an NNRTI‐based regimen and people with a longer HIV treatment history.

**References**

1. van Wyk J, Ajana F, Bisshop F, De Wit S, Osiyemi O, Portilla Sogorb J, et al. Efficacy and safety of switching to dolutegravir/lamivudine fixed‐dose 2‐drug regimen vs continuing a tenofovir alafenamide–based 3‐ or 4‐drug regimen for maintenance of virologic suppression in adults living with human immunodeficiency virus type 1: phase 3, randomized, noninferiority TANGO study. Clin Infect Dis. 2020;71:1920‐9.

2. Aboud M, Orkin C, Podzamczer D, Bogner JR, Baker D, Khuong‐Josses M‐A, et al. Efficacy and safety of dolutegravir‐rilpivirine for maintenance of virological suppression in adults with HIV‐1: 100‐week data from the randomised, open‐label, phase 3 SWORD‐1 and SWORD‐2 studies. Lancet HIV. 2019;6:e576‐87.

3. Taiwo BO, Marconi VC, Berzins B, Moser CB, Nyaku AN, Fichtenbaum CJ, et al. Dolutegravir plus lamivudine maintains human immunodeficiency virus‐1 suppression through week 48 in a pilot randomized trial. Clin Infect Dis. 2018;66:1794‐7.

#### P086 Three‐year outcomes of dolutegravir/rilpivirine in virologically suppressed HIV‐infected PLWHIV: real‐world data from the prospective German JUNGLE cohort

F Schabaz ^1^, J Scherzer^2^, S Schneeweiß^3^, D Beer^4^, K Ummard‐Berger^5^, R Pauli^6^, C Wyen^7^, H Hillenbrand^8^, B Westermayer^9^, K Dymek^2^

^1^Private Practice, MVZ Müchen am Goetheplatz, Munich, Germany; ^2^Medical Affairs, ViiV Healthcare, Munich, Germany; ^3^Clinical Care, Praxis Hohenstaufenring, Cologne, Germany; ^4^Clinical Care, PZB Aachen ‐ Praxis Dr. H. Knechten, Aachen, Germany; ^5^Clinical Care, UBN / Praxis, Berlin, Germany; ^6^Clinical Care, Isarpraxis, Munich, Germany; ^7^Clinical Care, Praxis Ebertplatz, Cologne, Germany; ^8^Clinical Care, PraxisCitYOst, Berlin, Germany; ^9^Medical Affairs, GlaxoSmithKline GmbH, Munich, Germany

**Background**: Switching to dolutegravir (DTG)/rilpivirine (RPV) for maintenance of viral suppression is supported by large randomised clinical trials and retrospective cohorts. The German JUNGLE cohort provides prospective real‐world data over 3 years, focusing on virological effectiveness, tolerability and patient‐reported treatment satisfaction.

**Figure:**
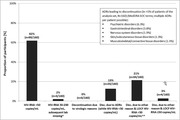
**Abstract P086 – Figure 1**. Virological outcomes at year 3 (effectiveness set: N = 160; n = 16/176 excluded due to missing data). ^ In three PLWHIV with HIV‐RNA 50 to 200 copies/mL have no follow‐up yet (subsequent lab missing); ^^ including one death. LOCF, last observation carried forward.

**Materials and methods**: JUNGLE is a non‐interventional, prospective 3‐year multi‐centre cohort study in PLWHIV on suppressive ART switched to co‐formulated DTG/RPV in accordance with the label. Viral suppression at year 3 was defined as HIV‐RNA <50 copies/mL in data window 33 to 39 months or 50 to 200 copies/mL with subsequent HIV‐RNA <50 copies/mL (discontinuation=failure; excluding missing data/lost to follow‐up). Patient‐reported symptom burden and treatment satisfaction were assessed using the HIV Symptom Distress Module [HIV‐SDM] and the HIV Treatment Satisfaction Questionnaire [status version; HIV‐TSQs].

**Results**: At data‐cut, 176 PLWHIV were eligible for analysis (men: 90%, median age: 48 years, median time on ART: 8 years, median CD4 count: 720/μL). Three‐year retention in the study was 66% (Kaplan‐Meier estimate); 58 PLWHIV (33%) discontinued the study, 11 (6%) were lost to follow‐up. Documented reasons for discontinuation were patient wish/withdrawal (14%), adverse drug reactions (ADRs, 11% [n = 20; year 1: n = 19; year 3: n = 1]), physician's decision (5%), death (1%), and other (2%). Until year 3, there were no confirmed HIV‐RNA >200 copies/mL and no discontinuations for virological reasons; year 3 viral suppression rate was 62% (n = 99/160; effectiveness set, n = 16 excluded due to missing data) which was primarily driven by discontinuations for non‐treatment related reasons (Figure 1). Until data‐cut, 37 ADRs (grades 1 to 2: n = 34; grade 3: n = 3; not applicable: n = 2) were reported in 27 individuals (15%). At years 2 and 3, mean changes in HIV‐SDM were ‐1.6 (p = 0.085) and ‐0.9 (p = 0.163), respectively. Mean changes in HIV‐TSQs were +4.2 (p < 0.001) at year 2 and +4.7 (p < 0.001) at year 3 (Table 1).

**Table:** 

**Abstract P086 – Table 1**. Patient‐reported outcomes: HIV Symptom Distress Module (HIV‐SDM, 20 items, range 0 to 80) and HIV Treatment Satisfaction Status Questionnaire (HIV‐TSQs, range 0 to 60) in PLWHIV completing baseline and year 3.					
	Baseline (BL) mean (SD)	Year 2 mean (SD)	Year 2 change from baseline mean (SD)	Year 3 mean (SD)	Year 3 change from baseline mean (SD)
HIV‐SDM total score^a^	12.6 (13.0); [n = 51]	10.0 (11.7); [n = 44]	−1.6 (7.2); [n = 44]	11.7 (11.9); [n = 51]	−0.9 (7.1); [n = 51]
HIV‐TSQs total score^b^	52.6 (8.3); [n = 50]	56.3 (8.0); [n = 41]	+4.2 (10.4)*; [n = 41]	57.3 (3.6); [n = 50]	+4.7 (8.7); [n = 50]*

SD, standard deviation. *p < 0.05 (BL vs year 2 or 3).

^a^Negative changes indicate improvement;

^b^positive changes indicate improvement.

**Conclusion**: In the prospective real‐world setting of the JUNGLE cohort, DTG/RPV maintained viral suppression over 3 years with no discontinuations for virological reasons. Discontinuation of DTG/RPV was attributed to ADRs in 11% of PLWHIV, with <1% in year 2 and year 3, consistent with a sustained and significant improvement in treatment satisfaction in PLWHIV remaining on DTG/RPV for 3 years.

#### P087 Systematic literature review of real‐world experience with the two‐drug regimen dolutegravir and lamivudine in people with HIV who would not have met inclusion criteria for the phase III clinical program

J Slim ^1^, D Ward^2^, S Schneider^3^, M Kabra^4^, G Verdier^5^, B Jones^4^, E Letang^6^

^1^Infectious Diseases, Saint Michael's Medical Center, Newark, NJ, USA; ^2^Infectious Diseases, Dupont Circle Physicians Group, Washington, DC, USA; ^3^Infectious Diseases, Long Beach Education and Research Consultants, Long Beach, CA, USA; ^4^Medical Affairs, ViiV Healthcare, Brentford, UK; ^5^Medical Affairs, ViiV Healthcare, Montréal, Canada; ^6^Medical Affairs, ViiV Healthcare, Madrid, Spain

**Background**: In phase III randomized controlled trials (RCTs), dolutegravir/lamivudine (DTG/3TC) demonstrated durable efficacy in treatment‐naive (GEMINI‐1/‐2) and virologically suppressed switch (TANGO, SALSA) participants. Eligibility criteria for these RCTs included no history of treatment failure or any major nucleoside reverse transcriptase inhibitor or integrase inhibitor–associated mutations, no hepatitis B virus (HBV) or need for hepatitis C virus (HCV) therapy, and viral load (VL) <500 000 copies/mL at screening (GEMINI) or <50 copies/mL for >6 months (TANGO, SALSA). We analyzed real‐world evidence (RWE) for DTG + 3TC use in people with HIV (PWH) with baseline characteristics not consistent with these inclusion criteria.

**Materials and methods**: We conducted a systematic literature review according to the Preferred Reporting Items for Systematic Reviews and Meta‐analysis statement. RWE studies that reported on DTG + 3TC use in PWH were retrieved from Ovid MEDLINE^®^, Embase^®^, PubMed, Cochrane library, and relevant international conference proceedings from January 2013 to February 2022.

**Results**: This review includes 122 publications from 103 RWE studies of 44 unique cohorts (N = 8034; 42 cohorts outside the United States; Table 1, Figure 1). In the one study that described outcomes in PWH with previous virologic failure (VF; N = 194), probability of VF at 1 year was low (0.4% or 1.2%, depending on VF criteria). In cohorts with >10 PWH with baseline resistance that reported outcomes (mostly M184V/I; four cohorts, N = 211), VF was low (ranging from 0% to 5.4% at ∼1 year), and difference in VF between those with or without M184V/I was not significant in three of four cohorts. A treatment‐emergent resistance mutation (M41L, not selected by DTG or 3TC) was observed in one PWH with evidence of baseline resistance. None of the 35 PWH with HBV experienced VF, and 89% (16/18) of treatment‐naive PWH with baseline VL >500 000 copies/mL achieved virologic suppression at week 24. No studies described effectiveness outcomes in PWH with HCV receiving DTG + 3TC.

**Table:** 

**Abstract P087 – Table 1**. Summary of the number of PWH with reported use of DTG + 3TC by treatment history and baseline characteristic.				
Treatment history			No. of PWH, n (N = 8034)	
Treatment naive			788	
Suppressed switch			6082	
Treatment experienced with detectable viral load at baseline			58	
Treatment experienced with unknown viremia			1106	
	**Studies identifying PWH with characteristic**		**Studies reporting effectiveness outcomes**	
**Baseline characteristic**	**No. of cohorts**	**No. of PWH**	**No. of cohorts**	**No. of PWH**
Previous virologic failure	7	1134	1	194
Evidence of baseline resistance	10	253	4	211
Evidence of HBV	6	166	1	35
Evidence of HCV	13	431	0	0
Treatment‐naive PWH with HIV‐1 RNA >500 000 copies/mL at baseline	1	18	1	18
Treatment‐experienced PWH with HIV‐1 RNA <50 copies/mL for <6 mo before switch	1	13	0	0

3TC, lamivudine; DTG, dolutegravir; HBV, hepatitis B virus; HCV, hepatitis C virus; PWH, people with HIV.

**Figure:**
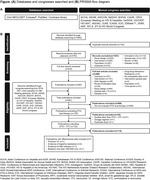
**Abstract P087 – Figure 1**. (A) Databases and congresses searched and (B) PRISMA flow diagram. 3TC, lamivudine; ACHA, Asian Conference on Hepatitis and AIDS; ASHM, Australasian HIV & AIDS Conference; ASICON, National Conference of AIDS Society of India; BASHH, British Association for Sexual Health and HIV; BHIVA, British HIV Association; CAHR, Canadian Conference on HIV/AIDS Research; CROI, Conference on Retroviruses and Opportunistic Infections; DTG, dolutegravir; GeSIDA, Grupo de Estudio del SIDA‐SEIMC; HBV, hepatitis B virus; HIV/HEP, HIV & Hepatitis in the Americas; HIV‐NAT, The HIV Netherlands Australia Thailand Research Collaboration; IAS/IAC, International AIDS Society/International AIDS Conference; ICAR, International Conference on Antiviral Research; ICASA, International Conference on AIDS and STIs in Africa; ICID, International Congress on Infectious Diseases; INSTI, integrase strand transfer inhibitor; JSAR, Japanese Society for AIDS Research; KAP, Kenya Association of Physicians; NRTI, nucleoside reverse transcriptase inhibitor; SFLS, Société Française De Lutte Contre Le Sida; SGA, small for gestational age; STI, sexually transmitted infection; VF, virologic failure; XTC, emtricitabine or lamivudine.

**Conclusions**: DTG + 3TC has been used in PWH with various baseline characteristics, including RCT exclusion criteria. Outcomes from published RWE in these subgroups further support clinical data demonstrating the high effectiveness and barrier to resistance of DTG + 3TC.

### Treatment Strategies: Switch Studies

#### P088 Outcomes after switching from 144 weeks of blinded DTG/ABC/3TC or DTG+F/TAF to 96 weeks of open‐label B/F/TAF

C Orkin ^1^, A Antinori^2^, J Rockstroh^3^, S Moreno Guillén^4^, C Martorell^5^, J Molina^6^, A Lazzarin^7^, F Maggiolo^8^, Y Yazdanpanah^9^, K Andreatta^10^, H Huang^11^, J Hindman^12^, H Martin^12^, J Baeten^12^, A Pozniak^13^

^1^HIV Medicine, Queen Mary University of London, London, UK; ^2^Lazzaro Spallanzani, IRCCS, National Institute for Infectious Diseases, Rome, Italy; ^3^Medizinische Klinik und Poliklinik I, University Hospital Bonn, Bonn, Germany; ^4^Department of Infectious Diseases, Hospital Ramón y Cajal, Madrid, Spain; ^5^Infectious Disease, The Research Institute, Springfield, MA, USA; ^6^Infectious Diseases, University of Paris, Paris, France; ^7^Lazzaro Spallanzani, IRCCS, San Raffaele Hospital Milan, Milan, Italy; ^8^Infectious Diseases, Azienda Ospedaliera Papa Giovanni XXIII, Bergamo, Italy; ^9^Infectious Diseases, AP‐HP Hôpital Bichat, Paris, France; ^10^Clinical Virology, Gilead Sciences, Foster City, CA, USA; ^11^Biostatistics Virology, Gilead Sciences, Foster City, CA, USA; ^12^Clinical Development, Gilead Sciences, Foster City, CA, USA; ^13^Clinical Research, Chelsea and Westminster Hospital, London, UK

**Background**: HIV guidelines offer switch strategies for virologically suppressed people with HIV‐1 (PWHIV), but long‐term clinical follow‐up after the regimen switch is often lacking. Here we assess 96‐week (W) outcomes on bictegravir/emtricitabine/tenofovir alafenamide (B/F/TAF) in an open‐label extension (OLE) that followed 144W of blinded DTG‐based treatment in two phase III studies of PWHIV initiating treatment.

**Materials and methods**: Two randomized, double‐blind, phase III studies of B/F/TAF were conducted in PWHIV initiating first‐line therapy – Study 1489: B/F/TAF versus dolutegravir/abacavir/lamivudine (DTG/ABC/3TC) and Study 1490: B/F/TAF versus DTG+F/TAF. We examined cumulative results for participants who were originally randomized/treated with either DTG/ABC/3TC or DTG+F/TAF for 144W and then switched to 96W of B/F/TAF in an OLE (total of 240W of follow‐up). Efficacy was assessed as the proportion with HIV‐1 RNA <50 copies/mL at each visit after starting B/F/TAF (missing=excluded [M=E] analysis); safety assessed by adverse events (AEs) and laboratory results.

**Results**: In Study 1489, 315 participants randomized to DTG/ABC/3TC; 254 (81%) entered the OLE. In Study 1490, 325 randomized to DTG+F/TAF; 265 (82%) entered the OLE. After switch to B/F/TAF, efficacy was >96% at every visit through W240 (M=E). Eleven participants had HIV‐1 RNA ≥ 50 copies/mL at time of switch, two of whom were later found to have M184V while on blinded DTG/ABC/3TC and resuppressed on B/F/TAF. No resistance to any components of B/F/TAF occurred in any group of the final resistance analysis population. Across both studies (1489: n = 254, 1490: n = 265), 2/519 (0.4%) switch participants experienced an AE that led to drug discontinuation during the OLE. There were no discontinuations due to renal AEs. Grade 3 drug‐related AE occurred in one participant, no grade 4 AEs. Median fasting lipid changes from time of switch to B/F/TAF to OLE W96 were small (Table 1). Participants switching from DTG/ABC/3TC had numerically greater weight increases than those switching from DTG+F/TAF.

**Conclusion**: Over 5 years of follow‐up, adults initially taking DTG/ABC/3TC or DTG+F/TAF who then switched to B/F/TAF and were followed for 96W maintained high virologic suppression and few discontinuations. These results provide additional long‐term evidence of the safety and efficacy of B/F/TAF in those who switch from a DTG‐containing regimen.

**Table:** 

**Abstract P088 – Table 1. **Summary of participant characteristics at time of switch, outcomes through week 240.		
	Study 1489	Study 1490
	DTG/ABC/3TC to B/F/TAF (n=254)	DTG+F/TAF to B/F/TAF (n=265)
Participant characteristics at time of switch^a^		
Median age (Q1, Q3)	36 (30, 45)	38 (30, 48)
Female sex at birth	11%	10%
Black or African descent	37%	30%
Latinx/Hispanic	21%	28%
CD4 count, median, cells/mm^3^ (Q1, Q3)	766 (599, 1023)	730 (550, 958)
eGFRCG, median, mL/min (Q1, Q3)	115.6 (98.5, 137.6)	111.0 (95.1, 134.8)
Body weight, median, kg (Q1, Q3)	83.0 (72.6, 94.3)	81.7 (71.0, 96.0)
Efficacy and safety, W240		
Median duration of exposure to B/F/TAF, W (Q1, Q3)	96 (95.7, 96.3)	96 (95.9, 96.4)
HIV‐1 RNA <50 c/mL (n/N), [Missing=Excluded]	99.5% (217/218)	99.1% (232/234)
HIV‐1 RNA < 50 c/mL (n/N), [Missing=Failure]	85.4% (217/254)	87.5% (232/265)
CD4 count, median change^b^, cells/mm^3^ (Q1, Q3)	‐6 (‐116, 82)	3 (‐91, 110)
eGFRCG, median change^b^, mL/min (Q1, Q3)	2.0 (‐7.2, 12.7)	1.3 (‐7.3, 11.1)
Body weight, median change^b^, kg (Q1, Q3)	2.4 (‐0.4, 5.6)	1.3 (‐1.9, 5.0)
Fasting lipids, mmol/L, median (time of B/F/TAF start → OLE wk 96)		
Total cholesterol	4.27 → 4.53	4.42 → 4.50
LDL	2.90 → 2.97	3.08 → 3.03
HDL	1.24 → 1.24	1.22 → 1.22
Triglycerides	1.08 → 1.11	1.12 → 1.20
Total cholesterol: HDL ratio	3.3 → 3.5	3.5 → 3.7

^a^Defined as the last non‐missing value obtained on or prior to the first dose of open‐label B/F/TAF;

^b^from time of B/F/TAF start. Prior to B/F/TAF, all participants completed 144 weeks of blinded DTG/ABC/3TC or DTG+FTC/TAF.

#### P089 Switching to bictegravir/emtricitabine/tenofovir alafenamide single tablet regimen from boosted protease inhibitor‐based ART in virologically suppressed adults with HIV‐1 harbouring drug resistance: a phase IV randomised, open‐label pilot study (PIBIK study)

C Iwuji ^1^, L Waters^2^, A Milinkovic^3^, C Orkin^4^, N Perry^5^, N Dailey^6^, Y To^6^, S Bremner^7^, A Geretti^8^, D Churchill^9^

^1^Global Health and Infection, University of Sussex, Brighton, UK; ^2^Genitourinary Medicine, The Mortimer Market Centre, Central and North West London NHS Foundation Trust, London, UK; ^3^Genitourinary Medicine, Chelsea and Westminster Hospital NHS Foundation Trust, London, UK; ^4^HIV Medicine, Barts Health NHS Trust, London, UK; ^5^Clinical Trials Unit, University Hospitals Sussex NHS Foundation Trust, Brighton, UK; ^6^Clinical Trials Unit, Brighton & Sussex Clinical Trials Unit, University of Sussex, Brighton, UK; ^7^Primary Care and Public Health, Brighton and Sussex Medical School, University of Sussex, Brighton, UK; ^8^Institute of Infection, University of Liverpool, Liverpool, UK; ^9^Genitourinary Medicine, University Hospitals Sussex NHS Foundation Trust, Brighton, UK

**Background**: There are limited data evaluating the genetic barrier of bictegravir/emtricitabine/tenofovir alafenamide (B/F/TAF) when switching away from boosted protease inhibitor‐based (bPI) antiretroviral therapy in people who are virologically suppressed but harbour nucleoside reverse transcriptase inhibitor (NRTI) resistance. We evaluate this approach.

**Methods**: Eligible adults with HIV‐1 RNA <50 copies/mL were randomised to continue their bPI regimen or switch immediately to B/F/TAF. Individuals with ≤2 thymidine analogue mutations (TAMs) with or without other nucleoside analogue mutations were included unless they had the K65R/N/E mutation or any documented INSTI mutations. After 24 weeks, individuals in the bPI arm were offered a switch to B/F/TAF and followed for a further 24 weeks. We present the primary endpoint ‐ the proportion of participants with HIV‐1 RNA <50 copies/mL at week 24 using pure virological response (PVR). Secondary outcomes include efficacy at 48 weeks, safety, and patient‐reported outcome measures.

**Results**: Seventy‐two patients were randomised (N = 39 bPI vs N = 33 B/F/TAF); 66 (92%) have completed 24 weeks (N = 35 bPI vs N = 31 B/F/TAF). Baseline characteristics: median age 54 years, 89% male and 74% White, median CD4 count 632 cells/mm^3^, 58 (81%) on boosted darunavir and 14 (19%) on boosted atazanavir. Thirty‐eight (53%) had ≥2 NRTI resistance mutations, of the 34 (47%) with <2 NRTI mutations, 19 had an isolated M184V/I. At week 24, all 31 participants who switched to B/F/TAF versus 35/36 (97%) of participants who remained on bPI maintained PVR. Adverse events were reported in 74% (23/31) of participants who switched to B/F/TAF versus 60% (21/35) continuing bPI. Ten of 23 of the adverse events on B/F/TAF were considered drug‐related, all were grade 1 and 2. There was no study withdrawal related to adverse events.

**Conclusions**: The presence of limited NRTI resistance did not compromise efficacy after a switch to B/F/TAF in virologically suppressed adults on bPI after 24 weeks of follow‐up. Switching to B/F/TAF was well tolerated.

#### P090 Efficacy and safety of dolutegravir/lamivudine (DTG/3TC) in Black and Asian participants from TANGO and SALSA: pooled 48‐week data analyzed by race

P Kumar ^1^, D Hagins^2^, J Andrade‐Villanueva^3^, P Lu^4^, E Adachi^5^, R Rubio^6^, T Lutz^7^, M Ait‐Khaled^8^, R Grove^9^, B Wynne^10^, B Jones^11^, C Okoli^11^

^1^Infectious Diseases, Georgetown University Medical Center, Washington, DC, USA; ^2^Infectious Diseases, Georgia Department of Public Health, Savannah, GA, USA; ^3^Infectious Diseases, Hospital Civil, Guadalajara, Mexico; ^4^Infectious Diseases, Kaohsiung Medical University, Kaohsiung, Taiwan; ^5^Infectious Diseases, University of Tokyo, Tokyo, Japan; ^6^Infectious Diseases, Hospital Universitario 12 de Octubre, Madrid, Spain; ^7^Infectious Diseases, Infektiologikum, Frankfurt, Germany; ^8^Clinical Development, ViiV Healthcare, Brentford, UK; ^9^Statistics, GSK, Brentford, UK; ^10^Clinical Development, ViiV Healthcare, Durham, NC, USA; ^11^Global Medical Affairs, ViiV Healthcare, Brentford, UK

**Background**: International guidelines recommend the two‐drug regimen DTG/3TC in switch settings. Globally, Black and Asian populations represent a large proportion of people with HIV; however, some racial and ethnic groups are underrepresented in clinical trials. To increase the sample size of these underrepresented groups, efficacy and safety data from the phase III TANGO and SALSA trials were pooled and analyzed by race.

**Materials and methods**: Week (W) 48 (primary endpoint) data from TANGO and SALSA evaluating switch to once‐daily DTG/3TC fixed‐dose combination or continuing various current antiretroviral regimens (CAR) were pooled. Proportions of participants with HIV‐1 RNA ≥50 and <50 copies/mL (Snapshot, ITT‐E) and safety were analyzed by race. Adjusted mean change from baseline in CD4+ cell count and CD4+/CD8+ ratio was analyzed using mixed‐models repeated measures.

**Results**: Of 1234 participants (DTG/3TC, n = 615; CAR, n = 619), 878 (71%) identified as White, 202 (16%) as Black, 96 (8%) as Asian, and 58 (5%) as other races. At W48, proportion of participants with HIV‐1 RNA ≥50 copies/mL was low in the DTG/3TC versus CAR group, respectively, in participants identifying as White (0% vs <1%), Black (1% vs 2%), and Asian (0% vs 2%; Table 1). Proportion of participants with HIV‐1 RNA <50 copies/mL was high in both treatment groups and all race subgroups (Table 1). High baseline CD4+ cell count was generally maintained across treatment groups and races. Across races, adjusted mean change from baseline to W48 in CD4+/CD8+ ratio was comparable between treatment groups and maintained near baseline (0.9 to 1.2). Proportions of AEs were similar between treatment groups overall and across races, with few withdrawals due to AEs (Table 1). As expected in stable switch settings, drug‐related AEs were more frequent with DTG/3TC versus CAR across race subgroups.

**Table:** 

**Abstract P090 – Table 1**. Week 48 efficacy (ITT‐E population, Snapshot) and safety (safety population^a^) results from the pooled analysis of the TANGO and SALSA trials.								
	White		Black		Asian		Other races^b^	
	DTG/3TC	CAR	DTG/3TC	CAR	DTG/3TC	CAR	DTG/3TC	CAR
Efficacy parameter	(N = 445)	(N = 433)	(N = 96)	(N = 106)	(N = 44)	(N = 52)	(N = 30)	(N = 28)
HIV‐1 RNA ≥50 copies/mL, n (%)	0	2 (<1)	1 (1)	2 (2)	0	1 (2)	1 (3)	0
HIV‐1 RNA <50 copies/mL, n (%)	418 (94)	405 (94)	88 (92)	96 (91)	42 (95)	49 (94)	28 (93)	25 (89)
CD4+ count, cells/mm^3^								
Baseline, mean (SD)	718.3	729.9	747.6	751.5	576.2	577.9	667.1	653.6
	(301.2)	(277.7)	(322.2)	(285.7)	(196.4)	(236.7)	(329.1)	(284.1)
Adjusted mean change (SE)^c^	25.0	‐1.5	42.6	‐12.3	‐30.5	18.2	6.8	‐13.5
	(8.4)	(8.2)	(18.3)	(17.0)	(26.9)	(24.2)	(32.0)	(32.7)
CD4+/CD8+ ratio								
Baseline, mean (SD)	1.1 (0.6)	1.1 (0.5)	1.1 (0.6)	1.2 (0.6)	0.9 (0.4)	0.9 (0.4)	1.0 (0.3)	1.0 (0.4)
Adjusted mean change (SE)^d^	0.039	0.056	0.041	0.045	0.021	0.033	0.027	0.064
	(0.0097)	(0.0104)	(0.0211)	(0.0215)	(0.0308)	(0.0304)	(0.0365)	(0.0412)
	**White**		**Black**		**Asian**		**Other races^b^**	
	**DTG/3TC**	**CAR**	**DTG/3TC**	**CAR**	**DTG/3TC**	**CAR**	**DTG/3TC**	**CAR**
Safety parameter, n (%)	(N = 445)	(N = 432)	(N = 96)	(N = 106)	(N = 44)	(N = 52)	(N = 30)	(N = 28)
Any AE	344 (77)	331 (77)	72 (75)	77 (73)	33 (75)	38 (73)	26 (87)	18 (64)
AEs leading to withdrawal	14 (3)	3 (<1)	2 (2)	2 (2)	2 (5)	0	0	0
Grade 2 to 5 AEs	205 (46)	215 (50)	42 (44)	51 (48)	17 (39)	24 (46)	17 (57)	13 (46)
Drug‐related AEs	60 (13)	9 (2)	16 (17)	3 (3)	8 (18)	8 (15)	9 (30)	1 (4)
Any SAE	18 (4)	18 (4)	6 (6)	9 (8)	3 (7)	4 (8)	1 (3)	1 (4)

3TC, lamivudine; CAR, current antiretroviral regimen; DTG, dolutegravir; MMRM, mixed‐models repeated‐measures.

^a^In TANGO, one participant was found to be taking a TDF‐based regimen and was excluded from the safety population;

^b^includes American Indian or Alaska Native, Native Hawaiian or Other Pacific Islander, mixed White race, and multiple races;

^c^MMRM analysis adjusting for treatment; visit; age; sex; baseline CD4+ cell count; baseline third agent; baseline BMI; race; study (combined analysis only); and treatment‐by‐visit, baseline CD4+ cell count‐by‐visit, visit‐by‐race, treatment‐by‐race, and treatment‐by‐visit‐by‐race interactions, with visit as repeated factor;

^d^MMRM analysis adjusting for treatment; visit; age; sex; baseline CD4+ cell count; baseline third agent; baseline CD4+/CD8+ ratio; baseline BMI; race; study (combined analysis only); and treatment‐by‐visit, baseline CD4+/CD8+ ratio‐by‐visit, visit‐by‐race, treatment‐by‐race, and treatment‐by‐visit‐by‐race interactions, with visit as repeated factor.

**Conclusions**: Switching to DTG/3TC resulted in high rates of maintained virologic suppression in Black and Asian participants, which were similar across races, with maintenance of high CD4+ cell count overall and by race. Withdrawals due to AEs were low and consistent across subgroups. Results show that DTG/3TC is a robust switch option with high efficacy and good safety and tolerability in people with HIV across races.

#### P091 Effectiveness and safety of dolutegravir‐based two‐drug regimens in a multicentre cohort in Spain

B Alejos^1^, I Suárez‐García^2^, L Martín‐Carbonero^3^, A Moreno‐Zamora^4^, R Font^5^, J Portilla^6^, O Martinez^7^, M Omar^8^, D Vinuesa^9^, S Moreno Guillén ^4^, I Jarrin^1^, on behalf of the CoRIS Cohort

^1^CoRIS, Institute of Health Carlos III, Madrid, Spain; ^2^CoRIS, Hospital Universitario Infanta Sofia, Madrid, Spain; ^3^CoRIS, La Paz University Hospital, Madrid, Spain; ^4^CoRIS, Hospital Ramón y Cajal, Madrid, Spain; ^5^CoRIS, Hospital Universitario 12 de Octubre, Madrid, Spain; ^6^CoRIS, Hospital General Universitario de Alicante, Alicante, Spain; ^7^CoRIS, Hospital General Universitario Santa Lucia, Cartagena, Spain; ^8^CoRIS, Hospital Universitario de Jaen, Jaen, Spain; ^9^CoRIS, Hospital Universitario San Cecilio, Granada, Spain

**Background**: We assessed the use, effectiveness and safety of dolutegravir‐based two‐drug regimens in ART‐naïve and virologically suppressed individuals.

**Methods**: We included individuals from the Spanish AIDS Research Network Cohort (CoRIS). We used multivariable logistic regression to calculate odds ratios (OR) for viral suppression (VS) (HIV‐RNA <50 copies/mL) and linear regression for mean differences in CD4 count increases at 24 and 48 weeks from ART initiation, comparing first‐line regimens prescribed in >5% of individuals in real‐world setting from August 2018 to November 2020. We also calculated the proportion of virologically suppressed individuals who maintained VS at 24 and 48 weeks after switching to RPV/DTG and 3TC/DTG from January 2018 to November 2020.

**Results**: The most frequent first‐line regimens among 1524 ART‐naïve individuals analysed are shown in Table 1. The preferred starting regimen changed over time: while ABC/3TC/DTG (34.8%), TDF/FTC+DTG (27.2%) and TAF/FTC/EVG/COBI (24.5%) were the preferred option in 2018, TAF/FTC/BIC (57.4%) and 3TC/DTG (25.7%) were the most frequent options in 2020. At 24 and 48 weeks from initiation, 82.4% and 88.8% of individuals achieved VS, and mean (95% CI) CD4 count increase from initiation was 192.24 (180.73 to 203.75) and 253.23 (239.08 to 267.38) cells/mL, respectively. No significant differences by first‐line regimen were found for immunological and virological response except for a lower chance of VS for TAF/FTC+DRV/COBI compared to 3TC/DTG at 24 weeks (Table 1). At 48 weeks the proportion of discontinuations due to AEs was higher with TDF/FTC+DTG (8.1%) and TAF/FTC/EVG/COBI (5.8%). Among virologically suppressed individuals, the proportion of switchings to RPV/DTG and 3TC/DTG increased from 1% and 7.3% in 2018, to 3.8% and 41.9% in 2020, respectively. At 24 and 48 weeks, 96.1% (74/77) and 90% (45/50) of individuals who switched to RPV/DTG and 97.0% (487/502) and 93.5% (300/321) of those switching to 3TC/DTG maintained VS.

**Table:** 

**Abstract P091 – Table 1**. Results from multivariable analyses on effectiveness to initial ART and treatment discontinuations due to AEs.					
	Treatment discontinuations due to AEs	Immunological response	Immunological response	Viral suppression	Viral suppression
		Adjusted mean difference (95% CI)^a^	Adjusted mean difference (95% CI)^a^	Adjusted OR (95% CI)^a^	Adjusted OR (95% CI)^a^
		24 weeks (n = 1288)	48 weeks (n = 951)	24 weeks (n = 1040)	48 weeks (n = 715)
3TC/DTG (n = 234, 15.35%)	1 (0.4%)	Ref.	Ref.	Ref.	Ref.
TAF/FTC/BIC (n = 580, 38.06%)	9 (1.5%)	30.05 (‐3.99 to 64.09)	21.19 (‐18.33 to 60.72)	1.23 (0.80 to 1.88)	0.73 (0.23 to 2.30)
ABC/3TC/DTG (n = 229, 15.03%)	7 (3.1%)	26.68 (‐10.11 to 63.47)	25.7 (‐22.52 to 73.86)	1.08 (0.56 to 2.11)	0.78 (0.26 to 2.37)
TAF/FTC+DRV/COBI (n = 127, 8.33%)	3 (2.4%)	3.89 (‐42.89 to 50.68)	3.20 (‐59.42 to 65.81)	0.55 (0.34 to 0.89)	0.63 (0.17 to 2.25)
TAF/FTC/EVG/COBI (n = 120, 7.87%)	7 (5.8%)	14.72 (‐28.56 to 58.00)	41.386 (‐5.00 to 87.77)	1.11 (0.53 to 2.34)	1.97 (0.48 to 8.04)
TDF/FTC+DTG (n = 234, 15.35%)	19 (8.1%)	43.13 (‐9.81 to 96.07)	35.39 (‐28.63 to 99.40)	1.27 (0.61 to 2.63)	0.95 (0.26 to 3.50)
Overall p‐value	<0.001	0.381	0.213	0.006	0.424

3TC, lamivudine; ABC, abacavir; BIC, bictegravir; COBI, cobicistat; DRV, darunavir; DTG, dolutegavir; FTC, emtricitabine; TAF, tenofovir alafenamide; TDF, tenofovir.

^a^Adjusted by sex, age at ART initiation, transmission category, educational level, country of origin, CD4 T‐cell count and viral load within 6 months prior to ART initiation, presence of hepatitis C virus antibodies, presence of hepatitis B virus surface antigen and AIDS diagnosis at start of ART.

**Conclusions**: In naïve individuals, the effectiveness and tolerability of 3TC/DTG was similar to other first‐line ART regimens. Switching to RPV/DTG or 3TC/DTG in virologically suppressed individuals did not seem to impair its effectiveness.

**Figure:**
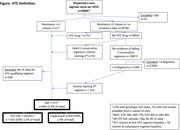
**Abstract P092 – Figure 1**. HTE definition

#### P092 Characteristics of heavily treatment‐experienced (HTE) people with HIV (PWHIV) in US clinical practice

J Eron^1^, M Dunbar^2^, J Radtchenko ^3^, J Gruber^2^, G Huhn^4^, G McComsey^5^, L Temme^6^, J Thompson^7^, R Elion^3^

^1^Department of Medicine, Division of Infectious Diseases, University of North Carolina at Chapel Hill, Chapel Hill, NC, USA; ^2^Medical Affairs Research ‐ Virology, Gilead Sciences, Foster City, CA, USA; ^3^Analytics, Trio Health, Louisville, CO, USA; ^4^The Ruth M. Rothstein Core Center, Rush University Medical Center, Chicago, IL, USA; ^5^HIV, University Hospitals Cleveland Medical Center and Case Western Reserve University, Cleveland, OH, USA; ^6^HIV Medical Affairs, Gilead Sciences, Foster City, CA, USA; ^7^HIV, Amity Medical Group, Charlotte, NC, USA

**Background**: Identification of HTE PWHIV in clinical settings is challenging due to disparate definitions. This study characterized PWHIV in clinical practice meeting various HTE criteria, focusing on demographic and clinical characteristics including virologic control at baseline of HTE‐qualifying antiretroviral (ART) regimen.

**Methods**: In Trio Health EMR and dispensing data we identified HTE PWHIV ≥18 years dispensed ART after January 2015 with viral load within 12 months of first HTE‐qualifying regimen using two definitions. Definition 1: resistance ≥2 classes OR drug used in HTE setting (maraviroc [MVC], etravirine [ETR], ibalizumab [IBA], enfuvirtide [ENF], fostemsavir [FTR], dolutegravir [DTG] BID or darunavir [DRV] BID) OR failed two consecutive regimens and viremic starting third OR viremic starting fourth regimen. Definition 2: above limited to viremic at HTE baseline. Univariate comparisons of virally suppressed (VS) PWHIV from definition #1 versus PWHIV from definition #2 were conducted via chi‐square for categorical and t‐test for continuous variables.

**Results**: Of 29 844 patients, 865 (3%) satisfied definition 1 and 435 (1.5%) satisfied definition 2 (Figure 1).  Of HTE definition 1, 53% were age >50, 73% male, 46% black, 35% commercially insured. Only 24% had historic genotypic tests; of those with results, 8% were resistant to ≥2 classes. Clinically, 20% presented with baseline CD4 <200 cells/mm^3^, 50% with normal eGFR, 70% with normal ALT; 26% were viremic on all regimens (Table 1). Half of definition 1 PWHIV were VS at baseline HTE‐qualifying regimen (n = 430).  Viremic patients (definition 2) were more likely to have historic genotypic tests, to be female, black, age 26 to 50, on Medicaid, underweight, with baseline CD4 <200 cells/mm^3^, normal eGFR and ALT. These patients received more prior regimens, but fewer once qualified as HTE, and had shorter time on first HTE‐qualifying regimen with more failures. Definition 2 PWHIV were less likely to receive FTR, ETR, MVC, DRV BID, more likely to be taking BIC‐ and DTG‐containing regimens.

**Conclusions**: HTE PWHIV represent a small but important population. HTE PWHIV viremic at baseline differ from VS PWHIV by baseline characteristics and treatment patterns and are more likely to fail in HTE setting. Genotype testing is underutilized among HTE PWHIV.

**Table:** 

**Abstract P092 – Table 1**. HTE characteristics by definition.				
HTE characteristics by definition	N (%) unless specified	HTE definition 1 total n = 865	HTE definition 2 viremic at HTE baseline n = 435	Suppressed at HTE baseline n = 430
Patient age	18 to 25	27 (3)	15 (3)	12 (3)
	26 to 50	383 (44)	226 (52)^a^	157 (37)
	>50	455 (53)	194 (45)	261 (61)^a^
Gender	Male	634 (73)	305 (70)	329 (77)^b^
	Female	164 (19)	101 (23)^b^	63 (15)
Race	White	384 (44)	161 (37)	161 (37)
	Black	402 (46)	241 (55)^a^	159 (37)
Payer	Commercial	305 (35)	146 (34)	159 (37)
	Medicare	184 (21)	76 (17)	108 (25)^b^
	Medicaid	182 (21)	118 (27)^a^	64 (15)
Baseline CD4	<200 cells/mm^3^	172 (20)	125 (29)^a^	47 (11)
Baseline BMI	Underweight <18.5 kg/m^2^	25 (3)	18 (4)^b^	7 (2)
	Overweight 25 to 30 kg/m^2^	284 (35)	128 (32)	156 (39)^b^
Baseline eGFR (mL/min/1.73 m^2^)	60 to 89	306 (38)	139 (32)	167 (43)^b^
	90+	409 (50)	242 (56)^a^	167 (43)
Baseline ALT	> upper limit of normal (ULN)	243 (30)	109 (26)	134 (35)^b^
Follow‐up (yrs)	Mean (SD)	7.9 (4.4)	7.9 (4.2)	7.8 (4.6)
Follow‐up since HTE baseline start (mo)	Mean (SD)	39.4 (24.2)	34.1 (22)	44.8 (25.2)^a^
Prior known regimens	Mean (SD)	1.3 (1.5)	2.1 (1.5)^a^	0.5 (1.1)
HTE regimens	Mean (SD)	1.9 (1.2)	1.8 (1.2)	2 (1.3)^b^
Duration of baseline HTE regimen (mo)	Mean (SD)	14.5 (15.6)	13.3 (13.5)	15.8 (17.4)^b^
Suppressed at last available observation on baseline HTE regimen	(<200 copies/mL)	372 (68)	143 (49)	229 (90)^a^
Failure at any time on baseline regimen	(2 consecutive viral loads >200 copies/mL)	152 (18)	115 (26)^a^	37 (9)
Viremic on all HTE regimens	(<200 copies/mL at last observation)	158 (26)	132 (42)^a^	26 (9)
First HTE regimen	Ibalizumab‐containing (IBA)	2 (0)	0 (0)	2 (0)
	Fostemsavir (FTR)	17 (2)	3 (1)	14 (3)^b^
	Maraviroc (MVC)	147 (17)	30 (7)	117 (27)^a^
	Etravirine (ETR)	317 (37)	75 (17)	242 (56)^a^
	Enfuvirtide (ENF)	2 (0)	1 (0)	1 (0)
	Bictegravir (BIC)	84 (10)	77 (18)^a^	7 (2)
	Dolutegravir + rilpivirine (DTG + RPV)	164 (19)	88 (20)	76 (18)
	DTG (not including DTG+RPV)	111 (13)	68 (16)^b^	43 (10)
	DTG twice daily (BID)	63 (7)	33 (8)	30 (7)
	Darunavir (DRV)	281 (32)	125 (29)	156 (36)^b^
	DRV BID	93 (11)	32 (7)	61 (14)^b^
	DRV+ DTG BID	13 (2)	9 (2)	4 (1)
With historic genotypic test		208 (24)	137 (31)^a^	71 (17)
	With genotypic result available	113 (13)	79 (18)^a^	34 (8)
	Resistant to ≥2 classes	9 (8)	5 (6)	4 (12)

^a^p < 0.001; ^b^0.001 ≤ p < 0.05.

p‐values are for viremic vs suppressed at baseline of HTE‐qualifying regimen.

Variable categories are limited to those with significant differences (p < 0.05).

#### P093 Efficacy and safety of switching to dolutegravir plus rilpivirine in virologically suppressed older PLWHIV: pooled week 148 results from SWORD‐1 and SWORD‐2

M Prakash ^1^, R Grove^2^, B Wynne^3^, J van Wyk^4^, B Jones^4^, A Clark^4^

^1^Global Scientific Affairs, ViiV Healthcare, Brentford, UK; ^2^Statistics, GSK, Brentford, UK; ^3^Clinical Development, ViiV Healthcare, Durham, NC, USA; ^4^Global Medical Affairs, ViiV Healthcare, Brentford, UK

**Background**: As older adults are among the fastest growing populations living with HIV, their inclusion in clinical trials is important. Among adults in the SWORD studies, switching to the two‐drug regimen dolutegravir plus rilpivirine (DTG + RPV) demonstrated non‐inferiority in maintaining virologic suppression versus continuing three‐ or four‐drug regimens at week 48 and maintained high levels of virologic suppression through week 148. We present pooled SWORD‐1 and SWORD‐2 efficacy and safety results analyzed by age (<50, ≥50 to <65, and ≥65 years).

**Materials and methods**: In the open‐label, phase III SWORD studies, virologically suppressed adults were randomized to switch to once‐daily DTG + RPV on day 1 (Early‐Switch [ES] group) or to continue their current antiretroviral regimen and switch to DTG + RPV at week 52 (Late‐Switch [LS] group). Proportions of participants with HIV‐1 RNA <50 copies/mL (Snapshot, ITT‐E) and safety were analyzed through week 148.

**Results**: Among participants switching to DTG + RPV (ES, n = 513; LS, n = 477), 72% were aged <50 years, 25% aged ≥50 to <65 years, and 3% aged ≥65 years. Through week 148, proportions of participants with HIV‐1 RNA <50 copies/mL were high across all ages in both groups. Of note, the lower response observed in participants aged ≥65 years (ES, 72%; LS, 69%) was mostly driven by no virologic data and low numbers of participants (ES, 28%, n = 18; LS, 15%, n = 13; Table 1). Mean CD4+ cell count was high (>500 cells/mm^3^) at baseline and maintained through week 148 across ages and treatments. Overall, similar proportions of AEs were observed in both treatment groups; all participants aged ≥65 years reported AEs. Low numbers of participants reported AEs leading to withdrawal (<50, ≥50 to <65, and ≥65 years, respectively: ES group, 8%, 8%, and 11%; LS group, 3%, 5%, and 15%). Drug‐related AEs occurred more frequently in the ES group (<50, ≥50 to <65, and ≥65 years, respectively: 19%, 20%, and 33%) versus the LS group (13%, 16%, and 8%).

**Conclusions**: Through 148 weeks, DTG + RPV maintained high rates of virologic suppression in all individuals irrespective of age after switch and demonstrated a good safety profile.

**Table:** 

**Abstract P093 – Table 1**. Summary of Snapshot analysis at weeks 48, 100, and 148 in the pooled SWORD studies (ITT‐E population).					
	ES group (N = 513)			LS group (N = 477)	
	DTG + RPV	DTG + RPV	DTG + RPV	DTG + RPV	DTG + RPV
n (%)	Weeks 1 to 48	Weeks 1 to 100	Weeks 1 to 148	Weeks 52 to 100	Weeks 52 to 148
HIV‐1 RNA <50 copies/mL					
<50 years	350/366 (96)	332/366 (91)	316/366 (86)	321/344 (93)	311/344 (90)
≥50 to <65 years	118/129 (91)	108/129 (84)	103/129 (80)	113/120 (94)	108/120 (90)
≥65 years	18/18 (100)	16/18 (89)	13/18 (72)	11/13 (85)	9/13 (69)
HIV‐1 RNA ≥50 copies/mL					
<50 years	1/366 (<1)	8/366 (2)	9/366 (2)	7/344 (2)	6/344 (2)
≥50 to <65 years	2/129 (2)	5/129 (4)	5/129 (4)	0/120	3/120 (3)
≥65 years	0/18	0/18	0/18	1/13 (8)	2/13 (15)
No virologic data					
<50 years	15/366 (4)	26/366 (7)	41/366 (11)	16/344 (5)	27/344 (8)
≥50 to <65 years	9/129 (7)	16/129 (12)	21/129 (16)	7/120 (6)	9/120 (8)
≥65 years	0/18	2/18 (11)	5/18 (28)	1/13 (8)	2/13 (15)

#### P094 Doravirine plus lamivudine (DOR/3TC) two‐drug regimen as a maintenance antiretroviral therapy in controlled HIV‐infected patients

P Perfezou^1^, N Hall^1^, J Duthe^1^, B Abdi^2^, S Seang^3^, A Marcelin^2^, C Katlama^3^, R Palich ^3^

^1^Infectious Diseases, Quimper Hospital, Quimper, France; ^2^Virology, Sorbonne University, Pitié‐Salpêtrière Hospital, Paris, France; ^3^Infectious Diseases, Sorbonne University, Pitié‐Salpêtrière Hospital, Paris, France

**Background**: Two‐drug regimens are increasingly used as maintenance strategies. They are all based on INSTIs or boosted PIs. Taking advantage of the high genetic barrier, the good tolerance and the absence of drug‐drug interaction of doravirine, we aimed to report here our experience with doravirine/lamivudine (DOR/3TC) for maintaining the viral suppression.

**Methods**: This observational study enrolled all adults who initiated DOR/3TC between 09/01/2019 and 10/31/2021, in two French hospitals. The primary outcome was the rate of virological success (no virological failure [VF]: confirmed HIV‐RNA ≥50 copies/mL or single HIV‐RNA ≥200 copies/mL, or ≥50 copies/mL with ART change) at week (W) 48. Secondary outcomes included: virological and strategy success rates (HIV‐RNA <50 copies/mL with no ART change), evolution of CD4 count and CD4/CD8 ratio over follow‐up.

**Results**: Forty‐three patients were included, with 29 (67%) men, median age: 59 years (IQR 52 to 63), ART duration: 21 years (14 to 25), duration of virological suppression: 14 years (8 to 19), CD4 count: 616/mm^3^ (774 to 878). ART prior to DOR/3TC was NNRTI‐based three‐drug regimen in 20 (46%) patients, INSTI‐based three‐drug regimen in 10 (23%), INSTI‐ or boosted PI‐based two‐drug regimen in 11 (26%), and boosted PI monotherapy in two (5%). All had HIV‐RNA <50 copies/mL at study entry. One had a past M184V mutation. All except one were naive to doravirine before switching. Median follow‐up was 51 weeks (IQR 33 to 70). The virological success rate was 97.7% (95% CI 87.7 to 99.9) and the strategy success rate was 93.0% (95% CI 80.9 to 98.5) at W48. One VF occurred at W18 (HIV‐RNA = 101 copies/mL), in a patient having briefly stopped his treatment due to intense nightmares; no resistance at baseline; no resistance emergence; HIV‐RNA <50 copies/mL after resumption of boosted PI monotherapy. There were two strategy discontinuations over the entire study period for adverse event (digestive disorder: n = 1; neuropsychic disorder: n = 1). There was no significant change in the CD4 count and CD4/CD8 ratio over follow‐up.

**Conclusion**: This preliminary observational study positions DOR/3TC as a promising two‐drug regimen, able to sustain a high virological success rate, in highly experienced patients with sustained viral suppression. A prospective trial is now needed to confirm these results.

#### P095 Performance of dolutegravir‐based two‐drug regimens (DTG‐2DR) in a large real‐world cohort of people with HIV

C Bowman, A Ambrose, P Simoes, K Florman, T Kanitkar, A Katiyar, A Hunter, J Hart, T Barber

HIV Medicine, Royal Free Hospital, London, UK

**Background**: Evidence supports DTG‐2DR use in appropriate people [1‐3]. In our centre regimens include DTG/3TC, DTG/RPV, DTG/FTC (multiple tablet regimens (MTR) until single tablet regimens (STR) available). Since 2015 we prescribed DTG‐2DR for 620 people (total cohort 3133 (19.8%)). Nine ART‐naïve people initiated; remainder switched or continued.

**Method**: Clinic database search 01/01/15 to 31/10/21 conducted for all receiving DTG‐2DR. Demographic, tolerability and HIV‐related data were analysed.

**Results**: Six hundred and twenty people identified; 561 had complete data. Four hundred and forty‐six male (79.5%); median age 54 years (IQR 46 to 59). Three hundred and forty‐three (61.1%) MSM. Median time to DTG‐2DR from diagnosis 16 years. Nine initiated naïvely became undetectable and remained suppressed (100%). Five hundred and forty‐six of 552 switched (99.0%) remained suppressed at data censor. Five hundred and thirty‐seven had a VL <50 at switch, 14 had a VL >50 at switch or initiation. Seventy‐four of 552 (13.4%) received DTG/RPV. Four hundred and eighty‐three of 552 (86.6%) DTG/XTC. Four (0.7%) died, but cause of death not related to HIV. Seventy (12.5%) switched off DTG‐2DR (13 DTG/RPV, 57 DTG/XTC); top three reasons insomnia 13 (18.6%), psychiatric (low mood 12 (17.1%)), or weight gain 10 (14.3%). Forty‐one episodes of blip (one off >50 copies/mL) occurred in 30 people (30/561, 5.3%). Eleven of 41 on DTG‐RPV (n = 7 MTR, n = 4 STR). Thirty of 41 on DTG‐XTC (n = 26 MTR, n = 4 STR). These prompted switch to alternate regimens in five; the remainder resuppressed on regimen. Six people (1.1%) encountered failure (confirmed VL >200 copies/mL or persistent LLV) (n = 4 DTG‐3TC STR, n = 1 DTG‐3TC MTR, n = 1 DTG‐RPV MTR). Four failures were at LLV only and rapidly resuppressed on switch. One failure due to non‐adherence and the individual switched to triple therapy. One failure was on DTG‐3TC MTR to higher VL. Resistance tests performed for 5/6. No treatment associated mutations were detected bar latter person with high VL failure on MTR DTG/3TC who had developed triple class resistance (further details to be presented).

**Conclusion**: Majority of DTG/3TC use is in stable switch. A minority of patients switch due to tolerability. Low number of virological failures noted, though one developed INI resistance; VF associated with MTR and it is imperative switch to STR occurs when available, commensurate with trial data showing no failure with resistance if DTG/3TC STR used. Overall DTG‐2DR demonstrates high efficacy in a real‐world setting.

**References**

1. van Wyk J, Ajana F, Bisshop F, De Wit S, Osiyemi O, Portilla Sogorb J, et al. Efficacy and safety of switching to dolutegravir/lamivudine fixed‐dose 2‐drug regimen vs continuing a tenofovir alafenamide–based 3‐ or 4‐drug regimen for maintenance of virologic suppression in adults living with human immunodeficiency virus type 1: phase 3, randomized, noninferiority TANGO study. Clin Infect Dis. 2020;71:1920‐9.

2. Llibre JM, Brites C, Cheng C‐Y, Osiyemi O, Galera C, Hocqueloux L, et al. Efficacy and safety of switching to the 2‐drug regimen dolutegravir/lamivudine versus continuing a 3‐ or 4‐drug regimen for maintaining virologic suppression in adults living with HIV‐1: week 48 results from the phase 3, non‐inferiority SALSA randomized trial. Clin Infect Dis. 2022 Mar 2;ciac130. doi: 10.1093/cid/ciac130.

3. Aboud M, Orkin C, Podzamczer D, Bogner JR, Baker D, Khuong‐Josses M‐A, et al. Efficacy and safety of dolutegravir‐rilpivirine for maintenance of virological suppression in adults with HIV‐1: 100‐week data from the randomised, open‐label, phase 3 SWORD‐1 and SWORD‐2 studies. Lancet HIV. 2019;6:e576‐87.

#### P096 Long‐acting combination of cabotegravir plus rilpivirine: a picture of potential eligible HIV‐positive individuals from the Italian ARCA cohort

A Cervo ^1^, A Russo^2^, D Di Carlo^3^, A De Vito^4^, L Fabeni^5^, S D'Anna^6^, L Duca^6^, A Colpani^4^, M Fois^4^, B Zauli^4^, G Mancarella^7^, A Carraro^7^, A Bezenchek^8^, A Cozzi‐Lepri^9^, M Santoro^6^

^1^Infectious Diseases Clinic, University Hospital of Modena, Modena, Italy; ^2^Infectious Diseases Unit, Department of Mental Health, University of Campania 'Luigi Vanvitelli', Naples, Italy; ^3^Dept of Biomedical & Clinical Sciences 'L. Sacco', University of Milan, Pediatric Clinical Research Center 'Romeo and Enrica Invernizzi', Milan, Italy; ^4^Unit of Infectious Diseases, Department of Medical, Surgical, and Experimental Sciences, University of Sassari, Sassari, Italy; ^5^L. Spallanzani, National Institute for Infectious Diseases, Rome, Italy; ^6^Tor Vergata, University of Rome, Rome, Italy; ^7^Unit of Infectious Diseases, Sapienza University of Rome ‐ Polo Pontino, Latina, Italy; ^8^EuResist Network GEIE, InformaPRO SRL, Rome, Italy; ^9^Centre for Clinical Research, Epidemiology, Modelling and Evaluation, Institute for Global Health, London, UK

**Background**: The aim of this study was to compare HIV‐1 positive individuals under virological control potentially eligible for the recently approved regimen with long‐acting (LA) cabotegravir (CAB) and rilpivirine (RPV) with those ineligible [1,2].

**Figure:**
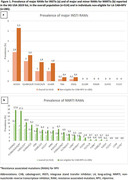
**Abstract P096 – Figure 1**. Prevalence of major RAMs for INSTIs (a) and of major and minor RAMs for NNRTIs (b) reported in the IAS‐USA 2019 list, in the overall population (n = 514) and in individuals ineligible for LA CAB+RPV (n = 285). * Resistance associated mutations (RAMs) for RPV. CAB, cabotegravir; INSTI, integrase strand transfer inhibitor; LA, long‐acting; RAM, resistance associated mutation; RPV, rilpivirine.

**Materials and methods**: This was an observational, cross‐sectional study from ARCA database including HIV‐positive adults with at least two consecutive viraemia <50 copies/mL after 1 January 2019 and at least one genotypic resistance testing (GRT) for RT/INT from plasma and/or PBMCs. Eligibility criteria for LA CAB+RPV were: negative HBsAg, absence of resistance‐associated mutations (RAMs) for NNRTIs, of major RAMs for INSTIs (IAS‐USA list 2019), and of previous virological failures (VFs) to INSTIs and/or NNRTIs [1‐3]. Prevalence of eligible individuals was calculated. Univariable analysis was performed to investigate potential differences between eligible and ineligible individuals.

**Results**: Five hundred and fourteen individuals were included: 377 (73.3%) were male, median age was 51 (IQR 43 to 58), 41 (8%) had HBV co‐infection, in ART from 9 years (IQR 4 to 17) and in virological suppression from 63 months (IQR 34.7 to 105.2) (Table 1). One hundred and nineteen (23%), 134 (26%), and 17 (3%) individuals experienced VFs to INSTIs, NNRTIs and RPV, respectively. B subtype was detected in 382 (74%) individuals. Thirty‐three (6%), 123 (24%) and 104 (20%) individuals had at least one major RAM for INSTIs, for NNRTIs (excluded RPV) and for RPV, respectively. The most common major RAMs were showed in Figure 1. Among 24 ineligible individuals with GRT on both RNA and DNA, one had RAMs for NNRTI only on DNA and three had previous VFs without any RAMs. Eligible individuals for LA CAB+RPV were 229 (44.5%, 95% CI 40.8 to 48.8): compared to ineligible individuals, they were younger, injected drugs less frequently, had a lower zenith viraemia (4.5 vs 5.1 log10 copies/mL) and higher CD4 count nadir (260 vs 170 cells/mm^3^)(Table 1). They had a more recent HIV diagnosis (2012 vs 2002) as well as year of ART‐start (2015 vs 2007), receiving a lower number of previous regimens (3 vs 6).

**Conclusions**: Less than half of virosuppressed HIV‐positive individuals with available GRTs in ARCA cohort were potentially eligible for LA CAB+RPV. They showed a lower zenith viraemia, higher CD4 cell count, a shorter history of HIV infection and exposure to ART compared to those ineligible to LA CAB+RPV.

**Table:** 

**Abstract P096 – Table 1**. Demographic, clinical, therapeutic and virological characteristics of the overall population and in individuals eligible and ineligible for LA CAB+RPV. Potential differences between eligible and ineligible individuals to CAB+RPV were evaluated by T‐test or Mann‐Whitney exact test for quantitative variables and Chi‐squared/Fisher's tests for qualitative variables, as appropriate.				
Variables	Overall population (n = 514)	Non‐eligible for CAB+RPV (n = 285)	Eligible for CAB+RPV (n = 229)	p‐value
Gender male, n (%)	377 (73.3)	198 (69.5)	179 (78.2)	0.034
Age (years), median (IQR)	51 (43 to 58)	54 (46 to 58)	48 (38 to 55)	<0.001
Ethnicity, n (%)				
Caucasian	314 (60.0)	169 (59.3)	145 (63.3)	0.353
Black	36 (7.0)	25 (8.7)	11 (4.8)	0.114
Other/unknown	164 (33.0)	91 (32.0)	73 (31.9)	0.597
HIV‐1 subtype, n (%)				
B	382 (74.3)	225 (78.9)	157(68.6)	0.007
A^a^	19 (3.7)	6 (2.1)	13 (5.7)	0.058
CRF02_AG	43 (8.4)	24 (8.4)	19 (8.3)	1.000
CRFs_BC	18 (3.5)	2 (0.7)	16 (7.0)	<0.001
CRFs_BF	11 (2.1)	6 (2.1)	5 (2.2)	1.000
Others	41 (8.0)	22 (7.7)	19 (8.3)	0.939
HIV‐1 risk factor, n (%)				
Heterosexual	212 (41.2)	118 (41.4)	94 (41.1)	0.935
MSM	121 (23.5)	57 (20.0)	64 (27.9)	0.035
Drugs injection	96 (18.7)	75 (26.3)	21 (9.2)	<0.001
Other/unknown	85 (16.5)	35 (12.3)	50 (21.8)	0.005
VL zenith (log10 copies/mL), median (IQR)	4.9 (4.0 to 5.5)	5.1 (4.4 to 5.7)	4.5 (3.1 to 5.2)	<0.001
CD4 count nadir (cells/mm^3^), median (IQR)	210 (80 to 370)	170 (50 to 320)	260 (120 to 450)	<0.001
Year of HIV‐1 diagnosis, median (IQR)	2008 (1995 to 2014)	2002 (1991 to 2011)	2012 (2006 to 2016)	<0.001
HCV co‐infection, n (%)	65 (12.6)	53 (18.6)	12 (5.2)	<0.001
HBV co‐infection, n (%)	41 (8.0)	41 (14.4)	0 (0.0)	−
First therapy (year), median (IQR)	2011 (2003 to 2016)	2007 (1997 to 2014)	2015 (2010 to 2017)	<0.001
Years from first therapy, median (IQR)	9 (4 to 17)	13 (6 to 23)	6 (3 to 10)	<0.001
Previous drug classes experienced, n (%)				
NRTI	498 (98.6)	276 (97.9)	222 (99.6)	0.14
NNRTI	315 (62.4)	194 (68.8)	121 (54.3)	<0.001
PI	349 (69.1)	227 (80.5)	122 (54.7)	<0.001
INSTI	388 (76.8)	244 (86.5)	144 (64.6)	<0.001
MVC	35 (6.9)	28 (9.9)	7 (3.1)	0.005
T20	17 (3.4)	17 (6.0)	0 (0.0)	<0.001
No. of previous therapies, median (IQR)	4 (2 to 7)	6 (3 to 11)	3 (2 to 4)	< 0.001
Therapy at last viraemia, n (%) (n = 497)				
2 NRTI + 1 INSTI	179 (36.0)	101 (36.6)	78 (35.6)	0.869
2 NRTI + 1 NNRTI	106 (21.3)	32 (11.5)	74 (33.8)	<0.001
2 NRTI + 1 PI	62 (12.5)	38 (13.7)	24 (11.0)	0.441
1 NRTI + 1 INSTI	39 (7.8)	16 (5.8)	23 (10.5)	0.074
Other	111 (22.3)	91 (32.7)	20 (9.1)	<0.001
Previous VFs, n (%)				
NNRTI	136 (26.4)	136 (47.7)	−	−
RPV	17 (3.3)	17 (6.0)	−	−
INSTI	119 (23.2)	119 (41.8)	−	−
Time from last VF (months), median (IQR)	63 (34.7 to 105.2)	62.1 (34 to 102)	64.4 (36.2 to 107.6)	0.583
At least one major RAM for INSTI, n (%)	33 (6.4)	33 (11.6)	0 (0)	−
At least one RAM for NNRTI, n (%)	168 (32.7)	168 (58.9)	0 (0)	−
At least one RAM for RPV, n (%)	104 (20.2)	104 (36.5)	0 (0)	−
CAB + RPV cumulative GSS, median (IQR)	2 (1.5 to 2)	1.5 (1 to 2)	2 (2 to 2)	<0.001

CAB, cabotegravir; GSS, genotypic susceptibility score (according to Stanford algorithm; HIVdb version 9.0, https://hivdb.stanford.edu/); INSTI, integrase strand transfer inhibitor; IQR, interquartile range; LA, long‐acting; MVC, maraviroc; PI, protease inhibitor; RAM, resistance associated mutation; RPV, rilpivirine; VF, virological failure; VL, viral load.

^a^Subtype A1/A6: n = 17; subtype A7: n = 2.

**References**

1. European AIDS Clinical Society. EACS Guidelines [Internet]. [cited 2022 Jan 22]. Available from: https://www.eacsociety.org/guidelines/eacs‐guidelines/.

2. Panel on Antiretroviral Guidelines for Adults and Adolescents. Guidelines for the Use of Antiretroviral Agents in Adults and Adolescents with HIV. Department of Health and Human Services; I 36‐38. [cited 2022 Jan 22]. Available from: https://clinicalinfo.hiv.gov/sites/default/files/guidelines/documents/AdultandAdolescentGL.pdf.

3. Cutrell AG, Schapiro JM, Perno CF, Kuritzkes DR, Quercia R, Patel P, et al. Exploring predictors of HIV‐1 virologic failure to long‐acting cabotegravir and rilpivirine: a multivariable analysis. AIDS. 2021;35:1333‐42.

#### P097 Switch to bictegravir in real‐life settings in the ANRS‐CO3‐AquiVIH‐NA cohort

O Leleux^1^, A Peyrouny‐Mazeau^1^, A Perrier^1^, M Hessamfar^2^, G Le Moal^3^, D Neau^4^, H Wille^5^, E Lazaro^2^, B Castan^6^, P Duffau^2^, C Cazanave^4^, V Gaborieau^7^, L Wittkop^1^, F Bonnet ^2^

^1^Centre de Méthodologie et de Gestion des Essais Clinique, Unité Mixte de Recherche, Centre d'Investigation Clinique‐EC, Université de Bordeaux, Institut de Santé Publique, d'Epidémiologie et de Développement, Inserm Bordeaux Population Health, Bordeaux, France; ^2^Service de Médecine Interne et Maladies Infectieuses, Centre Hospitalier Universitaire (CHU) de Bordeaux, Bordeaux, France; ^3^Service de Maladies Infectieuses et Tropicales, Centre Hospitalier Universitaire (CHU) de Poitiers, Poitiers, France; ^4^Service de Maladies Infectieuses et Tropicales, Centre Hospitalier Universitaire (CHU) de Bordeaux, Bordeaux, France; ^5^Service de Maladies Infectieuses, Centre Hospitalier de la Côte Basque, Bayonne, France; ^6^Service de Maladies Infectieuses et Tropicales, Centre Hospitalier de Périgueux, Périgueux, France; ^7^Service de Maladies Infectieuses, Centre Hospitalier de Pau, Pau, France

**Background**: Bictegravir (BIC) is an unboosted integrase strand‐transfer inhibitor (InSTI) co‐formulated with the NRTI emtricitabine (FTC) and the N(t)RTI tenofovir alafenamide (TAF) into a single tablet for once‐daily use. This B/F/TAF single tablet regimen is a safe, effective, convenient, and well‐tolerated regimen for people living with HIV (PLWHIV). However, data are lacking regarding the durability, long‐term efficacy and tolerability of this regimen in all subgroups of PLWHIV.

**Materials and method**: Patients included in the prospective ANRS‐CO3‐AquiVIH‐NA cohort were included in the present analysis if they had received BIC/FTC/TAF at least once during their follow‐up.

**Results**: Since 2018, 1927 PLWHIV have received at least once BIC/FTC/TAF of which 1433 after their inclusion in the cohort with CD4 count and HIV RNA available at the beginning of BIC/FTC/TAF regimen. Among them, 46 PLWHIV were naïve, 204 were experiencing treatment failure at BIC/FTC/TAF start, and 1183 were treatment experienced and virologically suppressed. In these 1183 PLWHIV (women 26%) who switched to BIC/FTC/TAF, median age was 53 years, median BMI was 24 and CD4 count 691 cells/mm^3^ [IQR 490 to 910]. Four hundred and two (34%) had a history of at least one virological failure before switch and the median number of therapeutic lines was 5 [IQR 3 to 8]. The most frequent prior regimen before switch to BIC/FTC/TAF were EVG/COBI/FTC/TAF (36%), DRV+RTV+FTC/TDF (9%), DTG+FTC/TDF (7%), DTG/3TC/ABC (6%), RPV/FTC/TAF (6%). Thirty‐seven percent of patients were taking TDF‐containing regimens before BIC/FTC/TAF. The most frequent reasons for switching to BIC/FTC/TAF were simplification (64%), side effects (12%) and drug‐drug interactions (6%). At 18 months of follow‐up, the cumulative probability of BIC/FTC/TAF discontinuation was 18% [IQR 16% to 20%] because of side‐effects (37%), patient's choice (15%) or physician choice (13%), drug reducing (12%), death (9%), pregnancy (3%), and treatment failure (3%). At 18 months, 72 patients (7%) experienced of HIV RNA between 50 and 200 copies/mL at least once and 25 (3%) an increase >200 copies/mL.

**Conclusion**: In this large observational study, more than 82% of patients who switched to BIC/FTC/TAF remained on treatment at M18. Virological failure was observed in 3% of patients. The switch to BIC/FTC/TAF in virologically suppressed patients is a safe and effective strategy.

#### P098 Effectiveness of bictegravir/emtricitabine/tenofovir alafenamide (BIC/FTC/TAF) as switch strategy in virologically suppressed: real‐world data from the ICONA cohort

A d'Arminio Monforte ^1^, A Tavelli^2^, A Cingolani^3^, L Taramasso^4^, C Mussini^5^, S Piconi^6^, A Calcagno^7^, G Orofino^8^, S Cicalini^9^, A Castagna^10^, F Ceccherini‐Silberstein^11^, A Gori^12^, G Guaraldi^5^, A Antinori^9^

^1^Department of Health Sciences, Clinic of Infectious Diseases, ASST Santi Paolo e Carlo, University of Milan, Milan, Italy; ^2^Icona Foundation, Milan, Italy; ^3^Department of Safety and Bioethics, Infectious Diseases Unit, Fondazione Policlinico Universitario Agostino Gemelli IRCCS, Catholic University of the Sacred Heart, Rome, Italy; ^4^Unit of Infectious Diseases, Ospedale Policlinico San Martino IRCCS, University of Genoa, Genoa, Italy; ^5^Infectious and Tropical Diseases Unit, Azienda Ospedaliero Universitaria di Modena, University of Modena and Reggio Emilia, Modena, Italy; ^6^Infectious Diseases Unit, ASST Lecco, Lecco, Italy; ^7^Unit of Infectious Diseases, Department of Medical Sciences, Ospedale Amedeo di Savoia, University of Turin, Turin, Italy; ^8^Division A of Infectious Diseases, Ospedale Amedeo di Savoia, ASL Città di Torino, Turin, Italy; ^9^Clinical Department, HIV/AIDS Unit, National Institute for Infectious Diseases Lazzaro Spallanzani IRCCS, Rome, Italy; ^10^Infectious Diseases Unit, IRCCS San Raffaele Scientific Institute, Vita‐Salute San Raffaele University, Milan, Italy; ^11^Department of Experimental Medicine, University of Rome Tor Vergata, Rome, Italy; ^12^Department of Pathophysiology and Transplantation, Clinic of Infectious Diseases, IRCCS Ca' Granda Ospedale Maggiore Policlinico, University of Milan, Milan, Italy

**Background**: Real‐life clinical data, especially from key populations, is still lacking for BIC/FTC/TAF. Aim of this study is to evaluate the effectiveness of BIC/FTC/TAF in ART‐experienced virologically suppressed (VS) people living with HIV (PLWHIV).

**Figure:**
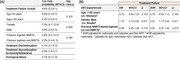
**Abstract P098 – Figure 1**. (A) Kaplan‐Meier estimated 1‐year probability of TF, TD for any reason, TD toxicity/intolerance and VF, overall and in the different groups for primary‐endpoint virologically suppressed ART‐experienced; (B) hazard ratios (HR) and adjusted hazard ratios (AHR) of TF from fitting different Cox regression models in the different subgroups of ART‐experienced virologically suppressed PLWHIV.

**Table:** 

**Abstract P098 – Table 1**. Baseline demographic and clinical characteristics of the 1237 PLWHIV switching to BIC/FTC/TAF in the Icona cohort.		
	ART‐experienced VS (N = 1237)	
Italian, n (%)	1043	(84.3)
Ethnicity, Caucasian, n (%)	1102	(89.1)
Gender, female, n (%)	229	(18.5)
Year of BIC start, median (IQR)	2019	(2019 to 2020)
Year cART start, median (IQR)	2015	(2011 to 2017)
Age, years, median (IQR)	47	(39 to 55)
− Age, >50 years, n (%)	544	(43.98)
Mode of HIV transmission, n (%)		
Heterosexual	456	(36.86)
IVDU	100	(8.08)
MSM	622	(50.28)
Other/unknown	59	(4.77)
HCVAb positive status, n (%)	129	(10.43)
HBsAg positive status, n (%)	49	(3.96)
Previous AIDS, n (%)	203	(16.41)
CD4, cells/mmc, median (IQR)	702	(505 to 928)
CD4 <200 cells/mmc, n (%)	28	(2.26)
CD4 <350 cells/mmc, n (%)	140	(11.3)
CD4/CD8 ratio, median (IQR)	0.87	(0.59 to 1.22)
HIV‐RNA, copies/mL, median (IQR)	2	(1 to 24)
LDL cholesterol, mg/dL, median (IQR)	120	(100 to 145)
HDL cholesterol, mg/dL, median (IQR)	49	(41 to 58)
Triglycerides, mg/dL, median (IQR)	116	(83 to 168)
Serum glucose, mg/dL, median (IQR)	88	(81 to 96)
eGFR, CKD‐EPI, mL/min, median (IQR)	89.5	(77.2 to 101.8)
Weight, kg, median (IQR)	74	(66 to 82)
BMI, kg/m^2^, median (IQR)	24.2	(22.2 to 26.9)
Follow‐up on BIC, years, median (IQR)	1.37	(0.97 to 1.67)
Previous ART regimen		
INSTI‐based	1061	(85.77)
NNRTI‐based	95	(7.68)
PI‐based	62	(5.01)
Other	19	(1.54)

**Materials and methods**: Observational study including ART‐experienced VS PLWHIV from the Icona cohort who switched for the first time to BIC/FTC/TAF from April 2018 to December 2021. Primary objective: treatment failure (TF) i.e. virological failure (VF: two HIV‐RNA >200 copies/mL or one HIV‐RNA >1000 copies/mL) or treatment discontinuation (TD) for any reason. Secondary objectives: TD for any reason; TD for toxicity/intolerance; VF; variation of CD4, CD4/CD8 and weight. Standard survival analysis (Kaplan–Meier curves and log‐rank test) and crude and adjusted Cox regression to evaluate risk of TF for different groups: ≥50 years old, female and PLWHIV switching from NNRTI‐based regimen. Paired t‐test for mean changes at 12 (±3) months.

**Results**: One thousand, two hundred and thirty‐seven PLWHIV included (44.0% >50 years, 18.5% female, 5.7% switching from NNRTIs). Patients’ characteristics shown in Table 1. Over a median follow‐up of 1.4 years (IQR 1.0 to 1.7) 112 TF occurred (9.1%, 14 VF and 98 TD). The 1‐year probability of TF was 4.6% (95% CI 3.5 to 6.1), subgroups probabilities in Figure 1A. In the adjusted Cox regression models, age ≥50 years and NNRTI class in the previous regimen were not with a higher risk of TF, while female had a 2‐fold higher risk of TF (Figure 1B). After excluding six TD for pregnancy as events, TF risk in female was attenuated to aHR 1.63 (95% CI 0.93 to 2.90). One hundred TD (8.1%): 41 simplifications (3.3%), 30 toxicity/intolerance (2.4%), three failures (0.2%), three patient's decision (0.2%) and 23 (1.9%) other reasons (including the six pregnancies). The 1‐year probabilities of TD for any reason, toxicity/intolerance and VF are in Figure 1A. Mean CD4 increase at 1 year was +36 cells/mmc (95% CI 22 to 51, p < 0.001), mean CD4/CD8 ratio change was +0.06% (0.04 to 0.08, p < 0.001). Weight change at 1 year among 232 PLWHIV was +1.9kg (1.1 to 2.7, p < 0.001) with greater increase in PLWHIV with previous NNRTIs +2.8 kg (0.15 to 5.5, p < 0.001).

**Conclusions**: BIC/FTC/TAF as switch strategy demonstrated high effectiveness (4.6% TF and 0.7% VF at 1 year). Higher risk for female seems mainly related to pregnancy discontinuation.

#### P099 Detection of 12‐month immunological changes in patients randomised to switch either to BIC/TAF/FTC or DTG/3TC (DEBATE study)

A Cossarizza^1^, M Mattioli^1^, A Paolini^1^, A Neroni^1^, S De Biasi^1^, D Lo Tartaro^1^, R Borella^1^, L Fidanza^1^, L Gibellini^1^, B Beghetto^2^, E Roncaglia^2^, G Nardini^2^, M Menozzi^2^, G Cuomo^2^, M Di Gaetano^2^, G Orlando^2^, V Borghi^2^, C Mussini ^2^

^1^Pathology, University of Modena and Reggio Emilia, Modena, Italy; ^2^Infectious Diseases, University of Modena and Reggio Emilia, Modena, Italy

**Background**: Dolutegravir/lamivudine (DTG/3TC) is recommended either as initial or switch regimen by international guidelines; however, few data exist on inflammation after switching to this regimen. The aim of the present study was to evaluate, in a randomised longitudinal study, the immunological impact of switching to DTG/3TC or to bictegravir/emtricitabine/tenofovir alafenamide (BIC/TAF/FTC).

**Methods**: Open‐label, prospective, randomised trial enrolling 66 patients on a triple‐drug regimen and with a stable (>12 months) undetectable HIV RNA. Blood was obtained from patients treated with BFTAF (group A, n = 33) or DTG/3TC (n = 33) longitudinally studied at time 0, after 6 (T6) and after 12 months (T12). By polychromatic flow cytometry, we characterised peripheral blood T cells, B lymphocytes and monocytes. Statistical analysis was performed by paired or unpaired Student's t test.

**Results**: At T12 absolute number of T and B lymphocytes were similar in both groups. However, differences were present in CD4+: the DTG/3TC group showed a more marked increase with time in transitional memory lymphocytes (T0 vs T6: p = 0.0022; T0 vs T12: p < 0.0001), terminally differentiated T cells (T0 vs T12: p = 0.0007), exhausted cells (T0 vs T12: p = 0.0004) and T stem cell memory (T0 vs T6: p = 0.0014; T0 vs T12: p = 0.0019). Activated CD4+ T cells were more represented in BFTAF group (T0 vs T12: p = 0.0367). Activated CD8+ T cells expressing HLA‐DR and CD38 increased similarly in both groups, while those with markers of exhaustion were more represented in DTG/3TC group (T0 vs T6: p = 0.0029; T0 vs T12: p = 0.0426; p = 0.0260 between groups at T12). No significant changes were seen among B cell populations. Total monocyte number and percentage did not change with time in both groups, but classical monocytes (CD14++CD16‐) increased in BFTAF group (T0 vs T6: p = 0.009; p = 0.0028 between groups at T6), while nonclassical monocytes (CD14+CD16++) increased with time in DTG/3TC group (T0 vs T6: p = 0.0002; T0 vs T12: p = 0.0195).

**Conclusion**: In this randomised study, switch to DTG/3TC was associated after 12 months with an increase both in CD4+ and CD8+ T lymphocytes with markers related to terminal differentiation  and exhaustion, and in nonclassical monocytes, a population of cells that has been recently associated with endothelial dysfunction.

#### P100 Durability of first‐line antiretroviral treatment in Russia: retrospective study

N Sizova^1^, Y Plotnikova^2^, T Shimonova^3^, O Chernova^4^, E Ivanova^5^, A Kruglova^6^, V Achikyan ^6^

^1^Administration, Saint Petersburg Center for Treatment and Prophylaxis of AIDS and Infectious Diseases, Saint Petersburg, Russian Federation; ^2^Administration, Irkutsk Regional Center for Treatment and Prophylaxis of AIDS and Infectious Diseases, Irkutsk, Russian Federation; ^3^Centre of Clinical Studies, Moscow Infectious Diseases Clinical Hospital #2, Moscow, Russian Federation; ^4^Administration, Samara Regional Clinical Center for Treatment and Prophylaxis of AIDS and Infectious Diseases, Samara, Russian Federation; ^5^Therapy Department, Perm Regional Center for Treatment and Prophylaxis of AIDS and Infectious Diseases, Perm, Russian Federation; ^6^Medical Affairs, MSD Pharmaceuticals LLC, Moscow, Russian Federation

**Background**: Adherence and antiretroviral treatment (ART) durability are important because of life‐long nature of the disease. Data on treatment durability in people living with HIV (PLWHIV) initiating ART in Russia is limited. Understanding durability of ART is critical for improving HIV management.

**Materials and methods**: A multicentre retrospective study was conducted including data from review of medical records of PLWHIV who signed informed consent form. PLWHIV with no experience of therapy at time of initiation of ART with non‐nucleoside reverse transcriptase inhibitors (NNRTIs) plus two nucleoside reverse transcriptase inhibitors (NRTIs) or ritonavir boosted protease inhibitor (PI) plus two NRTIs were followed up for at least 96 weeks according to daily clinical practice conducted by their physicians. Data were retrospectively collected at time points of baseline (pre‐treatment), 48±8 and 96±8 weeks after start of ART. ART durability was assessed as percentage of PLWHIV who remained on initial ART without change (switch or withdrawal) and time on therapy measured as the cumulative time in weeks that users were on their initial ART regimen without change. Association between ART durability and baseline characteristics (age, gender, marital, employment status, alcohol and/or substance abuse, duration of HIV diagnosis at baseline, route of infection, HIV stage, viral load, CD4 cells count at baseline, comorbidities, concomitant medications) was assessed. Descriptive statistics and logistic regression analysis were used.

**Figure:**
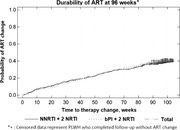
**Abstract P100 – Figure 1**. Durability of ART at 96 weeks*.

**Results**: Five hundred and thirty‐six PLWHIV were included in the analysis. Most PLWHIV were men (59.5%), less than 40 years old (63.1%), employed (66.2%), without alcohol and/or substance abuse (84.1%), infected via heterosexual contact (56.0%), with HIV stage IV (87.3%). The data on ART durability are presented in Table 1 and Figure 1. No baseline characteristics were associated with ART change prior to 48 weeks. Only age ≥40 years was statistically associated with ART change prior to 96 weeks. (OR 1.391, 95% CI 1.005 to 1.925).

**Table:** 

**Abstract P100 – Table 1**. Durability of ART and time on therapy without ART change.			
Variables	Subjects on NNRTI plus 2 NRTIs	Subjects on bPI plus 2 NRTIs	All participants
Patients at baseline, n (%)	387 (72.2)	149 (27.8)	536 (100.0)
Durability of ART at 48±8 weeks, % (95% CI for proportion, %)	75.9 (71.3 to 80.1)	76.4 (68.7 to 82.9)	76.0 (72.2 to 79.6)
Time on therapy without ART change at 48±8 weeks of follow‐up, weeks, mean (SD)	46.0 (15.0)	47.2 (14.7)	46.8 (15.1)
Durability of ART at 96±8 weeks, % (95% CI for proportion, %)	61.0 (55.9 to 65.9)	58.4 (50.0 to 66.4)	60.3 (56.0 to 64.4)
Time on therapy without ART change at 96±8 weeks of follow‐up, weeks, mean (SD)	78.9 (34.2)	75.7 (31.2)	78.8 (33.8)

**Conclusions**: In real clinical practice in Russia, durability of first‐line ART was 76.0% at week 48 and 60.3% at week 96 with mean time on ART 46.8 weeks and 78.8 weeks after therapy initiation, correspondingly. Age ≥40 years was associated with ART change at 96 weeks of follow‐up.

#### P101 Efficacy and safety of dolutegravir and doravirine dual therapy in the context of antiretroviral therapy switch: 48 weeks analysis

M Trizzino, C Gioè, A Medaglia, P Di Carlo, M Alfonzo, L Pipitò, G Valenti, A Cascio

Infectious and Tropical Disease Department, University of Palermo, Palermo, Italy

Dual therapy in HIV represents an attractive opportunity for HIV‐infected people in virological suppression. To date, there are few clinical data to support a dual regimen with dolutegravir and doravirine [1]. The aim of our study is to investigate whether a dual therapy regimen containing dolutegravir and doravirine is effective and safe.

**Figure:**
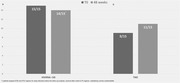
**Abstract P101 – Figure 1**. Summary of study result. (a) Number of patients with HIV‐RNA <50 copies/mL. (b) Number of patients with HIV‐RNA target not detected.

**Table:** 

**Abstract P101 – Table 1**. Data analysis at 48 weeks.			
	Before switch	After 48 weeks	p‐value
CD4+, cell/mm^3^, mean (SD)	692 (284)	689 (232)	0.930
CD4/CD8 ratio, mean (SD)	1.13 (0.52)	1.23 (0.55)	0.230
Neutrophil‐to‐lymphocyte ratio NLR, mean (SD)	1.929 (0.829)	1.857 (0.770)	0.671
Total cholesterol, mean (SD)	197.2 (37.1)	188.9 (33.3)	0.365
HDL cholesterol, mean (SD)	55.1 (24)	55.0 (17.7)	0.977
T/HDL cholesterol ratio, mean (SD)	3.9 (1.13)	3.6 (0.8)	0.152
Fasting glucose, mean (SD)	82.4 (23.6)	95 (14.2)	0.161
Triglycerides, mean (SD)	132.8 (68.9)	112.5 (44.9)	0.140
Creatinine, mean (SD)	0.9 (0.2)	0.9 (0.2)	0.582
eGFR, mean (SD)	90.9 (21.5)	87.9 (17.2)	0.419

A prospective observational study lasting 24 months (February 2021 to February 2023) is conducted at the Infectious Diseases Unit of University of Palermo to evaluate the efficacy, safety and tolerability of a two‐drugs antiretroviral scheme, containing dolutegravir and doravirine, in a cohort of patients with HIV infection and already on effective antiretroviral therapy. The primary endpoint of the study is maintenance of viral suppression at 48 weeks (±6 weeks) with HIV‐1 RNA <50 copies/mL. Secondary endpoints are: 1) percentage of participants without virological failure; 2) strategy success rate, defined as the percentage of participants without virological failure or without interruption of study treatment; 3) genotypic resistance profile in case of virological failure; 4) changes in CD4 cell counts, CD4/CD8 ratio, neutrophil‐to‐lymphocyte ratio (NLR) as a marker of systemic inflammation; 5) eGFR, metabolic profile; 6) clinical and laboratory adverse events. Fifteen patients were included in this analysis, 10 (66.7%) males and 5 (33.3%) women. Mean age was 50.4 years (SD 12.2), with a mean length of HIV disease of 13.2 years (SD 7.94), mean nadir CD4 was 379/mmc (SD 201). Most frequent comorbidities were hypertension (40%), dyslipidaemia (46.7%), osteoporosis and CKD (13.3%), NCS disorders, metabolic syndrome, diabetes and HCV coinfection (6.7%). Switch to DOR and DTG were mainly due to high CV and metabolic risk (9/15, 60%). Antiretrovirals that patients were switched off were mostly integrase inhibitors (7/15, 46.7%), followed by boosted protease inhibitors (8/15, 53.3%). All 15 patients meets baseline criteria for HIV‐RNA viraemia <50 copies, 9/15 (60%) had complete suppression (TND). After 48 weeks, we observed a maintainance in viral suppression among all those who were undetectable at the baseline, 11/15 obtained complete suppression (TND). One patients stopped DOR and DTG regimen for sleep disorders before the follow‐up analysis, resolved after switch to PI regimen, maintaining viraemia undetectability. No significant changes in CD4, CD4/CD8 ratio, metabolic profile, renal function and NLR ratio were observed from the baseline. Combination strategies with a good tolerability profile, (Figure 1) (Table 1) a high genetic barrier and also the ability to be administered once‐daily should be preferred in a setting such as the current SARS‐CoV‐2 pandemic. The combination of doravirine together with dolutegravir, a drug characterised by an excellent profile of potency, safety and long‐term tolerability, appears to be a potential strategy in the setting of patients who require a regimen which maintains high virological efficacy but reduces long‐term toxicity and pharmacological potentials.

**Reference**

1. Mazzitelli M, Sasset L, Leoni D, Putaggio C, Cattelan AM. Real life use of dolutegravir doravirine dual regimen in experienced elderly PLWH with multiple comorbidities and on polypharmacy: a retrospective analysis. Medicine (Baltimore). 2021;100:e28488.

#### P102 One year of follow‐up after switching to doravirine in treatment‐experienced people living with HIV: a real‐world cohort study

V Lanting ^1^, P Oosterhof^2^, D Ait Moha^1^, R van Heerde^1^, M Kleene^1^, J Stalenhoef^1^, M de Regt^1^, S Vrouenraets^1^, G van den Berk^1^, K Brinkman^1^

^1^Internal Medicine, Onze Lieve Vrouwe Gasthuis Hospital, Amsterdam, Netherlands; ^2^Clinical Pharmacy, Onze Lieve Vrouwe Gasthuis Hospital, Amsterdam, Netherlands

**Background**: Doravirine is a non‐nucleoside reverse transcriptase inhibitor with demonstrated effect as third agent in treatment‐naive and ‐experienced people living with HIV (PLWHIV). Real‐world data studies are still scarcely available.

**Aim**: To evaluate efficacy and tolerability of doravirine‐based regimens 1 year after cART‐switch in treatment‐experienced and virologically suppressed PLWHIV.

**Methods**: A retrospective analysis was done of all treatment‐experienced, virologically suppressed PLWHIV in a Dutch HIV treatment centre (Onze Lieve Vrouwe Gasthuis (OLVG) Amsterdam) for whom doravirine was prescribed between September 2019 and June 2021 with a follow‐up of 1 year. In the OLVG care path, reasons for switching cART are documented prospectively. All PLWHIV who stopped doravirine within 1 year were identified and the reasons to stop were collected. Medication prices in the Netherlands were used for estimation of medication costs. Descriptive statistics using IBM SPSS were performed.

**Table:** 

**Abstract P102 – Table 1**. Percentage of patients that stopped therapy after one year and the reason to stop as percentage of the total 687 patients.		
Patients with one‐year of follow‐up	n=687	Percentage
**Continued**	**593**	**86.3%**
**Stopped**	**94**	**13.7%**
*Reason to stop*		
**Virologic failure**	**4**	**0.6**
Resistance ‐ V106A	1	0.1%
Incompliance	1	0.1%
Unknown	2	0.3%
**Medical reason**	**11**	**1.6%**
**Patient‐reported adverse event^a^**	**70**	**10.2%**
Insomnia	17	2.5%
Psychological symptoms	17	2.5%
Gastro‐intestinal	20	2.9%
Musculoskeletal	11	1.6%
Possible allergic skin reactions	8	1.2%
Other skin reactions	6	0.9%
**Other**	**8**	**1.2%**

^a^First row demonstrates the total number of patients followed by the categories of adverse events. Some patients had more than one adverse event as reason to stop.

**Results**: A total of 687 PLWHIV (92% men) were included: 97.7% switched to doravirine/tenofovir/lamivudine (DOR/TDF/3TC) and 2.3% to other doravirine‐based regimens. After 1 year 593/687 (86.6%) PLWHIV continued therapy. In the group of 94/687 (13.7%) PLWHIV that stopped therapy, the main reason was patient‐reported adverse events in 70/687 (10.2%); most frequent reasons were insomnia or psychological (4.9%) and gastrointestinal (2.9%) (see Table 1). Medical reasons to stop were 'increased alanine transaminase (ALT) levels' in 6/687 (0.9%), 'elevated creatinine levels' in 3/687 (0.4%), 'as precaution' after diagnosis of osteoporosis in 2/687 (0.3%) and 'virological failure' in 4/687 cases (0.6%). One person demonstrated a V106A resistance‐mutation to doravirine at failure, which in retrospect was already present in the medical history. No resistance could be demonstrated in the other three PLWHIV. Finally, 9/687 (1.3%) patients had another reason to stop. Within the PLWHIV that continued treatment, medication costs decreased from €4 553 716 to €3 470 974 (23.8% reduction) during 1 year of follow‐up. Body mass index (BMI) was available in 58/593 of PLWHIV at switch and after 1 year. No significant change was observed (BMI 26.05 vs 25.84, p = 0.34).

**Conclusion**: The large majority of patients successfully continued a doravirine‐based regimen after 1 year of follow‐up, demonstrating its effective, well‐tolerated and cost‐saving role in maintenance therapy.

#### P103 ART regimen persistence among treatment‐experienced patients with HIV switching to a MTR or STR since 2018

B Chastek^1^, A Anderson^1^, N Webb^1^, M Rock^2^, J Gruber^3^, S Majethia^4^, W Zachry ^4^, J Cohen^5^, A Colson^6^

^1^Health Economics and Outcomes Research, Optum, Eden Prairie, MN, USA; ^2^Global Value and Access, Gilead Sciences, Foster City, CA, USA; ^3^Medical Affairs Research, Gilead Sciences, Foster City, CA, USA; ^4^Patient Access and Quality, Gilead Sciences, Foster City, CA, USA; ^5^Joshua P. Cohen Healthcare Analytics, LLC, Boston, MA, USA; ^6^Access Health, Cambridge, MA, USA

**Background**: Persistence on antiretroviral therapy (ART) has been linked to better virologic and patient‐reported outcomes. This study describes regimen persistence for treatment‐experienced people living with HIV (PLWHIV) after switching ART to multiple (MTR) and single (STR) tablet regimens.

**Figure:**
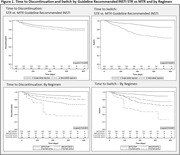
**Abstract P103 – Figure 1**. Time to discontinuation and switch by guideline recommended INSTI STR vs MTR and by regimen.

**Methods**: A retrospective study was performed using claims data from Optum Research Database (01/01/2010 to 01/03/2020). Lines of therapy (LOTs) were included for treatment‐experienced adults switching to Department of Health and Human Services (DHHS) guideline recommended triple drug regimens between 01/01/2018 and 12/31/2019. LOT duration and reason for LOT cessation (ART discontinuation [a gap in all ART therapy ≥60 days], ART switch, or end of available data) were compared for the following LOT types: 1) STR versus MTR; 2) INSTI‐based triple MTR versus STR; 3) specific INSTI‐based triple drug regimens. Descriptive analyses, including Kaplan‐Meier analysis, were conducted following inverse probability treatment weighting to control for differences in baseline characteristics. Hazard ratios (HRs) from Cox proportional hazards models were examined following weighting to control for residual differences in the regimen‐specific comparisons.

**Results**: A total of 7456 eligible treatment‐experienced PLWHIV were identified: STR (N = 6505) versus MTR (N = 951). Among those switching to INSTI triple therapy 3625 started a STR and 626 started a MTR. Regimens included: B/FTC/TAF (N = 2727; 64.2%), ABC/3TC/DTG (N = 898; 21.1%), FTC/TAF+DTG (N = 539; 12.7%), and FTC/TDF+DTG (N = 87; 2.1%). Discontinuation was roughly comparable between STR and MTR (15% vs 16%, p = 0.245) and switching was less common among STR than MTR (12% vs 31%, p < 0.01). For those treated with INSTI regimens, discontinuation (11% vs 15%, p < 0.01) and switching (7% vs 25%, p < 0.01) were less common for STR versus MTR. Compared to B/FTC/TAF, the rate of discontinuation was greater for ABC/3TC/DTG (HR 2.755, p < 0.01), FTC/TDF+DTG (HR 5.869, p < 0.01), and FTC/TAF+DTG (HR 1.944, p < 0.01). Similarly, compared to B/FTC/TAF, the rate of switching was greater for ABC/3TC/DTG (HR 3.340, p < 0.01), FTC/TDF+DTG (HR 13.080, p < 0.01), and FTC/TAF+DTG (HR 4.483, p <0.01) (Figure 1).

**Conclusion**: Patients starting B/FTC/TAF were less likely to switch or discontinue ART compared to other INSTI regimens. Compared to MTR, PLWHIV treated with STR were less likely to discontinue or switch off their regimen.

#### P104 Real‐life experience with bictegravir/emtricitabine/tenofovir alafenamide (B/F/TAF) in a single centre in Portugal

A Gorgulho ^1^, I Vaz‐Pinto^2^, C Santos^2^, M Guimaraes^2^, V Castro^2^

^1^Internal Medicine, Hospital de Cascais, Alcabideche, Portugal; ^2^HIV Unit, Hospital de Cascais, Alcabideche, Portugal

**Background**: The use of B/F/TAF is based on robust results from registrational clinical trials. Available data from its use in routine clinical practice seems to support these results. We report 6‐ and 12‐month analysis of the experience with B/F/TAF in a single centre in Portugal.

**Materials and methods**: This observational, retrospective, single centre analysis included all treatment‐naïve (TN) and treatment‐experienced (TE) people living with HIV (PLWHIV) who started B/F/TAF from 03/2020 until 06/2021. We describe baseline demographic and HIV‐related characteristics and evaluate effectiveness, tolerability, safety, and specific laboratory parameters in all patients who achieved 6 and 12 months of follow‐up.

**Results**: One hundred and seventy‐two PLWHIV (28 TN [16%] and 144 TE [84%]) were included in this analysis. Median (IQR) follow‐up was 19 (14.3 to 20.75) and 18 (13 to 21) months for TN and TE, respectively. Sixty‐one percent were male, 80.2% Caucasian, mean age 49 (20 to 83); 25% of TN and 48.6 of TE were older than 50. Median (IQR) TCD4+ count at baseline was 397 (196 to 561) cells/μL (25% <200) for TN and 583 (418 to 784) cells/μL (3.5% <200) for TE participants; baseline VL was 184 303 (138 to 2 547 424) copies/mL (28.6% >100 000 copies/mL) in TN. Of TE participants 43.9%/24.3%/31.8% switched from INSTI/NNRTI/PI‐based regimens and the main reason for switch was simplification (65.5%). At least two or more comorbidities were present in 46.4% of TN and 60.1% of TE. HCV coinfection was present in 34.3% of TE but only in 14.3% of TN participants. By on treatment (OT) and intent‐to‐treat (ITT) analysis, HIV RNA <50 copies/mL was 95.8%/92% at M6 and 100%/83.3% at M12 for TN and 99.2%/90.6% at M6 and 95.6%/82.2% at M12 for TE participants. Persistence in treatment at M6 and M12 was 89.3%/85.7% in TN and 95.8%/93.8% in TE, with nine discontinuations at M6 and four at M12. Median CD4 cell count increased from 397 to 511 and to 588/μL in TN and from 583 to 585 to 595/μL in TE PLWHIV at M6 and M12.

**Conclusions**: The use of B/F/TAF in this real‐world cohort supports the results from clinical trials, showing high rates of virological suppression and persistence at 6 and 12 months of follow‐up.

#### P105 Effectiveness and tolerability of the bictegravir/emtricitabine/tenofovir alafenamide regimen in a cohort of HIV‐1 infected treatment experienced adult patients: an observational retrospective single‐centre study

M Ceccarelli ^1^, M Ricciardetto^1^, B Bellocchi^2^, L Todaro^2^, V Boscia^3^, A Campanella^2^, G Nunnari^2^, B Cacopardo^1^, B Celesia^3^

^1^Unit of Infectious Diseases, Department of Clinical and Experimental Medicine, University of Catania, Catania, Italy; ^2^Unit of Infectious Diseases, Department of Clinical and Experimental Medicine, University of Messina, Messina, Italy; ^3^Unit of Infectious Diseases, Azienda di Rilevanza Nazionale e di Alta Specializzazione Garibaldi, Nesima Hospital, Catania, Italy

**Background**: Integrase strand transfer inhibitors (INSTIs) are effective and well tolerated. Bictegravir is also associated with low levels of cART resistance and decreased markers of inflammation. We aimed to evaluate the effects of switching to bictegravir/emtricitabine/tenofovir alafenamide from different regimens, especially comparing boosted and unboosted regimens.

**Materials and methods**: We collected data about lipid metabolism, HIV infection, HIV‐RNA plasma viral load, weight, creatinine at four time‐points (baseline, 6, 12 and 18 months after switch) of experienced people living with HIV (EPLWH) switching from 1 July 2019, to a fixed‐dose single‐tablet regimen of bictegravir/emtricitabine/tenofovir alafenamide. Categorical variables are expressed as count (percentages), while continuous variables as mean ± SD when normally distributed or median (IQR) when non‐normally distributed.

**Figure:**
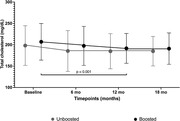
**Abstract P105 – Figure 1**. Total cholesterol trend in PLWH switched to BIC/FTC/TAF from boosted vs unboosted regimens.

**Results**: Ninety EPLWH, 77.8% male, median age 45.8 years (IQR 37.6 to 56.2), switching from 15 different regimens, were included. Seventy‐two (80.0%) came from an INSTI‐based regimen, 61.1% from elvitegravir/cobicistat/emtricitabine/tenofovir alafenamide, 10 (11.1%) from a protease inhibitor‐based regimen, and eight (8.9%) from a non‐nucleoside reverse‐transcriptase inhibitor‐based regimen. Globally 66 (73.3%) came from a boosted regimen. At baseline no statistically significant differences were found between boosted and unboosted regimens regarding having an undetectable viraemia (p = 0.794); those coming from a boosted regimen showed higher counts of CD8+ T‐cells (912±454) than unboosted ones (660±267) (p = 0.012). Moreover, those coming from a boosted regimen showed lower levels of HDL (p = 0.024). During follow‐up total cholesterol significantly decrease at 12 months (p < 0.001) in patients coming from a boosted regimen (Figure 1) while CD4+/CD8+ ratio was higher in patients coming from an unboosted treatment, although the values are not significantly different. Although the starting values are not significantly different at baseline (p = 0.402), total cholesterol in patients coming from a boosted regimen significantly decrease at 12 months (p < 0.001) compared with baseline.

**Conclusion**: Our data show that switching from a boosted regimen to bictegravir/emtricitabine/tenofovir alafenamide is safe and advisable, especially for the effects on total cholesterol. Moreover, our data seem to support the idea that boosted regimens negatively influence the status of the immune system, not being able to completely tame the inflammatory stimulus caused by the HIV infection.

#### P106 Effectiveness and safety of doravinine from a real‐world HIV cohort: DORWINS study

N Espinosa Aguilera ^1^, M Mejías Trueba^2^, A Gutiérrez Valencia^1^, S Llaves Flores^1^, M Herrero Romero^1^, C Sotomayor de la Piedra^1^, C Roca‐Oporto^1^, M Rodriguez Hernandez^1^, L Lopez Cortes^1^

^1^Unidad Clinica Enfermedades Infecciosas y Microbiologia Clínica, Hospital Universitario Virgen del Rocio, Sevilla, Spain; ^2^Unidad Clinica Farmacia Hospitalaria, Hospital Universitario Virgen del Rocio, Sevilla, Spain

**Background**: Doravirine is a new non‐nucleoside reverse transcriptase inhibitor (NNRTI) which has demonstrated long‐term safety and efficacy as an antiretroviral (ART) switch strategy in clinical trials, with a higher genetic barrier and fewer drug interactions than alternatives from the same family. We evaluate the effectiveness, safety, and tolerability of doravirine at week 48 in a real‐world HIV cohort.

**Materials and methods**: Observational and ambispective single‐centre cohort study that included HIV‐infected patients who switched to a doravirine‐based regimen from October 2020 to March 2022. Effectiveness was defined as the proportion of patients with a viral load ≤50 and ≤200 copies/mL at week 48. Routinely clinical and laboratory data were collected every 1 to 6 months based on clinical criteria.

**Results**: One hundred and eighty‐nine patients (84.7% males), with a median age of 51.8 (IQR 43.5 to 57.5) years were included. At baseline, the median of CD4+ T cell count was 684 (IQR 527 to 883) and 171 (90.5%) patients had a viral load (VL) of ≤50 copies/mL. The median duration of HIV infection was 14 (IQR 8 to 27) years and patients had received a median of 5 previous ART regimens (IQR 4 to 9). Resistance mutations were registered in 85 patients: 18 (9.5%) 184V/I; six (3.2%) 138K/A/G; 16 (8.5%) 103N/R/Q; and three (1.6%) 190A. The most prescribed combination was DOR+3TC+ABV, n = 110 (58.2%) followed by DOR+FTC+TDF, n = 37 (19.6%) and DOR+DTG, n = 25 (13.2%). The median follow‐up was 13.4 (IQR 6.63 to 17.36) months. Doravirine was discontinued in 20 patients: virological failure, n = 1 (0.5%); adverse events (AEs), n = 10 (5.2%); patient decision, n = 4 (2.1%); physician decision, n = 4 (2.1%); withdrawal, n = 1 (0.5%). All AEs were mild and self‐limited. The effectiveness in an intention‐to‐treat analysis at week 48 was 89.9%. In the analysis per protocol, the proportion of patients with viral load ≤50 and ≤200 copies/mL at week 48 was 95.1% and 98.8%, respectively. No immunological parameters, weight, lipid, liver, and renal profile changes were observed.

**Conclusions**: In our experience, doravirine is an effective and safe switch strategy in a real‐world setting even in treatment‐experienced HIV patients.

#### P107 Efficacy and safety of switching to co‐formulated DOR/TDF/3TC in virologically suppressed HIV‐1‐infected patients: early experience from a single Italian centre

F Lagi^1^, G Formica ^2^, F Ducci^2^, S Tekle Kiros^2^, M Pozzi^1^, F Bartalesi^1^, P Corsi^1^, C Malcontenti^1^, G Sterrantino^2^, A Bartoloni^2^

^1^Infectious and Tropical Diseases Unit, Careggi University Hospital, Florence, Italy; ^2^Department of Experimental and Clinical Medicine, University of Florence, Florence, Italy

**Background**: Doravirine (DOR) belongs to the class of non‐nucleoside reverse transcriptase inhibitors (NNRTI) and is also available co‐formulated with tenofovir disoproxil (TDF) and lamivudine (3TC). Real‐life data on DOR is lacking. The study aims to evaluate the efficacy and safety of switching to DOR/TDF/3TC in a cohort of HIV‐living persons treated in a single centre in Florence, Italy.

**Materials and methods**: This is a retrospective‐monocentric cohort study. From 01/01/2020 to 31/12/2021, we included all HIV‐1 positive, ART‐experienced persons switching to DOR/TDF/3TC with HIV‐RNA <50 copies/mL. Study entry was the date of drug initiation; exit was the date of discontinuation, virological failure (VF), or the end of follow‐up (FU) (30/05/2022).

**Table:** 

**Abstract P107 – Table 1**. Clinical/demographic characteristics and outcome of HIV‐1 infected patients, ART experienced with HIV‐RNA level <50 copies/mL switching to DOR/TDF/3TC at the Infectious and Tropical Diseases Unit in a tertiary teaching hospital in Florence, Italy.		
Italians, n (%)	35	66.0
Gender, n (%)		
Female	4	7.5
Male	43	81.3
Transgender	6	11.3
Age at entry, median [IQR]	52	44 to 58
Risk of HIV transmission, n (%)		
Heterosexual	15	28.3
MSM	31	58.5
IVDU	3	5.7
Other/not known	4	7.5
Cardiovascular disease	9	16.9
Diabetes	4	7.5
Dyslipidaemia	13	24.5
Mental disorders	5	9.4
No comorbidity	7	13.2
Years of undetectable viraemia, median [IQR]	6	[2 to 9]
AIDS diagnosis, n (%)	15	28.3
Previous virological failure	3	5.7
HBsAg positivity, n (%)	3	5.7
HIV‐RNA zenith, log 10 copies/mL, median [IQR]	5.0	4.6 to 5.4
Nadir CD4 (cells/mL), median [IQR]	350	131 to 477
Years of HIV, median [IQR]	10	4 to 18
Years of antiretroviral therapy, median [IQR]	9	4 to 16
CD4+ T cells at baseline/μL, median [IQR]	697	525 to 923
Type of pre‐switch regimen		
NNRTI	30	56.6
PI	5	9.4
INSTI	14	26.4
Other	4	7.5
Number of previous ART regimens, median [IQR]	3	2 to 5
Type of pre‐switch backbone		
3TC/ABC	3	5.6
FTC/TAF	34	64.1
FTC/TDF	12	22.6
3TC	3	5.7
No backbone	1	1.9
Major drug resistance on historical genotype^a^		
Low‐level resistance TDF	1 out of 31	3.23
Low‐level/potential low‐level resistance DOR	4 out of 31	12.9
High‐level resistance 3TC	3 out of 31	9.7
Discontinuation for all causes	7	13.2
Virological failure	1	
Gastrointestinal toxicity	3	
Itchy	2	
Rash	1	

^a^Historical genotype available in 31 out of 53 patients.

**Results**: We included 53 patients (median FU: 1.3 years). The majority were male (n = 43; 81.3%) and Italian‐born (n = 35; 66%). Baseline characteristics are  summarised in the Table (Table 1). Most had at least one comorbidity (n = 46; 86%): dyslipidaemia (24.5%), cardiovascular diseases (16.9%) and mental disorders (9.4%). Pre‐switch regimen was mainly NNRTI‐based (n = 30; 56.6%) and INSTI‐based regimen (n = 14; 26.4%). Historical genotype was available in 31 out of 53 patients. According to Stanford algorithm, low‐level TDF resistance was reported in one case, potential low‐level/low‐level DOR resistance in four cases, and high‐level resistance 3TC in three cases. Overall, we observed seven discontinuations: six due to grade 1 to 2 adverse events (mainly gastrointestinal three out of seven) and one due to virological failure (Table 1). No baseline or acquired resistance was identified in the VF patients. The 48‐week probability of remaining free of discontinuation was 88.6% (95% CI 74.6 to 95.1). The discontinuation rate was 11.1 x 100 py [5.3 to 23.3]. A lower, although not significant, discontinuation rate for adverse events was observed in people who came from an NNRTI‐based regimen compared to an INSTI‐based regimen (RR 0.25 [95% CI 0.04 to 1.62]; p = 0.1473) (Figure 1). No difference in total CD4 count, cholesterol, or triglycerides was observed at the end of FU.

**Figure:**
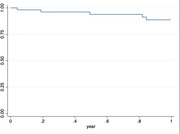
**Abstract P107 – Figure 1**. One‐year probability of remaining free from treatment discontinuation for all causes in HIV‐1 infected patients, ART experienced with HIV‐RNA level <50 copies/mL switching to DOR/TDF/FTC at the Infectious and Tropical Diseases Unit in a tertiary teaching hospital in Florence, Italy.

**Conclusions**: The switch to DOR/TDF/3TC maintained virological suppression with a low risk of VF. The discontinuation rate for adverse events seemed lower when the previous regimen was NNRTI‐based. Larger cohort studies are needed to confirm these findings.

#### P108 Doravirine used as a fixed combination doravirine/lamivudine/tenofovir disoproxil, among persons living with HIV at switch and start: real‐life data from the Croatian HIV cohort

S Zekan, J Begovac

Department of Infectious Diseases, School of Medicine University of Zagreb, Zagreb, Croatia

**Background**: Delstrigo (doravirine/lamivudine/tenofovir disoproxil) is a recommended first‐line antiretroviral (ART) regimen in the EACS guidelines [1]. It became available in Croatia in August 2020. This retrospective cohort study evaluated persons living with HIV (PLWHIV), ART naïve or experienced, who initiated or switched to Delstrigo, in Croatia.

**Table:** 

**Abstract P108 – Table 1**. Antiretroviral combinations used before a switch to Delstrigo.		
Switch from ART	Number of PLWHIV	%
ABC/3TC+EFV	14	23.7
TDF/FTC+NVP	11	18.6
ABC/3TC+NVP	7	11.9
TDF/FTC+RAL	6	10.1
TDF/FTC+LOP/r	5	8.5
ABC/3TC+LOP/r	2	3.4
ABC/3TC+DRV/c	2	3.4
TAF/FTC/BIC	2	3.4
ABC/3TC+DTG	2	3.4
TDF/FTC+DTG	2	3.4
TDF/FTC/EFV	2	3.4
TDF/FTC/EFV	1	1.7
DRV/r	1	1.7
TDF/FTC+DRV/r	1	1.7
TDF/FTC+DRV/c	1	1.7
Total	59	100.0

3TC, lamivudine; ABC, abacavir; BIC, bictegravir; DRV/c, darunavir with cobicistat; DRV/r, darunavir with ritonavir; DTG, dolutegravir; EFV, efavirenz; FTC, emtricitabine; LOP/r, lopinavir with ritonavir; NVP, nevirapine; RAL, raltegravir; TAF, tenofovir alafenamide; TDF, tenofovir disoproxil fumarate.

**Materials and methods**: We collected information from the electronic database of the Croatian HIV Reference Center and identified 90 PLWHIV who started Delstrigo from August 2020 until December 2021. Excluded from the analysis were three PLWHIV who started Delstrigo as a part of non‐standard regimens and three PLWHIV who started Delstrigo after a discontinuation a of previous ART regimen for >1 month. We analysed 84 PLWHIV, 59 who switched to Delstrigo, and 25 were ART naïve. The follow‐up was minimum 5 months. The data are presented by frequencies and percentages or median with first and third quartiles (IQR).

**Results**: All PLWHIV were males, 77 (91.7%) were MSM. The median age of those who switched to Delstrigo was 45.0 (IQR 38.7 to 52.5) years; 10 (16.9%) had previously clinical AIDS; median time from HIV diagnosis was 9.1 (IQR 5.2 to 14.2) years; median time on ART was 8.6 (IQR 5.1 to 12.5) years. PLWHIV switched from various ART regimens (Table 1). Before switch 57 PLWHIV had an undetectable viral load and two had low‐level viraemia. During follow‐up only one PLWHIV had low‐level viraemia (<200 copies/mL). Serum lipid parameters declined: total median cholesterol (5.8 to 4.7 mmol/L), low‐density lipoprotein (LDL) cholesterol (3.7 to 3.1 mmol/L), and triglycerides (1.9 to 1.2 mmol/L). The median age of 25 naïve PLWHIV was 36.8 (IQR 31.3 to 38.7) years; two had clinical AIDS. After 60 to 120 days of follow‐up, nine PLWHIV were undetectable, nine PLWHIV had low‐level viraemia (<200 copies/mL) and three PLWHIV had detectable viraemia (240, 410, and 1129 copies/mL respectively). A total of five of 84 (6.0%, four naïve, one experienced) PLWHIV discontinued Delstrigo, two for gastrointestinal side‐effects, two for other reasons, and one due to drug interactions. There were no discontinuations because of virological failure.

**Conclusion**: In our real‐life setting treatment with Delstrigo was virologically successful and well tolerated both as a switch strategy or as a first‐line regimen.

**Reference**

1. European AIDS Clinical Society (EACS). EACS guidelines, version 11 October 2021 [Internet]. [cited 2022 Jun 25]. Available from: https://www.eacsociety.org/guidelines/eacs‐guidelines.

#### P109 Treatment‐experienced HIV‐1 infected patients under salvage regimen with dolutegravir and boosted darunavir: a real‐life scenario

L Moura, C Batista, F Duarte, I Neves, R Correia de Abreu, J Laranjinha

Infectious Diseases, Unidade Local de Saúde de Matosinhos ‐ Hospital Pedro Hispano, Matosinhos, Portugal

**Background**: Dual therapy with dolutegravir (DTG) plus boosted darunavir with ritonavir or cobicistat (DRV/rc) is a drug combination with a robust genetic barrier and simple dosing regimen used in HIV‐infected patients, suitable as salvage therapy in multi‐experienced patients or in cases with documented resistance mutations to multiple classes of antiretrovirals.

**Materials and methods**: Retrospective analysis of the effectiveness of the combined use of DTG and DRV/rc in HIV‐1 treatment‐experienced patients followed in a district hospital.

**Results**: Among nearly 900 patients with HIV‐1 and ‐2 infection followed in our hospital, 93% have viral suppression, but a small number have limited therapeutic options. A total of 40 patients are under DTG plus DRV/rc. Among them, 18 females and 22 males, with a sexual risk of transmission observed in 77.5%, an average age of 52 years [36 to 77], 16 years [7 to 24] since HIV‐1 infection diagnosis and 15 years [1 to 23] of exposure to antiretroviral drugs: 47.5% had exposure to protease inhibitors and 60% to integrase inhibitors. Eighteen patients had a history of opportunistic disease with hospitalisation. Previous drug resistance mutations (72.5%), therapeutic simplification (22.5%) and toxicity with prior medication (5%) were the reasons to start DTG+DRV/rc. Mean duration of treatment is 3 years [6 months to 7 years], with the following dosage distribution: 87.5% with DTG 50 mg QD, 12.5% with DTG 50 mg BID, 12.5% with DRV 600 mg BID, 87.5% with DRV 800 mg QD. Globally, 67.5% of patients had cobicistat‐boosted DRV and 32.5% ritonavir‐boosted DRV. Initial mean TCD4+ was 484/mcL [48 to 1109], rising to 524/mcL [46 to 1316] 1 month after the switch and 567/mcL [55 to 1268] in the last assay performed. At the moment of DTG+DRV/rc initiation, 40% were virally suppressed reaching 70% 1 month later and 80% in the last available assay. Currently, 92.5% of patients maintain this combination with excellent tolerability.

**Conclusion**: Despite the progress in HIV therapeutic options, multi‐experienced patients are still a challenge. This study gives an insight into real‐world data from our clinical experience with the combination of DTG and boosted DRV, a valuable option when treating this population.

### Treatment Strategies: Other

#### P110 HIV‐2: the global experience of a Portuguese centre since the beginning of the epidemic

M Pereira, J Borralho, J Vasconcelos, J Alves, S Peres, A Miranda, T Baptista, I Antunes, F Borges, K Mansinho

Infectious Disease and Tropical Medicine Service, Centro Hospitalar Lisboa Ocidental ‐ Hospital Egas Moniz, Lisbon, Portugal

**Background**: HIV‐2 is endemic in West Africa, though some cases have been imported and transmission has occurred outside Africa, mainly in Portugal and France. Compared to HIV‐1, it has a slower decline in TCD4+ lymphocytes, lower viral replication, and a longer asymptomatic stage, which may challenge the diagnostic suspicion in non‐endemic areas. Until 2020, there were 2030 cumulative reported cases in Portugal.

**Material and methods**: Real‐life, retrospective, observational study. Socio‐demographic, clinical, laboratory and therapeutic data were collected from the whole cohort of HIV‐2 infected patients that have been followed in a Portuguese tertiary hospital since the beginning of the HIV‐2 epidemic.

**Results**: Total cohort of 205 HIV‐2 patients, mostly women (63%); mean age of 44 years (±14.3 years); one half originating from Guinea‐Bissau (49%) with the majority assumed as of heterosexual acquisition (86%). Most were remote diagnoses dating back to 1985 to 2009 (77%), while a minority were detected in 2010 to 2019 (18%) and 2020 to 2022 (5%). The diagnosis was mostly related to routine asymptomatic screening (37%), pregnancy screening (18%), or symptomatic HIV‐related disease (9%). Currently, 106 patients are actively followed (29 died and 71 lost follow‐up mainly because of hospital transfers and return to homeland countries). Most patients (88%) are under antiretroviral therapy (ART): mean baseline TCD4+ 364/mm^3^; mean gap between diagnosis and ART of 6 years; mean increase of 46 cells/mm^3^/year and mostly on integrase strand transfer inhibitors based regimen (60%). Thirteen patients have not thus far started on ART because of good immunological status and sustained virological control: mean baseline TCD4+ 749/mm^3^ at diagnosis, slow rate decrease of 17 cells/mm^3^/year, mean current TCD4+ 624/mm^3^, all with undetectable or residual viral load without therapy.

**Conclusions**: HIV‐2 remains a diagnostic challenge, mainly due to the frequent absence of symptoms and low suspicion level for the diagnosis of individuals coming from endemic areas. From our experience, the incidence of infection has been decreasing over the decades, perhaps due to better follow‐up and appropriate treatment in the countries of origin. Our cohort reflects the paradigm of universal treatment of people living with HIV and alerts for the close clinical and laboratory monitoring of those few who have not started treatment yet.

#### P111 High proportion of born‐abroad MSM acquire HIV after migration in France: first results from the ANRS‐MIE GANYMEDE study

A Arias Rodriguez^1^, L Assoumou^1^, O Rousset Torrente^2^, C Lascoux‐Combe^3^, J Ghosn^4^, T Chiarabini^5^, A Velter^6^, M Ben Mechlia^7^, L Béniguel^1^, M Duracinsky^8^, V Supervie^1^, R Palich ^9^

^1^Epidemiology, Sorbonne University, Paris, France; ^2^Epidemiology, University of Paris, Paris, France; ^3^Infectious Diseases, University of Paris, Saint Louis Hospital, Paris, France; ^4^Infectious Diseases, University of Paris, Bichât Hospital, Paris, France; ^5^Infectious Diseases, Sorbonne University, Saint Antoine Hospital, Paris, France; ^6^Epidémiology, Santé Publique France, Paris, France; ^7^Social Sciences, French National Research Agency on HIV/Aids, Hepatitis and Emerging Infectious Diseases (ANRS‐MIE), Paris, France; ^8^Infectious Diseases, University of Paris, Kremlin Bicêtre Hospital, Paris, France; ^9^Infectious Diseases, Sorbonne University, Pitié‐Salpêtrière Hospital, Paris, France

**Background**: Few studies recently showed that rates of new and undiagnosed HIV infections for born‐abroad MSM were high, if not the highest, in several European countries. However, this population remain understudied in most settings.

**Methods**: The French ANRS‐MIE GANYMEDE study is an ongoing cross‐sectional survey conducted in 16 HIV centres in the Paris region, on a random sample of born‐abroad MSM living with HIV. Data on migration history, socioeconomic conditions, sexual activity, health before, after and at the time of migration in France, are collected through self‐administered questionnaires and medical records. Here, we aimed to describe participants’ characteristics at time of migration, and estimated (minimum) proportions of MSM who acquired HIV before and after migrating in France. HIV infection was assumed to have occurred after migration in case of: (i) first sexual intercourse, (ii) negative HIV test, or (iii) diagnosis of primary infection occurrence in France, or in case of migration before the age of 15. It was assumed to have occurred before migration in case of: (i) HIV diagnosis, or (ii) ART start before arriving in France. Otherwise, the timing of HIV acquisition remained unknown.

**Results**: Early June 2022, 911 born‐abroad MSM had been included: 29% originating from Latin America and the Caribbean (LAC), 24% from Europe and North America (ENA), 17% from North Africa (NA), 15% from Asia and Oceania (AO) and 15% from sub‐Saharan Africa (SSA). Most frequent reasons for migration were educational opportunity (36%) and sexual orientation (35%). Among those who migrated after the age of 15 (83%), median age was 27 (IQR 23 to 33), and hardships were frequent with 25% who had no residence permit, 12% no health coverage, 27% no job and 45% had not their own housing. Overall, at least 47% acquired HIV after migration, 26% before, and 27% unknown. This minimum post‐migration HIV acquisition proportion was 26% for MSM originating from LAC, 45% for ENA, 61% for AO, 49% for SSA, and 71% for NA.

**Conclusion**: These high proportions of post‐migration HIV acquisition, and administrative and social insecurity on arrival in France, highlight the need for improved HIV policies targeting MSM migrants.

#### P112 A pooled analysis of eight clinical studies suggests a link between influenza‐like adverse events and pharmacodynamics of the toll‐like receptor 7 agonist vesatolimod

S Riddler^1^, Y Cai^2^, E Vendrame^3^, Y Zheng^4^, L Zhang^5^, D Verrill^6^, X Liu^7^, D SenGupta ^3^, D Lim^8^

^1^Dept. of Medicine, University of Pittsburgh, Pittsburgh, PA, USA; ^2^Biomarkers, Gilead Sciences, Inc., Foster City, CA, USA; ^3^Clinical Development, Gilead Sciences, Inc., Foster City, CA, USA; ^4^Clinical Pharmacology, Gilead Sciences, Inc., Foster City, CA, USA; ^5^Bioinformatics, Gilead Sciences, Inc., Foster City, CA, USA; ^6^Statistical Programming, Gilead Sciences, Inc., Foster City, CA, USA; ^7^Biostatistics, Gilead Sciences, Inc., Foster City, CA, USA; ^8^Medical Safety Science, Gilead Sciences, Inc., Cambridge, UK

**Background**: Vesatolimod is a toll‐like receptor 7 (TLR7) agonist in development for HIV remission. Nonclinical/clinical studies have established preliminary safety/efficacy of vesatolimod, alone or in combination with other agents [1‐4]. Consistent with TLR7 stimulation, vesatolimod may be associated with influenza‐like adverse events (flu‐like AEs). A broader understanding of the relationship between flu‐like AEs and vesatolimod mechanism of action (MoA) is needed to support future combination studies.

**Materials and methods**: Flu‐like AE data were pooled from eight clinical studies in healthy volunteers, people with chronic hepatitis B, and people with HIV. A subset of flu‐like AEs, selected based on clinical significance and termed 'flu‐like AEs of interest' (flu‐like AEI), was also analyzed (Table 1). To understand mechanisms underlying flu‐like AEI, pharmacokinetics (PK), inflammatory biomarkers, and pharmacodynamics (PD) were analyzed.

**Figure:**
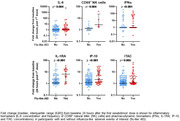
**Abstract P112 – Figure 1**. Inflammatory and pharmacodynamic biomarkers of vesatolimod in participants with and without influenza‐like adverse events of interest. Fold change [median, interquartile range (IQR)] from baseline 24 hours after the first vesatolimod dose is shown for inflammatory biomarkers [IL‐6 concentration and frequency of CD69+ natural killer (NK) cells] and pharmacodynamic biomarkers (IFNα, IL‐1RA, IP‐10, and ITAC concentrations) in participants with and without influenza‐like adverse events of interest (flu‐like AEI).

**Table:** 

**Abstract P112 – Table 1**. MedDRA Preferred Terms selected for analysis of influenza‐like adverse events and influenza‐like adverse events of interest. MedDRA Preferred Terms used to define influenza‐like adverse events (flu‐like AEs) and influenza‐like adverse events of interest (flu‐like AEI) are shown. The list of flu‐like AEI was selected on the basis of potential clinical significance given the preferred term and adverse event grade.	
Influenza‐like adverse events (all grades)	Influenza‐like adverse events of interest
**Cytokine release syndrome**	**Cytokine release syndrome ‐ any grade**
Hemophagocytic lymphohistiocytosis	
Cytokine storm	
Capillary leak syndrome	
Pyrexia	Pyrexia ‐ any grade
Chills	Chills ‐ any grade
Headache	Headache grade ≥2
Fatigue	Fatigue grade ≥2
Cancer fatigue	
Arthralgia	
Nausea	
Vomiting	
Systemic inflammatory response syndrome	
Hypotension	
Hypoxia	
Influenza‐like illness	Influenza‐like illness ‐ any grade
Myalgia	Myalgia grade ≥2
Influenza	Influenza ‐ any grade
Malaise	Malaise grade ≥2

**Results**: Six hundred and six participants received single or multiple vesatolimod doses (0.3 mg to 12 mg, N = 505) or placebo (N = 101). Some participants experienced flu‐like AEs (vesatolimod: 38.6%; placebo: 30.7%), mostly low grade (vesatolimod: 78.0% grade 1, 19.1% grade 2; placebo: 88.9% grade 1, 11.1% grade 2). Flu‐like AEI were more common with vesatolimod versus placebo (19.0% vs 7.9%). The most common flu‐like AEI were pyrexia, chills, and headache. Flu‐like AEI frequency increased with vesatolimod dose and exposure and was highest after doses 1 and 2. Early occurrence of flu‐like AEI (after doses 1 to 3) was predictive of occurrence after later doses (p < 0.01). There were no dose‐dependent changes in inflammatory biomarkers (CRP, IFNγ, IL‐6, TNF). Dose‐dependent elevation of PD biomarkers (IFNα, IL‐1RA, IP‐10, ITAC) was observed. Some inflammatory and PD biomarkers were higher in participants with versus without flu‐like AEI (IL‐6 p = 0.004, CD69+NK p = 0.004, IFNα p < 0.001, IL‐1RA p < 0.001, IP‐10 p < 0.001, ITAC p = 0.005, Figure 1).

**Conclusions**: Vesatolimod is generally safe and well tolerated. In some individuals, vesatolimod is associated with flu‐like AEs. Flu‐like AEI may be predicted by response to initial doses, supporting adaptive clinical monitoring. Flu‐like AEI increased with vesatolimod exposure and elevated PD biomarkers, suggesting a link with MoA. The relationship between vesatolimod PD and efficacy in the setting of HIV remission strategies will be explored in future trials.

**References**

1. Borducchi EN, Cabral C, Stephenson KE, Liu J, Abbink P, Ng'ang'a D, et al. Ad26/MVA therapeutic vaccination with TLR7 stimulation in SIV‐infected rhesus monkeys. Nature. 2016;540:284‐7.

2. Borducchi EN, Liu J, Nkolola JP, Cadena AM, Wen‐Han Yu W‐H, Fischinger S, et al. Antibody and TLR7 agonist delay viral rebound in SHIV‐infected monkeys. Nature. 2018;563:360‐4.

3. Walker‐Sperling VE, Mercado NB, Chandrashekar A, Borducchi EN, Liu J, Nkolola JP, et al. Therapeutic efficacy of combined active and passive immunization in SHIV+ macaques. Conference on Retroviruses and Opportunistic Infections; 2022 Feb 12‐16; Seattle, WA, USA. Abstract 63.

4. SenGupta D, Brinson C, DeJesus E, Mills A, Shalit P, Guo S, et al. The TLR7 agonist vesatolimod induced a modest delay in viral rebound in HIV controllers after cessation of antiretroviral therapy. Sci Transl Med. 2021;13:eabg3071.

#### P113 High rates of central nervous system adverse events among patients on dolutegravir‐based regimens in Uganda

E Laker Odongpiny ^1^, E Katana^2^, J Owori^3^, K Seden^4^, D Meya^5^, M Nicol^6^, M Kesby^7^, M Holden^8^, D Sloan^8^, C Sekaggya^5^

^1^Prevention, Care and Treatment, Infectious Diseases Institute, Kampala, Uganda; ^2^Clinical Epidemiology Unit, College of Health Sciences, Makerere University, Kampala, Uganda; ^3^Pharmacy School, Clinical Epidemiology Unit, College of Health Sciences, Makerere University, Kampala, Uganda; ^4^Department of Molecular and Clinical Pharmacology, University of Liverpool, Liverpool, UK; ^5^Infectious Diseases Institute, Makerere University, Kampala, Uganda; ^6^Experimental and Clinical Pharmacology, University of Minnesota, Minneapolis, MN, USA; ^7^Department of Geography and Sustainable Development, University of St Andrews, Fife, UK; ^8^Division of Infection and Global Health, School of Medicine, University of St Andrews, Fife, UK

**Background**: Dolutegravir (DTG) was recommended by WHO in 2017 as a preferred component of antiretroviral therapy for countries like Uganda with high rates of resistance to non‐nucleotide reverse transcriptase inhibitors (NNRTIs) [1]. Observational data from non‐black populations reported new adverse events (AEs), including weight gain and insomnia, which were not previously described in clinical trials [2,3]. Programmatic, observational safety data on DTG amongst people living with HIV (PLWHIV) in Africa are lacking.

**Materials and methods**: We carried out a retrospective review of patients who had been switched to, or newly initiated on, DTG‐based ART from 2017 to 2020 at the Infectious Diseases Institute in Kampala, Uganda. Demographic, clinical, and AE data were extracted from paper files and our clinic's electronic database. AEs were classified according to MedDRA preferred terminology and organ system classification. We used Modified Poisson regression analysis to identify factors associated with having any AE.

**Results**: Four thousand, five hundred and sixty patients (45.9% female) with the median age (IQR) of 45.0 (15 to 81) years were switched to (4463/4560; 97.9%) or initiated on (97/4560; 2.1%) DTG. The majority were of normal BMI (18.5 to 24.9) (58.8%), 2.7% were diabetic and 16.8% were hypertensive. 20.7% (946/4560) experienced at least one AE. The most common AEs by organ system and type were: nervous system (e.g. headaches [149/946; 15.8%]); gastrointestinal (e.g. abdominal pain [57/946; 6%]); endocrine (e.g. new or worsening hyperglycaemia [108/946; 11.4%]), musculoskeletal (e.g. arthralgia [60/946; 6.3%]), skin (e.g. pruritic rash [40/946; 4.2%]), reproductive system and breast disorders (e.g. erectile dysfunction [18/946; 1.9%]) and general systems (e.g. weight gain [32/946; 3.4%]). Being male was associated with lower risk of experiencing any AE (adjusted PR 0.73; 95% CI 0.64 to 0.82) whilst age ≥60 years increased the risk (adjusted PR 2.23; 95% CI 1.58 to 3.15). One hundred and fifty‐one (3.3%) patients discontinued DTG in the study period. The commonest reasons for this were hyperglycaemia (104/151; 68.9%), concerns about teratogenicity in women of child bearing age (25/151; 16.6%), erectile dysfunction (10/151; 6.6%), gynaecomastia (2/151; 1.3%) and headaches (2/151; 1.3%).

**Conclusion**: A large number of PLWHIV initiating or switching to DTG experienced nervous system AEs. Emerging signals like hyperglycaemia and erectile dysfunction need furthur investigation.

**References**

1. World Health Organization. Transition to New Antiretrovirals in HIV Programmes [Internet]. 2017 [cited 2018 Oct]. Available from: http://apps.who.int/iris/bitstream/10665/255888/1/WHOHIV‐2017.20‐eng.pdf?ua=1.

2. Hoffmann C, Welz T, Sabranski M, Kolb M, Wolf E, Stellbrink HJ, et al. Higher rates of neuropsychiatric adverse events leading to dolutegravir discontinuation in women and older patients. HIV Med. 2017;18:56‐63.

3. Menard A, Montagnac C, Solas C, Meddeb L, Dhiver C, Tomei C, et al. Neuropsychiatric adverse effects on dolutegravir: an emerging concern in Europe. AIDS. 2017;31:1201‐3.

#### P114 Effect of 12‐week cART on gut mucosal immunity and microbiome in primary HIV infection (PHI)

C Tincati ^1^, V Bono^1^, M Falleni^1^, D Tosi^1^, V Yellenki^1^, R Rovito^1^, S Rusconi^2^, A Giacomelli^3^, A Muscatello^4^, S Dispinseri^5^, A d'Arminio Monforte^1^, A Calcagno^6^, S Nozza^5^, A Gori^7^, G Marchetti^1^

^1^Department of Health Sciences, University of Milan, Milan, Italy; ^2^3UOC Malattie Infettive, ASST Ovest Milanese, Legnano, Italy; ^3^III Division of Infectious Diseases, Azienda Socio Sanitaria Territoriale Fatebenefratelli Sacco, Milan, Italy; ^4^Infectious Diseases Unit, Foundation Istituto di Ricovero e Cura a Carattere Scientifico Ca' Granda Ospedale Maggiore Policlinico, Milan, Italy; ^5^Viral Evolution and Transmission Unit, Istituto di Ricovero e Cura a Carattere Scientifico Ospedale San Raffaele, Milan, Italy; ^6^Department of Medical Sciences, University of Turin, Turin, Italy; ^7^Division of Infectious Diseases, Azienda Socio Sanitaria Territoriale Fatebenefratelli Sacco, Milan, Italy

**Background**: The short‐time effects of cART in PHI on mucosal immune and microbiome imbalances in the gastrointestinal (GI) tract are largely unknown.

**Materials and methods**: Eleven PHI subjects were studied at T0 and T12 weeks of cART and compared to 10 naive chronically infected individuals (C‐Naïve). Intestinal mucosal morphology, M1/M2 macrophage polarisation (IHC) as well as microbiome (MiSeq Illumina) were studied on gut biopsies. Th17 and Treg cells were assessed in GI and PBMCs (flow cytometry). Gut integrity (E‐cadherin) and peripheral inflammation (sCD14, IL‐6) were measured in plasma (ELISA).

**Results**: Collagen deposition, reduction of E‐cadherin expression as well as increased luminal accumulation of monocyte and M1/2 macrophages was found in untreated PHI. These features resembled those of C‐Naïve, although less pronounced (Figure 1). At T0, PHI displayed high variability in gut microbiome composition which was markedly greater than that of C‐Naïve. Untreated PHI and C‐Naïve showed comparable Th17 and Treg cells in gut and PBMCs. In contrast, at T0, PHI showed significantly lower plasma sCD14 (4.54 ug/mL, IQR 3 to 5 vs 5.6 ug/mL, IQR 5 to 5.9; p = 0.03) compared to C‐Naïve as well as a trend to lower IL‐6 and higher E‐cadherin. In PHI, cART was introduced at 12 (IQR 9 to 24) days from PHI diagnosis and lead to viral decay (T0: log10 5.58 copies/mL, 5.16 to 5.92; T12: log10 1.59 copies/mL 1.56 to 1.73, p < 0.0001) and CD4+ reconstitution (T0: 538/mmc, 446 to 615; T12: 756/mmc, 681 to 938, p = 0.0003). Alterations of gut structure progressed at T12 regardless cART (Figure 1). This was accompanied by a progressive decrease in gut microbial variability as well as a significant increase of Tregs in ileum (T0: 0.43 ug/mL, IQR 0.21 to 0.75; T12: 1.25 ug/mL, IQR 0.75 to 3.95; p = 0.001) and PBMCs (T0: 2.6 ug/mL, IQR 1.16 to 4.69; T12: 5.9 ug/mL, IQR 4.67 to 8.67; p = 0.004), with no effect of Th17 cells and peripheral markers of immune activation/inflammation (Table 1).

**Conclusions**: Untreated PHI show gut damage with stromal, immune and microbiome alterations, resembling those of C‐Naïve, yet less pronounced. Similarly, untreated PHI also display lower systemic activation than C‐Naïve. Twelve‐week cART appears to have little/no effect on gut impairment; however, it appears to hinder systemic immune activation. Longer follow‐up of treated PHI is needed to assess the effects of early cART in the gastrointestinal tract.

**Table:** 

**Abstract P114 – Table 1**. Demographical and clinical characteristics of PHI and C‐Naïve patients.			
Characteristic	PHI (N = 11)	Chronic naive (N = 10)	p‐value
Age at diagnosis [years], median (IQR)	41 (29 to 45)	42 (34 to 51)	0.3408
Male, sex (%)	11 (100)	8 (80)	0.2143
Ethnicity, n (%)			0.3108
Caucasian	10 (91)	7 (70)	
Other	1 (9)	3 (30)	
Epidemiology, n (%)			0.5865
MSM	10 (91)	8 (80)	
Heterosexual/unknown	1 (9)	2 (20)	
HBV/HCV coinfection, n (%)	3 (27)	3 (30)	1
Nadir CD4 [cell/mmc], median (IQR)	538 (493 to 609)	147 (12 to 279)	0.0006
CD4 count at colonoscopy [cell/mmc], median (IQR)	538 (446 to 651)	176 (94 to 279)	0.0008
CD4% at colonoscopy, median (IQR)	25 (14 to 28)	17 (10 to 18)	0.1295
CD8 count at colonoscopy [cell/mmc], median (IQR)	1332 (826 to 1752)	688 (248 to 925)	0.0183
CD8% at colonoscopy, median (IQR)	56 (49 to 72)	58 (49 to 66)	0.5256
CD4/CD8 ratio at colonoscopy, median (IQR)	0.50 (0.19 to 0.57)	0.26 (0.15 to 0.32)	0.1693
HIV‐RNA at colonoscopy [log10 copies/mL], median (IQR)	5.58 (5.16 to 5.92)	5.13 (4.80 to 5.42)	0.0528
Fiebig stage, n (%)			/
I to III	2 (18)	/	
IV to VI	9 (82)	/	
CDC stage, n (%)			0.0560
A1 to A3	9 (82)	3 (30)	
B1 to B3	1 (9)	3 (30)	
C1 to C3	1 (9)	4 (40)	
First HAART regimen, n (%)			/
INSTI‐based	6 (55)	/	
PI‐based	3 (27)	/	
Mega‐HAART (INSTI+PI)	2 (18)	/	

**Figure:**
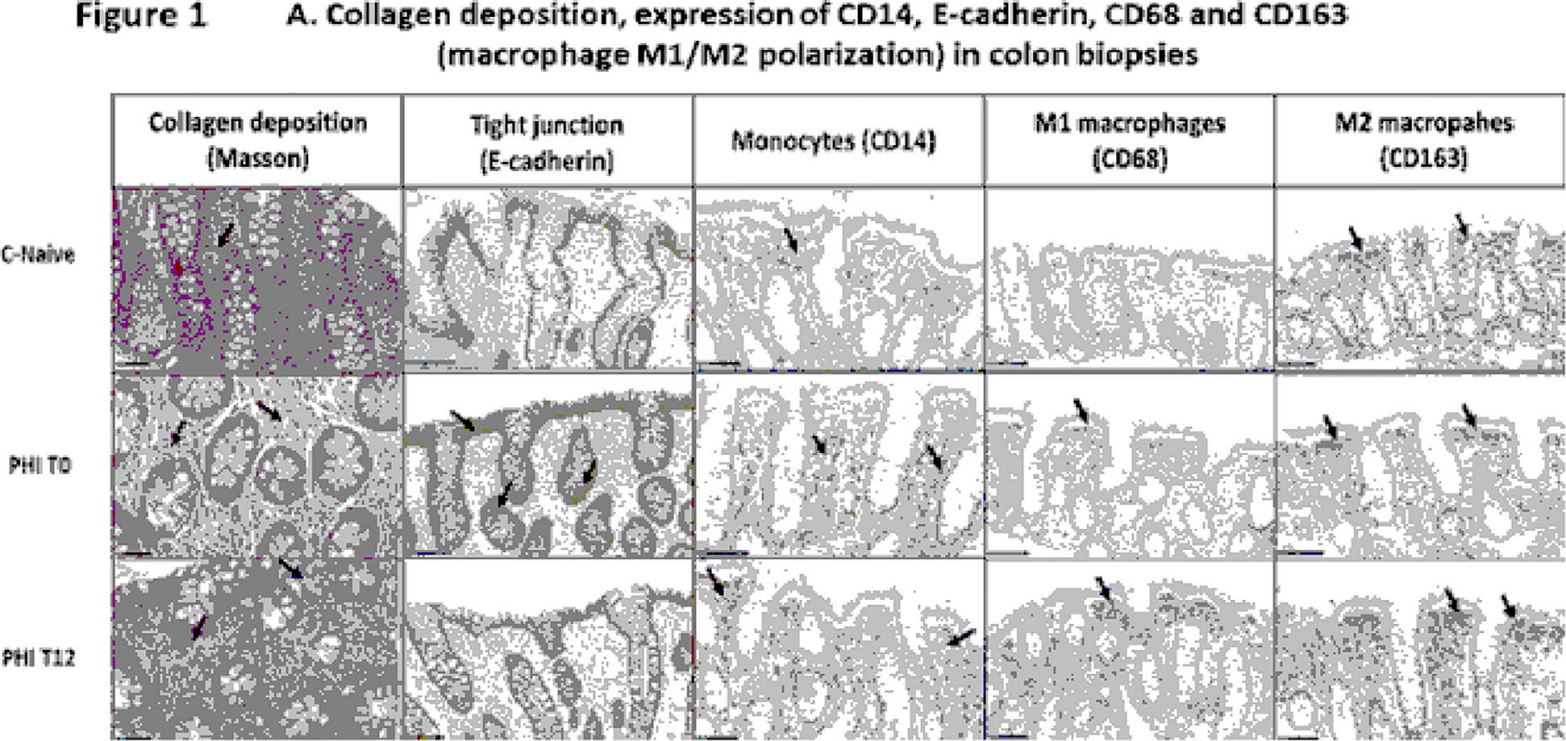
**Abstract P114 – Figure 1**. Collagen deposition, expression of CD14, E‐cadherin, CD68 and CD163 (macrophage M1/M2 polarisation) in colon biopsies.

#### P115 Abstract withdrawn

#### P116 Overcoming barriers and achieving optimal implementation of cabotegravir and rilpivirine long‐acting (CAB+RPV LA): staff study participant (SSP) results from the CAB+RPV Implementation Study in European Locations (CARISEL)

L Slama ^1^, M Crusells‐Canales^2^, J Olalla^3^, R DeMoor^4^, M Ait‐Khaled^5^, S Dakhia^6^, H Van Mechelen^7^, A Hamilton^8^, M Hadi^9^, D Filipenko^9^, F Brown^9^, E Low^9^, C Gutner^10^, M Czarnogorski^11^

^1^Infectious Diseases Unit, Hôtel‐Dieu Hospital, Assistance Publique Hôpitaux de Paris (APHP), Paris, France; ^2^Infectious Disease Service, Hospital Clínico Lozano Blesa, Zaragoza, Spain; ^3^Internal Medicine Department, Costa del Sol Hospital, Marbella (Málaga), Spain; ^4^R&D Biostatistics, GlaxoSmithKline, Collegeville, PA, USA; ^5^Clinical Sciences, R&D, ViiV Healthcare Ltd, Brentford, UK; ^6^Medical Affairs, ViiV Healthcare Ltd, Brentford, UK; ^7^Medical Affairs, ViiV Healthcare SRL, Wavre, Belgium; ^8^Psychiatry, University of California (LA), Los Angeles, CA, USA; ^9^Patient‐Centered Research, Evidera, London, UK; ^10^Global Medical Sciences, Innovation and Implementation Science, ViiV Healthcare Ltd, Durham, NC, USA; ^11^Global Medical Sciences, ViiV Healthcare, Durham, NC, USA

**Background**: Cabotegravir (CAB) and rilpivirine (RPV) is the first complete long‐acting (LA) regimen for the maintenance of HIV‐1 virological suppression, presenting a novel treatment paradigm. CARISEL, an implementation–effectiveness study, examines strategies to support implementation of CAB+RPV LA dosed every 2 months across European countries. Barriers and strategies for optimal implementation are reported herein.

**Materials and methods**: Quantitative and qualitative data on barriers and facilitators for optimal implementation were collected from staff study participants (SSPs) across 18 sites at month (M) 1, M5, and M12 (Table 1).

**Table:** 

**Abstract P116 – Table 1**. SSP sample size for quantitative and qualitative data by time point.			
	Month 1	Month 5	Month 12
Quantitative data	70	68	62
Qualitative interviews	70	68	62

SSP, staff study participant.

**Results**: All top five barriers reported at M1 markedly decreased by M12, including risk of resistance for non‐adherent patients (M1, 35% [n = 24/69]; M12, 16% [n = 10/62]), enough injectors (M1, 35% [n = 24/69]; M12, 24% [n = 15/62]), injection pain/soreness (M1, 33% [n = 23/69]; M12, 16% [n = 16/62]), scheduling around patient holidays (M1, 32% [n = 22/69]; M12, 8% [n = 5/62]), and patients being non‐virologically suppressed due to missed dose (M1, 30% [n = 21/69]; M12, 15% [n = 9/62]). At M12, quantitative data showed most SSPs (76%, n = 47/62) felt 'very' or 'extremely positive' about implementing CAB+RPV LA, and qualitative data revealed the most frequently reported SSP facilitator of acceptability was a positive opinion about CAB+RPV LA (90%, n = 56/62). At M1 and M12, SSPs believed CAB+RPV LA was appropriate for a wide variety of patients including, but not limited to, those who are tired of daily pills, concerned with disclosure of HIV status, and experienced stress regarding daily pill compliance. Top tips (>50%) for reducing pain included: slow speed of pushing the injection (60%, n = 37/62), relaxed muscle (53%, n = 33/62), and room temperature medication (57%, n = 35/62). During M12 interviews, SSPs (32%, n = 20/62) discussed the information they wished they had before starting the study, including on pain/soreness to discuss with patients before injections, additional information about medication preparation/injections, the level of patient interest in CAB+RPV LA, and treatment complexity.

**Conclusions**: SSP data demonstrated that perceptions of implementation barriers decreased over time and perceptions about CAB+RPV LA were positive throughout implementation. Tips for optimising implementation success include using techniques to minimise injection discomfort and education about the treatment. SSPs recognised that a wide range of patients were appropriate for CAB+RPV LA.

#### P117 Two‐year outcomes of dolutegravir (DTG) + lamivudine (3TC) in ART‐naïve and pre‐treated people living with HIV in Germany: real‐world data from the German URBAN cohort

D Beer ^1^, J Scherzer^2^, S Noe^3^, S Scholten^4^, C Wyen^5^, N Postel^6^, O Degen^7^, M Sabranski^8^, B Westermayer^9^, K Dymek^2^

^1^Clinical Care, PZB Aachen ‐ Praxis Dr. H. Knechten, Aachen, Germany; ^2^Medical Affairs, ViiV Healthcare, Munich, Germany; ^3^Clinical Care, MVZ München am Goetheplatz, Munich, Germany; ^4^Clinical Care, Praxis Hohenstaufenring, Cologne, Germany; ^5^Clinical Care, Praxis Ebertplatz, Cologne, Germany; ^6^Clinical Care, Prinzmed, Munich, Germany; ^7^Clinical Care, Universitätsklinikum Hamburg‐Eppendorf, Hamburg, Germany; ^8^Clinical Care, ICH Study Center, Hamburg, Germany; ^9^Medical Affairs, GlaxoSmithKline GmbH, Munich, Germany

**Background**: Although evidence from clinical trials with DTG+3TC for first‐line therapy or maintenance of viral suppression has increased remarkably during recent years, experience from clinical routine is needed to complement the findings for diverse populations in various real‐world settings. The URBAN cohort study provides prospective real‐world data on the effectiveness, tolerability, metabolic parameters and patient‐reported outcomes (PROs) in people living with HIV (PLWHIV) using DTG+3TC. Here we present the 2‐year results.

**Materials and methods**: URBAN is a prospective, non‐interventional, 3‐year German cohort study in ART‐naïve and pre‐treated PLWHIV receiving DTG+3TC in accordance with the label. Two‐year viral suppression was defined as HIV‐RNA <50 copies/mL in visit window (21 to 27 months) or 50 to 200 copies/mL with subsequent HIV‐RNA <50 copies/mL (discontinuation=failure; excluding missing data/lost to follow‐up). Persistence on DTG+3TC was estimated using Kaplan‐Meier analysis. Treatment satisfaction and symptom burden were assessed by the HIV treatment satisfaction questionnaire (status version; HIV‐TSQs) and the HIV Symptom Distress Module (HIV‐SDM).

**Results**: At data‐cut, 352 PLWHIV were eligible for analysis (91% pre‐treated, 93% men, and median age at baseline 47 years). Persistence on DTG+3TC through year 2 was 89%; 37 individuals (11%) discontinued DTG+3TC in total; and 13 (4%) were lost to follow‐up. Two‐year viral suppression rate was 88% for pre‐treated and 86% for ART‐naïve PLWHIV (Figure 1). Overall, four PLWHIV (1%; three pre‐treated, one ART‐naïve) discontinued DTG+3TC for virological reasons at investigator's discretion with VL <200. No emergent resistance was reported. Median weight change from baseline at year 2 was +2.0 kg (IQR ‐1.0 to +4.0; n = 127) in pre‐treated, and +5.0 kg (IQR +2.0 to +10.0; n = 17) in ART‐naïve PLWHIV. In pre‐treated PLWHIV completing questionnaires at baseline and year 2, mean total HIV‐TSQs score increased significantly, from 53.5 to 56.7 (5.1), with a change of +3.2; the total HIV‐SDM score was relatively stable in this group (Table 1).

**Figure:**
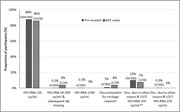
**Abstract P117 – Figure 1**. Virological outcomes at year 2 (effectiveness set: N = 328; n = 24/352 excluded due to missing data). ^ At investigator's discretion with HIV‐RNA <200 copies/mL (83, 89, 95 and 128 copies/mL); ^^ most common reasons (in >1% of participants) were patient decision (n = 15) and adverse drug reactions (ADRs; n = 13); n = 1 death, not related to DTG+3TC. LOCF, last observation carried forward.

**Table:** 

**Abstract P117 – Table 1**. HIV‐SDM and HIV‐TSQs for participants completing both questionnaires (at baseline and year 2); in ART‐naïve PLWHIV, the mean total HIV‐SDM scores at BL and year 2 were 12.1 (SD 14.1) and 12.2 (12.6; n = 11; p = 0.964), respectively; mean HIV‐TSQs at year 2 was 57.0 (SD 3.2; n = 12).					
	Pre‐treated, N	Baseline total score, mean (SD)	Year 2 total score, mean (SD)	Year 2 change from baseline, mean (SD)	p‐value (Wilcoxon sign rank test)
HIV‐SDM^a^	144	14.0 (11.5)	13.0 (11.9)	−1.0 (11.4)	0.258
HIV‐TSQs^b^	140	53.5 (8.4)	56.7 (5.1)	+3.2 (8.1)	<0.001

SD, standard deviation.

^a^HIV‐SDM: 20 items, range of total score 0 to 80; negative changes indicate improvement;

^b^HIV‐TSQs: range of total score 0 to 60; positive changes indicate improvement.

**Conclusion**: Prospective real‐world data from the URBAN cohort showed high virological suppression rates after 2 years on DTG+3TC with low numbers of discontinuations for virological reasons. In pre‐treated PLWHIV, a statistically significant improvement in treatment satisfaction was observed after 2 years of DTG+3TC use.

#### P118 Contributions to HIV cure research from Africa: a systematic review

G Kyei, E Bonney, J Aboagye, H Lamptey, C Abana, A Boateng, S Kaminta, P Wormenor, C Forfoe, C Bortey, P Nartey, D Attoh, P Boakye, K Sedzro

Virology, Noguchi Memorial Institute for Medical Research, University of Ghana, Accra, Ghana

**Introduction**: A cure is desirable to end the HIV/AIDS pandemic due to non‐adherence to medications, incomplete viral suppression, drug resistance, unsustainable costs and drug side effects. The past decade has seen intensive research into the mechanisms of HIV latency, persistence, and cure resulting in a number of early phase clinical trials. However, although close to 70% of people living with HIV (PLWHIV) are in Africa, a cursory look at the literature suggests that very little of the cure research happens on the continent. It is critical to undertake cure research in Africa because of the variety of subtypes, different immunological and genetic patient characteristics, and exposure to different pathogens, which may all influence the character of the HIV reservoir. In addition, people in Africa may have different attitudes and reception to interventions such as analytical treatment interruption needed to evaluate new cures.

**Materials and methods**: We reviewed original HIV cure‐related publications from 2010 to 2021 indexed in the PubMed database using the Preferred Reporting Items for Systematic Reviews and Meta‐analyses protocol as a guide. We excluded reviews, vaccine studies or other studies not related to cure. We studied African author proportions, study type, participant origin, and funding sources.

**Results**: Of the 518 studies that met criteria, 253 (49%) were performed in North America, 28% in Europe and 11% involved African participants. Only 40 (7.7%) papers had an author affiliated with an African institution of which 30 were either first, second or corresponding author. The African institution‐affiliated studies were mostly from South (26/40) and West Africa (8/40). Compared to North America, all types of studies (basic, ex vivo, animal, clinical trials) were significantly less likely to be performed in Africa (p < 0.001) except for epidemiology/social studies. Mainly the National Institutes of Health (54.6%), Bill and Melinda Gates Foundation (7.7%) and amfAR (7.1%) funded HIV cure studies.

**Conclusion**: The study shows that African researchers and patients are contributing little to HIV cure research. There is an urgent need for more HIV cure research investments in Africa to ensure that emerging curative therapies are appropriate for PLWHIV in Africa.

#### P119 HIV resistance patterns in a cohort of adults living with HIV failing first‐line efavirenz‐based antiretroviral therapy in South Africa

B Bosch, S Sokhela, J Woods, E Bhaskar, K Moller, N Manentsa, F Venter

Health Sciences, Ezintsha, Johannesburg, South Africa

**Background**: Human immunodeficiency virus (HIV) drug resistance poses a serious threat to antiretroviral therapy (ART) regimens. The ADORE study was designed to assess the efficacy of doravirine‐based ART in people living with HIV (PLWHIV) experiencing virological failure on efavirenz‐based first‐line ART in Johannesburg, South Africa. This paper reviews the resistance patterns obtained thus far, with particular reference to doravirine, in participants screened for the aforementioned study.

**Methods**: We are in the process of conducting a single‐arm, phase III, switch study to assess the efficacy of doravirine/lamivudine/tenofovir disoproxil fumarate in PLWHIV experiencing virological failure on first‐line efavirenz‐based antiretroviral therapy with non‐nucleoside reverse transcriptase inhibitor (NNRTI) resistance. HIV genotypic drug resistance profiles are obtained at screening visits and major drug resistant mutations (DRMs) scored using the Stanford University HIV Drug Resistance Database, with the corresponding algorithm used to predict drug susceptibility.

**Results**: A total of 40 individuals have been screened to date, with HIV genotypic drug resistance profiles obtained in 21 of those with unsuppressed viral loads. Of these participants, all were black, and 76.1% were female. The mean age was 35 years, and the mean baseline viral load 64 350 copies/mL with participants on ART for an average of 6 years. At screening, major NNRTI resistance mutations associated with the highest levels of reduced susceptibility to doravirine (i.e. M230L, V106M and Y188L) were present in 47.6% (n = 10) of participants, although 95.2% (n = 20) exhibited varying levels of resistance to doravirine. The most common mutations present included M184V (95.2%, n = 20) and K103N (71.4%, n = 15). High‐level resistance to both efavirenz and nevirapine was detected in 90.5% of participants, and full rilpivirine susceptibility maintained in 33.3% (n = 7).

**Conclusions**: Despite a small sample size, these insights offer valuable information into the resistance patterns of PLWHIV in South Africa, failing NNRTI‐based first‐line ART regimens. Cross‐resistance within NNRTIs may be more prevalent with doravirine than recorded in previous clinical trials, this potentially compromising their role as an option for patients failing EFV‐based regimens, particularly with the introduction of dolutegravir into first‐line ART programmes in South Africa.

#### P120 HIV testing training for non‐HIV specialists in a tertiary hospital: change in attitudes and rates of HIV screening

A García García ^1^, J Martínez‐Sanz^1^, M Vivancos Gallego^1^, C Cano^1^, B Romero^2^, M Sánchez‐Conde^1^, M Vélez Díaz‐Pallarés^2^, M González Vázquez^1^, F Gea Rodríguez^3^, J Galán Montemayor^4^, M Pérez‐Elías^1^

^1^Infectious Diseases Department, Hospital Ramón y Cajal, Madrid, Spain; ^2^Pharmacy Department, Hospital Ramón y Cajal, Madrid, Spain; ^3^Gastroenterology Department, Hospital Ramón y Cajal, Madrid, Spain; ^4^Microbiology Department, Hospital Ramón y Cajal, Madrid, Spain

**Background**: National and international HIV testing guidelines are poorly known and complied by non‐HIV specialists. Increasing awareness is essential to improve HIV screening. Few programmes targeting these groups have been reported.

**Materials and methods**: In a tertiary hospital, six infectious diseases physicians provided a 1‐hour personalised training session on HIV screening to each of the other departments. A brief questionnaire was used before and after the training to evaluate attitudes towards HIV screening. We compared the absolute number of tests requested, the screening rate per 1000 patients attended, and new HIV diagnoses in the 6‐month period before and after training, for each hospital department. Information on test requests and newly confirmed HIV diagnoses were obtained from the Microbiology and Infectious Diseases departments respectively. The number of patients attended was obtained from the hospital management systems.

**Results**: From March to November 2019, a total of 346 non‐HIV specialists (90% <55 years, 59% female), from 31 hospital departments (17 medical, 14 surgical), were trained and answered the questionnaire. According to the pre‐training questionnaire, only 20% of non‐HIV specialists (28% medical vs 8% surgical, p < 0.001) were aware of HIV testing guidelines (5% ordered routinely, 60% with any obvious exposure risk or indicator conditions, and 35% never did so). In post‐training responses, 98% considered the training received useful, and responses showing a positive attitude towards routine HIV testing increased to 20%, while those who never requested tests decreased to 2% (p < 0.001). Out of an estimated number of 785 499 patients attended in both periods, we observed a 24% increase in HIV tests requested (p < 0.001), significant in both medical and surgical services; however, this increase was driven by six medical and one surgical department (Figure 1). An increase in new HIV diagnoses in the post‐training period (25 vs 37) was also seen, although only in medical departments (19 vs 32; p = 0.068).

**Conclusions**: Non‐HIV specialists reported poor knowledge of HIV screening, being worse in surgical departments. Directed training was considered useful and significantly improved some attitudes towards HIV testing. We observed an increase in HIV testing coverage and its effectiveness, with marked differences between departments.

**Figure:**
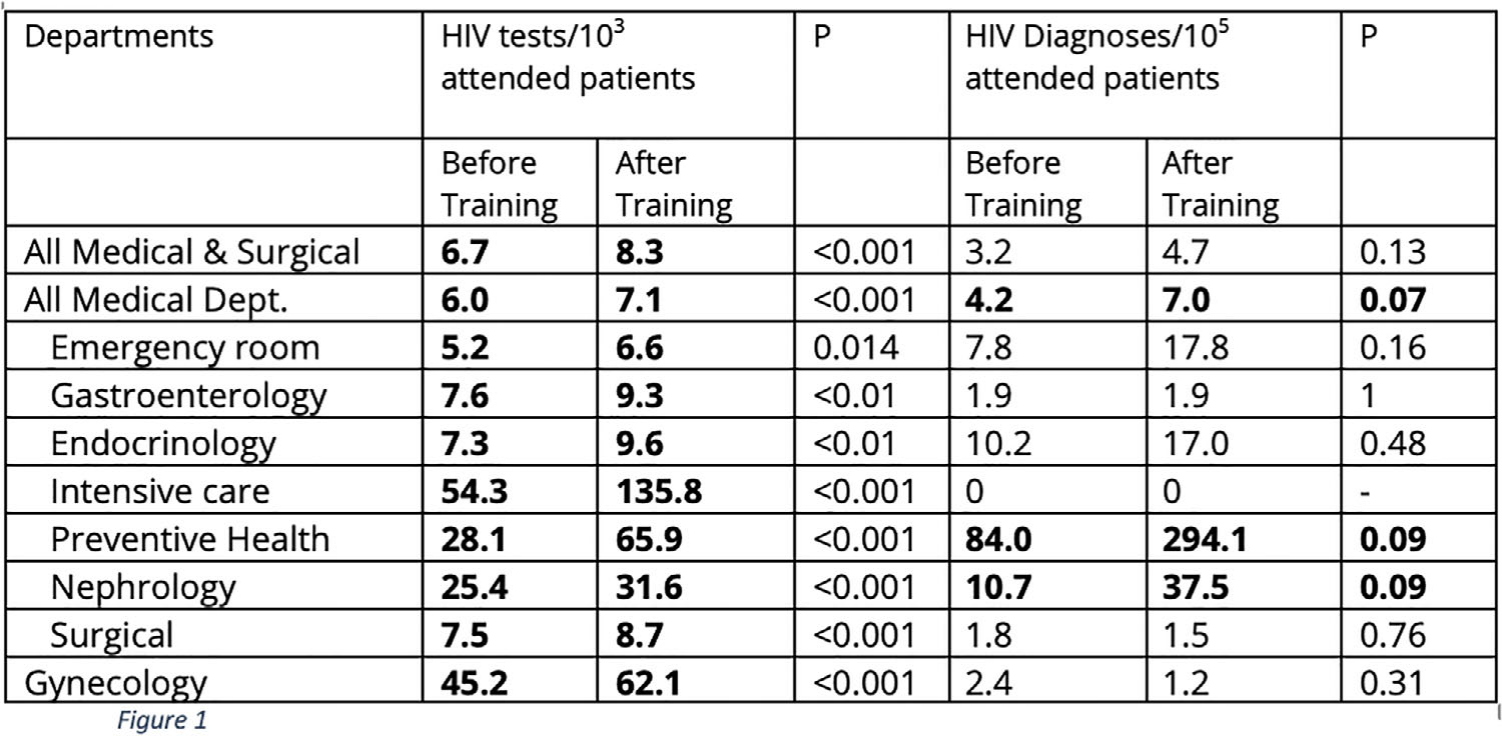
**Abstract P120 – Figure 1**. Distribution of HIV tests and diagnoses between departments before and after the training.

#### P121 Knowledge, attitudes, and practices in chronic inflammation and cardiovascular risk associated with HIV infection in Spain

S Serrano‐Villar ^1^, J Moltó^2^, M Montero‐Alonso^3^, C Diaz Torné^4^, L Pérez de Isla^5^

^1^Infectious Diseases, Ramón y Cajal Hospital, Madrid, Spain; ^2^Infectious Diseases, Germans Trias i Pujol Hospital, Badalona, Spain; ^3^Infectious Diseases, La Fe University Hospital, Valencia, Spain; ^4^Rheumatology, Santa Creu i Sant Pau Hospital, Barcelona, Spain; ^5^Cardiology, San Carlos Clinic Hospital, Madrid, Spain

**Background**: People with human immunodeficiency virus (PWH) exhibit increased inflammation compared to individuals without HIV [1‐3], and this is associated with an enhanced risk of comorbidities, suggesting that inflammation constitutes a contributing risk factor [4,5]. However, physician awareness about inflammation in PWH remains unclear, and specific evidence‐based clinical recommendations are lacking. Therefore, we analysed the knowledge, attitudes, and practices in inflammation among doctors treating PWH, with respect to other specialists treating inflammation directly (rheumatologists) or its cardiovascular consequences (cardiologists).

**Materials and methods**: A committee of HIV specialists, a cardiologist, and a rheumatologist designed a 16‐item questionnaire with sections on knowledge, attitude, and practices and 12 HIV‐specific questions for doctors treating PWH. A total of 405 participants (135 physicians per specialty) stratified by Spanish geography, hospital size, and number of PWH under care completed the survey.

**Results**: Doctors treating PWH scored higher on knowledge of inflammation than cardiologists and rheumatologists (5.5±1.4 out of 8 points vs 5.2±1.3 and 4.6±1.4 points, respectively; p < 0.05) (Figure 1A). Nevertheless, rheumatologists showed the most proactive attitude towards inflammation, followed by cardiologists and doctors treating PWH (13±3 of a total of 16 points vs 11±3 and 10±3.3 points, respectively; p < 0.05) (Figure 1B), independently of hospital size or years of practice. As expected, more rheumatologists (90%) monitor inflammatory markers compared to cardiologists (71%) and doctors treating PWH (42%) (Table 1). Among the latter, most agreed that interleukin‐6 and D‐dimer should not be systematically monitored in the absence of validated cut‐off points of reference. While most (59%) include inflammation in their therapeutic recommendations, the most frequently reported options being high‐dose statins and insistence on a healthy lifestyle, we observed a negative correlation between the years of experience of doctors treating PWH and their concern about the clinical consequences of inflammation.

**Conclusions**: We found that knowledge of inflammation among doctors treating PWH in Spain is high. While the majority considers inflammation as a relevant clinical event, most agree that it should not be systematically monitored in the absence of validated markers. Our findings underline the need for applied clinical research on the monitoring and treatment of inflammation in PWH.

**Figure:**
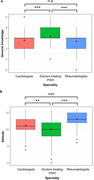
**Abstract P121 – Figure 1**. Knowledge and attitudes towards inflammation in PWH. (A) Score from non‐HIV‐related questions. (B) Total attitude results from questionnaire. n.s., not significant; PWH, people with HIV. ** p < 0.01; *** p < 0.001.

**Table:** 

**Abstract P121 – Table 1**. Practices and recommendations on inflammation.					
		Cardiologists (n = 135)	Doctors treating PWH (n = 135)	Rheumatologists (n = 135)	Overall (N = 405)
Do you evaluate inflammatory markers?	No	39 (28.9%)	78 (57.8%)	13 (9.6%)	130 (32.1%)
	Yes	96 (71.1%)	57 (42.2%)	122 (90.4%)	275 (67.9%)
Do you undertake any therapeutic intervention to reduce inflammation?	No	63 (46.7%)	56 (41.5%)	4 (3%)	123 (30.4%)
	Yes	72 (53.3%)	79 (58.5%)	131 (97%)	282 (69.6%)

**References**

1. Deeks SG, Tracy R, Douek DC. Systemic effects of inflammation on health during chronic HIV infection. Immunity. 2013;39:633‐45.

2. Hsu DC, Sereti I. Serious non‐AIDS events: therapeutic targets of immune activation and chronic inflammation in HIV infection. Drugs. 2016;76:533‐49.

3. Sieg SF, Shive CL, Panigrahi S, Freeman ML. Probing the interface of HIV and inflammaging. Curr HIV/AIDS Rep. 2021;18:198‐210.

4. Liberale L, Montecucco F, Tardif JC, Libby P, Camici GG. Inflamm‐ageing: the role of inflammation in age‐dependent cardiovascular disease. Eur Heart J. 2020;41:2974‐82.

5. Marcus JL, Leyden WA, Alexeeff SE, Anderson AN, Hechter RC, Hu H, et al. Comparison of overall and comorbidity‐free life expectancy between insured adults with and without HIV infection, 2000‐2016. JAMA Netw Open. 2020;3:e207954.

#### P122 A retrospective, multicentre study on the efficacy, durability, and tolerability of bictegravir/emtricitabine/tenofovir alafenamide (BIC/FTC/TAF) for the treatment of HIV in a real‐world setting in Belgium

R Nasreddine ^1^, E Florence^2^, J Yombi^3^, S Henrard^4^, G Darcis^5^, J Van Praet^6^, L Vandekerckhove^7^, S Allard^8^, R Demeester^9^, P Messiaen^10^, N Ausselet^11^, M Delforge^1^, S De Wit^1^

^1^Division of Infectious Diseases, Saint‐Pierre University Hospital, Brussels, Belgium; ^2^Department of Clinical Sciences, Institute of Tropical Medicine, Antwerp, Belgium; ^3^Division of Infectious Diseases, Cliniques Universitaires Saint‐Luc, Brussels, Belgium; ^4^Department of Internal Medicine, University Clinics of Brussels ‐ Erasme Hospital, Brussels, Belgium; ^5^Department of Infectious Diseases and General Internal Medicine, Liège University Hospital, Liège, Belgium; ^6^Department of Nephrology and Infectious Diseases, AZ Sint‐Jan Brugge‐Oostende, Brugge, Belgium; ^7^Department of Internal Medicine, Ghent University Hospital, Ghent, Belgium; ^8^Department of Internal Medicine and Infectious Diseases, Universitair Ziekenhuis Brussel, Brussels, Belgium; ^9^Department of Internal Medicine, University Hospital of Charleroi, Lodelinsart, Belgium; ^10^Department of Infectious Diseases and Immunity, Jessa Hospital, Hasselt, Belgium; ^11^Department of Infectious Diseases, UCL University Hospital Namur‐Godinne, Yvoir, Belgium

Several RCTs demonstrated BIC/FTC/TAF to be a first‐line option for treatment‐naïve (TN) and ‐experienced (TE) PLWHIV. Real‐world studies provide complementary data to RCTs ensuring those results can be generalised to broader populations seen in daily practice. The aim of this study was to describe the Belgian HIV population treated with BIC/FTC/TAF and to evaluate its efficacy, durability, and tolerability in a real‐world setting. Retrospective, multicentre study involving adult TN and TE PLWHIV on BIC/FTC/TAF between 1 January 2019 and 30 September 2020. Primary endpoint was virological suppression rate (plasma HIV‐1 viral load [VL] <50 copies/mL; on‐treatment analysis). Main secondary endpoints included loss of virological suppression (LVS; two consecutive HIV‐1 VLs of >200 copies/mL after being virologically suppressed) and analysis of resistance‐associated mutations (RAMs) at time of LVS; rate and reasons for treatment discontinuation; and on‐treatment weight gain, including patients reporting a >10% weight gain. Regression analysis examined associations between baseline variables and >10% weight gain. Overall, 2001 patients were included. Baseline characteristics included age ≥50 (40.8%), women (35.1%), black sub‐Saharan African (SSA; 32.3%), TN (20.4%), CD4+ count <500 cells/μL (37.3%), and virologically non‐suppressed (31.9%). Through 48 weeks, overall rate of virological suppression was 93.5% with similar results in the following sub‐groups: age ≥50 (92.7%), women (92.8%), SSA (91%), TN (94%), TE (93.2%), baseline CD4+ count <500 cells/μL (90.7%), and non‐suppressed at baseline (86.6%). LVS was observed in 0.7% (n = 14) of patients with two patients having RAMs to components of the regimen at time of LVS (Table 1). Of the 131 (6.5%) treatment discontinuations, most common cause was an adverse event (2.4%) with the most frequent being CNS toxicity (0.4%). Median (IQR) weight gain at week 48 was 2 kg (‐1 to 5) and >10% weight increase was observed in 11.6% of patients. TDF‐based regimen prior to BIC/FTC/TAF initiation (OR 2.29; 95% CI 1.31 to 4, p = 0.006) and baseline CD4+ count <350 cells/μL (OR 6.12; 95% CI 3.48 to 10.77, p < 0.001) were associated with >10% weight gain. In this large real‐world cohort, treatment with BIC/FTC/TAF was effective and well tolerated in a diverse set of patients.

**Table:** 

**Abstract P122 – Table 1**. Characteristics of the 14 participants with loss of virological suppression by week 48.									
P	Age	Gender/ethnicity	cART directly prior to baseline	RAMs prior to baseline	VL at baseline (copies/mL)	Number of viral blips prior to LVS (copies/mL)	Time to LVS (weeks)	VL at time of LVS (copies/mL)	RAMs at LVS
1	51	M/C	DRV/c/FTC/TAF	NDA	101	Once; 168	36	218	NDA
2	34	F/SSA	FTC/TAF + NVP	NDA	<50	None	48	397	NRTI: 67N, 70R, 184V
3	27	M/SSA	DTG/ABC/3TC	NDA	15 300	None	48	1188	None
4	22	M/SSA	Naïve	None	<50	None	48	588 000	NDA
5	33	F/SSA	DRV/c/FTC/TAF	None	1080	None	48	21 000	NDA
6	46	F/C	DTG/ABC/3TC	NDA	12 600	None	16	418	None
7	45	M/SSA	DRV/c/FTC/TAF	None	<50	None	27	385	NDA
8	55	F/SSA	ABC/3TC + DRV/r	NRTI: 184V INSTI: 66A, 92G	<50	None	32	578	NDA
9	51	M/SSA	Naïve	None	5152	None	17	2154	None
10	55	M/SSA	DTG + FTC/TAF	None	<50	Once; 77	37	457	None
11	31	M/C	Naïve	None	30 233	None	42	11 130	NDA
12	52	M/C	DTG + FTC/TAF	None	<50	None	48	22 700	None
13	34	M/SSA	DRV/c/FTC/TAF	None	<50	None	48	89 200	None
14	42	M/SSA	Naïve	None	932	Once; 182	48	836	NRTI: 184V INSTI: 263K/R

ABC/3TC, abacavir/lamivudine; C, Caucasian; cART, combined antiretroviral therapy; DRV/c, darunavir/cobicistat; DRV/r, darunavir/ritonavir; DTG, dolutegravir; F, female; FTC/TAF, emtricitabine/tenofovir alafenamide; INSTI, integrase strand transfer inhibitor; LVS, loss of virological suppression; M, male; NDA, no data available; NRTI, nucleoside/nucleotide reverse transcriptase inhibitor; NVP, nevirapine; P, patient; RAMs, resistance‐associated mutations; SSA, black sub‐Saharan African; VL, viral load.

#### P123 Perceptions of cabotegravir and rilpivirine long‐acting (CAB+RPV LA) from patients in the CAB+RPV Implementation Study in European Locations (CARISEL)

T Lutz^1^, E Jeanmaire^2^, J Portilla^3^, J Scherzer^4^, R Trehan^5^, M Pascual‐Bernáldez^6^, R DeMoor^7^, M Ait‐Khaled^8^, M Hadi^9^, S Anand^9^, E Low^9^, M Czarnogorski^10^, C Gutner ^11^

^1^Infektiologie, Infektiologikum Frankfurt, Frankfurt, Germany; ^2^Service de Maladies Infectieuses et Tropicales, Hôpital Brabois, Le Centre Hospitalier Régional Universitaire de Nancy, Vandoeuvre‐les‐Nancy, France; ^3^Departamento de Medicina Clínica, Hospital General Universitario de Alicante, Alicante, Spain; ^4^Global Health Outcomes, ViiV Healthcare GmbH, Munich, Germany; ^5^Innovation and Implementation Science, ViiV Healthcare Ltd, Brentford, UK; ^6^Clinical Operations, ViiV Healthcare Ltd, Madrid, Spain; ^7^R&D Biostatistics, GlaxoSmithKline, Collegeville, PA, USA; ^8^Clinical Sciences, R&D, ViiV Healthcare, Brentford, UK; ^9^Patient‐Centered Research, Evidera, London, UK; ^10^Global Medical Sciences, ViiV Healthcare, Durham, NC, USA; ^11^Global Medical Sciences, Innovation and Implementation Science, ViiV Healthcare Ltd, Durham, NC, USA

**Background**: Cabotegravir (CAB) + rilpivirine (RPV) long‐acting (LA) administered Q2M for HIV‐1 treatment offers a less frequent dosing alternative to daily oral pills. CARISEL, an implementation–effectiveness study, examines patient study participant (PSP) perspectives on CAB+RPV LA injections across HIV centres in Europe.

**Materials and methods**: PSPs completed acceptability, appropriateness, and feasibility questionnaires before their 1st (month [M] 1), 3rd (M4), and 7th (M12) injections. PSPs answered questions about facilitators/barriers to treatment, including stigma, pain/discomfort, and treatment preference.

**Table:** 

**Abstract P123 – Table 1**. Participant demographics.	
	Safety (n = 430)
Age, years	
Mean (SD)	44.2 (10.1)
Median (range)	44.0 (22 to 76)
≥50 years, n (%)	129 (30)
Gender (self‐reported), n (%)	
Male	315 (73)
Female	115 (27)
Race background, n (%)	
White	336 (78)
Non‐White	94 (22)
Ethnic background, n (%)	
Hispanic or Latinx	32 (7)
Not Hispanic or Latinx	398 (93)
Country, n (%)	
Belgium	71 (17)
France	171 (40)
Germany	54 (13)
The Netherlands	38 (9)
Spain	96 (22)

SD, standard deviation.

**Figure:**
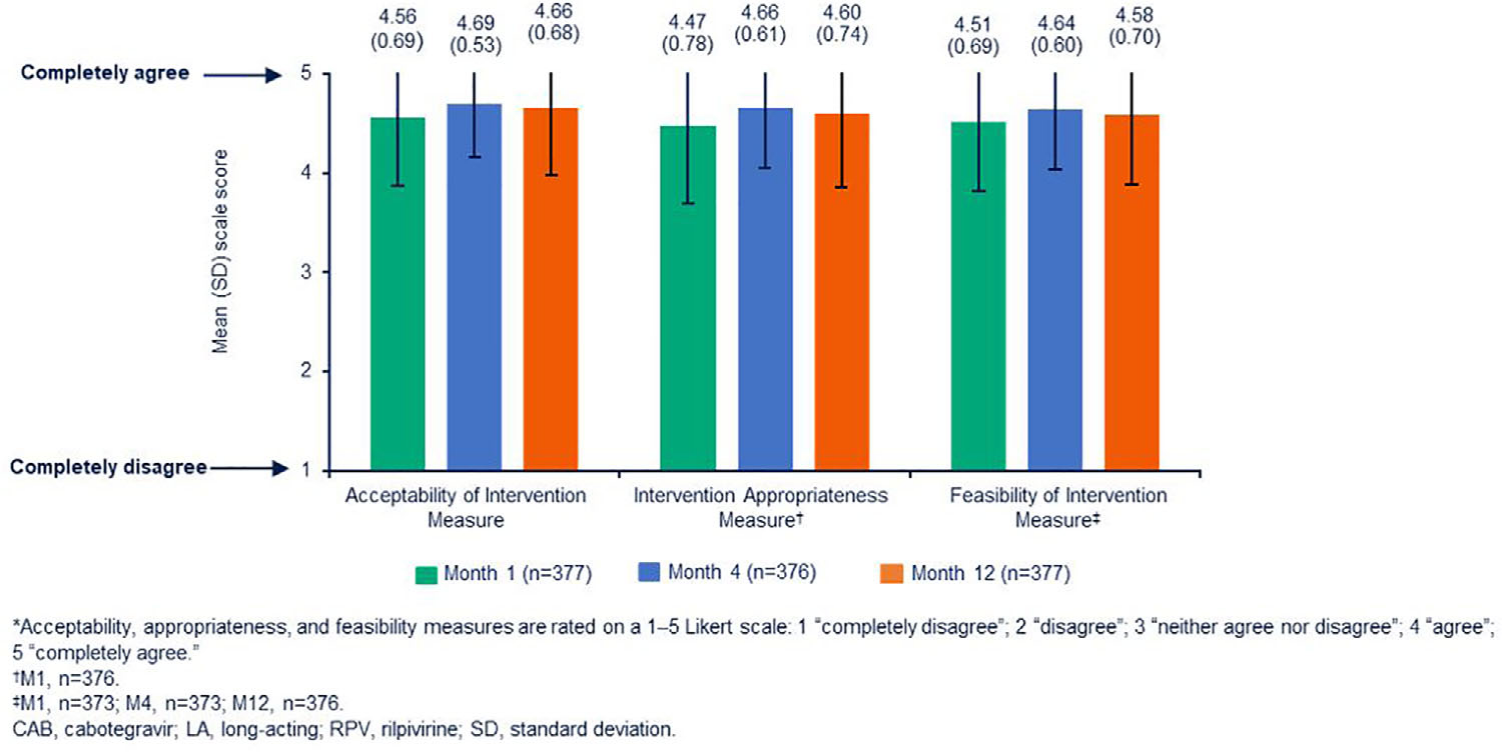
**Abstract P123 – Figure 1**. Acceptability, appropriateness, and feasibility^*^ of CAB+RPV LA over time.

**Results**: Three hundred and seventy‐nine of 437 (87%) PSPs completed questionnaires through M1 to M12. PSPs had a mean age of 45.1 years, 22% were non‐White, and 27% were women (self‐reported) (Table 1). Across time points, PSPs found CAB+RPV LA highly acceptable, appropriate, and feasible (mean scores ≥4.47; Figure 1). At M12, most PSPs (91%, n = 346/379) reported feeling 'very' or 'extremely positive' about CAB+RPV LA treatment. At M12, 78% (n = 290/374) waited ≤20 minutes in the exam room, 36% (n = 132/367) waited ≤40 minutes in clinic, and 26% (n = 97/367) waited ≤1 hour for injection visits. Seventy‐eight percent (n =291/374) found the time spent in clinic 'very' or 'extremely acceptable', and 85% (n = 317/374) found attending appointments Q2M 'very' or 'extremely acceptable'. PSPs primarily received information from their medical provider (77%, n = 290/379), followed by written material (24%, n = 90/379). Ninety‐nine percent (n = 275/277) preferred CAB+RPV LA to daily oral therapy, and 81% (n = 306/374) 'agreed' or 'completely agreed' that CAB+RPV LA was less stigmatising than oral medication. Fifty‐six percent (n = 212/379) noted that injection pain/soreness was the most difficult part of treatment; 31% (n = 119/379) reported 'nothing makes this treatment difficult'. At M12, the mean pain score during the most recent injection was 3 on a 0 ('no pain') to 10 ('extreme pain') scale. Ninety‐five percent (n = 358/375) of PSPs 'agreed' or 'completely agreed' with recommending CAB+RPV LA to other people living with HIV (PLWHIV).

**Conclusions**: PSPs found CAB+RPV LA to be an acceptable, appropriate, and feasible treatment option. PSPs most often received treatment information from their medical provider and reported positive implementation experiences and low pain from CAB+RPV LA injections. These factors may contribute to preference for CAB+RPV LA over oral therapy, perceptions of reduced stigma, and recommendation of CAB+RPV LA to other PLWHIV.

#### P124 Digital emergency healthcare seeking behaviour of Ukrainian refugees living with HIV

C Jordans, M Vasylyev, C Rokx

Infectious Diseases, Erasmus Medical Center, Rotterdam, Netherlands

**Background**: Ukraine is one of the European countries that is hardest‐hit by HIV which necessitates widespread and well‐accessible care for people living with HIV (PLWHIV) to stop the epidemic. The Russian invasion in February 2022 hit PLWHIV hard with HIV care services being abruptly interrupted. Several professional HIV networks within Europe started remote digital counselling platforms for PLWHIV from Ukraine who fled the country as part of emergency HIV care strategies. An overview of digital healthcare seeking behaviour can help tailoring these platforms to the needs of PLWHIV.

**Table:** 

**Abstract P124 – Table 1**. Number of people seeking contact and the country where they needed help over time.					
	Number of people seeking contact	Eastern European country, n (%)	Western European country, n (%)	Other country, n (%)	Unknown country, n (%)
Week 1 to 5	21	13 (62)	6 (29)	−	2 (9)
Week 6 to 10	44	23 (52)	14 (32)	−	7 (16)
Week 11 to 15	17	6 (35)	6 (35)	2 (12)	3 (18)

**Materials and methods**: 28 February 2022, we launched the #awarehivUkraine project to raise awareness and support for PLWHIV in Ukraine. The project and website (www.awarehiv.com/ukraine) were widely distributed across European professional HIV networks and HIV advocacy groups. People could contact HIV physicians directly for help by filling in a digital form. Demographical data and healthcare information provided were anonymously analysed. Main endpoints were: evolution of digital healthcare seeking behaviour, including the number of people seeking contact, country where they needed help, and the needs asked for.

**Results**: Since its start, the website has been visited 1197 times. Nineteen percent found the website by social media and 15% by search engines. Until 9 August, we were contacted 73 times (range 1 to 9/week, peak mid/end June) with questions for a total of 82 PLWHIV. Only three individuals could not be answered due to incorrect or missing contact details. Most were women (53/82), 23 were men and six did not disclose their sex. All but four contact topics were on antiretroviral therapy related questions (69/73). Over half (42) sought contact from Eastern Europe, most from Poland (28/82). Germany hosted most people who sought contact from Western Europe (10/82) (Figure 1). Over time, a decrease was seen in the number of people contacting from Eastern Europe along with an increase from Western Europe (Table 1).

**Figure:**
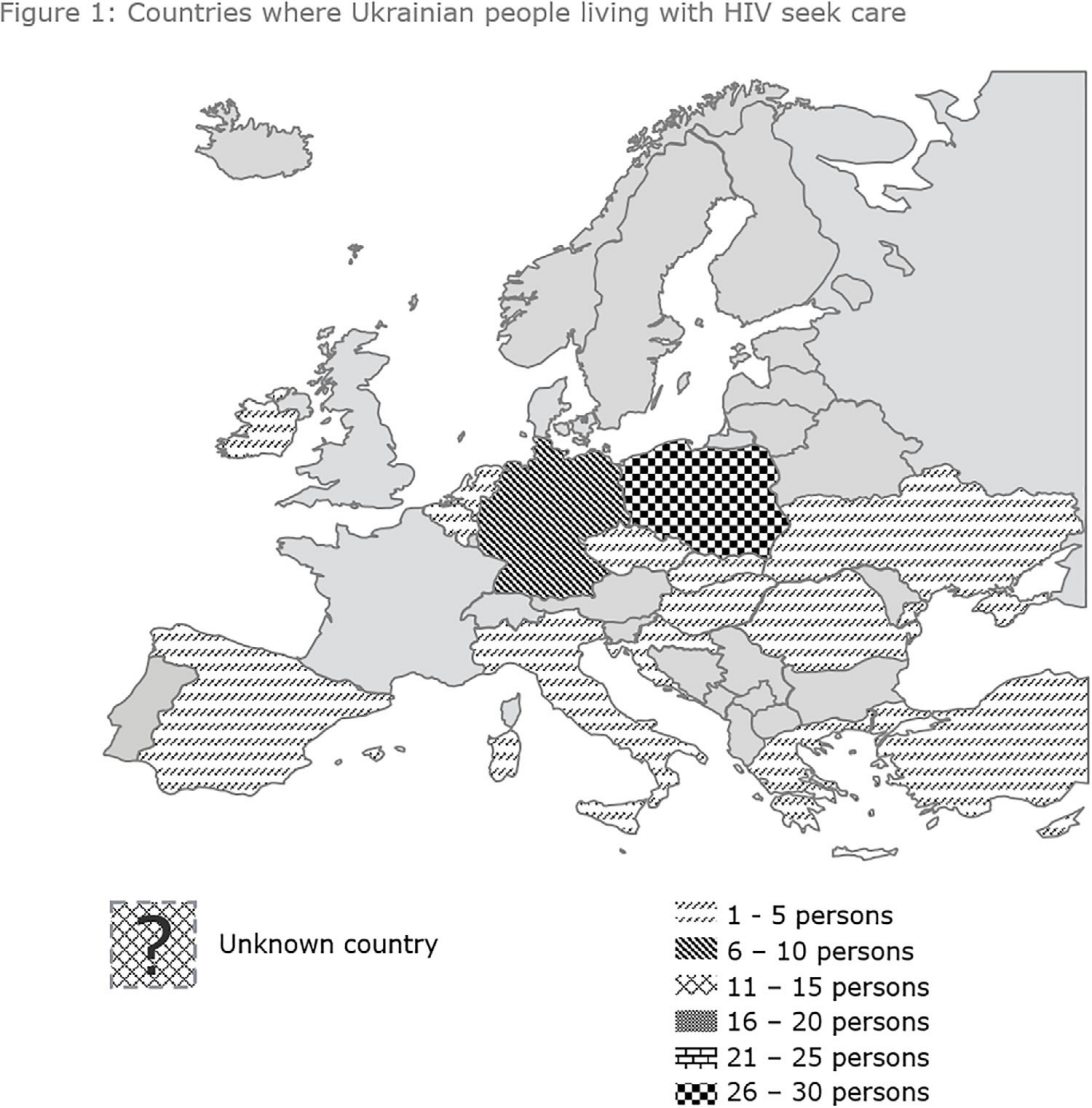
**Abstract P124 – Figure 1**. Countries where Ukrainian people living with HIV seek care.

**Conclusion**: HIV management related digital health consultation by healthcare professionals as a consequence of the war in Ukraine come from across Europe and is mostly on antiretroviral therapy. This type of healthcare seems feasible within professional HIV networks and can be a useful aid for PLWHIV and HIV care providers wanting to provide emergency HIV care.

#### P125 The application of HIV‐1 proviral DNA in patients with low‐level viraemia under antiretroviral therapy

S Lv ^1^, R Bai^1^, M Dai^2^, W Hua^1^, R Xin^2^, L Dai^1^

^1^No. 8, West Toutiao, outside You'anmen, Fengtai District, Beijing You'an Hospital Affiliated to Capital Medical University, Beijing, China; ^2^No. 16, Hepingli Middle Street, Dongcheng District, Institute for STD/AIDS Prevention and Treatment, Beijing Center for Disease Prevention and Control, Beijing, China

**Background**: One possible approach to characterise drug resistance of low‐level viraemia (LLV) patients is to sequence HIV proviral DNA. This study analysed the drug resistance characteristics in DNA of HIV/AIDS patients who developed LLV after antiretroviral therapy (ART) in a hospital in Beijing and evaluated its reliability compared to RNA genotypic resistance test (GRT).

**Figure:**
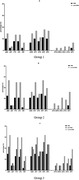
**Abstract P125 – Figure 1**. Comparison of percentage of resistance to individual ARVs between HIV‐1 RNA versus HIV‐1 DNA genotypes.

**Table:** 

**Abstract P125 – Table 1**. Patient characteristics and clinical features. Descriptive statistics are presented as median (IQR) and for categorical variables, as counts and proportions.	
Characteristics	Total (n = 150)
Sex	
Female	8 (5.3)
Male	142 (94.7)
Age	
Year, median	37 (32 to 36)
HIV transmission route	
MSM	116 (77.3)
Heterosexual	15 (10.0)
IVDU	4 (2.7)
NA	15 (10.0)
HIV‐RNA at baseline (copies/mL)	
50 to 200	118 (78.7)
201 to 400	16 (10.7)
401 to 999	16 (10.7)
Virological failure has occurred	
Yes	22 (14.7)
No	128 (85.3)
CD4 cell count at baseline (cells/μL)	488.19 (289.76 to 699.86)
Time since ART initiation (years)	3.12 (1.10 to 5.23)
Number of ARVs since ART initiation	4 (3 to 5)
Total known number of RNA genotypes	1 (1 to 2)
Duration of follow‐up (months)	5.13 (2.97 to 8.12)

IVDU, intravenous drug users; NA, not available.

**Materials and methods**: Peripheral venous blood was collected from HIV‐1 infected patients who had been routinely receiving ART for ≥6 months at the STD/AIDS clinic of Beijing Youan Hospital between January 2020 and September 2021 and who developed LLV. HIV‐1 DNA and RNA were extracted from concentrated white blood cells and plasma respectively, and the *pol* gene region of HIV‐1 virus was amplified. Combined with past RNA genotype, DNA and RNA genotypes were categorised into three groups: DNA genotypes compared with past RNA genotypes (group 1); comparing with RNA genotypes on the same day (group 2); comparing with past RNA genotypes and RNA genotypes on the same day (group 3).

**Results**: A total of 154 plasma samples with a viral load of 50 to 999 copies/mL were collected from 150 patients (Table 1), and 120 sequences were successfully amplified from 108 patients (77.9% amplification success rate). The resistance mutations were identified in 32 patients, with an overall resistance mutation rate of 29.6% (32/108) among which non‐nucleoside reverse transcriptase inhibitors (NNRTIs)‐associated resistance mutations predominated, accounting for 24.1% (26/108), followed by nucleoside reverse transcriptase inhibitors (NRTIs)‐associated mutations and protease inhibitors (PIs)‐associated mutations, accounting for 10.2% (11/108) and 5.6% (6/108) respectively. Of the 56, 89 and 47 patients included in groups 1, 2 and 3, comparing with RNA genotypes, the concordance rate of DNA drug resistance mutations (DRMs) were 73.0% (65/89), 75.0% (42/56) and 66.0% (31/47) respectively. The DRMs information in DNA genotypes was lost to varying degrees in all three groups and can be obviously examined within all classes of antiretrovirals (ARVs), especially in PIs (Figure 1).

**Conclusions**: Among LLV patients in Beijing, the drug resistance mutation rate is relatively more common with NNRTIs‐related resistance predominating. In the case of LLV, DNA GRT can provide certain resistance information, but the DRMs information in DNA genotypes may still be lost. The drug resistance of LLV patients should be combined with RNA GRT and past medical history for a comprehensive evaluation.

#### P126 Real‐world utilisation of doravirine among people living with HIV in England (DRIVE‐REAL)

C O'Halloran ^1^, Y Gilleece^2^, I Williams^3^, S Leung^4^, V Canuto^4^, C McAlpine^3^, S Ross^2^, C Norcross^2^, S Gaffney^1^, N Siani^1^, W Hickey^1^, A Moore^5^, O Rajkovic‐Hooley^5^, A Milinkovic^4^

^1^Medical Affairs (HIV), Merck Sharp & Dohme, London, UK; ^2^HIV and Sexual Health, Brighton & Sussex Medical School and University Hospitals Sussex NHS Trust, Brighton, UK; ^3^University College London & Mortimer Market Centre, Centre for Clinical Research in Infection and Sexual Health, London, UK; ^4^St Stephen's Centre, Chelsea and Westminster Hospital NHS Foundation Trust, London, UK; ^5^Real World, Adelphi, Bollington, UK

**Background**: Doravirine is a next‐generation non‐nucleoside reverse transcriptase inhibitor (NNRTI) recommended for treatment of treatment‐naïve and virologically suppressed people living with HIV (PLWHIV). Phase II clinical trials have demonstrated favourable efficacy and safety among suppressed PLWHIV switching to Delstrigo (DOR/3TC/TDF) through 144 weeks and among treatment‐naïve patients starting Delstrigo and doravirine‐based regimens through 192 weeks. The DRIVE‐REAL study aimed to evaluate doravirine use among a real‐world cohort.

**Methods**: A retrospective, observational, multi‐centre chart review was conducted for 300 PLWHIV, aged ≥18 years, initiating a doravirine‐containing regimen from July 2019 until January 2021, at three England‐based HIV services. Data were extracted using an electronic case report form from patient records. Primary objectives were to describe baseline patient characteristics and HIV treatment history. Secondary objectives included laboratory parameters at baseline and 6 months, baseline genotypic antiretroviral treatment (ART) resistance, comorbidities and outcomes stratified by subpopulations of interest.

**Results**: At baseline: 83% of participants were male, 45% aged ≥50 years, 65% were of white ethnicity. Median time since HIV diagnosis was 11.73 years (IQR 11.05 years). The majority (96%) of participants were ART‐experienced, 87% had an undetectable HIV viral load (VL) (<50 copies/mL) and 15% had recorded resistance to any ART drug. Two‐thirds (66%) of participants had comorbidities (most commonly: depression (26%), hyperlipidaemia (19%), anxiety disorders (13%) and hypertension (12%)), 55% of participants were overweight/obese, 30% were active smokers and 12% reported substance abuse. At 6 months 6% had discontinued their doravirine regimen. There were 6‐month VL data for 266 participants, 95% (n = 253) of whom were undetectable. Of the 13 with detectable VL at 6 months, three were non‐compliant, three discontinued doravirine (reason not stated). Among those with a VL recorded at 6 months, we observed no differences in the proportion achieving undetectable VL among baseline subgroups including: those aged ≥50 years versus <50 years (97% vs 94%), those with/out comorbidities (96% vs 93%), those overweight/obese versus normal/underweight (92% vs 97%) and those with/out ART resistance (97% vs 95%).

**Conclusions**: These findings provide reassurance that doravirine phase III clinical trial results translate into a real‐world setting. Additionally, the high proportion of viral suppression observed among individuals of older age, comorbidities, high BMI and ART resistance is encouraging.

#### P127 Association between fentanyl use and virologic suppression in an HIV‐infected cohort of people who use/inject drugs (PWID): results from a bictegravir/emtricitabine/tenofovir alafenamide (B/F/TAF) switch study

B Conway ^1,2^, S Yi^1^, S Sharma^1^, R Yung^1^, D Truong^1^

^1^Vancouver Infectious Diseases Centre, Vancouver, Canada; ^2^Simon Fraser University, Burnaby, Canada

**Background**: Although multi‐tablet antiretroviral therapy regimens have proven to be quite effective over the past 25 years, lower pill burdens (especially single‐tablet regimens, or STRs) have been associated with greater virologic suppression and patient satisfaction. This is particularly true of regimens including newer un‐boosted integrase strand transfer inhibitors (INSTIs) such as bictegravir, with an increased barrier to resistance, better tolerability, and few concerning drug interactions. This may offer a particular benefit among PWID in whom a higher likelihood of adherence and toxicity‐related challenges leading to incomplete treatment responses.

**Methods**: We identified patients who were prescribed multi‐tablet antiretroviral therapy and had no prior history of on‐treatment virologic failure and episodes of transient viremia in the previous year. Participants included in the study were switched to the single tablet B/F/TAF regimen and followed for up to 72 weeks. The primary endpoint of this analysis was the rate of achievement and maintenance of virologic suppression and its correlates.

**Results**: Between 03/19 and 03/20, we enrolled 46 subjects; one died of unrelated causes and was excluded from analysis. For the study cohort (n = 45), we note: median age 45 (34 to 66) years, 40 (89%) males; 32 (71.1%) actively using drugs; 21 (47%) cocaine/19 (42%) crystal/14 (31%) fentanyl users; median baseline CD4 cell count 620 (range 26 to 1490) cells/μL. All subjects remained available for analysis to week 72, with no drug‐related or other treatment discontinuations. At weeks 48 and 72, 38 (84.4%) and 37 (82.2%) had HIV plasma viral load measures <200 copies/mL. In longitudinal follow‐up, all returned to maximal virologic suppression while remaining on B/F/TAF. Twelve of 14 (86%) active fentanyl users were suppressed throughout the 72 weeks. There were eight (18%) participants with at least one incidence of transient viremia by week 72 (291 to 23 100 copies/mL). Over 72 weeks of observation, the incidence of transient viremia of patients on B/F/TAF was 13.61 cases/100 person years, compared to 25.83 cases/100 person years in a similar period prior to initiation of B/F/TAF (47% reduction in event rate).

**Conclusion**: Among marginalized HIV‐infected individuals experiencing transient viremia, a switch in antiretroviral therapy to B/F/TAF is safe, effective and is associated with a reduction in episodes of transient viremia.

#### P128 HIV testing in an infectious diseases unit: can we follow the guidelines?

L Buchanan, D Bell, S Debono

Infectious Diseases, Queen Elizabeth University Hospital, Glasgow, UK

**Background**: Up to December 2019, 5917 persons were diagnosed with HIV in Scotland, with almost one‐third resident in Glasgow. An estimated 505 patients still live undiagnosed across the country [1]. Diagnosis and treatment of HIV is critically important, and BHIVA/BASHH/BIA updated their HIV testing guideline in 2020 [2]. In addition, the Infectious Diseases (ID) department at the Queen Elizabeth University Hospital (QEUH) in Glasgow follows a local policy to HIV test all patients aged 16 to 65.   The aim of this project was to assess rates of HIV testing within the ID department of the QEUH compared to these guidelines. Testing rates were re‐assessed following a series of educational interventions to improve compliance with these guidelines.

**Materials and methods**: All patients discharged over a 2‐week period were identified. The local and national HIV guidelines were used to determine whether HIV testing was indicated for each patient. A total of three interventions were then introduced and data collected over subsequent 2‐week periods to assess the impact on testing. Interventions included a ward‐based poster, testing criteria information sheet given to medical staff, and a presentation of findings to doctors working in the ID unit.

**Results**: The percentage of patients that subsequently underwent appropriate testing increased following each intervention. Local policy testing improved from 25.7% (baseline), 36.1% (cycle 1), 42.9% (cycle 2), 45.5% (cycle 3); national guidance increased from 26.3% (baseline), 41.7% (cycle 1), 58.3% (cycle 2), to 61.5% (cycle 3). The most common indication to test was age. Other common indications included multiple sclerosis‐like symptoms, community‐acquired pneumonia and being known to addiction services. The improvement in testing rates noted was seen across a range of indicator conditions and patient groups.

**Conclusions**: Testing for HIV increased following each intervention, highlighting the effectiveness of continued education in improving testing for HIV. HIV testing according to national guidelines was achieved in a maximum of 61.5% of patients after multiple interventions. The results suggest that there remains a lack of knowledge and unrecognised barriers to HIV testing by healthcare professionals. Maintaining high levels of HIV testing is critical and can be improved by increasing awareness.

**References**

1. Health Protection Scotland. HIV in Scotland: Update to 31 December 2019 [Internet]. 23 June 2020 [cited 2022 Jun 30]. Available from: https://www.hps.scot.nhs.uk/publications/hps‐weekly‐report/volume‐54/issue‐25/hiv‐infection‐in‐scotland‐summary‐report‐to‐31‐december‐2019/.

2. Palfreeman A, Sullivan A, Rayment M, Waters L, Buckley A, Burns F, et al. British HIV Association/British Association for Sexual Health and HIV/British Infection Association adult HIV testing guidelines 2020. HIV Med. 2020;21(suppl 6):1‐26.

#### P129 Real‐life monocentric Biktarvy cohort from Perugia

S Benedetti, D Altobelli, G De Socio, A Lanzi, G Gamboni, D Francisci

Infectious Disease, Santa Maria della Misericordia, Perugia, Italy

**Background**: The aim of this study was to explore a real‐life cohort of people living with HIV (PLWHIV) in therapy with BIC/TAF/FTC (Biktarvy) to evaluate effectiveness, safety and durability.

**Figure:**
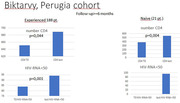
**Abstract P129 – Figure 1**. Effectiveness and improvement CD4 mean count in experienced and naive patients.

**Materials and methods**: Observational, retrospective cohort study that enrolled adult PWLHIV, treated with BIC/TAF/FTC. For each patient we collected information about: gender, age, nationality, CDC classification, route of transmission, comorbidities, CD4 cell count and HIV‐viraemia at T0 and at the last control, months on BIC/TAF/FTC, months of previous TARV, drug resistance test. In patients treated for more than 6 months, the analysis of therapy effectiveness was HIV‐RNA <50 copies/mL at last control. Durability was evaluated as discontinuation for any reason.

**Results**: We included 284 patients: 231 (81.3%) males, 218 (77%) Italians, with a mean age of 50.2 years (SD 12.2). The HIV stage was CDC C3 for 66 patients (23.2%), 31 (10.9%) were treatment naive, 253 (89.1%) were experienced. Two hundred and sixteen patients had comorbidities (76.1%). The nadir CD4 count mean was 265.2/mmc (SD 266.4). HIV genotype was available for 173 patients (60.9%) and 55 of them (19.4%) showed resistance for at least one antiretroviral drug, four (1.4%) presented M184V mutation. Median treatment time TAF/FTC/BIC was 17 months (IQR 8.7 to 23.6), treated for at least 6 months were 209. Adverse events were reported in five (1.8%) patients and in all cases the therapy was discontinued. The observation was interrupted in three cases for death (1.1%) and in 12 (4.2%) cases for lost at the follow‐up. In experienced patients after TAF/FTC/BIC switch mean CD4 count changed from 651/mmc to 683/mmc in 6 months follow‐up and also viral load <50 copies/mL passed from 83.9%  to 94.1%. All the patients with 184V mutation had undetectable viral load. Naive patients improve their CD4 mean count from 383/mmc to 545/mmc in a 6‐months follow‐up and viral load <50 copies/mL passed from 0% to 95% (Figure 1).

**Conclusions**: Our results show the high effectiveness both in naïve (95%) and experienced patients (94.1%) including a small number (four) of individuals treated with M184V, adverse effect with treatment discontinuation was observed in 1.8% of cases. The discontinuation for any cause was 7.4%.

#### P130 Clinical experience using dolutegravir + lamivudine in Glasgow

M Debono ^1^, K Ocker^1^, S Shepherd^2^, D Bell^1^

^1^Infectious Diseases, NHS Greater Glasgow and Clyde, Glasgow, UK; ^2^West of Scotland Specialist Virology Centre, NHS Greater Glasgow and Clyde, Glasgow, UK

**Background**: The two‐drug regimen (2DR) consisting of dolutegravir and lamivudine (DTG+LAM) has been shown to be a robust and efficacious treatment option in clinical trials. In Glasgow approximately 2000 patients attend the HIV service. We evaluated the clinical outcomes of those patients prescribed DTG+LAM in a real‐world setting.

**Materials and methods**: The HIV database was searched to identify patients prescribed DTG+LAM up to January 2022. Clinical information was obtained from their electronic records, including biochemistry and weight.

**Results**: Over 200 patients prescribed DTG+LAM were identified. One hundred and eighty‐three patients were treatment‐experienced and switched to DTG+LAM 2DR therapy. 96.7% were virologically suppressed at the time of switch. One hundred and sixty‐five patients had viral load (VL) checks at 3 to 6 months, with 97.5% showing virological suppression. Of the 98 patients who had a viral load at 7 to 12 months, 96 were suppressed (97.9%). Ninety‐nine percent of these patients remained on DTG+LAM 6 months post switch. No termination of therapy due to treatment failure was documented. Historic resistance test results were available on 51/183 of these patients; none had M184V. The most common reason for a switch to dolutegravir + lamivudine combination therapy was to reduce cardiovascular risk (40.4%) followed by switch to avoid current or future drug‐drug interactions (DDIs) (14.2%). Treatment simplification (13.1%) and adverse drug reactions to the current antiretroviral regimen (12%) were other popular reasons for switch. Eight treatment‐naïve patients were started DTG+LAM. Their pretreatment VLs ranged from <40 to 372 489 copies/mL with a median of 5194 copies/mL. By 6 months post treatment, all eight patients had VL <40 and this was sustained to 12 months in all five patients with follow‐up blood tests available. Ten patients with known multi‐class resistant HIV (five with M184V) received DTG+LAM in combination with at least one other HIV treatment. In 8/10 cases the additional medication was boosted darunavir. All 10 patients were undetectable during follow‐up.

**Conclusions**: Around 10% of the Glasgow HIV patient cohort are prescribed DTG+LAM, mostly as a 2DR switch therapy. Most switches were to reduce perceived cardiovascular risk or avoid DDIs. DTG+LAM appears well tolerated and effective in this real‐world setting.

### Opportunistic Infections

#### P131 Changes to LTBI screening in new diagnoses of HIV during the COVID‐19 pandemic

M Henderson, S Ojinnaka, L Garvey, N Mackie, B Mora‐Peris

HIV and Genitourinary Medicine, Imperial College Healthcare NHS Trust, London, UK

**Background**: The British HIV Association recommends screening for latent tuberculosis infection (LTBI) using an interferon gamma release assay (IGRA) in all persons with HIV (PWHIV) from medium‐ and high‐risk tuberculosis incidence countries, and low‐risk countries with additional tuberculosis risk factors [1]. The COVID‐19 pandemic, however, caused significant disruption to HIV services. We aimed to compare the percentage of newly diagnosed PWHIV screened for LTBI, prior to and during the first wave of the COVID‐19 pandemic.

**Materials and methods**: A retrospective case note review was performed for all new diagnoses of HIV attending a central London HIV service between 01/03/2019 and 30/03/2021. Data was collected ‘pre‐COVID‐19’ (01/03/2019 to 28/02/2020) and ‘during‐COVID‐19’ (01/03/2020 and 30/03/2021). Risk of tuberculosis was determined by Public Health England estimates of number and rate of tuberculosis cases by country and WHO region. PWHIV were risk categorised: medium‐ and high‐risk tuberculosis countries, and low‐risk countries with additional tuberculosis risk factors (Table 1). Those with active TB symptoms or previous TB were excluded from the analysis. Comparisons between time periods and risk‐factor groups were appropriately undertaken using Chi‐squared or Fisher's exact tests.

**Table:** 

**Abstract P131 – Table 1. **Number screened for LTBI based on risk category and year.				
	Total screened	Number screened in 2019‐2020, n=17	Number screened in 2020‐2021, n=7	p‐value
Total eligible for screening, n=34	24/34 (71%)	17/23 (77%)	7/11 (63%)	0.54
Medium‐high risk country, n=27	21/27 (78%)	14/17 (82%)	7/10 (70%)	0.64
Low risk country with additional TB risk factors, n=7	3/7 (43%)	3/6 (50%)	0/1 (0%)	1

**Results**: Eighty‐six new diagnoses of HIV were identified: 53/86 (62%) ‘pre’ and 33/86 (38%) ‘during’ COVID‐19. Median age was 36 years (range 17 to 72) and 67/86 (78%) were male. Absolute CD4 count was ≤200 cells/μL in 22/86 (26%) individuals. Thirty‐four of 86 (40%) were eligible for LTBI screening, of which 24/34 (71%) were screened over the 2‐year period, all using an IGRA. No statistically significant differences were seen in the number screened between time periods (Table 1). Overall, more patients who presented with a geographical tuberculosis risk were screened than those from low‐risk groups with additional tuberculosis risk factors (78% vs 43%; p = 0.16) (Table 1). Of those screened, 3/24 (12.5%) were positive, all from medium‐ and high‐risk countries.

**Conclusions**: A decline in the number of new diagnoses of HIV was seen during the first wave of the COVID‐19 pandemic without significant changes in LTBI screening patterns. Clinicians should be aware of the need to screen those with additional tuberculosis risk factors, regardless of geographical tuberculosis risk.

**Reference**

1. British HIV Association. BHIVA Guidelines for the Management of Tuberculosis in Adults Living With HIV 2018 (2021 Interim Update) [Internet]. 2021 [cited 2022 Jun 30]. Available from: https://www.bhiva.org/file/5c485f3dc7c17/BHIVA‐TB‐guidelines.pdf.

#### P132 Latent tuberculosis infection and associated risk factors among people living with HIV and HIV‐uninfected individuals in Lithuania

E Matulyte ^1^, Z Kancauskiene^2^, A Kausas^3^, J Urboniene^4^, V Lipnickiene^5^, I Razmiene^5^, J Kopeykiniene^6^, B Jonaityte^7^, A Eimutiene^2^, G Arstikaitiene^3^, A Lisinskaite^4^, T Gudaitis^8^, S Raudonis^8^, E Danila^9^, D Costagliola^10^, R Matulionyte^1^

^1^Faculty of Medicine, Institute of Clinical Medicine, Clinic of Infectious Diseases and Dermatovenerology, Vilnius University, Vilnius, Lithuania; ^2^Department of Infectious Diseases, University Hospital of Klaipeda, Klaipeda, Lithuania; ^3^Clinic of Conservative Medicine, Adult Infectious Diseases Unit, Republican Siauliai County Hospital, Siauliai, Lithuania; ^4^Centre of Infectious Diseases, Vilnius University Hospital Santaros Klinikos, Vilnius, Lithuania; ^5^Department of Clinical Trials, National Public Health Surveillance Laboratory, Vilnius, Lithuania; ^6^Department of Diagnostics, University Hospital of Klaipeda, Klaipeda, Lithuania; ^7^Centre of Pulmonology and Allergology, Vilnius University Hospital Santaros Klinikos, Vilnius, Lithuania; ^8^Faculty of Medicine, Vilnius University, Vilnius, Lithuania; ^9^Faculty of Medicine, Institute of Clinical Medicine, Clinic of Chest Diseases, Immunology, and Allergology, Vilnius University, Vilnius, Lithuania; ^10^INSERM, Institut Pierre Louis d'Épidémiologie de Santé Publique, Sorbonne Université, Paris, France

**Background**: People living with HIV (PLHIV) with latent tuberculosis infection (LTBI) are at increased risk for tuberculosis (TB) reactivation compared to HIV‐negative population. Lithuania belongs to the 18 high‐priority TB countries in the European region. Study aim was to compare the prevalence of LTBI and LTBI‐related risk factors between PLHIV and HIV‐negative population.

**Figure:**
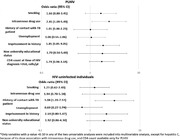
**Abstract P132 – Figure 1**. Multivariable analysis* of factors associated with LTBI among PLHIV and HIV‐uninfected individuals in Lithuania.

**Table:** 

**Abstract P132 – Table 1**. Sociodemographic and clinical characteristics of study participants (n = 833).									
Characteristic		PLHIV with LTBI (n = 70), n (%)	PLHIV without LTBI (n = 320), n (%)	Crude OR (95% CI)	p‐value	HIV‐uninfected individuals with LTBI (n = 49), n (%)	HIV‐uninfected individuals without LTBI (n = 394), n (%)	Crude OR (95% CI)	p‐value
Gender	Male	50 (71.43)	222 (69.38)	1.10 (0.62 to 1.95)	0.735	35 (71.43)	255 (64.72)	1.36 (0.71 to 2.62)	0.353
	Female	20 (28.57)	98 (30.63)			14 (28.57)	139 (35.28)		
Age	≤40 years	37 (52.86)	153 (47.81)	1.22 (0.73 to 2.05)	0.445	18 (36.73)	164 (41.62)	0.81 (0.44 to 1.51)	0.512
	>40 years	33 (47.14)	167 (52.19)			31 (63.27)	230 (58.38)		
Educational status	Not university	66 (94.29)	243 (75.94)	5.23 (1.85 to 14.81)	0.002	40 (81.63)	258 (65.48)	2.34 (1.10 to 4.97)	0.027
	University	4 (5.71)	77 (24.06)			9 (18.37)	136 (34.52)		
Unemployment	Yes	41 (58.57)	108 (33.75)	2.78 (1.64 to 4.71)	<0.001	7 (14.29)	54 (13.71)	1.05 (0.45 to 2.46)	0.912
	No	29 (41.43)	212 (66.25)			42 (85.71)	340 (86.29)		
History of contact with TB patient	Yes	11 (15.71)	44 (13.75)	1.17 (0.57 to 2.40)	0.669	8 (16.33)	27 (6.85)	2.65 (1.13 to 6.22)	0.025
	No, unknown	59 (84.29)	276 (86.25)			41 (83.67)	367 (93.15)		
Imprisonment in history	Yes	46 (65.71)	96 (30)	4.47 (2.58 to 7.74)	<0.001	12 (24.49)	31 (7.87)	3.80 (1.80 to 8.02)	<0.001
	No	24 (34.29)	224 (70)			37 (75.51)	363 (92.13)		
Hepatitis, yes vs no	Anti‐HCV positive (n = 534)	44 (75.86)	85 (34.84)	5.88 (3.05 to 11.34)	<0.001	20 (64.52)	88 (43.78)	2.33 (1.06 to 5.13)	0.035
	HBsAg positive (n = 487)	3 (5.17)	9 (3.72)	1.41 (0.37 to 5.39)	0.614	2 (10.53)	27 (16.07)	0.61 (0.13 to 2.81)	0.530
Smoking	Yes	58 (82.86)	186 (58.13)	3.48 (1.80 to 6.74)	<0.001	23 (46.94)	126 (31.98)	1.88 (1.03 to 3.43)	0.039
	No	12 (17.14)	134 (41.88)			26 (53.06)	268 (68.02)		
Intravenous drug use, ever vs never	Ever	50 (71.43)	104 (32.5)	5.19 (2.94 to 9.17)	<0.001	10 (20.41)	28 (7.11)	3.35 (1.52 to 7.41)	0.003
	Never	20 (28.57)	216 (67.5)			39 (79.59)	366 (92.89)		
Alcohol abuse	Yes	6 (8.57)	25 (7.81)	1.11 (0.44 to 2.81)	0.832	3 (6.12)	15 (3.81)	1.65 (0.46 to 5.91)	0.443
	No	64 (91.43)	295 (92.19)			46 (93.88)	379 (96.19)		
CD4 count at time of HIV diagnosis, ≤350 vs >350 (cells/L) (n = 364)	≤350	26 (41.27)	182 (60.26)						
	>350	37 (58.73)	120 (39.74)	2.16 (1.24 to 3.75)	0.006				
CD4 count at time of IGRA test, ≤350 vs >350 (cells/L) (n = 383)	≤350	18 (25.71)	115 (36.62)						
	>350	52 (74.29)	199 (63.38)	1.67 (0.93 to 2.99)	0.085				
HIV RNA at time of HIV diagnosis, copies/mL (n = 336)	<200	8 (13.56)	24 (8.63)						
	≥200	51 (86.44)	254 (91.37)	0.60 (0.26 to 1.42)	0.245				
HIV RNA at time of IGRA test, copies/mL (n = 378)	<200	41 (58.57)	188 (60.84)						
	≥200	29 (41.43)	121 (39.16)	110 (0.65 to 1.86)	0.726				

**Methods**: Cross‐sectional study was conducted in three Lithuanian infectious diseases centres 08/2018 to 05/2022. Interferon‐gamma‐release‐assay (IGRA) and tuberculin‐skin‐test (TST) in Vilnius, IGRA only in Siauliai and Klaipeda. Cohen's kappa coefficient was used to determine IGRA and TST agreement. A structured questionnaire was completed by study participants. LTBI‐related risk factors were identified by multivariable logistic regression.

**Results**: Three hundred and ninety PLHIV and 443 HIV‐uninfected individuals enrolled, median age 41 (IQR 36 to 48) and 43 (IQR 36 to 50), 69.7% and 65.5% male, respectively (Table 1). In the PLHIV group, median CD4 count was 475 (IQR 268.5 to 672.5) cells/μL, median HIV‐RNA – 39 (IQR 0 to 9750) copies/mL, 67.4% had started ART at time of LTBI test. The prevalence of LTBI defined by positive IGRA among PLHIV was higher compared to HIV‐negative population (17.9% vs 11.1%; OR 1.62; 95% CI 1.16 to 2.28; p = 0.005). Concordance between IGRA and TST was fair: kappa = 0.2 (95% CI 0.07 to 0.40). In univariable analyses, the factors associated with LTBI in PLHIV were non‐university educational status, intravenous drug use (IVDU), co‐infection with hepatitis C virus (HCV), imprisonment, smoking, unemployment and CD4 count >350 cells/mL at time of HIV diagnosis; and imprisonment, IVDU, co‐infection with HCV, a history of contact with a TB patient, non‐university educational status and smoking – in HIV‐uninfected individuals. In multivariable analyses, association with IVDU (ORa 2.45; 95% CI 1.09 to 5.49, p = 0.03) in PLHIV and a history of contact with a TB patient (ORa 3.08; 95% CI 1.26 to 7.53; p = 0.01) in HIV‐uninfected individuals were the only significant associations with LTBI evidenced (Figure 1).

**Conclusions**: The prevalence of LTBI among PLHIV in Lithuania is higher compared to HIV‐uninfected population and European average. The association with IVDU in PLHIV emphasises the need for integrated HIV/HCV/TB and substance abuse treatment to provide patient‐centred care.

#### P133 Pharmacovigilance and drug safety trainings to increase tuberculosis (TB) preventive treatment (TPT) for tuberculosis in Zimbabwe

J Jokwiro ^1^, N Kawaza^1^, K Sithole^1^, B Dube^1^, C Sandy^2^, M Ncube^2^, R Mubau^2^, C Gwanzura^2^, K Ndlovu^2^, A Nyambo^2^

^1^Tuberculosis Access, Clinton Health Access Initiative (CHAI), Harare, Zimbabwe; ^2^AIDS and Tuberculosis Unit, Ministry of Health and Child Care, Harare, Zimbabwe

**Background**: Zimbabwe's high tuberculosis (TB) ​​burden of 193 per 100 000 (16 019 notifications) is largely aggravated by a 54% co‐infection incidence of TB/HIV [1]. Since the introduction of isoniazid as a TB prevention therapy (TPT) regimen in 2012, sub‐optimal uptake has been reported [1]. In 2018, 27% newly enrolled people living with HIV (PLWHIV) and 37% eligible child contacts were initiated on TPT [1]. Undeveloped TPT pharmacovigilance frameworks contributed to suboptimal TPT uptake driven by inadequate pharmacovigilance reporting tools and health workers exhibiting limited knowledge on the reporting and management of TPT adverse events [2].

**Materials and methods**: Clinton Health Access Initiative (CHAI), in partnership with the government of Zimbabwe National TB Program and Medicines Control Authority of Zimbabwe, conducted TPT and 3HP pieces of training integrated with medicine safety monitoring and reporting pieces of training. These pieces of training targeted 10 TPT sites, four paediatric DTG, and 10 AHD therapeutics sites, training 414 healthcare workers [3]. Additionally, adverse event reporting forms were disseminated.

**Results**: 3HP enrolment improved from zero in 2019 to 49% in 2021, with over 15 000 patients enrolled on 3HP across 20 CHAI‐supported sites. Treatment completion increased exponentially from 26% in 2019 to 93% in 2021. Adverse event reporting in 3HP improved from sparse to solid documentation and systems, resulting in an incidence of 0.5 in 2021. Commonly reported adverse events included mild rash, vomiting and reddish body fluids which were self‐limiting and clients completed TPT after reassurance and counselling.

**Conclusions**: Drug safety monitoring is integral to CHAI's implementation approaches to help prevent and/or treat adverse events and improve patient care. Pharmacovigilance drug safety training, creation of robust drug safety systems, dissemination of adverse event forms ensured increased TPT intake and adverse event reporting. Thus, pharmacovigilance supported the smooth transition, adoption and uptake of new shorter TPT regimens in Zimbabwe.

**References**

1. World Health Organization. Global Tuberculosis Report [Internet]. 2021 [cited 2022 Aug 23]. Available from: https://www.who.int/publications/i/item/9789240037021.

2. Nyathi S, Dlodlo RA, Satyanarayana S, Takarinda KC, Tweya H, Hove S, et al. Isoniazid preventive therapy: uptake, incidence of tuberculosis and survival among people living with HIV in Bulawayo, Zimbabwe. PLoS One. 2019;14:e0223076.

3. Medicines Control Authority of Zimbabwe. Medicines Information Bulletin [Internet]. February 2022 [cited 2022 Aug 23]. Available from: https://www.mcaz.co.zw/bulletins/.

#### P134 Rapid efficiencies in policy adoption through policy influence plan and consultative engagements revolutionalise the TB preventive therapy (TPT) landscape in Zimbabwe

J Jokwiro ^1^, N Kawaza^1^, K Sithole^1^, B Dube^1^, C Sandy^2^, M Ncube^2^, C Gwanzura^2^, R Mubau^2^, A Nyambo^2^

^1^Blok 4, Arundel Office Park, Clinton Health Access Initiative (CHAI), Harare, Zimbabwe; ^2^AIDS and Tuberculosis Unit, Ministry of Health and Child Care, Harare, Zimbabwe

**Background**: In 2019, tuberculosis (TB) claimed the lives of 30% of people living with HIV (PLWHIV) worldwide [1]. A 54% TB/HIV co‐infection rate contributes to Zimbabwe's considerable TB burden with an incidence of 193 per 100 000 [1]. Since 2012, TPT uptake has been suboptimal at 39.1% in 2020. Similarly, in 20 select IMPAACT4TB sites, only 38% of PLWHIV initiating TPT and only 26% of those completed. Exclusion of shorter TPT regimens, health worker scepticism, fragmented TPT guidelines, and inadequate communication of guideline modifications are all problems associated with suboptimal TPT initiation [2].

**Materials and methods**: The Clinton Health Access Initiative and Ministry of Health harmonised catalytic interventions to necessitate a smooth TPT policy update through: LTBI stakeholder and resource mapping, integrated TB/HIV implementation, data‐driven and consultative TPT engagements, dissemination of evidence‐based TPT data guided by a robust policy influence plan, technical assistance on development of the LTBI addendum, training materials and reporting tools.

**Results**: Updated the TPT guidelines, Essential Drugs List for Zimbabwe (EDLIZ), LTBI communication strategy, job aides, treatment algorithms, training manuals, and reporting tools to include 3HP as the preferred TPT regimen. Improved TPT awareness among 820 healthcare workers and 85 community workers by December 2021. Resultantly, improving TPT documentation – 15, 092, completion – 93%, and adverse events – 0.5%, 100% access to updated guidelines by all public health facilities. The inclusion of 3HP in the EDLIZ enabled ring‐fencing resources through the Global Fund and PEPFAR towards 3HP procurement.

**Conclusions**: Efficiencies in evidence‐based policy adaptation based on global trends remains cornerstone for TPT programming and the adoption of novel TPT regimens. A robust policy influence plan provides the detailed roadmap for stakeholder mapping and consultative engagements that foster ownership, sustainability, and scalability. This rapid policy adoption culminated in over 15 000 ‐ 3HP initiations, sustained 3HP procurements and TPT surge plan to scale‐up TPT.

**References**

1. World Health Organization. Global tuberculosis report 2021. Geneva: World Health Organization; 2021.

2. Ministry of Health and Child Care. 2019 annual TB/HIV report. Harare: Ministry of Health and Child Care; 2020.

#### P135 Closing the science‐to‐service gap by increasing TB preventive therapy (TPT) uptake using the choice architecture tuberculosis preventive therapy (CAT) prescriptive stickers

J Jokwiro ^1^, N Kawaza^2^, K Sithole^2^, B Dube^2^, C Sandy^3^, M Ncube^4^, R Mubau^4^, C Gwanzura^4^, A Nyambo^4^, K Ndlovu^4^

^1^Blok 4, Arundel Office Park, Clinton Health Access Initiative (CHAI), Harare, Zimbabwe; ^2^TB Access, Clinton Health Access Initiative (CHAI), Harare, Zimbabwe; ^3^AIDS and TB Unit, Clinton Health Access Initiative (CHAI), Harare, Zimbabwe; ^4^AIDS and TB Unit, Ministry of Health and Child Care, Harare, Zimbabwe

**Background**: Tuberculosis (TB) accounts for 30% of deaths among people living with HIV (PLWHIV) worldwide in 2019 [1]. A 54% TB/HIV co‐infection rate contributes to Zimbabwe's considerable TB burden ‐ incidence of 193 per 100 000 [1]. In PLWHIV, TPT reduces TB incidence by 62% and death by 39% [2]. A global science‐to‐service gap of <20% of eligible PLWHIV starting TPT was reported [3]. Moreover, in Zimbabwe only 38% of PLWHIV were initiated among them 26% were completed in 2019 [4]. Health worker challenges contributing to the 38% science‐to‐service gap include inadequate understanding of the TPT benefits; high TPT prescriptive cognitive burden due to integrated service provision; TPT initiation scepticism and many reporting tools [4].

**Materials and methods**: The Clinton Health Access Initiative and the Ministry of Health and Child Care are implementing a two‐pronged choice architecture intervention study. The choice architecture TPT (CAT) prescriptive sticker intervention shifts clinician decision‐making from prescribing TPT to identifying people who should not receive TPT, making TPT delivery the default. The intervention included healthcare worker training, printing, and distribution of CAT prescription stickers. The intervention's purpose was to see if prescribing TPT using a 'default' CAT prescriptive strategy would dramatically increase TPT prescribing. Healthcare worker TPT prescriptive cognitive burden has been attributable to low TPT uptake; however, the 'default' CAT prescriptive strategy has the potential to dramatically increase TPT prescribing.

**Results**: Preliminary results reveal that the CAT prescriptive sticker is attributable to an increasing TPT initiation among newly PLWHIV clients from 52% to 61%. Intervention sites have reported a huge increase in Rujeko from 18% to 93%, Highfield 18% to 64%, Northern Suburbs from 0% to 100% as compared to control sites St Luke's from 30% to 33%, Beitbridge from 41% to 25%, Pumula 0% to 9%.

**Conclusions**: As a result of the CAT prescriptive sticker, TPT initiation among PLWHIV has increased from 52% to 61%. Other services including ART, cotrimoxazole, cervical cancer, and other drugs provided to PLWHIV are integrated into the CAT prescriptive sticker, ensuring that PLWHIV receives a quality, holistic and comprehensive package.

**References**

1. World Health Organization. Global tuberculosis report 2021. Geneva: World Health Organization; 2021.

2. Badje A, Moh R, Gabillard D, Guéhi C, Kabran M, Ntakpé J‐B, et al. Effect of isoniazid preventive therapy on risk of death in west African, HIV‐infected adults with high CD4 cell counts: long‐term follow‐up of the Temprano ANRS 12136 trial. Lancet Glob Health. 2017;5:e1080‐9.

3. World Health Organization. Global tuberculosis report 2019. Geneva: World Health Organization; 2020.

4. Ministry of Health and Child Care. 2019 annual TB/HIV report. Harare: Ministry of Health and Child Care; 2020.

#### P136 Effects of incentivizing TB contact investigation and linkage to treatments

B Dube ^1^, N Kawaza^1^, J Jokwiro^1^, K Sithole^1^, C Sandy^2^, M Ncube^2^, C Gwanzura^2^, R Mubau^2^, K Ndlovu^2^, N Siziba^2^

^1^TB Prevention, Clinton Health Access Initiative, Harare, Zimbabwe; ^2^NTP, Ministry of Health and Child Care, Harare, Zimbabwe

**Background**: Contacts of TB index cases have a high risk of contracting TB, making them an accessible group from which new cases can be quickly identified, treated, and TPT directed [1]. Zimbabwe has suboptimal contact investigation due to lack of human and material resources with an average of one contact per TB case, which is less that the MoHCC recommended four contacts per TB case [2].

**Materials and methods**: With support from UNITAID, CHAI in collaboration with MoHCC supported the initial scale up of 3HP. Environmental health technicians (EHTs) from one sentinel site, Highfields Polyclinic in Harare, were capacitated on TPT and provided with performance‐based incentives for TB contact tracing and TPT initiations among household contacts of all ages from January to June 2021.

**Results**: At baseline, about one contact per TB index case (34 per 22) was recorded, but this value quadrupled (99 per 25) during the intervention and dropped to one (14 per 19) afterward. Of the 34 contacts at baseline, 26% (9/34) were linked to TPT, with a 44% (4/9) completion rate. During the intervention, TPT initiations increased to 82% (81/99), with a 17% (14/81) completion rate. After the intervention 150% (21/14) initiations with a 205% (43/21) completion rate recorded, including a residual of contacts initiated during the intervention.

**Conclusions**: Investments in TPT contact tracing should include capacitation of contact tracers and household contacts on TB and TPT and enablers such as performance‐based incentives. Additionally, fostering seamless relationships between EHTs, community and facility‐based health care working will strengthen linkages to care, TPT adherence and positive treatment outcomes among identified contacts. This will prioritise contact tracing within the TB control programme and draw Zimbabwe closer to achieving the End TB Strategy goals.

**References**

1. Yuen CM, Amanullah F, Dharmadhikari A, Nardell EA, Seddon JA, Vasilyeva I, et al. Turning off the tap: stopping tuberculosis transmission through active case‐finding and prompt effective treatment. Lancet. 2015;386:2334‐43.

2. Zimbabwe Ministry of Health and Child Care. National Tuberculosis Program – Strategic Plan (2017‐2020); 45. [cited 2022 Aug 23]. Available from: https://depts.washington.edu/edgh/zw/hit/web/project-resources/TB-NSP.pdf.

#### P137 Tailored support visits to improve screening rates for cryptococcal meningitis and tuberculosis during early implementation of the advanced HIV disease package of care in Zimbabwe

G Saemisch^1^, T Chirindo ^2^, T Maparo^2^, C Giyava^2^, A Moore^2^, I Amamilo^2^, N Kawaza^2^

^1^Advanced HIV Disease, Clinton Health Access Initiative, Inc., Boston, MA, USA; ^2^Advanced HIV Disease, Clinton Health Access Initiative, Inc., Harare, Zimbabwe

**Background**: Zimbabwe has achieved one of the highest antiretroviral treatment coverage rates (95.8%) in Southern Africa. However, it is estimated that 35% of persons newly initiated on ART present with advanced HIV disease (AHD), and 50% of HIV/AIDS‐related deaths are due to cryptococcal meningitis (CM) and tuberculosis (TB). In 2021, the Zimbabwe Ministry of Health implemented the World Health Organization (WHO) recommended AHD package of care at 23 high‐volume sites. We documented the impact of tailored supportive visits as a tool to improve cryptococcal antigen (CrAg) and TB lipoarabinomannan (LAM) screening rates for CM and TB among patients with AHD during the early implementation of the WHO package of care in Zimbabwe.

**Methods**: In August 2021, AHD trainings were conducted at the 23 sites, and supportive visits were provided once per quarter to address implementation challenges. During supportive visits, primary outcome data on the proportion of AHD patients who were screened for CM and TB was collected from the improvised AHD register and electronic patient management system and inputted into a Microsoft Excel AHD data capture tool. Qualitative data on health care worker (HCW) awareness of AHD screening was gathered from HCW interviews using an open‐ended questionnaire and cross‐sectional survey. Following data collection and qualitative interviews, refresher trainings and job aides were provided to site staff to address any uncovered gaps in AHD screening performance and HCW awareness.

**Results**: At baseline, the 23 sites were not actively screening AHD patients for CM and TB. However, sites demonstrated quick improvement in CM and TB screening following each round of supportive supervision. Screening rates increased by an average of 14.5% for CM and 7% for TB per quarter from Q3 2021 to Q1 2022. As a result, 87% of AHD patients received a CrAg test and 90% received a TB‐LAM test at the 23 sites in Q1 2022 (Table 1).

**Table:** 

**Abstract P137 – Table 1**. Quarter‐over‐quarter screening improvements for CM and TB at 23 AHD sites in Zimbabwe.			
	Q3 2021	Q4 2021	Q1 2022
Percentage of PLWHIV with CD4 <200 who received a CrAg test during reporting period	58%	83%	87%
Percentage of PLWHIV with CD4 <200 who received a TB LAM test during reporting period	76%	83%	90%

**Conclusion**: Zimbabwe demonstrated the effectiveness of tailored support during early implementation of the AHD screening package of care. National HIV programmes can learn from Zimbabwe's experience when implementing AHD screening interventions.

#### P138 Utilizing electronic communications for clinical mentorship to improve optimal treatment rates for cryptococcal meningitis in Malawi

G Saemisch^1^, Y Gumulira ^2^, R Nyirendra^3^, B Wilson^3^, P Nyasulu^3^, D Telela^2^, E Matupa^2^, T Mwenifumbo^2^, A Moore^1^, I Amamilo^1^

^1^Advanced HIV Disease, Clinton Health Access Initiative, Inc., Boston, MA, USA; ^2^Advanced HIV Disease, Clinton Health Access Initiative, Inc., Lilongwe, Malawi; ^3^Department of HIV and AIDS, Malawi Ministry of Health, Lilongwe, Malawi

**Background**: Cryptococcal meningitis (CM) is one of the lead causes of death among people living with HIV (PLWHIV). Guidance from World Health Organization (WHO) in 2022 recommends optimized treatment for patients with CM utilizing flucytosine (5FC) and liposomal amphotericin B (L‐AmB), which is estimated to improve survival by up to 70% over fluconazole monotherapy. However, drug toxicity and side effects are known barriers to clinician adoption of this optimal regimen over historically utilized fluconazole. In August of 2020, the Department of HIV and AIDS (DHA) in Malawi scaled CM screening and treatment to 118 sites across 28 districts and included 5FC and L‐AmB as the preferred induction treatment regimen in the National HIV Clinical Guidelines. We documented our approach by utilizing WhatsApp communications to improve HCW adoption of the WHO‐recommended optimal CM treatment regimen.

**Materials and methods**: In July 2021, we collected CM treatment data via the HIV patient treatment registers at CM treatment sites. Utilizing a Microsoft Excel AHD capture tool, the primary outcome data collected was the proportion of PLWHIV with confirmed CM via lumbar puncture with rapid cerebral spinal fluid (CSF) cryptococcal antigen assay who were managed with the optimal regimen. Following this analysis, we utilized the DHA antiretroviral treatment (ART) WhatsApp communication platform to disseminate updated guidelines, job aides, and instructional videos to sites where patients were not being managed optimally. Additionally, we opened a CM clinical hotline available to ART coordinators at hospitals who were treating CM patients to address clinical case questions, support the management of side effects, and immediately address stockouts of CM commodities.

**Results**: Data collected in July 2021 uncovered most CM patients were still receiving fluconazole monotherapy in place of 5FC and L‐AmB. However, following the utilization of the communication platforms, 92.6% of patients with confirmed CM received the optimal regimen in Malawi from July to December 2021.

**Conclusions**: National HIV and CM programs can learn from Malawi's experience utilizing electronic platform communications to improve uptake of the optimal CM treatment regimen, a vital component in the global effort to end CM deaths by 2030.

#### P139 Abstract withdrawn

### Co‐morbidities and Complications of Disease and/or Treatment: Ageing

#### P140 Age‐related differences in quality of life outcomes of people living with HIV

A Clark^1^, C Donatti^2^, N Nwokolo^1^, F Hennessy ^3^, A McMillan^3^, O Carter^3^, T Holbrook^3^, J Priest^4^

^1^Global Medical, ViiV Healthcare, Brentford, UK; ^2^Global Health Outcomes, ViiV Healthcare, Brentford, UK; ^3^Real World Evidence & Epidemiology, Adelphi Real World, Bollington, UK; ^4^Global Health Outcomes, ViiV Healthcare, Durham, NC, USA

**Background**: Today in the United States (US), people living with HIV (PLWHIV) can lead long and healthy lives if they are diagnosed early and remain on suppressive treatment; whether there are differences in disease burden and quality of life (QoL) based on age are unclear. We aimed to describe disease burden and QoL of PLWHIV in the US stratified by age.

**Table:** 

**Abstract P140 – Table 1. **Demographics and quality of life reported by physician and PLWHIV.							
QoL measures overall and by age		Total	18 to 30	31 to 50	51 to 60	61 to 70	71 to 82
Length of time since diagnosis (years)	n	489	66	260	103	48	12
	mean	6.8	1.8	5.2	10.4	13.3	14.9
Physician‐reported quality of life	n	600	82	311	132	62	13
Level of general bodily pain or discomfort because of their HIV (mean, 1 is no pain, 5 is extreme pain)	mean	1.5	1.3	1.5	1.6	1.6	1.8
Level of general fatigue because of their HIV (mean, 1 is no fatigue, 5 is extreme fatigue)	mean	1.8	1.7	1.8	1.8	1.8	1.9
Level of anxiety because of their HIV (mean, 1 is no anxiety, 5 is extreme anxiety)	mean	1.8	1.7	1.8	1.8	1.8	2.2
PLWHIV‐reported quality of life	n	249	29	131	57	26	6
Physical burden of taking HIV medication (1 = never, 5 = always)	mean	1.7	1.8	1.8	1.6	1.6	1.3
Mental burden of taking HIV medication (1 = never, 5 = always)	mean	2.0	2.0	2.1	1.8	1.8	1.5
Comfort about taking HIV medication in front of other people (1 = strongly disagree, 5 = strongly agree)	mean	2.8	2.7	2.8	2.5	3.3	2.7
Concern about being dependent on medication (1 = strongly disagree, 5 = strongly agree)	mean	3.2	3.0	3.3	3.1	3.4	2.3
PLWHIV‐reported PRO instruments							
EQ‐5D VAS score	n	241	29	126	55	25	6
	mean	81.6	87.2	81.2	82.6	75.0	79.8
EQ‐5D 5L ‐ US	n	235	29	124	51	25	6
	mean	0.86	0.94	0.86	0.85	0.76	0.79
PozQoL overall QoL ‐ average score [1 to 5]	n	248	29	131	56	26	6
	mean	3.4	3.3	3.2	3.5	3.5	4.0
Physician‐ and PLWHIV‐reported treatment satisfaction							
Physician‐stated treatment satisfaction (very satisfied)	n	599	81	311	132	62	13
	%	56%	54%	56%	58%	58%	69%
Patient‐stated treatment satisfaction (very satisfied)	n	249	29	131	57	26	6
	%	51%	48%	50%	47%	62%	100%

EQ‐5D 5L, EuroQol 5‐Dimension Health Questionnaire; EQ‐5D VAS, EuroQol 5‐Dimension Visual Analogue Scale; PRO, patient‐reported outcome; QoL, quality of life; US, United States.

**Materials and methods**: Data were drawn from the Adelphi HIV II Disease Specific Programme™, a survey of PLWHIV and their physicians (Table 1). Physicians provided patient demographics, comorbidities, satisfaction with HIV control and clinical details. Patients reported their satisfaction with treatment, and QoL using the EuroQoL 5‐Dimension Health Questionnaire (EQ‐5D) and the HIV‐specific PozQoL. Patients were categorized into age groups (18 to 30, 31 to 50, 51 to 60, 61 to 70 and 71 to 82), and descriptive analysis performed on these groups.

**Results**: Sixty physicians provided data for 600 PLWHIV, mean age 44.7 years, 76% male and 51% white. The most common comorbid conditions were hypertension (28%), dyslipidemia (22%), anxiety (19%) and depression (19%). Overall, 249 PLWHIV reported data directly. Table 1 shows the QoL data reported by physicians and PLWHIV. PLWHIV aged over 60 reported better QoL through the HIV‐specific PozQoL, but lower QoL through the EQ‐5D than all other age groups. Physicians stated patients aged over 70 had a higher level of pain and fatigue, correlating with EQ‐5D outcomes, but levels of anxiety due to HIV were similar across all cohorts. PLWHIV over 71 did not find taking their medication burdensome, and were not concerned about being dependent on medication, but were less comfortable taking medication in front of others.  Physicians were more satisfied with the current treatment than PLWHIV; however, PLWHIV aged over 61 were most satisfied with their treatment, more so than their physicians.

**Conclusions**: All PLWHIV reported an impact of their disease on overall QoL. Older PLWHIV were more accepting and less impacted by HIV specifically but had lower overall health‐related QoL, reflecting age‐related health issues. These data show an unmet need in this population and further studies and interventions could be explored to improve health‐related QoL in older PLWHIV.

#### P141 Re‐framing how frailty is identified, diagnosed and managed among people living with HIV: exploratory perspectives from clinical practice

T Barber ^1^, T Levett^2^, D Brown^3^, P Pristera^4^, N Galbraith^5^, B Patterson^6^, J Williams^7^, M Boffito^8^

^1^HIV Medicine, Royal Free London NHS Foundation Trust, London, UK; ^2^Elderly Medicine, University Hospitals Sussex NHS Foundation Trust, Sussex, UK; ^3^Specialist Physiotherapy ‐ HIV, Chelsea and Westminster NHS Foundation Trust, London, UK; ^4^Patient Engagement Strategy Lead, Cuttsy + Cuttsy, Cambridge, UK; ^5^HIV Standards Support Team, Gilead Sciences, London, UK; ^6^HIV, Gilead Sciences, London, UK; ^7^Community Specialist HIV Nursing Service, Liverpool University Hospitals NHS Foundation Trust, Liverpool, UK; ^8^HIV Medicine, Chelsea and Westminster NHS Foundation Trust, London, UK

**Background**: Definitions and guidance traditionally position frailty as an 'age‐related' or 'geriatric' construct associated heavily with functional or mobility impairment. People living with HIV can present with frailty at an earlier age and via less typical routes than the general public [1‐3]. Prevalence of disability in this population is also high [4]. Despite more than 50% of people living with HIV in the UK estimated to be aged ≥50 by 2028 [5], there is still no consensus on how UK healthcare practitioners should identify, diagnose and manage frailty in this population. Frailty tools exist but are either not sufficiently validated or labelled as 'inappropriate' in the context of HIV [2,6]. We explored the perspectives and experiences of senior practitioners to understand what works in practice and what else needs to happen to improve uptake and models of care across the field.

**Materials and methods**: Semi‐structured interviews were conducted virtually with six healthcare professionals purposively selected for addressing frailty across HIV care in England. Interviews were audio‐recorded, transcribed and thematically analysed to (A) explore current practice, (B) identify key barriers, and (C) curate suggestions to make identification, assessment and management of frailty easier in practice.

**Results**: The interviews highlighted three core barriers to identifying, diagnosing and managing frailty in HIV: (1) lack of awareness and understanding within the field, (2) lack of optimised tools and processes for frailty/pre‐frailty in HIV, and (3) lack of care coordination, integration and resource. Approaches varied across centres but increasing and improving identification of those living with HIV and at risk of frailty was seen as the greatest priority by all. Suggested actions were compiled and a new ‘FRAIL in HIV’ acronym co‐developed to support staff in looking beyond a ‘FRAIL’ scale to consider implications in the wider context of HIV.

**Conclusions**: Addressing frailty in people living with HIV requires a more holistic approach than traditional models of care, and taking small actions now was seen as better than waiting for a unified approach. Our collated guidance hopes to reframe frailty in the context of HIV and address existing barriers to screening, diagnosis and management in clinical practice.

**References**

1. Jones HT, Levett T, Barber TJ. Frailty in people living with HIV: an update. Curr Opin Infect Dis. 2022;35:21‐30.

2. Levett T, Wright J. How to assess and manage frailty in patients with HIV. Sex Transm Infect. 2017;93:476‐7.

3. Levett TJ, Cresswell FV, Malik MA, Fisher M, Wright J. Systematic review of prevalence and predictors of frailty in individuals with human immunodeficiency virus. J Am Geriatr Soc. 2016;64:1006‐14.

4. Brown DA, O'Brien KK, Harding R, Sedgwick PM, Nelson M, Boffito M, et al. Prevalence, severity, and risk factors of disability among adults living with HIV accessing routine outpatient HIV care in London, United Kingdom (UK): a cross‐sectional self‐report study. PLoS One. 2022;17:e0267271.

5. Yin Z, Kall M, Skingsley A, Brown A, Kirwan P, Delpech V. Over half of people in HIV care in the United Kingdom by 2028 will be aged 50 years or above. HIV Med. 2017;18:53.

6. Brothers TD, Rockwood K. Frailty: a new vulnerability indicator in people aging with HIV. Eur Geriatr Med. 2019;10:219‐26.

#### P142 Frailty status and associated factors among older PLWHIV in Southern Ethiopia

E Woldesemayat ^1^, N Clair‐Sullivan^2^, A Kassa^3^, T Gari^1^, K Gutema^1^, N Chea^1^, K Wubshet^4^, N Bogale^4^, A Asefa^5^, J Vera^2^

^1^School of Public Health, Hawassa University, Hawassa, Ethiopia; ^2^Brighton and Sussex Medical School, University of Sussex, Brighton, UK; ^3^School of Nursing, Hawassa University, Hawassa, Ethiopia; ^4^School of Medicine, Hawassa University, Hawassa, Ethiopia; ^5^College of Health Sciences, Jima University, Jima, Ethiopia

**Background**: Studies addressing frailty are limited in the global south including Ethiopia. We measured the prevalence of frailty and associated factors among older people living with HIV (PLWHIV) compared to HIV negative controls attending a large Comprehensive Specialized Hospital in southern Ethiopia.

**Methods**: A systematic sample of 187 PLWHIV and 187 HIV negative controls >50 years old were recruited from the Hawassa University Hospital between 1 October and 30 November 2021. Data on socio‐demographic, behavioural and clinical characteristics were collected using a structured questionnaire. Frailty assessments were completed using a validated screening tool, the brief frailty instrument (B‐FIT‐2), consisting 6 components (cognition, physical function, depression, social function, nutrition and sensory impairments). Having 5 to 6 were frail, 2 to 4 points were pre‐frail and below 2 were considered as non‐frail. Multinomial logistic regression model was used to measure association between various characteristics and frailty.

**Results**: Median age (range) was 53 (50 to 80) for PLWHIV and 59 (50 to 88) for controls. For PLWHIV the median (range) CD4 count was 570 (58 to 3211). Prevalence of frailty was 9.1% for PLWHIV versus 5.9% for HIV negative controls. There was statistically significant difference among the two groups by most socio‐demographic characteristics. A significant proportion of PLWHIV were pre‐frail 141 (75.4%) compared to controls 110 (58.8%). Pre‐frailty status was associated with HIV diagnosis (adjusted odds ratio (aOR) 3.7; 95% CI 1.4 to 10.0), low age (aOR 0.3; 95% CI 0.2 to 0.7), lower educational attainment (aOR 3.9; 95% CI 1.0 to 14.8) and having high or low body mass index (BMI) (aOR 11.0; 95% CI 4.4 to 27.5). HIV diagnosis (aOR 1.5; 95% CI 1.7 to 12.7), lower educational attainment (aOR 51.1; 95% CI 3.2 to 822.1), single status (aOR 3.7; 95% CI 3.7 to 3.7), farmers (aOR 11.4; 95% CI 1.1 to 122.3) and high or low BMI predicted frailty (Table 1).

**Conclusion**: A high proportion of frailty and pre‐frailty was observed in a cohort of relatively younger PLWHIV attending care in Southern Ethiopia. Future research should focus on interventions targeting factors associated with pre‐frail and frail status.

**Table:** 

**Abstract P142 – Table 1**. Multinomial regression analysis for the risk factors of frailty status among the study participants.				
Characteristics	Pre‐frail		Frail	
	cOR (95% CI)	aOR (95% CI)	cOR (95% CI)	aOR (95% CI)
Patient type PLWHIV	2.9 (1.8 to 4.8)	3.7 (1.4 to 10.0)	3.5 (1.5 to 8.4)	1.5 (1.7 to 12.7)
Age 50 to 58 years	0.4 (0.2 to 0.7)	0.3 (0.2 to 0.7)	0.3 (0.1 to 0.7)	0.6 (0.2 to 2.8)
Education				
No education	6.4 (3.2 to 13.0)	3.9 (1.0 to 14.8)	19.5 (4.0 to 95.7)	51.1 (3.2 to 822.1)
Primary	3.2 (1.8 to 5.6)	2.4 (0.9 to 6.5)	5.2 (1.1 to 25.1)	6.0 (0.5 to 79.8)
Marital status				
Single			0.3 (0.3 to 0.3)	3.7 (3.7 to 3.7)
Married	0.4 (0.2 to 0.8)	1.7 (0.6 to 4.7)	0.1 (0.04 to 0.3)	0.5 (0.1 to 2.7)
Divorced	0.4 (0.1 to 1.0)	1.4 (0.4 to 5.8)	0.3 (0.1 to 1,5)	4.6 (0.4 to 49.7)
Occupation				
Housewife	3.8 (1.6 to 8.8)	3.2 (0.8 to 12.4)	8.3 (2.0 to 34.4)	1.9 (0.1 to 26.5)
Employed/pensioned	0.6 (0.4 to 1.1)	1.2 (0.5 to 3.1)	0.9 (0.4 to 1.1)	2.6 (0.4 to 16.9)
Farmer or other	3.3 (1.3 to 8.6)	3.3 (0.8 to 12.8)	15.1 (3.6 to 63.7)	11.4 (1.1 to 122.3)
Low or high BMI	8.0 (3.8 to 16.5)	11.0 (4.4 to 27.5)	23.9 (8.2 to 69.6)	69.6 (15.0 to 323.5)

#### P143 Effect of online education on physician knowledge and confidence regarding the impact of inflammation and the failure to recover CD4 T cell counts in people living with HIV

J Duffey ^1^, S Voorn^2^, C Ní Cheallaigh^3^, M Young Karris^4^, P Hunt^5^, G Guaraldi^6^

^1^Clinical Strategy, WebMD Global LLC, New York, NY, USA; ^2^Medical Education, WebMD Global LLC, New York, NY, USA; ^3^Department of Clinical Medicine, Trinity College, Dublin, Ireland; ^4^Department of Medicine, University of California San Diego, San Diego, CA, USA; ^5^Division of Experimental Medicine, University of California, San Francisco, CA, USA; ^6^Modena HIV Metabolic Clinic, University of Modena and Reggio Emilia, Modena, Italy

**Background**: People living with HIV (PLWHIV) experience persistent inflammation due to HIV itself, other chronic co‐infections and comorbidities such as obesity. Multiple various triggers contribute to low‐grade chronic inflammation. Additionally, a subset of PLWHIV also fail to recover CD4 T cell counts despite virologic suppression due to factors such as poor adherence to antiretrovirals, inflammation or lymph node fibrosis. We assessed whether two online independent medical education activities could improve the knowledge and confidence of HIV physicians regarding multiple aspects of chronic immune dysfunction and inflammation in PLWHIV and the relationship between the failure to recover CD4 T cell counts and the interaction between the immune system and morbidity and mortality.

**Materials and methods**: For both activities the educational effect was assessed using a repeated‐pairs design with pre‐/post‐assessment. Six multiple choice questions assessed knowledge, and two questions assessed confidence. Statistical tests to assess significance included: paired samples t‐test for overall average number of correct responses and confidence. McNemar's test for individual questions and learning objectives  (p < 0.05). Cohen's d estimated the effect size impact on number of correct responses (<0.20 modest, 0.20 to 0.49 small, 0.59 to 0.79 moderate, ≥0.80 large). Data were collected from 11/23/2021 to 3/1/2022.

**Results**: From a total of audience of 897 HIV physicians there were 188 assessment completers. Both activities resulted in significant overall knowledge gains with a large educational impact (p < 0.001 and Cohen's d 0.93). HIV physicians reported a 61% increase in confidence in their ability to discuss with their HIV‐positive patient the implications of low CD4 counts (confidence shift 64%) and a 54% increase in confidence in their ability to manage inflammation‐related comorbidities in PLWHIV (confidence shift 88%). Specifically, the relative change from baseline knowledge to post‐activity knowledge was highly significant in the use of interventions to manage comorbidities associated with inflammation (138%; p < 0.001) and the impact of the failure to recover CD4 T cell counts on morbidity and mortality (867%; p < 0.001)

**Conclusions**: Online medical education significantly improved HIV physician knowledge and confidence regarding the causes, consequences and management of inflammation and the failure to recover CD4 T cell counts in PLWHIV.

#### P144 Are iron metabolism parameters and relative telomere length possible biomarkers of ageing in HIV‐infected patients?

G Dragovic Lukic ^1^, S De Luka^2^, A Trbovich^2^, B Obradovic^1^, D Jevtovic^3^, B Toljic^4^, J Milasin^4^

^1^Department of Pharmacology & Clinical Pharmacology, University of Belgrade, School of Medicine, Belgrade, Serbia; ^2^Department of Pathological Physiology, University of Belgrade, School of Medicine, Belgrade, Serbia; ^3^Infective and Tropical Disease Hospital, University of Belgrade, School of Medicine, Belgrade, Serbia; ^4^Department of Human Genetics, Faculty of Dental Medicine, University of Belgrade, Belgrade, Serbia

**Background**: To date relative telomere length (RTL) and iron metabolism parameters (IMP) have attracted great deal of attention as candidates for biomarkers of ageing (BoA). During the last decade, a number of studies have documented age‐related iron accumulation in animal models [1]. There are still not enough data in humans, especially not in HIV‐infected patients [2]. On the other hand, the evidence suggesting RTL as a biomarker of ageing in humans is equivocal. Thus, we aim to investigate whether iron metabolism parameters and RTL meet the criteria to be considered as a BoA in HIV/AIDS patients.

**Material & methods**: In this cross‐sectional study we included 48 HIV‐infected patients older then 18 years on combined antiretroviral therapy for at least 12 months. Exclusion criteria were co‐infections with hepatitis B virus (HBV), and/or hepatitis C virus (HCV), TB, any acute diseases, radiotherapy or cytotoxic drug therapy, alcohol and/or narcotics usage. RTL was estimated by real‐time PCR. IMP considered of importance for BoA were following: serum iron concentration, transferrin iron binding capacity (TIBC), transferrin saturation, serum transferrin and ferritin concentrations. Spearman correlation analysis was used to assess correlation between continuous variables with non‐normal distribution. All p‐values less than 0.05 were considered significant.

**Results**: HIV‐infected patients characteristics were: mean age was 49.4 years (range: 23 to 79 years), median CD4+T‐count 567.0±317.7 cells/mm^3^, HIV RNA plasma viral load (pVL) was undetectable (pVL <50 copies/mL) in all patients. Mean plasma levels of serum iron, transferrin, TIBS and the percentage of transferrin saturation were 81.83±29.03 mg/dL, 36.70±16.08 μmol/L, 201.28±82.51 μg/dL and 41.97±12.37, respectively. Mean RTL was 0.77 (range: 0.17 to 1.74). Correlation analysis revealed significant relationship between serum iron and RTL (Rho = ‐0.365; p = 0.034) and transferrin and RTL (Rho = 0.350; p = 0.031).

**Conclusions**: Serum iron as well as transferrin levels could serve as possible biomarkers of ageing. More studies examining the relationships between relative telomere length and iron metabolism parameters and with measures that decline with 'normal' ageing in community samples are required.

**References**

1. Mather KA, Jorm AF, Parslow RA, Christensen H. Is telomere length a biomarker of aging? A review. J Gerontol A Biol Sci Med Sci. 2011;66:202‐13.

2. Aviv A, Valdes AM, Spector TD. Human telomere biology: pitfalls of moving from the laboratory to epidemiology. Int J Epidemiol. 2006;35:1424‐9.

### Co‐morbidities and Complications of Disease and/or Treatment: Cardiovascular

#### P145 Cardiovascular events in delayed presentation of HIV: the prospective PISCIS cohort study

R Martin‐Iguacel ^1^, M Vazquez‐Friol^2^, J Burgos^3^, A Bruguera^1^, J Reyes‐Urueña^1^, S Moreno‐Fornés^1^, J Aceitón^1^, Y Díaz^1^, P Domingo^4^, M Saumoy^5^, H Knobel^6^, D Dalmau^7^, B Borjabad^8^, I Johansen^9^, J Miró^10^, J Casabona^1^, J Llibre^11^

^1^CEEISCAT, Centre of Epidemiological Studies of HIV/AIDS and STI of Catalonia (CEEISCAT), Health Department, Generalitat de Catalunya, Badalona, Spain; ^2^Infectious Diseases Department, University Hospital of Ferrol, A Coruña, Spain; ^3^Infectious Diseases Department, Hospital Universitari de la Vall d'Hebron, Barcelona, Spain; ^4^Infectious Diseases Department, Hospital Universitari de la Santa Creu i Sant Pau, Barcelona, Spain; ^5^Department of Internal Medicine and Infectious Diseases, Hospital Universitari de Bellvitge, Hospitalet de Llobregat, Barcelona, Spain; ^6^Infectious Diseases Department, Hospital del Mar ‐ Parc de Salut MAR, Barcelona, Spain; ^7^Internal Medicine Department, Hospital Universitari Mútua Terrassa, Barcelona, Spain; ^8^Internal Medicine Department, Consorci Sanitari Integral, Hospitalet del Llobregat, Barcelona, Spain; ^9^Infectious Diseases Department, Odense University Hospital, Odense, Denmark; ^10^Hospital Clínic, Hospital Clínic‐Institut d'Investigacions Biomèdiques August Pi i Sunyer, University of Barcelona, Barcelona, Spain; ^11^Infectious Diseases Department, University Hospital Germans Trias i Pujol, Badalona, Spain

**Background**: Late HIV presenters (LP; CD4 ≤350 cells/μL) have increased risks of AIDS and non‐AIDS comorbidities, but the impact of late HIV presentation on cardiovascular events (CVE) remains unclear.

**Figure:**
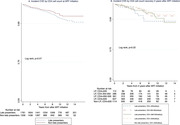
**Abstract P145 – Figure 1**. Kaplan‐Meier curves of time to CV events or all‐cause mortality. (A) Incident cardiovascular events by CD4 cell count at ART initiation; (B) Incident CV events by CD4 counts 2 years after ART initiation.

**Materials and methods**: From the prospective, multicentre PISCIS cohort, we included all adult people living with HIV (PLWHIV) in Catalonia initiating antiretroviral therapy (ART) between 2005 and 2019 without previous CVE. Additional data were retrieved from PADRIS, a central research‐oriented database that gathers real‐world health data generated by the public health system. The primary outcome was incidence of first CVE (ischaemic heart disease, congestive heart failure, cerebrovascular or peripheral vascular disease). The secondary outcome was all‐cause mortality after the first CVE. We used Poisson regression.

**Results**: We included 3317 PLWHIV (26 589.1 person‐years [PY], median follow‐up 8.0 years [IQR 5.0 to 11.2]): 1761 (53.1%) LP and 1556 (46.9%) non‐LP. Overall, 163 (4.9%) experienced a CVE (IR 6.1/1000PY [95% CI 5.3 to 7.1]): 105 (6.0%, IR 6.9/1000PY [95% CI 5.7 to 8.3]) LP versus 58 (3.7%, IR 5.1/1000PY [95% CI 3.9 to 6.6]) non‐LP. No differences were observed in the multivariate analysis adjusting for age, transmission mode, AIDS‐defining events, comorbidities (diabetes mellitus type 2, arterial hypertension, dyslipidaemia, chronic kidney, lung, or liver disease, malignancy or depression), and calendar time, regardless of baseline CD4 (aIRR 0.92 [0.62 to 1.36] and 0.84 [0.56 to 1.26] in LP with CD4 count <200 and 200 to ≤350 cells/μL, respectively, compared to non‐LP) (Table 1). Sensitivity analyses including only PLWHIV surviving the first 2 years yielded similar results, also among immunological non‐responders with 2‐year CD4 <200 cells/μL. Kaplan‐Meier curves for incident CV events stratified by CD4 count at ART initiation or by their 2‐year CD4 recovery showed no significant differences between groups (Figure 1). Overall mortality was 8.5% in LP versus 2.3% in non‐LP (p < 0.001). Mortality after the CVE was 31/163 (19.0%), with no differences between groups (aMRR 1.24 [0.45 to 3.44]). Women versus MSM, and individuals with chronic lung and liver disease, experienced a particularly high mortality after the CVE (aMRR 5.89 [1.35 to 25.60], 5.06 [1.61 to 15.91], and 3.49 [1.08 to 11.26], respectively).

**Conclusions**: LP without previous CVE did not exhibit an increased long‐term risk of CVE compared with non‐LP. Mortality after a first CVE was high, but no differences were observed between LP and non‐LP.

**Table:** 

**Abstract P145 – Table 1**. Incidence rate of a first CV event in the overall cohort, including all LPs and non‐LPs.				
	CV events (n)	IR per 1000 PY (95% CI)	IRR (95% CI)	aIRR (95%CI)^a^
Total	163	6.1 (5.3 to 7.1)		
Age (time‐updated) (years)				
<40	29	2.4 (1.7 to 3.4)	Ref^a^	Ref^a^
40 to 49	55	6.1 (4.7 to 8.0)	2.58 (1.64 to 4.04)	2.11 (1.33 to 3.33)
5 to 59	54	13.1 (10.0 to 17.1)	5.49 (3.50 to 8.62)	3.87 (2.42 to 6.20)
≥60	25	19.1 (12.9 to 28.3)	8.04 (4.71 to 13.72)	4.56 (2.53 to 8.20)
HIV transmission mode				
MSM	57	3.6 (2.8 to 4.7)	Ref^a^	Ref^a^
Heterosexual men	47	12.5 (9.4 to 16.6)	3.48 (2.37 to 5.12)	2.43 (1.62 to 3.67)
Women	16	4.7 (2.9 to 7.7)	1.32 (0.76 to 2.30)	1.14 (0.65 to 1.99)
IDU	28	13.7 (9.5 to 19.9)	3.82 (2.43 to 6.01)	2.23 (1.35 to 3.71)
Unknown/other	15	9.9 (5.9 to 16.4)	2.75 (1.56 to 4.85)	1.98 (1.11 to 3.55)
CD4 cell count at ART initiation				
Late presenters, CD4 <200 cells/μL	63	8.7 (6.8 to 11.2)	1.71 (1.19 to 2.44)	0.92 (0.62 to 1.36)
Late presenters, CD4 200 to ≤350 cells/μL	42	5.3 (3.9 to 7.1)	1.03 (0.69 to 1.53)	0.84 (0.56 to 1.26)
Non‐late presenters, CD4 >350 cells/μL	58	5.1 (3.9 to 6.6)	Ref^a^	Ref^a^
Calendar time (time‐updated)				
2005 to 2009	8	4.5 (2.2 to 9.0)	0.72 (0.35 to 1.48)	0.81 (0.39 to 1.69)
2010 to 2014	47	6.3 (4.8 to 8.4)	1.02 (0.72 to 1.44)	1.16 (0.82 to 1.64)
2015 to 2021	108	6.2 (5.1 to 7.5)	Ref^a^	Ref^a^
AIDS‐defining event at ART initiation				
Yes	29	13.8 (9.6 to 19.9)	2.52 (1.69 to 3.77)	1.49 (0.95 to 2.34)
No	134	5.5 (4.6 to 6.5)	Ref^a^	Ref^a^
Comorbidities at baseline				
Diabetes	6	32.0 (14.4 to 71.3)	5.38 (2.38 to 12.17)	1.32 (0.51 to 3.39)
Arterial hypertension	16	14.3 (8.8 to 23.3)	2.48 (1.48 to 4.15)	1.83 (1.07 to 3.11)
Dyslipidaemia	13	16.5 (9.6 to 28.5)	2.84 (1.61 to 5.01)	1.34 (0.71 to 2.55)
Chronic kidney disease	3	31.0 (10.0 to 96.1)	5.13 (1.64 to 16.08)	1.98 (0.58 to 6.76)
Chronic lung disease	6	26.5 (11.9 to 59.0)	4.45 (1.97 to 10.06)	1.67 (0.71 to 3.90)
Chronic liver disease	25	20.3 (13.7 to 30.1)	3.74 (2.44 to 5.72)	2.38 (1.48 to 3.82)
Malignancy	11	22.4 (12.4 to 40.5)	3.85 (2.09 to 7.10)	2.27 (1.20 to 4.30)
Depression	14	9.9 (5.9 to 16.8)	1.68 (0.97 to 2.90)	1.08 (0.61 to 1.91)

aIRR, adjusted incidence rate ratio; CVD, cardiovascular disease (includes ischaemic heart disease, congestive heart failure, cerebrovascular disease, peripheral vascular disease); IDU, injection drug use; IR, incidence rate; IRR, incidence rate ratio.

^a^Adjusted for age, transmission mode, CD4 cell count at ART initiation, calendar time, AIDS‐defining event at ART initiation and comorbidities (diabetes mellitus type 2, arterial hypertension, dyslipidaemia, chronic kidney, lung, or liver disease, malignancy or depression).

#### P146 Prevalence of cardiovascular disease (CVD) and comparison of risk category predictions of Systemic Coronary Risk Evaluation 2 (SCORE2) and four other CVD risk calculators among PLWHIV in Turkey

E Tükenmez Tigen ^1^, D Gökengin^2^, H Özkan Özdemir^3^, H Akalın^4^, B Kaya^5^, A Deveci^6^, A İnan^7^, D İnan^8^, A Altunsoy^9^, A Özel^10^, İ Karaoğlan^11^, H Eraksoy^12^, T Demirdal^13^, T Yıldırmak^14^, S Birengel^15^, A İnci^16^, A Nazlı^17^, B Kayaaslan^9^, S Sayın Kutlu^18^, Ç Ataman Hatipoğlu^19^, Y Esen^20^, T Koç^20^, V Korten^1^

^1^Infectious Disease and Clinical Microbiology, Marmara University School of Medicine, Istanbul, Turkey; ^2^Infectious Disease and Clinical Microbiology, Ege University, İzmir, Turkey; ^3^Infectious Disease and Clinical Microbiology, Izmir Bozyaka Research and Training Hospital, İzmir, Turkey; ^4^Infectious Disease and Clinical Microbiology, Bursa Uludag University Hospital, Bursa, Turkey; ^5^Infectious Disease and Clinical Microbiology, Istanbul Kartal Dr. Lütfi Kirdar Research and Training Hospital, İstanbul, Turkey; ^6^Infectious Disease and Clinical Microbiology, Samsun Ondokuz Mayis University Hospital, Samsun, Turkey; ^7^Infectious Disease and Clinical Microbiology, Istanbul Haydarpasa Numune Research and Training Hospital, İstanbul, Turkey; ^8^Infectious Disease and Clinical Microbiology, Antalya Akdeniz University Hospital, Antalya, Turkey; ^9^Infectious Disease and Clinical Microbiology, Ankara Bilkent City Hospital, Ankara, Turkey; ^10^Infectious Disease and Clinical Microbiology, Ümraniye Research and Training Hospital, İstanbul, Turkey; ^11^Infectious Disease and Clinical Microbiology, Gaziantep University Hospital, Gaziantep, Turkey; ^12^Infectious Disease and Clinical Microbiology, Istanbul University Istanbul Medicine Faculty Hospital, İstanbul, Turkey; ^13^Infectious Disease and Clinical Microbiology, Izmir Katip Çelebi University Atatürk R&T Hospital, İzmir, Turkey; ^14^Infectious Disease and Clinical Microbiology, Istanbul Prof. Dr. Cemil Tascioglu City Hospital, İstanbul, Turkey; ^15^Infectious Disease and Clinical Microbiology, Ankara University Hospital, Ankara, Turkey; ^16^Infectious Disease and Clinical Microbiology, Istanbul Research and Training Hospital, İstanbul, Turkey; ^17^Infectious Disease and Clinical Microbiology, Izmir 9 Eylül University Hospital, İstanbul, Turkey; ^18^Infectious Disease and Clinical Microbiology, Denizli Pamukkale University Hospital, Denizli, Turkey; ^19^Infectious Disease and Clinical Microbiology, Ankara Research and Training Hospital, Ankara, Turkey; ^20^Medical Affairs, Merck Sharp Dohme, Istanbul, Turkey

**Background**: CVD is a major cause of mortality among PLWHIV [1,2]. Data on the agreement between the commonly used risk estimation equations in Turkey are limited. We aimed to determine prevalence of CVD risk and compare the degree of agreement between Atherosclerotic Cardiovascular Disease (ASCVD), Framingham (FRS‐CVD), Modified Framingham (Mod‐FRS), Data Collection on Adverse Events of Anti‐HIV Drugs (D:A:D) and SCORE2 in a multicentre cohort.

**Materials and methods**: This retrospective cross‐sectional study included adult PLWHIV with a follow‐up visit between October 2019 and 2021 in 20 tertiary centres. Inclusion criteria were age 40 to 75 years and receiving antiretrovirals (ARVs) for at least 6 months. The data for the calculation of risk‐scores were collected with a standardised form. Web‐based tools for each score were used for calculations in the subgroup with no known CVD and not using lipid‐lowering treatments. The patients were categorised as high/very high and non‐high risk. Agreement between the scores was assessed by Cohen's kappa (k) statistics.

**Results**: Of 1425 patients (82.7% male) 151 had a confirmed CVD (10.6%). Median (IQR) age was 51 (45 to 58) years. Prevalence of CVD risk factors were: 45.7% current smoking, 34.9% hyperlipidaemia, 29.5% hypertension, 18.3% obesity, 17% diabetes mellitus and 7.2% family history of early‐onset CVD. One thousand, one hundred and thirty‐two PLWHIV were eligible to assess CVD risk‐scores. Risk strata distributions are displayed in Table 1. The FRS‐CVD, Mod‐FRS, D:A:D‐reduced and SCORE2 had an overall agreement of 82%, 94%, 91% and 36% compared with ASCVD (k = 0.42, 0.64, 0.55 and 0.06) respectively and agreement was higher for lower scores. According to the European [3] and the American Cardiology guidelines [4], 75.3% and 47.1% of patients would be eligible for lipid‐lowering agents, respectively.

**Conclusions**: We found moderate agreement among CVD risk prediction tools evaluated in this study, except for SCORE2 which attributed a considerably higher CVD risk in 71.7% of patients. Whether those scores accurately estimate risk at population level needs further evaluation. CVD risk among PLWHIV in Turkey might be underestimated, therefore close‐monitoring of CVD risk is warranted. Furthermore, the potential impact of ARVs on CVD risk factors and strategies to reduce clinical risk, such as initiation of lipid‐lowering agents should be strongly considered.

**Table:** 

**Abstract P146 – Table 1**. CVD risk prediction strata according to different CVD risk prediction models^a^.					
n = 1132					
ASCVD	Low risk (<5%) n (%)	Borderline risk (5% to 7.5%) n (%)	Intermediate risk (7.5% to 20%) n (%)	High risk (≥20%) n (%)	
	459 (40.5)	189 (16.7)	378 (33.4)	106 (9.4)	
FRS‐CVD 10 year	Low risk (<10%) n (%)		Moderate risk (10% to 20%) n (%)	High risk (≥20%) n (%)	
	464 (41)		372 (32.9)	296 (26.1)	
Modified Framingham	728 (64.3)		304 (26.9)	100 (8.8)	
	<1% n (%)	1% to 5% n (%)	5% to 10% n (%)	>10% n (%)	
D:A:D‐reduced 5 year	62 (5.5)	651 (57.5)	276 (24.4)	143 (12.6)	
D:A:D‐reduced 10 year	8 (0.7)	319 (28.2)	375 (33.1)	430 (38)	
SCORE2/SCORE2‐OP for high risk countries	Low‐moderate n (%)			High risk n (%)	Very high risk n (%)
	320 (28.3)			615 (54.3)	197 (17.4)

ASCVD, Atherosclerotic Cardiovascular Disease; FRS‐CVD, Framingham Heart Study General CVD; D:A:D, Data Collection on Adverse Events of Anti‐HIV Drugs; SCORE2, Systemic Coronary Risk Evaluation Score 2; SCORE2‐OP, SCORE2‐Older Persons.

^a^Patients were considered at higher risk if their 10‐year CVD risk was >20% for FRS‐CVD and Mod‐FRS, >20% for ASCVD, 5‐year risk >10% for D:A:D and high/very high risk for SCORE2 for high‐risk countries.

**References**

1. Escarcega RO, Franco JJ, Mani BC, Vyas A, Tedaldi EM, Bove AA. Cardiovascular disease in patients with chronic human immunodeficiency virus infection. Int J Cardiol. 2014;175:1‐7.

2. Shahbaz S, Manicardi M, Guaraldi G, Raggi P. Cardiovascular disease in human immunodeficiency virus infected patients: a true or perceived risk? World J Cardiol. 2015;7:633‐44.

3. Visseren FLJ, Mach F, Smulders YM, Carballo D, Koskinas KC, Bäck M, et al. 2021 ESC Guidelines on cardiovascular disease prevention in clinical practice. Eur Heart J. 2021;42, 3227‐337.

4. Arnett DK, Blumenthal RS, Albert MA, Buroker AB, Goldberger ZD, Hahn EJ, et al. 2019 ACC/AHA guideline on the primary prevention of cardiovascular disease: a report of the American College of Cardiology/American Heart Association Task Force on Clinical Practice Guidelines. Circulation 2019;140:e596‐e646.

#### P147 Study of the cardiovascular disease risk in people living with HIV‐1 in Portugal

F Palha ^1^, M Casella^2^, M Caixas Lima^2^, F Roxo^3^, S António^3^, C Piñeiro^4^, J Soares^4^, R Pinho^5^, L Pedro^5^, J Oliveira^6^, F Cunha^6^, R Pereira^7^, P Proença^7^, J Ferreira^8^, A Pimenta Castro^8^, C Delgado^1^

^1^Medical Department, Gilead Sciences, Lisbon, Portugal; ^2^Infectious Disease, Centro Hospitalar de Setúbal, Setúbal, Portugal; ^3^Internal Medicine, Hospital Distrital de Santarém, Santarém, Portugal; ^4^Infectious Disease, Centro Hospitalar Universitário de São João, Porto, Portugal; ^5^Internal Medicine, Hospital de Portimão, Centro Hospitalar e Universitário do Algarve, Portimão, Portugal; ^6^Infectious Disease, Centro Hospitalar e Universitário de Coimbra, Coimbra, Portugal; ^7^Infectious Disease, Serviço de Infeciologia do Hospital de Faro, Centro Hospitalar e Universitário do Algarve, Faro, Portugal; ^8^Internal Medicine, Serviço de Medicina Interna do Hospital de Faro, Centro Hospitalar e Universitário do Algarve, Faro, Portugal

**Background**: With more effective and widespread treatment of HIV, morbidity and mortality from non‐AIDS‐related events have surpassed those from AIDS‐related events. Cardiovascular disease (CVD) has emerged as an important cause of death in people living with HIV. HIV infection is associated with an increased risk of coronary artery disease, heart failure, ischaemic stroke, and lower extremity arterial disease beyond that explained by traditional atherosclerotic risk factors.

**Objective**: The aim of this study is to characterise the risk for CVD mortality in adult people living with HIV‐1 (PLWHIV) in mainland Portugal.

**Methods**: This is a multicentre, cross‐sectional, observational study of PLWHIV, aged 40 to 70 years, who attended a routine visit at one of seven hospital centres between September 2019 and March 2020. Clinical and laboratorial data were collected to determine each patient's 10‐year risk of CVD mortality. The main outcome is the percentage of PLWHIV in each risk category defined in the 2019 European Society of Cardiology/European Atherosclerosis Society guidelines for the management of dyslipidaemias.

**Results**: Overall, 566 PLWHIV were included, of whom 69.6% are male. The mean age of the study population is 51.6±7.4 years, and 56.4% of patients are 50 years old. Median time of HIV diagnosis is 13.6 years (interquartile range 8.0 to 19.6). Viral load was undetectable (<50 copies/mL) in 533/563 (94.7%) patients. CVD risk factors were common: 50.2% current smokers; 49.0% overweight or obese; 47.2% hypercholesterolaemia; 35.2% abdominal obesity; 27.6% hypertension; 9.0% diabetes mellitus. Established CVD was present in 81 (14.3%) patients. CVD risk was distributed as follows: 151 patients in the very high risk category (26.7%), 126 in the high risk category (22.3%), 208 in the moderate risk category (36.7%), and 81 in the low risk category (14.3%). Among the patients 50 years old, CVD risk was high or very high in 226 (70.8%).

**Conclusions**: In this unique multicentre observational study of PLWHIV in Portugal, aged 40 to 70 years, CVD risk was either high or very high in 49%. This result highlights the importance of routinely evaluating and addressing CVD risk in PLWHIV.

**References**

1. Neuhaus J, Angus B, Kowalska JD, La Rosa A, Sampson J, Wentworth D, et al. Risk of all‐cause mortality associated with nonfatal AIDS and serious non‐AIDS events among adults infected with HIV. AIDS. 2010;24:697‐706.

2. Antiretroviral Therapy Cohort Collaboration: Gill J, May M, Lewden C, Saag M, Mugavero M, Reiss P, et al. Causes of death in HIV‐1‐infected patients treated with antiretroviral therapy, 1996‐2006: collaborative analysis of 13 HIV cohort studies. Clin Infect Dis. 2010;50:1387‐96.

3. Mocroft A, Reiss P, Gasiorowski J, Ledergerber B, Kowalska J, Chiesi A, et al. Serious fatal and nonfatal non‐AIDS‐defining illnesses in Europe. J Acquir Immune Defic Syndr. 2010;55:262‐70.

#### P148 Evaluation of lipid profile and intima media thickness in HIV‐experienced patients treated with PI‐based regimens versus PI‐sparing regimens

S Martini, M Pisaturo, P Maggi, N Coppola

Infectious Diseases Unit, University of Campania Luigi Vanvitelli, Napoli, Italy

**Background**: Antiretroviral therapy has increasingly improved management of HIV infection, ensuring long‐term efficacy and tolerability. Each class of antiretrovirals has, however, different characteristics and different tolerability profiles. Literature data show that protease inhibitors (PIs) are associated with higher incidence of dyslipidaemia [1]. The aim of our study is to evaluate whether patients treated with PIs have both greater dyslipidaemia and increased IMT and atheromatous plaques compared to patients treated without PIs.

**Materials and methods**: To evaluate the association between PIs and dyslipidaemia associated with increased IMT, we enrolled 110 HIV‐experienced patients in a retrospective observational study. All enrolled patients were screened with Doppler ultrasonography of the supra‐aortic trunks in 2019. Patients were divided into two groups, 59 in the Cases group, treated with PIs, and 51 in Controls without PIs. In the two groups we evaluated lipids, cardiovascular risk factors (smoking, BMI, age, hypertension), increased IMT and eventual atheromatous plaques, assessed by Doppler ultrasonography (Table 1). We also performed a binary logistic regression analysis to assess the association of several patient factors (age, sex, BMI, smoke, lipids, PI regimen), to plaque appearance but without founding any significance.

**Figure:**
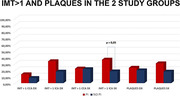
**Abstract P148 – Figure 1**. Evaluation of increased IMT and plaques in two groups of study.

**Results**: Analysis of the data showed a clear association between the Cases group and dyslipidaemia, although statistical significance was not achieved. Similarly, we observed a clear association between Cases and the evidence of increased IMT and plaques (Figure 1). In particular in the evaluation of left sections of carotid artery, Cases showed higher percentage of increased IMT than Controls (p = 0.05).

**Table:** 

**Abstract P148 – Table 1**. Characteristics of enrolled patients.				
Baseline data	Total	No PI/r controls	PI/r cases	p cases vs controls
Numbers of patients	110	51	59	
Age median (range)	52 (35‐80)	50 (31‐80)	56 (40‐85)	
Male/Female	85/25	41/10	44/15	
BMI > 24	58/110	32/51(62,7%)	42/59 (71,1%)	0.46
Risk factors for HIV infection	MSM 43 (47,77%) PWID 19 (7,03%) HETERO 46(45.18%) OTHER 2 (1,48%)	MSM 23 (44,96%) PWID 5 (6,20%) HETERO 22 (47,28%) OTHER 1 (1,55%)	MSM 20 (50,35%) PWID 14 (7,80%) HETERO 24 (43,26%) OTHER 1 (1,41%)	
Antiretroviral regimens	PI‐ based 59 (52%) NO PI ‐ based 51 (48%)	INI 13 (%) NNRTI 38 (%)	PI 46 (%) PI+INI 12 (%) PI+NNRTI 1 (%)	
Length of infection	10.5 ± 7.7	8.1 ± 6.5	13,4 ± 7,6	0.0002
Statin use	16/110	4/51 (2,04%)	12/59 (7,08%)	0.11
Other lower lipids drugs	22/110 (24,2%)	5/51 (2,55%)	17/59 (10,03 %)	0.11
Anti‐hypertensive drugs	29/110 (31,9%)	13/51 (6,63)	16/59 (9,44%)	0.98
Diabetes	8/110 (8,8%)	5/51 (2,55%)	3/59 (1,77%)	0.56
Smoker	45/110 (49,5%)	17/51 (8,67 %)	28/59 (16,52)	0.19
CDC CLASSIFICATION: C (late presenters)	C: 7 (11,48%)	C: 3 (5,88%)	C: 4 (6,77%)	0.86
CD4+, cell/μL mean ± SD	690,88 ± 311,38	691,42 ± 1104,42	690 ± 1167,43	0.99
CD4+ NADIR , cell/μL mean ± SD	317.56 ± 192.23	335 ± 194	270 ± 171.8	0.06
Triglycerides (mg/dl) mean± SD	145.8 ± 82.12	138,82 ± 91.47	153.34 ± 74.71	0.35
Cholesterol mean (mg/dl) ± SD	183.23 ± 33.13	178.64 ± 31.42	187.84 ± 34.99	0.15
HDL mean (mg/dl) ± SD	46.91 ± 12.99	49.78 ± 14.35	44.51 ± 11.45	0.03
LDL mean (mg/dl) ± SD	115 ± 31.17	109.64 ± 27.8	120 ± 34	0.08
% of elevated triglycerides >150 mg/dl	42/109 (38,53%)	16/51 (31,37%)	26/58 (44,82%)	0.21
% of elevated cholesterol > 200 mg/dl	32/109 (29,35%)	10/51 (19,6%)	22/58 (37,93%)	0.059
% of elevated HDL > 50 mg/dl	35/109 (32,11%)	19/51 (37,25%)	16/58 (27,58%)	0.38
% of elevated LDL > 160 mg/dl	8/109 (7,33%)	3/51 (5,88%)	5/58 (8,62%)	0.85
Hypertension	30/110 (33%)	13/51 (25,4%)	17/59 (28,8)	0.86
IMT > 1 CCA DX	12/110 (10.9%)	4/51 (7.84%)	8/58 (13.55%)	0.49
IMT > 1 ICA DX	29/110 (26.36%)	9/49 (17.64%)	20/59 (33.89%)	0.11
IMT > 1 CCA SX	25/109 (22.93%)	11/51 (21.56%)	13/58 (22.41%)	0.9
IMT > 1 ICA SX	30/108 (27.7%)	9/50 (18%)	21/58 (36.2%)	0.05
TOTAL PLAQUES DX	24/110 (21.81%)	10/51 (19.6%)	14/59 (23.7%)	0.77
TOTAL PLAQUES SX	28/110 (25.45%)	9/51 (17.64%)	18/59 (30.5%)	0.17

**Conclusions**: In conclusion, our real‐life data, although partial, show that patients treated with PIs have a trend to develop both greater dyslipidaemia, in accordance with the literature, and increased IMT and atheromatous plaques compared to patients treated without PIs. Our data reach statistical significance only for evidence of increased IMT in the left sections of carotid artery in Cases group. These findings, however, could be useful to optimise the therapy of patients with cardiovascular risk factors.

**Reference**

1. Maggi P, Di Biagio A, Rusconi S, Cicalini S, D'Abbraccio M, d'Ettorre G, et al. Cardiovascular risk and dyslipidemia among persons living with HIV: a review. BMC Infect Dis. 2017;17:551.

### Co‐morbidities and Complications of Disease and/or Treatment: Metabolic

#### P149 Long‐term risks of clinical obesity in the ADVANCE, NAMSAL and VISEND trials

A Hill ^1^, M Mirchandani^2^, B Simmons^3^, S Sokhela^4^, F Venter^4^

^1^Department of Pharmacology and Therapeutics, University of Liverpool, Liverpool, UK; ^2^Medicine, Imperial College London, London, UK; ^3^LSE Health, London School of Economics and Political Science, London, UK; ^4^Faculty of Health Sciences, Ezintsha, University of the Witwatersrand, Johannesburg, South Africa

**Background**: Integrase inhibitor‐based treatment and tenofovir alafenamide (TAF) have been associated with higher risks of weight gain and clinical obesity in a range of studies. Patients with clinical obesity show higher risks of cardiovascular disease, diabetes, and other adverse health conditions.

**Methods**: In ADVANCE, 1053 treatment‐naïve participants in South Africa were randomised to either TAF/FTC/DTG, TDF/FTC/DTG, or TDF/FTC/EFV and followed up to week 192. In NAMSAL, 613 treatment‐naïve participants in Cameroon were randomised to TDF/3TC/DTG or TDF/3TC/EFV for 192 weeks. In VISEND, 1201 NNRTI pre‐treated patients in Zambia were randomised to TAF/FTC/DTG, TDF/FTC/DTG, or PI‐based treatments for 144 weeks (stratified on baseline HIV RNA < or ≥1000 copies/mL). Kaplan‐Meier methods were used to evaluate probability of treatment‐emergent clinical obesity (BMI ≥30 kg/m^2^), with multivariable predictors evaluated by Cox proportional hazard models.

**Results**: In ADVANCE, by week 192, probability of obesity was 29% for patients on TAF/FTC/DTG, 18% on TDF/FTC/DTG, and 11% on TDF/FTC/EFV. In NAMSAL, by week 192, probability of obesity was 26% for TDF/3TC/DTG and 16% for TDF/3TC/EFV. In VISEND, across both strata, probability of obesity varied from 10% to 15%. Probabilities overall and by gender are shown in Table 1. TAF/FTC/DTG was predictive of obesity in ADVANCE (p < 0.001) and VISEND strata <1000 copies/mL (p = 0.002), but not in VISEND strata ≥1000 copies/mL. TDF/3TC/DTG was predictive of obesity in NAMSAL (p < 0.001). In all three trials, predictors of clinical obesity were female gender, higher baseline HIV RNA, and higher baseline BMI.

**Table:** 

**Abstract P149 – Table 1**. Probability of clinical obesity.				
Trial	Arm	Clinical obesity probability ‐ Men	Clinical obesity probability ‐ Women	Clinical obesity probability ‐ Overall
ADVANCE week 192	TAF/FTC/DTG	11%	42%	29%
ADVANCE week 192	TDF/FTC/DTG	8%	28%	18%
ADVANCE week 192	TDF/FTC/EFV	3%	20%	11%
NAMSAL week 192	TDF/3TC/DTG	28%	25%	26%
NAMSAL week 192	TDF/3TC/EFV	9%	20%	16%
VISEND BL<1000 copies/mL (week 96)	TAF/FTC/DTG	2%	22%	13%
VISEND BL<1000 copies/mL (week 96)	TDF/FTC/DTG	3%	14%	10%
VISEND BL>1000 copies/mL (week 96)	TAF/FTC/DTG	6%	14%	11%
VISEND BL>1000 copies/mL (week 96)	TDF/FTC/DTG	1%	19%	12%
VISEND BL>1000 copies/mL (week 96)	ZDV/3TC/LPVr	4%	14%	11%
VISEND BL>1000 copies/mL (week 96)	ZDV/3TC/ATVr	7%	21%	15%

**Conclusions**: Across three randomised trials in 2867 patients, the risks of clinical obesity were significantly higher for DTG‐based treatment, especially when combined with TAF/FTC. The adverse consequences of clinical obesity (e.g. diabetes, myocardial infarction) need to be factored into decisions on starting or switching to TAF/FTC/DTG, especially if already overweight at baseline.

#### P150 Understanding changes in metabolic parameters switching to 2DR or 3DR integrase strand inhibitors (InSTIs)

S Degroote, S Vanherrewege, E Tobback, E Caluwé, L Vincke, E Blomme, L Vandekerckhove, M De Scheerder

General Internal Medicine, Ghent University Hospital, Ghent, Belgium

**Background**: In HIV care we have been facing a paradigm change over the last years, culminating with the recommendation of a dual drug regimen (Dovato^®^; dolutegravir+lamivudine) both in naïve and in switch patients. Recent data show that newer antiretroviral therapies (ART), including integrase inhibitors and the nucleoside reverse transcriptase inhibitor, tenofovir alafenamide (TAF), are associated with weight gain. This raises the question of whether this has an impact on metabolic and cardiovascular health. We report the week 48 results of Rumba, the first randomised clinical trial evaluating the impact on metabolic health of switch from an integrase inhibitor (INI)‐based triple ART regimen Biktarvy^®^ towards Dovato^®^.

**Table:** 

**Abstract P150 – Table 1**. Descriptives of sociodemographic and metabolic variables at baseline and their change between week 48 and baseline.							
ITT‐E	N	Missing	Dovato (N = 87)	Biktarvy (N = 43)	p^a^	95% CI lower limit	95% CI upper limit
Baseline							
Gender, N male/N female	130	0	79/8	39/4	0.984		
Age (y), mean ± SD	130	0	47.31 ± 11.94	44.98 ± 11.60	0.292	‐2.03	6.70
Weight (kg), mean ± SD	130	0	81.21 ± 12.39	75.30 ± 13.00	0.013	1.26	10.55
Waist (cm), mean ± SD	128	2	95.35 ± 11.78	89.21 ± 11.20	0.006	1.82	10.46
BMI, median (IQR)	130	0	25.95 (23.37 to 28.43)	24.78 (21.78 to 26.13)	0.024	0.02	0.03
CD4 (cells/μL), median (IQR)	127	3	697.00 (554.25 to 931.25)	670.00 (522.00 to 916.00)	0.534	0.53	0.55
Fasting glucose (mg/dL), median (IQR)	118	12	88.5 (81.00 to 97.50)	89.50 (80.25 to 96.00)	0.789	0.79	0.81
Fasting insulin (mU/L), median (IQR)	117	13	9.40 (7.03 to 12.00)	8.60 (5.05 to 12.00)	0.203	0.20	0.21
HOMA_IR, median (IQR)	113	17	2.09 (1.44 to 2.84)	1.95 (1.04 to 2.79)	0.243	0.24	0.26
Triglycerides (mg/dL), median (IQR)	123	7	102.00 (79.00 to 141.50)	103.00 (72.00 to 151.00)	0.846	0.84	0.85
Cholesterol (mg/dL), mean ± SD	124	6	188.66 ± 36.73	193.36 ± 37.19	0.503	‐18.56	9.15
Cholesterol_HDL (mg/dL), median (IQR)	123	7	47.00 (40.00 to 55.00)	49.00 (39.75 to 54.25)	0.707	0.71	0.72
Cholesterol_LDL (mg/dL), median (IQR)	122	8	117.00 (100.50 to 136.75)	123.00 (105.75 to 138.00)	0.546	0.54	0.56
DXA_lean body (g), mean ± SD	130	0	55 540.95 ± 7349.08	53 282.33 ± 8858.59	0.126	‐646.57	5163.83
DXA_lean trunk (g), mean ± SD	130	0	27 887.73 ± 3943.05	26 514.77 ± 4618.68	0.080	‐167.67	2913.57
DXA_lean limb (g), mean ± SD	117	13	24 324.17 ± 3428.40	23 295.12 ± 4006.26	0.153	‐388.26	2446.36
DXA_trunk fat (g), median (IQR)	130	0	11 099.00 (8353.90 to 14 437.50)	9477.40 (6353.50 to 12 929.40)	0.018	0.02	0.02
DXA_limb fat (g), median (IQR)	129	1	10 557.20 (8347.15 to 12 937.40)	8744.10 (6658.30 to 11 246.30)	0.007	0.01	0.01
Fat percentage, median (IQR)	130	0	29.50 (24.10 to 33.70)	25.90 (21.30 to 30.40)	0.015	0.01	0.02
Total fat mass (g), median (IQR)	130	0	23 029.20 (18 071.20 to 2907.70)	20 176.60 (14 843.80 to 24 862.60)	0.010	0.01	0.01
Delta week 48 to baseline							
Weight (kg), mean ± SD	117	13	0.33 ± 4.91	0.17 ± 3.48	0.861	‐1.59	1.90
Waist (cm), mean ± SD	116	14	0.09 ± 4.68	0.90 ± 3.73	0.352	‐2.53	0.91
BMI, median (IQR)	117	13	‐0.06 (‐0.72 to 0.81)	0.08 (‐0.59 to 0.46)	0.954	0.96	0.96
CD4 (cells/μL), median (IQR)	110	20	‐10.00 (‐79.00 to 93.00)	‐50.00 (‐134.00 to 72.50)	0.097	0.09	0.10
Fasting glucose (mg/dL), median (IQR)	99	30	‐4.00 (‐12.00 to 6.00)	0.00 (‐7.00 to 6.00)	0.240	0.23	0.25
Fasting insulin (mU/L), median (IQR)	95	35	‐0.45 (‐2.75 to 2.23)	‐0.60 (‐4.70 to 1.00)	0.229	0.22	0.24
HOMA_IR, median (IQR)	91	39	‐0.07 (‐0.70 to 0.53)	‐0.21 (‐0.86 to 0.27)	0.306	0.30	0.32
Triglycerides (mg/dL), median (IQR)	105	25	‐7.00 (‐33.50 to 25.50)	‐5.00 (‐33.00 to 8.00)	0.568	0.56	0.58
Cholesterol (mg/dL), mean ± SD	110	20	‐2.25 ± 27.61	‐11.00 (‐37.00 to 11.50)	0.182	‐3.89	20.27
Cholesterol_HDL (mg/dL), median (IQR)	109	21	0.00 (‐4.00 to 4.00)	‐4.00 (‐7.50 to 1.00)	0.016	0.01	0.02
Cholesterol_LDL (mg/dL), median (IQR)	104	26	0.00 (‐16.00 to 13.65)	‐7.00 (‐30.60 to 8.50)	0.196	0.19	0.21
DXA_lean body (g), mean ± SD	118	12	132.29 ± 2268.77	‐640.01 ± 2496.40	0.095	‐136.95	1681.55
DXA_lean trunk (g), mean ± SD	116	14	95.19 ± 1248.99	‐499.53 ± 1529.56	0.028	67.04	1122.40
DXA_lean limb (g), mean ± SD	105	25	‐84.42 ± 1553.71	‐255.19 ± 1056.13	0.564	‐413.89	755.42
DXA_trunk fat (g), median (IQR)	116	14	144.70 (‐775.15 to 1000.30)	616.90 (‐308.10 to 1513.70)	0.062	0.06	0.07
DXA_limb fat (g), median (IQR)	117	13	45.05 (‐583.15 to 612.15)	387.30 (‐260.90 to 897.20)	0.174	0.16	0.18
Fat percentage, median (IQR)	116	14	0.10 (‐1.70 to 1.60)	1.00 (‐0.40 to 2.70)	0.016	0.01	0.02
Total fat mass (g), median (IQR)	117	13	291.50 (‐1324.40 to 1760.30)	755.35 (‐679.05 to 2383.25)	0.152	0.14	0.16

^a^Unpaired Student's t tests (when the data were normally distributed according to the Shapiro Wilk test of normality) or Mann‐Whitney U tests (in the other cases).

**Materials and methods**: In this open‐label, longitudinal study, patients were randomised 2:1 to switch to Dovato^®^ or to switch or stay on Biktarvy^®^. BMI, weight and waist circumference as well as lipids and insulin resistance (HOMA‐IR) were summarised for both groups. Dual‐energy x‐ray absorptiometry (DXA) scans were used to assess body composition and fat distribution. We report the differences in these metabolic parameters between week 48 and baseline.

**Results**: One hundred and thirty‐four patients were randomised and 130 patients (N = 87 Dovato^®^, N = 43 Biktarvy^®^) were included in the intention‐to‐treat efficacy analysis. Baseline sociodemographic characteristics were comparable between both groups. Baseline BMI, weight and waist circumference were significantly higher in the Dovato^®^ group, but did not significantly change within each treatment group after 48 weeks. Changes between week 48 and baseline were significantly different between the groups with regard to HDL cholesterol, DXA lean trunk mass and fat percentage, favoring Dovato^®^. We did not observe significant differences in other lipid parameters including triglycerides, LDL‐ and total cholesterol nor in fat distribution. Furthermore, changes in glucose, insulin and HOMA‐IR did not significantly differ between both groups (Table 1).

**Conclusions**: These preliminary data suggest that treatment with Dovato^®^ may have a favourable impact on metabolic outcomes as compared to Biktarvy^®^. Adjusted analyses are pending to further explore these observations.

#### P151 Genetic contribution to weight gain after initiation of antiretroviral therapy in treatment‐naïve patients with HIV

J Berenguer ^1^, I Jarrin^2^, J Bellón^1^, C Díez^1^, M Jiménez‐Sousa^3^, R Navarro‐Soler^4^, J Iribarren^5^, I Suárez‐García^6^, C Amador^7^, A Curran^8^, F Villarroya^9^, P Domingo^10^, S Resino^3^

^1^Infectious Diseases, Hospital General Universitario Gregorio Marañón (IiSGM), Madrid, Spain; ^2^Centro Nacional de Epidemiología, Instituto de Salud Carlos III, Madrid, Spain; ^3^Centro Nacional de Microbiología, Instituto de Salud Carlos III, Majadahonda, Spain; ^4^HIV Unit ‐ Internal Medicine, Hospital Universitario 12 de Octubre (imas12), Madrid, Spain; ^5^Infectious Diseases, Hospital Universitario Donostia (b+odonostia), San Sebastián, Spain; ^6^Infectious Diseases ‐ Internal Medicine, Hospital Universitario Infanta Sofía, San Sebastíán de los Reyes, Spain; ^7^Infectious Diseases ‐ Internal Medicine, Hospital de la Marina Baixa, Villajoyosa, Spain; ^8^Infectious Diseases, Hospital Universitario Vall d'Hebron, Barcelona, Spain; ^9^Biochemistry and Molecular Biology, Universitat de Barcelona, Barcelona, Spain; ^10^Infectious Diseases, Hospital de la Santa Creu i Sant Pau, Barcelona, Spain

**Background**: To the best of our knowledge, the role of genetic factors in weight gain after the start of antiretroviral therapy (ART) has not been analysed. We studied the association of obesity‐related single nucleotide polymorphisms (SNPs) with weight gain after ART in people with HIV (PWHIV).

**Materials and methods**: Participants were ART‐naïve PWHIV, recruited in the Spanish HIV Research Cohort (CoRIS), who have started ART from 2014 onwards, who have weight and height information at baseline and 96 (±24 wk) from the beginning of ART, and who have blood/DNA deposited in the CoRIS Biobank. The primary outcome variable was a change in weight at 96 wk after the beginning of ART. Secondary outcomes included a BMI change and an increase of 10% in weight. We genotyped 14 obesity‐related SNPs from a meta‐analysis of GWS BMI loci in European people, including FTO and MC4R [1]. Genotyping was performed using the iPLEX^®^ Gold technology and Agena Bioscience's MassARRAY platform (San Diego, CA, USA). Changes over time in weight and BMI were studied using linear mixed models (LMM), considering SNPs, time, and their interaction as fixed effects and the patient as a random effect.

**Results**: A total of 1021 PWHIV met the study inclusion criteria. The mean weight gain over 96 wk was 2.90 (95% CI 2.54 to 3.26) kg. Factors associated with weight gain were female sex, birth in sub‐Saharan Africa, prior AIDS, CD4+ <200 cells/uL, HIV‐RNA >100 000 copies/mL, negative HCV serology, negative HBsAg, and use of TAF/FTC as the NRTI backbone. By adjusted LMM, a significant association was found between ZC3H4 rs3810291 and BCDIN3D/FAIM2 rs7138803 polymorphisms and weight and BMI increase (Table 1). Weight gain >10% was more frequent among PWHIV with ZC3H4 rs3810291 GG versus AA/AG polymorphisms: 37/163 (22.7%) versus 133/856 (15.5%), respectively, p = 0.029.

**Conclusions**: Our findings suggest that genetic factors play a role in weight gain after ART initiation. Further work is needed to understand the mechanisms by which the polymorphisms in/near ZC3H4 and BCDIN3D/FAIM2 lead to higher weight gain in this clinical context.

**Table:** 

**Abstract P151 – Table 1**. Results of linear mixed models adjusted by age, sex, country of birth, prior AIDS‐defining conditions, CD4+ cell count, HIV‐RNA viral load, HCV antibodies, HBsAg, type of ART regimen according to anchor drug, and NRTI backbone.				
Weight mean (SE), kg	Baseline	96 weeks	Increase	p
ZC3H4 rs3810291				0.007
GG	72.22 (70.43 to 74.02)	76.43 (74.47 to 78.40)	4.21 (3.12 to 5.30)	
AA/AG	72.71 (71.98 to 73.45)	75.35 (74.54 to 76.16)	2.64 (2.27 to 3.02)	
BCDIN3D/FAIM2 rs7138803				0.019
GG	72.11 (71.16 to 73.06)	75.43 (74.36 to 76.49)	3.32 (2.76 to 3.88)	
AG/AA	73.11 (72.13 to 74.08)	75.59 (74.54 to 76.65)	2.49 (2.02 to 2.95)	
**BMI mean (95% CI), kg/m^2^**	**Baseline**	**96 weeks**	**Increase**	**p**
ZC3H4 rs3810291				0.009
GG	24.01 (23.50 to 24.52)	25.40 (24.83 to 25.98)	1.39 (1.03 to 1.76 )	
AA/AG	24.20 (23.99 to 24.42)	25.08 (24.85 to 25.32)	0.88 (0.76 to 1.01)	
BCDIN3D/FAIM2 rs7138803				0.016
GG	24.07 (23.79 to 24.34)	25.18 (24.87 to 25.49)	1.11 (0.93 to 1.30)	
AG/AA	24.26 (23.98 to 24.55)	25.09 (24.78 to 25.40)	0.82 (0.67 to 0.98)	

BCDIN3D, BCDIN3 domain containing RNA methyltransferase; FAIM2, Fas apoptotic inhibitory molecule 2; ZC3H4, zinc finger CCCH‐type containing 4.

**Reference**

1. Locke AE, Kahali B, Berndt SI, Justice AE, Pers TH, Day FR, et al. Genetic studies of body mass index yield new insights for obesity biology. Nature. 2015;518:197‐206.

#### P152 Metabolic‐related outcomes after switching from tenofovir disoproxil fumarate to tenofovir alafenamide in adults living with HIV: a multicentre prospective cohort study

J Martínez‐Sanz^1^, S Serrano‐Villar^1^, A Muriel^2^, L García Fraile^3^, E Orviz^4^, Á Mena de Cea^5^, A Campins^6^, S Moreno Guillén ^1^

^1^Infectious Diseases, Hospital Universitario Ramón y Cajal, Madrid, Spain; ^2^Biostatistics, Hospital Universitario Ramón y Cajal, Madrid, Spain; ^3^Internal Medicine, Hospital Universitario de La Princesa, Madrid, Spain; ^4^Instituto de Investigación Sanitaria del Hospital, Centro Sanitario Sandoval, Madrid, Spain; ^5^Infectious Diseases, Complejo Hospitalario Universitario a Coruña, A Coruña, Spain; ^6^Internal Medicine, Hospital Universitario Son Espases, Palma, Spain

**Background**: Tenofovir alafenamide (TAF) is widely used to avoid the bone and kidney toxicity associated with tenofovir disoproxil fumarate (TDF) [1,2]. However, concerns remain about potential metabolic complications of TAF. We aimed to evaluate changes in weight, laboratory markers, and metabolic‐related clinical events after replacing TDF with TAF [3‐5].

**Figure:**
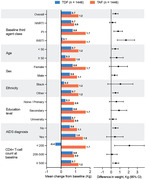
**Abstract P152 – Figure 1**. Overall and subgroup analysis for mean weight gain at 144 weeks. Mean change from baseline in each subgroup of participants in the propensity score‐matched cohort. The forest plot shows the mean difference in weight (95% CI) for each between‐group comparison. INSTI, integrase strand transfer inhibitor; PI, protease inhibitor.

**Table:** 

**Abstract P152 – Table 1**. Incidence of metabolic events during follow‐up.				
	TDF (n = 1446)	TAF (n = 1446)	OR (95% CI)	p
Diabetes, n (%)	20 (1.4)	20 (1.4)	1.00 (0.53 to 1.86)	1.000
Hypertension, n (%)	69 (4.8)	51 (3.5)	0.78 (0.54 to 1.13)	0.193
Use of lipid‐lowering therapy, n (%)	63 (4.4)	61 (4.2)	0.97 (0.67 to 1.38)	0.854
NAFLD, n (%)	84 (5.8)	76 (5.2)	0.90 (0.65 to 1.24)	0.515

NAFLD, non‐alcoholic fatty liver disease.

**Methods**: Multicentre prospective cohort study in the Spanish CoRIS cohort. We included virologically suppressed adults with HIV receiving TDF for more than 12 months who either switched to TAF or maintained TDF, with no changes in the core agent. Participants were matched by propensity score. We fitted generalised equation models to assess changes in weight, blood lipids, and hepatic steatosis index, and to compare the incidence of diabetes, hypertension, and lipid‐lowering drug use after 144 weeks.

**Results**: One thousand, four hundred and forty‐six participants were matched in each group. Median age was 38 years, 85% were male, mean weight at baseline was 73 kg. Participants who switched to TAF had a mean weight increase of +0.5 kg (95% CI 0.2 to 0.8, p = 0.001) at 144 weeks over those who maintained TDF (Figure 1), with no difference in the occurrence of overweight or obesity. Between‐group weight differences of TAF compared with TDF were larger among participants receiving INSTI, women, black ethnicity, and those with CD4+ <200 cells/μL. Participants who switched to TAF had a significantly higher increase in total cholesterol (+7.9 mg/dL), high‐density lipoprotein (HDL) cholesterol (+1.7 mg/dL), and triglycerides (+11.2 mg/dL) at 144 weeks. There were no differences in total cholesterol‐HDL ratio or hepatic steatosis index. During follow‐up, 20 individuals (1.4%) who switched to TAF were diagnosed with new‐onset diabetes, 51 (3.5%) with hypertension, 76 (5.2%) started a new lipid‐lowering agent, and 76 (5.2%) met NAFLD criteria. No significant differences were observed with participants who continued on TDF, of whom 1.4% developed diabetes, 4.8% developed hypertension, 4.4% used a new lipid‐lowering agent, and 5.8% met NAFLD criteria (Table 1).

**Conclusions**: Switching from TDF to TAF was associated with a 0.5 kg weight gain over 144 weeks with no difference in overweight or obesity or metabolic clinical events. LDL, HDL, and triglycerides increased with no difference in total cholesterol‐HDL ratio.

**References**

1. Saag MS, Gandhi RT, Hoy JF, Landovitz RJ, Thompson MA, Sax PE, et al. Antiretroviral drugs for treatment and prevention of HIV infection in adults: 2020 recommendations of the International Antiviral Society‐USA Panel. JAMA. 2020;324:1651‐69.

2. European AIDS Clinical Society Guidelines v11.0 [Internet]. 2021 [cited 2022 May 14]. Available from: http://www.eacsociety.org.

3. Surial B, Mugglin C, Calmy A, Cavassini M, Günthard HF, Stöckle M, et al. Weight and metabolic changes after switching from tenofovir disoproxil fumarate to tenofovir alafenamide in people living with HIV. Ann Intern Med. 2021;174:758‐67.

4. Mallon PWG, Brunet L, Hsu RK, Fusco JS, Mounzer KC, Prajapati G, et al. Weight gain before and after switch from TDF to TAF in a U.S. cohort study. J Int AIDS Soc. 2021;24:e25702.

5. Shah S, Pilkington V, Hill A. Is tenofovir disoproxil fumarate associated with weight loss? AIDS. 2021;35:S189‐95.

#### P153 Factors associated with weight loss or stable weight after continuing or switching to a doravirine‐based regimen

C Orkin ^1^, J Koethe^2^, P Kumar^3^, Z Xu^4^, R Plank^4^, W Greaves^4^, P Sklar^4^, R Lahoulou^5^

^1^HIV/AIDS Medicine, Queen Mary University of London, London, UK; ^2^Infectious Diseases, Vanderbilt University Medical Center, Nashville, TN, USA; ^3^Infectious Diseases and Travel Medicine, Medstar Georgetown University Hospital, Washington, DC, USA; ^4^Research, Merck & Co., Inc., Rahway, NJ, USA; ^5^Research, MSD, Puteaux, France

**Background**:  Minimal weight gain has been observed with doravirine (DOR)‐based regimens in first‐line and switch clinical trials. Characteristics of participants who maintained or lost weight (rather than gained) when continuing, or after switching to, a DOR‐based regimen were examined in three phase 3 trials.

**Materials and methods**: In DRIVE‐FORWARD (P018) and DRIVE‐AHEAD (P021), ART‐naïve adults were randomized to double‐blind treatment with a DOR regimen (DOR with two NRTIs or DOR/3TC/TDF) or the comparator regimen (darunavir/ritonavir with two NRTIs or EFV/FTC/TDF) for 96 weeks. After the double‐blind phase, eligible participants could continue DOR or switch to DOR in a 96‐week open‐label study extension. In DRIVE‐SHIFT (P024), adults who were virologically suppressed on a stable ART regimen were randomized to switch to DOR/3TC/TDF at day 1 or week 24 and continue through week 144. Weight loss was defined as percent change ≤‐5%, stable weight as change >‐5% to <5%, and weight gain as change ≥5%. Generalized logistic models were used to analyze factors associated with weight change category when continuing or after switching to DOR.

**Results**: Most participants who continued or switched to DOR had weight loss or stable weight: 11.8% and 65.5% of the P018+021 Continued group, 9.5% and 57.4% of the P018+021 Switch group, and 13.7% and 65.8% of the P024 Switch group, while weight gain occurred in 22.7%, 33.1%, and 20.5%, respectively. No clinical or demographic factors were associated with weight change from week 96 to 192 among participants who continued DOR in P018+021. Non‐black participants, particularly non‐black women, were more likely to have weight loss or maintain stable weight after switching to DOR (Table 1). Participants switched from NNRTIs were less likely to have weight loss in P024 and stable weight in P018+021 than those switched from PIs.

**Table:** 

**Abstract P153 – Table 1**. Factors impacting probability of weight change category after switch to doravirine.				
	Studies 018+021	Studies 018+021	Study 024	Study 024
Weight loss vs weight gain variable	OR (95% CI)	p‐value	OR (95% CI)	p‐value
Non‐Black vs Black	6.14 (1.53, 24.60)	0.010	2.37 (0.78, 7.17)	0.126
Non‐Black vs Black, Female	−		6.02 (1.15, 31.51)	0.033
Non‐Black vs Black, Male	−		0.93 (0.23, 3.81)	0.923
Female vs Male	4.76 (1.52, 14.87)	0.007	1.93 (0.60, 6.21)	0.272
Female vs Male, Non‐Black	−		4.89 (1.66, 14.39)	0.004
Female vs Male, Black	−		0.76 (0.11, 5.39)	0.782
Age <50 vs ≥50y	3.45 (0.71, 16.68)	0.124	0.80 (0.39, 1.63)	0.540
Baseline weight	1.06 (1.02, 1.10)	0.003	1.04 (1.01, 1.08)	0.009
Prior regimen: NNRTI vs PI	0.76 (0.36, 1.58)	0.460	0.41 (0.18, 0.95)	0.038
Time of switch: Day 1 vs Week 24	−		0.48 (0.25,0.94)	0.033
**Stable weight vs weight gain variable**	**OR (95% CI)**	**p‐value**	**OR (95% CI)**	**p‐value**
Non‐Black vs Black	2.15 (1.18, 3.91)	0.012	1.43 (0.67, 3.06)	0.359
Non‐Black vs Black, Female	−		2.47 (0.77, 7.85)	0.127
Non‐Black vs Black, Male	−		0.83 (0.31, 2.19)	0.703
Female vs Male	1.37 (0.67, 2.79)	0.383	1.01 (0.45, 2.27)	0.982
Female vs Male, Non‐Black	−		1.74 (0.73, 4.18)	0.214
Female vs Male, Black	−		0.58 (0.16, 2.13)	0.416
Age <50 vs ≥50y	1.14 (0.57, 2.30)	0.706	0.60 (0.37, 0.98)	0.043
Baseline weight	1.01 (0.99, 1.03)	0.471	1.03 (1.01, 1.06)	0.016
Prior regimen: NNRTI vs PI	0.60 (0.39, 0.92)	0.021	0.94 (0.58, 1.54)	0.815
Time of switch: Day 1 vs Week 24	−		0.72 (0.46, 1.11)	0.132

Odds ratios, 95% CIs and p‐values were from a generalized logistic model with the status of weight change (loss, stable and gain) as its outcome variable, and study ID, race, sex, age group, baseline BMI group and baseline weight as its explanatory variables. For P024, the model also included interaction of race and sex, categories of prior ART, and duration of prior ART as explanatory variables. The interaction term was not included in the model for P018‐021 as there were no black females in the weight loss category.

**Conclusions**: Switching to DOR resulted in weight loss or stable weight in most participants in three phase III trials, though weight change may differ by race, sex, and prior regimen. Because the proportions of women and black participants in the trials were low, further research is needed to better characterize the patient profile and mechanism for weight loss/stable weight with doravirine.

#### P154 Progression or regression of metabolic‐associated fatty liver disease in relation to INSTI/TAF exposure

J Milic^1^, S Renzetti^2^, F Motta^1^, J Bischoff^3^, A Dessilani^4^, J Conti^4^, F Medioli^4^, M Del Monte^4^, B Lebouche^5^, S Al Hinai^5^, M Deschenes^5^, S Calza^6^, C Mussini^1^, J Rockstroh^7^, G Guaraldi ^1^, G Sebastiani^5^

^1^Department of Surgical, Medical, Dental and Morphological Sciences, University of Modena and Reggio Emilia, Modena, Italy; ^2^Department of Medical‐Surgical Specialties, Radiological Sciences and Public Health, University of Brescia, Brescia, Italy; ^3^University of Bonn, Bonn, Germany; ^4^Department of Infectious Diseases, Azienda Ospedaliero‐Universitaria, Policlinico of Modena, Modena, Italy; ^5^McGill University, Montreal, Canada; ^6^Department of Molecular and Translational Medicine, University of Brescia, Brescia, Italy; ^7^Department of Medicine I, University of Bonn, Bonn, Germany

**Background**: Metabolic‐associated fatty liver disease (MAFLD) is a new construct that may describe metabolic health changes related to hepatic and extra‐hepatic clinical outcomes. The objective of the study was to estimate MAFLD progression or regression in PLWHIV in relation to exposure to integrase inhibitors (INSTI) with/without tenofovir alafenamide (TAF).

**Methods**: This was a longitudinal study of three large prospective cohorts of PLWHIV that were divided into four mutually exclusive groups: INSTI+/TAF+, INSTI+/TAF‐, INSTI‐/TAF+, INSTI‐/TAF‐. MAFLD was defined as the presence of hepatic steatosis (controlled attenuation parameter ≥248 dB/m assessed by transient elastography) and at least one criterion: 1) BMI ≥25 kg/m^2^; 2) diabetes; or 3) lean (BMI <25 kg/m^2^) with at least two immune‐metabolic alterations [1]. Significant liver fibrosis was defined as liver stiffness ≥7.1 kPa. A continuous‐time multi‐state Markov model was used to describe the process in which a study patient moved through a series of states allowing joint analysis of care length, incidence of MAFLD or its progression or reversion. The events were the transitions between the states. Minimum two and maximum six assessments for MAFLD were considered, 1 year apart.

**Results**: At baseline, 1339 PLWHIV were included, median age 52.7 years, 1032 (77%) males, median duration since HIV diagnosis was 18 years. Prevalence of MAFLD and liver fibrosis were 26.3% (352/1339) and 15.6% (169/1339), respectively. Prevalence of diabetes and obesity were 24.3% and 9%, respectively. At baseline, 100 (7.5%) and 452 (33.8%) PLWHIV were exposed to INSTI+/TAF+ and INSTI+/TAF‐. Figure 1 depicts alluvial plots of state transitions from no MAFLD to MAFLD across six patients’ visits. Table 1 shows factors associated with the risk of progression (from no MAFLD to MAFLD) or regression (from MAFLD to no MAFLD). In particular, age, weight and INSTI+/TAF+ regimens were associated with higher risk of MAFLD progression. Male sex was associated with reduced probability to progress to MAFLD but at the same time with reduced probability to revert it.

**Figure:**
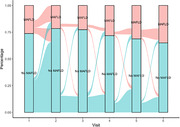
**Abstract P154 – Figure 1**. Depicts alluvial plots of state transitions from no MAFLD to MAFLD across 6 patients' visits.

**Conclusion**: This study explored dynamics of metabolic health changes in PLWHIV. Current INSTI+/TAF+ exposure only was associated with MAFLD progression, but this regimen was not an obstacle to its regression.

**Table:** 

**Abstract P154 – Table 1**. Factors associated with the risk of progression (from no MAFLD to MAFLD) or regression (from MAFLD to no MAFLD).		
Path	No MAFLD to MAFLD (progression)	MAFLD to no MAFLD (regression)
Age (continuous)	1.02 (1 to 1.04)	0.99 (0.97 to 1.02)
Male sex (vs female)	0.55 (0.32 to 0.95)	0.51 (0.31 to 0.85)
Years since HIV diagnosis (10 to 20 vs <10 years)	0.95 (0.53 to 1.70)	0.85 (0.47 to 1.56)
Years since HIV diagnosis (>20 years vs <10 years)	1.00 (0.56 to 1.80)	0.98 (0.54 to 1.79)
Nadir CD4 cell count <200 c/microL (vs >200)	0.84 (0.56 to 1.26)	0.97 (0.65 to 1.45)
Weight (continuous)	1.07 (1.05 to 1.10)	0.99 (0.97 to 1.02)
INSTI ‐ non TAF (vs non INSTI non TAF)	1.33 (0.82 to 2.14)	0.91 (0.56 to 1.46)
non INSTI ‐ TAF (vs non INSTI non TAF)	0.75 (0.38 to 1.48)	0.64 (0.34 to 1.22)
INSTI ‐ TAF (vs non INSTI non TAF)	2.11 (1.11 to 3.99)	0.90 (0.45 to 1.79)

**Reference**

1. Eslam M, Newsome PN, Sarin SK, Anstee QM, Targher G, Romero‐Gomez M, et al. A new definition for metabolic dysfunction‐associated fatty liver disease: An international expert consensus statement. J Hepatol. 2020;73:202‐9.

#### P155 Liver enzymes levels, metabolic and renal profile modifications after switching from TDF‐ to TAF‐based regimens among ART‐experienced PLWHIV in the ICONA cohort

M Poliseno^1^, S Lo Caputo^1^, A Tavelli ^2^, R Gagliardini^3^, L Gazzola^2^, A Saracino^4^, T Santantonio^1^, N Squillace^5^, M Puoti^6^, S Cicalini^3^, A Antinori^3^, A D'Arminio Monforte^2^, A Cozzi‐Lepri^7^

^1^Unit of Infectious Diseases, Foggia University Hospital, Foggia, Italy; ^2^Department of Health Sciences, University of Milan, Clinic of Infectious Diseases, San Paolo Hospital, Azienda Socio Sanitaria Territoriale (ASST) Santi Paolo e Carlo, Milan, Italy; ^3^Infectious Diseases Unit, National Institute for Infectious Diseases 'L. Spallanzani', Rome, Italy; ^4^Operative Unit of Infectious Diseases, Hospital‐University Polyclinic of Bari, Bari, Italy; ^5^Infectious Diseases Unit, Azienda Socio Sanitaria Territoriale (ASST) ‐ Monza, San Gerardo Hospital‐University of Milano‐Bicocca, Monza, Italy; ^6^Infectious Diseases Unit, Niguarda Hospital, Milan, Italy; ^7^Institute for Global Health, UCL, London, UK

**Background**: Alanine aminotransferase (ALT) elevation during treatment with tenofovir disoproxil fumarate (TDF) and their reduction after switching to tenofovir alafenamide (TAF) have been described among ART‐experienced PLWHIV [1,2]. However, the concomitant change of metabolic and renal markers and the possible role of the anchor drug in this condition remain unclear.

**Table:** 

**Abstract P155 – Table 1. ** Slopes from fitting a step‐linear mixed model with change at 1 year after the switch.						
	Pre‐switch		0 to 1 year after switch		>1 year after switch	
Laboratory parameter	Mean	p‐value^a^	Mean	p‐value^a^	Mean	p‐value^a^
Metabolic profile						
LDL Chol	3.5 (2.7 to 4.2)	<0.001	7.8 (6.1 to 9.5)	<0.001	‐16.2 (‐18.9 to 13.4)	<0.001
T‐Chol	4.2 (3.6 to 4.9)	<0.001	10.2 (8.7 to 11.8)	<0.001	‐21.1 (‐23.6 to ‐18.5)	<0.001
Triglycerides	1.4 (‐0.6 to 3.3)	0.163	10.5 (5.7 to 15.3)	<0.001	‐14.3 (‐22.1 to ‐6.6)	<0.001
T‐Chol/HDL Chol ratio	‐0.0 (‐0.1 to 0.0)	0.080	0.1 (0.0 to 0.2)	0.020	‐0.2 (‐0.3 to ‐0.1)	0.006
Glucose	‐0.3 (‐0.6 to 0.1)	0.176	2.5 (1.6 to 3.4)	<0.001	‐2.5 (‐3.9 to ‐1.0)	<0.001
Hepatic profile						
ALT	3.1 (‐0.0 to 6.1)	0.051	‐24.4 (‐32.5 to ‐16.3)	<0.001	30.3 (17.3 to 43.4)	<0.001
AST	1.5 (‐0.5 to 3.4)	0.146	‐12.7 (‐17.9 to ‐7.5)	<0.001	17.2 (8.8 to 25.7)	<0.001
GGT	‐1.3 (‐2.7 to 0.1)	0.070	‐4.6 (‐8.0 to ‐1.2)	0.008	5.9 (0.4 to 11.4)	0.035
ALP	‐2.6 (‐3.7 to ‐1.4)	<0.001	‐13.6 (‐16.3 to ‐10.9)	<0.001	22.9 (18.4 to 27.4)	<0.001
Renal profile						
Creatinine	‐0.0 (‐0.1 to 0.0)	0.101	0.1 (‐0.0 to 0.1)	0.172	‐0.1 (‐0.2 to 0.0)	0.185
Egfr	‐2.6 (‐2.8 to ‐2.4)	<0.001	2.3 (1.7 to 2.8)	<0.001	‐1.5 (‐2.4 to ‐0.6)	<0.001

ALP, alcaline phosphatase; ALT, alanine aminotransferase; AST, aspartate transaminase; eGFR, estimated glomerular filtration rate; GGT, gamma glutamyl transpeptidase; HDL Chol, high‐density lipoprotein cholesterol; LDL Chol, low‐density lipoprotein cholesterol; T‐Chol, total cholesterol.

^a^Wald test.

**Figure:**
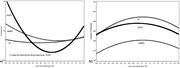
**Abstract P155 – Figure 1**. Predictions of ALT (a) and total cholesterol (b) changes after switching to TAF from fitting a quadratic mixed model, stratified by class of anchor drug used in the TAF‐based regimen. INSTI, integrase strand transfer inhibitors; NNRTI, non‐nucleosoidic reverse transcriptase inhibitors; PI, protease inhibitors.

**Materials and methods**: All undetectable, ART‐experienced, >18 years PLWHIV in the ICONA Foundation cohort switching from a TDF‐ to a TAF‐based regimen and having ≥2 ALT assessments before and after the switch were enrolled. Values were compared using a paired t‐test. Pearson rho was used to evaluate baseline correlations among the profiles. Mixed models with random intercept and slope with a change in slope at 1 year after switch were used to evaluate the trajectories of the markers. A quadratic model with interactions was used to assess the effect of the third drug used in the TAF regimen on ALT changes.

**Results**: Two thousand, nine hundred and eleven PLWHIV, 81% males, median (IQR) age 45 (37 to 53) years, were switched to a TAF‐based regimen after having received TDF for a median of 31 (19 to 47) months. At baseline, weak correlations were found between ALT and HDL cholesterol (ρ = ‐0.07, p < 0.0001), triglycerides (ρ = 0.13, p < 0.001), and glucose (ρ = 0.08, p < 0.001). Over a median of 42 (34 to 47) months follow‐up, a moderate decrease in ALT values (mean [±SD] 35.66 [±49.74] vs 34.28 [±49.74] U/L) was observed during the first year on TAF compared to when on TDF, followed by a slight increase (34.28 [±49.74] vs 35.66 [±99.15] U/L, p < 0.01). The opposite trend was reported for metabolic and renal profiles, although none of the changes was clinically significant (Table 1). U‐shaped trajectories of ALT (interaction p‐value = 0.04) and cholesterol from switch to 2 years according to third drug are shown in Figure 1.

**Conclusions**: After 42 months of follow‐up, no clinically significant changes were observed in hepatic and metabolic markers after the switch from TDF to TAF in our cohort. The anchor drug used appeared to slightly affect ALT changes. Further studies are warranted to define possible differences in liver markers in PLWHIV coinfected with HBV after switching from TDF to TAF.

**References**

1. Kovari H, Surial B, Tarr PE, Cavassini M, Calmy A, Schmid P; Swiss HIV Cohort Study. Changes in alanine aminotransferase levels after switching from tenofovir disoproxil fumarate (TDF) to tenofovir alafenamide (TAF) in HIV‐positive people without viral hepatitis in the Swiss HIV Cohort Study. HIV Med. 2021;22:623‐8.

2. Squillace N, Ricci E, Menzaghi B, De Socio GV, Passerini S, Martinelli C; CISAI Study Group. The effect of switching from tenofovir disoproxil fumarate (TDF) to tenofovir alafenamide (TAF) on liver enzymes, glucose, and lipid profile. Drug Des Devel Ther. 2020;14:5515‐20.

#### P156 Risk factors associated with 10% weight change in treatment‐naïve and treatment‐experienced people living with HIV initiating or switching to an NNRTI‐ or INSTI‐based antiretroviral therapy in four large cohort studies

O Robineau^1^, A Marongiu^2^, H Stellbrink^3^, J Meynard^4^, J Brunetta^5^, D Turner ^6^, A D'Arminio Monforte^7^, M Castaño‐Carracedo^8^, B van Welzen^9^, L Williams^2^, R Haubrich^10^, M Heinzkill^11^, S Sahali^12^, S Schreiber^11^, J Gruber^10^, C Cohen^10^, S Esser^13^

^1^Infectious Disease Department, University of Lille, Tourcoing, France; ^2^Real World Evidence, Gilead Sciences Ltd, Uxbridge, UK; ^3^Infektionsmedizinisches Centrum Hamburg Study Center, Hamburg, Germany; ^4^Saint Antoine Hospital, Assistance Publique des Hôpitaux de Paris, Paris, France; ^5^Maple Leaf Medical Clinic, Toronto, Canada; ^6^Sackler Faculty of Medicine, Crusaid Kobler AIDS Center, Tel‐Aviv Sourasky Medical Center, Tel‐Aviv University, Tel‐Aviv, Israel; ^7^Department of Health Sciences, Clinic of Infectious Diseases, University of Milan, Azienda Socio Sanitaria Territoriale, Santi Paolo e Carlo, Milan, Italy; ^8^Unidad de Enfermedades Infecciosas, Hospital Regional Universitario de Málaga, Málaga, Spain; ^9^Department of Internal Medicine and Infectious Diseases, University Medical Center Utrecht (UMCU), Utrecht, Netherlands; ^10^Medical Affairs, Gilead Sciences Global, Foster City, CA, USA; ^11^Medical Affairs, Gilead Sciences GmbH, Munich, Germany; ^12^Medical Affairs, Gilead Sciences, Boulogne‐Billancourt, France; ^13^Department of Venerology, Clinic of Dermatology, University Hospital Essen, Essen, Germany

**Background**: Pooled analyses of randomised controlled trials have shown weight gain (WG) following initiation/switching of antiretroviral therapy (ART) in people living with HIV (PLWHIV) [1,2]. We explored risk factors associated with ≥10% WG/weight loss (WL) in PLWHIV after initiating/switching ART.

**Materials and methods**: Weight data were analysed from treatment‐experienced (TE) and treatment‐naïve (TN) PLWHIV enrolled in four Gilead‐sponsored, post‐authorisation, observational HIV cohort studies (2010 to 2020) and who initiated/switched non‐nucleoside reverse transcriptase inhibitor (NNRTI)– or integrase strand transfer inhibitor (INSTI)–based ART. Participants had weight data available at baseline and 10 to 15 months post‐baseline. Adjusted odds ratios (ORs) for potential risk factors for ≥10% WG/WL at 12 months in participants were estimated using multivariable logistic regression.

**Results**: Two thousand, six hundred and sixty‐six participants were included. Median age: 38 (TN)/47 years (TE). Of TE participants, 914/1939 (47%) switched from emtricitabine (F)/tenofovir disoproxil fumarate (TDF) backbone to F/tenofovir alafenamide (TAF), and 162/1939 (8%) from abacavir (ABC)/lamivudine (3TC) to F/TAF. Two hundred and twenty‐nine of 727 (31%) TN participants had ≥10% WG (Table 1). Risk factors for ≥10% WG were F/TAF backbone (vs F/TDF; p < 0.001), low baseline CD4 (vs higher; p = 0.010), and being underweight (vs normal; p*=* 0.024; Figure 1). Eight of 727 (1%) TN participants had ≥10% WL. No significant risk factors for ≥10% WL were identified. Four hundred and fifty‐one of 1939 (23%) TE participants had ≥10% WG (Table 1). Risk factors for ≥10% WG were INSTI‐based ART (vs NNRTI; p = 0.029), F/TAF backbone (vs F/TDF; p < 0.001), being female (vs male; p = 0.002), switching from F/TDF to F/TAF (vs no switch; p = 0.001), being underweight (vs normal; p < 0.001), and having comorbidities associated with obesity (vs not; p = 0.046). Forty of 1939 (2%) TE participants had ≥10% WL. Only comedications associated with WL were predictors for ≥10% WL (OR 2.73 [95% CI 1.02 to 7.31]; p = 0.046).

**Figure:**
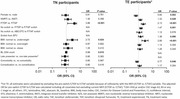
**Abstract P156 – Figure 1**. Risk factors associated with ≥10% weight gain in TN (n = 727) and TE (n = 1939) participants initiating/switching ART.

**Table:** 

**Abstract P156 – Table 1**. Baseline demographics/clinical characteristics of TN and TE participants by weight change.						
	TN (n = 727)			TE (n = 1939)		
Characteristic	Absolute weight loss^a^ (n = 153 [21%])	No weight change (n = 88 [12%])	Absolute weight gain^a^ (n = 486 [67%])	Absolute weight loss^a^ (n = 430 [22%])	No weight change (n = 225 [12%])	Absolute weight gain^a^ (n = 1284 [66%])
Male, n (%)	142 (93)	79 (90)	436 (90)	381 (89)	198 (88)	1072 (84)
Median (Q1, Q3) age, years	36.0 (29 to 45)	34.5 (29 to 46)	39.0 (31 to 46)	48.0 (38 to 55)	43.0 (36 to 51)	47.0 (38 to 54)
ART third agent: NNRTI, n (%)	60 (39)	38 (43)	120 (25)	144 (34)	125 (56)	440 (34)
ART third agent: INSTI, n (%)	93 (61)	50 (57)	366 (75)	286 (67)	100 (44)	844 (66)
ART backbone: F/TDF, n (%)	70 (46)	59 (67)	142 (29)	96 (22)	129 (57)	228 (18)
ART backbone: F/TAF, n (%)	83 (54)	29 (33)	344 (71)	334 (78)	96 (43)	1056 (82)
Pre‐switch efavirenz, n (%)	N/A	N/A	N/A	31 (5)	16 (5)	113 (6)
Switch: F/TDF to F/TAF ‐ no, n (%)	N/A	N/A	N/A	205 (48)	153 (68)	505 (39)
Switch: F/TDF to F/TAF ‐ yes, n (%)	N/A	N/A	N/A	185 (43)	69 (31)	660 (51)
Switch: ABC/3TC to F/TAF ‐ yes, n (%)	N/A	N/A	N/A	40 (9)	3 (1)	119 (9)
Median (Q1, Q3) baseline weight, kg	75.8 (69 to 85)	73.5 (64 to 84)	72.0 (65 to 82)	77.0 (70 to 88)	74.0 (66 to 84)	74.9 (66 to 84)
Median (Q1, Q3) baseline BMI, kg/m^2^	23.7 (21.8 to 25.6)	23.3 (20.9 to 25.2)	23.1 (21.4 to 25.5)	24.9 (22.7 to 27.8)	23.7 (21.6 to 25.6)	24.2 (21.8 to 26.9)
Median (Q1, Q3) weight change at 12 months, %	‐2.7 (‐5 to ‐1.4)	0 (0 to 0)	5.4 (2.8 to 9.5)	‐2.8 (‐5.2 to ‐1.4)	0 (0 to 0)	3.8 (1.8 to 6.8)
≥10% weight gain^b^ at 12 months, n (%)	N/A	N/A	229 (47)	N/A	N/A	451 (35)
≥10% weight loss^b^ at 12 months, n (%)	8 (5)	N/A	N/A	40 (9)	N/A	N/A
Any comorbidity, n (%)	13 (9)	9 (10)	63 (13)	144 (34)	44 (20)	377 (29)
Comedications associated with WG,^c^ n (%)	13 (9)	5 (6)	65 (13)	68 (16)	29 (13)	217 (17)
Comedications associated with WL,^d^ n (%)	2 (1)	1 (1)	26 (5)	24 (6)	8 (4)	59 (5)
Median (Q1, Q3) CD4 count, cells/μL	429 (309 to 582)	391 (302 to 511)	368 (221 to 534)	640 (458 to 840)	558 (383 to 754)	609 (406 to 823)
Median (Q1, Q3) HIV‐1 RNA, log10 copies/mL	4.35 (3.72 to 4.81)	4.48 (4.12 to 4.82)	4.64 (4.08 to 5.12)	1.59 (1.28 to 1.69)	1.69 (1.46 to 1.69)	1.57 (1.28 to 1.69)
Late presenters,^e^ n (%)	55 (36)	37 (42)	229 (47)	N/A	N/A	N/A

^a^Weight loss/gain includes any weight change;

^b^≥10% WG: yes = ≥10% WG, no = ≥–5% to <10% weight change; ≥10% WL: yes = ≥–10%, no = <–10% to <5% weight change;

^c^all individual comedications were classified as to whether they had an effect on WG, WL or none; drug classes of comedications with a known WG effect included: insulin, antidepressants, psychotropic agents, antiepileptics, antipsychotics, contraceptives, corticosteroids, antihistamines, beta‐androgenic blockers, anti‐gout preparation;

^d^all comedications were classified as to whether they had an effect on WG, WL or none; drug classes of comedications with a known WL effect included: antidepressants, antiepileptics, corticosteroids, beta‐androgenic blockers;

^e^CD4 <350 μL and/or CDC stage A3, B3 or any C stage (EU definition).

**Conclusions**: After initiating ART, WG was associated with F/TAF backbone, low baseline CD4 count, and being underweight in TN participants. In TE participants, WG was associated with INSTI‐based ART, F/TAF backbone, switching from F/TDF to F/TAF, being female, and being underweight at baseline. Findings are consistent with the documented WG suppressive effect of F/TDF [3‐6]. These data cannot determine the contribution of F/TAF to WG beyond the absence of the F/TDF WG suppressive effect.

**References**

1. Sax PE, Erlandson KM, Lake JE, Mccomsey GA, Orkin C, Esser S, et al. Weight gain following initiation of antiretroviral therapy: risk factors in randomized comparative clinical trials. Clin Infect Dis. 2020;71:1379‐89.

2. Erlandson KM, Carter CC, Melbourne K, Brown TT, Cohen C, Das M, et al. Weight change following antiretroviral therapy switch in people with viral suppression: pooled data from randomized clinical trials. Clin Infect Dis. 2021;73:1440‐51.

3. Glidden DV, Mulligan K, McMahan V, Anderson PL, Guanira J, Chariyalertsak S, et al. Metabolic effects of preexposure prophylaxis with coformulated tenofovir disoproxil fumarate and emtricitabine. Clin Infect Dis. 2018;67:411‐9.

4. Ogbuagu O, Ruane PJ, Podzamczer D, Salazar LC, Henry K, Asmuth DM, et al. Long‐term safety and efficacy of emtricitabine and tenofovir alafenamide vs emtricitabine and tenofovir disoproxil fumarate for HIV‐1 pre‐exposure prophylaxis: week 96 results from a randomised, double‐blind, placebo‐controlled, phase 3 trial. Lancet HIV. 2021;8:e397‐e407.

5. Landovitz RJ, Donnell D, Clement M, Hanscom B, Cottle L, Coelho L, et al. HPTN 083 final results: Pre‐exposure prophylaxis containing long‐acting injectable cabotegravir is safe and highly effective for cisgender men and transgender women who have sex with men. 23rd International AIDS Conference; 2020 Jul 6‐10. Oral OAXLB01.

6. Shah S, Pilkington V, Hill A. Use of tenofovir disoproxil fumarate shows weight loss vs placebo: a meta‐analysis of 7 clinical trials in 19,359 HIV‐negative individuals. IDWeek 2021; 2021 Sep 29‐Oct 3; virtual. Poster P882.

**Table:** 

**Abstract P157 – Table 1. **Univariate and multivariate logistic regression analyses of factors associated with obesity and diabetes mellitus in a cohort of people of black ethnicity living with HIV (n=398).								
	Obesity (univariate analysis)		Obesity (multivariate analysis)		DM (univariate analysis)		DM (multivariate analysis)	
	OR [95% CI]	p‐value	aOR [95% CI]	p‐value	OR [95% CI]	p‐value	aOR [95% CI]	p‐value
Male sex	0.35 [0.23 to 0.52]	<0.001	0.31 [0.19 to 0.51]	<0.001	1.60 [0.96 to 2.65]	0.07	1.94 [1.04 to 3.59]	0.04
Age 40 to 50 years old	2.21 [1.08 to 4.54]	0.03	1.89 [0.83 to 4.33]	0.13	1.28 [0.40 to 4.09]	0.68	1.00 [0.30 to 3.34]	1.00
Age >50 years old	2.39 [1.20 to 4.75]	0.01	2.17 [0.98 to 4.83]	0.06	3.37 [1.16 to 9.85]	0.03	2.51 [0.83 to 7.58]	0.10
Born in Africa/Caribbean	1.25 [0.75 to 2.08]	0.39	−	−	1.81 [0.86 to 3.83]	0.12	−	−
BMI (kg/m^2^)	N/A	N/A	N/A	N/A	1.04 [1.00 to 1.08]	0.05	1.05 [1.01 to 1.10]	0.03
Diabetes	2.22 [1.28 to 3.84]	0.004	2.33 [1.21 to 4.48]	0.01	N/A	N/A	N/A	N/A
HIV VL ≥200 copies/mL	0.42 [0.17 to 1.05]	0.06	0.35 [0.12 to 1.02]	0.05	1.98 [0.78 to 4.99]	0.15	−	−
CRP (mg/L)	1.16 [1.09 to 1.24]	<0.001	1.13 [1.06 to 1.21]	<0.001	1.06 [1.01 to 1.12]	0.01	1.06 [1.01 to 1.12]	0.02
Impaired function	1.62 [1.02 to 2.59]	0.04	1.42 [0.79 to 2.55]	0.25	1.52 [0.87 to 2.66]	0.14	−	−
Social isolation	1.24 [0.81 to 1.89]	0.32	−	−	0.83 [0.48 to 1.44]	0.52	−	−
Non‐disclosure of HIV	0.80 [0.50 to 1.28]	0.35	−	−	1.48 [0.84 to 2.62]	0.18	−	−
Discrimination	1.16 [0.75 to 1.78]	0.51	−	−	0.83 [0.47 to 1.46]	0.51	−	−
Anxiety	1.81 [1.08 to 3.03]	0.03	1.49 [0.79 to 2.82]	0.22	0.62 [0.31 to 1.25]	0.18	−	−
Depression	1.48 [0.81 to 2.71]	0.20	−	−	1.07 [0.50 to 2.29]	0.86	−	−
Financial insecurity	1.39 [0.93 to 2.06]	0.11	−	−	1.13 [0.68 to 1.87]	0.65	−	−
Food insecurity	1.08 [0.66 to 1.76]	0.75	−	−	1.77 [0.99 to 3.14]	0.05	2.28 [1.23 to 4.23]	0.009
Housing insecurity	0.74 [0.39 to 1.39]	0.35	−	−	1.11 [0.51 to 2.42]	0.79	−	−
Migration insecurity	0.83 [0.45 to 1.52]	0.54	−	−	1.17 [0.55 to 2.46]	0.69	−	−
Employment insecurity	1.27 [0.82 to 1.96]	0.29	−	−	1.24 [0.72 to 2.13]	0.44	−	−
Low educational status	1.30 [0.85 to 1.99]	0.23	−	−	0.63 [0.35 to 1.12]	0.12	−	−

Impaired function = moderate/severe/extreme difficulty in mobility, self‐care, or doing usual activities. Social isolation = loneliness/isolation (often feeling a lack of companionship, feeling left out from others, feeling isolated from others, or feeling lonely), no one to turn to for emotional support, or needed support with loneliness/isolation in the last year. Financial insecurity = behind with some/most bills, only some of the time enough money to meet basic needs, or needed financial support in the last year. Food insecurity = needed food support in the last year. Housing insecurity = temporary accommodation/living with friends or family/homeless. Migration status insecurity = temporary/limited/no status, or needed immigration support in the last year. Employment insecurity = unemployed, sick, or disabled, or needed employment support in the last year. Low educational status = O levels (age 16) or less.

#### P157 Socio‐economic deprivation, obesity, and diabetes mellitus in people of African ancestry living with HIV in South London

L Domínguez‐Domínguez ^1^, S Tariq^2^, L Cechin^1^, L Hamzah^3^, J Fox^4^, V Kolodin^1^, H Lempp^1^, L Goff^1^, D Onyango^5^, L Campbell^1^, F Post^1^

^1^HIV & Sexual Health, King's College London, King's College Hospital NHS Foundation Trust, London, UK; ^2^Centre for Clinical Research in Infection and Sexual Health Institute for Global Health, University College London, London, UK; ^3^HIV and Sexual Health, St George's Hospital NHS Foundation Trust, London, UK; ^4^HIV & Sexual Health, King's College London, Guy's and St Thomas' NHS Foundation Trust, London, UK; ^5^Africa Advocacy Foundation, London, UK

**Background**: Obesity, diabetes mellitus (DM), HIV and socio‐economic deprivation are highly prevalent in communities of African ancestry in South London. We explored associations between measures of socio‐economic deprivation and (i) obesity and (ii) DM in this population.

**Methods**: A cross‐sectional study of people of black ethnicity living with HIV aged 30 to 65 years who were enrolled between September 2020 and January 2022. Demographic, clinical and HIV data were collected, and height, weight, C‐reactive protein and HbA1c measured. Functional status, degree of social isolation and disclosure of HIV status, discrimination, mental health (anxiety and depression [HADS score >10]) and socio‐economic status were evaluated with validated questionnaires. We used logistic regression to explore associations between socio‐economic variables and obesity (body mass index [BMI] >30 kg/m^2^) or DM (HbA1c ≥6.5% or use of diabetic medications).

**Results**: We enrolled 398 participants (54.8% female; median age 52 [IQR 45 to 57] years; 72% born in sub‐Saharan Africa/9% in the Caribbean; median CD4 count 548 [372 to 749] cells/mm^3^; 94% HIV viral load <200 copies/mL). Obesity was present in 49.7%, DM in 18.8%, anxiety in 20.4% and depression in 13.3%. Socio‐economic deprivation was common: 52.3% reported financial insecurity, 22.3% food insecurity, 11.1% housing insecurity, 12.3% migration status insecurity, 28.4% employment insecurity, and 32.0% had low educational status. In multivariable analysis with adjustment for sex, age, DM, HIV viral load, C‐reactive protein, functional status and mental health, measures of socio‐economic deprivation were not associated with obesity (p > 0.1). A separate and similarly adjusted model revealed a significant association between food insecurity and DM (aOR 2.28, 95% CI 1.23 to 4.23) (Table 1).

**Conclusions**: We found no evidence of an association between various socio‐economic factors and obesity in people living with HIV of African ancestry.  However, food insecurity was associated with DM, highlighting the importance of ascertaining and addressing food security in this population, especially in the context of rising costs of living.

**Table:** 

**Abstract P158 – Table 1**. Difference in change from baseline in metabolic, liver and quality of life parameters for the maraviroc + OBT versus OBT group with bootstrapped 95% confidence intervals.				
Measure	Week	Number of participants	Difference between groups in change from baseline	95% confidence interval
BMI, kg/m^2^	48	45	‐0.2	‐1.0 to 0.6
	96	45	‐0.2	‐0.8 to 0.4
Waist circumference, cm	48	43	2.7	‐5.1 to 11
	96	42	1.3	‐7.3 to 9.9
CD4 cell count, cells/mm^3^	48	45	70	‐35 to 175
	96	44	‐47	‐135 to 41
Fasting glucose, mmol/L	48	44	‐0.1	‐0.8 to 0.7
	96	42	0.8	‐0.4 to 2.1
HbA1c, mmol/mol	48	45	‐2.3	‐6.6 to 2.1
	96	45	2.5	‐3.0 to 8.0
AST, U/L	48	43	‐0.5	‐7.2 to 6.1
	96	38	7.0	‐3.4 to 17
ALT, U/L	48	44	‐5.7	‐22 to 10
	96	45	0.5	‐19 to 20
Fasting triglycerides, mmol/L	48	44	0.7	‐0.1 to 1.4
	96	46	‐0.2	‐1.0 to 0.6
Fasting LDL, mmol/L	48	40	0	‐0.5 to 0.4
	96	41	0	‐0.4 to 0.5
Fasting HDL, mmol/L	48	44	0	‐0.2 to 0.2
	96	46	0.1	‐0.1 to 0.3
Fasting HDL: total cholesterol ratio	48	44	0.3	‐0.6 to 1.1
	96	46	‐0.3	‐1.0 to 0.4
Fasting total cholesterol, mmol/L	48	44	0.1	‐0.4 to 0.6
	96	46	0.1	‐0.4 to 0.7
Fibroscan median liver stiffness, kPa	48	43	‐1.7	‐3.8 to 0.4
	96	41	‐0.2	‐2.0 to 1.6
Fibroscan CAP score, dB/m	48	41	‐15	‐56 to 25
	96	39	‐5.8	‐41 to 30
ELF score	48	43	0.3	0 to 0.6
	96	42	0.1	‐0.3 to 0.4
Chronic Liver Disease Questionnaire for NAFLD	48	45	‐0.3	‐1.0 to 0.4
	96	44	‐0.5	‐1.3 to 0.2

#### P158 Results of Hepmarc: a 96‐week feasibility trial of add‐on maraviroc in people with well‐controlled HIV and NAFLD

D Bradshaw ^1^, I Abramowicz^2^, S Bremner^2^, S Verma^3^, Y Gilleece^1^, S Kirk^1^, M Nelson^4^, R Housman^4^, H Miras^5^, C Orkin^5^, A Fox^6^, M Curnock^6^, L Jennings^7^, M Gompels^7^, E Clarke^8^, R Robinson^8^, P Lambert^9^, D Chadwick^9^, N Perry^2^

^1^HIV, University Hospitals Sussex NHS Foundation Trust, Brighton, UK; ^2^Brighton & Sussex Clinical Trials Unit, Brighton and Sussex Medical School, Brighton, UK; ^3^Hepatology, Brighton and Sussex Medical School, Brighton, UK; ^4^HIV, Chelsea and Westminster Hospital NHS Foundation Trust, London, UK; ^5^HIV, Barts Health NHS Trust, London, UK; ^6^HIV, Nottingham University Hospitals NHS Trust, Nottingham, UK; ^7^HIV, North Bristol NHS Trust, Bristol, UK; ^8^HIV, Liverpool University Hospitals NHS Foundation Trust, Liverpool, UK; ^9^Infectious Diseases, South Tees Hospitals NHS Foundation Trust, Middlesbrough, UK

**Background**: Non‐alcoholic fatty liver disease (NAFLD) is common in people with HIV‐1. Maraviroc may reduce hepatic inflammation through CCR5‐receptor antagonism, which warrants further exploration.

**Materials and methods**: A multicentre, open‐label 96‐week randomised controlled feasibility trial of maraviroc + optimised background therapy (OBT) versus OBT alone, in a 1:1 ratio, for people with virologically suppressed HIV‐1 and NAFLD on imaging or biopsy, without cirrhosis. Dosing followed SmPC recommendations for HIV therapy. Primary outcomes were safety, recruitment and retention rates, adherence and data completeness. Secondary outcomes included Fibroscan‐assessed liver stiffness measurements (LSM), controlled attenuation parameter (CAP) scores, and Enhanced Liver Fibrosis (ELF) scores. A sample size of n = 20 per group would allow estimation of a difference in ELF score of 1 point with a 95% confidence interval (CI) of 0.6 to 1.4, assuming a standard deviation (SD) of 1.12. Assuming 33% attrition, this gave a target of n = 30 per group.

**Results**: Fifty‐three participants recruited, of whom 23 received MVC+OBT: 89% male, 89% white, median age 54 (IQR 47 to 60) years; 19% with type 2 diabetes mellitus. Median baseline LSM, CAP & ELF scores were 6.2 (IQR 4.6 to 7.8) kPa, 325 (IQR 279 to 351) dB/m and 9.1 (IQR 8.6 to 9.6) respectively. Primary outcomes: all individuals eligible after screening were randomised; 92% (SD 6.6%) adherence to maraviroc [target >90%]; 83% (95% CI 70% to 92%) participant retention [target >65%]; 5.5% of data were missing at week 96 [target <20%]. No serious adverse reactions (SARs); mild‐moderate intensity ARs were experienced by five participants (5/23, 22% (95% CI 5% to 49%)) [target <10%], including two (2/23, 8.7%) who subsequently discontinued maraviroc. All ARs resolved. One additional participant (1/23, 4.3%) discontinued maraviroc due to disruption to daily routine. Secondary outcomes: overall, median LSM, CAP and ELF scores remained stable over 96 weeks. No significant differences were seen by treatment group for change from baseline in LSM, CAP, ELF, CD4 count, glucose, lipid profile, liver function, weight, waist circumference or quality of life parameters (see Table 1).

**Table:** 

**Abstract P158 – Table 1**. Difference in change from baseline in metabolic, liver and quality of life parameters for the maraviroc + OBT versus OBT group with bootstrapped 95% confidence intervals.				
Measure	Week	Number of participants	Difference between groups in change from baseline	95% confidence interval
BMI, kg/m2	48	45	−0.2	−1.0 to 0.6
	96	45	−0.2	−0.8 to 0.4
Waist circumference, cm	48	43	2.7	−5.1 to 11
	96	42	1.3	−7.3 to 9.9
CD4 cell count, cells/mm^3^	48	45	70	−35 to 175
	96	44	−47	−135 to 41
Fasting glucose, mmol/L	48	44	−0.1	−0.8 to 0.7
	96	42	0.8	−0.4 to 2.1
HbA1c, mmol/mol	48	45	−2.3	−6.6 to 2.1
	96	45	2.5	−3.0 to 8.0
AST, U/L	48	43	−0.5	−7.2 to 6.1
	96	38	7.0	−3.4 to 17
ALT, U/L	48	44	−5.7	−22 to 10
	96	45	0.5	−19 to 20
Fasting triglycerides, mmol/L	48	44	0.7	−0.1 to 1.4
	96	46	−0.2	−1.0 to 0.6
Fasting LDL, mmol/L	48	40	0	−0.5 to 0.4
	96	41	0	−0.4 to 0.5
Fasting HDL, mmol/L	48	44	0	−0.2 to 0.2
	96	46	0.1	−0.1 to 0.3
Fasting HDL: total cholesterol ratio	48	44	0.3	−0.6 to 1.1
	96	46	−0.3	−1.0 to 0.4
Fasting total cholesterol, mmol/L	48	44	0.1	−0.4 to 0.6
	96	46	0.1	−0.4 to 0.7
Fibroscan median liver stiffness, kPa	48	43	−1.7	−3.8 to 0.4
	96	41	−0.2	−2.0 to 1.6
Fibroscan CAP score, dB/m	48	41	−15	−56 to 25
	96	39	−5.8	−41 to 30
ELF score	48	43	0.3	0 to 0.6
	96	42	0.1	−0.3 to 0.4
Chronic Liver Disease Questionnaire for NAFLD	48	45	−0.3	−1.0 to 0.4
	96	44	−0.5	−1.3 to 0.2

**Conclusions**: This feasibility study provides preliminary evidence of maraviroc safety amongst people with HIV/NAFLD who do not have cirrhosis, and acceptable recruitment, retention, and adherence rates. These data support a definitive RCT assessing maraviroc impact on hepatic steatosis and fibrosis.

#### P159 HIV‐associated wasting in the era of weight gain

M Wohlfeiler ^1^, R Weber^2^, L Brunet^2^, J Siddiqui^3^, M Harbour^4^, A Phillips^4^, B Hayward^4^, J Fusco^2^, R Hsu^5^, G Fusco^2^

^1^Medicine, AIDS Healthcare Foundation, Miami, FL, USA; ^2^Sciences/Epidemiology, Epividian, Inc., Durham, NC, USA; ^3^Medicine, TeleMed2U, Roseville, CA, USA; ^4^Medical Affairs, EMD Serono Inc., Rockland, MA, USA, an affiliate of Merck KGaA, Rockland, MA, USA; ^5^Medicine, AIDS Healthcare Foundation, New York, NY, USA

**Background**: Recently, attention has been focused on weight gain with antiretroviral therapy (ART) [1]. However, HIV‐associated wasting (i.e. progressive, involuntary weight loss with both fat and lean tissue loss; HIVAW) remained prevalent in two US claims studies (2005 to 2007: 8%, 2012 to 2018: 18%). We aimed to assess the prevalence and incidence of HIVAW/underweight in the US.

**Materials and methods**: People with HIV (PWHIV) in care in 2012 to 2020 were identified in the Observational Pharmaco‐Epidemiology Research & Analysis (OPERA^®^) cohort. HIVAW/underweight included a wasting or low BMI/underweight diagnosis (ICD codes, title search) or BMI <20 kg/m^2^. Prevalence was assessed between 2012 to 2015 and 2016 to 2020. Incidence rate in 2016 to 2020 was assessed using univariate Poisson regression among those without prior HIVAW/underweight.

**Results**: The prevalence of HIVAW/underweight was 12% in both 2012 to 2015 (7176 of 59 026 PWHIV) and 2016 to 2020 (11 995 of 101 026 PWHIV); low BMI/underweight accounted for most cases (Figure 1). Among 67 119 PWHIV (225 215 person‐years) without prior HIVAW/underweight in 2016 to 2020, 4962 incident cases were identified (incidence rate: 2.20 per 100 person‐years; 95% CI 2.14 to 2.27). PWHIV with incident wasting were more likely to be Black, less likely to be Hispanic or to have ever taken ART (specifically tenofovir alafenamide), and experienced longer delays between HIV diagnosis and ART initiation than PWHIV without wasting (Table 1).

**Table:** 

**Abstract P159 – Table 1**. Demographic and clinical characteristics at index^a^ in 2016 to 2020.		
	Incident HIVAW/ underweight^b^ N = 4962	No incident HIVAW/ underweight^b^ N = 62 157
Median age (IQR), years	40 (28 to 53)	41 (31 to 52)
Female, n (%)	926 (19)	11 389 (18)
Black race, n (%)	2559 (52)	28 655 (46)
Hispanic ethnicity, n (%)	805 (16)	13 708 (22)
Ever on ART, on or prior to first active visit, n (%)	2937 (59)	44 094 (71)
Ever on TAF	715 (24)	12 437 (28)
Median years from HIV diagnosis to ART initiation (IQR)	3.5 (0.1 to 12.4)	2.9 (0.1 to 10.6)

HIVAW, HIV‐associated wasting; IQR, interquartile range; TAF, tenofovir alafenamide.

^a^Index: first eligible visit in 2016 to 2020;

^b^wasting diagnosis (ICD codes or title search), low BMI or underweight diagnosis (ICD codes, title search) or BMI <20 kg/m^2^ (vitals).

**Figure:**
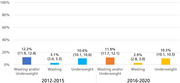
**Abstract P159 – Figure 1**. Prevalence of HIVAW/underweight^a^ in the era of weight gain [1]. ^a^Wasting diagnosis (ICD codes or title search), low BMI or underweight diagnosis (ICD codes, title search) or BMI <20 kg/m^2^ (vitals).

**Conclusions**: Wasting remains a challenge for PWHIV and may be underappreciated by providers based on the large proportion of underweight PWHIV without a diagnosis of wasting. Increasing awareness of HIVAW could improve the care of affected PWHIV in an era of weight gain associated with ART.

**Reference**

1. Sax PE, Erlandson KM, Lake JE, Mccomsey GA, Orkin C, Esser S, et al. Weight gain following initiation of antiretroviral therapy: risk factors in randomized comparative clinical trials. Clin Infect Dis. 2020;71:1379‐89.

#### P160 Comparison of HIV‐associated wasting definitions in the OPERA cohort

M Wohlfeiler ^1^, R Weber^2^, L Brunet^2^, J Siddiqui^3^, M Harbour^4^, A Phillips^4^, B Hayward^4^, J Fusco^2^, R Hsu^5^, G Fusco^2^

^1^Medicine, AIDS Healthcare Foundation, Miami, FL, USA; ^2^Sciences/Epidemiology, Epividian, Inc., Durham, NC, USA; ^3^Medicine, TeleMed2U, Roseville, CA, USA; ^4^Medical Affairs, EMD Serono Inc., an affiliate of Merck KGaA, Rockland, MA, USA; ^5^Medicine, AIDS Healthcare Foundation, New York, NY, USA

**Background**: HIV‐associated wasting (i.e. progressive, involuntary loss of fat and lean muscle tissue; HIVAW) among people with HIV (PWHIV) remains a concern despite effective antiretroviral therapy (ART). Multiple criteria are often considered to define HIVAW in observational research, though there is no consensus. We aimed to compare two approaches to identify HIVAW in electronic health records from the United States.

**Materials and methods**: ART‐experienced, adult PWHIV in care in 2016 to 2020 with no history of HIVAW were followed through 31 October 2021 in the Observational Pharmaco‐Epidemiology Research & Analysis (OPERA^®^) cohort. HIVAW definition 1 consisted of a new wasting or low BMI/underweight diagnosis (ICD codes, title search) or a BMI measurement <20 kg/m^2^. Definition 2 included the same diagnoses, two consecutive BMI measurements <18.5 kg/m^2^, or loss of ≥10% of baseline body weight within 12 months. Baseline characteristics were selected for inclusion in a multivariable logistic regression model based on scientific literature and expert opinion.

**Results**: Of 52 087 ART‐experienced PWHIV in OPERA^®^, 6% and 7% experienced incident HIVAW over follow‐up using definition 1 (n = 3343) and definition 2 (n = 3479), respectively. With both definitions, odds of HIVAW were lower with Black race, Hispanic ethnicity, and tenofovir alafenamide use (ever), but higher with Medicaid and protease inhibitors. There was a dose‐response relationship with increasing VACS scores associated with higher odds of HIVAW, though slightly attenuated for definition 2. Female sex was associated with a 17% decrease in odds (95% CI 0.73 to 0.93) using definition 1 but an 11% increase in odds (95% CI 1.00 to 1.23) using definition 2 (Table 1).

**Conclusions**: Despite consistency in the proportions of PWHIV experiencing HIVAW and most predictors between HIVAW definitions, there are differences to consider. Because HIVAW is not limited to underweight bodies, definition 1 could potentially overrepresent younger PWHIV, who are naturally thinner, and underrepresent females who tend to have higher BMI. Definition 2 allows for PWHIV of all body sizes to be identified as potentially experiencing HIVAW, highlighting unusual weight loss over time. In the absence of a standard definition, it is important to consider multiple avenues through which HIVAW may be identified in observational research.

**Table:** 

**Abstract P160 – Table 1**. Predictors of incident HIVAW among ART‐experienced people with HIV in OPERA^®^ using two definitions^a,b^ of HIVAW.						
Predictor at baseline^c^	Definition 1: N = 2306 with HIVAW	Definition 1: N = 36 860 without HIVAW	Definition 1: aOR^d^ (95% CI)	Definition 2: N = 2551 with HIVAW	Definition 2: N = 36 632 without HIVAW	Definition 2: aOR^d^ (95% CI)
Age, years						
18 to <40	947 (41)	15 414 (42)	Reference	913 (36)	15 459 (42)	Reference
40 to <55	790 (34)	14 560 (40)	0.76 (0.68 to 0.84)	1021 (40)	14 333 (39)	1.04 (0.94 to 1.14)
≥55	569 (25)	6886 (19)	0.92 (0.81 to 1.05)	617 (24)	6840 (19)	1.12 (0.99 to 1.27)
Female sex	443 (19)	6823 (19)	0.83 (0.73 to 0.93)	583 (23)	6683 (18)	1.11 (1.00 to 1.23)
Black race	1154 (50)	16 926 (46)	0.98 (0.89 to 1.08)	1240 (49)	16 846 (46)	0.93 (0.85 to 1.02)
Hispanic ethnicity	413 (18)	8513 (23)	0.78 (0.69 to 0.88)	470 (18)	8457 (23)	0.79 (0.71 to 0.89)
Medicaid	757 (33)	10 039 (27)	1.22 (1.11 to 1.34)	873 (34)	9924 (27)	1.25 (1.15 to 1.37)
History of AIDS	579 (25)	8341 (23)	0.96 (0.86 to 1.07)	661 (26)	8255 (23)	0.96 (0.87 to 1.07)
VACS Mortality Index^e^						
0 to <15	960 (42)	19 713 (53)	Reference	1126 (44)	19 561 (53)	Reference
15 to <30	705 (31)	10 890 (30)	1.36 (1.22 to 1.53)	760 (30)	10 838 (30)	1.12 (1.00 to 1.25)
30 to <45	343 (15)	3768 (10)	1.88 (1.61 to 2.20)	335 (13)	3776 (10)	1.38 (1.18 to 1.60)
≥45	298 (13)	2489 (7)	2.41 (2.00 to 2.90)	330 (13)	2457 (7)	2.04 (1.71 to 2.43)
CD4 cell count^e^, cells/μL						
≥500	1316 (57)	23 104 (63)	Reference	1525 (60)	22 907 (63)	Reference
200 to <500	701 (30)	10 824 (29)	0.95 (0.86 to 1.06)	735 (29)	10 795 (29)	0.93 (0.84 to 1.03)
0 to <200	289 (13)	2932 (8)	1.07 (0.90 to 1.27)	291 (11)	2930 (8)	1.02 (0.87 to 1.21)
Calendar year ART initiation						
≤2015	1373 (60)	21 291 (58)	Reference	1591 (62)	21 069 (58)	Reference
2016 to 2020	933 (40)	15 569 (42)	1.05 (0.94 to 1.18)	960 (38)	15 563 (42)	1.01 (0.91 to 1.12)
Ever used INSTI	1398 (61)	22 859 (62)	0.97 (0.88 to 1.06)	1520 (60)	22 748 (62)	0.98 (0.90 to 1.07)
Ever used PI	825 (36)	11 445 (31)	1.10 (1.00 to 1.21)	956 (37)	11 314 (31)	1.13 (1.03 to 1.23)
Ever used TAF	549 (24)	10 118 (27)	0.81 (0.72 to 0.92)	550 (22)	10 134 (28)	0.78 (0.69 to 0.88)

aOR, adjusted odds ratio; HIVAW, HIV‐associated wasting; INSTI, integrase strand transfer inhibitor; PI, protease inhibitor; PWHIV, people with HIV; TAF, tenofovir alafenamide; VACS, Veterans Aging Cohort Study.

^a^Wasting diagnosis (ICD text or code), low BMI/underweight diagnosis (ICD text or code), or BMI measurement <20 kg/m^2^;

^b^wasting diagnosis (ICD text or code), low BMI/underweight diagnosis (ICD text or code), or two consecutive BMI measurements <18.5 kg/m^2^, or loss of ≥10% of baseline body weight within 12 months after baseline;

^c^baseline = first date in the study period (2016 to 2020) where the PWHIV was HIV+, 18 years of age or older, and had an active OPERA^®^ visit;

^d^adjusted for all variables in the table;

^e^value closest to baseline: on or any time within 15 months prior to baseline if available; otherwise, up to 6 months after baseline (prior to censoring date).

#### P161 Metabolic effects of switching to tenofovir alafenamide/emtricitabine/bictegravir (B/F/TAF) from tenofovir difumarate (TDF) or tenofovir alafenamide (TAF) sparing regimens: METABIC study

C Busca^1^, D Ortega^1^, L Martín‐Carbonero^1^, R Mican^1^, L Ramos^1^, M Valencia^1^, R Montejano^1^, M Montes^1^, M Díaz‐Almirón^2^, A Delgado^1^, J Bernardino ^1^

^1^HIV Unit, La Paz Hospital, Madrid, Spain; ^2^Biostatistic Unit, La Paz Research Institute, IdiPAZ, Madrid, Spain

**Background**: Most of the published studies switching from TDF‐based regimens to TAF‐based regimens raise concern about a worse metabolic profile (weight gain, higher lipids, and liver steatosis) in people with HIV (PWHIV). This study aims to evaluate the changes in lipid fractions, glucose, and triglyceride to glucose ratio (TyG) after switching from a TDF/TAF sparing regimen to B/F/TAF at 6 and 12 months.

**Material and methods**: A single‐centre, retrospective cohort study of PWHIV who switched to B/F/TAF from ART regimens without TDF or TAF from January 2019 to May 2022 with at least 6 months of follow‐up. The primary endpoint was the absolute change in lipid fractions at 6 months. Secondary outcomes were changes in lipid fractions at 12 months and other metabolic parameters (glucose, creatinine, and hepatic steatosis) measured by TyG ratio at 6 and 12 months. Mixed linear regression models with random intercept and time as a fixed effect were used to analyse these changes.

**Results**: A total of 147 PWHIV were included: median (P25 to 75) age 55 years (46 to 58), 81% male, 89% Caucasian, CD4+ T cell count 675 cells/mm^3^ (449 to 879), 79.6% HIV‐RNA <50 copies/mL. Forty‐four (30%) had hypertension, 72 (49%) dyslipidaemia, 24 (16%) diabetes, and 46% had obesity or overweight. Most of the participants (97; 66%) switched from integrase inhibitor‐based triple therapy (ABC/3TC + dolutegravir or raltegravir), and 28 (19%) received a boosted protease inhibitor (PI; nine patients 3TC+PI, 10 patients PI monotherapy). At 6 months there was a significant reduction in total cholesterol of ‐9.45 mg/dL (95% CI ‐16.43 to ‐2.48; p = 0.004), and in TyG ratio of ‐0.147 (95% CI ‐0.25 to ‐0.04; p = 0.0023). The percentage of patients with TyG >8.38 at baseline, 6 and 12 months was 75.2, 65.1, and 71.7. These differences were not statistically significant. The rest of the metabolic parameters are shown in Table 1.

**Table:** 

**Abstract P161 – Table 1**. Changes in metabolic and renal parameters.			
Parameter	Baseline N = 147	6 months N = 146	12 months N = 137
Total cholesterol, mg/dL			
Mean (95% CI)	182.8 (176.3 to 189.2)	173.3 (166.8 to 179.8)	175.2 (168.6 to 181.8)
Change from baseline (95% CI); p‐value		‐9.45 (‐16.43 to ‐2.48); p = 0.004	‐7.54 (‐14.67 to ‐0.41); p = 0.034
LDL cholesterol, mg/dL			
Mean (95% CI)	46.46 (44.48 to 48.44)	45.60 (43.62 to 47.58)	43.91 (41.90 to 45.92)
Change from baseline (95% CI); p‐value		‐0.855 (‐2.65 to 0.94); p = 0.761	‐2.55 (‐4.39 to ‐0.70); p = 0.003
Triglycerides, mg/dL			
Mean (95% CI)	155.74 (138.95 to 172.53)	135.89 (119.10 to 152.68)	144.75 (127.51 to 161.99)
Change from baseline (95% CI); p‐value		‐19.85 (‐41.92 to 2.22); p = 0.093	‐10.98 (‐33.58 to 11.60); p = 0.727
TC:HDL ratio			
Mean (95% CI)	4.12 (3.93 to 4.30)	3.89 (3.71 to 4.08)	4.14 (3.95 to 4.33)
Change from baseline (95% CI); p‐value		‐0.22 (‐0.44 to ‐0.001); p = 0.048	0.02 (‐0.20 to 0.24); p = 1.0
Glucose, mg/dL			
Mean (95% CI)	99.23 (94.80 to 103.67)	101.56 (97.13 to 105.99)	102.86 (98.37 to 107.34)
Change from baseline (95% CI); p‐value		2.32 (‐6.37 to 1.72); p = 0.50	3.62 (‐7.76 to 0.52); p = 0.1088
Creatinine, mg/dL			
Mean (95% CI)	0.93 (0.89 to 0.96)	0.96 (0.92 to 0.99)	0.96 (0.93 to 1.0)
Change from baseline (95% CI); p‐value		0.03 (0.009 to 0.05); p = 0.0025	0.03 (0.013 to 0.06); p < 0.001
CKD‐EPI, mg/min			
Mean (95% CI)	85.71 (83.60 to 87.82)	83.84 (81.73 to 85.95)	82.97 (80.84 to 85.10)
Change from baseline (95% CI); p‐value		‐1.87 (‐3.62 to ‐0.11); p=0.0319	‐2.73 (‐4.52 to ‐0.95); p < 0.001
TyG ratio			
Mean (95% CI)	8.79 (8.69 to 8.89)	8.64 (8.55 to 8.74)	8.74 (8.64 to 8.84)
Change from baseline (95% CI); p‐value		‐0.147 (‐0.25 to ‐0.04); p = 0.0023	‐0.005 (‐0.158 to 0.005); p = 0.7387

Multiple comparisons adjusted using the Bonferroni method.

**Conclusion**: In our real‐life cohort, the effect of switching ART regimens without TDF/TAF to triple therapy with B/F/TAF improved total cholesterol at both 6 and 12 months and was neutral for the rest of the metabolic parameters after 1 year of follow‐up.

#### P162 Liver enzyme variation after switching to emtricitabine/tenofovir alafenamide/bictegravir is associated with glucose increase in a real‐life cohort

N Squillace ^1^, E Ricci^2^, P Maggi^3^, B Menzaghi^4^, G De Socio^5^, G Orofino^6^, B Celesia^7^, A Bandera^8^, E Salomoni^9^, A Di Biagio^10^, L Taramasso^10^, S Piconi^11^, E Sarchi^12^, L Valsecchi^13^, G Pellicanò^14^, G Cenderello^15^, P Bonfanti^1^

^1^Department of Internal Medicine, Infectious Diseases Unit Azienda Socio Sanitaria Territoriale (ASST)‐MONZA, San Gerardo Hospital‐University of Milano‐Bicocca, Monza, Italy; ^2^Fondazione ASIA Onlus, Buccinasco (MI), Italy; ^3^Department of Internal Medicine, Infectious Diseases Unit, Azienda Ospedaliera di Rilievo Nazionale (AORN) Sant'Anna e San Sebastiano, Caserta, Italy; ^4^Department of Internal Medicine, Unit of Infectious Diseases, ASST della Valle Olona, Busto Arsizio, Italy; ^5^Department of Internal Medicine, Unit of Infectious Diseases, Santa Maria Hospital, Perugia, Italy; ^6^Department of Internal Medicine, Division I of Infectious and Tropical Diseases, Azienda Sanitaria Locale Città di Torino, Torino, Italy; ^7^Department of Internal Medicine, Unit of Infectious Diseases, Garibaldi Hospital, Catania, Italy; ^8^Department of Internal Medicine, Infectious Disease Unit, Fondazione Istituti di Ricovero e Cura a Carattere Scientifico Ca' Granda Ospedale Maggiore Policlinico, Milano, Italy; ^9^Department of Internal Medicine, Soc 1 Uslcentro Firenze, Unit of Infectious Diseases, Santa Maria Annunziata Hospital, Firenze, Italy; ^10^Department of Internal Medicine, Infectious Diseases, San Martino Hospital Genoa, University of Genoa, Genoa, Italy; ^11^Department of Internal Medicine, Unit of Infectious Diseases, A. Manzoni Hospital, Lecco, Italy; ^12^Department of Internal Medicine, Infectious Diseases Unit, S. Antonio e Biagio e Cesare Arrigo Hospital, Alessandria, Italy; ^13^Department of Infectious Diseases, 1st Department of Infectious Diseases, Azienda Socio Sanitaria Territoriale (ASST) Fatebenefratelli Sacco, Milano, Italy; ^14^Department of Internal Medicine, Infectious Diseases, G. Martino Hospital ‐ University of Messina, Messina, Italy; ^15^Department of Infectious Diseases, Sanremo Hospital, Sanremo, Italy

**Background**: Our aim was to investigate the role of emtricitabine/tenofovir alafenamide/bictegravir (FTC/TAF/BIC) regimen on metabolic and hepatic safety in a real‐life setting.

**Material and methods**: Consecutive patients living with HIV infection (PLWHIV) enrolled in SCOLTA project switching to or initiating their first antiretroviral treatment (ART) with FTC/TAF/BIC were included. T0 and T1 were defined as results at baseline and 6‐month follow‐up respectively. PLWHIV with HBV co‐infection were excluded. AST/creatinine (ac)NASH score [1] was calculated.

**Figure:**
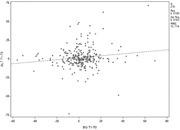
**Abstract P162 – Figure 1**. Correlation between change from baseline in ALT and glucose in nondiabetic experienced PLWHIV. ALT, alanine aminotransferase; BG, basal glucose.

**Results**: Out of 770 enrolled PLWHIV, 539 had at least one follow‐up visit and were included in the analysis. Mean age was 48 years (±12.1), 74% were male, 16.1% were naïve (N) to antiretrovirals, mean BMI was 25.4 (±4.7). Most experienced (Ex) PLWHIV were previously on FTC/TAF/elvitegravir/cobicistat (39.4%) or on FTC/TAF/dolutegravir (14.6%), had an undetectable HIV‐RNA <40 copies/mL (82.6%) and a median CD4 cell count of 600 cell/micrL (IQR 436 to 828). Eighty‐eight subjects (18.7%) were coinfected with HCV. At T1 (see Table 1), in patients N at baseline (BL), total cholesterol (TC), LDL‐cholesterol (LDL‐c), HDL‐cholesterol (HDL‐c) and triglycerides (TGL) showed a significant increase, while ALT decreased significantly. At T1 Ex‐PLWHIV showed a significant reduction of TC and TGL and an increase in glucose in PLWHIV without diabetes but with no difference in subjects with glucose >100 mg/dL at T0 versus T1. ALT increased significantly but within normal ranges. In Ex‐PLWHIV, at BL, a correlation was found between ALT and TGL (Spearman rho = 0.26, p < 0.0001), ALT and TGL/HDL‐c ratio (rho = 0.23, p < 0.0001), and glucose (rho = 0.13, p = 0.01). At T1 the same correlations were confirmed. Change from BL correlated in the case of ALT and glucose in nondiabetic Ex patients (rho = 0.14, p = 0.02 ; see Figure 1). Weight increased in naïve patients (+1.4 kg [95% CI 0.4 to 2.2], p = 0.04) but not in experienced ones.

**Conclusions**: ART initiation with FTC/TAF/BIC determined ALT decrease associated with acNASH reduction. In experienced PLWHIV, switching to FTC/TAF/BIC is associated with a significant amelioration of lipid profile, but with an increase of ALT and glucose. The correlation between ALT and glucose increase suggests a potential role of insulin resistance in the development of ALT increase.

**Table:** 

**Abstract P162 – Table 1**. Change from baseline by naive status.							
	Experienced	Experienced	Experienced	Naive	Naive	Naive	p^a^
	T0 mean SD or median (IQR) or N (%)	T1 mean SD or median (IQR) or N (%)	T1‐T0 mean (95% CI)	T0 mean SD or median (IQR) or N (%)	T1 mean SD or median (IQR) or N (%)	T1‐T0 mean (95% CI)	
Weight, kg	75.6±15.2	76.0±13.8	0.3 (‐0.2 to 0.8)	69.9±12.5	71.9±12.3	1.4 (0.4 to 2.2)^b^	0.04
Total cholesterol, mg/dL	194±42	188±40	‐5.4 (‐8.6 to ‐2.2)^b^	171±46	183±38	15.0 (7.3 to 22.6)^b^	<0.0001
LDL‐c, mg/dL	111±38	108±35	‐2.5 (‐5.5 to 0.4)	99±38	108±31	9.8 (3.4 to 16.2)^b^	0.001
HDL‐c, mg/dL	54±19	55±18	0.1 (‐1.0 to 1.3)	48±19	52±17	4.8 (1.9 to 7.8)^b^	0.002
TGL, mg/dL	115 (85 to 170)	104 (76 to 153)	‐13.1 (‐20.4 to ‐5.8)^b^	97.5 (77 to 149)	99 (77 to 131)	0 (‐13.5 to 13.5)	0.09
BG in nondiabetic	93±17	96±21	2.2 (0.5 to 4.0)^b^	89±13	89±12	0.4 (‐2.6 to 0.4)	0.29
BG in diabetic	165±72	168±70	2.3 (‐30.2 to 34.9)	170±26	173±72	32 (‐108 to 171)	0.53
eGFR mL/min	86.3±21.9	83.5±20.5	‐2.5 (‐2.9 to ‐1.1)^b^	105.1±29.4	89.9±23.7	‐15.3 (‐19.9 to ‐10.8)^b^	<0.0001
AST IU/L	22 (18 to 27)	23 (18 to 28)	‐0.6 (‐3.4 to 2.1)	25 (19 to 31)	22 (19 to 27)	‐3.8 (‐7.8 to 0.3)	0.22
ALT IU/L	22 (16 to 31)	23 (16 to 34)	2.4 (0.8 to 4.0)^b^	23 (17 to 31)	19 (14 to 26)	‐5.8 (‐11.0 to ‐0.7)^b^	0.0002
acNASH	(N = 311)	(n = 272)	‐0.22 (‐0.70 to 0.25)	(N = 77)	(N = 70)	‐0.95 (‐1.55 to ‐0.35)^b^	0.059
<4.15	272 (87.5%)	242 (89.0%)	——————————	52 (67.5%)	62 (88.6%)	————————————	————————————‐
4.15 to 7.73	33 (10.6%)	23 (8.5%)	———————————	21 (27.3%)	7 (10.0%)	——————————‐	————————————‐
≥7.74	6 (1.9%)	7 (2.6%)	——————————–	4 (5.2%)	1 (1.4%)	——————————–	————————————–

acNASH, AST/creatinine Non Alcoholic Steato‐Hepatitis score; ALT, alanine aminotransferase; AST, aspartate aminotransferase; BG, basal glucose; eGFR, estimated glomerular filtration rate; HDL‐c, high density lipoprotein‐cholesterol; IQR, interquartile range; IU, international unit; LDL‐c, low density lipoprotein‐cholesterol; SD, standard deviation; TGL, triglycerides.

^a^T1‐T0 comparison by naïve status;

^b^p < 0.05 for change from baseline.

**Reference**

1. Wu XX, Zheng KI, Boursier J, Chan W‐K, Yilmaz Y, Romero‐Gómez M, et al. acNASH index to diagnose nonalcoholic steatohepatitis: a prospective derivation and global validation study. EClinicalMedicine. 2021;41:101145.

#### P163 With or without TAF? What is the difference? Data from a real‐life setting

N Squillace ^1^, L Taramasso^2^, G Orofino^3^, P Maggi^4^, B Menzaghi^5^, S Piconi^6^, G De Socio^7^, E Sarchi^8^, L Valsecchi^9^, B Celesia^10^, F Vichi^11^, G Pellicanò^12^, G Cenderello^13^, K Falasca^14^, E Ricci^15^, A Di Biagio^2^, P Bonfanti^1^

^1^Department of Internal Medicine, Infectious Diseases Unit ASST‐MONZA, San Gerardo Hospital‐University of Milano‐Bicocca, Monza, Italy; ^2^Department of Internal Medicine, Infectious Diseases, San Martino Hospital Genoa, University of Genoa, Genoa, Italy; ^3^Department of Internal Medicine, Division I of Infectious and Tropical Diseases, ASL Città di Torino, Torino, Italy; ^4^Department of Internal Medicine, Infectious Diseases Unit, AORN Sant'Anna e San Sebastiano, Caserta, Italy; ^5^Department of Internal Medicine, Unit of Infectious Diseases, ASST della Valle Olona, Busto Arsizio, Italy; ^6^Department of Internal Medicine, Unit of Infectious Diseases, A. Manzoni Hospital, Lecco, Italy; ^7^Department of Internal Medicine, Unit of Infectious Diseases, Santa Maria Hospital, Perugia, Italy; ^8^Department of Internal Medicine, Infectious Diseases Unit, S. Antonio e Biagio e Cesare Arrigo Hospital, Alessandria, Italy; ^9^Department of Infectious Diseases, Department of Infectious Diseases, Luigi Sacco Hospital, Milano, Italy; ^10^Department of Internal Medicine, Unit of Infectious Diseases, Garibaldi Hospital, Catania, Italy; ^11^Department of Internal Medicine, Unit of Infectious Diseases, SOC1 USLCENTRO FIRENZE, Santa Maria Annunziata Hospital, Firenze, Italy; ^12^Department of Internal Medicine, Infectious Diseases, G. Martino Hospital ‐ University of Messina, Messina, Italy; ^13^Department of Infectious Diseases, Sanremo Hospital, Sanremo, Italy; ^14^Department of Medicine and Science of Aging, Clinic of Infectious Diseases, University 'G. d'Annunzio', Chieti, Italy; ^15^Fondazione ASIA Onlus, Fondazione ASIA Onlus, Buccinasco, Italy

**Background**: Our aim was to investigate the role of switching from emtricitabine/tenofovir alafenamide (FTC/TAF)‐based regimen to a dolutegravir (DTG)‐containing two‐drug regimen (2DR) versus continuing TAF‐based regimens (TAF‐BR) on metabolic and parameters and liver enzymes.

**Material and methods**: Consecutive people living with HIV infection (PLWHIV) enrolled in a multicentre observational cohort (SCOLTA) project, on a stable FTC/TAF‐based regimen with an HIV‐RNA <50 copies/mL were included. HBsAg‐positive PLWHIV were excluded. Changes from baseline (T0) to follow‐up (T1, week 24) were analysed.

**Results**: Three hundred and fifty‐seven PLWHIV met the inclusion criteria, 267 (74.8%) were males, 313 (87.7) Caucasians. One hundred and four switched to 2DR, 253 continued TAF‐BR. Twenty‐six PLWHIV had diabetes. The main characteristics at baseline were shown in Table 1. PLWHIV switching to 2DR or continuing TAF‐BR were different in terms of HCV coinfection, risk factor for HIV acquisition, previous regimen, CDC stage, and CD4. Comparing 2DR and TAF‐BR, we observed no differences in blood lipids modifications, whereas splitting by previous regimens with versus without cobicistat (COBI), total cholesterol (TC), LDL‐cholesterol (LDL‐c) and triglycerides (TGL) showed a significant decrease in patients switching from COBI‐containing regimens (mean change for TC ‐18 mg/dL vs ‐10 mg/dL; LDL‐c ‐12 mg/dL vs ‐7 mg/dL; TGL ‐22 mg/dL vs ‐18 mg/dL for 2DR vs TAF‐BR, p < 0.05 for each). Including current regimen and previous COBI in a general linear model, we confirmed the association between decreased blood lipids and COBI. Repeating the analyses on PLWH who did not take lipid‐lowering drugs at T0, we confirmed our results. Three patients began lipid‐lowering drugs during the follow‐up (one in 2DR and two in the TAF group). In PLWHIV who continued TAF‐BR, both with and without previous COBI, we observed a statistically significant increase in the ALT level (+4 IU/dL, p < 0.0001), when ALT at T0 was ≤40 IU/dL and within normal ranges value.

**Table:** 

**Abstract P163 – Table 1**. Baseline characteristics of 357 experienced patients in the study.			
Variables at enrolment	2DR N = 104 mean SD or N (%) or median (IQR)	TAF‐BR N = 253 mean SD or N (%) or median (IQR)	p
Age, years	48.5±13.2	48.4±11.5	0.96
Sex, M	80 (76.9%)	187 (73.9%)	0.55
BMI, kg/m^2^	25.4±3.9	26.0±6.0	0.35
Weight, kg	75.8±13.7	75.2±14.3	0.74
Caucasian	94 (90.4%)	219 (86.6%)	0.32
Risk factor for HIV acquisition			
Sexual	95 (91.4%)	174 (68.8%)	−
IDU	6 (5.6%)	40 (15.8%)	−
Other/ND	3 (2.9%)	39 (15.4%)	<0.0001
HCV coinfection (n = 107/329)	7 (6.9%)	55 (23.2%)	0.0004
Previous ART			
PI	7 (6.7%)	38 (15.0%)	0.03
INSTI	56 (53.8%)	191 (75.5%)	<0.0001
NNRTI	36 (34.6%)	14 (5.5%)	<0.0001
COBI‐including regimens	37 (35.6%)	161 (63.6%)	<0.0001
CDC stage			
A	80 (76.9%)	129 (51.2%)	−
B	19 (18.3%)	78 (31.0%)	−
C	5 (4.8%)	45 (17.9%)	<0.0001
CD4, cells/mm^3^	738 (614 to 926)	645 (490 to 838)	0.0002
Total cholesterol, mg/dL	197±37	196±41	0.91
HDL‐cholesterol, mg/dL	51±14	52±17	0.45
LDL‐cholesterol, mg/dL	119±32	116±36	0.35
Triglycerides, mg/dL	108 (80 to 154)	115 (85 to 163)	0.33
Blood glucose, mg/dL (nondiabetics)	88±12	93±15	0.004
Blood glucose, mg/dL (diabetics)	117±40	163±69	0.17
On lipid‐lowering drugs	8 (7.7%)	42 (16.6%)	0.03
AST, IU/dL	21 (17 to 25)	21 (18 to 26)	0.23
ALT, IU/dL	21 (16 to 27)	21 (16 to 29)	0.44

2DR, two‐drug regimen; ALT, alanine aminotransferase; AST, aspartate aminotransferase; BMI, body mass index; CDC, Centers for Disease Control and Prevention; COBI, cobicistat; HCV, hepatitis C virus; HDL, high density lipoprotein; IDU, intravenous drug user; INSTI, integrase strand transfer inhibitor; IQR, interquartile range; IU, international unit; LDL, low density lipoprotein; M, male; ND, not determined; NNRTI, non‐nucleoside reverse transcriptase inhibitor; PI, protease inhibitor; SD, standard deviation; TAF‐BR, tenofovir alafenamide‐based regimen.

**Conclusions**: No difference was found in TC, HDL‐c, LDL‐c, and blood glucose in PLWHIV continuing a TAF‐BR versus those switching to 2DR. Switching from a previous COBI‐including regimen was associated with a significant decrease in TC, LDL‐c, and TGL. In the TAF‐BR group  a significant ALT increase was observed.

#### P164 Abstract withdrawn

#### P165 Lipid changes in real‐world studies with the two‐drug regimen dolutegravir and lamivudine (DTG + 3TC) in people with HIV‐1: a systematic literature review

E Letang ^1^, J Lo^2^, A Milinkovic^3^, F Maggiolo^4^, S di Giambenedetto^5^, C Mussini^6^, I Ungan Yörük^7^, C Henegar^8^, J Priest^9^, B Young^10^, M Kabra^11^, B Jones^7^

^1^Global Medical Affairs, ViiV Healthcare, Madrid, Spain; ^2^Metabolism Unit, Massachusetts General Hospital, Harvard Medical School, Boston, MA, USA; ^3^Infectious Diseases, Chelsea and Westminster Hospital, London, UK; ^4^Antiviral Therapy Unit, Azienda Socio Sanitaria Territoriale Papa Giovanni XXIII, Bergamo, Italy; ^5^Infectious Diseases, Fondazione Policlinico Universitario Agostino Gemelli IRCCS and Università Cattolica del Sacro Cuore, Rome, Italy; ^6^Infectious Diseases, Clinic of Infectious Diseases, Azienda Ospedaliero‐Universitaria Policlinico, and University of Modena and Reggio Emilia, Modena, Italy; ^7^Global Medical Affairs, ViiV Healthcare, Brentford, UK; ^8^Epidemiology and Real World Evidence, ViiV Healthcare, Durham, NC, USA; ^9^Health Outcomes, ViiV Healthcare, Durham, NC, USA; ^10^Global Medical Affairs, ViiV Healthcare, Durham, NC, USA; ^11^Health Outcomes, ViiV Healthcare, Brentford, UK

**Background**: In randomized controlled trials of virologically suppressed people with HIV‐1 (PWH), switching to DTG/3TC from boosted TAF‐based regimens led to generally favorable changes in lipids through 144 weeks in the TANGO study and minimal changes in lipids when switching from various baseline regimens through 48 weeks in the SALSA study. Additionally, favorable decreases in total cholesterol/HDL‐cholesterol ratio were observed in treatment‐naive PWH initiating DTG + 3TC in the GEMINI‐1/2 studies. The purpose of this review was to summarize real‐world evidence (RWE) on the effect of DTG + 3TC on lipid parameters in PWH.

**Figure:**
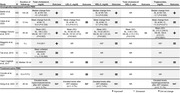
**Abstract P165 – Figure 1**. Lipid parameter outcomes with real‐world use of DTG + 3TC. ^a^ p‐value was the only value reported in the publication; ^b^ results were described as not significant and no other values were reported in the publication; ^c^ no statistical data were provided for change from baseline. Data shown are n (%) of PWH with elevated lipid parameters after ART initiation; ^d^ improved was defined as an increase from baseline in HDL‐C and as a decrease from baseline in total cholesterol, LDL‐C, total cholesterol/HDL‐C ratio, and triglycerides. NA, not available; NR, not reported; NS, not statistically significant; PWH, people with HIV‐1; PYFU, person‐years of follow‐up.

**Materials and methods**: We conducted a systematic literature review according to the Preferred Reporting Items for Systematic Reviews and Meta‐analysis statement. RWE studies reporting on DTG + 3TC use in PWH were retrieved from Ovid MEDLINE^®^, Embase^®^, PubMed, Cochrane library, and relevant international conference proceedings from January 2013 to February 2022. Publications providing data on lipid parameters associated with DTG + 3TC use were included.

**Results**: This systematic literature review includes 122 publications from 103 RWE studies of 44 unique cohorts (N = 8034) reporting on DTG + 3TC use. Of these 44 cohorts, eight reported data on lipid outcomes in 22 studies (N = 1141 PWH), including 20 studies of virologically suppressed PWH (n = 1094) and two of treatment‐naive PWH (n = 47) initiating DTG + 3TC (Table 1). Among virologically suppressed cohorts, mean/median age ranged from 47.1 to 60.5 years, 74% were male, and various ART regimens were used before switch (median treatment duration, 8.4 to 13 years). Duration of follow‐up ranged from 30 weeks to 5 years. In these studies, DTG + 3TC was associated with generally improved lipid profiles, with reductions or no changes in most lipid parameters reported (Figure 1). Among treatment‐naive cohorts, median age ranged from 31 to 34.5 years and 89% were male. Duration of follow‐up was 15.4 person‐years in one cohort and 48 weeks in the other. Lipid outcomes for these studies are summarized in Figure 1.

**Conclusions**: Consistent with clinical trial experience, RWE data from >1000 PWH suggest that switching to DTG/3TC from various regimens or initiating DTG + 3TC has minimal effects on lipid profiles.

**Table:** 

**Abstract P165 – Table 1**. Selected demographics and baseline characteristics by treatment experience and study.						
Virologically suppressed switch PWH						
Study (N)	Country	Age, median (IQR), y^a^	Sex, n (%)	Prior duration of ART, median (IQR), y^a^	Prior ART regimen	CD4+ cell count at switch, cells/mm^3^, median (IQR)^a^
Baldin et al, 2019	Italy	51.7	Female	11.5 (6.1 to 18.3)	2 NRTIs + NNRTI: 25.6%, 2 NRTIs + PI: 14.0%, 2 NRTIs + INI: 16.2%, dual therapy: 40.7%	668 (495 to 890)
(N = 556)		(45.3 to 57.4)	165 (29.7)			
Calza et al, 2020	Italy	47.1	Male	8.4 (2.6)^b^	Most common NRTIs: ABC/3TC: 49.1%, TDF/FTC: 45.8%; most common 3rd agent: DTG: 59.3%, bDRV: 18.6%	598 (217)^b^
(N = 59)		(18.5)^b^	43 (72.9)			
Hidalgo‐Tenorio et al, 2019	Spain	48.5	Male	13 (4 to 18)	Triple therapy: 65.5%, dual therapy: 18.1% (ATV/r + 3TC most common, 37.5%), monotherapy: 16.4% (bDRV most common, 93.1%)	697.7 (337.2)^b^
(N = 177)		(14.2)^b^	137 (77.4)			
Maggiolo et al, 2021	Multinational (Italy, 94%)	52 (12)	Male	10.2 (13)	NRTI: 93.6% (TDF most common, 59.2%), NNRTI: 49.5% (EFV most common, 18.8%), PI: 32.6% (DRV most common, 14.7%), INI: 22.5% (RAL most common, 11.0%)	669 (446)
(N = 218)			164 (75.2)			
Tan et al, 2019	UK	60.5	Male	9.4	TDF: 64%, ABC: 63%, EFV: 48%, DRV/r: 45%	94% with >350 cells/mm^3^
(N = 52)			44 (84.6)			
Yagci‐Caglayik, et al, 2017	Turkey	54	Male	Not reported	PI: 50% (LPV/r: 41%), NNRTI: 13% (EFV: 13%), INSTI: 53% (RAL: 22%, DTG: 22%), TDF/FTC: 66%, 3TC: 25%	272 (131 to 471)
(N = 32)		(41 to 64)^c^	27 (84.4)			
**Treatment‐naive PWH**						
**Study (N)**	**Country**	**Age, median (IQR), y^a^**	**Sex, n (%)**		**HIV‐1 RNA, median (IQR), copies/mL^a^**	**CD4+ cell count, cells/mm^3^, median (IQR)^a^**
Ciccullo et al, 2021	Italy	34.5	Male		4.78 log10 (4.01 to 5.00)	
(N = 20)		(25.2 to 53.5)	15 (75.0)			
Deng et al, 2022	China	31	Male		61 100 (33 500 to 229 000)^c^	222.07 (176.67)^b^
(N = 27)		(24 to 38)^c^	27 (100)			

^a^Unless otherwise indicated;

^b^mean (SD);

^c^median (range).

#### P166 New‐onset diabetes in persons with HIV on BIC/FTC/TAF in real‐world clinical practice

M Vivancos‐Gallego^1^, A Moreno‐Zamora^1^, M Pérez‐Elías ^1^, S Del Campo^1^, J Casado^1^, S Serrano‐Villar^1^, J Martínez‐Sanz^1^, R Ron^1^, M Sánchez‐Conde^1^, B Fernández‐Félix^2^, S Fernandez‐Garcia^2^, M Vélez Díaz‐Pallarés^3^, J Galán Montemayor^4^, S Moreno Guillén^1^

^1^Infectious Diseases, Hospital Ramón y Cajal and Instituto Ramón y Cajal de Investigación Sanitaria (IRYCIS), CIBERINFEC, Madrid, Spain; ^2^Biostatistics Unit, Hospital Ramón y Cajal and Instituto Ramón y Cajal de Investigación Sanitaria (IRYCIS), Madrid, Spain; ^3^Pharmacy Department, Hospital Ramón y Cajal and Instituto Ramón y Cajal de Investigación Sanitaria (IRYCIS), Madrid, Spain; ^4^Microbiology Department, Hospital Ramón y Cajal and Instituto Ramón y Cajal de Investigación Sanitaria (IRYCIS), Madrid, Spain

**Background**: The influence of new integrase strand‐transfer inhibitors (INSTI)‐based antiretroviral therapy (ART) on type 2 diabetes in people with HIV remains incompletely defined. Our aim is to describe the prevalence and incidence of prediabetes and diabetes in a cohort of people with HIV (PWHIV) receiving BIC/FTC/TAF.

**Methods**: Clinic‐based study in a tertiary, University Hospital in Madrid. HIV‐infected individuals starting on or switching to BIC/FTC/TAF (June 2018 to July 2021) were included. Data collected for sample selection included multiple fasting blood glucose and/or HBA1c measurements at first data collection date and all available values after starting treatment from BIC/FTC/TAF onwards and the date of BIC/FTC/TAF initiation. Diabetes and prediabetes were diagnosed if they met criteria in two or more consecutive blood tests according to the American Diabetes Association (ADA) definitions of diabetes (fasting plasma glucose at or above 126 mg/dL, HBA1c >6.5%) and prediabetes (HBA1c of 5.7% to 6.4% or fasting blood sugar of 100 to 125 mg/dL).

**Results**: The analysis focused on 1078 (360 naïve and 718 switch) BIC/FTC/TAF participants with completed data, excluding 63 (eight naïve and 55 switch) who had previous diabetes. Of the selected 1078 patients, median age stood at 48 years, 15% were women, 65% European. At their first visit on BIC/FTC/TAF, 198 out of 1078 (18.4%) met previous prediabetes criteria and in this group of patients diabetes developed in 16/198 (8.1%) after BIC/FTC/TAF initiation. Among 880 people without prediabetes criteria at their first visit on BIC/FTC/TAF, during follow‐up prediabetes developed in another 103 people (11.7%), mostly 62 (60.2%) ART‐experienced, and new diabetes developed in 11/880 (1.2%), six switched from previous ART.

**Conclusions**: A significant proportion of patients initiating BIC/FTC/TAF developed diabetes, although a causal relationship cannot be established. Since most patients progressed from prediabetes, information about previous prediabetes could be important in order to address the potential impact of each INSTI regimen influence on the onset of diabetes.

#### P167 Prevalence of dyslipidaemia and diabetes mellitus in people living with HIV and possible association with antiretroviral drug regimen: an 8 year follow‐up study

T Dorner^1^, T Obersteiner ^2^, H Schalk^2^, K Pichler^2^, I Grabovac^1^

^1^Department for Social and Preventive Medicine, Medical University Vienna, Center for Public Health, Vienna, Austria; ^2^Schalk‐Pichler Group Practice, Vienna, Austria

**Background**: For comprehensive care in people living with HIV (PLWHIV), not only antiretroviral therapy, but also detection and treatment of metabolic risk factors like type 2 diabetes mellitus (DM2), and dyslipidaemia are crucial. It was the aim of the study to assess the development of DM2 and dyslipidaemia prevalence in PLWHIV in a period of 8 years.

**Materials and methods**: Participants in the baseline study (2013) of one extramural HIV treatment centre in Vienna were recruited for follow‐up (2021). Dyslipidaemia and DM2 were indicated, if either the diagnosis was recorded in the patients' charts, OR the participant received lipid‐lowering or antidiabetic drugs OR there were suspicious laboratory findings (an LDL level >130 mg/dL OR an HDL level <40 mg/dL OR a triglyceride level >150 mg/dL for dyslipidaemia, and fasting blood sugar (FBS) >150 mg/dL for DM2).

**Results**: From the initially included 421 participants, 341 (81%) participated in the follow‐up. Mean age at baseline was 41.4 years, 5.3% were female. At baseline, the proportion treated with NRTI, NNTRI, PI, INSTI, and entry inhibitors were 88.3%, 56.4%, 25.0%, 5.6%, and 0.6%, and the corresponding proportions at follow‐up were 97.2%, 33.9%, 0.3%, 31.9%, and 0.6% (p < 0.001 for each category except entry inhibitors). Prevalence of dyslipidaemia was 73.6% at baseline and 71.4% at follow‐up (p = 0.504), and of DM2 1.9% at baseline and 5.3% at follow‐up (p = 0.017). Mean values for plasma lipids did not change significantly in the cohort; however, mean values for FBS changed from 84.1 to 78.0 mg/dL (p < 0.001). In patients who were treated with PIs at baseline and had switched to another drug class in the follow‐up, the change in FBS was 83.8 versus 78.6 mg/dL, p = 0.005, and for triglycerides 163.1 versus 144.2 mg/dL, p = 0.042.

**Conclusions**: Metabolic risk factors in PLWHIV remain a major challenge for comprehensive care. Prevalence of DM2 increased rapidly in 8 years, while prevalence of dyslipidaemia remained stable. Although there were more people detected with DM2 at follow‐up, the mean values for FBS were lower. Metabolic changes attributable to PI seem to be at least partially reversible after change of the therapeutic regimen.

### Co‐morbidities and Complications of Disease and/or Treatment: Neurological

#### P168 Drug‐related neuropsychiatric adverse events across phase III/IIIb studies of long‐acting cabotegravir + rilpivirine through week 48

E Elliot ^1^, P Teichner^2^, S Dakhia^1^, J Polli^2^, P Patel^2^, L Garside^3^, S Byrapuneni^4^, S Thiagarajah^5^, R D'Amico^2^, R Van Solingen‐Ristea^6^, E Birmingham^7^, B Baugh^8^, J van Wyk^1^

^1^ViiV Healthcare, Brentford, UK; ^2^ViiV Healthcare, Research Triangle Park, NC, USA; ^3^PHASTAR, Macclesfield, UK; ^4^Parexel International, Research Triangle Park, NC, USA; ^5^GSK, London, UK; ^6^Janssen Research & Development, Beerse, Belgium; ^7^Janssen Research & Development LLC, Raritan, NJ, USA; ^8^Janssen Research & Development, Titusville, NJ, USA

**Background**: Cabotegravir (CAB) + rilpivirine (RPV) is the first complete long‐acting (LA) regimen recommended by guidelines and approved for the maintenance of HIV‐1 virological suppression. Neuropsychiatric adverse events (NPAEs) have been reported in patients treated with multiple antiretroviral classes including integrase strand transfer inhibitors and non‐nucleoside reverse transcriptase inhibitors. This post hoc analysis summarises NPAEs in participants in the FLAIR, ATLAS, and ATLAS‐2M phase III/IIIb studies through week (W) 48.

**Materials and methods**: Data from CAB + RPV‐naive participants in FLAIR, ATLAS, and ATLAS‐2M through W48 were pooled. Incidence and characteristics of reported NPAEs through W48 were evaluated.

**Results**: In total, 1245 participants were randomised to switch to CAB + RPV LA, dosed every 8 weeks (n = 327) or every 4 weeks (n = 918), and 591 continued daily oral therapy (ATLAS and FLAIR studies only); baseline characteristics were similar between treatment groups (Table 1). One hundred and eleven drug‐related NPAEs (per investigator assessment) were reported by 53 of the 1245 participants switching to CAB + RPV LA, of which 96% (n = 107/111) were grade 1 or 2. There were four drug‐related grade 3 NPAEs and no grade 4 or serious drug‐related NPAEs in the CAB + RPV LA arm. The most common drug‐related NPAEs were nervous system events, headache (3%, n = 41/1245) and dizziness (2%, n = 23/1245), and psychiatric events, insomnia (1%, n = 15/1245), abnormal dreams (<1%, n = 12/1245), anxiety disorders (<1%, n = 11/1245), and depressive disorders (<1%, n = 10/1245) (Table 1). Withdrawal due to drug‐related NPAEs occurred in <1% (n = 10/1245) of participants. The conference presentation will include subgroup analyses of drug‐related NPAEs by baseline characteristics and by dosing regimen. 

**Conclusions**: NPAE results in this pooled phase III/IIIb analysis were consistent with antiretroviral therapy switch studies. Most drug‐related NPAEs were mild to moderate, with few grade 3 events and few leading to treatment withdrawal. No drug‐related NPAEs were classified as serious. These data support the central nervous system safety of CAB + RPV LA as a complete regimen for the treatment of HIV‐1.

**Table:** 

**Abstract P168 – Table 1**. Neuropsychiatric AEs through week 48 across the CAB + RPV LA phase III/IIIb programme.		
	CAB + RPV LA	Daily oral therapy^a^
	Q8W + Q4W pooled	
	(N = 1245)	(N = 591)
Baseline characteristics		
Median age (range), years	39 (19 to 83)	38 (18 to 82)
Female sex at birth, n (%)	310 (25)	168 (28)
Race, n (%)		
White	924 (74)	408 (69)
Black or African American	211 (17)	133 (23)
Other races	110 (9)	48 (8)
Safety summary		
Neuropsychiatric AEs overall, n (%)		
Total	319 (26)	104 (18)
Drug related	111 (9)	14 (2)
Grade 3^b^	4 (<1)	1 (<1)
Leading to withdrawal	10 (<1)	3 (<1)
Drug‐related^c^ neuropsychiatric disorders occurring in ≥5 participants in either arm, n (% of participants)^d^		
Nervous system disorders		
Headache	41 (3)	4 (<1)
Median duration, days (IQR)^e^	4 (2 to 8)	75 (45 to 104)
Dizziness	23 (2)	1 (<1)
Median duration, days (IQR)	2 (1 to 4)	Event not resolved
Psychiatric disorders		
Insomnia	15 (1)	1 (<1)
Median duration, days (IQR)^f^	14 (4 to 57)	112 (112 to 112)
Abnormal dreams	12 (<1)	2 (<1)
Median duration, days (IQR)	37 (7 to 58)	47 (41 to 52)
Anxiety disorders^g^	11 (<1)	3 (<1)
Median duration, days (IQR)	53 (6 to 74)	27 (3 to 73)
Depressive disorders^h^	10 (<1)	1 (<1)
Median duration, days (IQR)	28 (9 to 94)	3 (3 to 3)
Suicidal ideation/behaviour^i^	1 (<1)	1 (<1)
Median duration, days	4	3
Other (of potential interest)		
Seizures/seizure‐like events	0	0

AE, adverse event; CAB, cabotegravir; IQR, interquartile range; LA, long‐acting; Q4W, every 4 weeks; Q8W, every 8 weeks; RPV, rilpivirine.

^a^Participants receiving integrase strand transfer inhibitors, n = 382 (FLAIR, n = 283; ATLAS, n = 99);

^b^there were no grade 4 or 5 neuropsychiatric AEs;

^c^per investigator assessment;

^d^occurred in ≥5 participants except for the suicidal ideation/behaviour category (depression suicidal, n = 1 [CAB + RPV LA]; suicidal ideation, n = 1 [daily oral therapy]);

^e^median duration based on CAB + RPV LA n = 39 and daily oral therapy n = 2;

^f^median duration based on CAB + RPV LA n = 13;

^g^includes the following recorded terms: anxiety, anxiety disorder, nervousness, and panic attack;

^h^median duration based on CAB + RPV LA n = 8. Includes the following recorded terms: depression, depressed mood, and adjustment disorder with depressed mood. Median duration based on CAB + RPV LA n = 8;

^i^includes the following terms: depression suicidal and suicidal ideation. Suicidal ideation/behaviour occurred in <5 participants but is included as it may be of potential interest.

#### P169 Evaluation of the psychometric properties of health‐related quality of life patient‐reported outcome measures for use in people living with HIV with cognitive symptoms

K Alford ^1^, S Daley^2^, S Banerjee^3^, D Trotman^4^, E Hamlyn^4^, J Vera^5^

^1^Global Health and Infection, Brighton and Sussex Medical School, Brighton and Hove, UK; ^2^Centre for Dementia Studies, Brighton and Sussex Medical School, Brighton and Hove, UK; ^3^Faculty of Health, University of Plymouth, Plymouth, UK; ^4^Sexual Health and HIV Medicine, King's College Hospital NHS Foundation Trust, London, UK; ^5^Global Health and Infection, University Hospitals Sussex NHS Foundation Trust, Brighton and Hove, UK

**Background**: People living with HIV (PLWHIV) with cognitive symptoms report poor health‐related quality of life (HRQoL) [1]. Research and clinical care aiming to target and improve HRQoL in this population relies on HRQoL patient‐reported outcome measures (PROMs) to assess impact; however, there is limited evidence regarding which PROMs best capture this outcome in PLWHIV with cognitive symptoms. This study aimed to examine the psychometric properties of existing PROMs to assess HRQoL in PLWHIV with cognitive symptoms.

**Materials and methods**: PLWHIV with cognitive symptoms were identified (based on the European AIDS Clinical Society screening questions for cognitive impairment) from two HIV clinics in Brighton and London (UK) and asked to complete the Montreal Cognitive Assessment‐Blind (MoCA‐B) and four generic or illness‐specific (HIV or mild/moderate dementia) quality of life (QoL) or HRQoL PROMs. PROMs were selected based on frequency of use in both sites HIV memory services. PROMs included the WHOQOL‐BREF [2], EQ‐5D‐5L [3], HIVPROM [4] and DEMQOL [5]. We followed the COnsensus‐based Standards for the selection of health status Measurement INstruments (COSMIN) [6] recommendations for evaluating measurement properties which included statistical psychometric evaluations and cognitive debriefing exercises with a sub‐sample to assess content and face validity.

**Results**: One hundred and three PLWHIV with cognitive symptoms (male = 93 (90.3%); median age 58 years (range 33 to 88); White British = 66 (66.1%), Black African = 11 (10.7%); MoCA‐B mean score 17.85 (standard deviation 3.12)(<18 indicative of CI)) showed that the WHOQOL‐BREF and DEMQOL performed best in the statistical psychometric evaluations (including item skew, total score and domain score floor and ceiling effects, internal reliability, structural validity and convergent/divergent validity) (Table 1). However, PLWHIV with cognitive symptoms reported strong preferences for the relevance and comprehensibility of the DEMQOL and HIVPROM in the cognitive debriefing exercises (n = 10 PLWHIV with cognitive symptoms).

**Table:** 

**Abstract P169 – Table 1**. Statistical psychometric evaluations of the PROMs.					
PROM/psychometric evaluation	Item skew	Sub‐domain floor or ceiling effects	Internal validity (Cronbach's alpha)	Structural construct validity	Convergent/ divergent validity (average of hypothesised comparators, higher indicates better)
WHOQOL‐BREF	5 items + skew	None	Good (all >0.8)	All confirmed	0.65/0.43 (0.22)
EQ‐5D‐5L	1 item ‐ skew	4 sub‐domains	N/A	N/A	0.54/0.35 (0.19)
HIVPROM	11 items +/‐ skew	2 sub‐domains	2 domains poor (both <0.4)	1 domain unconfirmed	0.49/0.31 (0.18)
DEMQOL	None	None	Good (all >0.7)	All confirmed	0.62/0.43 (0.19)

**Conclusions**: The DEMQOL performed best overall in both the statistical psychometric evaluations and cognitive debriefing exercises and therefore could be used as the primary PROM for the assessment of HRQoL in PLWHIV with cognitive symptoms. Further research is needed to evaluate this PROM in PLWHIV with objective cognitive impairment.

**References**

1. Alford K, Daley S, Banerjee S, Hamlyn E, Trotman D, Vera JH. "A fog that impacts everything": a qualitative study of health‐related quality of life in people living with HIV who have cognitive impairment. Qual Life Res. 2022 May 17: doi: 10.1007/s11136‐022‐03150‐x.

2. WHO Group. The development of the World Health Organization WHOQOL‐BREF Quality of Life Assessment (the WHOQOL). Psychol Med. 1998;28:551‐8.

3. Herdman M, Gudex C, Lloyd A, Janssen MF, Kind P, Parkin D, et al. Development and preliminary testing of the new five‐level version of EQ‐5D (EQ‐5D‐5L). Qual Life Res. 2011;20:1727‐36.

4. Bristowe K, Murtagh FEM, Clift P, James R, Josh J, Platt M, et al. The development and cognitive testing of the positive outcomes HIV PROM: a brief novel patient‐reported outcome measure for adults living with HIV. Health Qual Life Outcomes. 2020;18:214.

5. Smith SC, Lamping DL, Banerjee S, Harwood RH, Foley B, Smith P, et al. Development of a new measure of health‐related quality of life for people with dementia: DEMQOL. Psychol Med. 2007;37:737‐46.

6. Mokkink LB, Terwee CB, Patrick DL, Alonso J, Stratford PW, Knol DL, et al. The COSMIN checklist for assessing the methodological quality of studies on measurement properties of health status measurement instruments: an international Delphi study. Qual Life Res. 2010;19:539‐49.

#### P170 Understanding the lived experience research priorities for improving quality of life in people living with HIV and cognitive impairment

K Alford ^1^, J Hammond^2^, J Vera^1^, S Daley^3^

^1^Global Health and Infection, Brighton and Sussex Medical School, Brighton and Hove, UK; ^2^Switchboard Helpline, Brighton and Hove LGBTQ+ Switchboard, Brighton and Hove, UK; ^3^Centre for Dementia Studies, Brighton and Sussex Medical School, Brighton and Hove, UK

**Background**: Cognitive impairment in people living with HIV (PLWHIV) is expected to rise as the HIV population continues to age. Estimates suggest 14% to 28% of PLWHIV have a cognitive impairment and are disproportionately affected at younger ages. PLWHIV with cognitive impairment report poor health‐related quality of life (HRQoL); however, interventions aimed at assisting PLWHIV to live well with cognitive impairment do not currently exist and represent an important unmet need in this population. This study aimed to identify the lived experience research priorities for improving HRQoL.

**Methods**: A research advisory group was established with 15 PLWHIV with cognitive impairment and healthcare, academic and voluntary sector staff. Two semi‐structured focus groups were undertaken, one with healthcare and voluntary sector staff (staff participants) and one with PLWHIV with cognitive impairment (lived experience participants). Participants were presented with seven factors influencing HRQoL from a previous study [1]. All participants were asked to rank factors in terms of priority and consider how each factor could be supported by intervention. Findings were analysed using content analysis. Study findings were fed back to our research advisory group.

**Results**: Five PLWHIV with cognitive impairment, recruited through voluntary sector agencies (male 80%; median age 59 (range 56 to 78); White British 60%, mixed race 20%, White other 20%; homosexual 60%, heterosexual 20%) and three healthcare and voluntary sector participants (66% voluntary sector; 33% clinical) confirmed the relevance of factors influencing HRQoL and suggested interventions to address them (Table 1). All participants ranked interventions targeting improvement in social connectedness and cognition as being most important, and lived experience participants further ranked perceived control over health and acceptance of cognitive impairment as important, and staff participants ranked physical functioning as a key priority.

**Conclusion**: This study is the first to identify the research priorities for improving or maintaining HRQoL in PLWHIV with cognitive impairment, and in doing so guides future work for interventional studies. Given the absence of intervention and support guidelines for PLWHIV with cognitive impairment, this provides a roadmap for future research in this important and growing area of HIV clinical care.

**Table:** 

**Abstract P170 – Table 1**. Factors and components influencing HRQoL and key interventions suggested based on focus group discussions.	
Factors influencing HRQoL [1]	Key intervention suggestions from focus groups
1. Physical function (components: Activities of daily living, Recreational activities and Employment/vocational activities)	Online tools for finance/medication management; Help to maintain employment and signposting to advocates to support this
2. Cognition (components: Memory and executive functioning, Concentration and attention, Communication)	Clarity of information on CI (easy language/written text); Staff training on indicators of CI and when to screen
3. Social connectedness (components: Social engagement and withdrawal, Social support)	Mapping of available services in area; Receiving and giving peer support; Social groups (safe/non‐judgemental space)
4. Physical and mental health and wellbeing (Physical health, Mental health, Global health appraisal)	Continued support/signposting following cognitive impairment diagnosis; Clear communication of CI management plan
5. Stigma (Self‐stigma, Enacted stigma, Barriers to helpseeking)	Awareness of indicators of CI (non‐adherence misinterpreted); Understanding complex stigma and impact of CI
6. Self‐concept (Self‐esteem and confidence, Identity, Independence, Future self (concerns), Attitude and personality)	Information ‐ what CI diagnosis means (not dementia diagnosis); Opportunities to socialise with similar age adults (care home residents)
7. Perceived control over health and acceptance (Understanding of health conditions and management, Employment of strategies)	Information ‐ multifactorial causes of CI, how to support good cognitive health; Knowledgeable staff; Collaborative strategy development

CI, cognitive impairment; HRQoL, health‐related quality of life.

**Reference**

1. Alford K, Daley S, Banerjee S, Hamlyn E, Trotman D, Vera JH. "A fog that impacts everything": a qualitative study of health‐related quality of life in people living with HIV who have cognitive impairment. Qual Life Res. 2022 May 17: doi: 10.1007/s11136‐022‐03150‐x.

#### P171 Effects of an online‐based cognitive stimulation training as a preventive programme in patients with HIV: a proof of concept study

J Cano‐Smith^1^, A Gonzalez‐Baeza^2^, G Rua‐Cebrian^1^, R Mican^1^, J Bernardino^1^, C Busca^1^, L Martín‐Carbonero^1^, J González‐García^1^, V Moreno^1^, E Valencia^1^, M Montes^1^, L Ramos^1^, I Pérez‐Valero ^3^

^1^HIV Unit, Hospital Universitario La Paz, Madrid, Spain; ^2^Biological and Health Psychology Department, Universidad Autónoma de Madrid, Madrid, Spain; ^3^Infectious Diseases ‐ HIV, Hospital Universitario Reina Sofia, Cordoba, Spain

**Background**: Cognitive complaints, frequently detected among people living with HIV (PLWHIV), could be associated with interferences on daily living due to executive dysfunction that impact on their quality of life.

**Methods**: Our proof of concept study was to design and evaluate the feasibility and acceptability of an online cognitive stimulation programme (OCSP) as an adjuvant therapy for PLWHIV complaining of cognitive disturbances. The OCSP entailing 24 sessions distributed in 12 weeks with 40 minutes of self‐applied exercises, specifically shaped with tasks to work on most affected cognitive domains in PLWHIV. Feasibility and acceptability of the OCSP were assessed using specific self‐reported questionnaires [Patient's Assessment of Own Functioning Inventory (PAOFI), dysexecutive questionnaire (DEX), Hospital Anxiety and Depression Scale (HADS) and a satisfaction and utility visual analogue feedback survey (VAS)] focused on determinants of brain health and key health outcomes, applied before and after the OCSP.

**Results**: Current analysis included the first 36 participants who started the OCSP. These participants were mostly men (86%) in their 60s (mean age: 60.5±7), with >8 years of school education (86%). Around 40% were currently employed and 37% were retired. Mean global deficit score (GDS) at baseline was 0.6 (mean GDS: 0.6±0.5). The 81% of the participants completed all the sessions and the 19% left remain 1 to 2 sessions to complete. Numerically we observed a 33.2% reduction in the percentage of functional cognitive complaints (PAOFI) and a 9.4% reduction in the percentage of dysexecutive complaints (Table 1). We also observed a significant reduction of self‐reported anxiety levels (HADS anxiety subscale raw results), though self‐reported depressive symptoms (HADS depressive subscale) remained the same. The feedback of the participants indicated high grades of satisfaction (VAS score: 8.42 points), perceived value (VAS score: 8.33) and interest in the programme (VAS score: 8.61).

**Table:** 

**Abstract P171 – Table 1**. Self‐reported questionnaires online CSP.				
Questionnaire	Data	Pre	Post	p‐value
PAOFI (Patient's Assessment of Own Functioning Inventory)	QUANT M (SD)	4.72 (7.19)	4.61 (7.03)	0.87
PAOFI (Patient's Assessment of Own Functioning Inventory)	QUAL n (%)	9 (25.0)	6 (16.7)	0.375^a^
DEX TOTAL (Dysexecutive questionnaire)	QUANT M (SD)	22.75 (12.727)	20 (11.17)	0.12
DEX TOTAL (Dysexecutive questionnaire)	QUAL n (%)	32 (88.9)	29 (80.5)	0.161
DEX DISORG (Dysexecutive disorganization/apathy subscale)	QUANT M (SD)	12.36 (7.60)	11.14 (7.57)	0.21
DEX DISORG (Dysexecutive disorganization/apathy subscale)	QUAL n (%)	23 (63.95)	20 (55.6)	0.46
DEX DESINH (Dysexecutive disinhibition/impulsivity subscale)	QUANT M (SD)	10.39 (5.78)	8.86 (5.1)	0.12
DEX DESINH (Dysexecutive disinhibition/impulsivity subscale)	QUAL n (%)	18 (50.0)	16 (44.4)	0.774
HADS ANX (Hospital Anxiety and Depression Scale‐Anxiety subscale)	QUANT M (SD)	8.75 (3.99)	7.55 (3.86)	0.03*
HADS ANX (Hospital Anxiety and Depression Scale‐Anxiety subscale)	QUAL n (%)	20 (55.56)	15 (44.44)	0.17
HADS DEP (Hospital Anxiety and Depression Scale Depression subscale)	QUANT M (SD)	5.97 (4.19)	5.36 (4.21)	0.26
HADS DEP (Hospital Anxiety and Depression Scale Depression subscale)	QUAL n (%)	26 (72.2)	26 (72.2)	1.000^a^
Satisfaction degree	QUANT M (SD)		8.42 (1.91)	
Invested time in program	QUANT M (SD)		8.61 (1.87)	
Utility perceived	QUANT M (SD)		8.33 (2.13)	

^a^Binomial distribution used;

*p‐value <0.05 statistically significant.

**Conclusions**: Our findings support the feasibility and acceptability of our OCSP. A larger sample size is needed to confirm the potential benefits observed in our study on functional cognitive complaints, dysexecutive disability and anxiety and the effectiveness of online cognitive stimulation programmes.

**References**

1. Frain JA, Chen L. Examining the effectiveness of a cognitive intervention to improve cognitive function in a population of older adults living with HIV: a pilot study. Ther Adv Infect Dis. 2018;5:19‐28.

2. Vance DE, Cody SL, Moneyham L. Remediating HIV‐associated neurocognitive disorders via cognitive training: a perspective on neurocognitive aging. Interdiscip Top Gerontol Geriatr. 2017;42:173‐86.

3. Antinori A, Arendt G, Becker JT, Brew BJ, Byrd DA, Cherner M, et al. Updated research nosology for HIV‐associated neurocognitive disorders. Neurology. 2007;69:1789‐99.

#### P172 The effects of switching from dolutegravir/abacavir/lamivudine to bictegravir/emtricitabine/tenofovir alafenamide in virologically suppressed people living with HIV on neuropsychiatric symptoms: preliminary findings from a randomised study

B Rossetti^1^, M Ferrara^2^, L Taramasso^3^, F Bai^4^, F Lombardi^5^, N Ciccarelli^6^, M Durante^7^, F Alladio^2^, I Rancan^8^, F Montagnani^8^, A Di Biagio^9^, A d'Arminio Monforte^4^, M Zazzi^7^, M Fabbiani ^10^

^1^Azienda USL Toscana Sud‐Est, Ospedale Misericordia, Grosseto, Italy; ^2^Department of Medical Sciences, Unit of Infectious Diseases, University of Torino, Torino, Italy; ^3^Department of Internal Medicine, Infectious Diseases Clinic, Istituto di Ricovero e Cura a Carattere Scientifico, Policlinico San Martino Hospital, Genova, Italy; ^4^Department of Health Sciences, Clinic of Infectious Diseases, Azienda Socio Sanitaria Territoriale Santi Paolo e Carlo, Milano, Italy; ^5^Infectious Diseases Unit, Fondazione Policlinico Universitario A. Gemelli Istituto di Ricovero e Cura a Carattere Scientifico, Roma, Italy; ^6^Department of Psychology, Catholic University of Milano, Milano, Italy; ^7^Department of Medical Biotechnologies, University of Siena, Siena, Italy; ^8^Department of Medical Biotechnologies, Infectious and Tropical Diseases Unit, University Hospital of Siena, Siena, Italy; ^9^Department of Health Sciences, Infectious Diseases Clinic, San Martino Hospital‐IRCCS, Genova, Italy; ^10^Department of Specialized and Internal Medicine, Infectious and Tropical Diseases Unit, University Hospital of Siena, Siena, Italy

**Background**: Central nervous system adverse events (AE) occur with various antiretrovirals (ARVs) and have been a cause of discontinuation of dolutegravir‐containing ART, especially when used in combination with abacavir. The main aim of the study was to evaluate whether the switch to bictegravir/emtricitabine/tenofovir alafenamide (B/F/TAF arm) is associated with a reduction in severity and incidence of neuropsychiatric symptoms compared to continued dolutegravir/abacavir/lamivudine (DTG/ABC/3TC arm). Quality of life, suicide risk, cognitive impairment and other self‐reported symptoms (using validated questionnaires) were also evaluated.

**Materials and methods**: DOBINeuro is an ongoing, 12‐month, open label, randomised trial enrolling PLWHIV treated with DTG/ABC/3TC for >6 months and with HIV‐1 RNA (VL) <50 copies/mL for >12 months. Exclusion criteria include previous AIDS events, active alcohol intake or substance abuse, major psychiatric disorders, history of virological failure on InSTI and HBsAg+. At baseline (BL) PLWHIV were randomised to continue DTG/ABC/3TC or switch to B/F/TAF. Here we describe preliminary findings at 3 months (3M) follow‐up.

**Results**: We included 34 PLWHIV (73% males, median age 51 years, median CD4 693 cells/mm^3^): 17 were randomised to continue DTG/ABC/3TC and 17 to switch to B/F/TAF (Table 1). At BL, clinical and laboratory characteristics were homogeneous in the two arms; overall, 14.7% of participants showed cognitive impairment at BL (def. global Z score ≤‐1) with no difference between arms. At 3 months no significant differences were observed between and within arms regarding self‐reported adherence, quality of life assessment and suicide risk. Being depressed (p = 0.029) and having muscle aches (p = 0.017) were less frequently reported with B/F/TAF at 3M than at BL. VL was confirmed <50 copies/mL for all participants in both arms. Only two non‐serious AE were reported: one grade 3 triglycerides with DTG/ABC/3TC and one self‐limited episode of abdominal pain with B/F/TAF, which was not treatment‐related and did not lead to drug discontinuation.

**Table:** 

**Abstract P172 – Table 1**. Baseline characteristics (n = 34).			
	Arm A n = 17	Arm B n = 17	p
	DTG/3TC/ABC	B/F/TAF	
Age, years (median, IQR)	50.4 (41.8 to 56.8)	51.8 (30.0 to 58.1)	0.718
Male gender, n (%)	12 (70.6)	13 (76.5)	1.000
Caucasian ethnicity, n (%)	15 (88.2)	12 (70.6)	0.398
Risk category			0.308
Heterosexuals, n (%)	8 (47.1)	4 (23.5)	
Men who have sex with men, n (%)	6 (35.3)	9 (52.9)	
Injecting drug users, n (%)	1 (5.9)	0	
Other/unknown, n (%)	2 (11.8)	4 (23.5)	
Years from HIV diagnosis (median, IQR)	10.7 (4.9 to 21.5)	9.9 (5.4 to 19.0)	0.796
Years from first ART (median, IQR)	9.8 (4.7 to 21.5)	9.8 (4.7 to 14.8)	0.570
Nadir CD4, cells/mm^3^ (median, IQR)	299 (123 to 420)	334 (138 to 516)	0.796
Years on DTG/3TC/ABC (median, IQR)	4.6 (3.2 to 4.9)	4.6 (3.6 to 5.2)	0.399
Years from last HIV‐RNA >50 copies/mL (median, IQR)	6.2 (3.7 to 8.2)	6.0 (4.2 to 11.7)	0.419
HCV Ab+, n (%)	0	0	
BMI, kg/m^2^ (median, IQR)	23.5 (21.6 to 28.7)	24.5 (19.9 to 29.4)	0.896
CD4, cells/mm^3^ (median, IQR)	613 (457 to 995)	784 (522 to 1288)	0.296
CD4/CD8 ratio (median, IQR)	0.99 (0.77 to 1.39)	0.84 (0.42 to 1.65)	0.692

**Conclusions**: Switch from DTG/ABC/3TC to B/F/TAF in virologically suppressed PLWHIV was associated with improvement in two reported symptoms. Further data from the 12‐month study follow‐up are required to evaluate the potential impact on the incidence and severity of neuropsychiatric symptoms of treatment switch to B/F/TAF compared to continued DTG/ABC/3TC.

#### P173 Sleep health of Nigerian adults living with HIV: looking beyond the absence of disease

A Osiyemi ^1^, E Owoaje^2^, J Mundt^3^, B Taiwo^4^

^1^Family Medicine, University College Hospital, Ibadan, Nigeria; ^2^Department of Community Medicine, Faculty of Public Health, College of Medicine, University of Ibadan, Ibadan, Nigeria; ^3^Department of Neurology, Center for Circadian and Sleep Medicine, Northwestern University Feinberg School of Medicine, Chicago, IL, USA; ^4^Division of Infectious Diseases, Northwestern University Feinberg School of Medicine, Chicago, IL, USA

**Background**: Sleep research among persons living with HIV (PLWHIV) has historically focused on identifying and treating sleep disorders [1‐6]. Sleep health is a positive framework to view the sleep of individuals [7]. It is a multidimensional construct of sleep and wakefulness that operationalises optimal sleep as more than the absence of disease [8‐10]. Six important dimensions of sleep include sleep regularity, subjective satisfaction, appropriate timing, adequate duration, high sleep efficiency, and sustained alertness during the day. These six constructs are well‐measured by the RU‐SATED scale [10]. Research on sleep health of PLWHIV is limited in Africa. This study assessed sleep health of treatment‐experienced PLWHIV, mostly on dolutegravir (DTG)‐based therapies, and associated factors of sleep health.

**Materials and methods**: Questionnaires were administered to 300 adult participants recruited from the Infectious Diseases Institute, Ibadan, Nigeria (IDI). We collected data regarding sociodemographic, sleep health (RU‐SATED scale where higher scores indicate more optimal sleep), sleep disturbance (PSQI), depression (Patient Health Questionnaire; PHQ‐9), and anxiety (Generalized Anxiety Disorder‐7; GAD‐7). HIV‐specific data were retrieved from IDI's medical records. Pearson correlation coefficient was used to assess factors associated with sleep health.

**Result**: For the sample, the mean age was 44.5 years±11.4 with 230 females (76.7%) and 95% on DTG‐based ART regimens. The mean duration of HIV diagnosis was 8.3 years±5.1. The average sleep health score was 6.3±2.4. Depression and anxiety were present in 14% and 17.3% respectively. Prevalence of sleep disturbance (PSQI score of 6 or more) was 21.7%. Higher sleep health scores were associated with younger age (r = ‐0.15; p = 0.007), longer duration of HIV diagnosis (r = 0.12, p = 0.041), lower PHQ‐9 scores (r = ‐0.34; p < 0.001), lower GAD‐7 scores (r = ‐0.33; p < 0.001) and lower PSQI (r = ‐0.15, p = 0.009). There was no significant association between sleep health and the most recent viral load count.

**Conclusions**: PLWHIV report average sleep health scores using validated measures. Age, sleep disturbance, depression and anxiety are significant factors associated with lower sleep health scores and as such, addressing these factors may improve the sleep health of PLWHIV.

**References**

1. Taibi DM, Price C, Voss J. Sleep disturbances in persons living with HIV. J Assoc Nurses AIDS Care. 2013;24:411‐21.

2. Oshinaike O, Akinbami A, Ojelabi O, Dada A, Dosunmu A, John Olabode S. Quality of sleep in an HIV population on antiretroviral therapy at an urban tertiary centre in Lagos, Nigeria. Neurol Res Int. 2014;2014:298703.

3. Yunusa M, Yunusa MA, Obembe A, Su F. Prevalence of self reported sleep problems among patients with HIV infection in Sokoto, Nigeria. Br J Med Med Res. 2016;15:5‐8.

4. Ogunbajo A, Restar A, Edeza A, Jin H, Iwuagwu S, Williams R, et al. Poor sleep health is associated with increased mental health problems, substance use, and HIV sexual risk behavior in a large, multistate sample of gay, bisexual and other men who have sex with men (GBMSM) in Nigeria, Africa. Sleep Health. 2020;6:662‐70.

5. Shittu RO, Odeigah LO, Moradeyo AK, Sanni MA, Aderibigbe SA, Sule AG, et al. Short sleep duration and correlates among sero‐positive HIV patients in Nigeria, West Africa. Br J Med Med Res. 2015;10:1‐10.

6. Bhaskar S, Hemavathy D, Prasad S. Prevalence of chronic insomnia in adult patients and its correlation with medical comorbidities. J Family Med Prim Care. 2016;5:780.

7. Buysse DJ. Sleep health: can we define it? Does it matter? Sleep. 2014;37:9‐17.

8. Dalmases M, Benítez I, Sapiña‐Beltran E, Garcia‐Codina O, Medina‐Bustos A, Escarrabill J, et al. Impact of sleep health on self‐perceived health status. Sci Rep. 2019;9:7284.

9. Dalmases M, Benítez ID, Mas A, Garcia‐Codina O, Medina‐Bustos A, Escarrabill J, et al. Assessing sleep health in a European population: results of the Catalan Health Survey 2015. PLoS One. 2018;13:e0194495.

10. Ravyts SG, Dizerzewski JM, Perez E, Donovan EK, Dautovich N. Sleep health as measured by RU SATED: a psychometric evaluation. Behav Sleep Med. 2021;19:48‐56.

### Co‐morbidities and Complications of Disease and/or Treatment: Mental Health Disorders

#### P174 Resolution of neuropsychiatric adverse events after switching to a doravirine‐based regimen in the open‐label extensions of the DRIVE‐AHEAD and DRIVE‐FORWARD trials

G Moyle ^1^, H Wan^2^, F Meng^2^, R Plank^2^, P Sklar^2^, R Lahoulou^3^

^1^HIV Research Strategy, Chelsea & Westminster Hospital, London, UK; ^2^Research, Merck & Co., Inc., Rahway, NJ, USA; ^3^Research, MSD, Puteaux, France

**Background**: Neuropsychiatric adverse events (NPAEs) are observed with multiple antiretrovirals. Doravirine (DOR) does not significantly interact in vitro with known neurotransmitter receptors. Among phase III study participants receiving the fixed combination of DOR with lamivudine/tenofovir (3TC/TDF) as first‐line therapy, the rate of NPAEs was significantly lower than with efavirenz plus emtricitabine/tenofovir (EFV/FTC/TDF) and similar to darunavir/r (DRV/r) with two NRTIs. We examined NPAEs in participants who switched to a DOR‐based regimen in the open‐label extensions of two phase III clinical trials.

**Materials and methods**: In DRIVE‐AHEAD (NCT02403674) and DRIVE‐FORWARD (NCT02275780), participants were randomized to a DOR regimen (DOR/3TC/TDF or DOR with two NRTIs) or the comparator regimen (EFV/FTC/TDF or DRV/r with two NRTIs) for 96 weeks of double‐blind (DB) treatment. Upon completing the DB phase, eligible participants in the comparator groups could switch to the DOR‐based regimen for 96 weeks in an open‐label study extension.

**Results**: At DRIVE‐AHEAD study week 96 (W96), 269 participants switched from their original randomly assigned regimen of EFV/FTC/TDF to DOR/3TC/TDF. During the DB phase, NPAEs were reported by 155 (57.6%) of these participants, compared with 26.4% of those originally randomized to DOR/3TC/TDF. At W96, 26 participants on EFV/FTC/TDF had ongoing NPAEs, of which 19 were resolved/resolving following switch to DOR/3TC/TDF. At DRIVE‐FORWARD W96, 233 participants switched from their original randomly assigned DRV/r to DOR. During the DB phase, NPAEs were reported by 41 (17.6%) of these participants while receiving DRV/r, compared with 15.7% of those originally randomized to DOR. At W96, 15 participants on DRV/r had ongoing NPAEs, of which six were resolved/resolving following switch to DOR. In the two open‐label extensions, new NPAEs (most commonly sleep disorders and depression) were reported by 25 participants (9.3%) who switched from EFV/FTC/TDF to DOR/3TC/TDF and 18 participants (7.7%) who switched from DRV/r to DOR; by end of treatment, these NPAEs were resolved/resolving in 15 and 11 participants, respectively (Table 1).

**Conclusions**: Among participants with ongoing NPAEs while receiving EFV/FTC/TDF, a high proportion (19/26) experienced resolution after switching to DOR/3TC/TDF. The similar rate of NPAEs with doravirine and darunavir‐based regimens may represent the background rate for these events.

**Table:** 

**Abstract P174 – Table 1**. Resolution of NPAEs after switch to DOR‐based regimen.		
	EVF/FTC/TDF	DRV/r + 2 NRTIs
Switched to DOR regimen at week 96	269	233
NPAE during DB phase (week 0‐96)	155 (57.6%)	41 (17.6%)
NPAE ongoing at week 96	26 (9.7%)	15 (6.4%)
NPAE still ongoing at week 192 after switch to DOR	7 (2.6%)	9 (3.9%)
New NPAE after switch to DOR (week 96‐192)	25 (9.3%)	18 (7.7%)
New NPAE still ongoing at week 192	10 (3.7%)	7 (3.0%)

#### P175 Defining the cascade of mental healthcare: increased prevalence of depression and lack of access to care for people ageing with HIV

H Okhai ^1^, A Winston^2^, F Post^3^, M Boffito^4^, P Mallon^5^, J Vera^6^, I Williams^1^, M Sachikonye^7^, M Johnson^8^, J Anderson^9^, C Prechtl^10^, C Sabin^1^

^1^Institute for Global Health, University College London, London, UK; ^2^Department of Medicine, Imperial College Healthcare NHS Trust, London, UK; ^3^Caldecot Centre, King's College Hospital, London, UK; ^4^St Stephen's Centre, Chelsea and Westminster Hospital, London, UK; ^5^School of Medicine, University College Dublin, Dublin, Ireland; ^6^Elton John Centre, Brighton and Sussex University Hospital, Brighton, UK; ^7^UK Community Advisory Board (UK‐CAB), London, UK; ^8^Ian Charleson Day Centre, Royal Free Hospital, London, UK; ^9^Centre for the Study of Sexual Health and HIV, Homerton University Hospital, London, UK; ^10^Imperial Clinical Trials Unit, Imperial College London, London, UK

**Background**: Depressive symptoms remain prevalent among people with HIV (PWH) who are suppressed on antiretroviral therapy (ART). Although some data describe the unmet need of mental healthcare in this population, we now conceptualise the cascade of mental healthcare for PWH using data from the Pharmacokinetic and Clinical Observations in People Over Fifty (POPPY) study.

**Figure:**
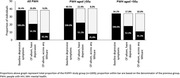
**Abstract P175 – Figure 1**. Cascade of mental healthcare; defining the proportion of people with HIV (PWH) who report depressive symptoms, clinical diagnosis of depression and those who have accessed mental health services, stratified by older (>50 years) and younger (<50 years) age groups.

**Materials and methods**: PWH participating in the POPPY study who completed the Patient Health Questionnaire‐9 (PHQ‐9) or Center for Epidemiologic Studies Depression Scale (CES‐D) tools at the baseline visit (conducted between 2013 and 2016) were included. Moderate/severe depressive symptoms were defined as PHQ‐9≥10 or CES‐D≥16. Self‐reported medically diagnosed depression and access to any specialist mental healthcare in the 12 months prior to the person's baseline visit were described. The proportion of individuals experiencing depressive symptoms and reporting a clinical diagnosis of depression, and proportion of those with clinical depression reporting mental healthcare were compared between PWH aged >50 years and <50 years using χ2 tests.

**Results**: In total, 1009 PWH (65.5% aged >50 years, 85.8% male, 85.1% white, 90.6% undetectable viral load) were included. At baseline, nearly 40% (387/1009) of individuals were experiencing depressive symptoms, over half of whom (210/387) reported having a medical diagnosis of depression. Only 43.3% (91/210) of individuals who were medically diagnosed with depression reported accessing some form of mental healthcare. The most accessed mental healthcare included visiting a psychologist/psychiatrist (35/91) or counsellor (22/91). The prevalence of depressive symptoms was higher amongst PWH aged >50 years (40.5%) versus <50 years (34.2%, p = 0.05) (Figure 1). Although amongst those, there was no difference in the reported rates of medical diagnosis of depression between PWH aged >50 years (55.2%) and <50 years (52.1%, p = 0.57), a lower proportion of older PWH reported accessing mental health services (35.8% vs 61.3%, respectively, p < 0.001).

**Conclusions**: This analysis highlights the higher prevalence of depressive symptoms and lack of use of mental health services for people with HIV, especially amongst those ageing with HIV. It is important to understand the barriers to accessing mental healthcare, particularly as the population of people with HIV in the UK increases in age.

#### P176 Psychological wellbeing and sleep in human immunodeficiency virus (HIV) ‐ a retrospective analysis of assessment and interventions within HIV services in the UK and Ireland (UKI)

N Galbraith ^1^, J Paparello^1^, S Gyampo^1^, K Carroll^1^, A Brown^2^

^1^HIV Standards Support Team, Gilead Sciences, London, UK; ^2^Analytics, Gilead Sciences, London, UK

**Background**: People living with HIV are disproportionately affected by psychological wellbeing and sleep issues which can detrimentally impact their quality of life, adherence and health outcomes [1,2]. Despite monitoring and assessment being imperative to improve long‐term health, evidence indicates a variation in incidence of this and absence in guidance for sleep issues [3]. To support the generation of evidence in this field, a market research study was designed to gain insights into current interventions for psychological wellbeing and sleep assessment within HIV services in UKI.

**Materials and methods**: The study was managed by a market research agency where an online survey link was disseminated to healthcare professionals (HCPs) in multiple HIV centres across UKI. To ensure accuracy of data, HCPs randomly selected a maximum 20 patient notes reviewed between 2020 and 2022. No identifiable patient information was recorded or shared with resulting data presented at an aggregate level.

**Results**: Thirty‐nine clinics participated contributing 665 patient notes with demographics reflective of UKI population. Since Covid‐19 77% of HCPs perceived an increasing demand for mental health support with 64% stating they routinely assess mental health; however, the majority express issues with capacity and resourcing to sufficiently support these patients. Thirty‐three percent of patients included were identified as experiencing a decline in psychological wellbeing, the majority of which self‐reported during face‐to‐face (F2F) routine appointments; 14% of these patients had a Patient Health Questionnaire 9 (PHQ9). Seventy‐eight percent received support with the majority signposted to external resources. For those who did not receive support, the primary driver was patient request. Forty‐six percent of services state they do not routinely assess for sleep issues. A lower proportion of patients (17%) were identified as having such issues; however, of those identified the primary method was self‐reporting during F2F routine appointments. Six percent of these patients had a Pittsburgh Sleep Quality Index (PSQI). Of those who did not receive sleep support, a lack of guidance was the main cited reason.

**Conclusions**: This study indicates high variation between local management of psychological wellbeing and sleep in HIV, in addition to key gaps in clinical guidance, identifying, managing and ongoing monitoring which is required to ensure long‐term health.

**References**

1. Gooden TE, Gardner M, Wang J, Chandan JS, Beane A, Haniffa R, et al. The risk of mental illness in people living with HIV in the UK: a propensity score‐matched cohort study. Lancet HIV. 2022;9:e172‐81.

2. Brown A, Paparello J, Alfred S, Khamlichi S, Gibson S, Mbewe R. HIV service provision: how can we empower the HIV community to have access and confidence in a sustainable and person‐centric care model? HIV Med. 2021:85.

3. Patterson B, Mitchell C, Mbewe R, Sabin C. Guidelines for the assessment and monitoring of comorbidities in people living with human immunodeficiency virus (PLHIV): a systematic review. HIV Med. 2021;22:253‐4.

### Co‐morbidities and Complications of Disease and/or Treatment: Other

#### P177 Measuring what matters: how do we assess sleep in HIV care?

M Croston^1^, K Bourne^2^, E Hurt^3^, N Galbraith ^4^, M Hayter^3^

^1^Faculty of Medicine and Health Sciences, University of Nottingham, Nottingham, UK; ^2^Infectious Diseases, Greater Manchester Mental Health NHS Foundation Trust, Manchester, UK; ^3^Department of Nursing, Manchester Metropolitan University, Manchester, UK; ^4^HIV Standards Support Team, Gilead Sciences, London, UK

**Background**: Despite medical advances, people living with HIV experience significant issues affecting health‐related quality of life; one such issue is sleep. Although poor sleep quality is common in this population, there remains a lack of understanding of how to identify sleep issues within clinical practice to improve outcomes for people living with HIV.

**Materials and methods**: A scoping review was conducted searching Cinahl, Pubmed, Psychinfo and the grey literature. Inclusion and exclusion criteria were developed with data selection and charting undertaken by two reviewers using a qualitative content approach.

**Results**: Out of 2932 retrieved articles 60 met the inclusion criteria. Publication dates ranged from 1992 to 2021, a third of papers were published in 2020 and 2021 (n = 17). Over half the studies were conducted in the US (n = 35), and the majority were cross‐sectional in design (n = 48). Across all studies there were 25 904 participants, of which 21 561 were people living with HIV. The following themes were identified when exploring how sleep was measured; range of methods available to assess, self‐reported and objective. The review found a number of different measures of sleep used, with the most favoured approach being the PSQI (N = 48). Due to the variety of approaches used (n = 18) there was a lack of consistency to what aspects of sleep were being explored, and in many cases why the measure was chosen.

**Conclusions**: Clinicians need more awareness of the different types of sleep difficulties and disorders there are, consider the aspect of sleep they are concerned about and choose a suitable assessment tool or tools. Future research should explore the effectiveness of different methods of assessing sleep to establish the best way to monitor sleep within clinical practice. The results help healthcare professionals consider the multivariant nature of sleep to identify appropriate measures of sleep to be explored further, including potential alternatives to the Pittsburgh Sleep Quality Index (PSQI) such as the SATED questionnaire or a single question approach. Despite all 60 studies highlighting sleep issues there was a lack of meaningful clinical recommendations on how findings could be used to improve outcomes for people living with HIV.

#### P178 Pre‐ART platelet‐to‐lymphocyte ratio and the risk of serious non‐AIDS events, AIDS events and mortality in PLWHIV starting first‐line ART

P Saltini ^1^, A Cozzi‐Lepri^2^, G Bozzi^3^, G Marchetti^4^, L Taramasso^5^, S Mazzanti^6^, V Rizzo^7^, A Antinori^8^, C Mussini^9^, A Gori^3^, A D'Arminio Monforte^10^, A Bandera^3^

^1^Infectious Disease Unit, University of Milan, Milan, Italy; ^2^Institute for Global Health, UCL, London, UK; ^3^Infectious Disease Unit, Fondazione Istituti di Ricovero e Cura a Carattere Scientifico (IRCCS) Ca' Granda Ospedale Maggiore Policlinico, Milan, Italy; ^4^Infectious Disease Unit, Department of Health Sciences, Azienda Socio Sanitaria Territoriale (ASST) Santi Paolo e Carlo, Milan, Italy; ^5^Infectious Disease Unit, San Martino Polyclinic Hospital ‐ Istituti di Ricovero e Cura a Carattere Scientifico (IRCCS) for Oncology and Neurosciences, Genoa, Italy; ^6^Infectious Disease Unit, Ospedali Riuniti Umberto I, Ancona, Italy; ^7^Immunodeficiency and Gender Related Infectious Diseases Unit, Department of Infectious Disease and Infectious Emergencies, Azienda Ospedaliera di Rilievo Nazional dei Colli, P.O. Cotugno, Naples, Italy; ^8^HIV/AIDS Department, National Institute for Infectious Diseases Lazzaro Spallanzani ‐ Istituti di Ricovero e Cura a Carattere Scientifico (IRCCS), Rome, Italy; ^9^Infectious Disease Unit, University Hospital of Modena, Modena, Italy; ^10^Infectious Disease Unit, Azienda Socio Sanitaria Territoriale (ASST) Ospedali Santi Paolo e Carlo, Department of Health Services, Milan, Italy

**Background**: Among people living with HIV (PLWHIV) on effective antiretroviral therapy (ART), serious non‐AIDS events (SNAEs), linked to systemic inflammation, are the main cause of morbidity. Neutrophil‐to‐lymphocyte ratio (NLR), platelet‐to‐lymphocyte ratio (PLR) and lymphocyte‐to‐monocyte ratio (LMR) are markers of inflammation and prognostic factors for many conditions in general population, but they have been poorly analysed in PLWHIV.

**Materials and methods**: PLWHIV starting first‐line ART (baseline) between 1997 and 2021 enrolled in ICONA Foundation Study cohort with a measure of NLR, PLR and LMR over 6 months before ART initiation were included. Baseline PLR, NLR and LMR values were divided in tertiles based on their distribution (T1, T2, T3). The association between baseline PLR, NLR and LMR and the risk of SNAEs or death, AIDS events or death and all‐cause mortality were tested using Kaplan‐Meier and Cox proportional hazard models adjusting for age, CD4 count, viral load, HCV status, and year of starting ART.

**Results**: We included 9248 patients in the PLR analysis, 8727 for NLR analysis and 1090 for LMR analysis. Participants were mainly males, aged 38 years, with a median baseline CD4 count of 330/mmc. Baseline PLR was significantly associated with age, female gender, mode of HIV transmission, nationality, AIDS diagnosis, HBV and HCV coinfection, current and nadir CD4 count, CD8 count, smoking and lower time from HIV diagnosis (Table 1). After a median follow‐up of 5 years, 489 SNAEs, 390 AIDS events and 371 deaths were observed. By 15 years, the risk of SNAEs or death and all‐cause mortality were significantly higher for T1‐PLR (Figure 1), also in the adjusted analysis. Similarly, in the unadjusted analysis, the risk of SNAEs or death, AIDS or death and all‐cause mortality was higher for T3‐NLR and T1‐LMR, but none of these associations retained statistical significance after controlling for previously mentioned confounding factors.

**Figure:**
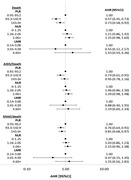
**Abstract P178 – Figure 1**. Adjusted hazard ratios (AHR) of death, AIDS/death, SNAE/death from fitting standard Cox regression models with time‐fixed covariates.

**Table:** 

**Abstract P178 – Table 1**. Characteristics of PLWHIV according to PLR ratio tertile.					
Characteristics at starting ART	T1 (n = 3083)	T2 (n = 3085)	T3 (n = 3080)	p‐value	Total
Female, n (%)	463 (15%)	681 (22.1%)	978 (31.8%)	<0.001	2122 (22.9%)
Mode of HIV transmission				<0.001	
PWID	597 (19.4%)	436 (14.1%)	387 (12.6%)		1420 (15.4%)
MSM	1388 (45.0%)	1390 (45.1%)	1039 (33.7%)		3817 (41.3%)
Heterosexual contacts	952 (30.9%)	1114 (36.1%)	1444 (46.9%)		3510 (38.0%)
Other/unknown	146 (4.7%)	145 (4.7%)	210 (6.8%)		501 (5.4%)

Not Italian	763 (24.7%)	934 (30.3%)	1150 (37.3%)	0.001	2847 (30.8%)
AIDS diagnosis, n (%)	150 (4.9%)	165 (5.3%)	668 (21.7%)	<0.001	983 (10.6%)
CVD diagnosis, n (%)	23 (0.7%)	19 (0.6%)	29 (0.9%)	0.337	71 (0.8%)
HBsAg, n (%)				0.009	
Negative	2595 (84.2%)	2579 (83.6%)	2630 (85.4%)		7804 (84.4%)
Positive	28 (0.9%)	25 (0.8%)	8 (0.3%)		61 (0.7%)
Not tested	460 (14.9%)	481 (15.6%)	442 (14.4%)		1383 (15.0%)
HCV‐Ab, n (%)				<0.001	
Negative	2029 (65.8%)	2204 (71.4%)	2205 (71.6%)		6438 (69.6%)
Positive	642 (20.8%)	441 (14.3%)	400 (13.0%)		1483 (16.0%)
Not tested	412 (13.4%)	440 (14.3%)	475 (15.4%)		1327 (14.3%)
Hepatitis co‐infection, n (%)				<0.001	
No	1914 (62.1%)	2075 (67.3%)	2139 (69.4%)		6128 (66.3%)
Yes	660 (21.4%)	460 (14.9%)	405 (13.1%)		1525 (16.5%)
Not tested	509 (16.5%)	550 (17.8%)	536 (17.4%)		1595 (17.2%)
Calendar year of starting ART, median (IQR)	2012 (2003 to 2016)	2013 (2005 to 2016)	2013 (2006 to 2017)		2013 (2004 to 2016)
Age, median (IQR)	38 (31 to 46)	37 (31 to 45)	39 (33 to 47)	<0.001	38 (32 to 46)
CD4 count cells/mmc, median (IQR)	404 (276 to 579)	370 (254 to 505)	195 (54 to 337)	<0.001	330 (184 to 479)
CD4 count nadir cells/mmc, median (IQR)	383 (260 to 536)	351 (240 to 476)	186 (51 to 321)	<0.001	312 (174 to 452)
CD8 count cells/mmc, median (IQR)	1270 (920 to 1706)	899 (703 to 1153)	559 (376 to 766)	<0.001	866 (590 to 1243)
CD4 count <200/mmc, n (%)	436 (14.2%)	530 (17.2%)	1552 (51.1%)	<0.001	2518 (27.4%)
Months from HIV diagnosis, median (IQR)	13 (2 to 60)	6 (1 to 44)	2 (1 to 21)	<0.001	4 (1 to 43)

**Conclusions**: Our data show that in PLWHIV starting ART, baseline PLR is a strong predictor of the risk of SNAEs and mortality independently of key confounding factors. Because the biomarker is derived from blood parameters routinely collected, its use should be encouraged to identify PLWHIV at higher risk of poor long‐term outcomes.

#### P179 Lymphogranuloma venereum is highly prevalent among rectal chlamydia infections and HIV‐positive men who have sex with men in Austria: findings from an observational multicentre study

D Chromy ^1^, B Sadoghi^2^, I Gasslitter^3^, M Skocic^4^, A Okoro^1^, K Grabmeier‐Pfistershammer^1^, B Willinger^5^, W Weninger^1^, A Öllinger^4^, M Sarcletti^3^, G Stary^1^, W Bauer^1^

^1^Department of Dermatology, Medical University of Vienna, Vienna, Austria; ^2^Department of Dermatology and Venereology, Medical University of Graz, Graz, Austria; ^3^Dermatology, Venereology and Allergy, Medical University of Innsbruck, Innsbruck, Austria; ^4^Department of Dermatology, Kepler Universitätsklinikum, Linz, Austria; ^5^Division of Clinical Microbiology, Department of Laboratory Medicine, Medical University of Vienna, Vienna, Austria

**Background**: Serovar L1‐L3 of *Chlamydia trachomatis* (Ct) causes a rare invasive infection known as lymphogranuloma venereum (LGV). In high‐income countries, a surge in cases has been observed predominantly among HIV‐positive men who have sex with men (MSM) in recent years. With the increasing use of HIV‐preexposure prophylaxis (PrEP), HIV‐negative MSM are also becoming progressively at risk of infection. Discrimination between LGV and non‐LGV Ct is pivotal since it has major treatment implications. Here, we aimed to determine the prevalence of LGV among Ct infections and the characteristics of affected individuals in Austria.

**Materials and methods**: For this multi‐centre study, available Ct‐positive results between 04/2014 and 12/2021 at the four largest Austrian HIV and STI clinics, accounting for 61% of Austrian's residents, were evaluated retrospectively. Disease characteristics and patients' demographics were analysed.

**Figure:**
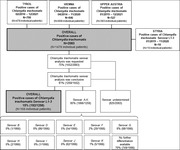
**Abstract P179 – Figure 1**. Flow chart.

**Results**: Overall, N = 2083 infections of Ct were documented among N = 1479 individual patients: the median age was 31.4 years, 81% were male, 59% MSM, 44% HIV positive and 13% on PrEP. Results on serovar analysis were available in 61% (1258/2083) of cases; 15% (192/1258) showed serovar L1‐L3 (Figure 1). Considering only MSM with rectal Ct infection, serovar L1‐L3 accounted for 23% (101/439). Those affected by LGV versus other Ct infections were significantly older (median 37.1 years IQR 11.8 vs median 31.6 years IQR 13.9; p < 0.001), primarily MSM (92% (177/192) vs 62% (1179/1891), p < 0.001) and more often HIV positive (64% (116/180) vs 46% (631/1376); p < 0.001). No significant difference in PrEP use (16% (29/180) vs 19% (268/1376); p < 0.280) was observed, whereas LGV cases were more often accompanied by both previous (65% (117/180) vs 40% (553/1376); p < 0.001) and concomitant syphilis infection (18% (32/180) vs 7% (52/749); p < 0.001), but not concomitant gonorrhoea (20% (36/180) vs 17% (232/1376); p = 0.294). The most common manifestation of LGV was proctitis observed in 38% of cases (72/192) whereas 45% (87/192) were asymptomatic.

**Conclusions**: Lymphogranuloma venereum was predominantly observed among HIV‐positive and HIV‐negative MSM and, importantly, accounted for 23% of rectal Ct infections in MSM. Furthermore, almost half (45%) of all LGV cases were asymptomatic. In the absence of Ct serovar analysis, a high LGV prevalence should be considered in populations at risk and empiric treatment should be selected accordingly.

#### P180 Osteoporosis among older persons living with HIV in Kenya: baseline results from the BFTAF elderly switch study

L Ombajo ^1^, J Penner^2^, J Nkuranga^1^, R Wanjohi^3^, J Mecha^1^, M Mburu^1^, F Ndinya^4^, S Eshiwani^5^, S Wahome^5^, E Otieno^1^, A Pozniak^6^, S Bhagani^7^

^1^Clinical Medicine and Therapeutics, University of Nairobi, Nairobi, Kenya; ^2^Center for Epidemiological Modelling and Analysis, University of Nairobi, Nairobi, Kenya; ^3^Radiology, The Nairobi Hospital, Nairobi, Kenya; ^4^Clinical Services, Jaramogi Oginga Odinga Teaching and Referral Hospital, Kisumu, Kenya; ^5^Critical Care Center, Kenyatta National Hospital, Nairobi, Kenya; ^6^Clinical Research, London School of Hygiene and Tropical Medicine, London, UK; ^7^Infectious Disease/ HIV Medicine, Royal Free London Foundation Trust, London, UK

**Background**: There is a paucity of data on bone mineral density (BMD) among elderly people living with HIV (PLWHIV). We evaluated BMD among virally suppressed PLWHIV age ≥60 years.

**Materials and methods**: The bictegravir/emtricitabine/tenofovir alafenamide (BFTAF) elderly study is an open‐label, randomised, active‐controlled, non‐inferiority trial conducted at two sites in Kenya. Eligible participants were HIV‐1 positive adults ≥60 years of age who were virally suppressed on any antiretroviral regimen without prior virological failure. Participants were randomised (1:1) to switch to BFTAF or continue their pre‐enrolment regimen. At enrolment a BMD monitoring population underwent dual‐energy x‐ray absorptiometry (DXA) of the lumbar spine and total hip using a Hologic Discovery A scanner with APEX software (version 4.5.3), with T‐scores calculated for BMD. Osteoporosis was defined as T‐score ≤–2.5 and osteopenia as a T‐score between –1 and –2.5. Fracture risk was calculated using Fracture Risk Assessment Tool (FRAX).

**Results**: Between February and May 2022, 296 participants were enrolled into the BMD monitoring population. All participants were black African, 147 (49.7%) were female, median age was 64 years (range 60 to 77) and 280 (94.6%) were on tenofovir disoproxil fumarate (TDF)‐containing regimens (Table 1). Median BMD of the lumbar spine and total hip were 0.88 g/cm^2^ (IQR 0.78 to 1.00) and 0.88 g/cm^2^ (IQR 0.80 to 0.99), respectively, translating to median T‐scores of –2.7 (IQR –3.5 to –1.6) and –1.3 (IQR –1.9 to –0.7), respectively. Osteoporosis and osteopenia were found in 60.5% and 29.1% of participants, respectively. Using FRAX score alone (without BMD results) only identified 10 (3.4%) participants who qualified for treatment of osteoporosis.

**Conclusions**: Prevalence of osteoporosis among elderly PLWHIV in Kenya is high. DXA is not readily available in Kenya, and risk calculation without BMD did not identify the majority of participants who qualify for treatment of osteoporosis. Additional data for this population are required on clinical implications of undiagnosed osteoporosis, ARV regimen selection to reduce osteoporosis risk, particularly in populations with widespread use of TDF, and on the impact of investment in osteoporosis screening and treatment, including population‐specific risk calculators.

**Table:** 

**Abstract P180 – Table 1**. Baseline characteristics. Data are median (IQR) or n (%) unless otherwise stated.	
	Total (n = 296)
Age (years); median (max, min)	64 (60, 77)
Sex	
Female	147 (49.7%)
Male	149 (50.3%)
Race: Black	296 (100%)
Any alcohol use in past 12 months	
No	292 (98.7%)
Yes	4 (1.4%)
Ever smoked	
No	289 (97.6%)
Yes	7 (2.4%)
Body mass index (kg/m^2^)	27.5 (24.0, 30.9)
HBV co‐infection	3 (1.0%)
HIV‐1 RNA <50 copies/mL	296 (100%)
Median enrolment CD4 count (cells/uL)	632 (483, 808)
Median nadir CD4 count (cells/uL)	25 (18, 31)
CrCl <60 mL/min	105 (35.6%)
Time since HIV diagnosis (years)	12.6 (9.1, 15.4)
Time on ART (years)	9.5 (5.3, 9.6)
Baseline ART regimen	
DTG + TDF + 3TC	279 (94.3%)
DTG + ABC + 3TC	15 (5.1%)
DTG + AZT + 3TC	1 (0.3%)
EFV + TDF + 3TC	1 (0.3%)
BMD lumbar spine (g/cm^2^)	0.88 (0.78, 1.0)
BMD T‐score lumbar spine	‐2.7 (‐3.5, ‐1.6)
BMD total hip (g/cm^2^)	0.88 (0.80, 0.99)
BMD T‐score total hip	‐1.3 (‐1.9, ‐0.7)
BMD category (using lowest T‐score from lumbar spine or total hip)	
Normal	31 (10.5%)
Osteopenia	86 (29.1%)
Osteoporosis	179 (60.5%)
Treatment of osteoporosis recommended based on FRAX (without input of BMD)^a^	10 (3.4%)

3TC, lamivudine; ABC, abacavir; AZT, zidovudine; BMD, bone mineral density; CrCl, creatine clearance using the Cockcroft‐Gault equation; DTG, dolutegravir; EFV, efavirenz; FRAX, fracture risk assessment tool; TDF, tenofovir disoproxil fumarate.

^a^Treatment of osteoporosis is recommended if 10‐year probability of major osteoporotic fractures is ≥20% or of hip fracture is ≥3%.

#### P181 Lack of prediction of fragility fractures by risk assessment tools in a cohort of people with HIV

P Vizcarra^1^, A Moreno ^1^, M Vivancos Gallego^1^, A Muriel García^2^, J González‐García^3^, S Moreno Guillén^1^, S Ibarra Ugarte^4^, J Olalla^5^, D Dalmau^6^, J Casado^1^

^1^Infectious Diseases, Ramón y Cajal University Hospital, Madrid, Spain; ^2^Biostatistics Unit, Ramón y Cajal University Hospital, Madrid, Spain; ^3^Internal Medicine II, La Paz University Hospital, Madrid, Spain; ^4^Infectious Diseases, Hospital Universitario Basurto, Bilbao, Spain; ^5^Internal Medicine, Hospital Costa del Sol, Málaga, Spain; ^6^Infectious Diseases, Hospital Universitari Mutua Terrassa, Barcelona, Spain

**Background**: Current guidelines recommend screening people with HIV (PWHIV) for bone disease using predictive tools developed in the general population, though data on PWHIV are scarce.

**Materials and methods**: In this study, the accuracy of FRAX and Qfracture scoring systems to predict the occurrence of fragility fractures was assessed in a prospective cohort of 17 671 adults with HIV infection of the AIDS Research Network (CoRIS) in Spain. We calculated the survival estimates of fragility fractures during follow‐up and computed the FRAX and Qfracture scores for each individual at cohort inclusion. For both tools, discriminatory measures and the observed to expected ratios were assessed. Fracture risk was expressed overall, by age and quintiles of risk.

**Results**: During a follow‐up time of 42 411.55 person‐years, 113 first episodes of fragility fractures were recorded (86 major osteoporotic fractures and 11 hip fractures). Areas under the receiver operating curve (AUC) were 0.66 for FRAX and 0.67 for Qfracture for major osteoporotic fractures, and 0.72 and 0.81 for hip fracture, respectively. The observed to expected ratios were 1.92 for FRAX and 5.28 for Qfracture for major osteoporotic fractures, and 2.00 for FRAX and 4.67 for Qfracture for hip fractures, indicating underestimation of the risk. Moreover, observed to expected ratios increased as the risk increased for both tools and in almost all age groups. When using the recommended assessment thresholds, less than 6% and 10% of major osteoporotic and hip fractures would have been identified, respectively.

**Conclusions**: FRAX and Qfracture displayed similar discriminative capacity in PWHIV compared with studies in the general population. However, the tools significantly underestimated the risk of fractures in PWHIV. The recommended assessment thresholds were not able to identify fragility fractures during follow‐up. A fracture prediction tool developed for PWHIV is needed.

#### P182 Neurosyphilis in people living with HIV: current incidence and diagnostic approach

P Vizcarra, A Moreno, M Vivancos Gallego, J del Pino, S Martín‐Colmenarejo, A Abad, J Casado

Infectious Diseases, Ramón y Cajal University Hospital, Madrid, Spain

**Background**: The diagnosis of neurosyphilis in people living with HIV (PLWHIV) remains controversial. We evaluated the current diagnostic approach based on neurological signs or treatment failure, rather than the recommended one based on CD4+ counts ≤350 cells/μL or RPR titers ≥1:32.

**Materials and methods**: Prospective longitudinal study of PLWHIV with syphilis serological testing (RPR, EIA, HAI) every 6 months. A lumbar puncture was performed to individuals with neurological signs at presentation or treatment failure at 12‐month follow‐up.

**Results**: A total of 205 PLWHIV were included. Median age was 43 years (interquartile range [IQR] 36 to 52), all were male, and 97% were MSM. Median time of HIV infection was 81 (IQR 22 to 143) months, median nadir CD4+ counts were 302 (IQR 166 to 420) cells/μL, and current CD4+ counts were 581 cells/μL (IQR 423 to 856, <350 cells/μL in 16%). Syphilis stage was classified as early in 130 individuals (63%; of which it was primary in 30 ‐15%‐, secondary in 40 ‐20%‐, and latent in 60 ‐46%‐), and late latent or of unknown duration in 75 (37%). RPR titers ≥1:32 were observed in 71% of PLWHIV. Of note, there was an inverse correlation between CD4+ counts at diagnosis and RPR titers (rho = ‐0.172; p = 0.02). Neurological symptoms at diagnosis were referred by four (2%) individuals. After a median time of 147 (IQR 109 to 214) days, 156 (76%) PLWHIV had serological response, increasing to 177 (87%) after 366 days (IQR 288 to 467). RPR titers ≥1:32 were associated with serological response (89% vs 76% if RPR <1:32; p = 0.06), whereas CD4+ counts ≤350 cells/μL were associated with treatment failure (response: 73% vs 89% if CD4 350 cells/μL; p = 0.021). After excluding reinfection, 19 (9%) PLWHIV with neurological symptoms or treatment failure underwent a lumbar puncture, with cerebrospinal fluid (CSF) pleocytosis ≥6 white blood cells/μL in eight (42%) individuals, increased protein concentration in three (16%), and positive VDRL in two (11%). Thus, a final diagnosis of neurosyphilis was performed in seven PLWHIV (3% overall, 37% of those with CSF testing criteria).

**Conclusions**: Neurosyphilis was unusual in our population. The current diagnosis approach enabled the selection of individuals at high risk of neurosyphilis, avoiding the use of invasive tests and saving resources.

#### P183 Evolution of TDF‐associated tubular dysfunction after switching to tenofovir alafenamide (TAF) in people living with HIV

P Vizcarra^1^, J del Rey^2^, C Santiuste^2^, A Moreno ^1^, S Gomez‐Maldonado^1^, J Casado^1^

^1^Infectious Diseases, Ramón y Cajal University Hospital, Madrid, Spain; ^2^Clinical Biochemistry, Ramón y Cajal University Hospital, Madrid, Spain

**Background**: We report the evolution of tubular and renal function in people living with HIV (PLWHIV) who had tubular dysfunction during TDF treatment, and who were switched to a tenofovir alafenamide (TAF)‐containing regimen.

**Methods**: Virologically suppressed PLWHIV were enrolled in a study of TDF‐associated renal toxicity during 2014 to 2016 and followed up after TDF discontinuation, independently of the cause. In this analysis, we included individuals with a prior diagnosis of tubular dysfunction (≥2 alterations in tubular parameters: proteinuria, glycosuria, hyperuricosuria, phosphaturia) who received TAF‐containing regimens during follow‐up. The primary endpoint was the change in estimated glomerular filtration rate (eGFR) and the changes in parameters of tubular dysfunction.

**Results**: One hundred and ninety‐eight PLWHIV who discontinued TDF received TAF during follow‐up. Of them, 32 (16%) had a diagnosis of tubular dysfunction, including three (2%) cases of Fanconi syndrome. PLWHIV with tubular dysfunction were significantly older, had a worse eGFR and lower levels of phosphataemia compared to those without tubular dysfunction, leading to early TDF discontinuation (after 9.7 vs 30.1 months; p < 0.01). After a median of 25.5 months, all the individuals were switched to a TAF‐containing regimen and received it for 28.4 months (IQR 12.5 to 62.7). During TAF‐containing regimens, tubular parameters improved (proteinuria ‐11.4 mg/gr, FE of phosphate ‐0.66%, FE of uric acid ‐0.92) but not the eGFR (‐6.5 mL/min) due to the inhibition of creatinine secretion by concomitant antiretrovirals. Tubular dysfunction persisted in 19% of individuals, associated with age (57.6 vs 50.8 years, p < 0.001), previous tubular dysfunction (relative risk [RR] 3.03; 95% CI 1.62 to 5.66), longer time on TDF (95 vs 76.2 months; p = 0.036), presence of hypertension‐diabetes (RR 2.4; 95% CI 1.2 to 7.1), and a worse renal and tubular status at the time of TDF switch (eGFR, 75.6 vs 94.3mL/min; p = 0.003; FE of phosphate 72% vs 79.1%, p = 0.003; proteinuria 166.7 vs 92.3 mg/gr; p = 0.009; albuminuria 59.1 vs 15.6 mg/gr; p = 0.003).

**Conclusions**: In PLWHIV with tubular dysfunction who switched to TAF there was an overall improvement in tubular parameters. However, even after several years, there was no or mild benefit in individuals who switched after long exposure to TDF and/or with advanced renal and tubular impairment.

#### P184 HIV‐related stigma and its relationship to quality of life among people living with HIV enrolled in the Swiss HIV Cohort Study

J Damas^1^, A Justice^2^, D Satre^3^, M Silverberg^4^, J Sterne^5^, A Wrona^6^, K McGinnis^6^, F Kidwai‐Khan^6^, E Kampouri^1^, D Jackson‐Perry^1^, D Braun^7^, M Cavassini^8^, K Darling ^8^

^1^Infectious Diseases, Lausanne University Hospital, Lausanne, Switzerland; ^2^Medicine, Veterans Administration Healthcare System, Yale University Schools of Medicine and Public Health, New Haven, CT, USA; ^3^UCSF Weill Institute for Neurosciences, Kaiser Permanente Northern California, Oakland, CA, USA; ^4^Research Division, Kaiser Permanente Northern California, Oakland, CA, USA; ^5^Population Health Sciences, University of Bristol Medical School, Bristol, UK; ^6^US Department of Veterans Affairs, VA Connecticut Healthcare System, New Haven, CT, USA; ^7^Division of Infectious Diseases and Hospital Epidemiology, University Hospital Zurich, University of Zurich, Zurich, Switzerland; ^8^Infectious Diseases, Lausanne University Hospital, University of Lausanne, Lausanne, Switzerland

**Background**: In a recent cross‐sectional study among 5563 Swiss HIV Cohort Study (SHCS) participants, we observed that HIV‐related stigma is highly prevalent among people living with HIV (PLWHIV).

**Methods**: We examined associations between HIV‐related stigma and quality of life based on pooled data from 395 PLWHIV participating in both the SHCS stigma study and the Medications, Alcohol, and Substance use in HIV (MASH) study at Lausanne University Hospital, Switzerland. Using validated scores, we sought associations between stigma (12‐item HIV‐stigma scale [1]) and quality of life (SF‐12), depression (PHQ‐9), anxiety (GAD‐2), alcohol consumption (AUDIT‐C), smoking and substance use. Univariable and multivariable linear regression analyses were performed.

**Results**: A total of 367 PLWHIV were included: median age: 51 years (IQR 41 to 59); 116 female (31.6%); 89 Black (24.3%); 347 (94.6%) with undetectable plasma HIV‐1 RNA; 66 (18%) on antidepressant or anxiety treatment; all had at least mandatory schooling. Among Black individuals, 61% reported frequent HIV status non‐disclosure, and 75% were receiving state‐run health insurance. HIV‐stigma scale Cronbach‐alpha was 0.8. In univariable analyses, lower mean stigma scores were associated with age (‐0.0048 per year [95% CI ‐0.0094 to ‐0.0002]; p = 0.04), better general health (‐0.01 per t‐score [‐0.01 to ‐0.0008]; p = 0.02), higher versus mandatory education (‐0.26 [‐0.33 to ‐0.03]; p = 0.001), and active involvement versus being uninvolved in HIV treatment decisions (‐0.22; [‐0.37 to ‐0.07]; p = 0.04). Being Black (0.51; CI 0.39 to 0.038; p < 0.001) and having depression were associated with increased mean stigma scores (0.19; CI 0.03 to 0.45; p = 0.02) in comparison to White and non‐depressed participants, respectively. In multivariable analyses, being Black remained significantly associated with higher stigma scores (0.38; CI 0.22 to 0.54, p < 0.001) (Figure 1).

**Figure:**
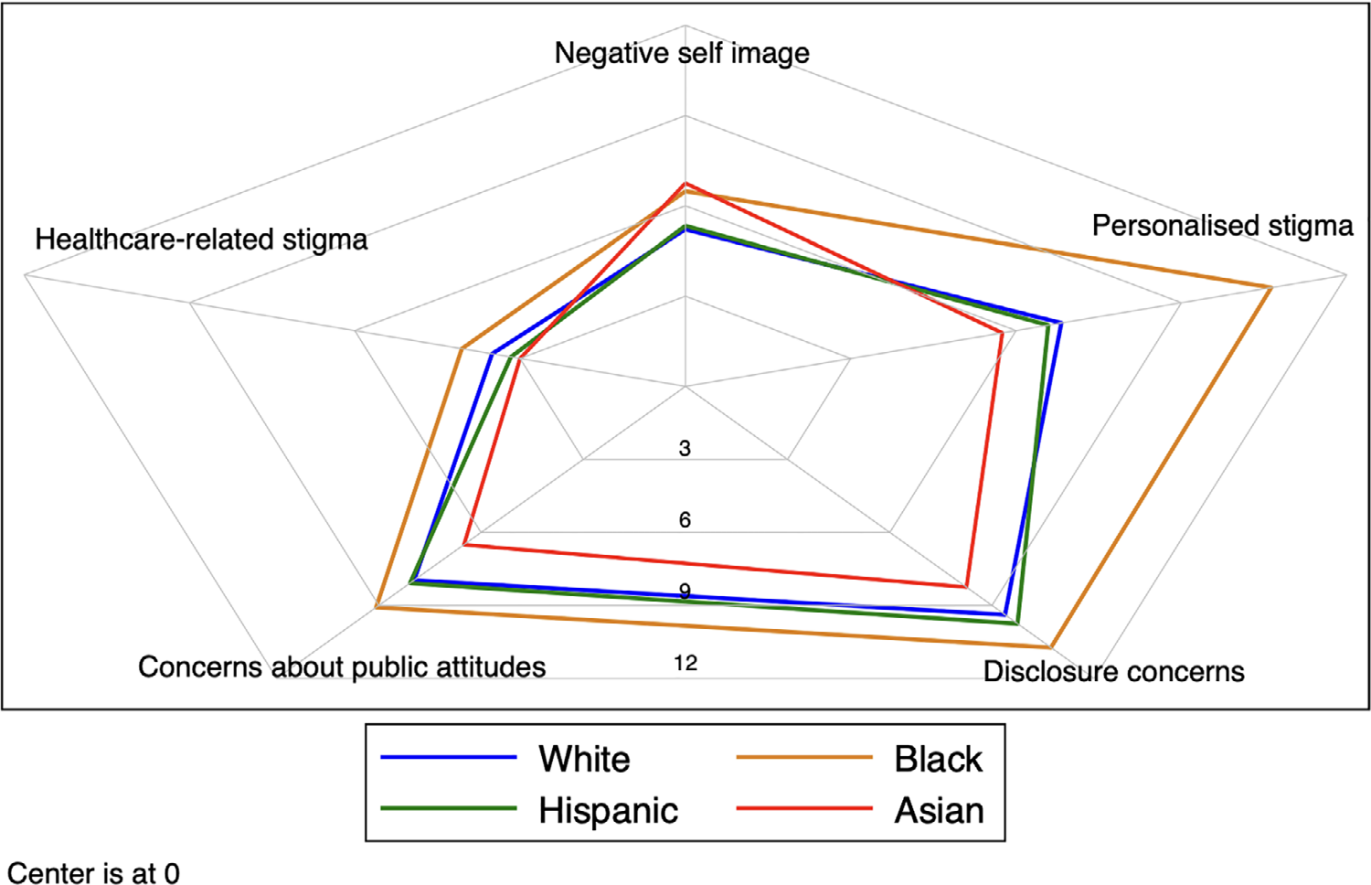
**Abstract P184 – Figure 1**. Radar plot of mean HIV‐stigma subscale scores by race/ethnicity.

**Conclusions**: In this sample of well‐treated and educated PLWHIV, being Black was the main factor associated with higher HIV‐stigma scores, suggesting intersectional stigma based on race, economic status and social attitudes to HIV. Associations between stigma and depression may have been masked by antidepressant treatment. Finally, we observed a trend of better general health and feeling empowered (shared decision‐making with healthcare personnel) being associated with reduced stigma scores.

**Reference**

1. Reinius M, Wettergren L, Wiklander M, Svedhem V, Ekström AM, Eriksson LE. Development of a 12‐item short version of the HIV stigma scale. Health Qual Life Outcomes. 2017;15:115.

#### P185 Increasing incidence of syphilis among people living with HIV in Croatia during the COVID‐19 years 2020 and 2021

J Begovac ^1^, V Romih Pintar^2^, L Mocibob^2^, N Vrsaljko^2^, N Bogdanic^2^, S Zekan^1^, A Atelj^2^, D Lukas^2^, O Dakovic Rode^3^

^1^Department of Infectious Diseases, School of Medicine University of Zagreb, Zagreb, Croatia; ^2^HIV Department, University Hospital for Infectious Diseases, Zagreb, Croatia; ^3^Virology Department, University Hospital for Infectious Diseases, Zagreb, Croatia

**Background**: Croatia has a low‐level HIV epidemic, men who have sex with men (MSM) are mainly affected [1]. HIV care is centralised, all people living with HIV (PLWHIV) are treated at the University Hospital for Infectious Diseases (UHID) in Zagreb [2]. We assessed temporal trends and associated risk factors for newly diagnosed syphilis in PLWHIV in the period 2018 to 2021.

**Materials and methods**: Incident syphilis infection occurring in PLWHIV were reviewed from the electronic database at UHID. New syphilis cases were defined based on a combination of established laboratory parameters as well as clinical diagnoses. All adults (≥18 years) followed at UHID for at least 1 year with at least one contact between 1 January 2018 and 31 December 2021 were included in this study (N = 1315). The observation period ended with the last date of contact or 31 December 2021. We calculated the annual incidence density of syphilis diagnoses and performed Poisson GEE regression analysis for factors related to new syphilis diagnoses and testing.

**Table:** 

**Abstract P185 – Table 1. **Main characteristics of people living with HIV with and without a new diagnosis of syphilis and the rate of syphilis in selected populations. Values are frequencies with percentages and median with first and third quartile. Rates are presented per 100 person‐years (PY) with 95% confidence intervals (95% CI).					
	Syphilis No (N = 1104)	Yes (N = 211)	Total (N = 1315)	p‐value	Syphilis rate per 100 PY and 95% CI
Sex at birth				<0.001	
Female	128 (11.6)	0 (0)	128 (9.8)		
Male	973 (88.4)	211 (100.0)	1184 (90.2)		6.4 (5.6 to 7.2)
Age, years				<0.001	
<35	261 (23.6)	73 (34.6)	334 (25.4)		8.5 (6.7 to 10.6)
35 to 50	520 (47.1 )	103 (48.8 )	623 (47.4)		5.7 (4.7 to 6.9)
>50	323 (29.3 )	35 (16.6)	358 (27.2)		3.5 (2.4 to 5.0)
Mode of transmission				<0.001	
MSM	775 (70.2)	201 (95.3)	976 (74.2)		7.4 (6.5 to 8.5)
Heterosexual	254 (23.0)	5 (2.4)	259 (19.7)		0.6 (0.3 to 1.6)
Other/unknown	75 (6.8)	5 (2.4)	80 (6.1)		1.8 (0.8 to 4.1)
Living in Zagreb				<0.001	
No	633 (57.3)	81 (38.4)	714 (54.3)		4.2 (3.3 to 5.3)
Yes	471 (42.7)	130 (61.6)	601 (45.7)		7.5 (6.4 to 8.9)
Had clinical AIDS				<0.001	
No	816 (73.9)	184 (87.2)	1000 (76.0)		
Yes	288 (26.1)	27 (12.8)	315 (24.0)		
Antiretroviral therapy				0.39	
After baseline	77 (7.0)	20 (9.5)	97 (7.4)		
Before baseline	1018 (92.2)	190 (90.0)	1208 (91.9)		
No ART	9 (0.8)	1 (0.5)	10 (0.8)		
Baseline HIV‐1 RNA				0.005	
≥50 copies/mL	251 (23.1)	68 (32.2)	319 (24.6)		
<50 copies/mL	835 (76.9)	143 (67.8)	978 (75.4)		
Baseline CD4 cell count per mm^3^				0.28	
>500	628 (60.3)	135 (64.3)	763 (60.9)		
≤500	414 (39.7)	75 (35.7)	489 (39.1)		
Duration of HIV infection, years	5.5 (1.4 to 12.0)	2.8 (0.3 to 6.5)	5.0 (1.2 to 11.3)	<0.001	
Antibody to hepatitis C				0.3	
Negative	1037 (93.9)	202 (95.7)	1239 (94.2)		
Positive	67 (6.1)	9 (4.3)	76 (5.8)		
Hepatitis B surface antigen				0.67	
Negative	1049 (95.0)	199 (94.3)	1248 (94.9)		
Positive	55 (5.0)	12 (5.7)	67 (5.1)		

**Figure:**
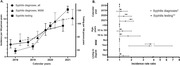
**Abstract P185 – Figure 1**. Incidence of syphilis diagnoses in the whole study population and in MSM, and testing per 100 person‐years of follow up in the period 2018 to 2021 (A), and factors related to syphilis incidence and testing on multivariable analysis (B). * Adjusted also for syphilis testing; ** adjusted also for syphilis diagnoses.

**Results**: Of 1315 PLWHIV, 1184 (90.2%) were men, median age was 42.0 (34.9 to 51.1) years (Table 1). Two hundred and eleven PLWHIV had a total of 271 new syphilis diagnoses during 4740.1 years of follow‐up. Of 271 events, 128 (47.2%) were first syphilis diagnoses, and 162 (59.8%) had symptomatic syphilis (secondary, n = 97). The overall syphilis incidence was 5.7 (95% CI 5.0 to 6.6) per 100 person‐years (PY), it increased from 3.2 (95% CI 2.3 to 4.5) to 8.2 (95% CI 6.8 to 10.0) per 100 PY during 2018 to 2021 (Figure 1). On adjusted analysis (adjusted for the number of tests and other variables) younger age, calendar year, MSM risk was associated with syphilis, the association with living in Zagreb was less pronounced (Figure 1). The rate of syphilis testing doubled between 2018 (50.4, 95% CI 56.5 to 64.6 per 100 PY) and 2021 (123.0, 95% CI 118.1 to 128.1 per 100 PY). Factors related to the frequency of syphilis testing were: age, calendar year, MSM risk, and living in Zagreb (Figure 1).

**Conclusions**: During the COVID‐19 epidemic years, the incidence rate of syphilis was more than 2 times higher than in previous years reflecting ongoing sexual risk in predominantly younger MSM and highlighting the need for enhanced prevention interventions.

**References**

1. European Centre for Disease Prevention and Control/WHO Regional Office for Europe. HIV/AIDS surveillance in Europe 2021 – 2020 data. Stockholm: ECDC; 2021.

2. Bogdanić N, Bendig L, Lukas D, Zekan Š, Begovac J. Timeliness of antiretroviral therapy initiation in the era before universal treatment. Sci Rep. 2021:11:10508.

#### P186 Pancreatic cancer in people living with HIV: an emerging concern?

S Chawki ^1^, N Lourenço^2^, G Hamet^3^, A Brun^4^, O Bouchaud^5^, J Bottero^6^, P Sellier^1^, J Molina^1^

^1^Infectious Disease, Saint Louis/Lariboisière ‐ Assistance Publique Hôpitaux de Paris, Paris, France; ^2^Gastroenterology, Saint Louis ‐ Assistance Publique Hôpitaux de Paris, Paris, France; ^3^COREVIH Ile de France EST, Saint Louis/Lariboisière ‐ Assistance Publique Hôpitaux de Paris, Paris, France; ^4^COREVIH Ile de France EST, Saint Louis‐ Assistance Publique Hôpitaux de Paris, Paris, France; ^5^Infectious Disease, Avicenne ‐ Assistance Publique Hôpitaux de Paris, Bobigny, France; ^6^Infectious Disease, Jean Verdier ‐ Assistance Publique Hôpitaux de Paris, Bondy, France

**Background**: Thanks to antiretroviral drug therapy (ART) life expectancy in people living with HIV (PLWHIV) continues to increase. Consequently, cancer is now one the leading causes of death in PLWHIV, surpassing opportunistic infections. Pancreatic cancer (PC) is one of the leading causes of oncological death in high‐income countries. Furthermore, pancreatic neoplasms are on the rise. Data on pancreatic cancer in PLWHIV are scarce but tend to show that PLWHIV have a high incidence with aggressive tumours. We aimed to estimate the incidence and to assess the risk factors of pancreatic cancer in a cohort of PLWHIV.

**Materials and methods**: A case‐control study was designed using data extracted from the COREVIH Ile‐de‐France‐Est database. Each case (PLWHIV and pancreatic cancer) was matched (on age, gender and duration of HIV infection) to four controls. We collected data on patient demographics, treatment history, and immuno‐virological status.

**Results**: From 2009 to 2020, in six hospitals in the Paris region, 25 cases were identified from the database, and 24 were included in the case control study with 96 controls. Incidence for PC was estimated at 28 cases (95% CI 5 to 108) per 100 000 person per year. Median age was 57 years (IQR 51 to 58) at cancer diagnosis (Table 1). Twenty‐two cases were male (88%). Median CD4+ T cell count at cancer diagnosis was 641/mm^3^ (IQR 352 to 840) and nadir CD4+ T cell count was 208 (IQR 102 to 387). Twelve (48%) had metastasis on diagnosis. Death rate among cases was 91% (n = 18). Median time to death was 11 months (IQR 1 to 21).  No statistically significant risk factors for PC were found in the case‐control analysis but there was a trend towards an increased risk with the duration of emtricitabine, abacavir, tenofovir, or atazanavir use (p = 0.08; 0.11; 0.12 and 0.16, respectively).

**Conclusions**: Although pancreatic cancer remains a rare occurrence in PLWHIV, it seems to occur at a younger age and to be aggressive. A larger study is needed to confirm these trends and identify risk factors for PC in patients PLWHIV.

**Table:** 

**Abstract P186 – Table 1**. Characteristics of the 25 cases of PC among PLWHIV.	
Characterististic	n or median (% or IQR)
Age (years)	57 (51 to 68)
HIV duration of evolution (years)	17 (3 to 22)
Sex (male)	22 (88)
Alcohol consumption (current or past)	2 (10)
Tobacco consumption (current or past)	8 (38)
Mode of HIV transmission	
MSM	12 (48)
Heterosexual	8 (32)
Blood transfusion	2 (8)
Unknown	3 (12)
CD4+ T cells nadir	208 (102 to 387)
History of opportunistic infection	4 (33)
Peak plasma viral load (log copies/mL)	10 (5 to 12)
Duration of undetectability of plasma viral load before cancer (years)	7 (5 to 7)
CD4+ T‐cells at diagnosis of cancer	641 (352 to 840)
Metastasis at diagnosis of cancer	12 (48)
ART received:	
Protease inhibitors	16 (64)
Nucleoside inhibitors	21 (84)
Non‐nucleoside inhibitors	10 (40)
Integrase inhibitors	14 (56)
Outcome	
Death	19 (91)
Survival (months)	11 (1 to 21)

#### P187 Prevalence of Mycoplasma genitalium infection and resistance‐associated mutations to macrolides and fluoroquinolones among people living with HIV and people on pre‐exposure prophylaxis in Taiwan

Y Su ^1^, L Su^1^, W Liu^1^, H Sun^1^, W Liu^1^, K Lin^1^, Y Huang^1^, G Chen^2^, S Chang^3^, C Hung^1^

^1^Department of Internal Medicine, National Taiwan University Hospital, Taipei, Taiwan; ^2^Department of Internal Medicine, Min‐Sheng General Hospital, Taoyuan, Taiwan; ^3^Clinical Laboratory Sciences/Medical Biotechnology, National Taiwan University College of Medicine, Taipei, Taiwan

**Background**: *Mycoplasma genitalium* (MG) is an emerging aetiology of sexually transmitted infection (STI) with increasing trends of antimicrobial resistance. Data are limited among at‐risk populations because molecular diagnosis of MG infection is infrequently performed in individuals seeking counselling and treatment of STIs in Asia‐Pacific region. This study aimed to examine the prevalence of MG infection and its genotypic resistance to macrolides and fluoroquinolones among people living with HIV (PLWHIV) seeking STI care and people seeking pre‐exposure prophylaxis (PrEP) for HIV at a university hospital in Taiwan.

**Materials and methods**: Between August 2021 and June 2022, PLWHIV presenting with STIs and PrEP users were enrolled and clinical specimens were collected from the rectum, urethra, and oral cavity for identification of seven pathogens (MG, *Chlamydia trachomatis*, *Neisseria gonorrhoeae*, *Trichomonas vaginalis*, *M. hominis*, *Ureaplasma urealyticum*, and *U. parvum*) with the use of multiplex PCR assay (Allplex™ STI Essential Assay, Seegene Inc., South Korea). Tests for rapid plasma reagin (RPR) titer and HCV RNA were performed. Resistance‐associated mutations of MG to macrolides were examined in region V of the 23S rRNA gene (A2058 and A2059) and that to fluoroquinolones were examined in *parC* gene.

**Results**: During the study period, 392 participants were enrolled, 224 PLWHIV and 168 PrEP users. PrEP users were significantly younger than PLWHIV (median, 30.1 vs 35.8 years) and less likely to have an RPR titer of 4 or greater (14.3% vs 74.1%) (both p < 0.001). The overall prevalence of MG infection was 8.4% (n = 33) (95% CI 5.6 to 11.2%): 6.4% in rectal swab, 1.8% in urethral swab, and 0.2% in oral rinse specimens. The rate of co‐infection with other STIs was 69.7% among MG‐infected participants. The prevalence of MG infection was similar between PLWHIV and PrEP users (9.8% vs 6.5%, p = 0.33). The overall prevalence of resistance‐associated mutations of MG to macrolides only was 6.3%, fluoroquinolones only 3.1%, and both macrolides and fluoroquinolones 9.4%.

**Conclusions**: While the rate of MG infection is currently low among PLWHIV seeking STI care and PrEP users, surveillance studies to follow the trends of antimicrobial resistance are warranted to inform the treatment recommendations for MG infection.

#### P188 Identification of quality‐of‐life alterations in PLWHIV in routine practice: benefit of the OCTAVE self‐administered questionnaire

L Slama ^1^, F Bastides^2^, O Robineau^3^, J Leporrier^4^, G Barriere^5^, E Estrabaud^5^, V Pourcher^6^, E Piet^7^

^1^Department of Immunology and Infectious Diseases, Hotel‐Dieu Assistance Publique des Hôpitaux de Paris, Paris, France; ^2^Department of Infectious Diseases, CHRU, Tours, France; ^3^Department of Infectious Diseases, Hôpital Dron, Tourcoing, France; ^4^Department of Infectious Diseases, Hôpital Necker‐Enfants Malades, Paris, France; ^5^Medical Department, Gilead France, Paris, France; ^6^Department of Infectious Diseases, Hôpital de la Pitié‐Salpêtrière, Assistance Publique des Hôpitaux de Paris, Paris, France; ^7^Department of Infectious Diseases, Centre Hospitalier Annecy Genevois, Epagny Metz‐Tessy, France

**Background**: Improving quality of life (QoL) of people living with HIV (PLWHIV) is a cardinal objective of care [1]. Existing QoL questionnaires, designed for clinical research, may not be well suited in daily clinical practice.

**Material and methods**: We developed OCTAVE, a didactic self‐administered questionnaire, to screen QoL alterations in daily clinical practice. The objective of our survey was to assess the contribution of OCTAVE to unravel unknown QoL impairments and modifications of follow‐up. Physicians were asked to administer the questionnaire to 10 of their virologically suppressed PLWHIV. After each visit, physicians had to note QoL alterations discovered and modifications of follow‐up for each patient.

**Results**: OCTAVE includes 42 optional questions divided into 8 items: Treatment, Mental & Physical Health, Sleep, Sexuality, Emotional Well‐being, Comorbidities, Social & Professional Life. Between September 2021 and April 2022, 39 out of 244 solicited physicians took part in the survey, mostly infectious disease specialists (77%), 56% had more than 20 years’ experience in PLWHIV follow‐up. A total of 382 PLWHIV were enrolled and completed OCTAVE. Among them 70% were men, the median age was 52 years, 83% declared French as their mother tongue. The median duration of HIV infection was 15 years (SD 9.76), and the transmission routes were heterosexual, homosexual and drug use for respectively 48%, 40% and 6% of them. QoL alterations, of which the physician had no previous knowledge or awareness, were found in 44% of PLWHIV who completed. Alterations were mainly related to sleep disorders (42%) and sex life (40%). Modifications of follow‐up to improve QoL were reported for 70% of PLWHIV. Among most common interventions: 28% of PLWHIV were referred to a gynecologist/proctologist, 26% to their general practitioner or another specialist; 22% to adapted physical activity, 17% to a psychologist/psychiatrist, 16% for a closer follow‐up visit. Intentions to modify antiretroviral regimen occurred in 14% of the PLWHIV.

**Conclusion**: The routine use of OCTAVE, a didactic self‐administered 8‐items questionnaire, revealed unknown QoL alterations and allowed positive interventions to improve PLWHIV 'real' QoL and global care.

**Reference**

1. Lazarus JV, Safreed‐Harmon K, Barton SE, Costagliola D, Dedes N, Del Amo Valero J, et al. Beyond viral suppression of HIV ‐ the new quality of life frontier. BMC Med. 2016;14:94.

#### P189 Quality of sleep in people living with HIV (PLWHIV) in the era of highly active antiretroviral treatment (HAART)

V Petrakis, P Panagopoulos, N Papanas, G Trypsianis, P Steiropoulos, D Papazoglou

2nd University Department of Internal Medicine, University General Hospital Alexandroupolis, Alexandroupolis, Greece

**Background**: Although HIV infection has become a chronic disease with a significant improvement in life expectancy, people living with HIV (PLWHIV) often develop disorders that affect their quality of life [1]. Sleep disturbances could occur in all stages of infection, eventually in advanced disease [2]. They can lead to impairment of concentration and memory, fatigue, increased risk for psychiatric disorders and cardiovascular morbidity, and reduced adherence to treatment [3]. The aim of the present study is to evaluate the quality of sleep in PLWHIV monitored at the HIV Unit of the University General Hospital of Evros (Greece), the possible risk factors and association with antiretroviral treatment.

**Materials and methods**: Patients completed questionnaires for the possible presence of restless legs syndrome, the Epworth Scale, the Athens Sleep Scale, the Fatigue Severity Scale (FSS Questionnaire), the Hospital Anxiety and Depression Scale (HADS), the Sleep Quality Scale MOS, the STOP‐Bang questionnaire for obstructive sleep apnoea, the Pittsburgh Sleep Quality Index  and the International Physical Activity Questionnaire [4‐14]. Demographic and anthropometric characteristics, antiretroviral regimen, stage of HIV infection, coinfections and comorbidities were recorded. Statistical analysis was performed using the Statistical Package for Social Sciences (SPSS) version 19.0.

**Results**: A total of 154 patients, 120 males, were included in the study. The percentage of patients diagnosed with restless legs syndrome was 26.6% and more than half suffer from insomnia. Obstructive sleep apnoea was documented in 35% of patients. The results of the questionnaires indicate that 31.16% experience daytime sleepiness, 27.9% fatigue, 54.54% symptoms of anxiety and 46.1% depression. The sleep disturbances were significantly associated with detectable viral load, lower CD4 cell count, lower number of years after diagnosis and HAART initiation, presence of multi‐comorbidities and limited physical activity (p < 0.001). Sleep disorders were not proven to be associated with antiretroviral regimen.

**Conclusions**: Sleep disturbances are common in people living with HIV infection affecting their quality of life. The present study has shown that sleep disorders are not associated with new antiretroviral regimens but increase the risk of depression and anxiety and limit the daily physical activity.

**References**

1. Gueler A, Moser A, Calmy A, Günthard HF, Bernasconi E, Furrer H, et al. Life expectancy in HIV positive persons in Switzerland: matched comparison with general population. AIDS. 2017;31:427‐36.

2. Norman SE, Resnick L, Cohn MA, Duara R, Herbst J, Berger JR. Sleep disturbances in HIV‐seropositive patients. JAMA. 1988;260:922.

3. Rubinstein ML, Selwyn PA. High prevalence of insomnia in an outpatient population with HIV infection. J Acquir Immune Defic Syndr Hum Retrovirol. 1998;19:260‐5.

4. Saberi P, Neilands TB, Johnson MO. Quality of sleep: associations with antiretroviral nonadherence. AIDS Patient Care STDS. 2011;25:517‐24.

5. Tsara V, Serasli E, Amfilochiou A, Constantinidis T, Christaki P. Greek version of the Epworth Sleepiness Scale. Sleep Breath. 2004;8:91‐5.

6. Bakalidou D, Skordilis EK, Giannopoulos S, Stamboulis E, Voumvourakis K. Validity and reliability of the FSS in Greek MS patients. Springerplus. 2013;2:304.

7. Soldatos CR, Dikeos DG, Paparrigopoulos TJ. Athens Insomnia Scale: validation of an instrument based on ICD‐10 criteria. J Psychosom Res. 2000;48:555‐60.

8. Michopoulos I, Douzenis A, Kalkavoura C, Christodoulou C, Michalopoulou P, Kalemi G, et al. Hospital Anxiety and Depression Scale (HADS): validation in a Greek general hospital sample. Ann Gen Psychiatry. 2008;7:4.

9. Bougia MK, Oikonomou N‐T, Ntafouli M, Ilias I, Gourgoulianis K, Vagiakis E, et al. Investigation of the reliability of the Medical Outcomes Study (MOS) Sleep Scale in Greek patients with sleep‐related breathing disorders. Ann Transl Med. 2016;4:AB023.

10. Allen RP, Picchietti D, Hening WA, Trenkwalder C, Walters AS, Montplaisi J, et al. Restless legs syndrome: diagnostic criteria, special considerations, and epidemiology. A report from the restless legs syndrome diagnosis and epidemiology workshop at the National Institutes of Health. Sleep Med. 2003;4:101‐19.

11. Walters AS, LeBrocq C, Dhar A, Hening W, Rosen R, Allen RP, et al. Validation of the International Restless Legs Syndrome Study Group rating scale for restless legs syndrome. Sleep Med. 2003;4:121‐32.

12. Miskedaki A, Bacopoulou F, Vlachakis D, Artemiadis A, Chrousos GP, Darviri C. Validation of the STOP‐Bang questionnaire in Greek patients suffering from obstructive sleep apnea. Adv Exp Med Biol. 2021;1337:77‐82.

13. Kotronoulas GC, Papadopoulou CN, Papapetrou A, Patiraki E. Psychometric evaluation and feasibility of the Greek Pittsburgh Sleep Quality Index (GR‐PSQI) in patients with cancer receiving chemotherapy. Support Care Cancer. 2011;19:1831‐40.

14. Papathanasiou G, Georgoudis G, Georgakopoulos D, Katsouras C, Kalfakakou V, Evangelou A. Criterion‐related validity of the short International Physical Activity Questionnaire against exercise capacity in young adults. Eur J Cardiovasc Prev Rehabil. 2010;17:380‐6.

#### P190 Oropharyngeal infection due to HPV in people living with HIV (PLHIV)

C Hidalgo‐Tenorio ^1^, L Benitez Cejas^2^, I Calle^3^, R Moya^3^, P Gomez Ronquillo^1^, J Lopez Hidalgo^4^, J Rodriguez^5^

^1^Infectious Diseases, Hospital Universitario Virgen de las Nieves, Granada, Spain; ^2^Facultad de Medicina, Universidad de Granada, Granada, Spain; ^3^Internal Medicine, Hospital Universitario Virgen de las Nieves, Granada, Spain; ^4^Pathology Service, Hospital Universitario Virgen de las Nieves, Granada, Spain; ^5^Microbiology Service, Hospital Universitario Virgen de las Nieves, Granada, Spain

**Background**: Antiretroviral therapy (ART) has increased life expectancy of PLHIV, and therefore the risk of non‐AIDS‐defining diseases. The most common non‐AIDS‐defining cancer in PLHIV, especially in men who have sex with men (MSM), is anal cancer related to human papillomavirus (HPV) infection. So far, there have been little data on infection and dysplasia in oropharyngeal mucosa in PLHIV.

**Material and methods**: This is a prospective study carried out between November 2021 and April 2022, that included PLHIV who belonged to a HPV‐associated anal and genital cancers screening programme. Data were gathered at baseline visit on sexual habits, CD4/CD8 cell/counts, HIV viral load, and the results of anal and cervical cytology (Thin Prep^®^ Pap Test); oral, anal and cervical HPV PCR genotyping (Linear Array HPV Genotyping Test), high‐resolution anoscopy (Zeiss 150 fc © colposcope) and colposcopy in case of women (WLHIV).

**Results**: One hundred and forty‐two PLHIV were included with a mean age of 44 years, 15.5% were WLHIV, 44 (31%) with a history of AIDS, 100% receiving ART, and one (0.7%) with virological failure. Thirty‐one (21.8%) had received completed HPV vaccine. The patients with a history of dysplasia associated with HPV infection were: one (0.7%) had epidermoid cancer of the tonsil; four (18.2%) WLHIV had CIN1 y 8 (36.4%) CIN2‐3/ C. in situ; and finally, 84 (59.2%) had LSIL (AIN1), and 30 (21.1%) HSIL (AIN2‐3). Sixteen (11.2%) had oropharyngeal HPV infection and the most frequent genotypes were 33, 59 and 68 (high risk), and one (0.75) had a wart in oral mucosa. Twelve (54.5%) of WLHIV had genital HPV, and two (9%) had coincident orogenital HPV infection; the most frequent genotype was HPV‐44 (22.5%); and one (4.5%) had CIN1. 76.1% PLHIV had anal HPV infection, the most frequent HPV were 44 (16.9%), 16 (14.8%) and 52 (13.4%); 32 (22.5%) had LSIL (AIN1) and one (0.7%) HSIL (AIN2/3); and 14 (9.9%) had oral and anal HPV infection, simultaneously. Finally, the factor associated with the risk of oropharyngeal HPV infection was a concomitant syphilis (HR 10.45, 95% CI 1981 to 55 148).

**Conclusions**: The prevalence of HPV infection and dysplasia in the oropharyngeal mucosa were lower than those in the female genital mucosa and anal canal of men and women living with HIV. Syphilis is a marker of oropharyngeal HPV infection.

#### P191 Detection of *Treponema pallidum* DNA among men who have sex with men who presented with early syphilis

T Wu ^1^, L Su^1^, S Chang^2^, H Sun^1^, W Liu^1^, Y Huang^1^, W Liu^1^, K Lin^1^, C Hung^1^

^1^Internal Medicine, National Taiwan University Hospital, Taipei City, Taiwan; ^2^Laboratory Medicine, National Taiwan University Hospital, Taipei City, Taiwan

**Background**: Early diagnosis and disease activity measurement of syphilis remain challenging with the use of serological tests and clinical assessment. The study aimed to investigate the presence of *Treponema pallidum* DNA (TP‐DNA) in various sample types and syphilis stages among men who have sex with men (MSM).

**Materials and methods**: The prospective study was conducted to include adult MSM seeking care for sexually transmitted infections during September 2021 and June 2022. Serological tests were routinely performed to confirm the diagnosis of syphilis. Clinical samples, including oral rinse, rectal swab, and urethral swab, were collected from participants and tested for TP‐DNA with the use of PCR assay targeting the 47 kDa gene. The cycle threshold (Ct) value of polymerase chain reaction (PCR) assay was determined, and the result was considered positive with a Ct value <38.

**Results**: During the study period, 285 men were included, 145 participants with early syphilis (14 primary syphilis, 52 secondary syphilis, and 79 early latent syphilis) and 101 without serological and clinical diagnosis of syphilis (Table 1). TP‐DNA was detected in at least one study sample in 49% (71/145) of participants with early syphilis and 4.0% (4/101) participants without syphilis (p < 0.001), resulting in a specificity of 96.0% and sensitivity 49.0%. Of participants with early syphilis, the detection rate of TP‐DNA was highest in oral rinse (41%), compared with 28% in rectal swabs and 18% in urethral swabs. TP‐DNA was most frequently detected in participants with secondary syphilis (73%), followed by primary and early latent stage (64% and 30%, respectively). While the detection rate of TP‐DNA was higher in participants with rapid plasma reagin (RPR) titers of 1:32 or higher compared with those with lower titers (55% vs 17%, p = 0.001), a negative association between Ct values of PCR assays and RPR titers was also found (p < 0.001) (Figure 1).

**Table:** 

**Abstract P191 – Table 1**. Clinical characteristics and TP‐DNA detection of included participants. Data are median [IQR], n (%), or n/N (%).					
	Early syphilis (n = 145)	Primary syphilis (n = 14)	Secondary syphilis (n = 52)	Early latent syphilis (n = 79)	No early syphilis (n = 101)
Age, years [IQR]	36 [32 to 43]	33 [30 to 43]	33 [29 to 41]	38 [34 to 44]	33 [30 to 40]
MSM	138 (99%)	12 (92%)	52 (100%)	74 (99%)	88 (97%)
>5 sexual partners in past 3 months	18 (15%)	2 (17%)	10 (23%)	6 (9%)	6 (7%)
HIV‐positive	134 (92%)	12 (86%)	46 (88%)	76 (96%)	62 (61%)
CD4 count, cells per μL	619 [490 to 811]	661 [530 to 823]	641 [461 to 806]	605 [503 to 794]	578 [423 to 798]
On ART	130/134 (97%)	12/12 (100%)	43/46 (93%)	75/76 (99%)	59/62 (95%)
HIV‐negative	11 (8%)	2 (14%)	6 (12%)	3 (4%)	39 (39%)
On PrEP	2/11 (18%)	1/2 (50%)	1/6 (17%)	0/3 (0%)	24/39 (62%)
Not on PrEP	9/11 (82%)	1/2 (50%)	5/6 (83%)	3/3 (100%)	15/39 (38%)
RPR [IQR]	128 [64 to 256]	256 [64 to 256]	256 [128 to 512]	128 [32 to 256]	−
TP‐DNA positivity					
Oral rinse	60 (41%)	7 (50%)	35 (67%)	18 (23%)	4 (4%)
Rectal swab	40 (28%)	4 (29%)	26 (50%)	10 (13%)	1 (1%)
Urethral swab	25 (17%)	4 (29%)	16 (31%)	5 (6%)	0 (0%)
Any sample	71 (49%)	9 (64%)	38 (73%)	24 (30%)	4 (4%)
Ct value of PCR test					
Oral rinse	31.78 [29.68 to 33.76]	31.43 [30.14 to 32.85]	31.24 [29.41 to 33.40]	32.74 [32.01 to 33.80]	33.70 [‐]
Rectal swab	30.91 [27.90 to 33.84]	32.47 [‐]	30.79 [28.05 to 33.70]	31.00 [27.23 to 34.60]	37.93 [‐]
Urethral swab	35.53 [33.51 to 36.99]	34.62 [‐]	34.94 [33.47‐37.02]	35.93	−

Ct, cycle threshold; PrEP, pre‐exposure prophylaxis; RPR, rapid plasma reagin; TP‐DNA, *Treponema pallidum* DNA.

 

**Figure:**
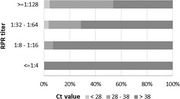
**Abstract P191 – Figure 1**. Distribution of Ct values of *T. pallidum* PCR detected from oral rinse by serum RPR titer in participants with early syphilis. Ct, cycle threshold; RPR, rapid plasma reagin.

**Conclusions**: The frequent detection of *T. pallidum* shedding in the mouth and anus among MSM with early syphilis supports the use of TP‐DNA in early diagnosis. The higher detection rate and lower Ct value of TP‐DNA in those with secondary syphilis and higher RPR titers may indicate that TP‐DNA load predicts transmissibility.

#### P192 A qualitative exploration of HIV stigma and its impact on accessing healthcare

D Jackson‐Perry^1^, C Le Saux^2^, I Gilles^2^, C Storari^2^, O Keserue Pittet^2^, O Nawej Tshikung^3^, E Cart^2^, M Cavassini^1^, K Darling ^1^

^1^Infectious Diseases Service, Lausanne University Hospital, Lausanne, Switzerland; ^2^Centre Universitaire de Médecine Générale et Santé Publique, Unisanté, Lausanne, Switzerland; ^3^Division of Infectious Diseases, University Hospital Geneva, Geneva, Switzerland

**Background**: Between March 2020 and January 2021, we led a cross‐sectional quantitative study nested within the Swiss HIV Cohort Study to examine the prevalence of HIV stigma among 5563 people living with HIV (PLWHIV). Following this quantitative study, we have undertaken a qualitative study to explore the impact of HIV stigma on PLWHIV accessing and receiving healthcare.

**Methods**: Between 1 March and 31 May 2022, PLWHIV followed up at Lausanne University Hospital participated in an observational study using qualitative methods within the conceptual framework of the grounded theory. Exclusion criteria were less than conversation‐level French, neurocognitive impairment and previous/current psychiatric history including depression. Consenting participants attended a confidential one‐to‐one semi‐structured interview of up to 90 minutes’ duration with a social science researcher. Interviews were recorded then transcribed. Data analysis was based on the grounded theory methodology with four steps: broadly coding data, establishing a coding system, identifying and organising categories to construct a theory of how PLWHIV live with and navigate HIV stigma. Analyses were conducted by researchers working in pairs then with the whole team. A member of the Lausanne HIV Community Council was an integral part of the research team throughout.

**Results**: Twenty PLWHIV participated. HIV stigma was experienced during interactions with healthcare professionals (HCP) outside of HIV care. Breaches of confidentiality and irrelevant questions, notably on mode of HIV acquisition, were frequently reported. While most participants felt compelled to reveal their HIV status to HCP, some developed strategies similar to those they apply in social (non‐medical) interactions to gauge whether to disclose or not. Ease of contact and trust were key factors in these strategies. Finally, participants expressed disappointment at the lack of HIV knowledge of some HCP, similar to that observed in the general public (inappropriate questions, irrational precautions) resulting in frequent changes of HCP as well as apprehension prior to new appointments.

**Conclusion**: Healthcare experiences for PLWHIV would be improved if HCP were more aware of HIV stigma. Efforts should be made to update HCP HIV knowledge and provide HIV stigma training, particularly regarding confidentiality and how to communicate with PLWHIV.

#### P193 Is HIV still a barrier to sex for people living with HIV in the UK?

C Tugulu ^1^, N Ahmed^1^, P Bullemor‐Day^2^, D Weston^2^, J Ashby^2^

^1^HIV/Sexual Health, Central and North West London NHS Foundation Trust, London, UK; ^2^Sexual Health, Central and North West London NHS Foundation Trust, London, UK

**Background**: Despite advances in HIV care and treatment, people living with HIV (PLWHIV) may still face barriers to a healthy and fulfilling sex life. We aimed to identify amongst PLWHIV attending our services, whether not being sexually active was related to HIV or to sexual difficulties.

**Methods**: All patients who attended our services between 11/07/2017 and 20/05/2022 and reported that they were not sexually active were included in this review. The electronic patient record of these PLWHIV was reviewed to ascertain demographic data and whether lack of sexual activity was related to either HIV or a sexual difficulty. This data was collated and analysed using Microsoft Excel.

**Results**: Three hundred and eight PLWHIV were identified as not currently being sexually active, of whom 25.2% (n = 74) reported not ever being sexually active since being diagnosed with HIV. Of these 47.3% (n = 35) stated this was due to HIV. A similar proportion of male and female identifying PLWHIV reported they were currently not sexually active due to HIV; 29.7% (n = 202) males and 29.1% (n = 106) females. A sexual problem was reported as the reason for no sex in 4% of males and 1.3% of females. Heterosexual identifying PLWHIV were 2.6 times more likely to not be sexually active, compared to those identifying as homosexual, 34% and 13.5%, respectively. HIV was stated as the reason for 63% of heterosexuals compared to 54% in the homosexual group, with 5% of homosexuals reporting sexual problems as the cause compared to zero heterosexuals. Data showed nearly double all Black PLWHIV not being sexually active due to HIV (31.1%), compared to all white PLWHIV (16.1%). Sexual problems were stated as the reason for abstinence in 1.2% and 6.1%, respectively.

**Conclusions**: The proportion of all Black and heterosexual PLWHIV reporting no sex due to HIV was higher compared to white British and men who have sex with men group. Our findings suggest further support to understand and address barriers for PLWHIV who would wish to have sex. A specific focus on understanding barriers to sexual activity amongst non‐white and heterosexual PLWHIV is needed.

#### P194 Effect of online education on physician knowledge and confidence regarding the impact that stigma has on health outcomes in people living with HIV

J Duffey ^1^, S Voorn^2^, C Ní Cheallaigh^3^, M Young Karris^4^, J Rockstroh^5^

^1^Clinical Strategy, Manifold Medical, Matlock, UK; ^2^Medical Education, WebMD Global LLC, New York, NY, USA; ^3^Department of Clinical Medicine, Trinity College, Dublin, Ireland; ^4^Department of Medicine, University of California San Diego, San Diego, CA, USA; ^5^Head of HIV Outpatient Clinic, University of Bonn, Bonn, Germany

**Background**: People living with HIV (PLWHIV) experience high levels of stigma which may manifest as self‐stigma, perceived stigma or external/enacted stigma. Additionally, stigma is also compounded by experiencing health inequity. Experiencing stigma has been found to impact PLWHIV antiretroviral adherence, virological failure, morbidity and mortality. We assessed whether an online independent medical education activity could improve the knowledge and confidence of HIV physicians and public health and preventive medicine specialists (PHPMS) regarding the impact that stigma has for PLWHIV and the association between stigma and other negative health outcomes.

**Materials and methods**: Educational effect was assessed using a repeated‐pairs design with pre‐/post‐assessment. Three multiple‐choice questions assessed knowledge, and one question assessed confidence. Statistical tests to assess significance included: paired samples t‐test for overall average number of correct responses and confidence. McNemar's test for individual questions and learning objectives (p < 0.05). Cohen's d estimated the effect size impact on number of correct responses (<0.20 modest, 0.20 to 0.49 small, 0.59 to 0.79 moderate, ≥0.80 large). Data were collected from 4/6/2021 to 10/14/2021.

**Results**: From a total audience of 746 physicians there were 121 assessment completers. Overall, there was a significant increase in knowledge (p < 0.001 for both) with a large impact in HIV physician knowledge gains (Cohen's d 0.82). Both HIV physicians and PHPMS reported an increase in confidence (HIV physicians 48%; PHPMS 60%) with a total average confidence shift of 66% and 79%, regarding their ability to assess stigma in their patients with HIV, respectively. Specifically, large knowledge gains were found in questions regarding the prevalence of bipolar disease in PLWHIV who experience stigma as measured by the relative change from baseline knowledge to post‐education (HIV physicians 162%; PHPMS 186%; p < 0.0001 for both).

**Conclusions**: Online medical education significantly improved physician knowledge and confidence regarding the types of stigma that PLWHIV experience and the consequences of stigma for comorbidities and for PLWHIV's mental health. Additionally, physicians who participated in the education experienced an increase in their confidence to assess stigma and the consequences of stigma for their patients living with HIV.

#### P195 Knowledge of STIs and HIV among people with and without HIV: where are we now?

A Colpani ^1^, A De Vito^1^, B Zauli^1^, B Menzaghi^2^, A Calcagno^3^, B Celesia^4^, M Ceccarelli^4^, G Nunnari^5^, G De Socio^6^, A Di Biagio^7^, N Leoni^8^, G Angioni^8^, S Babudieri^1^, G Madeddu^2^

^1^Department of Medicine, Surgery and Pharmacy, University Hospital of Sassari, Sassari, Italy; ^2^Unit of Infectious Disease, Busto Arsizio Hospital, Busto Arsizio, Italy; ^3^Department of Medical Science, University of Turin, Turin, Italy; ^4^Department of Clinical and Experimental Medicine, University of Catania, Catania, Italy; ^5^Department of Clinical and Experimental Medicine, University of Messina, Messina, Italy; ^6^Department of Medicine and Surgery, University of Perugia, Perugia, Italy; ^7^Infectious Disease Clinic, IRCCS Policlinico San Martino Hospital, Genoa, Italy; ^8^Unit of Infectious Disease, SS Trinità Hospital, Cagliari, Italy

**Background**: Poor knowledge of sexually transmitted infections (STIs) and HIV among people living with HIV (PWH) could result in a worsening in quality of life [1]. We aimed to investigate the knowledge of STIs prevention, transmission and awareness regarding living with HIV and the U=U campaign, among PWH compared to people without HIV (PWoH).

**Materials and methods**: We proposed a questionnaire regarding STIs and HIV to PWH attending eight outpatient clinics for infectious diseases in Italy. The same questionnaire was administered to PWoH. We collected demographical data, and matched participants 1:2 by age, gender, and level of education. We assigned 1 point to correct, 0.5 point to partially correct, and 0 point to wrong answers. Additional questions regarding disclosure and U=U campaign were proposed to PWH. Differences among two groups were evaluated using Student's t test or Pearson chi‐squared test, as appropriate. The statistical significance level was established as p < 0.05.

**Results**: We collected 132 answers from PWH, matched to 264 PWoH. The mean age was 50.09±11.11 years. No differences were present in gender, level of education, or work status, while there was a higher percentage of homosexual and bisexual people among PWH. Overall, PWH scored better than PWoH (p *<* 0.001). Furthermore, PWH answered significantly better about the route of transmission of HIV (p *=* 0.018) and STIs (p = 0.003), the risk of transmission of HIV living with a PWH (p < 0.001), and the U=U campaign (p < 0.001), while PWoH scored significantly better regarding contraceptive pill as an effective barrier for transmission (p = 0.016) (Figure 1). Regarding HIV‐status disclosure, 88/132 (66.7%) declared it to the general practitioner; reasons for non‐disclosure were no need, fear of judgement, and lack of trust. Moreover, 109/132 (82.6%) disclosed their status to family, friends, or partners. In this case, fear to be avoided, judged, and no need were reasons for non‐disclosure. Of note, 58/132 (43.9%) declared U=U has changed their self‐perceptions, bringing back freedom, self‐confidence, and 'normality'.

**Conclusions**: PWH showed a better knowledge about STIs than PWoH. However, several gaps in the knowledge of these topics and U=U campaign still need to be addressed among both populations. Finally, more effort is needed to reduce stigma and self‐stigma.

**Figure:**
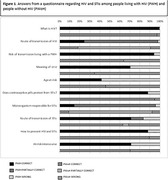
**Abstract P195 – Figure 1**. PWH, people living with HIV; PWoH, people without HIV.

**Reference**

1. Okoli C, Van de Velde N, Richman B, Allan B, Castellanos E, Young B, et al. Undetectable equals untransmittable (U = U): awareness and associations with health outcomes among people living with HIV in 25 countries. Sex Transm Infect. 2021;97:18‐26.

#### P196 Increased serum APOC3 in syphilitic patients can predict the absence of Jarisch‐Herxheimer reaction after benzathine penicillin treatment

H Tsai

Infectious Diseases, Kaohsiung Veterans General Hospital, Kaohsiung, Taiwan

**Background**: Apolipoprotein C3 (APOC3) activates the NLRP3 inflammasome in human monocytes by inducing an alternative NLRP3 inflammasome via caspase‐8 and dimerization of Toll‐like receptors 2 and 4. Little was known about the role of APOC3 in syphilitic patients with Jarisch–Herxheimer reaction (JHR).

**Methods**: This prospective cohort study enrolled adult syphilitic patients with/without JHR between July 2018 and February 2022. Serum samples before and after the administration of the first dose of benzathine penicillin were obtained. The serum level of APOC3 was determined by ELISA. The Kruskal‐Wallis test was used to compare the levels of APOC3 in different groups, and the Wilcoxon signed‐rank test was used to compare changes in APOC3 before and after benzathine penicillin treatment.

**Results**: A total of 40 syphilitic patients and three control groups were enrolled. All 40 syphilitic patients were MSM, and 30 patients (75%) were HIV‐infected individuals. Forty‐seven percent (19/40) of the patients developed JHR. There were statistically significantly higher serum levels of APOC3 (median 38.3 ug/mL, IQR 34.5 to 48.0 ug/mL) in the active syphilis groups (p = 0.02). Among the 21 patients without JH reaction, their serum levels of APOC3 were higher before or after benzathine penicillin treatment compared to the controls (38.9 ug/mL [IQR 34.5 to 66.7 ug/mL] and 39.4 ug/mL [IQR 33.7 to 62.9] ug/mL vs 31.8 ug/mL [IQR 27.5 to 42.2 ug/mL]). The ROC showed the best cut off APOC3 for the absence of JHR before benzathine penicillin therapy compared to the controls were 34.2 (μg/mL) (AUC 0.695, p = 0.017, CI 0.544 to 0.846, sensitivity 0.81, specificity 0.406).

**Conclusions**: A high baseline APOC3 level can predict the absence of the JHR in syphilitic patients treated with benzathine penicillin.

#### P197 A curious tale: quetiapine toxicity with cobicistat but not with ritonavir in a person living with HIV

A Duncan ^1^, T Syme^2^, K Mackie^1^, L Grannell^1^, S Lewin^3^

^1^Pharmacy Department, The Alfred Hospital, Melbourne, Australia; ^2^Department of Psychiatry, The Alfred Hospital, Melbourne, Australia; ^3^Department of Infectious Diseases, The Peter Doherty Institute for Infection and Immunity, Melbourne, Australia

**Background**: Co‐administration of quetiapine with a strong cytochrome P450 3A4 (CYP3A4) inhibitor, such as ritonavir or cobicistat, is predicted to increase quetiapine exposure by 5‐8‐fold, increasing the risk of toxicity. In Europe, quetiapine combination with strong CYP3A4 inhibitors is contraindicated; in the United States, a 1/6 dose reduction is recommended.

**Method**: To describe the effects of a drug interaction and subsequent toxicity caused by switching from ritonavir to cobicistat in a person living with HIV (PLWHIV) previously stable on a high dose of quetiapine.

**Results**: A change in antiretroviral therapy was made for a PLWHIV whose co‐morbidities included Barrett's oesophagus, bipolar disorder, renal impairment and a rash secondary to emtricitabine. As the new regimen included darunavir/ritonavir, the established quetiapine dose was reduced from 700 mg/day to 400 mg/day, to minimise potential toxicity. The other antiretrovirals were lamivudine, tenofovir alafenamide and dolutegravir. Over the following 2 years, the quetiapine dose gradually increased to 1000 mg at night, with no adverse effects. No other medication changes occurred. Darunavir/ritonavir was then simplified to darunavir/cobicistat. After several weeks, the patient complained of severe sedation; his psychiatrist reduced the quetiapine dose to 700 mg, and symptoms improved. However, a month later, due to swallowing difficulties, the patient switched back to ritonavir/darunavir. He was advised to only cautiously increase the quetiapine, if required. After 2 months, the quetiapine dose returned to 1000 mg at night and he has remained stable. This case report is consistent with a more significant interaction between cobicistat and quetiapine than ritonavir and quetiapine. Because ritonavir is a mixed inducer/inhibitor of CYP3A4, effects on other drugs can be variable. In contrast, cobicistat only inhibits CYP3A4, and consequently may have caused greater quetiapine toxicity.

**Conclusion**: Individual assessment of drug dosing to manage drug interactions is an important principle of patient management. In this case, the dose of quetiapine was progressively increased to 1000 mg daily with no apparent adverse effects, despite co‐administration with ritonavir. The changes post initiation of cobicistat were unexpected and may differ in other individuals. In addition to regular psychiatric assessment, measuring quetiapine levels may have a potential future role in managing this challenging interaction.

#### P198 Efficacy of 2DR HIV therapy in polypathological patients with polypharmacy

L Pagnucco, R Bruno, R Gulminetti

Infectious Diseases Unit Department of Medical Science and Infectious Diseases, Fondazione Istituti di Ricovero e Cura a Carattere Scientifico (IRCCS) Policlinico San Matteo, Pavia, Italy

**Background**: Modern combination antiretroviral therapy has prolonged the life expectancy for patients with HIV infection. Furthermore, compared to their peers, PLWHIV are more likely to suffer from comorbidities that often require the use of numerous medicines with a consequent increased risk of therapeutic failure or poor tolerability [1,2]. Guidelines for the Use of Antiretroviral Agents recommend the use of two‐drug regimens (2DR) both in naïve patients [3,4] and, in switch, in experienced patients [3‐6]. It is therefore relevant, in the 2DR, to evaluate the efficacy, safety and durability of these therapeutic regimens in polypathological patients with polypharmacy, haemodialysis treatment and patients receiving immunosuppressive therapy.

**Materials and methods**: We conducted a retrospective cohort study, from December 2015 to February 2022, in patients on 2DR with dolutegravir plus lamivudine (DTG+3TC) or dolutegravir plus rilpivirine (DTG+RPV) and multiple comorbidities. The primary endpoint was to assess the efficacy of a 2DR in these patients. Variables collected were sex, age, VL, CD4, comorbidities, concomitant medication. Follow‐up accrued from 2DR initiation to date of treatment discontinuation or last available visit.

**Results**: Seventy‐two patients, without documented resistance for studied drugs, HBsAg negative, were enrolled; 63% in DTG/3TC and 37% in DTG+RPV. The presence of polypharmacy was defined as the use of four or more medications other than cART. They took overall 199 non‐ARV drugs for 67 different comorbidities of cardiovascular system, bone, kidney, liver, CNS, lung or lipids and glucose homeostasis comorbidities. Three patients were organ transplant recipients (one liver, two kidney) and one bone marrow transplant recipient, five patients on dialysis and, among the 33 patients with a neoplastic disease, 29 patients underwent chemotherapy or immunotherapy. Patients had median follow‐up of 42.3 months after treatment switch. During the follow‐up no virological failure occurred. Three patients presented a viral blip during follow‐up in the DTG+3TC group (>200 copies/mL) and two in the DTG+RPV group (>200 copies/mL) that returned negative at the following control without modifying the antiretroviral treatment. Five subjects discontinued the treatment because of death (one neoplastic diseases and two cirrhosis in the DTG/3TC group and one neoplastic disease in DTG/RPV) and one patient was lost to follow‐up (DTG/3TC). CD4 mean increment was of 113 cells/mcl without a significant change in CD4/CD8 ratio (values relating to immunosuppressed/neoplastic patients were not considered). A slight improvement of the lipid profile was observed.

**Conclusions**: 2DR appears to be a safe, effective, well‐tolerated alternative cART in polypathological patients and in haemodialysis or with severe immunosuppression. Virological suppression was kept in all patients, although five temporary viral blips emerged that returned negative at the following control.

**References**

1. Cantudo‐Cuenca MR, Jiménez‐Galán R, Almeida‐Gonzalez CV, Morillo‐Verdugo R. Concurrent use of comedications reduces adherence to antiretroviral therapy among HIV‐infected patients. J Manag Care Spec Pharm. 2014;20:844‐50.

2. Greene M, Steinman MA, McNicholl IR, Valcour V. Polypharmacy, drug‐drug interactions, and potentially inappropriate medications in older adults with human immunodeficiency virus infection. J Am Geriatr Soc. 2014;62:447‐53.

3. European AIDS Clinical Society. EACS guidelines version 11.0 [Internet]. October 2021 [cited 2022 Sep 23]. Available from: https://www.eacsociety.org/media/final2021eacsguidelinesv11.0_oct2021.pdf.

4. Cahn P, Sierra Madero J, Arribas JR, Antinori A, Ortiz R, Clarke AE, et al. Three‐year durable efficacy of dolutegravir plus lamivudine in antiretroviral therapy ‐ naive adults with HIV‐1 infection. AIDS. 2022;36:39‐48.

5. Aboud M, Orkin C, Podzamczer D, Bogner JR, Baker D, Khuong‐Josses MA, et al. Efficacy and safety of dolutegravir‐rilpivirine for maintenance of virological suppression in adults with HIV‐1: 100‐week data from the randomised, open‐label, phase 3 SWORD‐1 and SWORD‐2 studies. Lancet HIV. 2019;6:e576‐87.

6. Llibre JM, Brites C, Cheng CY, Osiyemi O, Galera C, Hocqueloux L, et al. Efficacy and safety of switching to the 2‐drug regimen dolutegravir/lamivudine versus continuing a 3‐ or 4‐drug regimen for maintaining virologic suppression in adults living with HIV‐1: week 48 results from the phase 3, non‐inferiority SALSA randomized trial. Clin Infect Dis. 2022 Mar 2:ciac130. doi: 10.1093/cid/ciac130.

#### P199 Could we consider U=U as a potential driver of new syphilis infection in HIV population during the Covid‐19 pandemic?

L Todaro ^1^, B Bellocchi^1^, M Paternò Raddusa^1^, S Pulvirenti^1^, A Guarneri^1^, V Boscia^2^, G Nunnari^3^, B Cacopardo^4^, M Ceccarelli^4^, B Celesia^2^

^1^Unit of Infectious Diseases, Azienda Ospedaliera di Rilievo Nazionale e di Alta Specializzazione Garibaldi, Nesima Hospital, Catania, Italy; ^2^Unit of Infectious Diseases, Azienda Ospedaliera di Rilievo Nazionale e di Alta Specializzazione Garibaldi, Catania, Italy; ^3^Department of Clinical and Experimental Medicine, University of Messina, Messina, Italy; ^4^Department of Clinical and Experimental Medicine, University of Catania, Catania, Italy

**Background**: Partner studies support risk‐zero of HIV transmission through condomless sex in serodiscordant couples with the HIV‐positive partner virally suppressed by taking ART. A wide and acritical interpretation of this message could paradoxically reduce the perception of the risk of transmission of other STIs.

**Figure:**
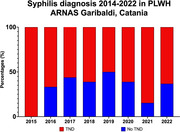
**Abstract P199 – Figure 1**. Syphilis diagnosis 2014 to 2022 in TND (target‐non‐detected) and no‐TND PLWH [TND: VL <20 copies/mL].

**Materials and methods**: This is a retrospective observational study. We analysed the trend of syphilis infection in general population (PLWoH) and among people living with HIV (PLWH) in the period between 1 January 2014 and 30 June 2022. All data have been collected in a worksheet before analysis.

**Results**: Two hundred and fifty‐six patients were enrolled for a total of 315 cases of syphilis infection (256 first diagnosis, 59 (18.7%) re‐infections). One hundred and forty‐six (46.4%) were HIV‐positive, 113 (35.9%) were on antiretroviral treatment, 33 (10.5%) received both the diagnosis of HIV and syphilis. Median age was 37.4 years (IQR 29.9 to 47.7), with a significant difference between PLWoH [33 (IQR 27.9 to 43.6)] and PLWH [41 (IQR 34.3 to 51.7)] (p < 0.001). Among HIV‐positive, median age was 41 years (IQR 36 to 51.8) in treatment‐experienced versus 36 (IQR 32.1 to 51.1) in naïve (p = 0.25). We observed some peaks of diagnosis during 2018 (46), 2020 (42) and 2022 (51 only in the first semester). The distribution of diagnosis of syphilis by years is described in Table 1. Re‐infections occurred in 59 patients (18.7%), 10 (16.9%) PLWoH and 49 (83.1%) in PLWH (p < 0.001). Among PLWH 46 (93.9%) were treatment‐experienced and three were naïve (p < 0.001). Finally, 71 (62.8%) HIV‐positive patients on treatment were virally suppressed (VL <20 copies/mL) before the diagnosis of syphilis. However, six of them had a viral load >200 copies/mL. Data regarding the distribution of viraemia during pre‐ and pandemic period are reported in Figure 1.

**Conclusions**: A significant increase in syphilis diagnosis was observed during the last 6 months. The increased awareness of the meaning of U=U might have reduced the perception of the risk of transmitting and acquiring sexually transmitted infections. More data are necessary to evaluate the relationship between an undiagnosed STI and HIV‐RNA replication.

**Table:** 

**Abstract P199 – Table 1**. Distribution of diagnosis of syphilis by years among people living with HIV (PLWH) and people living without HIV (PLWoH).			
Year of diagnosis	PLWoH (%)	PLWH (%)	Total
2014	20 (95.2)	1 (4.8)	21
2015	14 (77.8)	4 (22.2)	18
2016	15 (48.4)	16 (51.6)	31
2017	22 (59.5)	15 (40.5)	37
2018	24 (52.2)	22 (47.8)	46
2019	17 (54.8)	14 (45.2)	31
2020	24 (57.1)	18 (42.9)	42
2021	24 (63.2)	14 (36.8)	38
2022^a^	9 (17.6)	42 (82.4)	51

^a^Period between 1 January 2022 and 30 June 2022.

### Viral Hepatitis

#### P200 The path to hepatitis C elimination: analysis of the epidemic in 110 countries

K Heath^1^, A Hill ^2^

^1^Public Health, Burnet Institute, Melbourne, Australia; ^2^Department of Pharmacology and Therapeutics, University of Liverpool, Liverpool, UK

**Background**: Hepatitis C (HCV) cures must exceed new HCV infections for eradication to be achieved. Direct acting antivirals (DAAs) such as sofosbuvir‐velpatasvir (SOF/VEL) and sofosbuvir‐daclatasvir (SOF/DAC) can cure over 90% of people treated. Cost remains a barrier to HCV treatment uptake.

**Materials and methods**: Epidemiological data on the HCV epidemic by country were extracted from the Polaris Observatory (https://cdafound.org/polaris/). One hundred and ten countries had data available on HCV prevalence and treatment for 2020. The number of new annual infections – 1.5 million – was extracted from the World Health Organization (WHO) [1] and non‐HCV related deaths were estimated at 1.5% of the total viraemic population. The annual net change was calculated by subtracting the number treated, HCV‐related and non‐HCV related deaths from the number of new infections. The per‐person cost of treatment was estimated by analysing exports of Active Pharmaceutical Ingredients of DAAs using previously described methods [2]. Annual pharmaceutical sales for drugs to treat HCV were compiled from pharmaceutical quarterly sales reports [3].

**Results**: The total HCV epidemic size in 2020 for the 110 countries analysed was estimated to be 51.8 million (90% of the estimated global total of 58 million [1]), with 674 000 (1.3%) on treatment. We estimated 239 000 HCV‐related and 777 000 non‐HCV‐related deaths. The net annual change in epidemic size was estimated to be ‐190 000 (0.4% of the global epidemic size). The estimated cost of cure with generic drugs was $79 for SOF/VEL and $35 for SOF/DAC. In 2019, the global sales for HCV pharmaceuticals to treat 1.3% of people with HCV totalled $3.1 billion. If all 51.8 million HCV cases were treated at $35 per cure, the total cost would be $1.8 billion, or 58% of current global HCV pharmaceutical sales (Figure 1).

**Figure:**
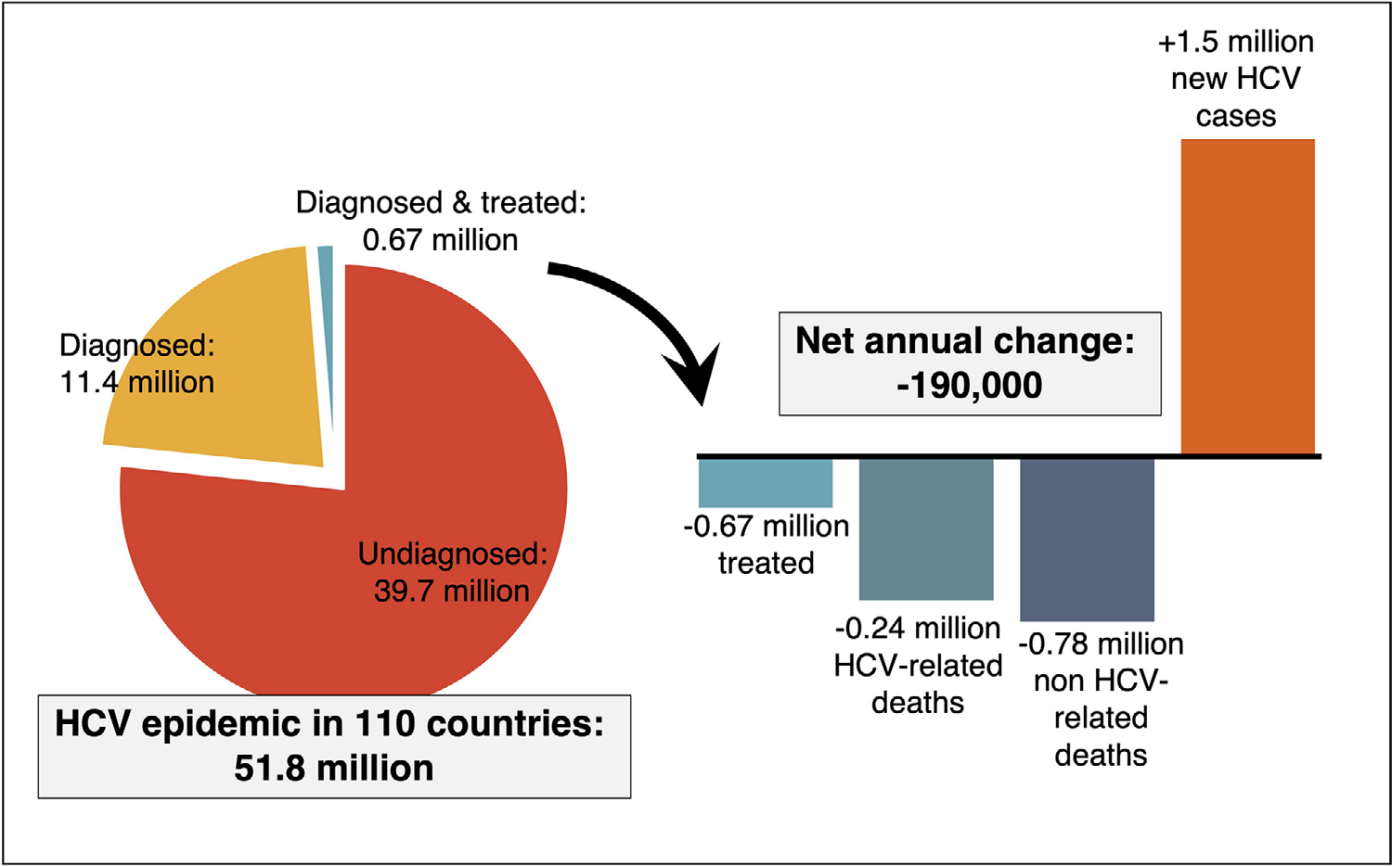
**Abstract P200 – Figure 1**. Visual representation of the global HCV epidemic. The number of new cases is offset by HCV‐related deaths, non HCV‐related deaths and people treated to estimate the net annual change in epidemic size.

**Conclusions**: Despite approximately $92 billion spent on DAAs since launch, and $3.1 billion in 2019, there is no potential for elimination at current rates of treatment. The epidemic size decreased by only 0.4% in 2020, from 51.8 million to 51.6 million. At $35 per cure, we could spend 42% less annually and treat 77 times more people.

**References**

1. World Health Organization. Hepatitis C: Fact Sheet [Internet]. 2022 [cited 2022 Jun 23]. Available from: https://www.who.int/news‐room/fact‐sheets/detail/hepatitis‐c.

2. Hill A, Wang J, Levi J, Heath K, Fortunak J. Minimum costs to manufacture new treatments for COVID‐19. J Virus Erad. 2020;6:61‐9.

3. Barber MJ, Gotham D, Khwairakpam G, Hill A. Price of a hepatitis C cure: cost of production and current prices for direct‐acting antivirals in 50 countries. J Virus Erad. 2020;6:100001.

#### P201 Coinfection with hepatitis B virus and/or hepatitis C virus is a risk factor for HIV virological rebound after achieving virological suppression on first ART: results from ICONA cohort

V Malagnino ^1^, A Cozzi‐Lepri^2^, V Svicher^3^, E Girardi^4^, C Perno^5^, A Saracino^6^, G Cuomo^7^, S Rusconi^8^, M Puoti^9^, A D'Arminio Monforte^10^, M Andreoni^1^, L Sarmati^1^

^1^Medicine of Systems, University of Tor Vergata, Rome, Italy; ^2^Medical School, UCL, London, UK; ^3^Experimental Medicine, Tor Vergata University, Rome, Italy; ^4^Epidemiology and Pre‐Clinical Research, National Institute for Infectious Diseases L. Spallanzani, Rome, Italy; ^5^Microbiologia e Diagnostica di Immunologia, Istituto di Ricovero e Cura a Carattere Scientifico Bambino Gesu Children's Hospital, Rome, Italy; ^6^Infectious Diseases, Università degli Studi, Bari, Italy; ^7^Clinic of Infectious Diseases, Azienda Ospedaliero‐Universitaria Di Modena, Modena, Italy; ^8^Infectious Diseases Unit, Legnano General Hospital, Azienda Socio Sanitaria Territoriale Ovest Milanese, Milan, Italy; ^9^Department of Infectious Diseases, Grande Ospedale Metropolitano Niguarda, Milan, Italy; ^10^Department of Health Sciences, Azienda Socio Sanitaria Territoriale Santi Paolo e Carlo, Milan, Italy

**Background**: The aim of this study was to investigate the impact of viral hepatitis coinfection on the risk of HIV viral rebound (VR) after achieving suppression with a first‐line antiretroviral therapy (ART) in real‐world data.

**Materials and methods**: Patients living with HIV (PLWHIV), enrolled in the ICONA Foundation Study cohort, achieving viral suppression ≤50 copies/mL on their first‐line ART, were prospectively evaluated and divided in five exposure groups according to serology test results at ART initiation: (A) HIV mono‐infected; (B) HCV+/HBsAg‐/HBcAb‐; (C) HBcAb+/HCV‐/HBsAg‐; (D) HCV+/HBcAb+/HBsAg‐; (E) HBsAg+/HBcAb+/HCV‐. We investigated the occurrence of VR, defined as the time of two consecutive HIV‐RNA values >50 copies/mL after achieving viral suppression. Standard survival analysis by means of KM curves and Cox regression analysis with time‐fixed covariates measured at baseline was employed after controlling for mode of HIV transmission and nation of birth.

**Results**: Of a total of 6380 patients included and followed for a median of 47 months (IQR 23 to 80), 4108 (64%) resulted HIV mono‐infected, 290 (5%) HCV+, 1362 (21%) HBcAb+, 390 (6%) HCV+/HBcAb+ and 230 (4%) HBsAg+ coinfected. Overall, 829 (13%) PLWHIV experienced VR over follow‐up (Figure 1). After controlling for key confounders, coinfected PLWHIV showed an increased risk of experiencing VR compared to the HIV mono‐infected. In particular, the strongest associations were seen for the HCV+/HBcAb+ (aHR 2.0, p < 0.0001) and HCV+ (aHR 1.72, p = 0.0001) followed by HBsAg+ (aHR 1.47, p = 0.026), and HBcAb+ group (aHR 1.23, p = 0.019), the latter showing also a smaller magnitude of the effect (Table 1).

**Conclusions**: Coinfection with all major hepatotropic viruses had an impact on the probability of maintaining HIV viral suppression after beginning of ART. Of note, even PLWHIV with HBcAb, a marker of occult HBV infection, appeared to be at considerably higher risk of VR than HIV mono‐infected and should be carefully monitored.

**Figure:**
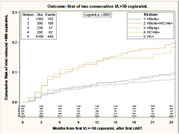
**Abstract P201 – Figure 1**. Cumulative risk of viral rebound >50 copies/mL by baseline HBV and HCV serology group.

**Table:** 

**Abstract P201 – Table 1**. Unadjusted and adjusted relative hazards of virological rebound >50 copies/mL.				
	Unadjusted HR (95% CI)	p‐value	Adjusted HR^a^ (95% CI)	p‐value
HIV+ (group A)	1		1	
HCV+/HBsAg‐/HBcAb‐ (group B)	2.10 (1.61 to 2.74)	<0.001	1.72 (1.27 to 2.33)	<0.001
HCV‐/HBsAg‐/HBcAb+ (group C)	1.24 (1.04 to 1.47)	0.015	1.23 (1.03 to 1.47)	0.019
HCV+/HBsAg‐/HBcAb+ (group D)	2.60 (2.11 to 3.21)	<0.001	2.00 (1.51 to 2.65)	<0.001
HCV‐/HBsAg+/HBcAb+ (group E)	1.49 (1.07 to 2.09)	0.019	1.47 (1.05 to 2.05)	0.026

^a^Adjusted for nationality, and mode of transmission.

#### P202 Persistently high rates of HCV viraemia among high‐risk people living with HIV in Taiwan in the era of direct‐acting antivirals

H Sun ^1^, L Su^1^, Y Chen^1^, S Huang^2^, Y Huang^3^, S Ho^4^, W Liu^1^, Y Su^1^, S Chang^4^, C Hung^1^

^1^Department of Internal Medicine, National Taiwan University Hospital, Taipei, Taiwan; ^2^Department of Internal Medicine, National Taiwan University Hospital, Hsin‐Chu Branch, Hsin‐Chu, Taiwan; ^3^Department of Internal Medicine, National Taiwan University Hospital, Biomedical Park Branch, Hsin‐Chu, Taiwan; ^4^Department of Laboratory Medicine, National Taiwan University Hospital, Taipei, Taiwan

**Background**: The treatment‐as‐prevention strategy with direct‐acting antivirals (DAAs) has substantially lowered the rates of HCV viraemia among people living with HIV (PLWHIV) in the several EU countries, UK and Australia. However, the impact of improved access to DAA treatments on HCV viraemia among high‐risk PLWHIV in the Asia‐Pacific region remains unknown.

**Materials and methods**: High‐risk PLWHIV, defined as those with sexually transmitted infections (STIs), elevated aminotransferases within the past 6 months, or spontaneous HCV clearance or achievement of sustained virological response by antivirals (past HCV infection), were enrolled in Taiwan prospectively from 2019/6/25 to 2022/6/24 (period 1: 2019/6/25 to 2020/6/24; period 2: 2020/6/25 to 2022/6/24) and underwent three‐stage pooled‐plasma HCV RNA testing every 12 weeks until detection of HCV RNA or completion of 48‐week follow‐up. PLWHIV with HCV viraemia before enrolment were excluded. The three‐stage strategy combined 20 individual specimens into a stage‐1 pool, five individual specimens from the stage‐1 pool tested positive for HCV RNA into stage‐2 mini‐pool, followed by testing of individual specimens of stage‐2 mini‐pool tested positive for HCV RNA. Those with HCV viraemia were linked to reimbursed DAA treatments.

**Results**: A total of 1155 participants (609 in period 1 and 546 in period 2) with a mean age of 38.1 years were enrolled. At enrolment, 1131 (97.9%) were men who have sex with men (MSM), 78.2% had STIs, and 30.5% past HCV infection. Clinical characteristics of PLWHIV enrolled in two periods were similar. After a total of 825.96 person‐years of follow‐up (PYFU) (523.79 in period 1 and 302.17 PYFU in period 2), 79 cases of HCV viraemia (6.8%) were detected, 6.7% [41/609] in period 1 and 7.0% [38/546] period 2, (p = 0.907). Forty‐one cases (51.9%) were detected at enrolment (day 1), and 29 cases (36.7%) were classified as HCV re‐infections (46.3% and 26.3% in period 1 and 2, respectively; p = 0.101). The incidence rate of HCV viraemia was 46.01 per 1000 PYFU without significant difference between the two periods (47.73 vs 43.02 per 1000 PYFU, p = 0.775).

**Conclusions**: Despite the improvement in access to DAAs in Taiwan, the incidence of HCV viraemia among high‐risk PLWHIV remained high with the frequent HCV RNA testing.

#### P203 Liver fibrosis and steatosis in hepatitis B‐positive individuals with and without HIV coinfection in Senegal

A Ramírez Mena ^1^, K Ndiaye^2^, J Tine^3^, A Gaye^2^, L Fortes^4^, O Ndiaye^5^, D Ka^3^, M Fall^3^, N Ngom^2^, M Seydi^3^, G Wandeler^1^

^1^Infectious Diseases Department, Bern University Hospital, Bern, Switzerland; ^2^Centre de Traitement Ambulatoire, Fann University Hospital, Dakar, Senegal; ^3^Service de Maladies Infectieuses et Tropicales, Fann University Hospital, Dakar, Senegal; ^4^Service de Maladies Infectieuses, Centre Hospitalier National Dalal Jamm, Guediawaye, Senegal; ^5^Centre de Recherche et Formation Clinique, Fann University Hospital, Dakar, Senegal

**Background**: Hepatitis B virus (HBV) infection is the first cause of liver cirrhosis and cancer in West Africa. Although >10% of the general population is affected by chronic HBV infection, only a few large‐scale, dedicated research efforts have been undertaken to date. Here, we present data on liver fibrosis and steatosis, two important drivers of liver disease, at study enrolment.

**Material and methods**: We included all HIV/HBV as well as HBV‐monoinfected individuals presenting after October 2019 at two HIV clinics in Dakar, Senegal. We measured HBV_DNA using the COBAS/TaqMan^®^ platform, liver fibrosis (liver stiffness [LS] ≥7.1 kPa), cirrhosis (LS ≥11.1 kPa), and steatosis (controlled attenuation parameter [CAP] ≥248 dB/m) using transient elastography (TE). TE results were compared between persons with and without HIV using descriptive statistics, and the association between HIV infection and liver fibrosis and steatosis was assessed using multivariable with logistic regression adjusted for age, sex, HBV DNA, and body mass index (BMI).

**Results**: Between October 2019 and November 2021, we included 581 individuals, of whom 110 were HIV/HBV‐coinfected and on antiretroviral therapy (ART). The median age was higher in HIV/HBV‐coinfected compared to HBV‐monoinfected participants (45 vs 33 years, p < 0.001) but the proportion of women was similar in both groups (53% vs 46%, p = 0.18). Among HIV/HBV‐coinfected, 89/107 (83%) had a suppressed HBV DNA, and 102/107 (95%) had a value below 2000 IU/mL. The prevalence of significant fibrosis was 13% in HBV‐monoinfected and 6% in HIV/HBV‐coinfected (p = 0.07) individuals, whereas the proportion of cirrhosis was 4% in both groups. Liver steatosis was found in 16/110 (15%) HIV/HBV‐coinfected and 40/471 (8%) HBV‐monoinfected participants (p = 0.05). In multivariable analyses, male sex (adjusted odds ratio 3.83, 95% confidence interval 1.72 to 8.53) and HBV DNA >2000 IU/mL (2.86, 1.41 to 5.83) were associated with liver fibrosis, whereas age >40 years (4.88, 1.86 to 12.80) and BMI >25 (4.52, 1.88 to 10.84) were associated with liver steatosis.

**Conclusion**: SEN‐B is one of the first prospective cohorts of HBV‐infected individuals established in sub‐Saharan Africa. The overall prevalence of liver fibrosis and steatosis was low, and no association with HIV infection status was found. The strong association between high HBV viral load and liver fibrosis could be an argument for early antiviral therapy.

#### P204 Long‐term trends in qHBsAg levels in persons with and without functional HBV cure in the Swiss HIV Cohort Study

L Begré ^1^, A Boyd^2^, F Suter‐Riniker^3^, C Béguelin^1^, J Rockstroh^4^, H Günthard^5^, A Calmy^6^, M Cavassini^7^, M Stöckle^8^, P Schmid^9^, E Bernasconi^10^, M Levrero^11^, F Zoulim^11^, G Wandeler^1^, A Rauch^1^

^1^Department of Infectious Diseases, Inselspital, Bern University Hospital, University of Bern, Bern, Switzerland; ^2^Department of Infectious Diseases, Research & Prevention, Public Health Service of Amsterdam, Amsterdam, Netherlands; ^3^Institute for Infectious Diseases, University of Bern, Bern, Switzerland; ^4^HIV Clinic, Department of Medicine, University Hospital Bonn, Bonn, Germany; ^5^Department of Infectious Diseases, University Hospital Zurich and Institute of Medical Virology, University of Zurich, Zurich, Switzerland; ^6^Division of Infectious Diseases, University Hospital Geneva, University of Geneva, Geneva, Switzerland; ^7^Division of Infectious Diseases, University Hospital Lausanne, University of Lausanne, Lausanne, Switzerland; ^8^Division of Infectious Diseases & Hospital Epidemiology, University Hospital Basel, University of Basel, Basel, Switzerland; ^9^Division of Infectious Diseases & Hospital Epidemiology, Cantonal Hospital St. Gallen, St. Gallen, Switzerland; ^10^Division of Infectious Diseases, Regional Hospital Lugano, University of Geneva and University of Southern Switzerland, Lugano, Switzerland; ^11^Hepatology Department, INSERM U1052, Hospices Civils de Lyon, Cancer Research Center of Lyon, University of Lyon, Lyon, France

**Background**: Functional cure of hepatitis B virus (HBV) infection occurs infrequently in persons living with HIV and hepatitis B and could be predicted by individual levels of quantitative hepatitis B surface antigen (qHBsAg). We aimed to compare long‐term trends in qHBsAg levels among persons living with HIV/HBV who did or did not exhibit functional HBV cure during treatment with tenofovir in the Swiss HIV Cohort Study.

**Materials and methods**: We included 29 participants who experienced functional HBV cure on tenofovir‐containing antiretroviral therapy. These individuals were then matched 1:1 on age, sex, pre‐treatment with lamivudine and CD4 cell count category to those who did not experience functional HBV cure. We defined functional HBV cure as the first moment when qHBsAg level was <0.05 IU/mL. We assessed time to functional HBV cure and modelled qHBsAg levels over time using linear regression with fractional polynomials.

**Results**: The 58 participants had a median age of 41 years (interquartile range (IQR) 37 to 46), 21% were female, 83% were pre‐treated with lamivudine before starting tenofovir, and 14% had a CD4 cell count <200 cells/μL. In those with functional HBV cure, median time to qHBsAg <0.05 IU/mL was 48 months (range 6 to 168) with cure occurring in 8/29 (28%) participants within 1 and in another five participants within 2 years (17%) after starting tenofovir. Median qHBsAg at start of tenofovir was 9731 IU/mL (IQR 2994 to 16 368) in participants without functional HBV cure and 2293 (IQR 113 to 28 574) in participants with functional HBV cure (p = 0.15). Individual and modelled qHBsAg levels are shown in Figure 1. After 2 years, 24/29 (83%) participants with functional HBV cure and 3/29 (10%) participants without functional HBV cure had a qHBsAg decline of at least 1 log10 IU/mL or qHBsAg <1 log10 IU/mL at start of tenofovir (p < 0.001).

**Conclusions**: Most persons living with HIV/HBV exhibiting functional HBV cure experienced a more than 1‐log10 decline in qHBsAg levels within the first 2 years on tenofovir. However, some persons with functional HBV cure did not have an immediate qHBsAg decline. In patients without functional HBV cure, qHBsAg levels remained remarkably stable during long‐term follow‐up.

**Figure:**
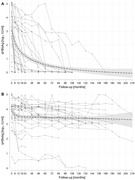
**Abstract P204 – Figure 1**. Trends in quantitative HBsAg levels in participants with (A) and without (B) functional HBV cure during treatment with tenofovir. qHBsAg levels modelled as fractional polynomials of time (dashed line) with 95% confidence intervals (shaded area) and individual trajectories of qHBsAg (circles with connecting solid lines).

#### P205 HCV testing training for non‐HCV specialists in a tertiary hospital: change in attitudes and rates of HCV screening

A García García ^1^, M Vivancos Gallego^1^, J Martínez‐Sanz^1^, C Cano^1^, A González‐Sarria^1^, B Romero^2^, M Sánchez‐Conde^1^, M Vélez Díaz‐Pallarés^2^, F Gea Rodríguez^3^, J Galán Montemayor^4^, M Pérez‐Elías^1^

^1^Infectious Diseases Department, Hospital Ramón y Cajal, Madrid, Spain; ^2^Pharmacy Department, Hospital Ramón y Cajal, Madrid, Spain; ^3^Gastroenterology Department, Hospital Ramón y Cajal, Madrid, Spain; ^4^Microbiology Department, Hospital Ramón y Cajal, Madrid, Spain

**Background**: HCV infection is a curable cause of chronic hepatitis, but many patients remain undiagnosed. National and international HCV testing guidelines are poorly known by non‐HCV specialists. Increasing awareness is essential to improve HCV screening. Few programmes targeting these groups have previously been reported.

**Methods**: In a tertiary hospital, six infectious diseases physicians provided a 1‐hour training session on HCV screening to each of the departments. A brief questionnaire was used before and after the training to evaluate attitudes towards HCV screening. We compared the absolute number of tests requested, the screening rate per 1000 patients attended, and new HCV diagnoses in the 6‐month period before and after training, for each hospital department. Information on test requests and newly confirmed HCV diagnoses were obtained from the Microbiology and Infectious Diseases departments respectively. The number of patients attended was obtained from the hospital management service.

**Results**: From March to November 2019, a total of 345 non‐HCV specialists from 30 hospital departments (17 medical, 13 surgical) were trained and responded to the questionnaire. According to the pre‐training questionnaire, 18% of non‐HCV specialists (30% medical vs 3% surgical, p < 0.001) were aware of HCV testing guidelines (5% routinely ordered HCV testing, 60% if there was an obvious exposure risk or indicator conditions and 34% never did so). In post‐training responses, 98% considered the training useful, and responses showing a positive attitude towards routine HCV testing increased to 20%. The rate of those who claimed to never request tests decreased to 2% (p < 0.001). Out of an estimated number of 783 842 patients attended in both periods, we observed a 12.6% increase in HCV tests requested (p = 0.022), with a significant increase in medical (17.8%, p = 0.019) but not in surgical departments (1.29%, p = 0.5). We also observed a global 15.78% increase in HCV positivity rate (p = 0.11) due to a 16.5% increase in medical departments (p = 0.027) (Table 1).

**Table:** 

**Abstract P205 – Table 1**. Distribution of HCV tests, positive serologies and active HCV infections among the different departments before and after training.									
Department	HCV tests/103 patients	HCV tests/103 patients	p	HCV Ab (+)/ 105 patients	HCV Ab (+)/ 105 patients	p	HCV ag/PCR (+)/105 patients	HCV ag/PCR (+)/105 patients	p
	Before‐Training	After‐Training		Before‐Training	After‐Training		Before‐Training	After‐Training	
All	5.79	6.52	<0.001	19.39	22.45	0.111	5.99	6.76	0.212
Medical	6.54	7.71	<0.001	29.36	34.22	0.027	9.51	10.77	0.187
Surgical	4.66	4.72	0.725	4.19	4.51	0.564	0.64	0.64	1.000

**Conclusions**: Non‐HCV specialists reported poor knowledge of HCV screening guidelines, being worse in surgical departments. Directed training was considered useful and significantly improved predisposition for HCV screening in both medical and surgical departments. An improve in HCV screening was detected after the intervention, although concentrated only in medical departments.

#### P206 Hepatitis A outbreak in men who have sex with men in a tertiary care facility in Romania

C Oprea, A Paun, T Paduraru, I Popa, I Ianache

Infectious Diseases ‐ HIV/AIDS, 'Victor Babes' Hospital for Infectious and Tropical Diseases, Bucharest, Romania

**Background**: Faecal‐oral transmission through sexual activity is a recognised route of hepatitis A (HAV) transmission and there have been outbreaks of hepatitis A among MSM (men who have sex with men) in recent decades. The aim of our study was to assess the clinical features, laboratory testing and outcomes in MSM who acquired HAV through sexual contact and to raise professional and public awareness about an ongoing outbreak of HAV in Bucharest.

**Table:** 

**Abstract P206 – Table 1**. Values of liver enzymes at admission in MSM with HIV and HAV infection.			
Enzyme		Median (IQR)	Normal values
ALAT	IU/L	3255 (1679 to 4898)	6 to 63
ASAT	IU/L	964 (527 to 2687)	0 to 38
Total bilirubin	mg/dL	10.7 (7.0 to 12.4)	0 to 1.1
Direct bilirubin	mg/dL	9.9 (6.3 to 10.5)	0 to 0.3
Prothrombin time	%	68.5 (55.5 to 84.7)	

**Methods**: A prospective study was conducted by collecting epidemiological, clinical and laboratory data from patients who identified as MSM, admitted with HAV at Victor Babes Hospital for Infectious and Tropical Diseases, Bucharest between 15 March and 15 June 2022. HAV was confirmed by positive serology (IgM anti HAV) and negative markers for non‐hepatotropic viral infections.

**Results**: During 3 months, 22 MSM were diagnosed with HAV. Out of these, 17 were PLWHIV under cART and five were HIV negative. The median age at HAV diagnosis was 30 years (IQR 25 to 37) and the majority (18) were from urban areas. Six of them engaged in sexual practices such as groupsex/chemsex. The most frequent clinical onset was with fever and gastro‐intestinal symptoms. Marked elevations of transaminases and bilirubin were observed at admission (Table 1). The median CD4 cell count/μL was 775 (IQR 451 to 886), 12 had undetectable HIV viral load, two low level viraemia and three were recently diagnosed with HIV. Two patients were diagnosed simultaneously with syphilis and 11 had serological markers of a previous infection. The median time of hospitalisation was 12 days (IQR 7 to 11). The outcome was favourable with full clinical recovery in all patients. Due to continuous risk behaviour one patient was diagnosed with monkeypox 2 months after HAV.

**Conclusions**: We report an alarming increase in the numbers of cases with HAV among the MSM in Bucharest. Moreover, an increasing engagement in sexual practices with a higher risk for HIV, HAV and STIs acquisition was observed. In order to reduce the healthcare burden it would be important to strengthen prevention methods, contact tracing and to reinforce the HAV vaccination recommendation in these high‐risk groups.

#### P207 Liver transplantation due to fulminant HBV infection in individuals whose HIV infection was diagnosed during pretransplant evaluation: report on two cases

A Gutierrez Villanueva^1^, A Arias Milla^1^, P Ruiz^2^, A Diaz de Santiago^1^, A Forner^2^, M Laguno^3^, V Cuervas‐Mons^1^, L Benitez^1^, A Moreno^3^, A Moreno Maroto^1^, A Rimola^2^, J Miró ^3^

^1^Internal Medicine, Puerta de Hierro University Hospital Madrid, Instituto De Investigación Sanitaria Puerta de Hierro‐Segovia de Arana, Madrid, Spain; ^2^Hepatology, Hospital Clinic ‐ August Pi i Sunyer Biomedical Research Institute (IDIBAPS), University of Barcelona, Barcelona, Spain; ^3^Infectious Diseases, Hospital Clinic ‐ August Pi i Sunyer Biomedical Research Institute (IDIBAPS), University of Barcelona, Barcelona, Spain

**Background**: Liver transplantation (LT) in patients living with HIV (PLWHIV) is part of routine clinical care. Most cases have end‐stage liver disease or hepatocellular carcinoma, and LT recipients have known HIV infections, are on antiretroviral therapy (ART) and have undetectable plasma HIV RNA viral loads and CD4+ T cell counts above 100 cells/mm^3^ [1,2]. In contrast, there is limited information on LT in patients with acute liver disease whose HIV infection is diagnosed during pretransplant evaluation [3]. This study aims to describe the evolution of two successful LT patients diagnosed with HIV infection in the context of fulminant HBV hepatitis.

**Results**: CASE 1: A 50‐year‐old male was admitted to the ER, due to liver failure, needing LT. He was an untreated hepatitis B surface antigen (HBsAg) carrier since childhood. HIV‐1 infection was diagnosed with no genotypic antiretroviral resistances. CD4 was 384 cells/mm^3^. ART with TDF and FTC plus raltegravir was started. The evolution of plasma HBV and HIV viral load and CD4+ T cell counts is depicted in Table 1. The immunosuppressants used were basiliximab, mycophenolate, a tapering dose of corticoids and tacrolimus. HBV immunoglobulin (HBIG) was used monthly for 2 years. Four years later, plasma HIV and HBV were both undetectable, CD4 was 268 cells/mm^3^ and graft function was normal. CASE 2: A 44‐year‐old male was admitted, due to fulminant HBV liver failure, needing an emergent liver transplant. HIV‐infection was diagnosed with no genotypic antiretroviral resistances. CD4+ T cell count was 82 cells/mm^3^. ART, immunosuppressants and HBV prophylaxis were the same. The evolution of plasma HBV and HIV viral load and CD4+ T cell counts is depicted in Table 1. Seven years later plasma HIV and HBV were both undetectable, CD4 was 549 cells/mm^3^ and graft function was normal.

**Table:** 

**Abstract P207 – Table 1**. Evolution of plasma HBV and HIV viral load and CD4+ T cell counts.						
	CASE 1	CASE 1	CASE 1	CASE 2	CASE 2	CASE 2
	HBV (U/ml)	HIV (copies/ml)	CD4/mm^3^	HBV (U/ml)	HIV (copies/ml)	CD4/mm^3^
Before LT	1,580,000	162,000	384	35,328,501	41820	82
+1m	34.9	ND	129	1370	<50	321
+3m	17.5	<50	200	565	<50	277
+6m	<10	<50	236	426	<50	329
+12m	<10	<50	230	517	<50	454
+24m	<10	<50	339	<10	<50	309
+3y	<10	<50	262	153	<50	364
+4y	<10	<50	268	28	<50	421
+5y				<10	<50	425
+6y				<10	<50	544
+7y				<10	<50	549

**Conclusion**: LT can be safely performed in individuals with fulminant HBV infection whose HIV‐infection is diagnosed during pretransplant evaluation and who therefore do not meet the HIV‐dependent criteria for solid organ transplantation. TDF plus FTC and raltegravir allowed rapid control of HIV and HBV viraemia, and both patients had very good short‐ and long‐term results.

**References**

1. Miró JM, Torre‐Cisnero J, Moreno A, Tuset M, Quereda C, Laguno M, et al. GESIDA/GESITRA‐SEIMC, PNS and ONT consensus document on solid organ transplant (SOT) in HIV‐infected patients in Spain (March, 2005). Enferm Infecc Microbiol Clin. 2005;23:353‐62.

2. EACS guidelines. Version 11.0 [Internet]. October 2021 [cited 2022 Aug 23]. Available from: https://www.eacsociety.org/media/final2021eacsguidelinesv11.0_oct2021.pdf.

3. Werbel WA, Durand CM. Solid organ transplantation in HIV‐infected recipients: history, progress, and frontiers. Curr HIV/AIDS Rep. 2019;16:191‐203.

#### P208 Detectable HBV viraemia in HIV/HBV co‐infected patients undergoing HBV active antiretroviral therapy

G Morsica^1^, R Lolatto^1^, C Bertoni ^2^, A Siribelli^1^, M Bottanelli^2^, H Hasson^1^, A Castagna^2^, C Uberti‐Foppa^2^

^1^Department of Infectious Diseases, San Raffaele, Scientific Institute, Milan, Italy; ^2^Department of Infectious Diseases, Vita Salute University, Milan, Italy

**Background**: HBV resistance to tenofovir disoproxil fumarate (TDF) and tenofovir alafenamide fumarate (TAF) has not been reported, so the reasons for HBV DNA detectability during HBV‐active ART are unclear.

**Methods**: Of 147 patients living with HIV (PLWHIV) and coinfected with HBV followed as outpatients at San Raffaele Hospital, Italy, 14 (9.6%) who had HBV‐DNA detectable at last evaluation available (2021 to 2022) were investigated.

**Results**: Characteristics of patients with HIV/HBV coinfection and HBV‐DNA positivity are summarised in Table 1. Most patients were males, the main route of transmission was the sexual route; cirrhosis was present in 2/14 PLWHIV, both with HBV/HCV coinfection. Antiretroviral treatment (ART) including drugs against HIV/HBV was: TDF + emtricitabine (FTC) in five PLWHIV, (TAF) 10 mg + FTC in seven PLWHIV and TAF 25 mg + FTC in two PLWHIV. No adherence was observed in 4/14 patients. None had resistance to TDF or TAF, while in 2/9 cases was identified a resistant strain to lamivudine and entecavir. Interestingly, in one other case previously harbouring a resistant strain to lamivudine, a reversion to wild‐type was detected, although HBV‐DNA was positive at high levels (35 800 000 IU/mL). This patient was on ART including TAF 10 mg + FTC and treatment was changed to TDF + FTC with a dramatic decrease of HBV viraemia (127 000 IU/mL). Comparison of patients with HBV‐DNA detectable with those with undetectable HBV‐DNA according to the dosage of TAF showed that among HBV‐DNA pos, N=9, the dosage of TAF was: TAF 10 mg, N=7 and TAF 25 mg, N=2, versus HBV‐DNA neg, N=95: TAF 10 mg, N=30, TAF 25 mg, N=65; so, treatment with TAF 10 mg was prevalent in the group of HBV‐DNA pos, p = 0.047.

**Table:** 

**Abstract P208 – Table 1**. Characteristics of HIV/HBV coinfected patients with detectable HBV‐DNA.	
Number of patients (N) = 14	
Age	54 (48.5 to 59)
Sex, male	13 (92.8)
Risk for HIV infection, sexual exposure/IVDU/unknown	5 (36)/2(14)/7(50)
CD4 cells count, number/mmc	357 (266 to 971)
CD8 cells count, number/mmc	1060 (789 to 1313)
CD4/CD8 ratio	0.6 (0.55 to 0.62)
AST, IU/L	54 (25 to 72)
ALT, IU/L	53 (37 to 92)
GGT, IU/L	76 (33 to 111)
HBeAg pos	6 (43)
Anti‐HBeAg pos	4 (28.5)
HBeAg/anti‐HBeAg unknown	4 (28.5)
Anti‐HDV pos	1 (7.1)
Anti‐HCV pos	5 (36)
HBV‐DNA IU/mL	95 (18 to 1690)
HIV‐RNA ≥50 copies/mL	5 (36)
HIV‐RNA copies/mL	114 (73 to 900)

Results described by median (interquartile range [IQR]) or frequency (%).

**Conclusions**: In this small but well characterised group of PLWHIV with HBV coinfection, about 10% of patients had positive HBV viraemia, five with concomitant HIV viraemia. None had resistance to TDF or TAF, while in 2/9 cases was identified a resistance to nucleoside analogues (NA) lamivudine and entecavir. Over than poor compliance, the dosage of TAF and resistance to NA may have contributed to HBV‐DNA detectability.

### Clinical Pharmacology

#### P209 Physiologically based pharmacokinetic modelling of long‐acting rilpivirine in pregnancy

S Atoyebi, S Granana‐Castillo, M Siccardi, C Waitt

Department of Pharmacology and Therapeutics, University of Liverpool, Liverpool, UK

**Background**: Long‐acting rilpivirine has been approved for the management of HIV in non‐pregnant adults, but pharmacokinetics in pregnant women have not been fully characterised. The aim of this study was to develop a physiologically based pharmacokinetic (PBPK) model for the prediction of intramuscularly (IM) administered long‐acting rilpivirine (900 mg rilpivirine followed by 600 mg rilpivirine monthly) in pregnancy.

**Materials and methods**: A whole‐body adult PBPK model was developed in SimBiology (MATLAB, R2019a). The model was qualified using clinical PK data of oral and long‐acting rilpivirine in adults. The model was assumed to be verified if the simulated values were within 2‐fold of the reported clinical values and if the absolute average‐fold error (AAFE) was below 2. Afterwards, the qualified adult PBPK model was modified to incorporate pregnancy‐induced physiological and metabolic changes known to influence drug PK (e.g. organ blood‐flow rates, gestational age‐defined CYP3A4 activity, etc.). Clinical PK data of oral rilpivirine in pregnancy were used to qualify the pregnancy PBPK model. The qualified pregnancy PBPK model was used to simulate the pharmacokinetics of 900 mg IM rilpivirine followed with 600 mg IM rilpivirine monthly initiated for 12 weeks at different stages of pregnancy.

**Results**: The model was successfully qualified with a mean (min‐max) AAFE of 1.18 (1.04‐1.31) for oral rilpivirine and 1.32 (1.03‐1.68) for long‐acting rilpivirine PK parameters in the adult population. AAFE values for oral rilpivirine PK parameters in the second and third trimesters of pregnancy were 1.46 (1.24‐1.80). Predicted PK‐time profiles of 900 mg IM followed by 600 mg IM rilpivirine monthly in non‐pregnant and pregnant adults for 12 weeks are shown in Figure 1.

**Conclusions**: The developed PBPK modelling approach suggests that approved doses of long‐acting rilpivirine may maintain therapeutic concentrations and clinical efficacy during pregnancy similar to that achieved in non‐pregnant adults without dose adjustments. Confirmation of these findings in clinical trials are warranted. This approach could be employed to explore different dosing scenarios in pregnancy and support the design of future clinical trials in pregnant adults.

**Figure:**
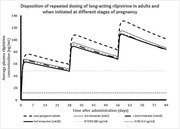
**Abstract P209 – Figure 1**. Predicted plasma pharmacokinetics of repeated dosing of long‐acting rilpivirine (900 mg IM rilpivirine followed by 600 mg IM rilpivirine monthly) initiated at different stages of pregnancy compared with non‐pregnant adults.

#### P210 Effects of the HIV‐1 maturation inhibitor GSK3640254 on QT interval in healthy participants

Y Zhang ^1^, M Johnson^2^, M Bush^2^, P Yazdani^3^, J Zhan^4^, B Wen^5^, V Bainbridge^6^, B Wynne^7^, S Joshi^8^, M Lataillade^9^

^1^Clinical Pharmacology Modeling and Simulation, GSK, Collegeville, PA, USA; ^2^Clinical Pharmacology, ViiV Healthcare, Durham, NC, USA; ^3^Clinical Development, GSK, Collegeville, PA, USA; ^4^Statistics, GSK, Collegeville, PA, USA; ^5^Clinical Pharmacology, GSK, Collegeville, PA, USA; ^6^Clinical Development, GSK, Brentford, UK; ^7^Clinical Development, ViiV Healthcare, Durham, NC, USA; ^8^Clinical Development, ViiV Healthcare, Branford, CT, USA; ^9^Global Research Strategy, ViiV Healthcare, Branford, CT, USA

**Background**: GSK3640254 (GSK’254) is a next‐generation HIV‐1 maturation inhibitor with pharmacokinetics (PK) supporting once‐daily (QD) therapy for HIV‐1 treatment. This thorough QT/corrected QT (QTc) study evaluated the effect of GSK’254 on cardiac repolarization.

**Figure:**
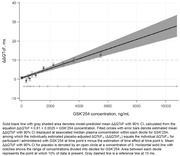
**Abstract P210 – Figure 1**. Model‐predicted ΔΔQTc (mean and 90% CI) and estimated placebo‐corrected ΔQTc (mean and 90% CI) across deciles of GSK’254 plasma concentrations.

**Materials and methods**: In this two‐part, randomized study, healthy participants received GSK’254 or placebo QD for 7 days (part 1) to determine safety and PK of a 500‐mg GSK’254 supratherapeutic dose. Four sequential treatment periods composed the main QTc study (part 2): GSK’254 100 mg, GSK’254 500 mg, placebo QD for 7 days, or placebo QD for 6 days with a 400‐mg moxifloxacin dose on day 7 (all administered with a moderate‐fat meal). Pharmacokinetic parameters were calculated by standard noncompartmental analysis. Concentration–QTc (cQT) analyses modeled the relationship between individually observed GSK’254 plasma concentrations and placebo‐adjusted change from baseline in QT interval corrected with Fridericia's formula (ΔΔQTcF).

**Results**: Of 50 participants enrolled, 3/8 (38%) and 18/42 (43%) reported adverse events in parts 1 and 2, respectively (all maximum grade 1). On day 7 in part 2, geometric mean (95% CI) maximum GSK’254 concentrations were observed 5 hours post‐dose with GSK’254 doses (100 mg: 830 [738 to 934] ng/mL; 500 mg: 4260 [3750 to 4840] ng/mL). Estimated population slope of the cQT model was 0.0025 ms per ng/mL (90% CI 0.00200 to 0.00308; p < 0.0001), with a treatment effect–specific intercept of 0.61 ms (90% CI −0.038 to 1.253). No effect on ΔΔQTcF >10 ms is expected up to GSK’254 plasma concentrations of ∼3070 ng/mL (Figure 1). Least squares (LS) mean ΔΔQTcF for GSK’254 100 mg followed the placebo pattern across time points (maximum LS mean ΔΔQTcF, 1.7 ms); the upper bound of the 90% CI remained <10 ms. Maximum LS mean ΔΔQTcF for GSK’254 500 mg exceeded the 10‐ms threshold: 10.6 ms (90% CI 7.75 to 13.38). Neither GSK’254 dose had clinically relevant effects on heart rate or cardiac conduction (i.e. PR and QRS intervals).

**Conclusions**: No clinically relevant effects on QTc prolongation, heart rate, or cardiac conduction were seen in healthy participants at concentrations associated with projected therapeutic GSK’254 doses being evaluated in phase IIb studies. These results support continued clinical development of GSK’254.

#### P211 Bioequivalence of a paediatric fixed‐dose combination tablet darunavir/cobicistat/emtricitabine/tenofovir alafenamide compared with coadministration of the separate agents in healthy adults

S Van Hemelryck ^1^, E Van Landuyt^2^, J Ariyawansa^3^, S Vanveggel^4^, M Palmer^5^

^1^Clinical Pharmacology & Pharmacometrics, Janssen Pharmaceutica NV, Beerse, Belgium; ^2^Medical Department, Janssen Pharmaceutica NV, Beerse, Belgium; ^3^Statistical & Decision Sciences, Janssen Pharmaceutica NV, Beerse, Belgium; ^4^Janssen Pharmaceutica NV, Beerse, Belgium; ^5^Global Clinical Operations, Janssen Pharmaceutica NV, Beerse, Belgium

**Background**: A darunavir/cobicistat/emtricitabine/tenofovir alafenamide (D/C/F/TAF) 800/150/200/10 mg fixed‐dose combination (FDC) tablet is approved for HIV‐1 infection in individuals aged ≥12 years and weighing ≥40 kg. This study evaluated bioequivalence of a D/C/F/TAF paediatric formulation administered as an FDC tablet versus coadministration of separate commercial formulations.

**Table:** 

**Abstract P211 – Table 1**. Statistical analysis summary of darunavir, cobicistat, emtricitabine, and TAF after oral administration of a paediatric formulation of D/C/F/TAF FDC versus coadministration of separate commercial agents.						
Parameter	DRV + COBI + FTC/TAF (reference) N	D/C/F/TAF (test) N	GMR	90% CI	Intra‐individual CV (%) of reference	Intra‐individual CV (%) of test
Darunavir						
C_max_ (ng/mL)	32	32	0.98	0.94 to 1.02	10.3	13.2
AUC_last_ (ng.h/mL)	32	32	1.03	0.98 to 1.08	9.6	9.5
Cobicistat						
C_max_ (ng/mL)	32	32	0.94	0.90 to 0.98	14.2	14.5
AUC_last_ (ng.h/mL)	32	32	0.97	0.93 to 1.02	11.0	10.7
Emtricitabine						
C_max_ (ng/mL)	32	32	0.99	0.94 to 1.04	17.6	17.6
AUC_last_ (ng.h/mL)	32	32	1.01	0.99 to 1.02	6.3	5.8
TAF						
C_max_ (ng/mL)	32	32	1.05	0.91 to 1.22	56.9	49.3
AUC_last_ (ng.h/mL)	32	32	1.13	1.07 to 1.20	23.1	14.8

COBI, cobicistat; CV, coefficients of variation; DRV, darunavir; FTC, emtricitabine.

**Materials and methods**: This phase I, randomised, open‐label, two‐treatment, two‐sequence, four‐period, crossover pivotal bioequivalence study was conducted in healthy adults aged 18 to 55 years. During each period, participants received one treatment (single oral dose of D/C/F/TAF 675/150/200/10 mg [test] or oral darunavir 600 mg and 75 mg, emtricitabine/TAF 200/10 mg FDC, and cobicistat 150 mg [reference]) under fed conditions, sequentially as test‐reference‐reference‐test or reference‐test‐test‐reference, with a ≥7 days washout between periods. As TAF maximum plasma concentration (C_max_) is highly variable, a traditional two‐way crossover design would require a high sample size to achieve sufficient power. Therefore, a replicate crossover design was utilised per regulatory guidelines, allowing widening of TAF C_max_ bioequivalence limits based on observed intra‐individual variability for the reference treatment. A minimum of 28 participants was estimated to yield ≥90% power to establish bioequivalence at a 5% significance level, assuming the test and reference treatment geometric means differed by ≤5%. To meet bioequivalence criteria, 90% confidence intervals (CIs) of the geometric mean ratios (GMRs) for darunavir and emtricitabine C_max_ and area under the plasma concentration‐time curve from time zero to last measurable concentration (AUC_last_) and for TAF AUC_last_, and the GMR for TAF C_max_ needed to fall within 80.00% to 125.00%, inclusive. The 90% CI for TAF C_max_ needed to fall within the calculated widened limits.

**Results**: Thirty‐seven participants were randomised; 32 completed the study (n = 16 per treatment sequence). Most participants were male (64.9%) and White (91.9%). Median (range) age was 26 years (18 to 55 years). Results of inferential statistical analysis of the pharmacokinetic parameters are presented (Table 1). As the 90% CIs of the GMRs for C_max_ and AUC_last_ for darunavir, emtricitabine, and TAF fell within predefined bioequivalence limits, both treatments can be considered bioequivalent. No serious adverse events were reported.

**Conclusions**: Administration of D/C/F/TAF 675/150/200/10 mg as an FDC tablet was bioequivalent to coadministration of separate commercial formulations. Treatment with both was considered safe; no new safety findings emerged.

#### P212 Pharmacokinetic interaction between single and multiple doses of darunavir, in combination with cobicistat or ritonavir, and single‐dose dabigatran etexilate in healthy adults

S Van Hemelryck ^1^, E Van Landuyt^2^, J Ariyawansa^3^, M Palmer^4^, M Kothe^5^, C Pollefliet^5^

^1^Clinical Pharmacology & Pharmacometrics, Janssen Pharmaceutica NV, Beerse, Belgium; ^2^Medical Department, Janssen Pharmaceutica NV, Beerse, Belgium; ^3^Statistical & Decision Sciences, Janssen Pharmaceutica NV, Beerse, Belgium; ^4^Global Clinical Operations, Janssen Pharmaceutica NV, Beerse, Belgium; ^5^Clinical Pharmacology Unit, Janssen Research & Development, Merksem, Belgium

**Background**: Darunavir, coadministered with cobicistat or ritonavir, is a P‐glycoprotein inhibitor. Dabigatran etexilate, prodrug of the anticoagulant dabigatran, is a P‐glycoprotein probe substrate. This study evaluated the effect of single and repeated doses of darunavir, coadministered with cobicistat or ritonavir, on the pharmacokinetics of single‐dose dabigatran etexilate.

**Materials and methods**: In this open‐label, fixed‐sequence, single‐centre, two‐panel, phase I study in healthy adults aged 18 to 60 years, participants were equally divided over two panels. On day 1, all participants were administered dabigatran etexilate 150 mg. On day 4, participants received darunavir/cobicistat 800/150 mg (panel 1) or darunavir/ritonavir 800/100 mg (panel 2) and dabigatran etexilate 150 mg. On days 5 to 20, participants received once‐daily darunavir/cobicistat 800/150 mg (panel 1) or once‐daily darunavir/ritonavir 800/100 mg (panel 2) and, on day 18, single‐dose dabigatran etexilate 150 mg. Drug intakes took place under fed conditions. Key pharmacokinetic parameters were the estimated maximum plasma concentration (C_max_) and area under the plasma concentration‐time curve from time zero to infinity (AUC_inf_) on days 1, 4, and 18 for dabigatran. Safety was assessed throughout the study.

**Results**: Twenty‐eight participants were enrolled and treated (n = 14 per panel); one participant (panel 2) did not complete the study. All participants were White except one participant in panel 2 characterised as 'multiple races'; 64.3% and 35.7% of participants were male in panels 1 and 2, respectively. Median (range) age for panels 1 and 2 was 51.5 (21 to 57) and 54.5 (22 to 59) years, respectively. Dabigatran pharmacokinetic parameters and statistics are presented in Table 1. Dabigatran C_max_ and AUC_inf_ increased 2.64‐fold after a single dose of darunavir/cobicistat and increased 1.99‐ and 1.88‐fold, respectively, after multiple doses of darunavir/cobicistat. Dabigatran C_max_ and AUC_inf_ increased 1.64‐ and 1.72‐fold, respectively, after a single dose of darunavir/ritonavir and increased 1.22‐ and 1.18‐fold, respectively, after multiple doses of darunavir/ritonavir. In both panels, the most commonly reported treatment‐emergent adverse events were diarrhoea and headache.

**Table:** 

**Abstract P212 – Table 1**. Pharmacokinetic parameters and statistical analysis summary of dabigatran alone (day 1) and after single (day 4) and multiple (day 18) doses of darunavir coadministered with cobicistat or ritonavir. Panel 1: treatment A, dabigatran etexilate 150 mg on day 1; treatment B, darunavir/cobicistat 800/150 mg and dabigatran etexilate 150 mg on day 4; treatment C, darunavir/cobicistat 800/150 mg (once daily) on days 5 to 20 and dabigatran etexilate 150 mg on day 18. Panel 2: treatment D, dabigatran etexilate 150 mg on day 1; treatment E, darunavir/ritonavir 800/100 mg and dabigatran etexilate 150 mg on day 4; treatment F, darunavir/ritonavir 800/100 mg (once daily) on days 5 to 20 and dabigatran etexilate 150 mg on day 18.			
Panel 1			
Parameter	Treatment A (dabigatran etexilate)	Treatment B (dabigatran etexilate + single‐dose darunavir/cobicistat)	Treatment C (dabigatran etexilate + multiple doses darunavir/cobicistat)
n	14	14	14
C_max_, mean (SD), ng/mL	130 (41.6)	344 (96.8)	254 (62.5)
t_max_, median (range), h	3.00 (1.50 to 5.00)	4.00 (3.00 to 5.00)	4.00 (3.00 to 5.02)
AUC_inf_, mean (SD), ng.h/mL	1207 (325)	3233 (1054)	2252 (520)
t_1/2_, mean (SD), h	9.3 (1.8)	10.7 (2.7)	10.7 (3.0)
	Comparison	Geometric LSM ratio	90% CI
C_max_, ng/mL	Treatment B vs A	2.64	(2.29 to 3.05)
	Treatment C vs A	1.99	(1.72 to 2.30)
AUC_inf_, ng.h/mL	Treatment B vs A	2.64	(2.32 to 3.00)
	Treatment C vs A	1.88	(1.65 to 2.13)
**Panel 2**			
**Parameter**	**Treatment D (dabigatran etexilate)**	**Treatment E (dabigatran etexilate + single‐dose darunavir/ritonavir**	**Treatment F (dabigatran etexilate + multiple doses darunavir/ritonavir)**
n	14	14	13
C_max_, mean (SD), ng/mL	186 (65.4)	301 (95.2)	266 (116)
t_max_, median (range), h	3.00 (1.00 to 5.00)	4.01 (2.02 to 5.02)	4.02 (1.52 to 5.00)
AUC_inf_, mean (SD), ng.h/mL	1565 (509)	2667 (847)	2068 (877)
t_1/2_, mean (SD), h	8.8 (1.1)	9.8 (2.1)	8.9 (1.8)
	Comparison	Geometric LSM ratio	90% CI
C_max_, ng/mL	Treatment E vs D	1.64	(1.21 to 2.23)
	Treatment F vs D	1.22	(0.89 to 1.67)
AUC_inf_, ng.h/mL	Treatment E vs D	1.72	(1.33 to 2.23)
	Treatment F vs D	1.18	(0.90 to 1.53)

LSM, least squares mean; SD, standard deviation; t_1/2_, half‐life; t_max_, time at C_max_.

**Conclusions**: Findings of increased dabigatran exposure with darunavir/cobicistat and darunavir/ritonavir coadministration indicate an inhibitory effect of single‐dose boosted darunavir on P‐glycoprotein, and a mixed inhibitory/inductive effect of multiple doses of boosted darunavir on P‐glycoprotein. Study treatments were generally safe and well tolerated in healthy adults.

### Models of Care: Evaluation of ARV Delivery and Coverage

#### P213 Use of generic antiretroviral drugs in 2021 in three health centres in the Paris area, France

P Leroy ^1^, S Diamantis^2^, P Sellier^1^, G Hamet^3^, A Brun^3^, W Rozenbaum^3^, J Molina^4^

^1^Maladies Infectieuses et Tropicales, Assistance Publique ‐ Hôpitaux de Paris Hôpital Lariboisière, Paris, France; ^2^Maladies Infectieuses et Tropicales, Groupe Hospitalier Sud Île‐de‐France, Melun, France; ^3^Corevih Île‐de‐France Est, Assistance Publique ‐ Hôpitaux de Paris Hôpital Saint‐Louis, Paris, France; ^4^Maladies Infectieuses et Tropicales, Assistance Publique ‐ Hôpitaux de Paris Hôpital Saint‐Louis, Paris, France

**Background**: Antiretroviral therapy (ART) accounts for 86% of the total cost of HIV care in France. Generic antiretrovirals can reduce this burden. The aim of this study was to evaluate the proportion of generic ART prescription and identify potential factors associated with their prescription.

**Materials and methods**: Multicentric retrospective descriptive study in three health centres in the Paris area (two in Paris, one in the suburban area) including people living with HIV (PLWHIV) aged at least 18 years, taking ART for at least 6 months and who were given at least one prescription of ART (triple therapy only) during year 2021. We analysed the last ART prescription (regimen type, use of international nonproprietary name [INN]), PLWHIV demographics (age, country of birth, gender), HIV parameters (HIV transmission mode, treatment history and duration), physician parameters (age, gender, experience in HIV care and centre). Analysis of risk factors was performed using a multivariate logistic model.

**Results**: Five thousand, two hundred and thirty‐six patients were included, 30.2% of the prescriptions included generic drugs expressed as INN. Emtricitabine/tenofovir disoproxil (FTC/TDF) was the most prescribed generic ART, accounting for 2/3 of all these prescriptions. Women were more likely given generic ART than men (OR 1.65 [1.40 to 1.96], p < 0.001). Generic ART were less frequently prescribed to patients aged more than 50 years (OR 0.85 [0.74 to 0.97], p < 0.05). Fewer generic ART were prescribed in the suburban centre than in Paris. A recent HIV diagnosis (less than 3 years) was associated with a higher use of generic ART. HIV transmission mode, Centers for Disease Control and Prevention (CDC) stage and country of birth did not influence the use of generic ART. Female physicians prescribed less frequently generic ART than male physicians (OR 0.63 [0.55 to 0.72], p < 0.001).

**Conclusions**: Generic ART were prescribed in only one‐third of patients and we identified patients, physicians and centre factors associated with their prescription.

#### P214 Abstract withdrawn

#### P215 Cascade of care in Romanian patients who grew up with HIV from a tertiary care facility

I Ianache^1^, R Radoi^1^, G Tardei^2^, L Ene^1^, C Oprea ^1^

^1^Infectious Diseases, HIV/AIDS, Victor Babes Hospital for Infectious and Tropical Diseases, Bucharest, Romania; ^2^Virology and Molecular Biology, Victor Babes Hospital for Infectious and Tropical Diseases, Bucharest, Romania

**Background**: A particular aspect of HIV epidemic in Romania is the homogenous group of patients, infected by parenteral mode, with subtype F1, in early childhood, in the late 1980s. Among 11 500 reported cases at national level, 45% were in active care at the end of 2021. The aim of the study was to evaluate antiretroviral treatment (ART) coverage and effectiveness in PLWHIV, infected by parenteral mode during childhood (PI) in active care in Victor Babes regional centre (VBH).

**Methods**: Demographic data, ART and HIV parameters (CD4 count and viral loads [VL]) were analysed for patients from VBH. Data from PI was compared with vertically infected patients (VI). Cascade of care for this subpopulation was conducted for year 2021. Patients were considered virally suppressed if their most recent VL, measured within the past year, was less than 50 copies/mL.

**Results**: Out of 2357 PLWHIV in care at VBH, 403 (17.1%) were PI and 58 (2.4%) VI. A total of 29 (6.2%) patients were lost to follow‐up during 2021: 13.5% VI versus 5.2% PI (p = 0.02). The study group included 432 patients, 382 (88.4%) PI and 50 (11.6%) VI. The median age (years) was 33 (IQR 33 to 34) for the PI and 19 (IQR 13 to 24) for VI (p < 0.0001). The proportion of patients on ART was 93.7%, 94.7% in the PI and 94.0% in the VI group. Most patients were on regimens containing INSTI 47.4%, followed by salvage regimens 25.8%, PI/r 18.9% and NNRTI 7.7%. VL was performed for 364 patients, with 78.5% (249/317) undetectable in PI (Figure 1) and 72.3% (34/47) in the VI group (p = 0.20). The median CD4 cell count/μL was 626 (IQR 392 to 880) in PI versus 846 (IQR 429 to 1184) in VI (p = 0.08). Viral suppression was higher in females than in males 52.6% (131/249) in PI versus 38.2% (13/34) in VI (p = 0.082). Virological failure was mainly due to lack of adherence and treatment fatigue in both groups.

**Figure:**
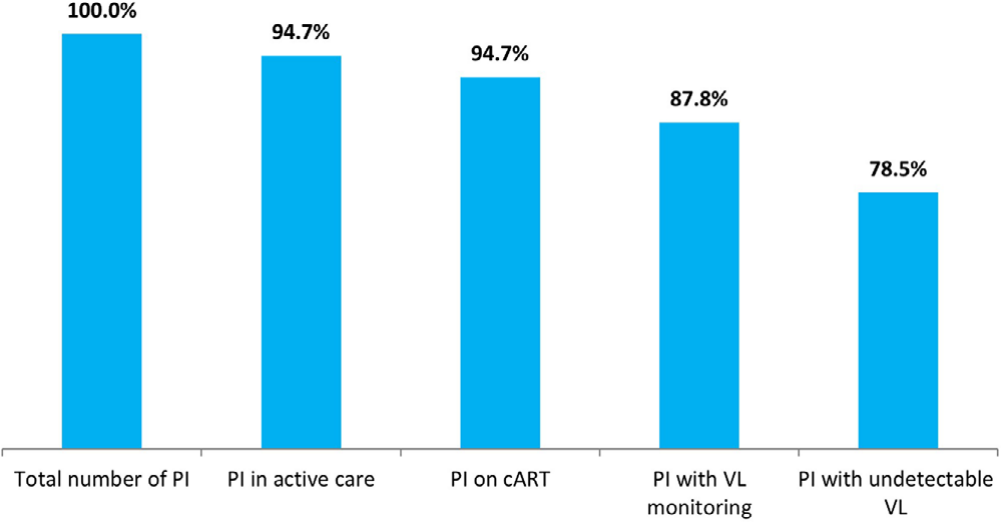
**Abstract P215 – Figure 1**. Cascade of care in PLWHIV parenterally infected during childhood from VBH.

**Conclusions**: Despite a good ART coverage, there are still some barriers in reaching the 95‐95‐95 UNAIDS targets in both groups. Intervention strategies to optimise treatment response and psychological support must be prioritised.

#### P216 Pilot model of the HIV drug resistance testing coverage cascade

A Pokrovskaya ^1^, D Kireev^1^, K Emerole^2^, A Kirichenko^1^, V Pokrovsky^1^

^1^AIDS/HIV Department, Central Research Institute of Epidemiology of Rospotrebnadzor, Moscow, Russian Federation; ^2^Medical Institute, Department of Infectious Diseases, Peoples' Friendship University of Russia, Moscow, Russian Federation

**Background**: The effectiveness of ART and achievement target indicator of the HIV care cascade depends on various factors, including HIV resistance to ART. Prompt HIV viral load testing, detection of virus mutations and appropriate modification of ART regimens are factors for successful HIV treatment and prevention. However, in countries with large PLHIV population and limited resources, HIV drug resistance testing is recommended only in cases of virological failure. Purpose of the study: to test the methodology of the HIV drug resistance testing coverage cascade.

**Materials and methods**: We analysed data from one of the Russian regional HIV/AIDS centres through the year 2021. The data included the number of PLHIV in clinic; number of patients on ART for >12 months; number of patients on ART for >12 months who had HIV viral load (VL) test during the year; number of patients on ART for >12 months with VL <1000 copies/mL at last follow‐up; number of patients on ART for >12 months with a VL >1000 copies/mL tested for HIV drug resistance (DR); number of patients on ART for >12 months who were tested for HIV DR and had at least one major HIV DR mutation; number of patients who switched ART regimen during the year due to virological failure.

**Results**: Eighteen thousand, eight hundred and thirty‐five PLHIV were retained in the HIV care in clinic. Eleven thousand, seven hundred and forty‐nine (62.4%) received ART for >12 months and all of them had VL testing during the year. Among these patients 10 200 (86.8%) had virological response (VL <1000 copies/mL). Out of 1549 patients who had VL >1000 copies/mL, 120 (7.7%) were tested for HIV DR. At least one major HIV DR mutation was found in 66 patients (55% of patients who were tested for HIV DR and 4.2% of patients with VL >1000 copies/mL). Switching of ART regimen due to virological failure during the year were in 483 patients (4.1% of patients on ART for >12 months, 31.2% of patients with VL >1000 copies/mL).

**Conclusions**: Surveillance of HIV drug resistance among PLHIV could supplement the classic HIV care cascade and serve as a new tool for epidemiological surveillance (Figure 1).

**Figure:**
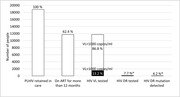
**Abstract P216 – Figure 1**. The HIV drug resistance testing coverage cascade assessment of Russian regional HIV/AIDS centre. * Proportion calculated from patients with VL >1000 copies/mL.

### Virology and Immunology: Biomarkers

#### P217 Residual viraemia and viral blips in the modern cART era: a glimpse beneath the surface

P Oomen, S Dijkstra, A Hoepelman, B van Welzen

Department of Internal Medicine and Infectious Diseases, University Medical Centre Utrecht, Utrecht, Netherlands

**Background**: Viral blips are a common phenomenon in HIV treatment, though their aetiology remains uncertain. One explanation could be that blips are preceded by higher levels of residual viraemia (RV) and are caused by variations around this higher setpoint. Data supporting this hypothesis are limited and (modifiable) factors associated with a lower setpoint are largely unknown. As earlier research showed that integrase inhibitor (INSTI)‐based therapy was associated with lower blip incidences compared to protease inhibitors (PIs) or non‐nucleoside reverse transcriptase inhibitors (NNRTIs), the antiretroviral anchor might influence RV [1]. In this study, we investigate whether RV is associated with blips and the specific anchor that is used.

**Table:** 

**Abstract P217 – Table 1**. Results of multivariable generalised linear mixed model regressions for the occurrence of blips and level of residual viraemia.				
	Occurrence of blips		Level of residual viraemia^a^	
Variables	OR (95% CI)	p‐value	OR (95% CI)	p‐value
Time since study inclusion (per year increase)	0.87 (0.78 to 0.98)	0.02	0.88 (0.86 to 0.90)	<0.001
Female sex (vs male sex)	0.90 (0.61 to 1.31)	0.56	0.90 (0.77 to 1.05)	0.17
Age at start of follow‐up (per year increase)	1.02 (0.89 to 1.19)	0.76	1.00 (1.00 to 1.00)	0.36
cART anchor				
INSTI	−^b^	−^b^	1	−
PI	−^b^	−^b^	1.29 (1.15 to 1.44)	<0.001
NNRTI	−^b^	−^b^	0.76 (0.68 to 0.85)	<0.001
Time since ART initiation (per year increase)	0.97 (0.94 to 1.00)	0.06	0.95 (0.94 to 0.96)	<0.001
Fiebig stage VI at ART initiation (vs stage I to V)	1.99 (0.66 to 6.05)	0.23	1.51 (1.04 to 2.20)	0.03
Lowest available CD4+ count (per 10 cells/mm^3^ increase)	1.00 (0.99 to 1.01)	0.56	0.99 (0.99 to 1.00)	<0.001
Zenith VL copies/mL				
<10 000	1	−	1	−
10 000 to 99 999	2.03 (0.66 to 6.23)	0.22	2.10 (1.54 to 2.85)	<0.001
100 000 to 999 999	2.80 (0.90 to 8.76)	0.08	3.49 (2.59 to 4.70)	<0.001
≥1 000 000	5.16 (1.54 to 17.29)	0.01	5.40 (3.59 to 8.13)	<0.001
Residual viraemia copies/mL				
RNA‐	1	−	−	−
<20 copies/mL	2.72 (2.03 to 3.67)	<0.001	−	−
20 to 49 copies/mL	5.00 (3.49 to 7.17)	<0.001	−	−

cART, combination antiretroviral therapy; INSTI, integrase strand transfer inhibitor; PI, protease inhibitor; VL, viral load.

^a^RV, categorised as an ordinal outcome (RNA‐, <20 copies/mL and 20 to 49 copies/mL), was analysed using backward continuation ratio model ordinal regression. The odds of <20 copies/mL versus RNA‐ and 20 to 49 copies/mL versus <20 copies/mL and RNA‐ were investigated and since the proportional odds assumption was not violated, one OR was given per variable;

^b^as the variable ‘cART anchor’ is in the causal pathway as a precursor for the association between residual viraemia and the occurrence of viral blips, it was not included in the regression.

**Methods**: All treatment courses in 2010 to 2020 consisting of two nucleos(‐t)ide reverse transcriptase inhibitors and one anchor in virologically suppressed people living with HIV (PLWHIV) were evaluated for RV [viral loads (VLs) with detectable viraemia <50 copies/mL] and blips [isolated VLs 50 to 499 copies/mL between measurements <50 copies/mL]. RV was categorised as RNA‐, <20 copies/mL or 20 to 49 copies/mL. If VLs ≥50 copies/mL were deemed to result from non‐adherence, the course was excluded from analysis. Factors associated with blips and RV were identified using multivariable generalised linear mixed models.

**Results**: In total, 23 596 VLs from 1661 PLWHIV were categorised as blips (332; 1.4%), RNA‐ (15 326; 65.0%), <20 copies/mL (6318; 26.8%) or 20 to 49 copies/mL (1620; 6.9%). Of all VLs, 5082 (21.5%), 8568 (36.3%) and 9946 (42.2%) were during INSTI, PI and NNRTI use. Preceding RV <20 copies/mL and 20 to 49 copies/mL (vs RNA‐) were significantly associated with a 2.72 and 5.00 higher odds of a blip, respectively (Table 1). Moreover, compared with INSTIs, PIs had significantly higher odds of RV (OR 1.29) whereas NNRTIs had lower odds (OR 0.76). In the analysis, several other non‐modifiable parameters were identified to be associated with RV, displayed in Table 1.

**Conclusions**: In this large cohort, we show that viral blips are associated with higher RV levels. Among several other factors, NNRTI and INSTI use was associated with lower RV levels compared with PIs. These findings suggest blips having a multifactorial origin, with RV attributable to anchor type partially contributing to this phenomenon.

**Reference**

1. Dijkstra S, Hofstra LM, Mudrikova T, Wensing AMJ, Oomen PGA, Hoepelman AIM, et al. Lower incidence of HIV‐1 blips observed during integrase inhibitor–based combination antiretroviral therapy. J Acquir Immune Defic Syndr. 2022;89:575‐82.

#### P218 ACBP and control of HIV in progressors and elite controllers

S Isnard^1^, L Royston^1^, J Lin^1^, B Fombuena^1^, S Bu^1^, S Kant^1^, T Mabanga^1^, C Berini^1^, M Durand^2^, M El‐Far^2^, C Tremblay^2^, N Bernard^1^, G Kroemer^3^, J Routy ^1^

^1^IDIGH, McGill University Health Centre ‐ Research Institute, Montreal, Canada; ^2^CRCHUM, Centre de Recherche du Centre Hospitalier de l'Université de Montréal, Montreal, Canada; ^3^Centre de Recherche des Cordeliers, Unité Inserm U1138, Paris, France

**Background**: HIV elite controllers (EC) have genetic (HLA) and immunometabolic charateristics including autophagy that contribute to HIV control via CD4 and CD8 T‐cell responses [1,2]. Acyl‐coenzyme A binding protein (ACBP) is one of the regulators of autophagy [3]. Intracellularly, ACBP favors bioenergetic autophagic reactions by shuttling acyl‐CoA‐bound lipids to cellular organelles, whereas when secreted, extracellular ACBP inhibits autophagy and increases appetite [4]. As autophagy is elevated in EC and is regulated in part by ACBP, we assessed the influence of ACBP in different groups of people living with HIV (PLWHIV) receiving or not antiretroviral therapy (ART).

**Material and methods**: Plasma levels of ACBP and inflammatory cytokines were assessed by ELISA in 37 EC, 27 ART‐naïve, and 55 ART‐treated PLWHIV, compared to 31 HIV‐uninfected controls. HLA typing was performed by next‐generation sequencing in PBMCs. Intracellular ACBP levels were measured by flow cytometry in PBMCs.

**Results**: Plasma ACBP levels were lower in EC compared to ART‐naïve or ART‐treated PLWHIV (medians of 109.8, 238.7 and 264.6 ng/mL respectively, p < 0.01 for both comparisons), independently of age and sex, and were similar to levels in HIV‐uninfected controls (121.3 ng/mL, p = 0.52). ACBP levels in EC were not associated with protective HLA allele expression, CD4, CD8 T‐cell counts or CD4 T‐cell loss overtime. In contrast, ACBP levels correlated with interleukin (IL)‐1β (r = 0.29, p = 0.03) levels but not with other inflammatory cytokines such as IL‐6, IL‐8, IL‐32 or TNF‐α, even in ART‐treated PLWHIV. Moreover, in EC and in ART‐treated PLWHIV, plasma levels of ACBP were inversely associated with plasma IL‐21 (r = ‐0.54, p < 0.01), a cytokine known to participate in maintenance of anti‐HIV T‐cell function [5‐7]. In both ART‐treated and EC, plasma levels of ACBP inversely correlated with intracellular expression of ACBP in T‐cells (r = ‐0.9, p = 0.02).

**Conclusions**: Independently of HLA types, EC are characterized by lower plasma and higher intracellular levels of ACBP compared to ART‐naïve or ART‐treated PLWHIV [8]. High circulating ACBP levels were associated with innate inflammation (IL‐1β) and low IL‐21 plasma levels. Our findings indicate that high intracellular ACBP levels might promote IL‐21 production and persistent anti‐HIV T‐cell response. The ACBP pathway could constitute an interesting target in HIV cure strategies.

**References**

1. Saag M, Deeks SG. How do HIV elite controllers do what they do? Clin Infect Dis. 2010;51:239‐41.

2. Tarancon‐Diez L, Rodriguez‐Gallego E, Rull A, Peraire J, Vilades C, Portilla I, et al. Immunometabolism is a key factor for the persistent spontaneous elite control of HIV‐1 infection. EBioMedicine. 2019;42:86‐96.

3. Pedro JMB, Sica V, Madeo F, Kroemer G. Acyl‐CoA‐binding protein (ACBP): the elusive 'hunger factor' linking autophagy to food intake. Cell Stress. 2019;3:312‐8.

4. Bravo‐San Pedro JM, Sica V, Martins I, Pol J, Loos F, Maiuri MC, et al. Acyl‐CoA‐binding protein is a lipogenic factor that triggers food intake and obesity. Cell Metab. 2019;30:754‐67.e9.

5. Loucif H, Dagenais‐Lussier X, Avizonis D, Choiniere L, Beji C, Cassin L, et al. Autophagy‐dependent glutaminolysis drives superior IL21 production in HIV‐1‐specific CD4 T cells. Autophagy. 2021:1‐18.

6. Loucif H, Dagenais‐Lussier X, Beji C, Cassin L, Jrade H, Tellitchenko R, et al. Lipophagy confers a key metabolic advantage that ensures protective CD8A T‐cell responses against HIV‐1. Autophagy. 2021;17:3408‐23.

7. Iannello A, Boulassel MR, Samarani S, Debbeche O, Tremblay C, Toma E, et al. Dynamics and consequences of IL‐21 production in HIV‐infected individuals: a longitudinal and cross‐sectional study. J Immunol. 2010;184:114‐26.

8. Isnard S, Royston L, Lin J, Fombuena B, Bu S, Kant S, et al. Distinct plasma concentrations of acyl‐CoA‐binding protein (ACBP) in HIV progressors and elite controllers. Viruses. 2022;14:453.

#### P219 The preservation of thymic activity and the naïve T cell compartment is a hallmark of HIV‐2 and early treated HIV‐1 individuals

G Farias^1^, R Marques^1^, D Santos^1^, A Gomes^1^, Z Junginger^1^, A Sousa^1^, R Badura ^2^

^1^Clinical Immunology, Instituto de Medicina Molecular João Lobo Antunes, Faculdade de Medicina, Universidade de Lisboa, Lisbon, Portugal; ^2^Department for Infectious Diseases, Hospital de Santa Maria, Centro Hospitalar Universitário Lisboa Norte, Lisbon, Portugal

**Background**: Antiretroviral therapy (ART) markedly improved disease prognosis; however, ART‐treated subjects maintain some degree of immune activation eventually associated with comorbidities. Importantly, HIV‐2 infected individuals also feature significant immune activation, despite a relatively benign course and low to undetectable plasma viral load even in the absence of ART. To better understand the mechanisms underlying persistent immune activation, we investigated the correlation profile of immune parameters in circulating lymphocyte subsets of HIV‐1 and HIV‐2 infected individuals under effective ART. We specifically asked which parameters would allow the segregation of: 1) HIV‐2 from HIV‐1 infection; and 2) HIV‐1 individuals who started ART during acute infection (early ART) and those only treated during the chronic stage (late ART).

**Materials and methods**: To profile chronic immune activation, we enrolled patients who started ART while seroconverting (early‐ART, n = 12), late‐ART HIV‐1 infected individuals (n = 11), HIV‐2 infected individuals (n = 9) along with seronegative individuals (n = 11). We stained 5 million circulating leukocytes in whole blood, with a panel of combined 18 markers allowing the discrimination of cell differentiation stages and levels of activation of CD4+ and CD8+ αβ and γδ T cells, NK and B cells by spectral flow cytometry. For a clear and comprehensive visualisation of these populations and their respective phenotypes, we applied dimension reduction and clustering algorithms. We then generated correlograms and PCA analysis to uncover patterns of immune activation, integrating all main lymphocyte subsets and correlate the resulting profile with clinical parameters.

**Results and conclusions**: αβ T cell clusters were the main segregators of HIV‐1 infected patients treated early versus later. The CD31+ naïve CD4+ T cell cluster, which includes the recently produced T cell in the thymus (recent thymic emigrants, RTEs), distinguishes the three HIV infected cohorts, indicating that HIV‐2 and early treated HIV‐1 patients better preserve thymic function. In addition to αβ, B cell clusters contribute to the segregation of HIV‐2 infected individuals, which may be related to the impact of prolonged HIV‐2 disease in the secondary lymphoid organs. Finally, there was an overlap of the immune profile of early treated HIV‐1 infected and seronegative cohorts, emphasising the relevance of early ART.

#### P220 Reactive oxygen species (ROS) in T‐cell subsets of HIV+cART naive and HIV+cART+ individuals

R Emilova ^1^, Y Todorova^1^, M Aleksova^1^, I Alexiev^2^, L Grigorova^2^, R Dimitrova^2^, N Yancheva^3^, M Nikolova^1^

^1^National Reference Laboratory of Immunology, National Center of Infectious and Parasitic Diseases, Sofia, Bulgaria; ^2^National Reference Confirmatory Laboratory of HIV, National Center of Infectious and Parasitic Diseases, Sofia, Bulgaria; ^3^Department for Treatment of Acquired Immunodeficiency, Specialized Hospital for Active Treatment of Infectious and Parasitic Diseases, Sofia, Bulgaria

**Background**: Cellular activation and chronic inflammation are consequences of the increased production of ROS. Identifying and understanding the mechanisms of oxidative stress in HIV infection is an important element of an integrated approach to antiretroviral therapy (cART) monitoring.

**Aim**: To compare intracellular ROS in CD4+ and CD8+ T‐cells of cART‐naive HIV+ individuals (cART‐HIV+) to those on continuous cART (HIV+cART+), with suppressed HIV viral load (VL), and to HIV‐negative healthy volunteers.

**Materials and methods**: Peripheral blood samples (Li‐heparin) were collected from cART+HIV+ with sustained viral suppression and HIV VL <40 copies/mL (A, n = 28), cART‐HIV+ individuals (B, n = 10) with HIV VL >1000 copies/mL, and HIV‐ volunteers (C, n = 10) of similar age and sex. HIV VL was determined in plasma by Abbott RT (LLOD 40 copies/mL). Absolute count (AC) of CD4+ and CD8+ T‐lymphocytes were determined by flow cytometry. Intracellular ROS were determined by incubating CD4+ and CD8+ stained peripheral blood mononuclear cells (PBMCs) at 37°C for 30 minutes with a sensor, forming fluorescent ROS complex (λex = 520/λem = 605 nm). ROS levels were quantified according to the mean fluorescence intensity (MFIROS/100) by flow cytometry (FACSDiva 6.1.2).

**Results**: No difference in CD4AC was found between groups A and C in contrast to group B (935±261 vs 866±434, p = 0.66 vs 422±296, p < 0.01). The CD4/CD8 ratio in both patients’ groups was lower as compared to group C (1.4±0.4 and 0.5±0.4, vs 2.4±0.8, p < 0.001) (Figure 1A). MFI_ROS_ in CD4+T was significantly higher in both HIV+ groups as compared to C (28.8±12.3 and 44.3±23.6 vs 18.3±7.9, p < 0.01 for both) (Figure 1B). MFI_ROS_ in CD8+T was not significantly different between groups A and C (30.6±11.9 vs 22.9±11.6, p = 0.11) while in group B we observed significantly higher levels (40.8±16.5, p < 0.01) (Figure 1C). Noteworthy, MFI_ROS_ in CD4+T correlated positively with HIV VL (R = 0.4, p < 0.01) and inversely with CD4/CD8 ratio (R = ‐0.4, p < 0.01) (Figure 1D), unlike MFI_ROS_ in CD8+T.

**Conclusions**: MFI_ROS_ in CD4+T production may be an indicator of residual HIV activity in the settings of undetectable HIV VL. A better understanding of the relationship between ROS in CD8 and CD4 T cells could lead to improved cART monitoring.

**Figure:**
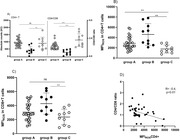
**Abstract P220 – Figure 1**. (A) Absolute count of CD4+ cells and CD4/CD8 ratio in HIV+ individuals on continuous cART with suppressed viral load (group A), HIV+ cART‐naive people with detectable VL (group B) and HIV‐negative volunteers (group C); (B) MFI_ROS_ in CD4+T‐cells in group A, group B and group C; (C) MFI_ROS_ in CD8+T‐cells in group A, group B and group C; (D) correlation between MFI_ROS_ in CD4+T and CD4/CD8 ratio in HIV+ individuals.

### Virology and Immunology: Resistance

#### P221 Development of second‐generation integrase inhibitor resistance over the last 6 years in Germany

F Wiesmann, A Maennling, G Naeth, P Braun, H Knechten

Viral Diagnostics, PZB Aachen, Aachen, Germany

**Background**: Selection of HIV‐1 drug resistance mutations under second‐generation integrase inhibitors bictegravir‐ (BIC, approved 2018), dolutegravir‐ (DTG, 2014) and more recently cabotegravir‐ (CAB, 2021) containing treatment has been reported to occur rarely due to a higher genetic barrier against resistance. The focus of this retrospective cohort was to assess the prevalence of major integrase resistance mutations over the last 6 years.

**Materials and methods**: EDTA plasma samples from patients with documented viraemia and suspected resistance development were subject to next‐generation sequencing on an Iseq‐100 sequencer platform. From 2017 to 2018, n = 655 patients from different centres in Germany were tested for development of integrase inhibitor resistance. From 2019 to 2020 n = 710 patients were tested accordingly and n = 667 between 2021 and July 2022. Major integrase resistance mutations at position G118R, Q148H/K/R/N, N155H, S230R and R263K were documented anonymously and associated with historical and current treatment data, viral load, CD4 count and HIV‐1 subtype.

**Results**: Integrase inhibitor resistance was rarely observed and remained on a constantly low level over the years with N155H being the most frequently detected major mutation in the range of 1.8% to 2.9% of resistance tests. About 50% of patients with available historic resistance data showed prior NRTI and/or NNRTI resistance. The development of G118R occurred in just two cases between 2021 and 2022. Substitutions at position Q148 increased from 0.9% to 1.1% and 2.1% over the years. Of seven specified pretreatments, three Q148 substitutions were most likely associated with a failing CAB‐treatment, and two associated with EVG or RAL each, respectively. The prevalence of substitution R263K increased from 0.15% to 0.85% and 1.2%, respectively. Of seven specified pretreatments, six R263K substitutions were most likely associated with failing DTG‐treatment, and one associated with CAB.

**Conclusions**: Integrase inhibitor resistance was rarely observed and remained on a constantly low level over the years. Pretreatment data suggests that a major part of these patients already had anamnestic NRTI and/or NNRTI resistance before re‐analysis. All documented cases were viraemic. The increasing amount of patients receiving integrase inhibitor‐containing treatment may explain the slight increase of detected substitutions at position Q148 and R263 over time but should be further documented.

#### P222 Long‐term efficacy and resistance analyses of D/C/F/TAF in the phase III AMBER and EMERALD studies: post 96‐week data

E Lathouwers ^1^, B Baugh^2^, S Vanveggel^3^, E Van Landuyt^4^, M Opsomer^4^, C Orkin^5^, J Eron^6^

^1^Clinical Virology, Janssen Pharmaceutica NV, Beerse, Belgium; ^2^Medical Affairs, Janssen Research & Development, LLC, Raritan, NJ, USA; ^3^Biostatistics, Janssen Pharmaceutica NV, Beerse, Belgium; ^4^Medical, Janssen Pharmaceutica NV, Beerse, Belgium; ^5^Department of Infection and Immunity, Queen Mary University of London, Royal London Hospital, London, UK; ^6^Department of Medicine, University of North Carolina at Chapel Hill School of Medicine, Chapel Hill, NC, USA

**Background**: Treatment through 96 weeks (wks) with once‐daily D/C/F/TAF has demonstrated high virologic suppression rates, a high genetic barrier, and a favorable safety profile in treatment‐naïve adults in AMBER and treatment‐experienced, virologically suppressed adults in EMERALD. We evaluated the long‐term efficacy and the resistance to D/C/F/TAF treatment beyond wk96.

**Materials and methods**: In AMBER, treatment‐naive adults with HIV‐1 were randomized to D/C/F/TAF or control (DRV/C + F/TDF followed by a switch to open‐label D/C/F/TAF after wk48). In EMERALD, virologically suppressed, treatment‐experienced adults with HIV‐1 were randomized to D/C/F/TAF or control (continuing a boosted PI with F/TDF and switched to D/C/F/TAF at wk52). During the post‐wk96 extension phase, patients attended visits every 6 months (m). Patients exited the study when commercially available D/C/F/TAF became available, when they switched to other ARVs or discontinued. Proportion of patients with HIV‐1 RNA <50 copies/mL (observed case) post‐wk96 in the D/C/F/TAF group and control group was analyzed. Samples for genotyping/phenotyping were selected in patients with protocol‐defined VF (PDVF) and with VL ≥400 copies/mL at failure or later timepoints. Resistance development post‐wk96 and overall is presented.

**Results**: In AMBER, 311 patients in the D/C/F/TAF group and 310 (control group) continued D/C/F/TAF treatment in the extension phase of which 290 (93.2%) and 284 (91.6%), respectively, completed the study. The post‐wk96 mean D/C/F/TAF exposure was 67.4 and 71.5 weeks in the D/C/F/TAF and control group, respectively. In EMERALD, 699 patients in the D/C/F/TAF group and 337 (control group) continued with D/C/F/TAF treatment in the extension phase of which 648 (92.7%) and 314 (93.2%), respectively, completed the study. The mean D/C/F/TAF exposure post‐wk96 in both groups was ∼84 wks. Post‐wk96 observed virologic response rates are provided in Table 1. In the D/C/F/TAF groups across both studies, post‐wk96, only one AMBER patient was observed to develop an M184M/V FTC RAM (at wk96+30m). In both AMBER and EMERALD, over the entire treatment period, including the extension phase, no patients developed DRV or TFV (delivered as TDF or TAF) RAMs (Table 1).

**Conclusions**: In AMBER (treatment‐naïve adults) and EMERALD (treatment‐experienced, virologically suppressed adults), long‐term D/C/F/TAF treatment was considered efficacious with a high genetic barrier to resistance development.

**Table:** 

**Abstract P222 – Table 1. Proportion of patients with HIV RNA <50 copies/mL (observed case) by analysis time point in the extension phase of AMBER and EMERALD, ITT**.				
	AMBER	AMBER	EMERALD	EMERALD
	D/C/F/TAF	Control	D/C/F/TAF	Control
Analysis set: Intent‐to‐treat, N	311	310	699	337
				
Week 96 + 6 months (N)	303	296	688	334
<50 copies/mL, n (%)	296 (97.7)	285 (96.3)	673 (97.8)	327 (97.9)
				
Week 96 + 12 months (N)	194	214	611	302
<50 copies/mL, n (%)	192 (99.0)	207 (96.7)	601 (98.4)	294 (97.4)
				
Week 96 + 18 months (N)	158	167	461	225
<50 copies/mL, n (%)	155 (98.1)	164 (98.2)	459 (99.6)	222 (98.7)
				
Week 96 + 24 months (N)	81	92	280	134
<50 copies/mL, n (%)	79 (97.5)	88 (95.7)	278 (99.3)	132 (98.5)
				
Week 96 + 30 months (N)	57	58	135	64
<50 copies/mL, n (%)	54 (94.7)	53 (91.4)	134 (99.3)	64 (100)
				
Week 96 + 36 months (N)	19	16^a^	52	26
<50 copies/mL, n (%)	19 (100.0)	11 (68.8)	51 (98.1)	26 (100)
				
Week 96 + 42 months (N)	NA	NA	16	7
<50 copies/mL, n (%)	NA	NA	16 (100)	7 (100)

^a^Of the 5 patients with HIV RNA ≥50 copies/mL, 3 had HIV RNA <200 copies/mL.

#### P223 Reverse transcriptase M184V resistance mutation: back to the future?

E Teyssou^1^, C Soulie^1^, R Palich^2^, A Nouchi^2^, B Abdi^1^, C Katlama^2^, M Wirden^1^, V Pourcher^2^, A Marcelin^1^, V Calvez ^1^

^1^Laboratoire de Virologie, Sorbonne Université, INSERM, IPLESP, AP‐HP, Hôpitaux Universitaires Pitié Salpêtrière ‐ Charles Foix, Paris, France; ^2^Service des Maladies Infectieuses, Sorbonne Université, INSERM, IPLESP, AP‐HP, Hôpitaux Universitaires Pitié Salpêtrière ‐ Charles Foix, Paris, France

**Background**: With life expectancy HIV‐infected patients, optimisation of antiretroviral therapy (ART) is a key challenge considering the resistance and toxicities past histories. Resistance‐associated mutations (RAMs) are archived in the HIV reservoir, at least for years, and can re‐emerge with an inappropriate ART use limiting treatment options. However, recent studies, using ultra‐deep sequencing (UDS), showed a decrease of the amount of quasispecies harbouring RAMs, suggesting that a recycling of some antiretrovirals could be considered and some trials and academic studies suggest this could be feasible in some cases. The aim of this study was to characterise the precise kinetics of the M184V mutation decrease in proviral DNA, to establish a predictive model of M184V mutation clearance over time.

**Materials and methods**: We selected 25 HIV‐infected treated patients virally suppressed (HIV RNA <50 copies/mL) for at least 5 years with, in all cases, the M184V resistance mutation detectable using Sanger or UDS sequencing. UDS was performed on HIV DNA from frozen blood cells at different time points to quantify the percentage of M184V positive quasispecies. The sequence reads were analysed with a minimum coverage set at 50 and an ambiguity filter at 5% or 2%.

**Results**: We analysed the UDS results of 25 HIV patients (19 male and six female, with median age of 56 years [49 to 65] and CD4 cell count of 579/mm^3^ [478 to 779]). At 5 years, using an ambiguity filter at 2%, M184V mutation was no more detected for 64% of patients with a slope of decrease of ‐13.56% per year (Figure 1A) and using 5% ambiguity filter, for 73% of patients with a slope of decrease of ‐15.01% per year (Figure 1B). Survival curve linear regressions predict that the M184V mutation will become undetectable in HIV DNA of patients after at least 6.7 years using the <2% filter or 6.4 years using the <5% filter.

**Conclusion**: Our study provides new information concerning the clearance speed of M184V mutation over time in patients with fully suppressed viraemia and opens the discussion about duration needed to consider a 3TC/FTC recycling.

**Figure:**
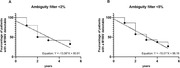
**Abstract P223 – Figure 1**. Clearance speed of the M184V mutation over time in HIV‐infected patients. Survival curves for the percentage of patients with a M184V detectable over time with an ambiguity filter at <2% (A) or at <5% (B).

#### P224 Phenotypic analyses of clinical isolates with capsid substitutions observed in people with HIV (PWHIV) treated with lenacapavir

N Margot ^1^, V Naik^1^, M Hendricks^2^, L VanderVeen^1^, S Yant^2^, C Callebaut^1^

^1^Clinical Virology, Gilead Sciences Inc., Foster City, CA, USA; ^2^Biology, Gilead Sciences Inc., Foster City, CA, USA

**Background**: Lenacapavir (LEN) is an investigational, first‐in‐class, long‐acting inhibitor of HIV‐1 capsid (CA) function suitable for twice‐yearly subcutaneous administration. Of the >250 PWHIV treated with LEN to date in clinical studies, 12 viremic participants developed CA substitutions (M66I, Q67H/K/N, K70H/N/R/S, N74D/H/S, A105S/T, and T107C/N) at residues associated with in vitro LEN resistance. Most mutations were unfit in vitro except Q67H. Herein we further characterize the mutations in clinical isolates and site‐directed mutants (SDMs) using multiple distinct infectious assays.

**Materials and methods**: The gag‐PR fragments from patient‐derived plasma virus and associated SDMs were cloned into various HIV‐1 infectious molecular clones and transfected into 293T cells. The fold change (FC) in LEN susceptibility and/or percent replication capacity (RC) relative to wild‐type (WT) were evaluated using multi‐cycle infectious assays in T‐cell lines (MT‐2, CEM‐NKR‐CCR5/Luc), primary human PBMCs, and 293T cells using the single‐cycle PhenoSense Gag‐Pro assay (Monogram Biosciences).

**Results**: A total of 21 clinical isolates and 15 SDMs were generated. The majority of clinical isolates and SDMs containing the M66I substitution in various genetic contexts (n = 12) were non‐infectious in the MT‐2 cytopathic assay except the M66I+Q67H variants (n = 2), which showed high‐level LEN resistance (FC >1000). In the CEM LTR‐driven luciferase reporter assay, isolates with M66I were more successfully phenotyped with a mean LEN FC >1000 (n = 9), with comparable data obtained in the Gag‐Pro single‐cycle assay. The RC of individual isolates with M66I were low, ranging from 0.6 to 24 and 0.5 to 24% of WT in the Gag‐Pro and PMBC assays (n = 10 for each), respectively. Additional mutations provided RC rescue in some combination of mutations. Finally, the Q67H+K70R variant in the absence of M66I was associated with a LEN FC of 31 and 28 in the MT‐2 and CEM assays, respectively, and RC of 67% and 86% in the PBMC and Gag‐Pro assays, respectively.

**Conclusions**: LEN resistance observed in clinical isolates harboring M66I was associated with low RC. Addition of Q67H in isolates with M66I led to partial rescue of RC in some cases. Isolates with Q67H without M66I showed low‐level LEN resistance and near WT RC, confirming prior observations.

#### P225 The Virostar study: analysis of emergent resistance‐associated mutations at first‐ or second‐line HIV‐1 virological failure with second‐generation InSTIs in two‐ and three‐drug regimens

A Marcelin ^1^, C Soulie^1^, M Wirden^1^, C Charpentier^2^, D Descamps^2^, V Calvez^1^

^1^Virology, Pitie‐Salpetriere Hospital, Paris, France; ^2^Virology, Bichat Hospital, Paris, France

**Background**: Second‐generation integrase strand transfer inhibitors (InSTIs) have a high genetic barrier against resistance and potent antiretroviral activity. They are now recommended as components of both two‐ and three‐drug regimens (2DR, 3DR) as initial or switch options in international guidelines. However, there are limited real‐world data on emerging resistance at the time of first‐ or second‐line HIV‐1 virological failure (VF) on these regimens. The Virostar study objective is to analyse emergent resistance‐associated mutations (RAMs) at VF with DTG 2DRs and DTG or BIC 3DRs in these settings.

**Materials and methods**: Retrospective analysis. Study period was 2019 to 2021 in France. Virological failure was defined as the occurrence of two consecutive HIV‐1 plasma viral loads >50 copies/mL. Sanger genotypic resistance assays were performed during standard clinical care at the time of a first‐ or second‐line VF in patients failing treatment with DTG+3TC; DTG+RPV; BIC/FTC/TAF and DTG+ABC/3TC.

**Results**: Few emergent InSTIs and NRTI RAMs were detected at VF with both DTG+3TC and DTG‐ or BIC‐based 3DRs. More emergent NNRTI RAMs were detected with DTG+RPV (see Table 1).

**Table:** 

**Abstract P225 – Table 1**. Emerging RAMs according to two‐ and three‐drug regimens with second‐generation InSTIs.				
Regimens and number of analysed VF	DTG+3TC; n = 26	DTG+RPV; n = 12	BIC/FTC/TAF; n = 150	DTG+ABC/3TC; n = 88
Emerging InSTI RAMs	3.8% (n = 1 N155H)	0% (n = 0)	0.7% (n = 1 E138K)	1.1% (n = 1 G140S+Q148H)
Emerging NRTI RAMs	15.4% (n = 4 M184V)	N/A	2.7% (n = 4 M184V)	6.8% (n = 6 M184V)
Emerging NNRTI RAMs	N/A	41.7% (n = 3 E138A; 1 M230L; 1 K101E)	N/A	N/A

**Conclusions**: Rare emergent InSTI RAMs and few emergent NRTI RAMs were detected in patients failing DTG+3TC and DTG‐ or BIC‐based 3DR at a first‐ or second‐line VF in real‐world settings. More emergent NNRTI RAMs were detected with failure of DTG+RPV.

#### P226 Integrase inhibitors mutational viral load in HIV‐infected pregnant women in Argentina

D Cecchini ^1^, J Sfalcin^2^, I Zapiola^3^, A Gomez^2^, S Fernandez Giuliano^3^, C Rodriguez^1^, L Mammana^3^, A Seravalle^2^, F Fay^2^, G Bugarín^4^, M Bouzas^3^

^1^Infectious Diseases Unit, Hospital Cosme Argerich, Buenos Aires, Argentina; ^2^Molecular Biology, Centro de Diagnóstico Médico de Alta Complejidad Sociedad Anónima ‐ CIBIC, Rosario, Argentina; ^3^Virology Unit, Clinical Analysis Division, Hospital Francisco J. Muñiz, Buenos Aires, Argentina; ^4^Therapeutic Area Head Vaccines ID and Gen Med Cluster South, Merck Sharp & Dohme ‐ MSD, Munro, Argentina

**Background**: Integrase inhibitors (INSTI), raltegravir (RAL) and dolutegravir, constitute a preferred option for antiretroviral therapy (ART) in HIV‐infected pregnant women (HPW) and RAL is recommended as part of neonatal prophylaxis in high‐risk newborns. In HPW population, Argentina has reported moderate to high levels of transmitted drug resistance to non‐integrase drug classes, with high frequency of mutational viral loads (ML) >1000 copies/mL (threshold for highest risk for mother‐to‐child transmission, formal indication of Caesarean section) for non‐nucleoside reverse transcriptase inhibitors. We aim to describe the ML for integrase resistance major and accessory mutations among INSTI‐unexposed HPW of an historical cohort (2008 to 2014).

**Material and methods**: ML was estimated considering baseline viral load value and the obtained frequency of each INSTI resistance mutation by Ultra Deep sequencing (UDS) using a Public Health Agency of Canada genotyping protocol on Miseq sequencer (Illumina) and HyDRA web. Stored baseline samples of 56 INSTI‐naïve HPW were included (38 ART naïve; 18 exposed to other drug classes) for this analysis.

**Results**: Median (interquartile range, IQR) viral load of the cohort was 15 545 (5228 to 47 688) copies/mL.  Subtype B and B/F: 21.4 and 78.5%, respectively. Major INSTI‐mutations were detected at <5% cut‐off sensitivity threshold; overall prevalence of 8.6% (5/56). Median (range) ML (copies/mL) was: 355 (50.2 to 11 705); only one case >1000 copies/mL (1/56; 1.7%), at expenses of a high baseline maternal viral load (487 732 copies/mL). ML for Y143C, Y143S, E92G, E138K, T66I resistance‐associated mutations were: 63.5, 11 705, 50.2, 761.7 and 355 copies/mL, respectively. Accessory mutations were detected mostly with 20% sensitivity threshold; overall prevalence 23.2% (13/56). Median (IQR) ML (copies/mL) was: 23 929 (4009 to 63 158); all cases >1000 copies/mL. The following accessory mutations were described: T97A (2/13), ML: 213 449 and 17 447, respectively; G163K (5/13), median (range) ML: 23 929 (3327 to 62 922) and G163R (6/13), median (range) ML: 28 614 (1317 to 154 567).

**Conclusion**: In a cohort of INSTI‐naïve HPW, major integrase resistance associated mutations rarely exceed highest perinatal transmission risk threshold of 1000 copies/mL, as not predominant within viral quasispecies. Conversely, accessory mutations exceed this threshold with higher risk of potential transmission to the newborn. Potential clinical impact on maternal ART and neonatal prophylaxis remains to be determined.****

### Virology and Immunology: Other

#### P227 Effect of HIV‐1 subtype C transactivator of transcription (Tat) P21A variant on nuclear levels of active positive transcription elongation factor b (P‐TEFb), TAR binding and disease outcome

Z Mkhize ^1^, D Boehm^2^, R Crespo‐Galvan^3^, M Ott^2^, T Mahmoudi^3^, T Ndung'u^1^, P Madlala^1^

^1^HIV Pathogenesis Program, University of KwaZulu‐Natal, Durban, South Africa; ^2^Gladstone Institutes, University of California San Francisco, San Francisco, CA, USA; ^3^Biochemistry, Erasmus Medical Centre, Rotterdam, Netherlands

The HIV‐1 transactivator of transcription (Tat) enhances viral gene transcription and is important for HIV‐1 pathogenesis.  Genetic variation that translates to functional differences has been reported [1,2]. Specifically, HIV‐1 subtype C (HIV‐1C) alanine at position 21 was reported to be associated with reduced Tat transactivation activity [1]. Although it is well established that the Tat binds to the transactivator RNA (TAR) element of the 5’ long terminal repeat (LTR) and subsequently recruits the host positive transcription elongation factor b (P‐TEFb) for efficient viral gene transcription [3], the effect of Tat variation on its ability to recruit P‐TEFb is unknown. Therefore, this study sought to determine the effect of HIV‐1 subtype C Tat P21A mutant alone and/or in combination with other Tat mutations on the ability of Tat to recruit P‐TEFb to 5’ LTR to enhance viral gene transcription. To this effect, site‐directed mutagenesis (SDM) was performed to introduce Tat P21A mutation alone or together with other mutations using designed primers and the Q5 DNA polymerase kit. Transactivation activity of Tat mutants was measured using Tat transactivation assay where the luciferase activity was the measured output in TZM‐bl cell lines. Co‐immunoprecipitation was performed using mutant P21A Tat protein and cycT1 or CDK9 proteins, components of P‐TEFb. Lastly, RNA immunoprecipitation (RNA IP) was performed using stably expressing TatA21 and TatP21 in Jurkat cells. Our data show HIV‐1C P21A mutant had significantly lower transactivation (p = 0.0004) compared to wildtype Tat. Moreover, TatA21 and TatP21 formed a complex with cycT1 and CDK9. The RNA IP results revealed that TatP21 had more TAR bound by mRNA expression shown by qPCR (p = 0.0151). Taken together, our data shows that HIV‐1C TatA21 significantly reduced its transactivation activity but does not affect its ability to recruit P‐TEFb. Interestingly, TatP21 is able to bind TAR more efficiently than TatA21 thus revealing a possible mechanism but which the reduced functionality in SDMs and in patients was observed. Further investigation is required to determine whether these Tat variants play a role in latency development or reversal.

**References**

1. Kandathil AJ, Kannangai R, Abraham OC, Pulimood SA, Sridharan G. Amino acid sequence divergence of Tat protein (exon1)of subtype B and C HIV‐1 strains: does it have implications for vaccine development? Bioinformation. 2009;4:237‐41.

2. Rossenkhan R, Macleod IJ, Sebunya TK, Castro‐Nallar E, Mclane MF, Musonda R, et al. tat Exon 1 exhibits functional diversity during HIV‐1 subtype C primary infection. J Virol. 2013;87:5732‐45.

3. Jeang KT, Xiao H, Rich EA. Multifaceted activities of the HIV‐1 transactivator of transcription, Tat. J Biol Chem. 1999;274:28837‐40.

#### P228 Initial plasma HIV‐1 RNA and CD4+ T‐cell count are determinants of virological outcomes with initial integrase inhibitor‐based regimens: a prospective multinational RESPOND cohort consortium

H Álvarez ^1,2^, A Mocroft^3,4^, L Ryom^3,5^, B Neesgaard^3^, S Edwards^6^, V Svedhem‐Johansson^7^, H Günthard^8^, R Zangerle^9^, C Smith^10^, A Castagna^11^, A d'Arminio Monforte^12^, F Wit^13^, M Stecher^14^, C Lehman^14^, C Mussini^15^, E Fontas^16^, E González^17^, J‐C Wasmuth^18^, A Sönnerborg^19^, S De Wit^20^, N Chkhartishvili^21^, C Stephan^22^, K Petoumenos^23^, N Jaschinski^3^, V Vannappagari^24^, J Rooney^25^, L Young^26^, A Volny Anne^27^, L Greenberg^3,4^, R Martin‐Iguacel^28^, E Poveda^29^, J Llibre^30^, for the RESPOND (International Cohort Consortium of Infectious Diseases) Study Group

^1^Infectious Diseases Unit, Department of Internal Medicine, Complexo Hospitalario Universitario de Ferrol, SERGAS, A Coruña, Spain; ^2^Universidade de Vigo, Vigo, Spain; ^3^CHIP, Department of Infectious Diseases, Rigshospitalet, University of Copenhagen, Copenhagen, Denmark; ^4^Centre for Clinical Research, Epidemiology, Modelling and Evaluation (CREME), Institute for Global Health, University College London, London, UK; ^5^Department of Infectious Diseases 144, Hvidovre University Hospital, Copenhagen, Denmark; ^6^Department of HIV, Mortimer Market Centre, London, UK; ^7^Infectious Diseases Department, Karolinska University Hospital, Stockholm, Sweden; ^8^Swiss HIV Cohort Study (SHCS), University of Zurich and University Hospital Zurich, Zurich, Switzerland; ^9^Austrian HIV Cohort Study (AHIVCOS), Medizinische Universität Innsbruck, Innsbruck, Austria; ^10^The Royal Free HIV Cohort Study, Royal Free Hospital, University College London, London, UK; ^11^San Raffaele Scientific Institute, Università Vita‐Salute San Raffaele, Milano, Italy; ^12^Italian Cohort Navie Antiretrovirals (ICONA), ASST Santi Paolo e Carlo, Milano, Italy; ^13^AIDS Therapy Evaluation in the Netherlands (ATHENA) Cohort, HIV Monitoring Foundation, Amsterdam, the Netherlands; ^14^University Hospital Cologne, Cologne, Germany; ^15^Modena HIV Cohort, Università degli Studi di Modena, Modena, Italy; ^16^Nice HIV Cohort, Université Côte d'Azur et Centre Hospitalier Universitaire, Nice, France; ^17^PISCIS Cohort Study, Centre Estudis Epidemologics de ITS i VIH de Catalunya (CEEISCAT), Badalona, Spain; ^18^University Hospital Bonn, Bonn, Germany; ^19^Swedish InfCare HIV Cohort, Karolinska University Hospital, Stockholm, Sweden; ^20^Centre de Recherche en Maladies Infectieuses Association Sans But Lucratif, CHU Saint‐Pierre, Brussels, Belgium; ^21^Georgian National AIDS Health Information System (AIDS HIS), Infectious Diseases, AIDS and Clinical Immunology Research Center, Tbilisi, Georgia; ^22^Frankfurt HIV Cohort Study, Infectious Diseases Unit, University Hospital Frankfurt, Goethe‐University, Frankfurt, Germany; ^23^The Kirby Institute, University of New South Wales, Sydney, NSW, Australia; ^24^ViiV Healthcare, Research Triangle Park, Raleigh, NC, USA; ^25^Gilead Sciences, Foster City, CA, USA; ^26^Medical Affairs, Merck Sharp & Dohme, Brussels, Belgium; ^27^European AIDS Treatment Group (EATG), Brussels, Belgium; ^28^Infectious Diseases Department, Odense University Hospital, Odense, Denmark; ^29^Group of Virology and Pathogenesis, Galicia Sur Health Research Institute (IIS Galicia Sur)‐Complexo Hospitalario Universitario de Vigo, SERGAS‐UVigo, Vigo, Spain; ^30^Infectious Diseases Division and Fight Infections Foundation, University Hospital Germans Trias i Pujol, Badalona, Spain.

**Background**: There are conflicting data regarding determinants of virological outcomes in people living with HIV who initiate antiretroviral treatment (ART) [1‐4]. We evaluated the impact of different baseline variables in the prospective multinational RESPOND cohort consortium.

**Methods**: We included participants aged >18, treatment‐naïve, who initiated a three‐drug ART regimen (two nucleosides and either dolutegravir, raltegravir, elvitegravir, darunavir or rilpirivine), 2014 to 2019. We assessed the odds of virological suppression (VS) (HIV‐1 RNA <50 copies/mL) at weeks 48 and 96, using logistic regression. The incidence of viral blips (isolated HIV‐1 RNA >50 copies/mL), low‐level viraemia (LLV) (>2 consecutive HIV‐1 RNA 50 to 199 copies/mL) and virological failure (VF) (two consecutive HIV‐1 RNA >50, one of them >200 copies/mL) rates were assessed using Cox regression.

**Results**: Out of 3475 eligible participants, 83.5% were male, median age 38 (interquartile range [IQR] 30 to 47) years and 71% initiated integrase inhibitor (INSTI)‐based regimens, of whom 1591 (65%) initiated dolutegravir. Median baseline CD4+ count and HIV‐1 RNA were 383 (IQR 209 to 562) cells/μL and 4.68 (IQR 4.08 to 5.27) log10 copies/mL, respectively. VS at weeks 48 and 96 was achieved in 90.6% and 92.9% participants, respectively. At 12 months, Kaplan‐Meier estimates of the proportion with viral blips were 10.9%, LLV were 3.2% and VF were 4.7%. HIV‐1 RNA >100 000 (compared to 10 000 to 100 000) copies/mL and CD4+ count <200 (compared to >500) cells/μL were negatively associated with VS at weeks 48 and 96, and with higher rates of blips and LLV. Overall, CD4+ count <200 cells/μL was associated with higher risk of VF. Results were consistent in those starting INSTIs versus other regimens or those initiating dolutegravir compared to other INSTIs (p > 0.5, tests for interaction) (Table 1).

**Conclusions**: Initial high HIV‐1 RNA and low CD4+ T‐cell counts are associated with lower rates of VS at 48 and 96 weeks, and higher rates of viral blips and LLV. Low baseline CD4+ T‐cell counts are associated with higher VF rates. This association remains even in the context of INSTIs including dolutegravir‐based regimens. While we cannot exclude confounding by indication, these findings suggest virological outcomes independence of the antiretroviral regimen received.

**Table:** 

**Abstract P228 – Table 1**. Participants included for each virological outcome and multivariate analysis of factors associated with viral blip, low‐level viraemia, virological failure and virological suppression. Models were adjusted for gender, age, year of ART initiation, HIV transmission route, ethnicity, HBV and HCV status, body mass index, smoking status, origin region, prior AIDS diagnosis, baseline HIV‐1 RNA, baseline CD4+ T‐cell count and initial ART classes.					
Virological outcome	Viral blip	Low‐level viraemia	Virological failure	Virological suppression week 48	Virological suppression week 96
N included	2977	2977	3116	3055	2435
N with endpoint	448	113	162	2767	2262
% (95% CI)	N/A	N/A	N/A	90.6 (89.5 to 91.6)	92.9 (91.9 to 93.9)
K‐M % at 12 months (95% CI)	10.9 (7.9 to 13.9)	3.2 (1.5 to 4.9)	4.7 (2.7 to 6.7)	N/A	N/A
Multivariate analysis	Adjusted HR (95% CI), p‐value	Adjusted HR (95% CI) p‐value	Adjusted HR (95% CI) p‐value	Adjusted OR (95% CI) p‐value	Adjusted OR (95% CI) p‐value
HIV viral load, HIV‐1 RNA copies/mL					
10 000 or under	0.52 (0.36 to 0.75), <0.001	0.45 (0.17 to 1.18), 0.10	0.70 (0.44 to 1.11), 0.13	1.41 (0.91 to 2.20), 0.13	1.18 (0.69 to 2.00), 0.55
10 001 to 100 000	1.0 (Ref)	1.0 (Ref)	1.0 (Ref)	1.0 (Ref)	1.0 (Ref)
>100 000	1.94 (1.55 to 2.42), <0.001	2.54 (1.58 to 4.07), <0.001	0.90 (0.61 to 1.32), 0.58	0.51 (0.37 to 0.70), <0.001	0.57 (0.38 to 0.84), 0.004
CD4+ T‐cell count, cells/μL					
200 or under	2.84 (2.07 to 3.89), <0.001	7.87 (3.40 to 18.20), <0.001	2.94 (1.78 to 4.87), <0.001	0.42 (0.28 to 0.62), <0.001	0.40 (0.24 to 0.66), <0.001
201 to 350	2.07 (1.51 to 2.82), <0.001	5.13 (2.19 to 11.98), <0.001	1.02 (0.58 to 1.79), 0.95	0.63 (0.41 to 0.95), 0.03	0.57 (0.34 to 0.93), 0.02
351 to 500	1.33 (0.96 to 1.85), 0.09	3.13 (1.29 to 7.60), 0.01	1.34 (0.82 to 2.20), 0.25	0.93 (0.60 to 1.44), 0.75	1.35 (0.76 to 2.39), 0.31
>500	1.0 (Ref)	1.0 (Ref)	1.0 (Ref)	1.0 (Ref)	1.0 (Ref)

K‐M, Kaplan‐Meier; N, number of participants; N/A, not applicable.

**References**

1. Jacobs JL, Halvas EK, Tosiano MA, Mellors JW. Persistent HIV‐1 viremia on antiretroviral therapy: measurement and mechanisms. Front Microbiol. 2019;10:2383.

2. Young J, Rickenbach M, Calmy A, Bernasconi E, Staehelin C, Schmid P, et al. Transient detectable viremia and the risk of viral rebound in patients from the Swiss HIV Cohort Study. BMC Infect Dis. 2015;15:382.

3. Bachmann N, von Siebenthal C, Vongrad V, Turk T, Neumann K, Beerenwinkel N, et al. Determinants of HIV‐1 reservoir size and long‐term dynamics during suppressive ART. Nat Commun. 2019;10:3193.

4. Halvas EK, Joseph KW, Brandt LD, Guo S, Sobolewski MD, Jacobs JL, et al. HIV‐1 viremia not suppressible by antiretroviral therapy can originate from large T cell clones producing infectious virus. J Clin Invest. 2020;130:5847‐57.

#### P229 COVID‐19 pandemic and continuum of care in a cohort of people living with HIV: will we have a price to pay in the future?

B Bellocchi ^1^, M Ceccarelli^2^, R Bruno^1^, L Todaro^1^, E Pistarà^1^, C Giarratana^1^, V Boscia^1^, G Nunnari^3^, B Cacopardo^2^, B Celesia^1^

^1^Unit of Infectious Diseases, Azienda Ospedaliera di Rilievo Nazionale e di Alta Specializzazione Garibaldi, Catania, Italy; ^2^Department of Clinical and Experimental Medicine, University of Catania, Catania, Italy; ^3^Department of Clinical and Experimental Medicine, University of Messina, Messina, Italy

**Background**: COVID‐19 pandemic had a considerable impact on Italian healthcare system which faced the emergency of the epidemic by ensuring continuum of care. Due to the severe restrictive measures, including quarantine and social distancing, PLWHIV were at risk of interrupting follow‐up and treatment refill.

**Materials and methods**: This is a retrospective observational study. We registered the number of virological and immunological blood tests performed during the period before and after COVID‐19 pandemic in a cohort of PLWHIV comparing the number of clinical access and the trend of median CD4 cells count and percentage of detectable and undetectable viraemia. Data were retrieved from administrative and paper clinical files.

**Results**: Data from 763 patients, 25% cis‐women, were analysed. Median age was 49.6 (IQR 39.7 to 57.6). Globally 4099 visits were registered (1217, 970, 1272 and 640 respectively from January 2019 to June 2022). We observed a rise in missed visits at the onset of the pandemic (n = 0) with a compensatory increase in July 2020 (n = 196). The number of CD4 and viral load determinations per patient moved from 2.15 tests in 2019, to 1.61 in 2020, 1.89 in 2021 to 1.12 in 2022 (first 6 months). The median value of CD4 cell count move from 648 (IQR 460 to 835) in 2019 to 619 (IQR 433 to 839) in 2020 and 691 (IQR 489 to 900) in 2021 and 703 (IQR 506 to 944) in 2022 with a significant increase from 2020, period in which vulnerable and naive patients were strictly monitored, to 2021 and 2022 (p < 0.05). Data regarding HIV‐RNA viraemia are reported in Figure 1. No differences in the number of new HIV diagnosis were observed in 2020 versus 2019 (n = 36).

**Conclusion**: The COVID‐19 pandemic had the potential to disrupt HIV care continuum outcomes among PLWHIV. Although we globally observed a 20% reduction of follow‐up during 2020, the resilience shown by healthcare systems was highlighted by a high prevalence of maintained viral suppression and a significant increase in median value of CD4 cell count in the last 2 years. We can conclude that ‘the price is right’.

**Figure:**
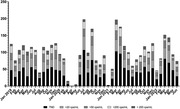
**Abstract P229 – Figure 1**. Cumulative number of viral load determinations per month divided per HIV‐RNA viral load.

#### P230 Recent detectable viral loads among adults living with HIV in the Asia‐Pacific between 2015 and 2020

M Palatino ^1^, D Rupasinghe^2^, A Widhani^3^, N Chi^4^, I Somia^5^, N Kumarasamy^6^, S Khusuwan^7^, V Khol^8^, M Lee^9^, S Kiertiburanakul^10^, T Pham^11^, S Pujari^12^, A Avihingsanon^13^, J Choi^14^, R Chaiwarith^15^, Y Chan^16^, I Azwa^17^, R Ditangco^18^, R Borse^19^, O Ng^20^, J Tanuma^21^, F Zhang^22^, Y Gani^23^, J Ross^24^, A Jiamsakul^2^

^1^Department of Diagnostic Radiology and Nuclear Medicine, University of Maryland School of Medicine, Columbia, SC, USA; ^2^The Kirby Institute, University of New South Wales Sydney, Sydney, Australia; ^3^Faculty of Medicine, Universitas Indonesia, Jakarta, Indonesia; ^4^Department of Infectious Diseases, Bach Mai Hospital, Hanoi, Vietnam; ^5^Faculty of Medicine, Udayana University, Bali, Indonesia; ^6^Antiviral Research / Treatment Clinical Research, Voluntary Health Services, Chennai, India; ^7^Medicine Department, Chiangrai Prachanukroh Hospital, Chiangrai, Thailand; ^8^Medicine, National Center for HIV/AIDS, Dermatology & STDs, Phnom Penh, Cambodia; ^9^Department of Medicine, Queen Elizabeth Hospital, Hongkong, China; ^10^Faculty of Medicine, Ramathibodi Hospital, Mahidol University, Bangkok, Thailand; ^11^Medicine, National Hospital for Tropical Diseases, Hanoi, Vietnam; ^12^Medicine, Institute of Infectious Diseases, Pune, India; ^13^Faculty of Medicine, Chulalongkorn University, Bangkok, Thailand; ^14^Department of Internal Medicine, Yonsei University College of Medicine, Seoul, South Korea; ^15^Department of Medicine, Faculty of Medicine, Chiang Mai University, Chiang Mai, Thailand; ^16^Department of Medicine, Taipei Veterans General Hospital, Taipei, Taiwan; ^17^Department of Medicine, University of Malaya, Kuala Lumpur, Malaysia; ^18^AIDS Research Group, Research Institute for Tropical Medicine, Muntinlupa, Philippines; ^19^Intensive Coronary Unit of Medicine, Byramjee Jeejeebhoy Government Medical College and Sassoon General Hospital, Pune, India; ^20^Department of Infectious Diseases, Tan Tock Seng Hospital, Singapore, Singapore; ^21^AIDS Clinical Center, National Center for Global Health and Medicine, Tokyo, Japan; ^22^Clinical and Research Center of Infectious Diseases, Beijing Ditan Hospital, Capital Medical University, Beijing, China; ^23^Infectious Disease, Hospital Sungai Buloh, Sungai Buloh, Malaysia; ^24^Research, TREAT Asia, amfAR ‐ The Foundation for AIDS Research, Bangkok, Thailand

**Background**: A proportion of people living with HIV (PLWHIV) continue to have detectable viral load (VL) while on robust antiretroviral therapy (ART) regimens, thus, persist to have the risk of onward HIV transmission. We aimed to estimate the proportion and factors associated with having a detectable VL during 2015 to 2020 in a regional Asia‐Pacific research cohort.

**Materials and methods**: We included adult PLWHIV enrolled in the TREAT Asia HIV Observational Database (TAHOD) and TAHOD Low Intensity Transfer (TAHOD‐LITE) cohorts of IeDEA Asia‐Pacific on ART for ≥1 year, in follow‐up between 2015 and 2020, and with ≥1 VL measurement during the follow‐up period. Detectable VL was defined as having ≥1 VL measurement ≥50 copies/mL during 2015 to 2020. Factors associated with detectable VL were analyzed using repeated measures logistic regression (GEE).

**Results**: Of the 20 765 PLWHIV included, the majority were male (64%) and had heterosexual contact as their mode of HIV exposure (72%). During the study period, 6038 (29%) had ≥1 detectable VL. Of these, 2881 (48%) had VL measurements between 50 and <200 copies/mL, 684 (11%) between 200 and <1000, 495 (8%) between 1000 and <5000, and 1978 (33%) at least 5000 copies/mL. Factors associated with decreased odds of detectable VL included female sex (OR 0.84, 95% CI 0.78 to 0.90) compared to males; older age (31 to 40 years: OR 0.68, 95% CI 0.61 to 0.77; 41 to 50: OR 0.56, 95% CI 0.50 to 0.631; ≥51: OR 0.44, 95% CI 0.39 to 0.50) compared to ≤30 years; male‐male sex (OR 0.87, 95% CI 0.77 to 0.98), and injecting drug use (OR 0.66, 95% CI 0.52 to 0.83) compared to heterosexual contact as mode of HIV exposure; non‐nucleoside reverse transcriptase inhibitor‐based ART (OR 0.48, 95% CI 0.44 to 0.52) compared to integrase strand transfer inhibitor (INSTI)‐based regimens; hepatitis B co‐infection (OR 0.80, 95% CI 0.70 to 0.92); hepatitis C co‐infection (OR 0.82, 95% CI 0.69 to 0.97), higher CD4 count (200 to 350 cells/μL: OR 0.34, 95% CI 0.30 to 0.38; 351 to 500: OR 0.20, 95% CI 0.18 to 0.23; >500: OR 0.16, 95% CI 0.15 to 0.18) compared to CD4 <200 cells/μL; and higher country income (upper‐middle: OR 0.22, 95% CI 0.20 to 0.24; high: OR 0.36, 95% CI 0.32 to 0.41) compared to lower‐middle country income. Detectable VL was more likely among those on protease inhibitor‐based ART regimens (OR 1.54, 95% CI 1.40 to 1.69) compared to INSTI‐based ART.

**Conclusions**: Almost one‐third of PLWHIV in our analysis had detectable VL between 2015 and 2020, indicating the importance of maintaining life‐long adherence counseling and follow‐up, particularly among those with increased odds of detectable VL.

#### P231 Contribution to the study of origin, evolution and pathogenesis of CRF19_cpx, a recombinant form of HIV‐1 with high prevalence in Cuba

V Kouri ^1^, L Pérez^1^, Y Martínez^1^, A Zhukova^2^, A Suárez^3^, O Gascuel^4^, Y Pintos^1^, L Machado^3^, E Noa^3^, M Dávila^5^, J Voznica^2^, T To^6^, J Pérez^7^

^1^Virology, Institute of Tropical Medicine 'Pedro Kouri' (IPK), Havana, Cuba; ^2^Department of Computational Biology, Institute Pasteur, Bioinformatics and Biostatistics Hub, Paris, France; ^3^Molecular Biology, Center for Research of Civil Defense, Mayabeque, Cuba; ^4^Department of Computational Biology, Institut de Systématique, Evolution, Biodiversité (ISYEB‐URM 7205 CNRS & Muséum National d'Histoire Naturelle, SU, EPHE & UA), Paris, France; ^5^Laboratory of Applied Mathematics, Technological University of Compiègne, LMAC, Compiègne, France; ^6^Department of Animal and Aquacultural Sciences, Centre for Integrative Genetics (CIGENE), Faculty of Biosciences, Norwegian University of Life Sciences, As, Norway; ^7^Medicine, Institute of Tropical Medicine 'Pedro Kouri' (IPK), Havana, Cuba

**Background**: CRF19_cpx is a recombinant form of HIV‐1 subtypes D, A1 and G, which was first reported in Cuban patients [1]. This variant has spread in Cuba and has been associated with rapid progression to AIDS [2]. This study aims to take a deep‐dive into the origin, evolution and pathogenesis of CRF19_cpx.

**Material and methods**: HIV‐1 subtype was determined in 701 samples from the 2014 to 2019 periods by *pol* gene sequencing [3]. The relationship between the subtypes and the use of the co‐receptor was carried out with 176 plasmas: 107 from the years 2014 to 2016 and 69 from 2017 to 2019. The prediction of the viral phenotype (R5, R5X4 or X4) was generated with Geno2pheno, from partial sequences of the *env* gene [4,5]. To determine the origin and evolution of CRF19_cpx, 350 partial sequences (*pol* and *env*) from Cuban patients and 350 from Los Alamos were used. Analyses were performed using maximum likelihood approaches including: reconstruction of phylogeny, spatio‐temporal analysis of virus spread, reconstruction of ancestral character and mode of transmission [6,7].

**Results**: The most frequent HIV‐1 subtypes were B, CRF19_cpx and CRF_BG (20, 23, 24). CRF19_cpx was associated with the use of the CXCR4 co‐receptor (p < 0.05), being frequent among individuals with a recent diagnosis (36.4%, p = 0.0081). CRF19_cpx was recombined between 1966 and 1977, possibly among the Cuban community stationed in Congo, and was introduced in Cuba in the late 1970s, probably through the province of Villa Clara and then to Havana.

**Conclusions**: The preferential tropism for the CXCR4 co‐receptor, detected in CRF19_cpx, accompanied by greater viral replication and unrelated to the time of diagnosis of the patients, reinforces the hypothesis that this viral variant could have greater pathogenicity. Phylogenetic analysis showed a very early introduction of CRF19_cpx in Cuba, which could explain its epidemiological success.

**References**

1. Casado G, Thomson MM, Sierra M, Najera R. Identification of a novel HIV‐1 circulating ADG intersubtype recombinant form (CRF19_cpx) in Cuba. J Acquir Immune Defic Syndr. 2005;40:532‐7.

2. Kouri V, Khouri R, Aleman Y, Abrahantes Y, Vercauteren J, Pineda‐Pena AC, et al. CRF19_cpx is an evolutionary fit HIV‐1 variant strongly associated with rapid progression to AIDS in Cuba. EBioMedicine. 2015;2:244‐54.

3. Alemán‐Campos Y, Kourí‐Cordellá V, Pérez‐Santos L, Fonseca‐Gómez C, Pérez‐Ávila J, Ortega‐González LM, et al. HIV‐1 antiretroviral resistance in Cuba, 2009‐2014. MEDICC Rev. 2018;20:15‐21.

4. Van Laethem K, Schrooten Y, Lemey P, Van Wijngaerden E, De Wit S, Van Ranst M, et al. A genotypic resistance assay for the detection of drug resistance in the human immunodeficiency virus type 1 envelope gene. J Virol Methods. 2005;123:25‐34.

5. Lengauer T, Sander O, Sierra S, Thielen A, Kaiser R. Bioinformatics prediction of HIV coreceptor usage. Nat Biotechnol. 2007;25:1407‐10.

6. Ishikawa SA, Zhukova A, Iwasaki W, Gascuel O. A fast likelihood method to reconstruct and visualize ancestral scenarios. Mol Biol Evol. 2019;36:2069‐85.

7. Letunic I, Bork P. Interactive Tree Of Life (iTOL): an online tool for phylogenetic tree display and annotation. Bioinformatics. 2007;23:127‐8.

#### P232 Viral reservoir diversity in circulating PMBC and T cell subsets under suppressive ART

Y Zhang, F Otte, T Klimkait

Department of Biomedicine, University of Basel, Basel, Switzerland

**Background**: Even during extended periods of effective, suppressive immune control, substantial viral dynamics are observed in HIV‐1 positive individuals suggesting persisting, continuously active viral reservoirs.

**Materials and methods**: A longitudinal analysis of proviral Env sequences was performed by next‐generation sequencing (NGS) in HIV‐infected individuals from the Swiss HIV Cohort Study right after diagnosis. HIV‐1 proviral load and intracellular viral poly‐A transcripts (pA) were quantified by qPCR. Peripheral blood mononuclear cells (PBMCs), sorted and cultured for 3 weeks for viral outgrowth, were monitored for viral reactivation by Tat‐induced LTR‐activation and HIV‐1 protein expression by FACS. Single‐genome sequencing (SGA) analysis of the 3' half of the HIV‐genomes was carried out to assess re‐activated viral RNA.

**Results**: Nine representative, viraemic patients with at least low HIV‐1 provirus in PBMCs (median: 231/10^6 cells (3 to 4414)) and detectable HIV‐1 pA (median: 1129/10^6 cells (19 to 85003)) were included. Five of nine HIV‐infected individuals maintained a high diversity of their viral reservoirs for extended periods of time after therapy initiation, four of nine of which consisted of unique virus variants (241 to 1454 days). The low viral diversity in the TN and TCM cell subsets largely agreed with the diversity of free viral genomes, while TTM and TEM presented patterns of higher and distinct viral reservoir diversity. In five of nine individuals, certain T‐cell subsets were capable of inducing high HIV‐1 polyA‐RNA, in two individuals, virus from TN and TCM was activated despite persistently low proviral loads and absence of detectable polyA transcripts in the clinical specimen (proviral load: 240 to 2485/10^6 cells). NGS analysis of the re‐activated virus strongly suggests that most of the infectious virus came from virus variants emerging during the outgrowth period. Genomic SGA analysis confirmed the presence of functionally intact virus.

**Conclusions**: Even during continuous virological control in HIV‐infected individuals, certain archived proviral HIV‐1 remains intact in diverse T‐cell reservoirs over very long periods during therapy. Key reservoirs for replication‐competent, infectious virus include mainly TN and TCM, which harbour single or limited numbers of intact viral variants. A distinct, smaller contribution of archived HIV sequences stems from TTM and TEM and is characterised by a higher viral variability. This might form the basis for an ongoing evolution of new viral variants during therapy.

#### P233 Genetic characteristics of a novel HIV‐1 recombinant lineage (CRF103_01B) and its prevalence in northern China

M Dai ^1^, J Li^2^, S Lv^3^, J Li^2^, H Lu^2^, H Huang^4^, R Xin^2^

^1^Institute for School of Public Health, China Medical University, Shenyang, China; ^2^Institute for STD/AIDS Prevention and Treatment, Beijing Center for Disease Prevention and Control, Beijing, China; ^3^Institute for STD/AIDS Diagnosis and Treatment Center, Beijing You An Hospital, Capital Medical University, Beijing, China; ^4^Institute for Department of Infectious Diseases, The Fifth Medical Center of People's Liberation Army General Hospital, Beijing, China

**Background**: The epidemic of HIV‐1 continues to be one of the most significant public health challenges. The circulating strains are genetically complicated, as a consequence of high rates of mutation and high frequency of recombination, especially in Chinese men who have sex with men (MSM).

**Methods**: During the routine HIV‐1 genotype resistance surveillance for optimal antiretroviral therapy (ART) in Beijing, we observed six individuals possibly subtyped as CRF103_01B strain. Near full‐length genome (NFLG) sequences were obtained to be aligned with former reported four CRF103_01B NFLG sequences, and neighbour‐joining (NJ) phylogenetic tree was constructed using MEGA11. SimPlot3.5 was used to analyse the putative parental strains and determine the breakpoints. Based on alignment from the homologous NFLG sequences with the parental strains, the longer fragments were selected to construct the fragment NJ trees, to infer the possible origins of the parental strains. The origination of the CRF103_01B was traced back by the Bayesian coalescent theory. Genotypic drug resistances were interpreted using the Stanford University HIV drug resistance database.

**Result**: NFLG sequences were amplified from five MSM individuals and a woman. Phylogenetic inference indicated that CRF103_01B genome was composed of six fragments separated by five breakpoints. The *gag*, *pol*, and *nef‐3'‐LTR* gene fragments in the CRF01_AE backbone were substituted by the counterparts from subtype B. Fragments IV and V of CRF103_01B clustered into large monophyletic clusters of subtype B (Accession No.: KU724105) and CRF01_AE cluster 5 strain (Accession No.: JX112804) from Beijing, with bootstrap values of 98% and 99%, respectively. CRF103_01B was estimated to originate around 2001.4 to 2006.7 in Beijing. Thirteen (92.9%, 13/14) patients of CRF103_01B carried V106I mutation of non‐nucleoside reverse transcriptase inhibitor.

**Conclusion**: The CRF103_01B strain was convinced to originate from Beijing and continued to spread among MSM population in northern China at a low level. Currently, this strain was spread to the general population via heterosexual contact. Molecular epidemiology and drug resistance surveillance should be reinforced, focusing on the transmission and disease progression.

#### P234 HIV subtypes: where are we now?

C Afonso, A Zagalo, A Ayres Pereira

Infectious Diseases, Hospital de Santa Maria, Centro Hospitalar Universitário Lisboa Norte, Lisbon, Portugal

**Background**: Published guidelines recommend HIV genotype resistance testing for all patients. Our aim was to characterise from an epidemiological point of view the different HIV subtypes present in our outpatient clinic.

**Material and methods**: Retrospective analyses of 821 patients admitted to our clinic until 2021, with available HIV subtype was made: age at diagnosis, year of diagnosis, country of origin, race, sex and form of transmission were obtained. Patients with vertical transmission were excluded.

**Results**: The main subtypes found were: B (38.8%); G (19.8%), CRF_02AG (15.8%) and C (10.7%). Until 2010 the main subtypes were B 41.5% and G 27.5%. After 2010 the main subtypes were B 35.9% and CRF_02AG 21% (G representing 11.9%). Comparative study of the main different subtypes showed epidemiological differences: ‐Subtype B (n = 319): 79.3% of patients were male, 75% of Portuguese origin, 52% were MSM and 34.4% had heterosexual sex as form of transmission, mean age at diagnosis was 33.6 years and 54% were diagnosed after year 2010. ‐Subtype G (n = 163): 49.6% of patients were male, 70.5% of Portuguese origin, 73.6% had heterosexual sex as form of transmission, mean age at diagnosis was 36.8 years and 30% were diagnosed infected after year 2010. ‐Subtype CRF_02AG (n = 130): 57.6% of patients were female, 26.9% of Portuguese origin, 70.7% of sub‐Saharan Africa origin, 82.3% had heterosexual sex as form of transmission, mean age at diagnosis was 37.3 years and 69.2% were diagnosed after year 2000. ‐Subtype C (n = 88): 55.6% of patients were female, 28.4% of Portuguese origin, 68.1% of sub‐Saharan Africa origin, 80.6% had heterosexual sex as form of transmission, mean age at diagnosis was 35.6 years and 61.3% were diagnosed after year 2010.

**Conclusions**: The analysis of this data shows a bimodal distribution of the epidemic in Portugal: an initial epidemic with B strains, as in Western Europe, and a second, latter one, with non‐B subtypes, disseminated through patients from African and South American origin. The presence on non‐B strains, with intrinsic patterns of resistance and underrepresented in clinical trials, puts a burden on the management of these patients.

#### P235 FluVac: influenza vaccination in PLWHIV in Germany during flu seasons 2018/19 and 2019/20 ‐ real‐world data extraction from electronic health record systems used by general practitioners and specialists

E Wolf ^1^, A Balogh^1^, C Roll^2^, S Noe^3^, N Postel^4^, H Heiken^5^, I Ruck^6^, W Schmidt^7^, U Kutscher^8^, U Meyer‐Bunsen^8^, S Preis^9^

^1^Clinical Research, MUC Research GmbH, Munich, Germany; ^2^Clinical Care, Praxis Schwabstraße 26, Stuttgart, Germany; ^3^Clinical Care, MVZ München am Goetheplatz, Munich, Germany; ^4^Clinical Care, Prinzmed, Munich, Germany; ^5^Clinical Care, Praxis Georgstrasse, Hannover, Germany; ^6^Clinical Care, Private Practice Dr. Ines Ruck, Leipzig, Germany; ^7^Clinical Care, MVZ Infektiologie Aerzteforum Seestrasse, Berlin, Germany; ^8^Medical Affairs, Janssen‐Cilag GmbH, Neuss, Germany; ^9^Clinical Research, Clinovate NET, Munich, Germany

**Background**: In Germany, there is no general vaccination registry collecting data on vaccine coverage of the populations at risk. Since the majority of vaccines are provided in the doctor's office, a major source of vaccination data are the medical record systems used by general practitioners and specialists. The aim of FluVac was to evaluate flu vaccination rates in adult PLWHIV in Germany.

**Table:** 

**Abstract P235 – Table 1**. Characteristics of the study population.		
	Flu season 2018/19	Flu season 2019/20
Total number of patients per flu season	6451	5289
Age [years]; median (IQR)	48 (38 to 55)	48 (39 to 56)
Age ≥60 years; n (%)	864 (13)	770 (15)
Female; n (%)	1238 (19)	926 (18)
Time since first HIV diagnosis [years]; median (IQR)	11 (6 to 19)	11 (5 to 19)
Absolute CD4 cell count (pre‐vaccination/Q4 [cells/μL]; median (IQR)	688 (500 to 910)	688 (505 to 893)
Absolute CD4 cell counts <200/μL (pre‐vaccination/Q4) [cells/μL]; n (%)	181 (3)	135 (3)

IQR, interquartile range.

**Methods**: Anonymised data extraction from electronic patient files of PLWHIV attending medical centres specialised in HIV care during flu seasons 2018/19 and/or 2019/20 – using a software solution (cvSentinel) with interface adaptions to the site‐specific electronic patient management systems. Variables of interest were vaccines (pre‐specified by available brand names), age, gender, ICD‐10 diagnoses with a focus on indication for influenza vaccination other than HIV diagnosis (e.g. asthma; COPD; diabetes mellitus; cardiovascular, liver or kidney diseases) as recommended by the STIKO (Standing Committee on Vaccination).

**Results**: In total, 11 740 anonymised patient records (from six centres) were extracted (flu season 2018/19: 6451; 2019/20: 5289; range across centres and seasons: 312 to 2246). Characteristics of the study populations are depicted in Table 1. In 2018/19 (2019/20) flu vaccination was documented in 33.5% (36.3%) of PLWHIV (range across centres in 2018/19: 27.1% to 57.7%; 2019/20: 30.9% to 60.4%). Vaccination rates with respect to age, gender or clinical indication are shown in Figure 1. Rates were slightly higher among men (vs women; difference (Δ) 7 to 9 percentage points), among PLWHIV aged 60+ (vs younger; Δ 7 percentage points) and among individuals with (potential) clinical indications (vs without; Δ 5 to 10 percentage points).

**Figure:**
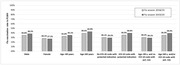
**Abstract P235 – Figure 1**. Flu vaccination rates in the FluVac cohort stratified by gender, age and clinical indication for flu vaccination other than HIV infection (based on ICD‐10 diagnoses corresponding to recommendations of the German Standing Committee on Vaccination).

**Conclusion**: In this project evaluating influenza vaccination in PLWHIV in Germany, the feasibility of using real‐world data (RWD) extracted from routine electronic patient records has been demonstrated. RWD use provided insights from centres specialised in HIV care in Germany: in the year prior to the SARS‐CoV‐2 epidemic, about one‐third of PLWHIV received flu vaccination. For PLWHIV at higher risk for severe disease, i.e. individuals aged 60+ or with (potential) clinical indications, vaccination rates were 5 to 10 percentage points higher. The development of interfaces to electronic health record systems widely used in Germany is essential for generating real‐world evidence.

### Late Presenters

#### P236 Estimating the risk of mortality attributable to late HIV diagnosis following admission to the intensive care unit: a single‐centre observational cohort study

N Bakewell ^1^, T Kanitkar^2^, O Dissanayake^3^, M Symonds^3^, S Rimmer^4^, A Adlakha^4^, M Lipman^5^, S Bhagani^3^, B Agarwal^4^, R Miller^6^, C Sabin^1^

^1^Institute for Global Health, University College London, London, UK; ^2^Intensive Care Unit and HIV Services, Royal Free Hospital, London, UK; ^3^HIV Services, Royal Free Hospital, London, UK; ^4^Intensive Care Unit, Royal Free Hospital, London, UK; ^5^HIV Services, Respiratory Medicine, and UCL Respiratory, Division of Medicine, Royal Free Hospital, London, UK; ^6^HIV Services and Centre for Clinical Research in Infection and Sexual Health, Institute for Global Health, Royal Free Hospital, London, UK

**Background**: Despite improvements in survival of people with HIV admitted to the intensive care unit (ICU), late diagnosis remains a major cause of in‐ICU mortality. We quantify the population attributable fraction (PAF) of in‐ICU mortality for late diagnosis among people with HIV admitted to a London ICU.

**Materials and methods**: Retrospective study (2000 to 2019) using data on index ICU admissions among people with HIV. Late diagnosis was a CD4+ T‐cell count <350 cells/mm^3^ and/or AIDS‐defining illness at/within 6 months prior to ICU admission. We compared patient characteristics (age, sex, Acute Physiology and Chronic Health Evaluation II, primary diagnosis, advanced HIV (CD4+ T‐cell count <200 cells/mm^3^ and/or AIDS at admission), undetectable viral load (VL; ≤50 copies/mL), receipt of combination antiretroviral therapy (cART)) and length of stay (LOS) by late diagnosis group using Wilcoxon‐rank‐sum/Chi‐squared/Fisher's exact tests. We used Poisson regression (robust standard errors) to estimate unadjusted/adjusted (age, sex, year of ICU admission) risk ratios (RRs), followed by regression standardisation to estimate PAFs.

**Results**: Two hundred and seven index admissions were included (median (interquartile range (IQR)) age: 46 (38 to 53) years; 72% male). Fifty‐eight (28%) were diagnosed late, of whom all had a CD4+ T‐cell count <350 cells/mm^3^ (vs 73% of those not diagnosed late; p < 0.001) and 95% had advanced HIV (vs 57%; p < 0.001). As expected, those diagnosed late were less likely to have an undetectable VL (9%, 58%; p < 0.001) and be receiving cART (33%, 88%; p < 0.001) (Table 1). Overall, the median (IQR) LOS was 5 (2 to 12) days, and 27% died in ICU. Those diagnosed late had a higher median LOS (6, 4 days; p = 0.02), and risk of in‐ICU mortality (unadjusted RR 1.71 (95% CI 1.10 to 2.68), with 16.64% (95% CI 15.60 to 17.69) of deaths being attributable to late diagnosis. Late diagnosis was independently associated with an increased risk of in‐ICU mortality (adjusted RR 1.75 (95% CI 1.05 to 2.91)), with 17.08% (95% CI 16.04 to 18.12) of deaths being attributable to this.

**Conclusions**: Improved public health efforts focused on HIV testing and serious incident reporting are needed to better understand potentially missed opportunities for earlier HIV diagnosis in all healthcare services. Such efforts would aid in reducing potentially preventable in‐ICU deaths.

**Table:** 

**Abstract P236 – Table 1**. Summary of patient characteristics at ICU admission.				
Characteristic, n (%) or median (interquartile range)	Overall n=207	Not diagnosed late n=149	Diagnosed late n=149	p‐value
Demographic factors				
Age (years)	46 (38‐53)	46 (40‐54)	44 (36‐52)	0.11
Male (sex at birth)	148 (71.5%)	110 (73.8%)	38 (65.5%)	0.23
Clinical factors				
Acute Physiology and Chronic Health Evaluation II score	19 (14‐25)	19 (13‐25)	21 (14‐26)	0.28
Advanced HIV^a^	138 (67.3%)	83 (56.5%)	55 (94.8%)	<0.001
Undetectable (viral load ≤50 copies/mL)	90 (45.0%)	85 (58.2%)	5 (9.3%)	<0.001
CD4+ T‐cell count <350 cells/mm^3^ at admission	164 (80.4%)	106 (72.6%)	58 (100.0%)	<0.001
Receipt of combination antiretroviral therapy (cART)^b^	147 (73.5%)	129 (88.4%)	18 (33.3%)	<0.001
Primary diagnosis at admission category				
Cardiovascular	7 (3.4%)	6 (4.0%)	1 (1.7%)	0.14
Gastrointestinal	23 (11.1%)	18 (12.1%)	5 (8.6%)	
Infection	27 (13.0%)	18 (12.1%)	9 (15.5%)	
Lower respiratory tract infection	65 (31.4%)	43 (28.9%)	22 (37.9%)	
Neurological	20 (9.7%)	12 (8.1%)	8 (13.8%)	
Oncological/Haematological	18 (8.7%)	11 (7.4%)	7 (12.1%)	
Renal	12 (5.8%)	10 (6.7%)	2 (3.4%)	
Other	35 (16.9%)	31 (20.8%)	4 (6.9%)	

^a^CD4+ T‐cell count <200 cells/mm and/or an AIDS‐defining illness at admission;

^b^patients were considered to be receiving cART if they had (1) data on cART start date pre‐dating ICU admission; or (2) a VL <1000 copies/mL at ICU admission but no cART date, as it was considered unlikely these patients exhibiting viraemic control were not on any form of cART.

#### P237 Improving the HIV testing cascade: implementing HIV teams to support HIV indicator condition‐guided testing in general practice in the Netherlands

C Jordans ^1^, D van Otterdijk^1^, L Rokx‐Niemantsverdriet^2^, J Struik^2^, P van der Voorn^3^, E van der Waal^3^, N Bakker^4^, C Rokx^1^

^1^Infectious Diseases, Erasmus Medical Centre, Rotterdam, Netherlands; ^2^General Practice, Gezondheidscentrum Mathenesserlaan, Rotterdam, Netherlands; ^3^General Practice, Huisartsenpraktijk Händellaan, Delft, Netherlands; ^4^General Practice, Huisartsen Rozenburcht, Capelle aan den IJssel, Netherlands

**Background**: In the Netherlands, many patients newly diagnosed with HIV in general practices are late presenters with multiple missed testing opportunities prior to HIV diagnosis. HIV indicator condition (IC)‐guided testing facilitates a timelier diagnosis of HIV. This pilot study aimed to evaluate the prevalence, diagnostic gaps and opportunities of HIV IC‐guided testing at general practitioners (GPs) in a region with a relatively high rate of undiagnosed HIV infections in the Netherlands.

**Materials and methods**: In an ongoing prospective observational study in 14 general practices on three locations in the Rotterdam region, we set up an HIV team consisting of HIV physicians and GP ambassadors from each location to evaluate HIV testing adequacy of 14 preselected common HIV ICs in general practices. In addition, GPs were informed at pilot start on the relevance of HIV IC‐guided testing and received free point‐of‐care HIV tests in their general practices. The main endpoint was the HIV testing rate adequacy of identified HIV ICs. Patient and GP experiences with the implemented strategy were assessed by questionnaires.

**Results**: A total of 377 HIV ICs, including 238 sexually transmitted infections (STIs), were identified on 54 248 screened GP appointments over a 1‐year period (prevalence: 0.7%, 95% CI 0.63% to 0.77%). Overall HIV testing rate of HIV ICs was 25.7%. Major deficiencies in HIV testing adequacy were observed in all 14 HIV ICs, with STIs having a 30.2% testing rate and unexplained weight loss having the highest testing rate (55.6%) (Table 1). Most common given reasons not to test for HIV when offered a test were: patient was unreachable (33%) and no‐show on follow‐up appointment (19%). Questionnaires performed amongst participating GPs and in a subset of patients who received point‐of‐care HIV testing showed, however, near universal positive attitudes towards HIV testing with GPs unanimously perceiving benefits for patient care and the implementation of a more proactive HIV testing strategy.

**Conclusions**: This pilot indicates that a significant gap exists between the positive attitudes of GPs and patients on HIV testing and the actual HIV IC‐guided testing adequacy. More pro‐active interventions will likely improve HIV testing rates with GPs in this low HIV prevalence setting.

**Table:** 

**Abstract P237 – Table 1**. HIV indicator condition HIV testing rates, grouped by sexually transmitted infections and non‐sexually transmitted infections.					
		Reported, n (%)	Test offered^a^, n (%)	Test performed^a^, n (%)	p‐value^b^
Total (n, %)		377 (100)	118 (31.3)	97 (25.7)	<0.001
Sexually transmitted infections		238 (63.1)	91 (38.2)	72 (30.2)	<0.001
Non‐sexually transmitted infections	(All other HIV indicator conditions)	139 (36.9)	27 (19.4)	25 (18.0)	<0.001
	Cervical dysplasia	4 (1.1)	0 (0.0)	0 (0.0)	
	Community‐acquired pneumonia	25 (6.6)	0 (0.0)	0 (0.0)	
	Herpes zoster	47 (12.5)	4 (8.5)	3 (6.4)	
	Mononucleosis‐like illness	2 (0.5)	1 (50.0)	1 (50.0)	
	Seborrhoeic dermatitis/exanthema	9 (2.4)	4 (44.4)	3 (33.3)	
	Severe or atypical psoriasis	4 (1.1)	1 (25.0)	1 (25.0)	
	Unexplained chronic diarrhoea	3 (0.8)	0 (0.0)	0 (0.0)	
	Unexplained fever	0 (0.0)	0 (0.0)	0 (0.0)	
	Unexplained leukocytopenia	4 (1.1)	1 (25.0)	1 (25.0)	
	Unexplained lymphadenopathy	8 (2.1)	3 (37.5)	3 (37.5)	
	Unexplained oral candidiasis	13 (3.4)	3 (23.1)	3 (23.1)	
	Unexplained thrombocytopenia	2 (0.5)	0 (0.0)	0 (0.0)	
	Unexplained weight loss	18 (4.8)	10 (55.6)	10 (55.6)	

^a^Percentages calculated based on reported total of HIV indicator conditions;

^b^calculation based on comparison between observed HIV test rate and the assumed baseline HIV test uptake of 50% (z‐test).

#### P238 Late diagnosis of HIV during the COVID‐19 pandemic in a LMIC: an emerging challenge

B Domecq, X Varrese, V Ortiz, R Zhumi Chacón, N D'Amico, M Bertolini, C Frola, J Barletta, M Jaume, A Sisto, M Rolón

Infectious Diseases, Hospital General de Agudos Dr. Juan A. Fernández, Buenos Aires, Argentina

**Background**: The impact of the disruption of HIV‐related services due to the COVID‐19 pandemic on the late diagnosis of HIV (LD) and AIDS‐defining diseases (DD) in low and low middle income countries (LMIC) is not well established. The aim of this study is to describe the prevalence of LD and DD in a cohort of recently diagnosed PLWHIV in a LMIC.

**Materials and methods**: This is a cross‐sectional study of a retrospective cohort of individuals aged ≥18 years, with a new HIV diagnosis between 2016 and 2021 in a referral centre. Two study periods were defined: P1 (pre‐pandemic: January 2016 to March 2020) and P2 (pandemic: April 2020 to December 2021). Sociodemographic and clinical features at diagnosis were evaluated. LD was defined as CD4 <200 cells/mm^3^ and/or diagnosis of a DD (WHO stage 4) within 3 months of HIV diagnosis.

**Results**: A total of 853 PLWHIV were diagnosed in the study period, 35.5% (303) were LD, 35.7% (254) in P1 and 40.2% (49) in P2 (p = 0.2). Among subjects with LD 72% (218) were cis‐men, 21% (64) cis‐women and 7% (21) trans‐women. Median age was 36 years (IQR 16) in P1 and 38 years (IQR 22) in P2 (p = 1.7). Median CD4 cell count was 84 cells/mm^3^ (IQR 117) in P1 versus 69 cells/mm^3^ (IQR 120) in P2 (p = 0.3). Prevalence of at least one DD was 16.9% (124) in P1, versus 24.6% (30) in P2 (p = 0.04). Burden of co‐existing DD (≥2 DD at diagnosis) was higher in P2 (2.5% in P1 vs 9% in P2, p < 0.01). Prevalence of extrapulmonary tuberculosis and disseminated histoplasmosis was higher in P2 versus P1 (8 (16.3%) vs 16 (6.3%) (p = 0.01) and 6 (12.2%) vs 11 (4.3%) (p = 0.02) respectively).

**Conclusions**: During the COVID‐19 pandemic, there was an increase in the prevalence of DD and in the proportion of subjects with ≥2 DD at diagnosis, particularly extrapulmonary tuberculosis and disseminated histoplasmosis. Additional efforts are needed to address the challenge of LD in LMIC.

#### P239 Missed opportunities for an early HIV diagnosis in Greece (MORFEAS): preliminary results

S Roussos ^1^, K Protopapas^2^, A Antoniadou^2^, A Papadopoulos^2^, G Lourida^3^, V Papastamopoulos^3^, M Chini^4^, K Alexakis^5^, E Barbounakis^5^, D Kofteridis^5^, L Leonidou^6^, M Marangos^6^, V Petrakis^7^, P Panagopoulos^7^, E Mastrogianni^8^, P Palla^9^, N Sipsas^9^, V Paparizos^10^, S Metallidis^11^, I Katsarolis^12^, V Sypsa^1^, M Psichogiou^8^

^1^Hygiene, Epidemiology and Medical Statistics, National and Kapodistrian University of Athens, Medical School, Athens, Greece; ^2^4th Department of Internal Medicine and HIV/ID Unit, ATTIKON University General Hospital, Athens, Greece; ^3^5th Department of Internal Medicine and HIV/ID Unit, Evangelismos General Hospital, Athens, Greece; ^4^3rd Department of Internal Medicine and HIV/ID Unit, 'Red Cross' Korgialeneio‐Benakeio General Hospital, Athens, Greece; ^5^Department of Internal Medicine and HIV/ID Unit, Herakleion University General Hospital (PAGNH), Herakleion Crete, Greece; ^6^Department of Medicine and HIV/ID Unit, Patras University General Hospital, Patras, Greece; ^7^Department of Internal Medicine and HIV/ID Unit, Alexandroupolis University General Hospital, Alexandroupolis, Greece; ^8^1st Department of Internal Medicine, Laiko General Hospital, Athens, Greece; ^9^Pathophysiology Department, Laiko General Hospital, Athens, Greece; ^10^1st Department of Dermatology and Venereology, HIV Unit, Andreas Syggros University Hospital, Athens, Greece; ^11^1st Internal Medicine Department, Infectious Diseases Division, AHEPA Hospital, Thessaloniki, Greece; ^12^Medical Affairs, Gilead Sciences Hellas and Cyprus, Athens, Greece

**Background**: People living with HIV (PLWHIV) presenting late in care may have been evaluated for symptoms associated with HIV, but the opportunity for earlier diagnosis may have been lost. COVID‐19 pandemic caused significant disruptions limiting access to testing. The goals of this preliminary analysis are to determine the frequency and describe features of health care contacts (HCC) prior to an HIV diagnosis and quantify missed opportunities before and within the COVID‐19 waves.

**Materials and methods**: This is a retrospective, non‐interventional study in 10 HIV clinics in Greece. Demographic data and details of HCC were collected from medical files of adult PLWHIV diagnosed from 1/1/2019 till 31/12/2021 with an available CD4 count within 6 months from diagnosis. A missed opportunity (MO) was defined as a HCC due to a clinical manifestation or laboratory abnormality of an indicator condition that did not lead to HIV testing within the 5 years before diagnosis. We estimated the proportion of PLWHIV with at least one MO. Late presentation (LP) was defined as diagnosis with a CD4 count <350/mm^3^ or an AIDS‐defining condition regardless of CD4 count.

**Results**: A total of 557 new HIV cases (85.3% males, 76.1% of Greek nationality, 54.9% MSM, 24.6% heterosexuals, 12.4% PWID) were included in this preliminary analysis (31% of the national total of 1804 cases diagnosed during 2019 to 2021). Mean age (SD) was 39.4 (11.7) years. In total 42% reported previous HIV testing. Acute HIV infection represented 13% of the cases. Ninety‐five individuals (17.1%) had already developed AIDS (most frequently reported indicative diseases being *Pneumocystis jirovecii* pneumonia 35.8%, wasting syndrome 25.3% and tuberculosis 13.7%). Median (25th, 75th) CD4 count at diagnosis was 330 (144, 511). HIV diagnosis was established concurrently with hospitalisation in 42%. A MO was identified in 39.8% of new cases for year 2019, 33.3% for 2020 and 45.1% for 2022 (p = 0.07), without any difference among transmission groups. The most prominent indicator condition was sexually transmitted infections.

**Conclusions**: MO for earlier testing/diagnosis of HIV infection and LP are key challenges in combatting the HIV epidemic. It remains important to increase awareness and uptake of pre‐emptive testing and educate on indication‐based testing.

#### P240 Impact of the COVID‐19 pandemic on the diagnosis of late presenters of HIV‐infected patients at a tertiary hospital in Lisbon, Portugal in 2018 to 2021

S Casanova^1^, J Borralho ^1^, C Fernandes^2^, A Miranda^1^, J Vasconcelos^1^, J Alves^1^, S Peres^1^, T Baptista^1^, I Antunes^1^, F Borges^1^, J Nina^1^, K Mansinho^1^

^1^Infectious Diseases and Tropical Medicine Department, Hospital Egas Moniz ‐ Centro Hospitalar de Lisboa Ocidental, Lisbon, Portugal; ^2^Internal Medicine Department, Centro Hospitalar de Leiria, Leiria, Portugal

**Background**: A European survey reported a reduction of HIV testing up to 50% during the first months of the COVID‐19 pandemic in 2020, and the Centers for Disease Control and Prevention reported 50% of late presenters (LP) by the end of 2020. The latest Portuguese annual HIV report, that evaluates new HIV infection cases, during 2019, reported 49.7% patients diagnosed as LP.

**Materials and methods**: Retrospective observational cohort study conducted in a Portuguese tertiary hospital, in central Lisbon, that analysed late presenter patients diagnosed with HIV infection between 2018 and 2021.

**Results**: From 2018 to 2021, 468 patients were referenced to our centre following recent HIV infection diagnosis. One hundred and eighty‐seven (39.9%) were diagnosed as LP, including 95 (20.3%) with advanced disease (TCD4+ <200 cells/mm^3^). LP were mainly men (70.6%), and older than non‐LP patients (mean age of 44 [21 to 79] for LP vs 37.6 [21 to 78] for non‐LP). Patients were mostly Portuguese (LP 38.0%; non‐LP 42.3%) and Brazilian (LP 21.4%; non‐LP 29.3%). Sexual transmission was the principal route of HIV acquisition. Heterosexual behaviour was more prevalent in the LP group and homosexual in the non‐LP group (LP 43.9% vs non‐LP 25.3% heterosexual and LP 39.0% vs non‐LP 60.9% homosexual). In the LP group 23.0% presented with an AIDS‐defining disease. Tuberculosis (28.6%), oesophageal candidosis (18.4%), and pneumocystosis (16.3%) were the main opportunistic conditions found. When comparing the pre‐ (2018 to 2019) and pandemic period (2020 to 2021), we found 13% less HIV diagnosis in the pandemic period but 1.0% more LP. Understanding the need to maintain active screening and rapid referral to health care after HIV diagnosis, our outpatient clinic did not interrupt activity during the pandemic period and continued receiving new patients.

**Conclusions**: Our cohort evidenced a lower percentage of late presentation of HIV diagnosis, facing the previous published national data. The group of non‐LP were mainly MSM (60.9%), which can be partially explained by a greater search for health care by this group of patients. In the pandemic period, our cohort showed a reduction (13%) in the number of new HIV referenced patients but without a significant increase in late.

#### P241 Persisting and emerging challenges of late HIV presentation in Germany

C Boesecke^1^, S Schellberg ^2^, J Schneider^3^, G Schuettfort^4^, H Stocker^5^

^1^Department of Medicine I, Bonn University Hospital, Bonn, Germany; ^2^Novopraxis Berlin GbR, Berlin, Germany; ^3^Technical University of Munich, University Hospital rechts der Isar, Munich, Germany; ^4^Department of Infectious Diseases, University Hospital Frankfurt, Frankfurt, Germany; ^5^St Joseph's Hospital, Berlin, Germany

**Background**: Approximately 30% of HIV diagnoses in Germany continue to be late diagnoses. We aimed to identify factors preventing further progress in this area.

**Materials and methods**: A panel of five German physicians with established research expertise in HIV was assembled to discuss contemporary challenges associated with late HIV presentation in Germany. Outputs from these discussions were analysed to identify key themes, broadly categorised as persisting challenges of late HIV presentation in Germany, and emerging factors with acute implications for HIV care.

**Results**: Late HIV diagnosis, particularly among heterosexual, older men in rural areas, remains a persisting challenge in Germany. Ongoing development of scoring systems to identify patients in primary care who should be tested for HIV (i.e. provider‐initiated indicator testing) can help to address this issue. However, limitations of this approach, such as constraints on primary care physicians’ capacity to apply such tools in the face of more prevalent/higher‐priority diseases, and the lack of any previous healthcare consultation before late HIV (or AIDS) diagnosis in many individuals, require that universal HIV testing strategies are also applied. Provider‐initiated universal testing strategies (e.g. emergency room HIV testing, universal testing in high‐prevalence areas) have been well implemented in countries such as the UK, which has reduced undiagnosed HIV by a further ∼6% versus Germany, and is the first European country to achieve the 90‐90‐90 goal. Unfortunately, acceptance of universal testing initiatives still needs to be built in Germany. In addition, HIV testing is not reimbursed in Germany. These persisting barriers to improved HIV diagnosis reflect the somewhat conservative German political landscape, and perceptions that undiagnosed HIV is not a relevant problem. This is particularly concerning in light of emerging healthcare stressors, most notably increased numbers of undiagnosed HIV cases likely to result from the influx of refugees from the Ukraine war, and hitherto unknown downstream impacts of the COVID‐19 pandemic on the burden of late HIV presentation.

**Conclusion**: Provider‐initiated HIV testing strategies are lacking in Germany. These are essential to achieving further reductions in late HIV diagnosis rates, and to future resilience of the German healthcare system to unforeseen challenges.

### COVID‐19: In the Context of HIV

#### P242 Humoral immunogenicity to third dose SARS‐COV‐2 mRNA vaccine in people living with HIV (PLWHIV) by current CD4 count and CD4/CD8 ratio

A Vergori ^1^, A Cozzi‐Lepri^2^, A Tavelli^3^, M Giannella^4^, S Cicalini^1^, L Marconi^5^, V Yellenki^3^, S Meschi^6^, G Pellicanò^5^, N Caroccia^4^, G Matusali^6^, A Latini^7^, M Lichtner^8^, S Lo Caputo^9^, F Fusco^10^, G Marchetti^3^, E Tacconelli^4^, A Antinori^1^, A D'Arminio Monforte^3^

^1^HIV/AIDS Unit, National Institute for Infectious Diseases L.Spallanzani, Istituti di Ricovero e Cura a Carattere Scientifico (IRCCS), Rome, Italy; ^2^CREME, Institute for Global Health, UCL, London, UK; ^3^Infectious Diseases Unit, Azienda Socio Sanitaria Territoriale Santi Paolo e Carlo, University of Milan, Milan, Italy; ^4^Department of Medical and Surgical Sciences, Alma Mater Studiorum University of Bologna, Bologna, Italy; ^5^Department of Human Pathology, University of Messina, Messina, Italy; ^6^Laboratory of Virology, National Institute for Infectious Diseases L.Spallanzani, Istituti di Ricovero e Cura a Carattere Scientifico (IRCCS), Rome, Italy; ^7^Infectious Diseases Unit, San Gallicano Institute Istituti di Ricovero e Cura a Carattere Scientifico (IRCCS), Rome, Italy; ^8^Infectious Diseases Unit, Santa M. Goretti Hospital, Sapienza University, Latina, Italy; ^9^Department of Clinical and Experimental Medicine, University of Foggia, Foggia, Italy; ^10^Infectious Diseases Unit, Azienda Ospedaliera di Rilievo Nazionale Ospedali Delli Colli, PO D Cotugno, Napoli, Italy

**Background**: Aim was to investigate humoral response elicited after the third dose of SARS‐CoV‐2 mRNA vaccination, according to CD4 count and CD4/CD8 ratio, in a large cohort of PLWHIV.

**Materials and methods**: PLWHIV of the VAXICONA‐ORCHESTRA cohort who received a complete SARS‐CoV‐2 mRNA vaccine cycle and for whom anti‐S serology was available were included. Participants stratified by CD4 count pre‐vaccination (LCD4 = CD4 count <200 cells/mm^3^; ICD4 = CD4 count 201 to 500 cells/mm^3^; HCD4 = CD4 count >500 cells/mm^3^) and by CD4/CD8 ratio (low ratio [LR]: 0.0 to 0.59; intermediate ratio [IR]: 0.60 to 0.99; high ratio [HR]: 1.0). Immune response defined as: anti‐S ≥7.1 BAU/mL (Abbott), ≥0.82 BAU/mL (Roche) and ≥4.8 BAU/mL (DiaSorin); low response: ≤46 BAU/mL (any assay). ANOVA was used to compare titres (log2 scale); association between CD4, CD4/CD8 ratio groups and risk of undetectable/low level anti‐S was evaluated by means of ANOVA and logistic regression.

**Results**: Six hundred and twenty‐five PLWHIV: LCD4 = 96, ICD4 = 199, HCD4 = 330 [56 years (48 to 61), CD4 nadir 124 cells/mm^3^ (39 to 299), 89% HIV‐RNA <50 copies/mL]. After a median of 164 days (151 to 193) from the second dose the proportion of undetectable/low response was 15.6/72.7% for LCD4, 5.6/33.9% for ICD4 and 8.8/27.5% for HCD4 (p < 0.0001/p < 0.0001). After a median of 29 days (16 to 56) after the third dose, the proportion of PLWHIV with undetectable/low immune response showed a sharp decline to 5.21/12.5% for LCD4, 0.5/2.0% for ICD4 and 0.3/1.2% for HCD4 (p < 0.0001/p < 0.0001). Among those who had available CD4/CD8 ratio at T0 [n = 596; LR = 250; IR = 183; HR = 163; 56 years (47 to 61), CD4 nadir 125 cells/mm^3^ (40 to 298), 89.5% HIV‐RNA <50 copies/mL], the proportion of PLWHIV with undetectable/low immune response were 0.6/1.2% for LR, 0/1.6% for IR, 2.4/6% for HR. aOR from fitting a logistic regression for all vaccine doses responses according with CD4 count and CD4/CD8 ratio are reported in Table 1.

**Table:** 

**Abstract P242 – Table 1**. OR of non‐response after third dose according to CD4 count (Panel A) and to CD4/CD8 ratio (Panel B) at the time of vaccination from fitting a logistic regression analysis.					
		Logistic regression of the probability of anti‐S response post full 3 doses vaccination cycle			
	Unadjusted		Adjusted^a^		
Panel A	odds ratio (95% CI)	p‐value	odds ratio (95% CI)	p‐value	Type III p‐value^b^
CD4 count					
		Undetectable ‐ after 3rd dose			
HCD4	1		1		0.451
ICD4	1.83 (0.11 to 29.41)	0.671	1.53 (0.09 to 25.78)	0.767	
LCD4	18.51 (2.13 to 160.8)	0.008	4.41 (0.33 to 59.31)	0.264	
Low level (below 46 BAU/mL) ‐ after 3rd dose					
HCD4	1		1		0.005
ICD4	0.99 (0.48 to 2.06)	0.985	0.95 (0.45 to 1.99)	0.893	
LCD4	4.40 (2.29 to 8.46)	<0.001	3.43 (1.58 to 7.44)	0.002	
**Panel B**					
Undetectable ‐ 3rd dose					
HR	1		1		0.292
IR	−		−		
LR	3.98 (0.48 to 33.40)	0.203	1.14 (0.10 to 13.25)	0.914	
Low level (below 46 BAU/mL) ‐ after 3rd dose					
HR	1		1		0.082
IR	1.34 (0.22 to 8.13)	0.749	1.30 (0.21 to 7.94)	0.777	
LR	5.14 (1.16 to 22.78)	0.031	3.77 (0.81 to 17.47)	0.090	

HCD4, CD4 >500/mm^3^; HR, ≥1; ICD4, CD4 201‐500/mm^3^; IR, CD4/CD8 ratio 0.60‐0.99; LCD4, CD4 <200/mm^3^; LR, CD4/CD8 ratio 0‐0.59.

^a^Adjusted for age, CD4 nadir, VL 50 copies/mL at T0 and no. of comorbidities;

^b^from the adjusted model.

**Conclusions**: In this large cohort, the third dose elicited a strong humoral immune response and quickly over‐compensated the waning of immune after the second dose. CD4 count but no CD4/CD8 ratio was confirmed as a strong predictor of humoral response to SARS‐CoV‐2 vaccination in PLWHIV. These data are essential for targeted strategies for appropriate delivery of a fourth dose in PLWHIV.

#### P243 Evolution of anti‐SARS‐CoV‐2 spike protein titers after two‐dose COVID‐19 vaccination among people living with HIV

W Liu ^1^, S Chang^2^, J Wang^1^, H Sun^1^, Y Huang^1^, K Lin^1^, U Wu^3^, G Li^1^, W Liu^1^, Y Su^2^, P He^1^, C Lin^4^, C Yeh^4^, Y Chen^5^, Y Luo^5^, P Wu^5^, L Chen^5^, H Chang^5^, W Sheng^1^, S Hsieh^1^, C Hung^1^, S Chang^1^

^1^Department of Internal Medicine, National Taiwan University Hospital, Taipei City, Taiwan; ^2^Department of Clinical Laboratory Sciences and Medical Biotechnology, National Taiwan University Hospital, Taipei City, Taiwan; ^3^Department of Medicine, National Taiwan University Cancer Center, Taipei City, Taiwan; ^4^Department of Nursing, National Taiwan University Hospital, Taipei City, Taiwan; ^5^Center of Infection Control, National Taiwan University Hospital, Taipei City, Taiwan

**Background**: While SARS‐CoV‐2 vaccines have demonstrated promising results in general population, real‐world information on the immunogenicity remains limited among people living with HIV (PLWHIV).

**Materials and methods**: PLWHIV receiving the first dose of SARS‐CoV‐2 vaccine between January 2021 and December 2021 were enrolled. Determinations of anti‐SARS‐CoV‐2 spike IgG titers were performed every 1 to 3 months, until the participants received the third dose of SARS‐CoV‐2 vaccine or the diagnosis of COVID‐19. All serum samples were tested for anti‐nucleocapsid antibody and those tested positive were excluded from analysis.

**Results**: One thousand, three hundred and four participants were enrolled: 829 (63.6%) receiving two doses of AZD1222 vaccine, 232 (17.8%) mRNA‐1273 vaccine, 128 (9.8%) BNT162b2 vaccine, and 64 (4.9%) MVC‐COV1901 vaccine. There were 51 (3.9%) participants who underwent heterologous vaccination with AZD1222 vaccine followed by mRNA‐1273 or BNT162b2 vaccine. Of all time periods, participants receiving two doses of mRNA vaccine had consistently higher antibody levels than those receiving AZD1222 or MVC‐COV1901 vaccine (p < 0.001 for all time‐period comparisons). Of participants receiving two doses of AZD1222 vaccine, factors associated with failure to reach an anti‐spike IgG titer >141 Binding Antibody Units (BAU)/mL within 12 weeks included diabetes mellitus (adjusted hazard ratio [aHR] 2.63; 95% CI 1.34 to 5.18), chronic kidney disease (aHR 2.55; 95% CI 1.09 to 5.97) and a CD4 count <200 cells/mm^3^ upon the first dose of vaccination (aHR 4.16; 95% CI 1.14 to 15.19). For those receiving two doses of mRNA vaccine, factors associated with failure to reach an anti‐spike IgG titer >141 BAU/mL within 12 weeks were CD4 count <200 cells/mm^3^ on first‐dose vaccination (aHR 5.22; 95% CI 1.33 to 20.47) and an interval of more than 4 months between two doses of vaccination (aHR 2.44; 95% CI 1.03 to 5.81). Of all participants, factors associated with a rapid decline of anti‐spike IgG titer to <141 BAU/mL 12 to 24 weeks after second‐dose vaccination included chronic HBV infection (aHR 0.045; 95% CI 1.02 to 5.56) and two doses of AZD1222 vaccine (aHR 9.9; 95% CI 5.34 to 18.37).

**Conclusions**: Two doses of homologous mRNA vaccination had significantly higher immunogenicity than vaccination with AZD1222 or MVC‐COV1901 among PLWHIV. PLWHIV with CD4 count <200 cells/mm^3^ had consistently lower antibody responses with either mRNA or non‐mRNA vaccination.

#### P244 COVID‐19 in HIV‐infected patients: does tenofovir‐based ART have any impact?

F Rombini^1^, D Cecchini ^1^, D Pinto^1^, L Calanni^2^, R Cuini^3^, E Obieta^4^, M Greco^5^, F Morales^6^, C Migazzi^7^, L Morganti^8^, Y El Kozah^1^, P Parenti^9^, I Cassetti^1^, for the COVIDARE study team

^1^Medical Area, Helios Salud, Buenos Aires, Argentina; ^2^Infectious Diseases Unit, Unidad Infectológica de Prevención, Diagnóstico y Tratamiento Neuquén ‐ CEIN, Neuquen, Argentina; ^3^Infectious Diseases Unit, Hospital T. Alvarez, Buenos Aires, Argentina; ^4^Infectious Diseases Unit, Hospital Municipal Ciudad de Boulogne, Boulogne, Argentina; ^5^Infectious Diseases Unit, Hospital Español de La Plata, La Plata, Argentina; ^6^IPTEI‐Investigación Prevención y Tratamiento de las Enfermedades Infecciosas, Buenos Aires, Argentina; ^7^Infectious Diseases Unit, Hospital Presidente Perón, Avellaneda, Argentina; ^8^Infectious Disease Unit, Hospital Cosme Argerich, Buenos Aires, Argentina; ^9^Infectious Disease Unit, Centro de Asistencia e Investigación Clínica Integral ‐ CAICI, Rosario, Argentina

**Background**: The nucleotide analogue tenofovir has been hypothesised to be effective in COVID‐19, still being a controversial issue. Tenofovir is available as two prodrugs, tenofovir disoproxil fumarate (TDF) and tenofovir alafenamide (TAF), both currently backbone of antiretroviral therapy (ART) regimens. People living with human immunodeficiency virus (PLWHIV) might be at increased risk for severe COVID‐19 progression.  We aim to describe clinical outcomes of COVID‐19 in PLWHIV with and without tenofovir‐based ART in a national cohort.

**Table:** 

**Abstract P244 – Table 1**. Demographic profile, clinical characteristics and outcomes of COVID‐19 in HIV‐infected patients with tenofovir and non‐tenofovir based baseline ART. Values are numbers (percentages) unless otherwise stated.			
	Tenofovir (TDF/TAF) cohort (n = 929)	Non‐tenofovir cohort (n = 228)	p‐value
Male sex	612 (65.8%)	145 (63%)	0.091
Age, years (median, IQR)	43 (36 to 51)	50 (40 to 57)	0.011
Comorbidities	352 (37.8%)	115 (59.4%)	0.001
Virological suppression	758 (81.5%)	179 (78.5%)	1.000
CD4 T‐cell count cel/uL (median, IQR)	598 (434 to 800)	656.5 (472.5 to 824)	0.824
Symptomatic COVID‐19 (n, %)	891 (95.9%)	221 (96.9%)	0.509
Radiographic abnormalities (n/N, %)	85/197 (45.1%)	24/59 (40.6%)	1.000
Hospitalisation	146 (15.7%)	41 (17.9%)	0.119
Oxygen therapy	83 (8.94%)	31 (13.5%)	0.040
COVID‐19 therapy	105 (11.3%)	30 (13.1%)	0.618
Mortality	8 (0.86%)	5 (2.1%)	1.000

**Material and methods**: Prospective observational multicentric study in Argentina (COVIDARE). PLWHIV with confirmed COVID‐19 were enrolled from September 2020 to mid‐June 2022 with a standardised method. Patients were stratified according baseline ART regimen into two groups: those with tenofovir (either TDF or TAF) and those without any of them. Univariate and multivariate analysis were performed to evaluate impact of tenofovir versus non‐tenofovir containing regimens on major clinical outcomes adjusting by potential confounders.

**Results**: One thousand, one hundred and fifty‐seven patients were included, 929 (80%) received tenofovir‐based ART (79% TDF, 21% TAF). Accompanying drugs included almost universally XTC and, as third drug, mainly BIC (77%) for TAF regimens and mainly DRV/r (39.6%), EFV (32%) or DTG (14.4%) for those with TDF. Non‐tenofovir ART was predominantly based on ABC/XTC (63.5%) or single 3TC (19%) as nucleoside analogues + DRV/R (34.6%), DTG (32%), or EFV (20%), among others. A comparison between both cohorts is shown in Table 1. Considering demographics, both groups were similar except for age, older in non‐tenofovir group. Regarding prevalence of symptomatic disease, radiological findings, hospitalisation and mortality no differences were observed between cohorts. Oxygen therapy requirement was higher in the non‐tenofovir group. Age and non‐tenofovir ART remain associated to requirement of oxygen therapy on multivariate analysis (p = 0.025 and 0.031, respectively).

**Conclusion**: No differences were observed in hospitalisation and mortality rates in tenofovir versus non‐tenofovir groups. Conversely, requirement of oxygen therapy was associated to older age and non‐tenofovir ART which may suggest a protective effect of tenofovir. Considering the intrinsic limitations of the observational design of this study, clinical trials are needed to better define impact of tenofovir‐containing ART in clinical outcomes of COVID‐19.

#### P245 Antibody response and HIV‐1 RNA response following a booster of SARS‐CoV‐2 vaccination in people living with HIV

C Fedeli ^1^, P Ustero^1^, V Portillo^1^, E Mereles^1^, I Savkarelidze^1^, M Puntel^1^, I Petignat^1^, C Jaksic^2^, S Yerly^3^, A Calmy^1^

^1^Service of Infectious Diseases, Geneva University Hospital (HUG), Geneva, Switzerland; ^2^CRC & Division of Clinical Epidemiology, Department of Health and Community Medicine, Geneva University Hospital (HUG), Geneva, Switzerland; ^3^Division of Laboratory Medicine; Centre for Emerging Viral Diseases and Laboratory of Virology, Geneva University Hospital (HUG), Geneva, Switzerland

**Background**: People living with HIV (PLWH) are at higher risk of a more severe clinical course of SARS‐CoV‐2 disease [1,2], but most individuals have shown a good serological response after two vaccine doses, with limited adverse events reported [3]. We aimed to assess the impact of a third dose ('booster dose') on the antibody response and HIV‐1 RNA levels among this high‐risk population.

**Materials and methods**: Single‐centre, open‐label, observational study where PLWH were offered vaccination with two doses of mRNA1273 or BNT162b2 1 month apart, plus a booster dose administered between 6 to 10 months after the first dose. Anti‐receptor‐binding domain (RBD) and anti‐nucleoprotein antibodies were measured together with HIV‐1 RNA levels: before the booster, at the first dose (M0) and then at month 1 (M1), 2 (M2), and 6 (M6) after the first dose; thereafter at 1 (M1b) and 6 (M6b) months after the booster dose. CD4 counts were measured at baseline, M6 and 12 months after the first dose (M12). Anti‐RBD was compared with a group of 49 healthy volunteers.

**Results**: One hundred and thirty‐one individuals (median age: 54 years [IQR 47.0 to 60.5]; male: 70.2%; median baseline CD4 T‐cell: 602 cells/μL [IQR 445.0 to 825.5]; median nadir CD4 T‐cells: 223 cells/μL [IQR 111.0 to 330.0]) were included. Median age of healthy volunteers was 30 years (IQR 27.0 to 34.5; male: 61.2%). The anti‐RBD response was lower for PLWH at M1, M2 and M6 compared with the control group, but this difference lost its statistical significance 1 and 6 months after the booster dose (M1b to M6b) (Figure 1). No statistically significant differences in antibody levels were observed between individuals with HIV‐1 RNA below or >20 copies/mL. Nineteen of 128 (14.8%) had detectable HIV‐1 RNA (>20 copies/mL) at M0, 13/128 (10.2%) at M1, 15/124 (12.1%) at M2, 12/107 (11.2%) at M6, and 8/92 (8.7%) and 4/47 (8.5%) at M1/M6 post‐booster, respectively. No serious adverse effects were reported.

**Conclusions**: All participants elicited a good anti‐RBD response after three doses of mRNA vaccine. No impact was observed on HIV‐1 RNA levels or CD4 T‐cell count over 6 months after each dose.

**Figure:**
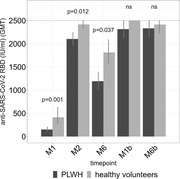
**Abstract P245 – Figure 1**. Quantification of anti‐RBD Ig (GMT) in PLWH (dark grey bars) and healthy volunteers (light grey bars) at each time point. p‐value significant if <0.05. GMT, geometric mean titers; M1, 30 days after the first vaccine dose; M2, 60 days after the first vaccine dose; M6, 6 months after the first vaccine dose; M1b, 30 days after the booster dose; M6b, 60 days after the booster dose; RBD, receptor binding domain.

**References**

1. Mirzaei H, McFarland W, Karamouzian M, Sharifi H. COVID‐19 among people living with HIV: a systematic review. AIDS Behav. 2021;25:85‐92.

2. Boffito M, Waters L. More evidence for worse COVID‐19 outcomes in people with HIV. Lancet HIV. 2021;8:e661‐2.

3. Calmy A, Bel M, Nguyen A, Combescure C, Delhumeau C, Meier S, et al. Strong serological responses and HIV RNA increase following AS03‐adjuvanted pandemic immunization in HIV‐infected patients. HIV Med. 2012;13:207‐18.

#### P246 Lower T‐cell response against SARS‐CoV‐2 variants of concern after mRNA vaccine and risk of breakthrough infections in people living with HIV

P Vizcarra^1^, S Martín‐Colmenarejo^1^, J del Pino^1^, M Pérez‐Elías^1^, A Moreno ^1^, S Gomez‐Maldonado^1^, A Martín‐Hondarza^2^, A Abad^1^, A Vallejo^2^, J Casado^1^

^1^Infectious Diseases, Ramón y Cajal University Hospital, Madrid, Spain; ^2^Laboratory of Immunovirology, Ramón y Cajal University Hospital, Madrid, Spain

**Background**: SARS‐CoV‐2 variant of concern (VOC) B.1.1.529 (Omicron) presents a surprisingly large number of mutations in its spike protein escaping from antibody neutralisation. Thus, it is important to determine how well T‐cell response performs against different variants including Omicron in people living with HIV (PLWHIV) following COVID‐19 vaccination, and the impact on new infections during follow‐up.

**Materials and methods**: Prospective cohort study of PLWHIV who underwent blood tests for humoral and cellular response after two‐doses vaccination schedule with a mRNA vaccine against SARS‐CoV‐2. Humoral (anti‐S IgG, CLIA, Abbott; binding antibody units (BAU)/mL) and IFN‐γ producing T‐cell responses to spike peptides of the ancestral virus, and Delta and Omicron variants were performed.

**Results**: Overall, 142 PLWHIV were included. Median age was 53 years (range, 24 to 78), 83% were male, and 41% had at least one comorbidity. Nadir CD4+ count was 273/mmc, and 26% had a previous AIDS diagnosis, but current CD4+ was 659/mmc and HIV RNA level was ≤50 copies/mL in 99% of cases. A median time of 53 days after the second vaccine dose, humoral response was observed in 96% of cases (median BAU/mL 834, IQR 169 to 1871). Humoral and T‐cell response to original SARS‐CoV‐2 were highly correlated (rho = 0.657; p < 0.01). Also, there was a high correlation between T‐cell response to the original strain, Delta, and Omicron variants. However, the magnitude of CD4+ and CD8+ T‐cell responses were significantly lower to Delta in proportion (83% and 82% against Delta variant, 72% and 74% against Omicron variant, respectively), and in magnitude (3% and ‐20% for Delta, ‐33% and ‐28% for Omicron variant). A total of 29 (17%) breakthrough infections were observed during a median follow‐up of 351 days, associated with a lower level of specific antibodies (890.8 vs 1559.7 BAUs; p = 0.027), and with a lower magnitude of CD4+ and CD8+ T‐cell response to the different variants (statistically significant for CD8+ T‐cell response).

**Conclusions**: T‐cell responses against Delta and Omicron spike peptides, although preserved in nearly two‐thirds of PLWHIV, were significantly lower than to the original strain after two doses of mRNA vaccine. Importantly, this lower response was associated with breakthrough infections during follow‐up.

#### P247 Impact of COVID‐19 pandemic on HIV health services in Russia

N Ladnaia, V Pokrovsky, E Sokolova, O Yurin

AIDS Prevention and Control Research Department, Central Research Institute of Epidemiology, Moscow, Russian Federation

**Background**: Assess the impact of the COVID‐19 pandemic on HIV health care coverage rates in Russia.

**Methods**: Analysis of data of Federal statistic surveillance form containing the findings of HIV antibody tests, data the Federal Rospotrebnadzor forms for monitoring HIV response, and personalised data on newly detected cases of HIV infection among Russian citizens in 2019 to 2021.

**Results**: Main data (Table 1) show that due to aggressive COVID‐19 response measures, such as lockdown in 2020 and different types of self‐isolation, 12.7% less people were tested for HIV. In 2021 in Russia, the testing coverage rate returned to the 2019 levels, largely to testing for HIV antibodies in people seeking care in connection with COVID‐19. This may be the main reason of a sharp (24.1% to 26.9%) decline in newly detected HIV cases between 2019 and 2020 to 2021. The total number of people receiving ART increased 23.5% by the end of 2021 compared to 2019. However, the number of PLWHIV first included in care and ART decreased by more than a quarter in 2021 and a total treatment coverage does not increase so significantly due to the substantial number of new HIV cases and the high proportion of PLWHIV not linked to care.

**Conclusions**: The COVID‐19 pandemic has had a negative impact on HIV health care coverage rates in Russia and lead to increase in the number of deaths among PLWHIV which may be also associated with COVID‐19 coinfection.

**Table:** 

**Abstract P247 – Table 1**. Indicators of HIV health care coverage rates in Russia.			
Indicators	2019	2020	2021
Number of HIV tests among Russian citizens	40 580 588	35 409 873	41 927 340
New HIV cases among Russian citizens	97 108	73 676	71 019
PLWHIV linked to care	776 868	788 938	803 796
PLWHIV linked to care in the reporting year	81 058	63 177	62 451
PLWHIV first taken on ART in the reporting year	116 510	106 106	85 828
PLWHIV receiving ART during the reporting year	534 990	604 999	660 821
The number of deaths of HIV‐infected Russian citizens in the reporting year	33 577	32 208	34 093

#### P248 T‐cell and humoral responses to mRNA‐1273 vaccine up to 6 months in late presenter (LP) people living with HIV (PLWH)

M Augello, V Bono, R Rovito, V Yellenki, C Tincati, A d'Arminio Monforte, G Marchetti

Department of Health Sciences, University of Milan, Milan, Italy

**Background**: Immune responses to SARS‐CoV‐2 mRNA vaccines in PLWH with a history of late presentation and their durability have not been fully characterised. We hereby longitudinally evaluated T‐cell and humoral responses to mRNA‐1273 vaccination up to 6 months in LP‐PLWH.

**Materials and methods**: SARS‐CoV‐2‐specific T‐cell and humoral responses were comparatively assessed in LP‐PLWH (CD4 nadir <350/μL and/or previous AIDS diagnosis) on effective cART, who received mRNA‐1273, and in HIV‐negative healthcare workers (HCWs), who received BNT162b2. We determined antigen‐specific activation induced markers (CD69+CD137+)‐expressing CM, EM, EMRA, cTfh/cTfc (CCR7/CD45RA, CXCR5) and cytokines (IFN‐γ, TNF‐α, IL‐2, IL‐4, IL‐17A)‐producing CD4 and CD8 T‐cells after wild‐type SARS‐CoV‐2 spike (S) peptides challenge (flow cytometry) as well as anti‐RDB antibodies (ELISA) and neutralisation activity (surrogate neutralisation assay) before vaccination (T0), 1 month (T1) and 5 months (T2) after second dose. Wilcoxon and Mann‐Whitney tests were used for statistical analyses.

**Results**: Twenty LP‐PLWH (median CD4 nadir = 67/μL; current median CD4 = 404/μL, CD4/CD8 = 0.57, HIV‐RNA <20 copies/mL) and 20 HCWs were included (Table 1). In each group, 10 had a clinical history of previous COVID‐19 (experienced) and 10 had not (naïve). LP‐PLWH showed at T1 and T2 significant increase of: (i) S‐specific total/CM/EM/EMRA and cTfh CD4 T‐cells (Figure 1A); (ii) polyfunctional Th1‐cytokine (IFN‐γ, TNF‐α, IL‐2)‐ and Th2‐cytokine (IL‐4)‐producing S‐specific CD4 T‐cells (Figure 1B); (iii) anti‐RBD antibodies and neutralisation activity (Figure 1C). Of note, immune responses to vaccine in LP‐PLWH were not inferior to HCWs. Interestingly, while SARS‐CoV‐2‐experienced HCWs displayed higher humoral and Th1‐cell responses at both T0 and T1 versus naïve, SARS‐CoV‐2‐experienced LP‐PLWH showed only higher anti‐RBD antibodies versus naïve at T0.

**Table:** 

**Abstract P248 – Table 1**. Demographic and clinical characteristics of the study population.			
	LP‐PLWH (n = 20)	HCWs (n = 20)	p‐value LP‐PLWH vs HCWs^a^
Age, years, median (IQR)	57 (47 to 62)	55 (45 to 61)	0.3500
Sex, n (%)			0.1908
Male	15 (75)	10 (50)	
Female	5 (25)	10 (50)	
Ethnicity, n (%)			0.1264
Caucasian	15 (75)	20 (100)	
Latin‐American	3 (15)	0 (0)	
Afro‐American	1 (5)	0 (0)	
African	1 (5)	0 (0)	
Comorbidities, n (%)			
Hypertension	5 (25)	2 (10)	0.4075
Chronic heart disease	2 (10)	0 (0)	0.4872
Myocardial infarction	1 (5)	0 (0)	>0.9999
Peripheral vascular disease	1 (5)	0 (0)	>0.9999
Chronic pulmonary disease	2 (10)	2 (10)	>0.9999
Chronic kidney disease	2 (10)	1 (5)	>0.9999
Liver disease	3 (15)	0 (0)	0.2308
Diabetes	3 (15)	0 (0)	0.2308
Charlson comorbidity index,^b^ median (IQR)	2 (0 to 4)	1 (0 to 2)	0.1153
Epidemiology, n (%)			
MSM	8 (40)		
IDU	2 (10)		
Other	10 (50)		
Viro‐immunological parameters, median (IQR)			
CD4 nadir, cells/μL	67 (32 to 215)		
HIV‐RNA zenith, copies/mL	60 816 (22 402 to 242 511)		
Current % CD4	25 (12 to 30)		
Current CD4, cells/μL	404 (192 to 615)		
Current % CD8	46 (35 to 60)		
Current CD8, cells/μL	809 (590 to 1008)		
Current CD4/CD8 ratio	0.57 (0.20 to 0.87)		
Current HIV‐RNA, copies/mL	<20		
Previous AIDS diagnosis, n (%)	8 (40)		
Current cART regimen, n (%)			
INSTI‐based triple	11 (55)		
INSTI‐based dual	6 (30)		
NNRTI‐based triple	3 (15)		
Duration of cART, years, median (IQR)	12 (6 to 17)		

cART, combination antiretroviral therapy; HCWs, healthcare workers; IDU, injective drugs use; INSTI, integrase strand transfer inhibitor; IQR, interquartile range; LP‐PLWH, late presenter people living with HIV.

^a^Statistical analyses: Mann‐Whitney U test, Fisher exact test, Chi‐square test, as appropriate;

^b^age‐adjusted.

**Figure:**
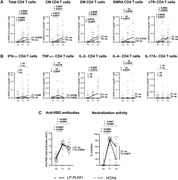
**Abstract P248 – Figure 1**. T‐cell and humoral responses to vaccine in LP‐PLWH and HCWs.

**Conclusions**: In PLWH with pre‐cART advanced immunodeficiency and full virological control on cART, a two‐dose mRNA‐1273 vaccine cycle is able to induce S‐specific memory polyfunctional Th1, Th2 and cTfh cells as well as anti‐RBD neutralising antibodies, not inferior to HIV‐uninfected peers, still detectable after 6 months. Interestingly, in our cohort of LP‐PLWH, natural SARS‐CoV‐2 infection, while able to sustain S‐specific antibody response, seems less efficacious in inducing a T‐cell memory and in boostering immune responses to vaccine, possibly reflecting an enduring partial immunodeficiency.

#### P249 An investigation into the psychological impact of the COVID‐19 pandemic for people living with HIV

S Lo ^1^, L Muschialli^1^, T Fernandez^2^, D Peppa^3^, F Burns^4^

^1^Division of Infection and Immunity, University College London, London, UK; ^2^Division of Infection and Immunity, Royal Free London NHS Foundation Trust, London, UK; ^3^Division of Infection and Immunity, Royal Free London NHS Foundation Trust, Mortimer Market Centre, Department of HIV, CNWL NHS Trust/University College London, London, UK; ^4^Division of Infection and Immunity, Royal Free London NHS Foundation Trust/Institute for Global Health, University College London, London, UK

**Background**: People living with HIV (PLWHIV) frequently report high levels of anxiety and depression. This study aimed to assess the prevalence and associations of dysfunctional COVID‐19 anxiety in PLWHIV.

**Materials and methods**: Participants were recruited from two UK sites (01/03/2020 to 30/05/2022) to an immunological study. Socio‐demographic, HIV‐related information, and pandemic concerns were collected. Participants could complete the Coronavirus Anxiety Scale (CAS), that is a uni‐dimensional scale measuring COVID‐19‐related dysfunctional anxiety level [1]. Scoring ≥9 out of 20 signifies dysfunctional pandemic‐related anxiety. Mean total CAS score and range were derived, using the Mann‐Whitney U or Kruskal‐Wallis tests to assess significance. CAS also collected data on pandemic concerns, analysed using simple linear regression.

**Results**: One hundred and fifteen PLWHIV were recruited and 114 completed the CAS (Table 1). The median age was 51 (22 to 93 years); 83% identified as male (n = 95) and 66% of participants as white (n = 66). The mean total CAS score was 1.18, with 4.4% scoring ≥9 (n = 5). A larger proportion of females (16.7%, n = 3) scored ≥9 on CAS than males (2.1%, n = 2). Black African and ‘Other’ ethnic minority PLWHIV (13.6%, n = 3; 25%, n = 2) had a greater proportion of CAS scores ≥9 than White or Asian PLWHIV (both 0%). People who reported more pandemic concerns scored greater on CAS (β = 0.33; p < 0.001). Top concerns included ‘increased anxiety’ and ‘practical aspects of life’. The mean total CAS score and proportion scoring ≥9 was greater in people who had COVID (p = 0.021; 6.8% vs 0%). A CAS ≥9 was not associated with a lower CD4 (<350 cells/mm^3^), a detectable HIV viral load (≥50 copies/mL), the presence of co‐morbidities nor a history of pre‐pandemic anxiety and depression.

**Conclusions**: In this study, the majority of CAS scores were low, suggesting PLWHIV did not experience heightened COVID‐related anxiety. Significant COVID anxiety was not associated with HIV parameters. Heightened anxiety was most prevalent in females, Black/Ethnic minorities, and people who have had COVID. In‐depth qualitative research is required to understand the drivers of disabling COVID anxiety in PLWHIV.

**Table:** 

**Abstract P249 – Table 1**. Mean total CAS scores of PLWHIV categorised into socio‐demographic and HIV‐related variables.		
PLWHIV	Mean total CAS score [min. score, max. score] (no. of participants ≥9, % of participants ≥9)	p‐value (comparing mean total CAS scores)
Overall (n = 114)	1.18 [0, 15] (5, 4.4%)	
Age in years		p = 0.524
20 to 29 (n = 2)	0.50 [0, 1] (0, 0%)	
30 to 39 (n = 13)	0.23 [0, 1] (0, 0%)	
40 to 49 (n = 26)	2.38 [0, 15] (3, 11.5%)	
50 to 59 (n = 43)	1.19 [0, 12] (2, 4.7%)	
60 to 69 (n = 10)	0.90 [0, 5] (0, 0%)	
70+ (n = 6)	0.17 [0, 1] (0, 0%)	
Gender		p = 0.116
Male (n = 95)	0.84 [0, 12] (2, 2.1%)	
Female (n = 18)	2.83 [0, 15] (3, 16.7%)	
Other (n = 1)	3.00 [0, 3] (0, 0%)	
Ethnicity		p = 0.079
White (n = 66)	0.61 [0, 3] (0, 0%)	
Asian (n = 4)	0.00 [0, 0] (0, 0%)	
Black African (n = 22)	2.77 [0, 15] (3, 13.6%)	
Other ethnic minorities (n = 8)	3.50 [0, 12] (2, 25%)	
Household		p = 0.503
Living alone (n = 36)	1.38 [0, 12] (2, 5.4%)	
Not living alone (n = 77)	1.08 [0, 15] (3, 3.9%)	
Education		p = 0.973
Post‐secondary education (e.g. university, vocational training) (n = 95)	1.53 [0, 15] (4, 4.2%)	
No post‐secondary education (n = 17)	1.12 [0, 13] (1, 5.9%)	
Enough money to cover basic necessities		p = 0.266
Always/mostly (n = 103)	1.04 [0, 15] (3, 2.9%)	
No/sometimes (n = 9)	3.00 [0, 13] (2, 22.2%)	
Pandemic concerns	N/A (Simple linear regression analysis, β = 0.332) ‐ for every increase in 1 pandemic concern, CAS score increases by 0.332	p < 0.001
Access to health and support services (n = 30)		
Increased anxiety (n = 48)		
Becoming mentally unwell (n = 23)		
Family and relationships (n = 37)		
Isolation (n = 35)		
Negative feelings (n = 43)		
Practical aspects of life (n = 47)		
Concern about yourself or your family contracting the virus (n = 42)		
No concerns (n = 17)		
History of pre‐pandemic anxiety/depression		p = 0.208
Yes (n = 38)	1.87 [0, 15] (3, 7.9%)	
No (n = 65)	0.95 [0, 13] (2, 3.1%)	
Past COVID infection (e.g. confirmed PCR (polymerase chain reaction) + or antibody test)		p = 0.021
Yes (n = 74)	1.59 [0, 15] (5, 6.8%)	
No (n = 30)	0.40 [0, 5] (0, 0%)	
Long COVID symptoms ‐ only those with past COVID infection		p = 0.085
Yes (n = 37)	2.27 [0, 15] (4, 10.8%)	
No (n = 37)	0.92 [0, 10] (1, 2.7%)	
HIV‐related variables:		
Most recent HIV viral load, mean in RNA/mL		p = 0.817
<50 (n = 109)	1.18 [0, 15] (5, 4.6%)	
>50 (n = 5)	1.00 [0, 5] (0, 0%)	
Most recent CD4+ T cell count, mean in cells/mm^3^		p = 0.106
CD4+ T cell count <350 (n = 11)	0.09 [0, 1] (0, 0%)	
CD4+ T cell count ≥350 (n = 75)	1.05 [0, 13] (3, 4.0%)	
Comorbidities (e.g. hypertension, diabetes, asthma, obesity etc.)		p = 0.804
One or more conditions (n = 62)	1.26 [0, 15] (3, 4.8%)	
None (n = 40)	1.18 [0, 13] (2, 5.0%)	

**Reference**

1. Lee SA, Coronavirus Anxiety Scale: a brief mental health screener for COVID‐19 related anxiety. Death Stud. 2020;44:393‐401.

#### P250 Immunogenicity of homologous versus heterologous prime‐boost vaccination regimens against SARS‐COV‐2 in people living with HIV in Argentina: a pilot study

R Mauas ^1^, D Cecchini^1^, A Urueña^1^, M Strada^2^, S Arietti^2^, I Cassetti^1^

^1^Infectious Diseases, Helios Salud, Ciudad Autónoma de Buenos Aires, Argentina; ^2^Immunoserology, Laboratorio Dr. Stamboulian, Ciudad Autónoma de Buenos Aires, Argentina

**Background**: Vaccination campaign against SARS‐CoV‐2 began in Argentina in late December 2020. It included vaccines from different platforms: mainly viral vector (Sputnik V, AstraZeneca) and inactivated virus (Sinopharm) followed by Messenger ribonucleic acid (mRNA) (Moderna, Pfizer), which were used as homologous or heterologous prime‐boost schemes. People living with HIV (PLWHIV) are vulnerable to COVID‐19 complications and their immunisation should be warranted. However, data are lacking regarding humoral response to those regimens in this population. We aim to assess such outcome in PLWHIV.

**Materials and methods**: Prospective study in PLWHIV assisted in an HIV ambulatory care centre in Argentina (August to December 2021), and who received two doses of SARS‐CoV‐2 vaccination. Detection of S1‐RBD IgG (immunoglobulin type G) antibodies was performed between days 28 and 60 after the second dose using the ADVIA Centaur^®^ SARS‐CoV‐2 IgG chemiluminescent immunoassay (reference value: positive ≥1 U/mL). Demographic, immunovirological status, history of COVID‐19, seroconversion, and median antibody (Ab) titers were assessed and compared according to the regimen received (homologous vs heterologous prime‐boost).

**Results**: One hundred individuals completed the study. Median age: 48 years (39 to 54); male: 68%. Plasma HIV‐RNA <20 copies/mL: 96%; median CD4+ T‐cell count: 620 (459 to 800) cells/mm^3^; history of COVID‐19: 10%; median time between second dose and Ab measurement: 37 days. Overall, 97% of the individuals seroconverted and median Ab titers were 40 U/mL (11 to 150). Seventy‐nine subjects received homologous vaccination regimens: 36 Sputnik V, 36 AstraZeneca and seven Sinopharm; 21 received heterologous regimens: 19 Sputnik V‐Moderna, one AstraZeneca‐Moderna, one Sputnik V‐AstraZeneca. There were no differences in all variables assessed between groups except for the age (older subjects in the heterologous group). Median Ab titers were significantly higher with heterologous prime‐boosted regimens: 150 U/mL (78 to 150) than with homologous ones: 30.7 U/mL (9 to 100), (p = 0.005) (Table 1).

**Conclusions**: Most PLWHIV developed an adequate immune humoral response regardless of the regimen received. Our pilot study indicates that heterologous prime‐boosted regimens, mostly including Moderna mRNA vaccine, provide stronger antibody response in PLWHIV. These findings agree with those observed in general population and supports the recommendation of heterologous vaccination use.

**Table:** 

**Abstract P250 – Table 1**. Clinical‐demographic comparison according to the regimen received (homologous vs heterologous prime‐boost).			
Variable	Homologous (n = 79)	Heterologous (n = 21)	p
Median age, years (IQR)	47 (37 to 53)	54 (51 to 59)	0.019
Sex, n (%)			
Female	25 (32)	7 (33)	0.155
Male	54 (68)	14 (67)	
Median CD4+ T‐cell count, cells/mm^3^ (IQR)	620 (447 to 791)	620 (571 to 815)	0.171
Plasma HIV‐RNA <20 copies/mL, n (%)	75 (95)	21 (100)	0.176
History of COVID‐19, n (%)			
No	70 (89)	20 (95)	0.907
Yes	9 (11)	1 (5)	
Median days between second dose and Ab measurement (IQR)	37 (32 to 50)	40 (36 to 50)	0.381
Ab titers ≥1 U/mL (POSITIVE), n (%)	76 (96)	21 (100)	0.590
Median Ab titers, U/mL (IQR)	30.7 (9 to 100)	150 (78 to 150)	0.005

#### P251 Immunogenicity of AZD1222 (ChAdOx1) SARS‐CoV‐2 vaccine in people living with HIV

J Begovac ^1^, S Zekan^1^, N Cetinic Balent^2^, I Javoric^3^, N Bogdanic^3^, R Mikulic^2^, O Dakovic Rode^2^

^1^Department of Infectious Diseases, School of Medicine, University of Zagreb, Zagreb, Croatia; ^2^Virology Department, University Hospital for Infectious Diseases, Zagreb, Croatia; ^3^HIV Department, University Hospital for Infectious Diseases, Zagreb, Croatia

**Background**: People living with HIV (PLWHIV) are at increased risk for severe COVID‐19 [1]. We aimed to evaluate the serological response after two doses of AZD1222 (ChAdOx1) SARS‐CoV‐2 vaccination in PLWHIV.

**Figure:**
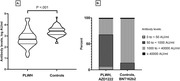
**Abstract P251 – Figure 1**. Comparison of antibody levels (log transformed) 3 months after the second dose of AZD1222 (ChAdOx1) SARS‐CoV‐2 vaccine in people living with HIV (PLWHIV) and second dose of BNT162b2 vaccine in HIV‐uninfected controls (A). Horizontal lines are the median, Q1 and Q3 values. Panel B: The distribution of antibody levels 3 months after the second dose of AZD1222 or BNT162b2 vaccine in 59 PLWHIV and 59 controls. The SARS‐CoV‐2 IgG II Quant Architect Abbot assay was used.

**Materials and methods**: Participants were evaluated before the first dose (baseline) and 3 months after the second dose of the ChAdOx1 vaccine. Patient's sera were analysed using the SARS‐CoV‐2 IgG II Quant Architect Abbott assay [2]. An antibody concentration ≥50 arbitrary units per milliliter (AU/mL) was defined as positive (seroconversion) [2]. We also compared the immunogenicity of AZD1222 in PLWHIV to sex‐matched healthcare workers (controls) who received two doses of the BNT162b2 vaccine. The study measurements were done from April to December 2021.

**Results**: Fifty‐nine PLWHIV were included: 57 men, median age 45.0 years, all receiving antiretroviral treatment and with controlled viral loads (58 with HIV RNA <50 copies/mL), 57 had >200 CD4+ cells/μL, and the median CD4 cell count was 722 (IQR 520 to 878) cells/μL (Table 1). Eight (13.6%) PLWHIV were obese (≥30.0 kg/m^2^), and 31 (52.5%) were overweight (excluding obese). At baseline 56 PLWHIV had an antibody concentration <50 AU/mL. Fifty‐five (93.2%) patients seroconverted after vaccination. An antibody concentration between 50 and 1000 AU/mL had 36 (61.0%) PLWHIV, 18 (30.5%) had between 1000 and 40 000 AU/mL and only one had above the upper threshold of quantification (Figure 1). No severe adverse events were reported. Of 59 healthcare workers, median age 45.5 years, 50 (84.8%) had an antibody concentration between 1000 and 40 000 AU/mL 3 months after the second dose of the BNT162b2 vaccine. The median antibody concentration in AZD1222 recipients was 505.4 (IQR 196.3 to 1685.8) AU/mL whereas in BNT162b2 recipients it was 2740.9 (IQR 1328.5 to 4847.4) AU/mL (Figure 1). No participant had COVID‐19.

**Conclusions**: Vaccination with two doses of AZD1222 in PLWHIV under effective antiretroviral treatment and a high CD4 cell count led to a moderately successful antibody response which was lower than in healthcare workers receiving the BNT162b2 vaccine.

**Table:** 

**Abstract P251 – Table 1**. Baseline characteristics of 59 PLWHIV and anti‐SARS‐CoV‐2 IgG antibodies levels, 3 months after two doses of AZD1222 (ChAdOx1). Values are frequencies or median with percentages or first to third quartiles in parenthesis.				
	Antibody levels after 3 months			
	<1000 AU/mL (N = 40)	≥1000 AU/mL (N = 19)	Total (N = 59)	p‐value
Male gender	38 (95.0)	19 (100.0)	57 (96.6)	>0.99
Age, years	43.5 (38.7 to 52.2)	46.2 (40.3 to 51.2)	45.0 (39.0 to 51.6)	0.73
Age >50 years	14 (35.0)	5 (26.3)	19 (32.2)	0.50
Transmission				>0.99
Heterosexual	2 (5.0)	1 (5.3)	3 (5.1)	
MSM	38 (95.0)	18 (94.7)	56 (94.9)	
Duration of HIV infection, years	6.1 (3.2 to 8.9)	7.9 (4.3 to 11.9)	6.1 (3.5 to 10.6)	0.36
Had clinical AIDS	8 (20.0)	1 (5.3)	9 (15.3)	0.14
CD4 cell count, per mm^3^	760.0 (570.5 to 873.0)	697.0 (452.0 to 962.0)	722.0 (520.0 to 878.0)	0.46
CD4 cell count, >800 per mm^3^	16 (40.0)	7 (36.8)	23 (39.0)	0.82
Nadir CD4 cell count, per mm^3^	290.0 (109.0 to 398.5)	292.0 (139.0 to 424.0)	292.0 (130.0 to 408.0)	0.82
CD4/CD8 ratio	0.9 (0.7 to 1.2)	0.9 (0.7 to 1.1)	0.9 (0.7 to 1.2)	0.83
BMI, kg/m^2^	26.0 (23.2 to 27.6)	27.0 (25.4 to 28.4)	26.2 (23.5 to 27.7)	0.15
BMI categories, kg/m^2^				0.52
<18.5	1 (2.5)	0 (0.0)	1 (1.7)	
18.5 to 24.9	15 (37.5)	4 (21.1)	19 (32.2)	
25.0 to 29.9	19 (47.5)	12 (63.2)	31 (52.5)	
≥30	5 (12.5)	3 (15.8)	8 (13.6)	
Antiretroviral therapy				0.29
1NRTI+INSTI	12 (30.0)	5 (26.3)	17 (28.8)	
2NRTI+INSTI	13 (32.5)	10 (52.6)	23 (39.0)	
2NRTI+NRTI	15 (37.5)	4 (21.1)	19 (32.2)	

BMI, body mass index; INSTI, integrase inhibitors; NRTI, nucleoside reverse transcriptase inhibitors; NNRTI, non‐nucloside reverse transcriptase inhibitors.

**References**

1. Dong Y, Li Z, Ding S, Liu S, Tang Z, Jia L, et al. HIV infection and risk of COVID‐19 mortality: a meta‐analysis. Medicine (Baltimore). 2021;100:e26573.

2. US Food and Drug Administration. AdviseDx SARS‐CoV‐2 IgG II [Internet]. [cited 2022 Aug 23]. Available from: https://www.fda.gov/media/146371/download.

#### P252 Does COVID‐19 vaccination have a striking protective effect in HIV infected people? A real‐life experience of a Portuguese centre

C Fernandes ^1^, A Duarte^2^, J Alves^2^, S Peres^2^, A Miranda^2^, T Baptista^2^, I Antunes^2^, F Borges^2^, J Nina^2^, K Mansinho^2^

^1^Internal Medicine, Leiria Hospital Centre, Leiria, Portugal; ^2^Infectious Diseases and Tropical Medicine, Egas Moniz Hospital, West Lisbon Hospital Centre, Lisbon, Portugal

**Background**: In the beginning of SARS‐COV‐2 pandemic, HIV infection was pointed out as a possible factor of a more severe COVID‐19 course. The aim of this study was to evaluate the severity of COVID‐19 in patients with HIV chronic infection and correlate it with vaccination status.

**Materials and methods**: We conducted a retrospective, observational study that included a total of 3904 patients. This number represents HIV infected patients that had at least one medical appointment from March 2020 to March 2022 in a Portuguese central hospital. From these, 877 subjects were identified as having had a COVID‐19 diagnosis along this period, of whom 105 were randomly selected for COVID‐19 severity characterisation and vaccination status, through phone call clinical interviews and medical records consultation.

**Results**: Male sex was predominant (72.4%). Median age was 49 (minimum 22, maximum 82). At the time of the COVID‐19 diagnosis the median TCD4 count was 710 cells/μL with six subjects (5.7%) presenting with TCD4 <200 cells/μL. 88.6% of the patients had undetectable viral load (<20 copies/mL) and 100% with <500 copies/mL. Only three patients were not on antiretroviral therapy (2.9%). Most patients did not have completed the anti‐SARS‐COV‐2 vaccination regimen such as recommended by the time they contracted COVID‐19 (73.3%). Severity of COVID‐19: mild, moderate, severe and critical corresponded respectively to 92.4% (97), 3.8% (four) and 3.8% (four). Seven patients (6.7%) needed hospitalisation. To evaluate the correlation between vaccination status (completed vs incompleted) and severity of disease we used a chi‐square test and found out that these two variables were independent with a p > 0.05.

**Conclusions**: The vast majority of our HIV patients contracted COVID‐19 before having completed the recommended anti‐SARS‐COV‐2 vaccination plan, even though the most had a mild course of the disease. No statistically significant correlation was found between vaccination status and COVID‐19 severity. Selection and ascertainment bias related with the global good immunological status and virological control of our HIV cohort; the low numbers of severe and critical COVID‐19 cases and the possible partial protection effect of an incomplete vaccination plan might have influenced our study results.

#### P253 The impact of COVID‐19 pandemic in HIV diagnosis: the experience of a Central Hospital in Lisbon, Portugal

C Fernandes ^1^, S Casanova^2^, J Borralho^2^, A Miranda^2^, J Alves^2^, S Peres^2^, T Baptista^2^, I Antunes^2^, F Borges^2^, J Nina^2^, K Mansinho^2^

^1^Internal Medicine, Leiria Hospital Centre, Leiria, Portugal; ^2^Infectious Diseases and Tropical Medicine, Egas Moniz Hospital, West Lisbon Hospital Centre, Lisbon, Portugal

**Background**: Since March 2020 the priority of public health was to manage the COVID‐19 pandemic. This event impaired our ability to assist patients with other diseases and HIV infection was not an exception. The aim of this study was to evaluate the number and characteristics of new HIV diagnosis during COVID‐19 pandemic in our hospital and to compare it with pre‐COVID‐19 era.

**Materials and methods**: We conducted a descriptive and retrospective study that included the patients with new HIV diagnosis referred to our outpatient care unit from 1 January 2016 to 31 December 2021. Clinical, laboratory and demographic data were collected from medical records.

**Results**: A total of 732 patients were included in this study. We divided the patients into three bienniums: new diagnosis made in 2016 to 2017, 2018 to 2019 and 2020 to 2021 revealing 264, 253 and 215 new cases, respectively. It corresponds to a reduction of 4.17% in new diagnosis between the first and second period (pre‐COVID‐19 era) and a reduction of 15.02% between the second and last period (COVID‐19 era). We verified an increase of referral of new HIV diagnosis from community‐based screening programmes (24.24%, 31.62% and 45.58%, respectively). Male sex was predominant in all the groups. New diagnosis were more common in men who have sex with men (46.59%, 60.21% and 72.25%, respectively). The three most common nationalities were the same in all groups: Portugal, Brazil and Guinea‐Bissau. Late presenters: 33.33% in the first group; 35.57% in the second group and 42.32% in the last group. At the time of diagnosis 47 subjects (17.80%) in the first group, 51 (20.16%) in the second group and 55 (25.58%) in the last group presented with AIDS‐defining disease.

**Conclusions**: We verified that there was a decline in new HIV diagnosis even before the COVID‐19 era, even though this decline aggravated after the beginning of this pandemic. During the pandemic, we were able to maintain our usual outpatient HIV clinic activity and receive patients referred with new HIV diagnosis, so other external factors contributed to this decline.

#### P254 Humoral immune response of patients living with HIV (PLWHIV) after three doses of mRNA BNT162b2 SARS‐CoV‐2 vaccine: a prospective cohort study

L Tau ^1^, T Halperin^2^, A Adler^2^, R Marom^2^, S Ahsanov^2^, N Matus^2^, I Levy^2^, G Gerber^3^, S Lev^3^, T Ziv‐Baran^3^, D Turner^1^

^1^Crusaid Kobler AIDS Center, Tel‐Aviv Sourasky Medical Center, Tel‐Aviv, Israel; ^2^Microbiological Laboratory, Tel‐Aviv Sourasky Medical Center, Tel‐Aviv, Israel; ^3^Tel‐Aviv University, Sackler Faculty of Medicine, Tel‐Aviv, Israel

**Background**: Recent studies had shown a robust humoral and cellular immune response following vaccination with two doses of mRNA SARS‐CoV2 vaccines among PLWHIV. However, data is missing regarding the vaccine response after three doses.

**Materials and methods**: We followed prospectively a group of PLWHIV in Crusaid Kobler AIDS Center, Tel‐Aviv, that received three doses of mRNA BNT162b2 vaccine and for whom serological data 4 to 6 months  after two vaccine doses was available from our previous study. Patients provided an additional blood sample in a timeframe of 4 to 6 months after the third vaccine dose. The aim of the study was to evaluate the difference of serological response after second and third doses and to investigate the factors that could influence the vaccine response. Humoral response was evaluated by measuring IgG titers of anti spike receptor‐binding domain antibodies (anti‐RBD IgG). The level of anti‐nucleocapsid IgG (anti N‐IgG) was measured as well.

**Results**: Of 136 patients for whom serological data after two vaccine doses was available from previous study, 50 had provided a serum sample for serological evaluation after booster dose. Five patients were excluded due to positive anti‐N‐IgG, suggesting previous SARS‐CoV‐2 infection. Mean age was 47 and a majority were male (82%). Median time between second and third vaccine doses was 6.87 months and a median time between the third vaccine dose and serological evaluation was 5.7 months. The levels of anti‐RBD IgG were higher after third vaccine dose with a median delta of 3240 arbitrary units/mL. Median CD4 count was 660 (515 to 958) cells/mm^3^ and had no influence on the antibody level. Factors that were associated with lower delta were lower anti‐RBD IgG level after second vaccine (p = 0.016), higher CD8 count (p = 0.024), longer time between the vaccine doses (p = 0.008) and longer time after the third vaccine dose (p = 0.005).

**Conclusions**: The level of anti‐RBD IgG after three doses of mRNA BNT162b2 vaccine was higher when compared to the level after two doses, suggesting additional value of the booster vaccine dose.

#### P255 The vaccinations against COVID‐19 efficacy and safety among people living with HIV: data from observational study in Poland

A Skrzat‐Klapaczynska, C Bienkowski, J Kowalska

Department of Adults' Infectious Diseases, Medical University of Warsaw, Warsaw, Poland

**Background**: People living with HIV (PLWHIV) are a heterogeneous group of immunocompromised persons. Detectable HIV viral load and chronic comorbidities are independently increasing the risk of severe outcomes from COVID‐19 among PLWHIV. We aimed to assess the efficacy and safety of vaccinations against COVID‐19 in PLWHIV.

**Materials and methods**: We performed a retrospective analysis of data collected from medical records of HIV‐positive individuals between 1 January 2021 and 30 April 2022 that are under care of HIV Out‐patient Clinic in Warsaw. Analysis included data on type and date of administration of subsequent doses of COVID‐19 vaccination, adverse vaccine reactions, and the history of SARS‐CoV‐2 infection. In 21 patients anti‐SRBD antibodies (Magnumi) were determined 14 days after the second dose of the vaccine.

**Results**: In total 218 patients were included in the analysis with a median age of 43 years [IQR 35.5 to 51.5 years] and median CD4+ count 591 cells/uL [IQR 459.5 to 745.0 cells/uL]. Most of patients were male (191/218, 87.6%) and were vaccinated with Comirnaty vaccine (143/218, 65.5%). In 21/218 (9.6%) patients the data on S‐RBD antibody titers was available. A significant increase in S‐RBD antibody titers >100 AU/mL was observed, compared the titers measured 1 week after the 1st dose to titers performed after the 2nd vaccine dose (regardless of vaccination type) (3/21, 14.3% vs 17/21, 81.0%; p < 0.0001). After 12 months of follow‐up four (4/21, 19%) patients had a breakthrough SARS‐CoV‐2 infection confirmed either by RT‐PCR or antigen test. Two patients after the third dose of vaccination. None of the patients diagnosed with COVID‐19 required hospitalisation. Vaccine adverse events (VAE) occurred more often after the 1st vaccine dose compared to VAE after 2nd and 3rd dose (33.3% vs 14.3% vs 4.8%, p = 0.0458)

**Conclusions**: Vaccination against COVID‐19 is safe and effective against severe course of the disease among PLWHIV. However, after vaccination it is still possible to acquire SARS‐CoV‐2 infection. Therefore, longer observations are required in order to measure the time length of protection against severe COVID‐19 in PLWHIV group (Table 1).

**Table:** 

**Abstract P255 – Table 1**. Baseline characteristics of the HIV‐positive individuals vaccinated against COVID‐19 regarding the type of vaccination.						
N (%)	All	Comirnaty	SpikeVax	mRNA	Vaxzevria	Janssen
All	218 (100.0)	143 (65.5)	16 (7.3)	159 (72.9)	36 (16.5)	22 (10.1)
	(100.0)	(100.0)	(100.0)	(100.0)	(100.0)	(100.0)
Women	27 (12.4)	21 (14.7)	2 (12.5)	23 (14.5)	2 (5.5)	2 (9.1)
HIV VL <50 copies/mL	216 (99.1)	142 (99.3)	16 (100.0)	158 (99.4)	35 (97.2)	22 (100.0)
CD4+ <200 cells/uL	6 (2.7)	5 (3.5)	0 (0.0)	5 (3.1)	0 (0.0)	0 (0.0)
CD4+ <350 cells/uL	24 (11.0)	16 (11.2)	1 (6.2)	17 (10.7)	5 (13.9)	1 (4.5)
CD4+ <500 cells/uL	75 (34.4)	51 (35.7)	6 (37.5)	57 (35.8)	11 (30.5)	6 (27.3)
Adverse reaction after 1st vaccine dose	33 (15.1)	14 (9.8)	2 (12.5)	16 (10.1)	13 (36.1)	4 (18.2)
COVID‐19 ever	50 (22.9)	35 (24.5)	2 (12.5)	37 (23.3)	4 (11.1)	9 (40.9)
Before vaccination	18 (8.3)	12 (8.4)	2 (12.5)	14 (8.8)	0 (0.0)	2 (9.1)
After the 1st dose	4 (1.8)	0 (0.0)	0 (0.0)	0 (0.0)	1 (2.8)	3 (13.6)
After the 2nd dose	11 (5.0)	9 (6.3)	0 (0.0)	9 (5.7)	2 (5.6)	0 (0.0)
After the 3rd dose	12 (5.5)	12 (8.4)	0 (0.0)	12 (7.5)	0 (0.0)	0 (0.0)

#### P256 The impact of the pandemic on PLWHIV: issues and opportunities for rethinking PLWHIV care management

G Marchetti ^1^, S Lo Caputo^2^, S Mattioli^3^, M Sacchi^4^, L Brogonzoli^4^, R Iardino^4^

^1^Dept of Health Sciences, Clinic of Infectious Diseases, University of Milan, Aziende Socio Sanitarie Territoriali (ASST) Santi Paolo e Carlo, Milan, Italy; ^2^Dept of Infectious Diseases, Azienda Ospedaliera Universitaria, Policlinico Foggia, Foggia, Italy; ^3^Social Services, PLUS, Bologna, Italy; ^4^Research & Policies, Fondazione The Bridge, Milano, Italy

With the intention to investigate the toll that the pandemic had on non‐Covid patients and aiming to find eventual new healthcare policies for the management of PLWHIV, we designed a survey to understand the point of view of clinicians and the community, using the Computer Assisted Web Interviewing method from March to November 2021. We focused our attention on care strategies, quality of life, patients’ health status and the definition of potential tools for the betterment of healthcare services. We received answers from 32 patients' associations (62% North Italy, 18% Centre, 18% South) and 63 infectious disease centres (50% North Italy, 21% Centre, 29% South). Regarding the impact of the pandemic, in terms of fears and barriers to healthcare services, both targets, even with different perspective, had emphasised the psychosocial burden, the difficulties in keeping scheduled appointments (Figure 1) and in maintaining relations between patients and hospitals as the most impacting problems. Some services were reorganised to overcome these problems. Forty‐four percent of patients' associations implemented psychological remote support (20%), treatment dispensation deployed directly at home (14%). Clinicians underlined the importance of digital health (79%) and, to a lesser extent, the relocation of testing/diagnostics in other departments or outside the premises (38%). Overall, the results showed both points of connection and divergence between the two targets (Table 1). The results of survey demonstrated that the pandemic was indeed a tragedy but also an opportunity to improve PLWHIV management. Considering the efforts Italian policy makers are putting in planning a new healthcare model, by enhancing the role of primary care and structuring new and more widespread facilities, it is important that PLWHIV achieve a more solid balance between services provided by hospitals and, on the other hand, at local level. In this context, to facilitate the shaping of an integrated network, all the tools put in place to get through the pandemic represent insights for the future. To promote the discussions on these topics and tackle core items, all findings will be shared with multiple stakeholders in a convention meeting.

**Figure:**
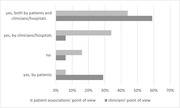
**Abstract P256 – Figure 1**. Scheduled appointments cancelled during pandemic?

**Table:** 

**Abstract P256 – Table 1**. Services provided during the pandemic and worth to keep.			
Services provided during the pandemic	by patient associations	by clinicians	and to keep after
PPE home delivery	✓	✓	
Home delivery services (such as therapies)	✓	✓	✓
Remote socialisation tools	✓		✓
Remote supportive tools (such as psychological support)	✓		✓
Digitalisation and digital health	✓	✓	✓
Linkage between hospitals and local clinics		✓	✓
Changing therapies administration		✓	
Relocation of testing/diagnostics		✓	✓

#### P257 Did COVID‐19 impact HIV control? Time to undetectable HIV viral load in newly HIV‐1 diagnosed patients before and during pandemic

J Borralho ^1^, S Casanova^1^, C Fernandes^2^, J Vasconcelos^1^, J Alves^1^, S Peres^1^, A Miranda^1^, T Baptista^1^, I Antunes^1^, F Borges^1^, J Nina^1^, K Mansinho^1^

^1^Infectious Diseases and Tropical Medicine Department, Hospital de Egas Moniz, Centro Hospitalar Lisboa Ocidental, Lisboa, Portugal; ^2^Internal Medicine, Centro Hospitalar de Leiria, Leiria, Portugal

**Background**: COVID‐19 pandemic brought difficulties to the optimal assistance of other chronic comorbidities, such as HIV infection. We hypothesised that the more extended time gap between appointments; forced telemedicine; routine laboratory restrictions and patients’ reluctance to access the hospitals may have impacted antiretroviral therapy (ART) adherence and early attain of virological suppression.

**Material and methods**: Retrospective, observational study, comparing time to undetectable viral load in naïve HIV infected patients diagnosed in a central Portuguese hospital along two discrete periods: pre‐pandemic period from 2016 to 2019 (period A); pandemic period from March 2020 to December 2021 (period B). Data were collected from medical records and laboratory results after the first medical appointment and analysed through Microsoft^®^ Excel^®^.

**Results**: There were 725 HIV patients newly admitted to our centre through 2016 to 2021, of whom 490 newly diagnosed and naïve to ART (354 in period A; 136 in period B). Comparing period A to period B: male genre was predominant in both groups (74% vs 79%); mean age was 44 versus 38 years; 50% versus 42% were Portuguese native; 17% versus 26% were Brazilian native; mean baseline TCD4+ was 434/mm^3^ versus 416/mm^3^ and opportunistic infections were the reason of HIV diagnosis in 9% versus 9%, respectively. All patients started on ART and mean time to attain plasmatic undetectability was 5.9 versus 3.5 months.

**Conclusions**: Despite the several constraints on the optimal follow‐up of HIV patients along the pandemic strikes, our results show that the patients followed along the pandemic period reached undetectability prior to those in the pre‐pandemic phase. Some factors may have impacted these data, such as a possible dynamic differential on the first‐line ART regimens choices, with a rising experience with INSTI. Furthermore, this data may somehow glimpse the efforts made by the clinicians in order not to choose one pandemic above other.

### COVID‐19: Novel Therapeutics

#### P258 Retrospective study comparing outcome, safety and virological clearance at day 7 in patients with mild‐moderate COVID‐19 disease treated with molnupiravir, nirmatrelvir/ritonavir and short‐course of remdesivir

T Beringheli, F Bai, V Vitaletti, F Molà, A Copes, N Gemignani, S Pettenuzzo, D Barbanotti, G Mulè, R Castoldi, B Varisco, R Nardo, L Lundgren, L Albertini, M Augello, L Biasioli, C Falcinella, D Tomasoni, G Marchetti, A d'Arminio Monforte

Clinic of Infectious Diseases, Department of Health Sciences, Università degli Studi di Milano, San Paolo Hospital, Milan, Italy

**Background**: Antiviral therapies against SARS‐CoV‐2 (molnupiravir‐MNP‐nirmatrelvir/ritonavir‐PAX‐remdesivir‐RDV) are indicated in mild‐moderate outpatients within 5 to 7 days from symptoms onset, ≥1 risk factor for progression. These treatments demonstrated efficacy in randomised trials, but real‐life data are lacking, especially in vaccinated patients [1,2]. We compared the efficacy, safety and virological clearance (VC) at day 7 (T7) post‐treatment with MNP, PAX and RDV in SARS‐CoV‐2‐infected patients at high risk (HR).

**Material and methods**: Retrospective study enrolling HR patients (BMI ≥30, chronic renal failure, immunodeficiencies, cardio‐cerebrovascular, chronic pulmonary or neurological disease, age ≥65) with mild‐moderate COVID‐19 between January and June 2022. Patients hospitalised for COVID‐19 were excluded. Treatments included: PAX (100 mg/300 mg BID) or MNP (800 mg BID) for 5 days; remdesivir (200 mg IV day 1, 100 mg day 2 to 3). A nasopharyngeal swab and blood tests were performed at T7. Recovery was defined by 72 hours asymptomatic and a PCR/antigenic‐negative nasopharyngeal swab (VC). Kruskal‐Wallis and Chi‐squared test were used.

**Results**: Seventy‐eight of 178 (43.8%) received MNP, 44/178 (24.7%) PAX and 56/178 (31.5%) RDV. Thirty‐two (18%) patients were either unvaccinated or incompletely vaccinated (Table 1). Patients treated with PAX were younger, presented more frequently immunodeficiency; RDV was used more in patients hospitalised for diseases other than COVID‐19. Few mild adverse events were recorded. Recovery was obtained in 70/73 (95.9%) patients with MNP, 31/32 (96.9%) PAX and 49/53 (92.5%) RDV. Only two (1.3%) patients were hospitalised (one MNP, one RDV); six (3.8%) died (two MNP, one PAX, three RDV). Interestingly, patients receiving PAX presented a higher proportion of VC at T7, and a shorter time to VC, compared to MNP/RDV (Table 1). Data were confirmed after adjustment for age and immunodeficiency (AOR 2.783 PAX vs MNP, 95% CI 0.977 to 7.928, p = 0.055; AOR 1.425 RDV vs MNP, 95% CI 0.633 to 3.206, p = 0.392). Early (<3 days from symptoms) treatment and previous vaccination did not result in higher rate of VC at T7.

**Table:** 

**Abstract P258 – Table 1. **Demographic and clinical characteristics of patients with mild COVID‐19, according to anti‐SARS‐CoV‐2 antiviral treatment.					
Characteristics	Study population	MNP	PAX	RDV	p‐value
	N = 178	N = 78	N = 44	N = 56	
		(43.8%)	(24.7%)	(31.5%)	
Age [years], median (IQR)	74 (58 to 82)	78 (66 to 84)	59 (49 to 78)	73 (63 to 83)	<0.001
Male sex, n (%)	100 (56.2%)	52 (67.5%)	20 (45.5%)	28 (50%)	0.031
COVID‐19 vaccination, n (%)					0.661
No	32 (18%)	14 (17.9%)	6 (18.8%)	12 (37.5%)	
Yes	144 (80.9%)	64 (82.1%)	36 (25%)	44 (30.6%)	
(complete schedule, <120 days)a					
Unknown	2 (1.1%)	0	2 (4.5%)	0	
Risk factor, n (%)					
Age >65 years	105 (59%)	55 (70.5%)	12 (27.3%)	38 (67.9%)	<0.001
BMI >30	20 (11.2%)	7 (9%)	6 (13.6%)	7 (12.5%)	0.689
Cardiovascular disease	75 (42.1%)	42 (53.8%)	8 (18.2%)	25 (44.6%)	<0.001
COPD or other respiratory disease	38 (21.3%)	15 (19.2%)	10 (22.7%)	13 (23.2%)	0.829
Neurological disease	11 (6.2%)	4 (5.1%)	2 (4.5%)	5 (8.9%)	0.582
Diabetes mellitus	34 (19.1%)	14 (17.9%)	5 (11.4%)	15 (26.8%)	0.141
Chronic kidney failure	10 (5.6%)	7 (9%)	0	3 (5.4%)	0.117
Cancer	12 (6.7%)	5 (6.4%)	3 (6.8%)	4 (7.1%)	0.986
Immunodeficiency	27 (15.2%)	8 (10.3%)	12 (27.3%)	7 (12.5%)	0.034
Treatments for COVID‐19, n (%)					
Heparin	16 (9%)	3 (4%)	2 (5.6%)	11 (20.8%)	0.004
Corticosteroid therapy	4 (2.2%)	1 (1.3%)	1 (2.8%)	2 (3.8%)	0.671
Symptoms, n (%)					<0.001
None	14 (7.9%)	2 (2.6%)	1 (2.3%)	11 (19.6%)	
Yes	164 (92.1%)	76 (97.4%)	43 (97.7%)	45 (80.4%)	
Type of symptoms					
Fever	76 (42.7%)	36 (46.2%)	21 (47.7%)	19 (33.9%)	0.273
Cough	85 (47.8%)	38 (48.7%)	22 (50%)	25 (44.6%)	0.846
Sore throat	34 (19.1%)	15 (19.2%)	14 (31.8%)	5 (8.9%)	0.015
Headache	15 (8.4%)	4 (5.1%)	6 (13.6%)	5 (8.9%)	0.264
Joint/muscle pain	14 (7.9%)	6 (7.7%)	5 (11.4%)	3 (5.4%)	0.540
Gastrointestinal disorders	7 (3.9%)	3 (3.8%)	2 (4.5%)	2 (3.6%)	0.968
Dyspnoea	16 (9%)	3 (3.8%)	3 (6.8%)	10 (17.9%)	0.017
Fatigue	32 (18%)	16 (20.5%)	10 (22.7%)	6 (10.7%)	0.221
Cold	13 (7,3%)	10 (12.8%)	10 (22.7%)	3 (5.4%)	0.037
Oxygen saturation >94%, median (IQR)	155 (87.1%)	97 (96 to 98)	97 (96 to 98)	96 (95 to 98)	0.180
Setting, n (%)					<0.001
Outpatient service	148 (83.1%)	75 (96.2%)	41 (93.2%)	32 (57.1%)	
Hospitalisation for diseases other than COVID‐19	30 (16.9%)	3 (3.8%)	3 (6.8%)	24 (42.9%)	
Days from symptom onset to antiviral treatment, median (IQR)	3 (2 to 4)	3 (2 to 4)	3 (2 to 4)	2 (1 to 4)	0.958
Days from symptom onset to virological clearance, median (IQR)	13 (10 to 18)	15 (11 to 18)	11 (9 to 15)	13 (9 to 19)	0.015
Outcome, n (%)	N = 158	N = 73	N = 32	N = 53	0.850
Recovery	150 (94.9%)	70 (95.9%)	31 (96.9%)	49 (92.5%)	
Hospitalisation	2 (1.3%)	1 (1.4%)	0	1 (1.9%)	
Death	6 (3.8%)	2 (2.7%)	1 (3.1%)	3 (5.7%)	
Adverse events, n (%)	N = 148	N = 67	N = 30	N = 51	0.321
No	144 (97.3%)	66 (98.5%)	28 (93.3%)	50 (98%)	
Yes	4 (2.7%)	1 (1.5%)	2 (6.7%)	1 (2%)	
Virological clearance at day 7 from symptoms onset, n (%)	N = 133	N = 60	N = 26	N = 47	0.053
No	82 (61.7%)	42 (70%)	11 (42.3%)	29 (61.7%)	
Yes	51 (38.3%)	18 (30%)	15 (57.7%)	18 (38.3%)	
Calendar period, n (%)					<0.001
Jan to Feb 2022	62 (34.8%)	35 (44.9%)	3 (4.8%)	24 (42.9%)	
Mar to Apr 2022	58 (32.6%)	33 (56.9%)	17 (38.6%)	8 (14.3%)	
May to Jun 2022	58 (32.6%)	10 12.8%)	24 (54.5%)	24 (42.9%)	

^a^Vaccination: no dose: 15, 8.4% ‐ yes, only 1 dose: 4, 2.2% ‐ complete cycle: 20, 11.2% ‐ first booster dose: 136, 76.4% ‐ second booster dose: 1, 0.6%.

Kruskal Wallis and Chi‐squared test for comparison among the three groups, as appropriate.

**Conclusions**: SARS‐COV‐2 antiviral treatments are an excellent therapeutic strategy in HR patients with mild‐moderate disease. PAX seems characterised by a higher proportion of patients with both clinical and VC as early as 7 days after treatment, confirming its likely superiority in indirect comparisons.

**References**

1. Najjar‐Debbiny R, Gronich N, Weber G, Khoury J, Amar M, Stein N, et al. Effectiveness of Paxlovid in reducing severe COVID‐19 and mortality in high risk patients. Clin Infect Dis. 2022 Jun 2;ciaci443. doi: 10.1093/cid/ciac443.

2. Lee TC, Morris AM, Grover SA, Murthy S, McDonald EG. Outpatient therapies for COVID‐19: how do we choose? Open Forum Infect Dis. 2022;9:ofac008.

#### P259 Vaccination and anti‐SARS‐CoV‐2 treatment reduce disease progression: a real‐life experience

A De Vito ^1^, A Colpani^1^, L Saderi^2^, B Zauli^1^, A Bitti^1^, M Fois^1^, C Di Castri^1^, M Meloni^1^, M Puci^2^, S Bacciu^1^, V Fiore^1^, I Maida^1^, S Babudieri^1^, G Sotgiu^2^, G Madeddu^1^

^1^Unit of Infectious Diseases, Department of Medicine, Surgery and Pharmacy, University of Sassari, Sassari, Italy; ^2^Clinical Epidemiology and Medical Statistics Unit, Department of Medicine, Surgery and Pharmacy, University of Sassari, Sassari, Italy

**Background**: After the identification of the first SARS‐CoV‐2‐infected patient, several drugs have been prescribed, mainly in patients with risk factors increasing the probability of disease progression. Three antivirals (molnupiravir, nirmatrelvir/r, remdesivir) and two monoclonal antibodies (casirivimab/imdevimab and sotrovimab) are currently available in Italy. Aim of the present study was to evaluate the association between risk factors and disease progression.

**Materials and methods**: A single‐centre retrospective cohort study was performed. Patients with a confirmed diagnosis of SARS‐CoV‐2 infection between 1 January 2022 and 10 May 2022 were recruited. Disease progression was defined by the prescription of oxygen therapy. Preventive treatment was prescribed in patients with recent symptom onset (≤5 days), no need of oxygen supplementation, and risk factors for disease progression. Student t‐test, chi‐square, or Fischer exact tests were used to assess differences for quantitative and qualitative variables. In addition, a logistic regression analysis was performed to test the association between the collected variable and the outcome disease progression. A two‐tailed p‐value less than 0.05 was considered statistically significant. All statistical analyses were performed with STATA version 17 (StataCorp, Texas, USA).

**Results**: One thousand, one hundred and eighteen patients were enrolled: a disease progression was recorded in 363 (32.5%). They showed an advanced age (74.8±13.1 vs 67.9±17.1, p < 0.0001), a higher burden of comorbidities (CCI score 5.51±2.65 vs 4.92±2.65, p < 0.001), lower vaccination coverage (56.5 vs 88, p < 0.001), and had fever and/or dyspnoea. Patients exposed to antivirals or monoclonal antibodies had a lower risk of disease progression (Figure 1). The regression analysis showed an increased risk of clinical severity with the increasing age, chronic respiratory disease, haematological tumour, and fever and dyspnoea. Preventive therapy for SARS‐CoV‐2 was associated with a lower risk of progression (Table 1). Patients treated with nirmatrelvir/r did not show any disease progression; of note, they were significantly younger (59.1 vs 70.6 years; p < 0.0001).

**Conclusions**: Vaccination and antiviral and monoclonal antibodies reduce the risk of disease progression in SARS‐CoV‐2 infected patients. Of note, patients enrolled in our study were older and had a higher comorbidity burden when compared with those enrolled in clinical trials [1‐3].

**Figure:**
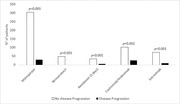
**Abstract P259 – Figure 1**. Prescription of antiviral treatment and monoclonal antibodies in 1118 patients affected by SARS‐CoV‐2 with and without disease progression.

**Table:** 

**Abstract P259 – Table 1**. Logistic regression analysis to assess the relationship between sociodemographic, clinical variables, and disease progression in 1118 patients infected by SARS‐CoV‐2.				
Variables	Univariate	Analysis	Multivariate	Analysis
	OR (95% CI)	p‐value	OR (95% CI)	p‐value
Age	1.03 (1.02 to 1.04)	<0.0001	1.04 (1.02 to 1.05)	<0.0001
Males	1.10 (0.86 to 1.42)	0.44	−	−
BMI >30 kg/m^2^	1.24 (0.93 to 1.66)	0.14	−	−
CKD	1.24 (0.88 to 1.76)	0.22	−	−
Immunodeficiency	0.89 (0.64 to 1.26)	0.53	−	−
Decompensated diabetes	1.61 (1.11 to 2.33)	0.01	1.03 (0.59 to 1.81)	0.91
Chronic liver disease	1.40 (0.86 to 2.28)	0.17	−	−
Chronic respiratory disease	1.69 (1.24 to 2.28)	0.001	1.73 (1.11 to 2.69)	0.02
Neurological disorders	1.52 (1.12 to 2.05)	0.006	1.42 (0.93 to 2.16)	0.10
Oncological disease	0.72 (0.51 to 1.02)	0.06	−	−
Haematological tumours	1.61 (0.99 to 2.63)	0.06	2.82 (1.41 to 5.65)	0.003
Cardiovascular diseases	1.22 (0.95 to 1.58)	0.12	−	−
Number of comorbidities	1.20 (1.09 to 1.31)	<0.0001	−	−
CCI	1.09 (1.04 to 1.14)	<0.001	−	−
4C‐score	1.36 (1.29 to 1.43)	<0.0001	−	−
Vaccine (at least two doses)	0.18 (0.13 to 0.24)	<0.0001	0.22 (0.15 to 0.33)	<0.0001
At least one symptom	3.80 (2.34 to 6.19)	<0.0001	−	−
Fever	1.92 (1.49 to 2.47)	<0.0001	2.20 (1.53 to 3.15)	<0.0001
Dyspnoea	16.49 (11.95 to 22.76)	<0.0001	13.24 (8.99 to 19.50)	<0.0001
Any antiviral	0.11 (0.08 to 0.16)	<0.0001	−	−
Molnupiravir	0.14 (0.09 to 0.21)	<0.0001	0.13 (0.08 to 0.21)	<0.0001
Nirmatrelvir/ritonavir	−	−	−	−
Remdesivir	0.36 (0.15 to 0.86)	0.02	0.18 (0.06 to 0.52)	0.002
Any monoclonal antibodies	0.35 (0.24 to 0.52)	<0.0001	−	−
Sotrovimab	0.27 (0.14 to 0.52)	<0.0001	0.37 (0.16 to 0.84)	0.02
Casirivimab/imdevimab	0.49 (0.32 to 0.75)	<0.0001	0.50 (0.28 to 0.89)	0.02

**References**

1. Fischer W, Eron JJ, Holman W, Cohen MS, Fang L, Szewczyk LJ, et al. Molnupiravir, an oral antiviral treatment for COVID‐19. Preprint. medRxiv. 2021 Jun 17;2021.06.17.21258639. doi: 10.1101/2021.06.17.21258639.

2. Hammond J, Leister‐Tebbe H, Gardner A, Abreu P, Bao W, Wisemandle W, et al. Oral nirmatrelvir for high‐risk, nonhospitalized adults with Covid‐19. N Engl J Med. 2022;386:1397‐408.

3. Gottlieb RL, Vaca CE, Paredes R, Mera J, Webb BJ, Perez G, et al. Early remdesivir to prevent progression to severe Covid‐19 in outpatients. N Engl J Med. 2022;386:305‐15.

#### P260 MP3 pulses COVID 19 trial: effect of intravenous pulses of methylprednisolone 250 mg versus dexamethasone 6 mg in hospitalised adults with severe COVID‐19 pneumonia

J Abadía Otero ^1^, L Corral Gudino^2^, I Cusacovich^3^, J Martín González^4^, A Muela Molinero^5^, R González Fuentes^3^, Á Ruiz de Temiño^2^, E Tapia Moral^3^, F Cuadrado Medina^2^, M Martín Asenjo^3^, P Miramontes González^2^

^1^Internal Medicine/Infectious Diseases, Río Hortega University Hospital, Valladolid, Spain; ^2^Internal Medicine, Río Hortega University Hospital, Valladolid, Spain; ^3^Internal Medicine, Hospital Clinico Universitario de Valladolid, Valladolid, Spain; ^4^Internal Medicine, Hospital Universitario de Salamanca, Salamanca, Spain; ^5^Internal Medicine, Hospital de León, León, Spain

**Background**: The efficacy and safety of high doses glucocorticoids for the treatment of COVID‐19 patients has shown mixed outcomes in controlled trials and observational studies [1‐3]. We try to evaluate the effectiveness of methylprednisolone bolus versus dexamethasone 6 mg in patients with severe COVID‐19.

**Material and methods**: Randomised, open‐label, controlled trial conducted between February and August 2021 at four hospitals in Spain. Trial was suspended after first interim analysis since investigators considered that continuing the trial would be futile. Patients were randomly assigned in aw 1:1 ratio to receive dexamethasone 6 mg once daily for up to 10 days or methylprednisolone 250 mg once daily for 3 days.

**Results**: Of the 128 randomised patients, 125 were analysed (mean age 60 ± 17 years; 82 males [66%]). Mortality at 28 days was 4.8% in the 250 mg methylprednisolone group versus 4.8% in the 6 mg dexamethasone group (absolute risk difference, 0.1% [95% CI ‐8.8 to 9.1%]; p = 0.98). The post hoc added composite outcome of mortality at 90 days or intubation was 15.9% in the 250 mg methylprednisolone group versus 15% in the 6 mg dexamethasone group (absolute risk difference, ‐0.9% [95% CI ‐13.8 to 12.3%]; p = 0.83). Hyperglycaemia was more frequent in the methylprednisolone group, at 27.0 versus 8.1% (absolute risk difference, ‐18.9% [95% CI ‐31.8 to ‐ 5.6%]; p = 0.007).

**Conclusions**: Among severe but not critical patients with COVID‐19, 250 mg/d for 3 days of methylprednisolone compared with 6 mg/d for 10 days of dexamethasone did not result in a decrease in mortality or intubation.

**References**

1. RECOVERY Collaborative Group; Horby P, Lim WS, Emberson JR, Mafham M, Bell JL, Linsell L, et al. Dexamethasone in hospitalized patients with Covid‐19. N Engl J Med. 2021;384:693‐704.

2. Papamanoli A, Yoo J, Grewal P, Predun W, Hotelling J, Jacob R, et al. High‐dose methylprednisolone in nonintubated patients with severe COVID‐19 pneumonia. Eur J Clin Invest. 2021;51:e13458.

3. Stahn C, Buttgereit F. Genomic and nongenomic effects of glucocorticoids. Nat Clin Pract Rheumatol. 2008;4:525‐33.

### Monkeypox Virus

#### P261 Clinical description of 50 cases of monkeypox virus (MKPV) infection among MSM in an HIV/PrEP French clinic

E Rubenstein ^1^, M Sarda^1^, C Lascoux‐Combe^1^, C Pintado^1^, J Zeggagh^1^, D Ponscarme^1^, M Tateo^1^, S Chawki^1^, G Liegeon^1^, J Gras^1^, M Lafaurie^1^, N De Castro^1^, D Descamps^2^, J Molina^1^

^1^Infectious Diseases, Saint Louis Hospital, Paris, France; ^2^Virology, Bichat Hospital, Paris, France

**Introduction**: While monkeypox infection was mostly localised in Western and Central Africa [1], multiple cases have been reported since May 2022 in Europe, mostly affecting men who have sex with men (MSM). We report our experience with 50 consecutive patients diagnosed with MKPV infection.

**Method**: This prospective observational study was conducted in the Infectious Diseases Department of Saint Louis University Hospital in Paris, from 1 June to 30 June 2022. We enrolled all patients with confirmed MKPV infection using specific PCR test. We recorded patients’ epidemiological, demographic and clinical data.

**Results**: Fifty patients were enrolled, all MSM, median age 38.4 years old (SD 9.2). Nine patients (18%) were infected with HIV, with CD4 rates over 600/mm^3^ and undetectable viral load, and 33 (66%) were taking PrEP. In 94% cases, sexual transmission was most probable, in 12 cases (24%) after a festive event, and eight patients (16%) reported contact with a person infected with MKPV. The average number of sexual partners was 17 in 3 months (SD 19). Eight patients (16%) received smallpox vaccine in their childhood. Thirty patients (60%) reported fever, 18 (36%) headaches, 30 (60%) muscle pain, and 24 (48%) had a sore throat. All had muco‐cutaneous symptoms: 46 (92%) vesicules or pustules, and 30 (60%) ulcerative lesions (Figure 1). A pre‐existing rash occurred in seven patients (14%). One patient had isolated penis oedema. On average, there were 12 cutaneous lesions (SD 16.5): on the face (13 patients, 26%), the palms or soles (five patients, 10%), in the mouth (one patient, 2%), on the genitals (27 patients, 54%), the anus (19 patients, 38%), or on other body parts (29 patients, 58%). There was cervical or inguinal lymphadenopathy in 36% and 54% cases respectively, and six patients (12%) had erythemato‐pultaceous angina. One patient necessitated hospitalisation because of compressive cervical adenopathy. Median disease duration was 16.2 days (SD 4.7).

**Figure:**
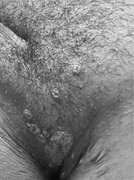
**Abstract P261 – Figure 1. **Genital umbilicated pustules with a positive MKPV PCR testing.

**Discussion**: In 1 month, we observed an outbreak of MKPV infection with various clinical presentations. Information among MSM, particularly those with HIV infection or PrEP is critical for early diagnosis and contact tracing. Vaccination of people at risk should be a priority to contain this epidemic.

**Reference**

1. McCollum A, Damon I. Human monkeypox. Clin Infect Dis. 2014;58:260‐7.

#### P262 First monkeypox cases in patients living with HIV from Romania

C Oprea ^1^, I Ianache^1^, S Piscu^1^, A Paun^1^, D Topa^1^, G Tardei^2^, C Popescu^1^, S Florescu^1^

^1^Infectious Diseases, Victor Babes Hospital for Infectious and Tropical Diseases, Bucharest, Romania; ^2^Virology and Molecular Biology, Victor Babes Hospital for Infectious and Tropical Diseases, Bucharest, Romania

**Background**: On 29 June 2022, ECDC reported 4178 cases of monkeypox (MPXV) from 31 countries throughout the European region. The aim of our study was to present the epidemiological, clinical characteristics and outcomes of the first three cases diagnosed with MPXV in Romania.

**Methods**: Diagnosis was confirmed in all cases by positive RT‐PCR test for MPXV in fluid from the skin lesions, nasopharyngeal swabs, urine and blood samples.

**Table:** 

**Abstract P262** – **Table 1**. Clinical manifestations in three MSM with HIV and MPXV.											
	Skin lesion	Skin lesion	Skin lesion	Skin lesion	Skin lesion	Skin lesion	Skin lesion	Skin lesion			
	Penile	Scrotum	Torso	Arms	Legs	Trunk	Facial	Rectal	Rectal bleeding	Adenopathy	White deposit on the pharynx
Case 1	+	++	+++	+	+	+	+	++	++	Cervical, inguinal	+
Case 2	++	+++	+++	+	+	+	+	++	++	Cervical, inguinal	+
Case 3	+	+	+	+	+	+	−	−	−	Inguinal	+

**Results**: We present three HIV‐positive young MSM, 26, 36 and 40 years old, admitted with MPXV to Victor Babes Hospital, Bucharest, Romania, in June 2022. Two patients had serological markers for syphilis and admitted to be involved in chemsex practices and one was diagnosed with HAV with faecal‐oral transmission through sexual activity 2 months prior to the current admission. The onset of the disease was 4 to 6 days before admission with fever and chills (three patients), sore throat (three), vesiculo‐pustular rash (three) and severe rectal pain and bleeding (three). The rash was seen predominantly in the genital area (penile, scrotum), perianal and on the torso, with few lesions on the neck, trunk, and upper and lower limbs in all three cases. Marked hyperaemia of the pharynx, with white deposits and enlarged cervical and inguinal lymph nodes was observed in all cases (Table 1). CD4 cell counts were 998/μL, 275/ μL and 1000 /μL and all patients were under effective cART with undetectable HIV VL. There were no other biological abnormalities apart from a discrete inflammatory syndrome. Real‐time PCR for MPXV showed lower Ct values in skin lesions and throat swabs compared to blood and urine, remaining positive for at least 1 week. The disease was mild to moderate in all cases, but the patient with lower CD4 cell count had an increased number of skin lesions, a longer evolution and developed cellulitis of the thigh.

**Conclusions**: HCPs must be aware of the diagnosis of monkeypox in all patients with typical rash and risky sexual behaviours. Isolation and effective contact tracing are important pillars for the strategy to control MPXV outbreak. Pre‐exposure vaccination for MPXV must be prioritised for high‐risk groups.

